# Loss of genes related to Nucleotide Excision Repair (NER) and implications for reductive genome evolution in symbionts of deep-sea vesicomyid clams

**DOI:** 10.1371/journal.pone.0171274

**Published:** 2017-02-15

**Authors:** Shigeru Shimamura, Takashi Kaneko, Genki Ozawa, Mamiko Nishino Matsumoto, Takeru Koshiishi, Yoshihiro Takaki, Chiaki Kato, Ken Takai, Takao Yoshida, Katsunori Fujikura, James P. Barry, Tadashi Maruyama

**Affiliations:** 1 Department of Marine Biodiversity Research, Japan Agency for Marine-Earth Science and Technology, 2–15, Natsushima-cho, Yokosuka-shi, Kanagawa, Japan; 2 Department of Subsurface Geobiological Analysis and Research, Japan Agency for Marine-Earth Science and Technology, Natsushima-cho, Yokosuka-shi, Kanagawa, Japan; 3 Tokyo College of Biotechnology, Kitakoujiya, Ota-ku,Tokyo, Japan; 4 Kitasato University, School of Marine Biosciences, Kitasato Minami-ku Sagamihara-shi Kanagawa, Japan; 5 Monterey Bay Aquarium Research Institute, Moss Landing, California, United States of America; 6 Research and Development Center for Submarine Resources, Japan Agency for Marine-Earth Science and Technology, Natsushima-cho, Yokosuka-shi, Kanagawa, Japan; Pusan National University, REPUBLIC OF KOREA

## Abstract

Intracellular thioautotrophic symbionts of deep-sea vesicomyid clams lack some DNA repair genes and are thought to be undergoing reductive genome evolution (RGE). In this study, we addressed two questions, 1) how these symbionts lost their DNA repair genes and 2) how such losses affect RGE. For the first question, we examined genes associated with nucleotide excision repair (NER; *uvrA*, *uvrB*, *uvrC*, *uvrD*, *uvrD* paralog [*uvrD*p] and *mfd*) in 12 symbionts of vesicomyid clams belonging to two clades (5 clade I and 7 clade II symbionts). While *uvrA*, *uvrD*p and *mfd* were conserved in all symbionts, *uvrB* and *uvrC* were degraded in all clade I symbionts but were apparently intact in clade II symbionts. *UvrD* was disrupted in two clade II symbionts. Among the intact genes in *Ca*. Vesicomyosocius okutanii (clade I), expressions of *uvrD* and *mfd* were detected by reverse transcription-polymerase chain reaction (RT-PCR), but those of *uvrA* and *uvrDp* were not. In contrast, all intact genes were expressed in the symbiont of *Calyptogena pacifica* (clade II). To assess how gene losses affect RGE (question 2), genetic distances of the examined genes in symbionts from *Bathymodiolus septemdierum* were shown to be larger in clade I than clade II symbionts. In addition, these genes had lower guanine+cytosine (GC) content and higher repeat sequence densities in clade I than measured in clade II. Our results suggest that NER genes are currently being lost from the extant lineages of vesicomyid clam symbionts. The loss of NER genes and *mutY* in these symbionts is likely to promote increases in genetic distance and repeat sequence density as well as reduced GC content in genomic genes, and may have facilitated reductive evolution of the genome.

## Introduction

Vesicomyid clams, commonly found in large, dense aggregations at deep-sea methane seeps and hydrothermal vents [1, 2] have single thioautotrophic gammaproteobacterial symbionts in their gill epithelial cells. Reconstruction of phylogenies using ribosomal RNA sequences indicates that the symbionts of vesicomyids comprise two phylogenetic clades (clade I and II) [3]. Genome sequences of symbionts are known from 2 vesicomyid species, *Phreagena* (formerly *Calyptogena*) *okutanii* (*Ca*. Vesicomyosocius okutanii, Vok, clade I) and *Calyptogena magnifica* (*Ca*. Ruthia magnifica, Rma, clade II), with sizes of 1.02 and 1.16 Mb, respectively [4, 5]. Comparative genome analysis has shown that their genomes are in an active phase of reductive genome evolution (RGE) [3, 6].

Kuwahara et al. (2008) proposed that genes for DNA repair and recombination were lost from the ancestral symbiont genome after it had established intracellular symbiosis with the ancestral host clam [6]. Vesicomyid symbionts belonging to clade I (symbionts of *P*. *okutanii*, *P*. *soyoae* [formerly *Calyptogena soyoae*], *Akebiconcha kawamurai* [formerly *C*. *kawamurai*], *Calyptogena laubieri* and *P*. *kilmeri* [formerly *C*. *kilmeri*]) do not possess the *mutY* gene, known to be involved in the repair of a transversion mismatch mutation from G·A to G·C [7]. In contrast, *mutY* is intact in nearly all vesicomyid symbionts examined from clade II (*C*. *pacifica*, *C*. *fausta*, *C*. *nautilei*, *Pliocardia stearnsii* [formerly *C*. *stearnsii*], *Isorropodon fossajaponicum* [formerly *C*. *fossajaponica*] and *Abyssogena phaseoliformis* [formerly *C*. *phaseoliformis*]). One exception is the clade II symbiont Rma from *C*. *magnifica*, which lacks *mutY* [3].

Loss of DNA repair genes is thought to increase the rate of nucleotide substitution and also increase the repeat sequences that drive RecA-independent deletion in genomes [6]. Intact *mutY* genes in most clade II symbionts likely helped maintain the higher GC content reported for their ribosomal RNA genes and several other genes, compared to clade I symbionts [3, 8]. Thus, even though the loss of a gene may be a spontaneous event, it necessarily affects subsequent genome evolution, including a reduction in its GC content [3].

The nucleotide excision repair (NER) system functions to repair large DNA adducts (e.g., pyrimidine dimers) or bulky damage to DNA [9, 10], as might occur in response to UV light exposure near the sea surface [11]. Although deep-sea environments are devoid of UV light, significant DNA adducts or other DNA damage have been reported in mussels from deep-sea vents, suggesting the presence of mutagens such as heavy metals, which are known to be abundant at hydrothermal vents and methane seeps [12]. In addition, some vent environments have higher levels of radioactivity than non-vent, abyssal sites [13]. Thus, exposure to potential mutagens may be typical of many deep-sea environments and DNA repair mechanisms, including the NER system, may be necessary to maintain the genomes of free-living, deep-sea bacteria.

Genes involved in the NER system are not uniformly present in symbionts of vesicomyid clams inhabiting vents and seeps. For example, *uvrC* (a NER-function gene) is found in the genome of Rma but has been lost from Vok, [6]. This observation suggests that the NER system is being lost from the extant lineage of vesicomyid clam symbionts.

Two different pathways, global repair (GR) and transcription coupled repair (TCR), contribute to genome repair in the NER system [10]. In GR, the mutation site is recognized by a complex (UvrA_2_B) of UvrA (ATP-dependent recognition of damaged DNA) and UvrB (helicase), which produce an SOS response to promote repair [14, 15]. Both the 3’- and 5’- termini of the recognized mutation site are incised by UvrC endonuclease. After incision, UvrD (DNA helicase II) is required to release the oligonucleotide and to fill the resulting gap with DNA polymerase I [10]. In TCR, the coupling factor protein Mfd (mutation frequency decline) promotes repair by removing stalled RNA polymerase from the DNA lesion, then recruits UvrA_2_B. After this first step of recognizing the mutation site, the subsequent repair process is the same as occurs in GR [9].

Here, we address two questions, 1) how did vesicomyid symbionts lose NER-related genes, and 2) how does the loss of both NER genes and *mutY* affect genome evolution? For the first question, we assessed the presence of NER-related genes [*uvrA*, *uvrB*, *uvrC*, *uvrD*, *uvrD*_paralog (*uvrD*p) and *mfd*] in symbionts of 12 vesicomyid clams by using polymerase chain reaction (PCR) with newly designed primers. To address question 2, we analyzed the genetic distances among NER-related genes and several other genes from a closely related symbiont of the symbiotic deep-sea mussel, *Bathymodiolus septemdierum*. These data were used as a proxy for substitutional mutation, and compared among symbionts with and without the genes for NER and other DNA repair pathways. The GC contents and repeat sequence densities of these genes in symbionts with or without NER-related genes and some other DNA-repair genes were also analyzed.

## Results

### NER-related genes

All *uvrA*s in the 12 clam symbionts examined had the same length (2817 bp, including start and stop codons, coding 938 amino acids; S1 Fig), but varied in amino acid identities by 87 to 100%. Their amino acid sequences were similar to that of *Escherichia coli* (940 amino acids; accession number = BAE78060) [16]. Two consensus ATPase sequences of bacterial UvrAs [17] were also found to be well conserved in *uvrA*s from vesicomyid clam symbionts (S1 Fig), suggesting that they code functional UvrAs.

**Fig 1:**
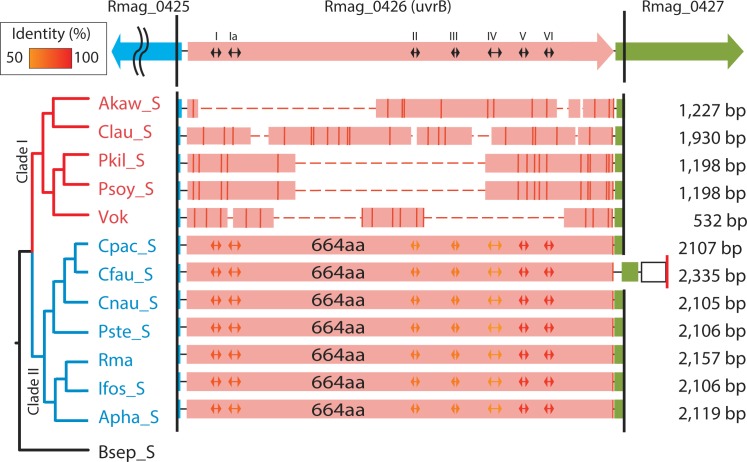
Deletion profiles of *uvrB* in the PCR products of vesicomyid clam symbionts. ORFs of Rma are shown at the top: Rmag_0425, aminotransferase gene; Rmag_0427, Radical SAM domain protein. Red bars in the columns indicate stop codons. Horizontal dashed lines indicate the lost portion of the fragmented gene. The additional vertical red line on the right side of the column of Cfau_S indicates the position of the redesigned PCR primer (S2 Table). Lengths for PCR products are shown on the right side of each column. ORFs of *uvrB* in clade I symbionts are highly fragmented. Bidirectional arrows in columns indicate the consensus helicase motifs. Color gradient of the bidirectional arrows indicate the identity to that of *E*. *coli*. Symbionts are abbreviated as follows: Akaw_S, *A*. *kawamurai* symbiont; Clau_S, *C*. *laubieri* symbiont; Pkil_S, *P*. *kilmeri* symbiont; Psoy_S, *P*. *soyoae* symbiont; Vok, *Ca*. Vesicomyosocius okutanii (*P*. *okutanii* symbiont); Cpac_S, *C*. *pacifica* symbiont; Cfau_S, *C*. *fausta* symbiont; Cnau_S, *C*. *nautilei* symbiont; Pste_S, *P*. *stearnsii* symbiont; Rma, *Ca*. Ruthia magnifica (*C*. *magnifica* symbiont); Ifos_S, *I*. *fossajaponicum* symbiont; Apha_S, *A*. *phaseoliformis* symbiont; Bsep_S, *Bathymodiolus septemdierum* symbiont.

Their *uvrBs*, on the other hand, were largely deleted from the clade I symbionts (Fig 1). In clade II, *uvrB* lengths were all 1995 bp (coding 664 amino acids) and their amino acid sequences showed only minor variation among symbiont taxa (amino acid identity = 94–98%; Fig 1) and were comparable to *E*. *coli* (678 aa.; BAA35437) [18]. Consensus helicase motifs of bacterial UvrBs were mostly found in the coded amino acid sequences of the intact *uvrBs* in clade II (Figs 1 and S2), suggesting that they were functional. In clade I symbionts *uvrB* was degraded and its deletion pattern differed among symbionts, except for a nearly identical pattern for the symbionts of 2 sibling species, *P*. *soyoae* and *P*. *kilmeri* (Figs 1 and S2).

Open reading frames (ORF) of *mfd* were found in all of the amplicons from the examined symbionts, which had similar lengths (3426–3441 bp) and only slight variation in amino acid coding (1141–1146 aa) (S3 Fig). Amino acid identities were similar among symbiont taxa (identity = 79–100%) and to that in *E*. *coli* (1148 aa; BAA35929)[19]. Seven helicase motifs reported in the Mfd of *E*. *coli* [19] were found to be well conserved (S3 Fig), suggesting that they are still functional.

**Fig 2:**
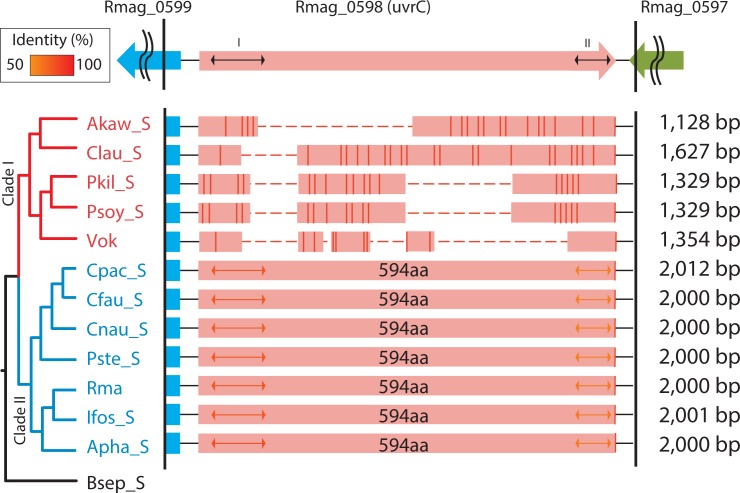
Deletion profiles of *uvrC* in vesicomyid clam symbionts. Open reading frames (ORFs) of *uvrC* are largely deleted in clade I symbionts. Lengths of the *uvrC* ORF and of the translated product are shown in the columns. The arrangement and direction of *uvrC* and neighboring genes of *Ca*. Ruthia magnifica are shown at the top: Rmag_0599, toluene tolerance protein; Rmag_0598, uvrC; Rmag_0597, hypothetical protein. Bidirectional arrows in columns indicate the consensus *uvrC* motifs. Other symbols and demarcations as in Fig 1.

While the ORFs for *uvrC* were found to be highly fragmented in clade I symbionts, in clade II symbionts their amino acid sequences were similar (amino acid identity = 91–96%) and had a constant length, 1785 bp (coding 594 aa) (Fig 2). Consensus amino acid sequences found in *E*. *coli* were also well-conserved in the intact *uvrC*s of clade II symbionts (Figs 2 and S4), suggesting that this gene is intact and functional in clade II symbionts. The deletion profiles of *uvrC* remnants varied among clade I symbionts, but were nearly identical for Psoy_S and Pkil_S, symbionts of the sibling vesicomyids *P*. *soyoae* and *P*. *kilmeri* (Figs 2 and S4).

**Fig 3:**
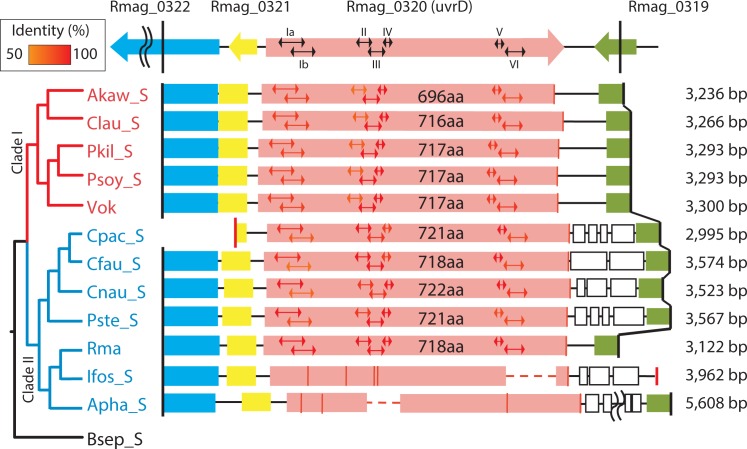
Deletion profiles of *uvrD* in the PCR products of vesicomyid clam symbionts. Vertical red lines on Cpac_S and Ifos_S indicate PCR primers redesigned for the second PCR. Gene arrangement and the direction of *uvrD* and neighboring genes of *Ca*. Ruthia magnifica are shown at the top, including Rmag_0319, hypothetical protein gene; Rmag_0320, *uvrD*; Rmag_0321, hypothetical protein gene; Rmag_3022, Lysine 2, 3-aminomutase YodO family protein gene. Bidirectional arrows in columns indicate the consensus helicase motifs. Symbols and demarcations as in Fig 1.

The ORFs for *uvrD* helicase were apparently well conserved in most of the symbionts belonging to clades I and II (amino acid identity = 76–100%) (Fig 3). Lengths of the ORFs were near to that in *E*. *coli* (720 aa; BAE77487). The consensus 7 helicase motifs of the UvrD were well conserved in the intact *uvrD*s found in most symbionts (Figs 3 and S5). In contrast, the ORF was collapsed and degraded in the Ifos_S and Apha_S amplicons (Figs 3 and S5). Their deletion patterns differed among symbiont taxa (Figs 3 and S5).

We found a paralog of *uvrD* in the genomes of Rma and Vok between Rma 0079 (COSY_0086, arg-tRNA ligase) and Rma 0081 (COSY_0087, pseudogene), which we named *uvrDp* (*uvrD* paralog). Amino acid sequence identities and similarities between *uvrD* and *uvrD*p were 20–25% and 35–38%, respectively. DNA fragments containing the ORF of *uvrDp* were amplified from all of the examined symbiont genomes (S6 Fig), and all had nearly identical lengths ranging from 3159 bp (coding 1052 aa) to 3174 bp (coding 1057 aa) (S6 Fig). Small, in-frame deletions (3–9 bps) were found in the sequences of some symbionts of clade I and II (S6 Fig). Although the 7 helicase motifs were not found in the uvrDps, a Pfam domain search showed a C terminal domain-like region of uvrD and a PDDEXK 1 domain (restriction endonuclease family) similar to the AAA_19 domain (ATPases associated with various cellular activities (S6 Fig).

### Expression of NER-related genes examined by RT-PCR

**Fig 4:**
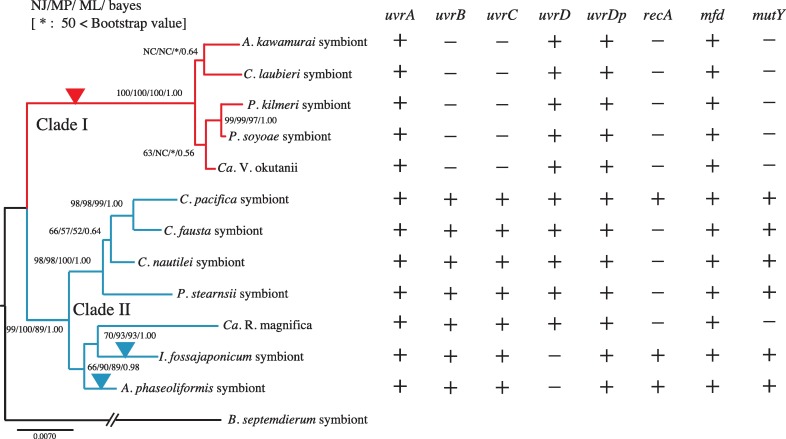
Presence or absence of NER-related genes, *mutY* and *recA* in genomes of vesicomyid clam symbionts. The presence (+) or absence (-) of intact genes are listed with a phylogenetic tree based on concatenated 16S-23S rRNA gene sequences. Possible positions of transition from an intact gene to a pseudogene for NER-related genes are indicated by vertical filled triangles. The red vertical triangle indicates the loss of *uvrB* and *uvrC* from the lineage of clade I symbionts. Vertical blue triangles indicate losses of *uvrD* from the lineage of two clade II symbionts.

**Fig 5:**
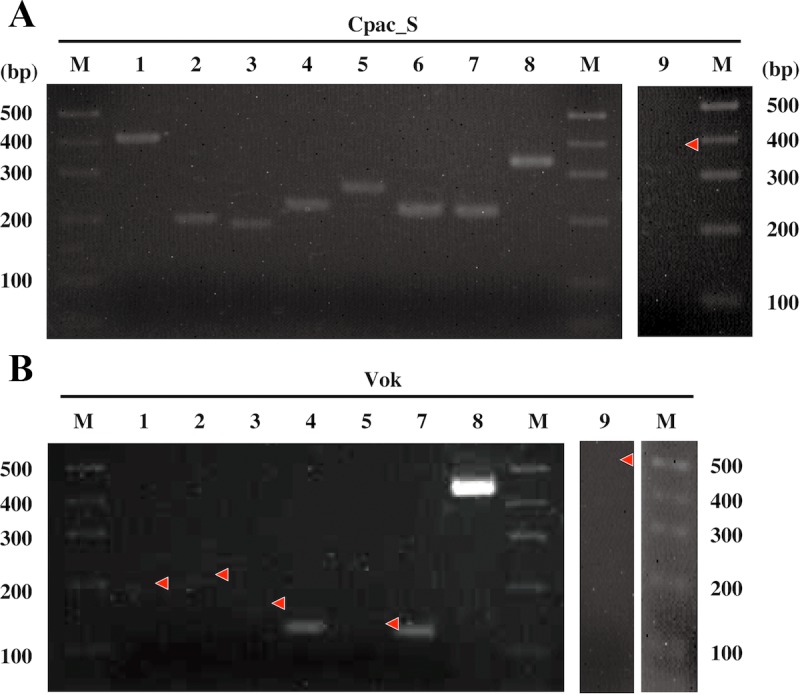
Electrophorograms of the RT-PCR products of NER genes in *Ca*. Vesicomyosocius okutanii (Vok) and the symbiont of *C*. *pacifica* (Cpac_S). A, Cpac_S; B, Vok. Expression of NER-related genes was analyzed by RT-PCR. Lane 1, *uvrA*; lane 2, *uvrB* or corresponding DNA region; lane 3, *uvrC* or corresponding DNA region; lane 4, *uvrD*; lane 5, *uvrDp*; lane 6, *recA*; lane 7, *mfd*; lane 8, 16S rRNA gene; lane 9, 16S rRNA gene negative control (with RNase treatment before RT PCR). M, molecular markers. Primers and the predicted lengths of amplicons are shown in S3 Table. Red arrowheads indicate the position of a band where no signal was detected.

A survey of NER genes with *recA* and *mutY* [3] revealed that clade I symbionts lacked *uvrB* and *uvrC*, and most clade II symbionts had a complete set of NER genes (Fig 4). NER, through either GP or TCP, does not proceed unless all of these genes are present, and it is interesting to examine whether the remaining NER-related genes in clade I symbionts are transcribed, even though they cannot contribute to NER. Among the apparently intact NER genes of Vok, RT-PCR indicated that *uvrD* and *mfd* were transcribed, but no transcript was detected for *uvrA* or *uvrDp* (Fig 5). Expressions of the remnants of fragmented *uvrB and uvrC* genes were not detected (Fig 5). In Cpac_S, all of the apparently intact NER-related genes (*uvrA*, *uvrB*, *uvrC*, *uvrD*, *uvrDp* and *mfd*) were transcribed (Fig 5). In addition, the expression of *recA* was also detected in Cpac_S (Fig 5).

### Genetic distances, GC contents and repeat sequence densities

**Fig 6:**
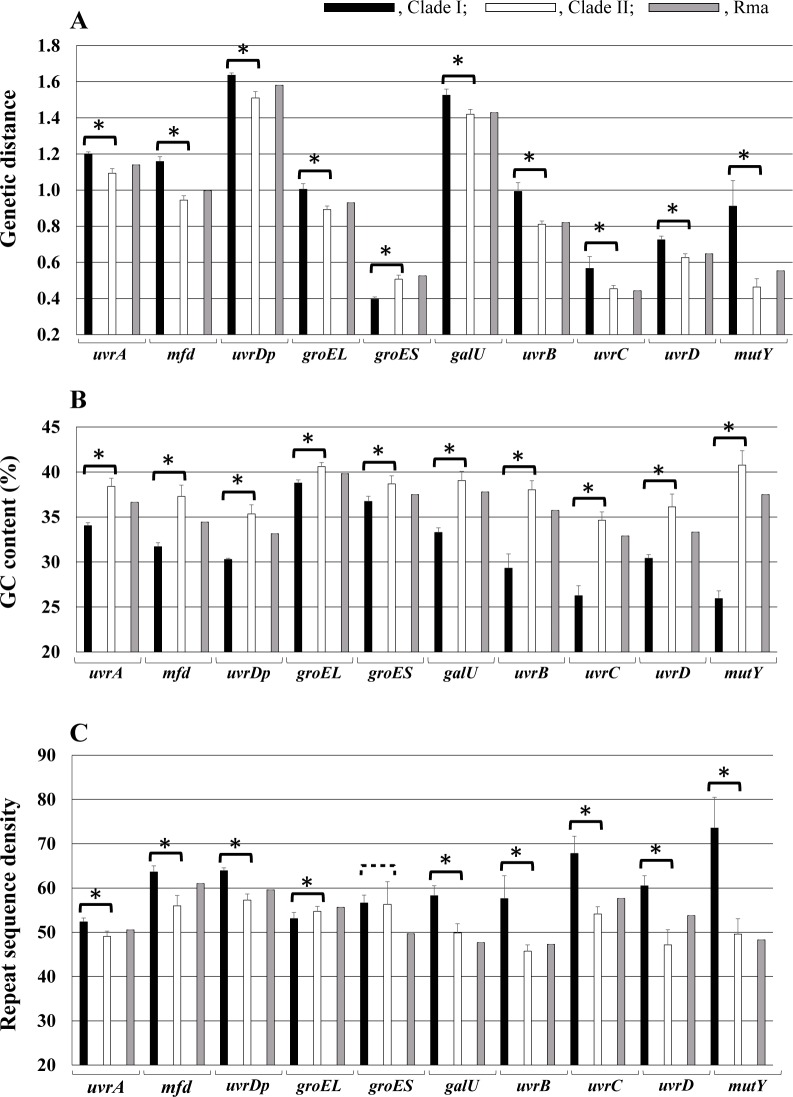
Comparison of genetic distances from the symbiont of *Bathymodiolus septemdierum*, GC contents and repeat sequence densities for ten genes among clade I, II symbionts and *Ca*. *Ruthia magnifica* (Rma). A, Averaged genetic distances from the *B*. *septemdierum* symbiont were calculated for genes in clade I symbionts (symbionts of *A*. *kawamurai*, *C*. *laubieri*, *P*. *kilmeri*, *P*. *soyoae* and *P*. *okutanii*) and clade II symbionts (symbionts of *C*. *pacifica*, *C*. *fausta*, *C*. *nautilei*, *P*. *steansii*, *C*. *magnifica*, *I*. *fossajaponicum* and *A*. *phaseoliformis*). B. Average GC contents (%) were calculated from the intact gene ORFs or their corresponding degraded regions in clades I and II. C. Moving average of densities of 5 bp repeat in a window of 200 bp DNA fragments was calculated with 10 bp shift of the window in the examined genes of clade I and II symbionts. Black filled columns indicate clade I symbionts; open columns represent all clade II symbionts; gray columns indicate Rma. Standard deviations indicated by crossed lines at top of bars. * on bracket indicates statistically significant difference (p<0.05).

To see the cumulative effect of the losses of *uvrA*, *uvrB*, *recA* and *mutY* from clade I symbionts, we measured genetic distance, GC content, and repeat sequence density (moving average of 5 bp repeat in 200 bp DNA sequence) for the genes *uvrA*, *uvrB*, *uvrC*, *uvrD*, *mfd*, *uvrDp*, *mutY*, *groEL*, *groES and galU* in clade I and clade II symbionts in relation to their respective genes in Bsep_S. (Fig 6). Because Rma lacks both *mutY* and *recA*, its values were also compared with clade I and II symbionts (Fig 6).

Clade I symbionts lack these 4 genes, and had significantly greater genetic distances for the examined genes than clade II, except those for *groESs*, which were significantly smaller in clade I (Fig 6A). The distances for Rma were mostly intermediate between those of clade I and clade II symbionts.

GC contents of all examined genes and the corresponding remnants of degraded genes in the clade I symbionts were significantly smaller than in clade II symbionts (Fig 6B). The differences between clade I and II symbionts were, however, smaller for the chaperonin genes (*groEL* and *groES*) than others (Fig 6B). Like genetic distances, GC contents in genes of Rma were intermediate between those of clade I and II symbionts (Fig 6B).

To see the effect of their losses on the RGE, we studied repeated sequence densities of the NER-related and other genes in clade I and clade II symbionts (Fig 6C). The densities of repeat sequences for nearly all examined genes (all except chaperonin genes *groEL* and *groES*) were significantly higher in clade I symbionts than for clade II symbionts (Fig 6C).

Although *uvrB*, *uvrC*, *recA and mutY* were commonly lost from clade I symbionts, the losses of *uvrD*, *recA* and *mutY* varied among clade II symbionts (Fig 4)[3]. The effects of losing DNA repair/recombination genes in various combinations were analyzed by comparing the genetic parameters of the 10 examined genes and their remnants between each symbiont pair using a round-robin paired t-test of (Tables 1–3). Differences were significant for each parameter in any pairs between clade I and clade II symbionts (Lower left halves of Tables 1, 2 and 3).

**Table 1:** Probability matrix of round-robin paired t-test of genetic distances of all examined genes (lower left) and of 6 intact genes (upper right) in 12 vesicomyid clam symbionts.

	Average-a*	Akaw_S^#^	Clau_S^#^	Pkil_S^#^	Psoy_S^#^	Vok^#^	Cpac_S^#^	Cfau_S^#^	Cnau_S^#^	Pste_S^#^	Rma^#^	Ifos_S^#^	Apha_S^#^
Average-b**		1.165	1.155	1.146	1.154	1.147	1.069	1.055	1.060	1.051	1.100	1.050	1.044
Akaw_S^#^	1.024		5.1.E-01	**9.0.E-03**	1.8.E-01	2.5.E-01	1.1.E-01	6.0.E-02	7.8.E-02	5.4.E-02	1.8.E-01	6.9.E-02	**3.4.E-02**
Clau_S^#^	1.037	5.1.E-01		6.1.E-01	9.4.E-01	7.4.E-01	1.7.E-01	1.0.E-01	1.3.E-01	9.2.E-02	2.9.E-01	1.1.E-01	6.3.E-02
Pkil_S^#^	0.993	3.4.E-01	1.9.E-01		1.9.E-01	9.0.E-01	1.7.E-01	9.5.E-02	1.2.E-01	8.4.E-02	3.0.E-01	9.8.E-02	5.2.E-02
Psoy_S^#^	0.997	4.2.E-01	2.5.E-01	2.2.E-01		6.6.E-01	1.0.E-01	5.4.E-02	7.1.E-02	**4.7.E-02**	1.9.E-01	6.1.E-02	**2.7.E-02**
Vok^#^	1.007	4.9.E-01	2.0.E-01	3.3.E-01	5.5.E-01		1.8.E-01	1.1.E-01	1.3.E-01	9.7.E-02	2.9.E-01	9.5.E-02	6.3.E-02
Cpac_S^#^	0.878	**3.2.E-02**	**2.4.E-02**	**1.3.E-02**	**1.3.E-02**	**1.7.E-02**		1.8.E-01	5.5.E-02	3.6.E-02	7.5.E-02	2.3.E-01	4.5.E-02
Cfau_S^#^	0.867	**2.5.E-02**	**1.7.E-02**	**1.0.E-02**	**6.1.E-03**	**1.2.E-02**	2.5.E-01		6.5.E-01	1.4.E-01	**2.9.E-02**	8.0.E-01	2.2.E-01
Cnau_S^#^	0.870	**1.2.E-02**	**1.7.E-02**	**9.4.E-03**	**8.9.E-03**	**1.1.E-02**	1.9.E-01	6.7.E-01		2.5.E-01	**1.6.E-02**	4.6.E-01	1.5.E-01
Pste_S^#^	0.860	**1.9.E-02**	**1.3.E-02**	**6.1.E-03**	**3.3.E-03**	**8.4.E-03**	**9.5.E-03**	1.4.E-01	**4.4.E-02**		**1.8.E-02**	9.8.E-01	3.8.E-01
Rma^#^	0.907	**4.3.E-02**	**3.4.E-02**	**2.6.E-02**	**1.4.E-02**	**2.6.E-02**	**3.3.E-02**	**2.3.E-02**	**1.2.E-02**	**8.8.E-03**		**3.2.E-03**	**4.6.E-03**
Ifos_S^#^	0.862	**2.8.E-02**	**2.1.E-02**	**1.0.E-02**	**1.2.E-02**	**1.3.E-02**	1.5.E-01	7.4.E-01	5.1.E-01	8.4.E-01	**1.4.E-02**		6.9.E-01
Apha_S^#^	0.862	**1.4.E-02**	**1.0.E-02**	**3.2.E-03**	**1.6.E-03**	**5.2.E-03**	6.4.E-02	6.8.E-01	4.0.E-01	6.3.E-01	**2.6.E-03**	9.4.E-01	

Lower-left half (gray-background), all of the 10 examined genes (*uvrA*, *uvrB*, *uvrC*, *uvrD*, *uvrDp*, *mfd*, *groEL*, *groES*, *galU* and *mutY*).

Upper-right half, 6 intact genes (*uvrA*, *uvrDp*, *mfd*, *groEL*, *groES* and *galU*).

*, average of the genetic distances of 10 genes in 12 symbionts.

**, average of the genetic distances of 6 intact genes in 12 symbionts.

Bold and underlined probabilities indicate statistically significant difference (P≤0.05).

#, Abbreviations of symbionts. See Fig 1.

**Table 2:** Probability matrix of round-robin paired t-test of GC content of all of the examined genes (lower left) and of 6 intact genes (upper right) in 12 vesicomyid clam symbionts.

	Average-a*	Akaw_S^#^	Clau_S^#^	Pkil_S^#^	Psoy_S^#^	Vok^#^	Cpac_S^#^	Cfau_S^#^	Cnau_S^#^	Pste_S^#^	Rma^#^	Ifos_S^#^	Apha_S^#^
Average-b**		34.13	33.87	34.25	34.25	34.17	38.33	38.33	38.23	38.53	36.55	38.80	38.87
Akaw_S^#^	31.64		3.9.E-01	7.5.E-01	7.6.E-01	9.0.E-01	**1.8.E-03**	**1.2.E-03**	**2.2.E-03**	**1.6.E-03**	**2.1.E-03**	**3.3.E-03**	**5.9.E-04**
Clau_S^#^	31.20	1.8.E-01		1.8.E-01	1.9.E-01	2.3.E-01	**1.6.E-03**	**1.4.E-03**	**2.2.E-03**	**1.8.E-03**	**3.6.E-03**	**5.1.E-03**	**5.8.E-04**
Pkil_S^#^	32.06	2.2.E-01	**1.6.E-02**		3.6.E-01	4.6.E-01	**2.6.E-03**	**3.1.E-03**	**4.2.E-03**	**4.1.E-03**	**1.7.E-02**	**9.7.E-03**	**1.5.E-03**
Psoy_S^#^	32.08	2.1.E-01	**1.5.E-02**	2.6.E-01		4.9.E-01	**2.6.E-03**	**3.1.E-03**	**4.2.E-03**	**4.1.E-03**	**1.7.E-02**	**9.7.E-03**	**1.5.E-03**
Vok^#^	31.38	5.0.E-01	4.2.E-01	8.0.E-02	8.0.E-02		**2.2.E-03**	**2.3.E-03**	**3.4.E-03**	**3.1.E-03**	**1.1.E-02**	**7.7.E-03**	**1.2.E-03**
Cpac_S^#^	38.05	**1.1.E-03**	**5.0.E-04**	**6.8.E-04**	**6.3.E-04**	**1.0.E-03**		9.9.E-01	4.7.E-01	4.9.E-01	**1.4.E-02**	4.6.E-01	8.8.E-02
Cfau_S^#^	37.91	**6.5.E-04**	**3.1.E-04**	**4.4.E-04**	**4.0.E-04**	**7.0.E-04**	4.2.E-01		4.0.E-01	1.8.E-01	**3.3.E-03**	3.5.E-01	**1.3.E-02**
Cnau_S^#^	37.96	**7.9.E-04**	**4.0.E-04**	**5.3.E-04**	**4.9.E-04**	**8.4.E-04**	9.7.E-01	6.4.E-01		1.1.E-01	**1.1.E-02**	2.9.E-01	**1.8.E-02**
Pste_S^#^	38.22	**7.0.E-04**	**3.5.E-04**	**4.9.E-04**	**4.5.E-04**	**7.4.E-04**	3.4.E-01	**1.2.E-02**	5.2.E-02		**3.0.E-03**	5.1.E-01	1.5.E-01
Rma^#^	35.88	**3.9.E-03**	**1.6.E-03**	**3.1.E-03**	**2.8.E-03**	**3.9.E-03**	**2.4.E-04**	**2.4.E-05**	**1.3.E-04**	**2.2.E-05**		**8.5.E-03**	**5.4.E-04**
Ifos_S^#^	38.72	**8.0.E-04**	**4.4.E-04**	**6.8.E-04**	**6.4.E-04**	**8.1.E-04**	1.2.E-01	**4.7.E-02**	5.4.E-02	1.3.E-01	**1.6.E-04**		9.0.E-01
Apha_S^#^	38.53	**3.0.E-04**	**1.4.E-04**	**1.8.E-04**	**1.6.E-04**	**3.2.E-04**	7.7.E-02	**1.4.E-03**	**2.5.E-03**	1.1.E-01	**2.4.E-06**	6.1.E-01	

Lower-left half (gray-background), all of the 10 examined genes (*uvrA*, *uvrB*, *uvrC*, *uvrD*, *uvrDp*, *mfd*, *groEL*, *groES*, *galU*, *mutY*).

Upper-right half, 6 intact genes (*uvrA*, *uvrDp*, *mfd*, *groEL*, *groES*, *galU*).

*, average of GC contents of all of 10 genes in 12 symbionts.

**, average of GC contents of 6 intact genes in 12 symbionts.

Bold and underlined probabilities indicate statistically significant difference (P≤0.05).

#, Abbreviations of symbionts. See Fig 1.

**Table 3:** Probability matrix of round-robin paired t-test of repeat sequence density (number of 5 bp repeat in 200 bp DNA) of all examined genes (lower left) and of 6 intact genes (upper right) in 12 vesicomyid clam symbionts.

	Average-a*	Akaw_S^#^	Clau_S^#^	Pkil_S^#^	Psoy_S^#^	Vok^#^	Cpac_S^#^	Cfau_S^#^	Cnau_S^#^	Pste_S^#^	Rma^#^	Ifos_S^#^	Apha_S^#^
Average-b**		57.78	59.12	57.49	57.50	57.98	54.27	53.56	53.00	54.22	54.02	53.15	54.88
Akaw_S^#^	60.83		9.5.E-02	8.0.E-01	8.0.E-01	2.2.E-01	1.3.E-01	9.4.E-02	8.0.E-02	5.8.E-02	9.0.E-02	8.3.E-02	3.1.E-01
Clau_S^#^	63.60	**1.6.E-02**		6.8.E-02	6.6.E-02	2.9.E-01	**3.7.E-02**	**2.2.E-02**	**9.2.E-03**	**1.2.E-02**	**2.3.E-02**	**1.5.E-02**	1.4.E-01
Pkil_S^#^	58.83	2.1.E-01	**1.8.E-02**		8.5.E-01	4.4.E-01	1.9.E-01	1.4.E-01	**4.8.E-02**	1.0.E-01	1.3.E-01	9.9.E-02	4.2.E-01
Psoy_S^#^	58.86	2.0.E-01	**1.7.E-02**	7.4.E-01		4.8.E-01	1.9.E-01	1.4.E-01	**4.8.E-02**	1.0.E-01	1.3.E-01	9.7.E-02	4.1.E-01
Vok^#^	61.53	3.8.E-01	6.3.E-02	7.0.E-02	6.9.E-02		1.2.E-01	1.0.E-01	**4.4.E-02**	9.0.E-02	1.5.E-01	8.3.E-02	6.2.E-01
Cpac_S^#^	52.22	**2.1.E-02**	**6.2.E-03**	**9.9.E-03**	**1.1.E-02**	**1.1.E-02**		5.4.E-01	6.4.E-01	4.8.E-01	9.1.E-01	5.9.E-01	5.4.E-01
Cfau_S^#^	51.56	**1.3.E-02**	**3.7.E-03**	**5.7.E-03**	**6.2.E-03**	**6.8.E-03**	3.5.E-01		6.5.E-01	7.3.E-01	8.4.E-01	9.5.E-01	4.0.E-01
Cnau_S^#^	52.24	**3.5.E-03**	**1.5.E-03**	**1.0.E-03**	**1.0.E-03**	**2.2.E-03**	9.9.E-01	5.4.E-01		1.9.E-01	7.3.E-01	8.2.E-01	5.6.E-01
Pste_S^#^	52.02	**1.3.E-02**	**3.7.E-03**	**5.0.E-03**	**5.4.E-03**	**6.3.E-03**	8.1.E-01	4.6.E-01	8.1.E-01		9.2.E-01	7.2.E-01	3.6.E-01
Rma^#^	53.11	**2.8.E-02**	**6.8.E-03**	**1.0.E-02**	**1.1.E-02**	**1.6.E-02**	5.9.E-01	2.8.E-01	5.3.E-01	4.9.E-01		6.1.E-01	7.8.E-01
Ifos_S^#^	50.46	**1.1.E-02**	**3.9.E-03**	**5.1.E-03**	**5.4.E-03**	**8.0.E-03**	7.4.E-02	2.8.E-01	2.0.E-01	4.9.E-01	7.8.E-02		3.6.E-01
Apha_S^#^	52.28	**2.9.E-02**	**1.0.E-02**	**2.9.E-02**	**3.0.E-02**	**1.9.E-02**	9.2.E-01	2.2.E-01	9.8.E-01	8.1.E-01	6.9.E-01	1.1.E-01	

Lower-left half (gray-background), all of the 10 examined genes (*uvrA*, *uvrB*, *uvrC*, *uvrD*, *uvrDp*, *mfd*, *groEL*, *groES*, *galU*, *mutY*).

Upper-right half, 6 intact genes (*uvrA*, *uvrDp*, *mfd*, *groEL*, *groES*, *galU*).

*, average of repeat sequence densities of 10 genes in 12 symbionts.

**, average of repeat sequence densities of 6 intact genes in 12 symbionts.

Bold and underlined probabilities indicate statistically significant difference (P≤0.05).

#, Abbreviations of symbionts. See Fig 1.

In contrast with the loss of *uvrB* and *uvrC* in clade I symbionts, *uvrD* was lost in 2 clade II symbionts of *A*. *phaseoliformis* and *I*. *fossajaponicum* (Fig 4). To see the effect of losing *uvrD*, we compared genetic parameters (genetic distance, GC content, repeat sequence density) for these two symbionts with those of Cpac_S, which had all the examined or reported DNA repair genes, including *mutY* and *recA*. Cpac_S was not significantly different from Apha_S or Ifos_S for any of the examined parameters (Tables 1, 2 and 3).

The effect of the loss of *recA* was examined by comparing Cpac_S with three clade II symbionts (Cfau_S, Cnau_S and Pste_S), each of which lacks *recA* but has *mutY* and other examined NER genes (Fig 4). The genetic distances of each gene examined differed significantly only between Cpac_S and Pste_S (Lower left half of Table 1). GC content and the repeat sequence density did not differ between these pairs (Lower left halves of Tables 2 and 3).

Rma is the only clade II symbiont lacking both *mutY* and *recA* (Fig 4). The effects of the apparently recent loss of *mutY* on genetic distance, GC content, and repeat sequence density were examined by comparing Rma with three clade II symbionts, Cfau_S, Cnau_S and Pste_S, which lack *recA*, using a round-robin t-test. The genetic distance for Rma was significantly greater (Lower left half of Table 1) than any of the clade II symbionts. GC content for Rma was smaller (Lower left half of Table 2) than not only the three symbionts but also any one of other clade II symbionts, though no significant difference was detected in the repeat sequence density (Lower left half of Table 3).

The round-robin paired t-test, parameters for exclusively intact genes (*uvrA*, *mfd*, *uvrDp*, *groEL*, *groES* and *galU*) in each symbiont pair indicated that Rma distance was significantly larger than those of other clade II symbionts except Cpac_S (upper right half of Table 1). GC content of the intact genes for all clade I symbionts was significantly lower than any clade II symbionts. The GC content of Rma differed from all examined symbionts, and was significantly larger than clade I but smaller than clade II symbionts (upper right half of Table 2). Comparison of the repeat sequence density of intact genes indicated that the density for Clau_S was significantly larger than 6 of the clade II symbionts (upper right half of Table 3). The repeat sequence density for Cnau_S was significantly smaller than all clade I symbionts except Akaw_S (upper right half of Table 3). No significant difference was detected within the clade I or II members (upper right half of Table 3).

When a protein coding gene becomes a pseudogene, selection pressure on the gene is generally reduced [20]. To see this change after the transition from a gene to a pseudogene or to a highly fragmented gene, we examined the ratios of clade I to clade II symbionts for the genetic distance, GC contents and repeat sequence densities of their intact genes or the corresponding remnant gene sequences (for a graphic presentation, see S7 Fig). The clade I / clade II ratios for the genetic distance of degraded genes (*uvrB*, *uvrC* and *mutY*; mean ± SD = 1.48±0.42) were significantly higher than for genes intact in both clades (*uvrA*, *mfd*, *groEL*, *groES*, *galU* and intact *uvrD*; mean ± SD = 1.08 ± 0.14; p = 0.030; Mann-Whitney U test). Thus, substitutional mutation rates were higher in degraded gene sequences than for intact genes. The ratios for GC contents were significantly lower for degraded clade I genes (mean ± SD = 0.72 ± 0.07) than for intact genes from both clades (mean ± SD = 0.89 ± 0.05; p = 0.017; Mann-Whitney U test). The ratio of repeat sequence density was significantly higher in the degraded genes from clade I (degraded genes, mean ± SD = 1.33 ± 0.13; intact genes, mean ± SD = 1.10 ± 0.10; p = 0.017; Mann-Whitney U test).

**Fig 7:**
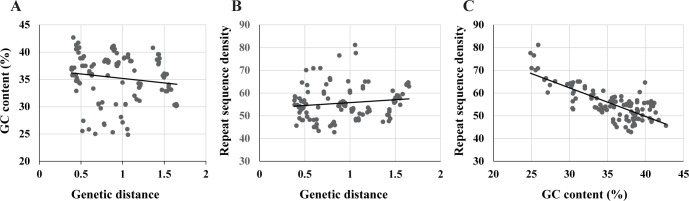
**Regressions of genetic distance versus (vs.) GC content (A), of genetic distance vs. repeat sequence density (B), and of repeat sequence density vs. GC content (C).** Each dot indicates genetic distance, GC content and/or the 5 bp repeat sequence density of each gene for each symbiont. Correlation coefficients (R) were 0.14 in genetic distance vs. GC content (A), 0.13 in genetic distance vs. repeat sequence density (B) and 0.76 in GC content vs. repeat sequence density (C). Coefficients of determination (R^2) were 0.020 in A, 0.018 in B and 0.58 in C. Regression was significant only for C (P = 2.9E-24).

Base substitution rates are expected to increase after the loss of DNA repair genes (including NER genes) [3, 6]. The higher rate of substitutional mutations is thought to be a driving force for the increment of the repeat sequence density, which is also thought to enhance the chance of illegitimate recombination and DNA deletions [6, 21]. To examine this possibility, we performed regression analyses among genetic distances, GC content, and the repeat sequence densities for all the examined genes (Fig 7A, 7B and 7C). Although GC content and repeat sequence density did not vary significantly with genetic distance (p>0.05; Fig 7A and 7B), repeat sequence density decreased significantly with increasing GC content (P = 2.9E-24, R = 0.765, R^2 = 0.585, Fig 7C).

## Discussion

### Losses of NER-related genes in the phylogeny of *Calyptogena* spp. symbionts

The phylogenetic topology of *Calyptogena* spp. symbionts suggested that genes *uvrB* and *uvrC* were lost from clade I symbionts after they diverged from clade II symbionts, but before their radiation within the clade (red arrowhead in Fig 4). In clade II symbionts, *uvrD*, on the other hand, seems to have been lost from these lineages after their diversification (Fig 4). From clade II lineages, *mutY* and *recA* have also been shown to be lost after their diversification [3].

Degradation of NER-related genes might be initiated by a base substitution and/or a small deletion, as previously estimated for the degradation of *recA* and *mutY* [3] in ancestral clade I symbionts. Deletion profiles for these clade I symbionts are considerably diversified, except among the symbionts of two sibling species, *Pliocardia soyoae* and *P*. *kilmeri* (Figs 1 and 2, S2 and S4 Figs). This suggests that after a pseudogenization event, small gene deletions have accumulated in their remnant gene regions in the descendant lineages, and that their DNA deletion profiles were diversified (Figs 1–3) as suggested previously for *mutY* and *recA* [3]. The deletion profile of a gene may be a useful proxy for understanding the phylogenetic relationships among very closely related species or taxa, though precise multi-gene alignment may be difficult. The almost identical deletion profiles of the respective genes in Psoy_S and Pkil_S (S2 and S4 Figs) agree well with the proposal that their hosts belong to the same transpacific species [22, 23].

### Raison d’être of NER-related genes after the loss of some of their members

In Vok (clade I), expressions of *uvrA* and *uvrDp* were not detected (Fig 5). This may indicate that *uvrA* does not function in Vok unless *uvrB* and *uvrC* are also intact; therefore, its expression is suppressed. It remains unclear whether *uvrA*, *uvrD* and *mfd* are poised for deterioration. If degradation to pseudogene status is imminent, the dN/dS (ratio of non-synonymous/synonymous base substitution rates) values of *uvrA*, *uvrD* and *mfd* would be expected to be higher than for functioning genes and near an equilibrium value of 1.0. However, these values (*uvrA* [clade I, 0.164±0.001; clade II, 0.156±0.005], *uvrD* [clade I, 0.260±0.004; clade II, 0.258±0.017], and *mfd* [clade I, 0.254±0.005; clade II, 0.234±0.007]) were in the range of other presumably functional genes (*groEL* [clade I, 0.178±0.008; clade II, 0.181±0.009], *groES* [clade I, 0.350±0.007; clade II, 0.283±0.015] and *galU* [clade I, 0.189±0.085; clade II, 0.207±0.116]; For a graphic presentation, see S8 Fig). This suggests that these genes may still be functional in either clade, and under selective stress. The dN/dS values of these genes and other examined genes were relatively high (0.1–0.5) suggesting they are under the relaxed purifying selection [24].

In Cpac_S, all examined NER genes were intact (Fig 4), and mRNAs of all of the examined genes were detected (Fig 5). This suggests that the NER pathways are functional in this symbiont.

It is not clear whether UvrDp complements the function of UvrD in these symbionts. Helicase is important not only for DNA repair but also for DNA replication, and is essential for growth. *UvrD*, a helicase, was found to be lost in the clade II symbionts Apha_S and Ifos_S (Figs 3 and 4). Although the *uvrDp* was similar (35–38% amino acid identity) to *uvrD*, it was shown to lack the 7 conserved helicase domains of UvrD (S6 Fig). However, expression of this gene was not detected in Vok (Fig 5B). The high dN/dS of *uvrDp* (clade I, 0.442±0.010; clade II, 0.395±0.006; S8 Fig) may indicate that selection pressure on *uvrDp* is relaxed, and that it does not play an important role (e.g., repair and replication of DNA).

### Effects of the loss of DNA repair genes on genome reductive evolution

Previously, we proposed that the loss of DNA repair genes may increase mutation rates [6], and reported that the loss of *mutY* resulted in increasing GC content [3]. The branch length of the phylogenetic tree based on 16S rRNA gene before the divergence of clade I symbionts is longer than the corresponding branch of the clade II symbionts (Fig 4) [3]. The differences of three examined parameters, genetic distance, GC content and repeat sequence density, between clade I symbionts and clade II symbionts (Fig 6A–6C) seemed to be the result of the loss of *uvrB*, *uvrC* and *mutY*. The differences in genetic distance and GC content between Rma and other clade II symbionts, including three *recA* lacking symbionts, are likely to be caused by the loss of *mutY* in Rma (Tables 1 and 2). Although the *mutY* was probably inactivated more recently in Rma than in clade I symbionts [3], the effect of its loss likely had a significant effect on both the mutation rate and GC content.

It is not clear whether the loss of *uvrD* affects genetic distance, GC content or repeat sequence density (Tables 1, 2 and 3). Thus, the loss of NER genes may be less important than the loss of *mutY* for affecting the genome through mutation rates. However, because it seems to have been lost after the diversification of clade II symbionts, this apparently slight effect may be explained by the shorter evolutionary period after the loss. Comparison of Cpac_S with Cfau_S, Cnau_S and Pste_S by the round-robin paired t-test suggested that the loss of *recA* did not have a significant effect within clade II symbionts. Therefore, it remains unclear how the loss of *recA* affected the parameters in these vesicomyid clam symbionts. RecA functions together with other *recA* related gene products [25], which have been shown to be lost from Vok and Rma [6]. The effect of the loss of *recA* must be re-evaluated by analyzing these related genes and larger (>200 bp) repeat sequences in the future.

The profiles of parameters studied for chaperonin genes *groEL* and *groES* (Fig 6A and 6C) differed from other genes examined. The GroEL-ES complex folds newly synthesized proteins or refolds partially unfolded proteins [26], and has been proposed to buffer genes against deleterious mutations [27]. GroEL is known to play a versatile function in intracellular symbionts and is highly expressed [28]. GroEL was also highly expressed in Vok (Yoshida, T., unpublished data). In insect endosymbionts, GroEL is under positive selection [29], and highly expressed genes, including GroEL and GroES, have a tendency to have high GC-biased amino acids [30]. This lead to the idea that *groEL-ES* may be under stronger selection pressure in genomes with higher mutation rates. However, the dN/dS values for *groEL* (clade I, 0.178±0.008; clade II, 0.181±0.009) and *groES* (clade I, 0.350±0.007; clade II, 0.283±0.015) were not lower than other examined genes (S8 Fig). This does not support the above idea, and it is interesting to consider whether chaperonin has a unique function in symbionts of deep-sea bivalves.

It is noteworthy that in Cpac_S, whose examined DNA repair genes were all apparently intact, genetic distance was not the smallest, GC content was not the highest, and its repeat sequence density was not the smallest among all examined symbionts (Fig 4, Tables 1–3). Factors other than the examined gene losses may have affected these parameters.

In addition to the effect of losing NER-related genes on mutation rates, the loss of *uvrA* and *uvrB* is thought to directly increase illegitimate recombination [31]. Their loss may affect the RGE directly in clade I symbionts.

Our results indicate that even though the direct influence of mutation rate on repeat sequence density is low, it has a significant influence on repeat sequence density via GC content (Fig 7A–7C). If the base substitution rate increases without changing the GC content, the repeat density would reach a dynamic equilibrium where its generation and degeneration are balanced. Mutations that either increase or decrease GC content would drive a shift of the repeat sequence density toward the new balanced state. This explains how base substitutional mutations regulate the repeat sequence density [6], and also indicates the importance of the loss of *mutY* for the RGE of the vesicomyid symbionts, which are thought to be in an intermediate stage of the RGE.

### Reductive genome evolution in *vesicomyid* clam symbionts.

Two major pathways are known in prokaryotic RGE, adaptive streamlining RGE and a ratchet driven path for random genetic drift [24, 32, 33]. Intracellular symbionts tend to reduce the genome size [34]. Loss of DNA repair genes and/or recombination genes, combined with an increase in the rate of base substitution mutation has been reported both in the streamline pathway [35] and in the random genetic drift pathway [36]. For symbiont genomes lacking *recA*, deletion is thought to proceed by RecA-independent illegitimate recombination [21, 37].

A smaller symbiont genome economizes energy and materials for unnecessary syntheses of genes and gene products, and hence, energy consuming biological materials [38]. Bacterial genome size is balanced by gene addition (gene acquisition and gene duplication) and gene loss (pseudogenization and gene erosion by deletion) [34]. When an intracellular symbiosis is established, gene acquisition is thought to be reduced, probably by the barrier of the host cell, and the balance moves towards reduction of genome size [34].

In deep-sea vesicomyid clams, the symbiont is protected from the outside environment in a symbiosome, a membrane-enclosed vesicle in gill epithelial cells, though they may be attacked by the host defense system and/or digestion [39]. If the genome of a symbiont is small, but contains enough essential genes for its reproduction, survival, and maintenance, it can survive within the host organism. Symbionts of extant vesicomyid clams have a medium-sized (approximately 1.0–1.2 Mb) genome, much larger than the very small genomes reported in insect symbionts [40], and is thought to be in an active stage of RGE [6]. While symbionts with smaller genomes may have a narrower survivable range of environmental variation, intracellular conditions in symbionts (i.e., inside host cells) are relatively stable, and if the growth rate of the symbiont is sufficient, symbiosis can be sustainable within the host cell without competitors, predators, and virulent viruses. Although it is still not clear whether a smaller genome is beneficial for the symbiont and the symbiotic system, the present study indicates that the loss of DNA repair genes would facilitate RGE in the symbionts.

## Conclusions

In the NER-related genes *uvrA*, *B*, *C*, *D*, *uvrDp* and *mfd* of vesicomyid clam symbionts, *uvrB* and *C* were shown to be lost from clade I symbiont lineages before their differentiation, and *uvrD* to be lost from two clade II symbionts after their diversification. The loss of NER-related genes and *mutY* seem to have had significant effects on their genetic parameters including; increased genetic distance, reduced GC content, and increased repeated sequence density. Interactions among these parameters indicate that GC content is the most influential in changing the repeat sequence density of genes, which is thought to be important for *recA*-independent DNA deletion [3]. Loss of *mutY* was also suggested to have an important role, leading to a decrease in the GC content of the genome and an increase in the rate of base substitutional mutations, which in turn may facilitate the RGE by increasing short repeat sequences in the genome.

## Materials and methods

### Sample collection and extraction of DNA and RNA

Nine vesicomyid clams (*A*. *kawamurai*, *C*. *laubieri*, *P*. *okutanii*, *P*. *soyoae*, *C*. *fausta*, *C*. *nautilei*, *I*. *fossajaponicum* and *A*. *phaseoliformis*) were collected from various deep sea habitats in Japan and stored at -80°C in a freezer (S1 Table). *C*. *pacifica*, *P*. *stearnsii* and *P*. *kilmeri* were collected in Monterey Bay in the United States of America, transported to Japan and stored in a -80°C freezer (S1 Table). No permit was required for their collection and research activities in the area in Japan. Specimens from the Monterey Bay area that were used in this study were collected under permits from the Monterey Bay National Marine Sanctuary and the California Dept. of Fish and Wildlife.

DNA was extracted from gill tissue (ca. 10 mg) from each vesicomyid clam species using a DNeasy tissue Kit (QIAGEN) as described previously [3].

Total RNA was extracted from approximately 10 mg of frozen gill tissue from each vesicomyid species with an RNeasy mini Kit (QIAGEN) according to the manufacturer’s instructions. After extracting total RNA, contaminated DNA was digested using a TURBO DNA-free Kit (Life Technologies).

### Amplification and sequencing of NER genes

DNA fragments containing the NER genes were amplified by PCR using LA Taq (TaKaRa) according to the manufacturer’s instruction. Primers for PCR amplification in this study were designed from their conserved sequence regions in Vok and Rma (S2 Table). When amplicons were not obtained from any symbionts, the primer set was re-designed by using the obtained amplicon sequences (S2 Table). The specific RT-PCR primer sets for NER genes were designed from aligned sequences of Vok and the *C*. *pacifica* symbiont (Cpac_S). Specificities of the designed primers were validated with PCR using DNA extracted from the gill tissues of *P*. *okutanii* and *C*. *pacifica*.

For the PCR, 25 μl of the reaction mixture contained 80 ng of template DNA, 2.5 μl of 10× LA Taq buffer (TaKaRa), 2.5 μl of dNTP mix (TaKaRa), 5 μl each of the 1 pmol/μl forward and reverse primer solutions, 0.13 μl of LA Taq polymerase solution (TaKaRa) and 8.87 μl of pure water was used. The gene fragments were amplified with an ABI 9700 Thermal cycler using a protocol; 96°C for 2 min and 30 cycles of 96°C for 20 s, 56°C for 15 s and 72°C for 2 or 5 or 7 min, followed by extension at 72°C for 10 min. After amplification, 2 μl of the reaction product mixture was applied to 1% agarose electrophoresis gel and stained with an ethidium bromide solution to visualize the reaction products.

After ethanol precipitation, the nucleotide sequences of the amplified DNA were determined using a Big Dye Terminator v3.1 Cycle Sequencing Kit (Applied Biosystems) with an ABI PRIZM 3130xl Genetic Analyzer and ABI 3730xl Genetic Analyzer (Applied Biosystems) according to the manufacturer’s instruction. The obtained sequences were assembled and analyzed with Sequencher ver 4.10.1 (Gene Codes). The sequences were submitted to DDBJ-EMBL-GENBANK, and their accession numbers are listed in S1 Table. Sequences of Rma were retrieved from DDBJ/GENBANK/EMBL database.

### RT-PCR

Using the total RNAs from Vok and Cpac_S as templates, their cDNAs were prepared by a PrimeScript RT-PCR Kit (TaKaRa) with random primers according to the manufacturer’s instruction. Each of the NER genes was amplified from the cDNAs using the specific primer sets (S3 Table). Composition of the reaction mixture was the same as that of the amplification from genomic DNAs.

We used an amplification protocol of 98°C for 2 min and 35 cycles of 98°C for 10 s, 55°C for 30 s and 72°C for 1 min, followed by extension at 72°C for 10 min with an ABI 9700 Thermal cycler. Following the amplification, the reaction products were electrophoresed in 3% agarose gel. Finally, the sequence of each amplification product was confirmed by DNA sequencing. For a negative control, RT-PCR of 16S rRNA was performed after RNase treatment.

### Genetic parameters; genetic distance, GC content and repeat sequence density

Genetic distances of 10 genes (*uvrA*, *uvrB*, *uvrC*, *uvrD*, *mfd*, *uvrDp*, *mutY*, *groEL*, *groES* and *galU*) from the symbiont of *Bathymodiolus septemdierum* (Bsep_S) were estimated by using CodeML in the PAML 4.5 package [41], and maximum-likelihood phylogenetic trees were constructed with the RAxML v7.2.6 [42] and MEGA6 [43] after aligning the sequences. The sequences of intact genes were aligned by using the MAFFT v7.164b[44]. The fragmented genes were manually aligned. Their GC contents were determined using GENETYX-MAC software, version 14 (Genetyx, Tokyo, Japan). We estimated the density of repeated sequences using a PERL programming script. Because the *groES* gene was the shortest (230 bp) gene examined, we searched for 5-base identical tandem repeats within windows of 200 bp DNA fragment, using a 10 base shift throughout each of the examined genes. Before selecting this condition, we examined 3 to 12 base tandem repeat (complete match) densities in the *groES* gene of Vok. Tandem repeat sequence densities (average ± standard deviation, SD) of 3, 4, 5, 6, 7, 8, 9, 10, 11 and 12 base repeats were 188 ± 1.83, 125.11 ± 8.37, 56.78 ± 11.38, 25.44 ± 8.18, 14.67 ± 5.96, 5.56 ± 3.98, 2.67 ± 2.98, 1.78 ± 1.99, 0.89 ± 0.99 and 0 ± 0, respectively. Each value indicates the number of single repeat sequences, but not that of the pairs, because three identical repeat sequences sometimes appeared in 200 bp fragments. A five base tandem repeat was thought to be appropriate (not too many and not too small) for comparing the repeat densities in various genes. A moving average of the raw repeat sequence density data was calculated by the script and was used as to indicate the repeat sequence density.

### Analyses of the effects of gene losses on the genomic genes

Two methods were used to analyze the effects of lost DNA repair/recombination genes on genetic parameters (genetic distance, GC content, and repeat sequence density) for 10 genes (*uvrA*, *uvrB*, *uvrC*, *uvrD*, *mfd*, *uvrDp*, *mutY*, *groEL*, *groES* and *galU*) among the 12 symbionts. First, we assessed the cumulative effect of lost genes, including losses of NER-related genes and the previously reported loss of *mutY* and/or *recA* on genomic genes [3]. For this analysis we compared these genetic parameters for clade I and clade II symbionts across 10 genes. The results were analyzed using a t-test or ANOVA. Second, we examined more detailed effects of the loss of DNA repair/recombination genes by comparing genetic parameters of 10 genes for each symbiont with those of other symbionts using a round-robin paired t-test among symbiont pairs. In addition, to exclude the effect of pseudogenization or deletion of some genes, we analyzed the genetic parameters of exclusively intact genes between every symbiont pair. The results were evaluated using round-robin paired t-tests.

### Statistical analyses

An R-platform was used to perform Student’s t-tests (including Welch test) to evaluate differences in the genetic distance, GC content, and repeat sequence density of genes between symbionts of clade I and II or between symbionts with or without certain DNA repair genes. Student’s t-tests, Paired t-tests, ANOVA, Mann-Whitney’s *U* tests and regression analyses were performed using Excel by using Statcel3 (Statcel-The Useful Add-in Forms on Excel-3rd ed., by Hisae Yanai, published by OMS Publishing Inc., Saitama, Japan) [45]. A probability threshold of 0.05 was used to determine statistical significance.

## Supporting information

S1 Table Accession numbers of DNA sequences obtained in this study or retrieved from databases. (PDF)

S2 Table List of primers for PCR of NER-related genes. (PDF)

S3 Table List of primers for RT-PCR of NER-related genes. (PDF)

S1 Fig Multiple alignment of *uvrA*s and the coding of amino acid sequences in symbionts of vesicomyid clams. For each symbiont listed, nucleotide sequences and amino acid sequences are shown on the upper and lower portions of each sub-table, respectively. * indicates stop codon. Conserved domains of *uvrA* found in an NCBI blast search are shown as blue bidirectional arrows below the alignment. More specific conserved sequences, such as ATP binding sites, are shown in bold/underline within the alignment. The consensus sequences of *uvrA* in *Escherichia coli* are also shown below the alignment [1].
(PDF)

S2 Fig Multiple alignment of DNA sequences and the coding of amino acid sequences for *uvrB* in symbionts of vesicomyid clams. The arrangement of nucleotide and amino acid sequences is as described in S1 Fig. Although the ORFs of this gene were shown to be collapsed in clade I symbionts and do not code proteins, residual amino acid coding of nucleotide sequences were estimated and used for manual alignment. # indicates the gap of amino acid sequence where no corresponding nucleotide sequence exist. * indicates stop codon. Conserved domains of *uvrB* found in NCBI blast search are shown as bidirectional arrows. Specific conserved sequences, 7 consensus helicase motifs of *E*. *coli*, are shown below the alignment [2, 3]. Among them, motifs I, Ia, V and VI were well conserved, while motifs II, III and IV were moderately conserved. The conserved motifs suggested that the remaining *uvrB*s in clade II symbionts were functional.
(PDF)

S3 Fig Multiple alignment of *mfd* nucleotide sequences and their translated amino acid sequences in symbionts of vesicomyid clams. The arrangement of nucleotide and amino acid sequences is as described in S1 Fig. Conserved domains of *mfd* found in an NCBI blast search are shown as bidirectional arrows. Seven helicase motifs of Mfd from *Escherichia coli* are shown below the alignment, and the corresponding sequences of the symbionts are underlined [4]. # indicates the gap of amino acid sequence where no corresponding nucleotide sequence exist. * indicates stop codon.
(PDF)

S4 Fig Multiple alignment of DNA sequences and translated amino acid sequences of *uvrC* in symbionts of vesicomyid clams. The arrangement of nucleotide and amino acid sequences is as described in S1 Fig. Although the ORFs of this gene were collapsed in clade I symbionts and do not code proteins, the remnant amino acid sequences for coding nucleotide sequences were estimated as was possible and used for the alignment. Conserved domains of *uvrC* found in an NCBI blast search are shown as bidirectional arrows. Specific conserved sequences found in *uvrC* from *E*. *coli* are shown below the alignment [5, 6]. Bold-face, underlined letters indicate the conserved amino acid residues. The conserved hydrophobic amino acid residues in the HhH (helix-hairpin-helix) domain are shown with a red background. The conserved glycine residues in the HhH domain have a yellow background. # indicates the gap of amino acid sequence where no corresponding nucleotide sequence exist. * indicates stop codon.
(PDF)

S5 Fig Multiple alignment of DNA sequences and translated amino acid sequences of *uvrD* in symbionts of vesicomyid clams. ORFs of *uvrD* of all clade I and II symbionts except *Abyssogena phaseoliformis* and *Isorropodon fossajaponica* were intact. Although the ORFs of this gene in symbionts of *A*. *phaseoliformis* and *I*. *fossajaponica* were collapsed and do not code proteins, remnant amino acid coding nucleotide sequences were estimated as was possible. Conserved domains of *uvrD* found in an NCBI blast search are shown as bidirectional arrows. Conserved helicase sequences (motifs Ia-VI) of *uvrD* from *Escherichia coli* are shown below the alignment [7]. Variation in their length seems to have resulted from small deletions. # indicates the gap of amino acid sequence where no corresponding nucleotide sequence exist. * indicates stop codon.
(PDF)

S6 Fig Multiple alignment of *uvrDp* (*uvrD* paralog) nucleotide sequences and their translated amino acid sequences in symbionts of vesicomyid clams. The arrangement of nucleotide and amino acid sequences is as described in S1 Fig. Conserved domains of *uvrD* found in an NCBI blast search are shown as bidirectional arrows. # indicates the gap of amino acid sequence where no corresponding nucleotide sequence exist. * indicates stop codon.
(PDF)

S7 Fig **Comparison of genetic distances (A), GC (guanine+cytosine) contents (B) and repeat sequence densities (C) for genes that are intact in both clade symbionts (I/I-genes) and genes which are degraded in clade I but intact in clade II symbionts (D/I-genes).** I/I-genes include *uvrA*, *uvrD* (excluding those of two clade II symbionts, *I*. *fossajaponica* and *A*. *phaseoliformis*, of which *uvrD* was shown to be degraded), *mfd*, *uvrDp*, *groEL*, *groES* and *galU*. D/I-genes include *uvrB*, *uvrC* and *mutY*. Differences between I/I-genes and D/I-genes were examined by comparing three parameters (genetic distance from *Bathymodiolus septemdierum*, GC content and repeat sequence density). We first calculated an average of each parameter for clade I and II symbionts. These averages for clade I symbionts were divided by those for clade II symbionts (clade I/clade II ratios). These ratios were significantly higher in the D/I-genes than I/I genes for genetic distance (Mann-Whitney’s U test, P = 0.030) and repeat sequence density (Mann-Whitney U test, P = 0.017) but were lower in D/I-genes than I/I genes for GC content (Mann-Whitney U test, P = 0.017). * on a bracket indicates statistical significance.
(EPS)

S8 Fig Ratios of non-synonymous substation rates to synonymous substitution rates (dN/dS) for the examined genes in clade I and clade II symbionts. Filled columns indicate clade I symbionts. Open columns indicate clade II symbionts. Bars above columns indicate the standard deviation. Solid bracket with * indicates a statistically significant (p< 0.05) difference between clade I and clade II symbionts. Dashed bracket indicates n.s. difference. n.d. indicates the datum was not determined because of the loss of the open reading frames.
(EPS)
