# Correction: Loss of genes related to Nucleotide Excision Repair (NER) and implications for reductive genome evolution in symbionts of deep-sea vesicomyid clams

**DOI:** 10.1371/journal.pone.0176805

**Published:** 2017-04-25

**Authors:** Shigeru Shimamura, Takashi Kaneko, Genki Ozawa, Mamiko Nishino Matsumoto, Takeru Koshiishi, Yoshihiro Takaki, Chiaki Kato, Ken Takai, Takao Yoshida, Katsunori Fujikura, James P. Barry, Tadashi Maruyama

## Notice of Republication

This article was republished on March 30, 2017, as an incorrect version of Fig 4 was published in error. Please download this article again to view the correct version.
