# Supplementary material for: Loss of genes related to Nucleotide Excision Repair (NER) and implications for reductive genome evolution in symbionts of deep-sea vesicomyid clams
Source: PLoS One. 2017 Feb 15;12(2):e0171274. doi: 10.1371/journal.pone.0171274 (PMC5310779; doi:10.1371/journal.pone.0171274)
Supplement: S1 Table — (PDF) [file pone.0171274.s001.pdf]

S1 Table

Symbiont (clade)*	Site#	Depth (m)	Accession number							
			uvrA	*uvrB*	*uvrC*	*uvrD*	*uvrDp*	*mfd*	*groES /groEL*	*pgi / galU*
Akaw_S	ONT	608	AB911366	AB911375	AB911386	AB911395	AB911406	AB911356	LC090393	LC090384
Clau_S	ONT	3761	AB911367	AB911377	AB911387	AB911397	AB911407	AB911357	LC090395	LC090385
Pkil_S	MB	900	AB911365	AB911376	AB911385	AB911396	AB911405	AB911355	LC090394	LC090383
Psoy_S	NT	1171	AB911368	AB911378	AB911388	AB911398	AB911408	AB911358	LC090399	LC090388
Vok	SaB	852	AP009247**	AP009247**	AP009247**	AP009247**	AP009247**	AP009247**	AP009247**	AP009247**
Cpac_S	MB	659-683.5	AB911372	AB911382	AB911392	AB911402	AB911412	AB911362	LC090397	LC090386
Cfau_S	SuB	1490	AB911369	AB911379	AB911389	AB911399	AB911409	AB911359	LC090391	LC090382
Cnau_S	NT	3257	AB911371	AB911381	AB911391	AB911401	AB911411	AB911361	LC090396	LC090389
Pste_S	MB	659-683.5	AB911374	AB911384	AB911394	AB911404	AB911414	AB911364	LC090400	LC090390
Ifos_S	JT	6181	AB911370	AB911380	AB911390	AB911400	AB911410	AB911360	LC090392	Unpublished Data***
Apha_S	JT	5347	AB911373	AB911383	AB911393	AB911403	AB911413	AB911363	LC090398	LC090387
Rma	EPR	-	CP000488**	CP000488**	CP000488**	CP000488**	CP000488**	CP000488**	CP000488**	CP000488**
Bsep_S	MK	-	AP013042**	AP013042**	AP013042**	AP013042**	AP013042**	AP013042**	AP013042**	AP013042**

*: Abbreviated symbionts, see Fig. 1.

#, Collection site: ONT, Okinawa-Nankai Trough, JAPAN; MB. Monterey Bay, USA; NT, Nankai Trough, JAPAN; SaB, Sagami Bay, JAPAN; SuB, Suruga Bay, JAPAN; JT, Japan Trench; EPR, East Pacific Rise, USA; MK, Myojin Knoll, JAPAN.

* * Retrieved from the genome data.

* * * Yoshida et al. unpublished genome data.
