# Supplementary material for: Loss of genes related to Nucleotide Excision Repair (NER) and implications for reductive genome evolution in symbionts of deep-sea vesicomyid clams
Source: PLoS One. 2017 Feb 15;12(2):e0171274. doi: 10.1371/journal.pone.0171274 (PMC5310779; doi:10.1371/journal.pone.0171274)
Supplement: S2 Table — (PDF) [file pone.0171274.s002.pdf]

S2 Table

Genes	Primer sequence*	Target symbionts#
uvrA	F5'GCRCGTARTGCTGCRCCAGTGTC3'	ALL
	R5'RCTTGATGGAGGYCCWATTATTGCC3'	ALL
uvrB	F5'YCTATCGGAAAGATRTRCTGACATG3'	ALL
	R5'YACRCTWGGCAARCCAACTTCACG3'	Akaw_S, Clau_S, Pkil_S, Psoy_S, Vok, Cpac_S, Cnau_S, Pste_S, Ifos_S, Apha_S
	R5'TCCAGCGCAATTAATGCCAATAACG3'	Cfau_S
uvrC	F5'YATTAAGYGCRGCTGAATTAGGCGG3'	ALL
	R5'AAAAHHCGATTGTTAATCCACCAYAC3'	ALL
uvrD	F5'AWYCCAATTGCTRATRGCATC3'	Akaw_S, Clau_S, Psoy_S, Vok, Cfau_S, Cnau_S, Pste_S, Ifos_S, Apha_S
	F5'CCCAATTGCTAATAGCATCATGCTC3'	Pkil_S
	F5'CCTGCAACCCAGCCTTGAATATAG3'	Cpac_S
	R5'CCAGGATTRAARCAYCCRCTTAG3'	Akaw_S, Clau_S, Pkil_S, Psoy_S, Vok, Cpac_S, Cfau_S, Cnau_S, Pste_S, Apha_S
	R5'TGTGTCCAAGTAGGTTAGAGCATTG3'	Ifos_S
uvrDp	F5'TATGTGAATGATGCRGGTCGGCAG3'	Akaw_S, Pkil_S, Cpac_S, Cfau_S, Cnau_S, Pste_S, Ifos_S
	F5'AARGYTTCCATGTCAACCCGTAGTG3'	Clau_S, Psoy_S, Vok, Cpha S
	R5'AAGCATTTGATGCTGGMTGTGATGC3'	ALL
mfd	F5'RCGTYAATRCTACYAACAACCTCAC3'	Clau_S, Pkil_S, Cpac_S, Cfau_S, Cnau_S, Pste_S, Apha_S
	F5'TGACCTACCAATAGAGTCATGAGTC3'	Psoy_S, Vok
	F5'TCTCACCTCATCCTGATATTACCTC3'	Akaw_S
	F5'ACCAACAACCTCACAACCATCAGC3'	Ifos_S
	R5'AGGTTGTGGWGAYGCTTATCGTGC3'	Clau_S, Cpac_S, Cfau_S, Cnau_S, Pste_S, Apha_S
	R5'GACAATTGGCAATGCGTTTATAAAGC3'	Akaw_S, Pkil_S
	R5'AGATCCAACAGGTTGTGGAGATGC3'	Psoy_S, Vok
	R5'CGATATTATCACCATCGCTACTAGC3'	Ifos_S
groEL-groES	F5'YGCACCAGAACAATAYTTAACCAATG3'	ALL
	R5'WAYCCTGCAATTGYYTCATCAACAC3'	Clau_S, Psoy_S, Vok, Cpac_S, Cfau_S, Cnau_S, Pste_S, Ifos_S, Apha_S
	R5'ACTTCAATAGAGGAAGAGTCGTAGC3'	Akaw_S, Pkil_S,
galU	F5'GCTTGTGGTACTAGTTATAACGCAG3'	Akaw_S, Clau_S, Pkil_S, Psoy_S, Vok, Cpac_S, Cfau_S, Apha_S
	F5'GCTTGTGGYACTAGTTATAAYGCAG3'	Cnau_S, Pste_S
	R5'GATGGCTGGATGTGTCGATACCAT3'	Akaw_S, Clau_S, Pkil_S, Psoy_S, Vok, Cpac_S, Cfau_S, Apha_S
	R5'GTTAAGGCTGTATCCCTATATGCAC3'	Cnau_S, Pste_S

*, F5' sequences indicate forward primer sequences; R5' sequences indicate reverse sequences.
#, Abbreviations of symbionts. See Fig. 1.
