# Supplementary material for: Loss of genes related to Nucleotide Excision Repair (NER) and implications for reductive genome evolution in symbionts of deep-sea vesicomyid clams
Source: PLoS One. 2017 Feb 15;12(2):e0171274. doi: 10.1371/journal.pone.0171274 (PMC5310779; doi:10.1371/journal.pone.0171274)
Supplement: S3 Table — (PDF) [file pone.0171274.s003.pdf]

S3 Table

Genes	Primer sequence for Vok*	Primer sequence for Cpac_S*	Predicted length of amplified-fragment	
			Vok	Cpac_S
uvrA	**F5'AATCTCAAACCATTTCTCAAATGG3'**	**F5'AGAATGATGCGACGTTATGAAG3'**	188bp	403bp
	**R5'CTTCCATTTCATCTATATACACAAC3'**	**R5'TCATCAAGCACATACAGCACAC3'**		
uvrB	**F5'TATAGCCAAACCTATTATCAATA3'**	**F5'ATGAATCACATGTGACTGTTAGC3'**	200bp	198bp
	**R5'GTTTAATCTGATTACGCATATC3'**	**R5'GAAACTTCCAACTCATATTGTGC3'**		
uvrC	**F5'GCTTCTCAAAATTTAGAGTTTGA3'**	**F5'GCCTCTCAAAATTTAGAGTTTGA3'**	150bp	192bp
	**R5'CCTTAATACCACTTACTCC3'**	**R5'TTGTCCAATTTGCTTACCAGAG3'**		
uvrD	**F5'ATTAAATGGATTTACCAACC3'**	**F5'CAACTCGCGGTATTGGCAATG3'**	125bp	231bp
	**R5'GTTTTATCATTAGAATAATGC3'**	**R5'TGCGCCATTAATCCTGATGTG3'**		
uvrDp	**F5'TTTGATGTTAATCAAACGCATG3'**	**F5'TTGTATTGTCACAACAAGACGG3'**	129bp	271bp
	**R5'ACTCATCAATCAAAATATGCTG3'**	**R5'CTGTTGCGCAGACTCACAATC3'**		
mfd	**F5'ATCACGATCTTGAAATTCGTGGTGC3'**	**F5'GCTTGCCATCCATTATTCCAG3'**	123bp	221bp
	**R5'GAGTACACGGCATCAATGGTGCG3'**	**R5'AGAGGTTGATTTCATCAATGCC3'**		
recA	**none**	**F5'CTCAGTACGCTTAGATATTCGC3'**	-	222bp
	**none**	**R5'AACACTATACCAAGCACCTGC3'**		
**16S**	**F5'GGGGGTACCCTTTAGTGGCGAAGCT3'**	**F5'CCGTAAACGATGATAACTAGTCG3'**	440bp	341bp
	**R5'AGCACCACCTCACGGCTTAGCGAC3'**	**R5'TATAGTTCCCACCATGATGTGC3'**		

*, F5' sequences indicate forward primer sequences; R5' sequences indicate reverse sequences.

#, Abbreviations of symbionts. See Fig. 1.
