# Supplementary material for: Loss of genes related to Nucleotide Excision Repair (NER) and implications for reductive genome evolution in symbionts of deep-sea vesicomyid clams
Source: PLoS One. 2017 Feb 15;12(2):e0171274. doi: 10.1371/journal.pone.0171274 (PMC5310779; doi:10.1371/journal.pone.0171274)
Supplement: S1 Fig — For each symbiont listed, nucleotide sequences and amino acid sequences are shown on the upper and lower portions of each sub-table, respectively. * indicates stop codon. Conserved domains of uvrA found in an NCBI blast search are shown as blue bidirectional arrows below the alignment. More specific conserved sequences, such as ATP binding sites, are shown in bold/underline within the alignment. The consensus sequences of uvrA in Escherichia coli are also shown below the alignment [1]. (PDF) [file pone.0171274.s004.pdf]

S1 Fig.

```
Akaw_S   1 ATGGATCAAATTAGTATTCGTGGTGCTAGAGTTCACAATTTAAAAAATATTGACATTGAT   60
Clau_S   1 ATGGATCATATTAGTATTCGTGGTGCTAGAGTTCACAATTTAAAAAATATTGACATTGAT   60
Pkil_S   1 ATGGATCAAATTAGTATTCGTGGTGCTAGAGTTCACAATTTAAAAAATATCGATATTGAT   60
Psoy_S   1 ATGGATCAAATTAGTATTCGTGGTGCTAGAGTTCACAATTTAAAAAATATCGATATTGAT   60
Vok      1 ATGGATCAAATTAGTATTCGTGGTGCTAGAGTTCACAATTTAAAAAATATCGATATTGAT   60
Cpac_S   1 ATGGATCAAATTAGTATTCATGGCGCCAGAGCCCACAATTTAAAAAATATCGATATTGAC   60
Cfau_S   1 ATGGATCAAATTAGTATTCATGGCGCTAGAGTCCACAATTTAAAAAATATCGATATTGAC   60
Cnau_S   1 ATGGATCAAATTAGTATTCGTGGCGCCAGAGTCCACAATTTAAAAAATATCGATATTGAC   60
Pste_S   1 ATGGATCAAATTAGTATTCGTGGTGCCAGAGTCCACAATTTAAAAAATATCGATATTAAC   60
Rma      1 ATGGATCAAATTAGTATTCGTGGCGCTAGAGTCCACAATTTAAAAAATATCGATATTGAT   60
Ifos_S   1 ATGGATCAAATTAGTATTCGTGGTGCCAGAGTCCACAATTTAAAAAATATCGATATTGAT   60
Apha_S   1 ATGGATCAAATTAGTATTCGTGGTGCCAGAGTCCACAATTTAAAAAATATCGATATTGAC   60
Bsep_S   1 ATGGATCAAATTAGCATACGCGGCGCACGCGTTCATAATCTAAAAAATATCGATATTGAT   60
Akaw_S   1   M  D  Q  I  S  I  R  G  A  R  V  H  N  L  K  N  I  D  I  D   20
Clau_S   1   M  D  H  I  S  I  R  G  A  R  V  H  N  L  K  N  I  D  I  D   20
Pkil_S   1   M  D  Q  I  S  I  R  G  A  R  V  H  N  L  K  N  I  D  I  D   20
Psoy_S   1   M  D  Q  I  S  I  R  G  A  R  V  H  N  L  K  N  I  D  I  D   20
Vok      1   M  D  Q  I  S  I  R  G  A  R  V  H  N  L  K  N  I  D  I  D   20
Cpac_S   1   M  D  Q  I  S  I  H  G  A  R  A  H  N  L  K  N  I  D  I  D   20
Cfau_S   1   M  D  Q  I  S  I  H  G  A  R  V  H  N  L  K  N  I  D  I  D   20
Cnau_S   1   M  D  Q  I  S  I  R  G  A  R  V  H  N  L  K  N  I  D  I  D   20
Pste_S   1   M  D  Q  I  S  I  R  G  A  R  V  H  N  L  K  N  I  D  I  N   20
Rma      1   M  D  Q  I  S  I  R  G  A  R  V  H  N  L  K  N  I  D  I  D   20
Ifos_S   1   M  D  Q  I  S  I  R  G  A  R  V  H  N  L  K  N  I  D  I  D   20
Apha_S   1   M  D  Q  I  S  I  R  G  A  R  V  H  N  L  K  N  I  D  I  D   20
Bsep_S   1   M  D  Q  I  S  I  R  G  A  R  V  H  N  L  K  N  I  D  I  D   20
```

ABC_UvrA_I domain (4-117)

```
Akaw_S  61 ATTCCTAGAAACAAATTGATTGTAATTACTGGACTATCTGGATCAGGTAAGTCTTCACTA  120
Clau_S  61 ATTCCTAGAAACAAATTGATTGTAATTACTGGATTATCTGGATCAGGTAAATCATCCCTA  120
Pkil_S  61 ATTCCTAGAAATAAATTGATTGTAATTACTGGACTATCTGGATCAGGTAAATCTTCACTA  120
Psoy_S  61 ATTCCTAGAAATAAATTGATTGTAATTACTGGACTATCTGGATCAGGTAAATCTTCACTA  120
Vok     61 ATTCCTAGAAATAAATTGATTGTAATTACTGGACTATCTGGATCAGGTAAATCTTCATTA  120
Cpac_S  61 ATCCCTAGAAATAAGTTAGTTGTGCTTACTGGTTTGTCTGGTTCGGGTAAATCTTCATTG  120
Cfau_S  61 ATCCCTAGAAATAAGTTAGTTGTGCTTACTGGTTTGTCTGGTTCGGGTAAATCTTCACTG  120
Cnau_S  61 ATCCCCAGAAATAAGTTAGTTGTGCTTACTGGTTTGTCTGGTTCGGGTAAATCTTCATTG  120
Pste_S  61 ATCCCTAGAAATAAGTTAGTTGTGCTTACTGGTTTGTCTGGTTCGGGTAAATCTTCATTG  120
Rma     61 ATCCCTAGAAACAAGTTAGTTGTGATTACTGGGTTATCTGGTTCGGGTAAATCCTCACTG  120
Ifos_S  61 ATTCCTAGAAACAAGTTAGTTGTGATTACTGGCTTGTCTGGTTCGGGTAAATCTTCACTG  120
Apha_S  61 ATCCCTAGAAACAAGTTAGTTGTGATTACTGGCTTGTCTGGTTCAGGTAAATCTTCACTG  120
Bsep_S  61 ATTCCTAGAAATAAACTGGTTGTTATTACAGGTTTGTCCGGATCAGGCAAGTCCTCACTT  120
Akaw_S  21   I  P  R  N  K  L  I  V  I  T  G  L  S  G  S  G  K  S  S  L   40
Clau_S  21   I  P  R  N  K  L  I  V  I  T  G  L  S  G  S  G  K  S  S  L   40
Pkil_S  21   I  P  R  N  K  L  I  V  I  T  G  L  S  G  S  G  K  S  S  L   40
Psoy_S  21   I  P  R  N  K  L  I  V  I  T  G  L  S  G  S  G  K  S  S  L   40
Vok     21   I  P  R  N  K  L  I  V  I  T  G  L  S  G  S  G  K  S  S  L   40
Cpac_S  21   I  P  R  N  K  L  V  V  L  T  G  L  S  G  S  G  K  S  S  L   40
Cfau_S  21   I  P  R  N  K  L  V  V  L  T  G  L  S  G  S  G  K  S  S  L   40
Cnau_S  21   I  P  R  N  K  L  V  V  L  T  G  L  S  G  S  G  K  S  S  L   40
Pste_S  21   I  P  R  N  K  L  V  V  L  T  G  L  S  G  S  G  K  S  S  L   40
Rma     21   I  P  R  N  K  L  V  V  I  T  G  L  S  G  S  G  K  S  S  L   40
Ifos_S  21   I  P  R  N  K  L  V  V  I  T  G  L  S  G  S  G  K  S  S  L   40
Apha_S  21   I  P  R  N  K  L  V  V  I  T  G  L  S  G  S  G  K  S  S  L   40
Bsep_S  21   I  P  R  N  K  L  V  V  I  T  G  L  S  G  S  G  K  S  S  L   40

E. coli                                G  L  S  G  S  G  K
```

ATPase site I.

ABC_UvrA_I domain (4-117)

```
Akaw_S 121 GCTTTTGATACTATTTATGCAGAAGGACATCGTCGTTATGTAGAGTCTTTATCAGCTTAT  180
Clau_S 121 GCTTTTGATACTATTTATGCAGAAGGACATCGTCGTTATGTAGAGTCTTTATCAACTTAT  180
Pkil_S 121 GCTTTTGATACTATTTATGCAGAAGGACATCGTCGTTATGTAGAGTCTTTATCAGCTTAT  180
Psoy_S 121 GCTTTTGATACTATTTATGCAGAAGGACATCGTCGTTATGTAGAGTCTTTATCAGCTTAT  180
Vok    121 GCTTTTGATACTATTTATGCAGAAGGACATCGTCGTTATGTAGAGTCTTTATCAGCTTAT  180
Cpac_S 121 GCTTTTGACACTATTTATGCAGAAGGGCAGCGTCGTTATGTGGAGTCTTTATCAACTTAT  180
Cfau_S 121 GCTTTTGACACTATTTATGCAGAAGGGCAGCGTCGTTATGTGGAGTCTTTATCAACTTAT  180
Cnau_S 121 GCTTTTGATACTATTTATGCAGAAGGGCAGCGTCGTTATGTGGAGTCTTTATCAACTTAT  180
Pste_S 121 GCTTTTGACACTATTTATGCAGAAGGGCAGCGTCGTTATGTGGAGTCTTTATCAACTTAT  180
Rma    121 GCTTTTGACACCATTTATGCAGAAGGACAGCGTCGTTATGTGGAGTCTTTATCAGCTTAT  180
Ifos_S 121 GCTTTTGATACCATTTATGCAGAAGGGCAGCGTCGCTATGTAGAGTCTTTATCGGCTTAT  180
Apha_S 121 GCTTTTGATACCATTTATGCAGAAGGGCAACGTCGCTATGTAGAATCTTTATCGGCTTAT  180
Bsep_S 121 GCATTTGATACCATTTATGCCGAAGGACAGCGTCGTTATGTAGAATCATTATCTGCCTAT  180
Akaw_S  41   A  F  D  T  I  Y  A  E  G  H  R  R  Y  V  E  S  L  S  A  Y   60
Clau_S  41   A  F  D  T  I  Y  A  E  G  H  R  R  Y  V  E  S  L  S  T  Y   60
Pkil_S  41   A  F  D  T  I  Y  A  E  G  H  R  R  Y  V  E  S  L  S  A  Y   60
Psoy_S  41   A  F  D  T  I  Y  A  E  G  H  R  R  Y  V  E  S  L  S  A  Y   60
Vok     41   A  F  D  T  I  Y  A  E  G  H  R  R  Y  V  E  S  L  S  A  Y   60
Cpac_S  41   A  F  D  T  I  Y  A  E  G  Q  R  R  Y  V  E  S  L  S  T  Y   60
Cfau_S  41   A  F  D  T  I  Y  A  E  G  Q  R  R  Y  V  E  S  L  S  T  Y   60
Cnau_S  41   A  F  D  T  I  Y  A  E  G  Q  R  R  Y  V  E  S  L  S  T  Y   60
Pste_S  41   A  F  D  T  I  Y  A  E  G  Q  R  R  Y  V  E  S  L  S  T  Y   60
Rma     41   A  F  D  T  I  Y  A  E  G  Q  R  R  Y  V  E  S  L  S  A  Y   60
Ifos_S  41   A  F  D  T  I  Y  A  E  G  Q  R  R  Y  V  E  S  L  S  A  Y   60
Apha_S  41   A  F  D  T  I  Y  A  E  G  Q  R  R  Y  V  E  S  L  S  A  Y   60
Bsep_S  41   A  F  D  T  I  Y  A  E  G  Q  R  R  Y  V  E  S  L  S  A  Y   60
```

ABC_UvrA_I domain (4-117)

```
Akaw_S 181 GCACGTCAATTTTTATCACTCATGGAAAAACCTGATGTTGATCATATTGAAGGGTTGTCT  240
Clau_S 181 GCACGTCAATTTTTATCACTCATGGAAAAACCTGATGTTGATCATATTGAAGGGTTATCT  240
Pkil_S 181 GCACGTCAATTTTTATCACTCATGGAAAAACCTGATGTTGATTATATTGAAGGGTTATCT  240
Psoy_S 181 GCACGTCAATTTTTATCACTCATGGAAAAACCTGATGTTGATTATATTGAAGGGTTATCT  240
Vok    181 GCACGTCAATTTTTATCACTCATGGAAAAACCTGATGTTGATCATATTGAAGGGTTATCT  240
Cpac_S 181 GTGCGTCAATTTTTGTCACTCATGGAAAAACCTGATGTTGATCATATTGAAGGACTCTCT  240
Cfau_S 181 GTGCGTCAATTTTTGTCACTCATGGAAAAACCTGATGTTGATTATATTGAAGGACTCTCT  240
Cnau_S 181 GTGCGTCAATTTTTGTCACTCATGGAAAAACCTGATGTTGATCATATTGAAGGACTCTCT  240
Pste_S 181 GTGCGTCAATTTTTGTCACTCATGGAAAAACCTGATGTTGATCATATTGAAGGACTCTCT  240
Rma    181 GCGCGTCAATTTTTGTCACTCATGGAAAAACCTGATGTTGACCATATTGAAGGACTATCT  240
Ifos_S 181 GCACGTCAGTTTTTATCACTCATGGAAAAACCTGATGTTGACCATATTGAAGGACTCTCT  240
Apha_S 181 GCACGCCAATTTTTATCACTCATGGAAAAACCTGATGTTGATCATATTGAAGGATTGTCT  240
Bsep_S 181 GCCAGACAGTTTTTATCGTTAATGGAAAAACCAGATGTTGATCATATTGAAGGTTTATCC  240
Akaw_S  61   A  R  Q  F  L  S  L  M  E  K  P  D  V  D  H  I  E  G  L  S   80
Clau_S  61   A  R  Q  F  L  S  L  M  E  K  P  D  V  D  H  I  E  G  L  S   80
Pkil_S  61   A  R  Q  F  L  S  L  M  E  K  P  D  V  D  Y  I  E  G  L  S   80
Psoy_S  61   A  R  Q  F  L  S  L  M  E  K  P  D  V  D  Y  I  E  G  L  S   80
Vok     61   A  R  Q  F  L  S  L  M  E  K  P  D  V  D  H  I  E  G  L  S   80
Cpac_S  61   V  R  Q  F  L  S  L  M  E  K  P  D  V  D  H  I  E  G  L  S   80
Cfau_S  61   V  R  Q  F  L  S  L  M  E  K  P  D  V  D  Y  I  E  G  L  S   80
Cnau_S  61   V  R  Q  F  L  S  L  M  E  K  P  D  V  D  H  I  E  G  L  S   80
Pste_S  61   V  R  Q  F  L  S  L  M  E  K  P  D  V  D  H  I  E  G  L  S   80
Rma     61   A  R  Q  F  L  S  L  M  E  K  P  D  V  D  H  I  E  G  L  S   80
Ifos_S  61   A  R  Q  F  L  S  L  M  E  K  P  D  V  D  H  I  E  G  L  S   80
Apha_S  61   A  R  Q  F  L  S  L  M  E  K  P  D  V  D  H  I  E  G  L  S   80
Bsep_S  61   A  R  Q  F  L  S  L  M  E  K  P  D  V  D  H  I  E  G  L  S   80
```

ABC_UvrA_I domain (4-117)

```
Akaw_S 241 CCAGCTATTTCTATTGAGCAAAAATTCACTTTACATAATCCACGTTCAACAGTTGGTACA  300
Clau_S 241 CCAGCTATCTCTATTGAGCAAAAATCCACTTCACATAATCCACGTTCAACAGTTGGTACG  300
Pkil_S 241 CCAGCTATTTCTATTGAGCAAAAATCCACTTCACATAATCCACGCTCAACAGTTGGTACA  300
Psoy_S 241 CCAGCTATTTCTATTGAGCAAAAATCCACTTCACATAATCCACGCTCAACAGTTGGTACA  300
Vok    241 CCAGCTATCTCTATTGAGCAAAAATCCACTTCACATAATCCACGCTCAACAGTTGGTACA  300
Cpac_S 241 CCAGCCATTTCCATTGAACAAAAAGCCACCTCACACAACCCACGCTCAACGGTTGGCACA  300
Cfau_S 241 CCAGCCATCTCCATTGAACAAAAAGCCATCTCACACAACCCCCGCTCAACGGTTGGCACA  300
Cnau_S 241 CCAGCCATCTCCATTGAACAAAAAGCCACCTCACATAACCCACGTTCAACGGTTGGTACA  300
Pste_S 241 CCAGCCATCTCTATTGAACAAAAAGCCACCTCACACAACCCACGCTCAACGGTTGGTACA  300
Rma    241 CCAACCATTTCTATTGAACAAAAAGCCACTTCTCACAATCCACGCTCAACGGTTGGTACA  300
Ifos_S 241 CCAGCCATCTCTATTGAGCAAAAAGCCACCTCACACAACCCACGCTCAACGGTCGGCACA  300
Apha_S 241 CCAGCCATCTCTATTGAGCAAAAAGCCACCTCGCATAATCCGCGCTCAACGGTCGGTACG  300
Bsep_S 241 CCTGCCATTTCCATAGAGCAAAAAGCCACTTCTCACAATCCGCGCTCAACCGTAGGTACC  300
Akaw_S  81   P  A  I  S  I  E  Q  K  F  T  L  H  N  P  R  S  T  V  G  T  100
Clau_S  81   P  A  I  S  I  E  Q  K  S  T  S  H  N  P  R  S  T  V  G  T  100
Pkil_S  81   P  A  I  S  I  E  Q  K  S  T  S  H  N  P  R  S  T  V  G  T  100
Psoy_S  81   P  A  I  S  I  E  Q  K  S  T  S  H  N  P  R  S  T  V  G  T  100
Vok     81   P  A  I  S  I  E  Q  K  S  T  S  H  N  P  R  S  T  V  G  T  100
Cpac_S  81   P  A  I  S  I  E  Q  K  A  T  S  H  N  P  R  S  T  V  G  T  100
Cfau_S  81   P  A  I  S  I  E  Q  K  A  I  S  H  N  P  R  S  T  V  G  T  100
Cnau_S  81   P  A  I  S  I  E  Q  K  A  T  S  H  N  P  R  S  T  V  G  T  100
Pste_S  81   P  A  I  S  I  E  Q  K  A  T  S  H  N  P  R  S  T  V  G  T  100
Rma     81   P  T  I  S  I  E  Q  K  A  T  S  H  N  P  R  S  T  V  G  T  100
Ifos_S  81   P  A  I  S  I  E  Q  K  A  T  S  H  N  P  R  S  T  V  G  T  100
Apha_S  81   P  A  I  S  I  E  Q  K  A  T  S  H  N  P  R  S  T  V  G  T  100
Bsep_S  81   P  A  I  S  I  E  Q  K  A  T  S  H  N  P  R  S  T  V  G  T  100
```

ABC_UvrA_I domain (4-117)

```
Akaw_S 301 ATTACTGAAATTTATGATTATTTAAGATTATTGTTTGCACGTATTGGTATTCCTAATTGT  360
Clau_S 301 ATTACTGAAATTTATGATTATTTAAGATTATTGTTTGCACGTATTGGTATTCCTAATTGT  360
Pkil_S 301 ATTACTGAAATTTATGACTATTTAAGATTATTGTTTGCACGTATTGGTATTCCTAATTGC  360
Psoy_S 301 ATTACTGAAATTTATGACTATTTAAGATTATTGTTTGCACGTATTGGTATTCCTAATTGC  360
Vok    301 ATTACTGAAATTTATGACTATTTAAGATTATTGTTTGCACGTATTGGTATTCCTATTTGC  360
Cpac_S 301 ATTACAGAAGTTTATGATTATTTAAGATTGTTGTTTGCTCGCGTAGGCATTCCTAAGTGT  360
Cfau_S 301 ATTACAGAAATTTATGATTATTTAAGATTATTGTTTGCTCGCGCAGGCATTCCTAAGTGC  360
Cnau_S 301 ATTACAGAAATTTATGATTATTTAAGATTGTTGTTTGCTCGTGCAGGCATTCCTAAGTGT  360
Pste_S 301 ATTACAGAAATTTATGATTATTTAAGATTGTTATTTGCTCGCGCAGGCATTCCTAAGTGT  360
Rma    301 ATTACAGAAATTTATGATTATTTAAGGTTGTTGTTTGCTCGTGCAGGTATTCCCAAGTGT  360
Ifos_S 301 ATTACAGAAATTTATGATTATTTAAGGTTGTTGTTTGCTCGCGCAGGCATTCCTAAGTGT  360
Apha_S 301 ATTACAGAAATTTATGATTATTTAAGGTTGTTGTTTGCTCGTGCAGGCATTCCTAAGTGT  360
Bsep_S 301 ATCACAGAAATCTACGATTATTTGCGACTGCTCTTCGCTCGCGTTGGTTTGCCAAAGTGT  360
Akaw_S 101   I  T  E  I  Y  D  Y  L  R  L  L  F  A  R  I  G  I  P  N  C  120
Clau_S 101   I  T  E  I  Y  D  Y  L  R  L  L  F  A  R  I  G  I  P  N  C  120
Pkil_S 101   I  T  E  I  Y  D  Y  L  R  L  L  F  A  R  I  G  I  P  N  C  120
Psoy_S 101   I  T  E  I  Y  D  Y  L  R  L  L  F  A  R  I  G  I  P  N  C  120
Vok    101   I  T  E  I  Y  D  Y  L  R  L  L  F  A  R  I  G  I  P  I  C  120
Cpac_S 101   I  T  E  V  Y  D  Y  L  R  L  L  F  A  R  V  G  I  P  K  C  120
Cfau_S 101   I  T  E  I  Y  D  Y  L  R  L  L  F  A  R  A  G  I  P  K  C  120
Cnau_S 101   I  T  E  I  Y  D  Y  L  R  L  L  F  A  R  A  G  I  P  K  C  120
Pste_S 101   I  T  E  I  Y  D  Y  L  R  L  L  F  A  R  A  G  I  P  K  C  120
Rma    101   I  T  E  I  Y  D  Y  L  R  L  L  F  A  R  A  G  I  P  K  C  120
Ifos_S 101   I  T  E  I  Y  D  Y  L  R  L  L  F  A  R  A  G  I  P  K  C  120
Apha_S 101   I  T  E  I  Y  D  Y  L  R  L  L  F  A  R  A  G  I  P  K  C  120
Bsep_S 101   I  T  E  I  Y  D  Y  L  R  L  L  F  A  R  V  G  L  P  K  C  120
```

ABC_UvrA_I domain (4-117)

```
Akaw_S 361 CCAAAACACAAAATTAATCTTGAATCTCAAACCATTTCTCAAATGGTTGATAGTATTGTT  420
Clau_S 361 CCAAAACACAAAATTAATCTTGAATCTCAAACCATTTCTCAAATGGTTGATAGTATTGGT  420
Pkil_S 361 CCAAAACATAAAATTAATCTTGAATCTCAAACCATTTCTCAAATGGTTGATAGTATTGTT  420
Psoy_S 361 CCAAAACATAAAATTAATCTTGAATCTCAAACCATTTCTCAAATGGTTGATAGTATTGTT  420
Vok    361 CCAAAACACAAAATTAATCTTGAATCTCAAACCATTTCTCAAATGGTTAATAATATTGTT  420
Cpac_S 361 CCAAAACACCAAATTAATCTTGAGTCTCAAACCATTTCTCAAATGGTAGATAGTATTGTC  420
Cfau_S 361 CCAAAACATCAAATTAATCTTGAGTCTCAAACCATTTCTCAAATGGTGGATAGTATTGTC  420
Cnau_S 361 CCAAAACACCAAATTAATCTTGAGTTTCAAACCATTTCTCAAATGGTGGATAGTATTGTC  420
Pste_S 361 CCAAAACACCAAATTAATCTTGAGTCTCAAACCATTTCTCAAATGGTGGATAGTATTGTC  420
Rma    361 CCAAAACACCAAATTAATCTTGAATCTCAAACCATTTCTCAAATGGTGGATAGTATTGTC  420
Ifos_S 361 CCAAAGCACCAAATTAATCTTGAATCTCAAACCATTTCTCAAATGGTGGACGGCATTGTC  420
Apha_S 361 CCAGAACATCAAATTAATCTTGAATCTCAAACCATTTCTCAAATGGTGGATAGTATTGTC  420
Bsep_S 361 CCAAAGCATGGCATTGATTTAGTCTCACAAACTATTTCGCAAATGGTGGACAGTATTATG  420
Akaw_S 121   P  K  H  K  I  N  L  E  S  Q  T  I  S  Q  M  V  D  S  I  V  140
Clau_S 121   P  K  H  K  I  N  L  E  S  Q  T  I  S  Q  M  V  D  S  I  G  140
Pkil_S 121   P  K  H  K  I  N  L  E  S  Q  T  I  S  Q  M  V  D  S  I  V  140
Psoy_S 121   P  K  H  K  I  N  L  E  S  Q  T  I  S  Q  M  V  D  S  I  V  140
Vok    121   P  K  H  K  I  N  L  E  S  Q  T  I  S  Q  M  V  N  N  I  V  140
Cpac_S 121   P  K  H  Q  I  N  L  E  S  Q  T  I  S  Q  M  V  D  S  I  V  140
Cfau_S 121   P  K  H  Q  I  N  L  E  S  Q  T  I  S  Q  M  V  D  S  I  V  140
Cnau_S 121   P  K  H  Q  I  N  L  E  F  Q  T  I  S  Q  M  V  D  S  I  V  140
Pste_S 121   P  K  H  Q  I  N  L  E  S  Q  T  I  S  Q  M  V  D  S  I  V  140
Rma    121   P  K  H  Q  I  N  L  E  S  Q  T  I  S  Q  M  V  D  S  I  V  140
Ifos_S 121   P  K  H  Q  I  N  L  E  S  Q  T  I  S  Q  M  V  D  G  I  V  140
Apha_S 121   P  E  H  Q  I  N  L  E  S  Q  T  I  S  Q  M  V  D  S  I  V  140
Bsep_S 121   P  K  H  G  I  D  L  V  S  Q  T  I  S  Q  M  V  D  S  I  M  140
```

```
Akaw_S 421 AAATTGCCAATGGGTAGAAAAATTATGTTGTTAGCACCTATTGTTCAGAACCGTAAAGGT  480
Clau_S 421 AAATTACCAATGGGTAGAAAAATTATGTTGTTAGCACCTATTGTTCAGAACCGCAAAGGT  480
Pkil_S 421 AGATTACCAATGGGTAGAAAAATTATGTTGTTAGCACCTATTGTTCAGAACCGCAAAGGT  480
Psoy_S 421 AGATTACCAATGGGTAGAAAAATTATGTTGTTAGCACCTATTGTTCAGAACCGCAAAGGT  480
Vok    421 AAATTACCAATTGGTAGAAAAATTATGTTGTTAGCACCTATTGTTCAGAACCGTAAAGGT  480
Cpac_S 421 AAATTACCAACAGGTGAGAAGATCATGCTGCTAGCACCCATTGTGCAAAATCGCAAAGGC  480
Cfau_S 421 AAATTACCAACAGGTGAGAAAATCATGCTGCTAGCACCCATTGTGCAAAATCGCAAAGGC  480
Cnau_S 421 AAATTACCAACAGGTGAGAAAATCATGCTGCTAGCACCCATTGTGCAAAATCGCAAAGGC  480
Pste_S 421 AAATTACCAACAGGTGAGAAAATCATGCTGCTAGCACCCATTGTACAAAATCGCAAAGGC  480
Rma    421 AAGTTACCAATGGGTGAGAAAATCATGTTGTTAGCACCCATTGTACAAAATCGAAAAGGT  480
Ifos_S 421 AAATTACCAACGGGTGAGAAAATCATGTTGCTAGCACCTATTGTGCAAAATCGCAAAGGC  480
Apha_S 421 AAATTACCAATGGGTGAAAAAATCATGTTGCTAGCACCCATTGTGCAAAATCGCAAAGGT  480
Bsep_S 421 GCATTGTCAGAAGGTGAAAAAATTATGATATTAGCCCCTGTGGTGCAAAACCGCAAGGGC  480
Akaw_S 141   K  L  P  M  G  R  K  I  M  L  L  A  P  I  V  Q  N  R  K  G  160
Clau_S 141   K  L  P  M  G  R  K  I  M  L  L  A  P  I  V  Q  N  R  K  G  160
Pkil_S 141   R  L  P  M  G  R  K  I  M  L  L  A  P  I  V  Q  N  R  K  G  160
Psoy_S 141   R  L  P  M  G  R  K  I  M  L  L  A  P  I  V  Q  N  R  K  G  160
Vok    141   K  L  P  I  G  R  K  I  M  L  L  A  P  I  V  Q  N  R  K  G  160
Cpac_S 141   K  L  P  T  G  E  K  I  M  L  L  A  P  I  V  Q  N  R  K  G  160
Cfau_S 141   K  L  P  T  G  E  K  I  M  L  L  A  P  I  V  Q  N  R  K  G  160
Cnau_S 141   K  L  P  T  G  E  K  I  M  L  L  A  P  I  V  Q  N  R  K  G  160
Pste_S 141   K  L  P  T  G  E  K  I  M  L  L  A  P  I  V  Q  N  R  K  G  160
Rma    141   K  L  P  M  G  E  K  I  M  L  L  A  P  I  V  Q  N  R  K  G  160
Ifos_S 141   K  L  P  T  G  E  K  I  M  L  L  A  P  I  V  Q  N  R  K  G  160
Apha_S 141   K  L  P  M  G  E  K  I  M  L  L  A  P  I  V  Q  N  R  K  G  160
Bsep_S 141   A  L  S  E  G  E  K  I  M  I  L  A  P  V  V  Q  N  R  K  G  160
```

```
Akaw_S 481 AGTCATGTGCAGCTATTAAAAGAGTTAAATCACCAAGGTTTTTAAGGGCTAGGATTGAT  540
Clau_S 481 AGCCATGTACAATTATTAAAAGAGTTAAATCAACAAGGTTTTTAAGGGCTAGAATTGAT  540
Pkil_S 481 AGCCATGTACAATTATTAAAAGAGTTAAATCAGCAAGGTTTTTAAGGGCTAGAATTGAT  540
Psoy_S 481 AGCCATGTACAATTATTAAAAGAGTTAAATCAGCAAGGTTTTTAAGGGCTAGAATTGAT  540
Vok    481 AGCCATGTACAATTATTAAAAGAGTTAAATCAGCAAGGTTTTTAAGGGCTAGAATTGAT  540
Cpac_S 481 AGCCATGCAAAATTATTGGAAGAATTAAACCACCAAGGTTTTTAAGGGCTAGAGTTAAT  540
Cfau_S 481 AGCCATGCAAAATTATTGGAAGAATTAAACTACCAAGGTTTTTAAGGGCTAGAGTTAAT  540
Cnau_S 481 AGCCATGCAAAATTATTGGAAGAATTAAACCACCAAGGTTTTTAAGGGCTAGAGTTAAT  540
Pste_S 481 AGCCATGCAAAATTATTGGAAGAATTAAACCACCAAGGTTTTTAAGGGCTAGAGTTAAT  540
Rma    481 AGCCATGCAAAATTATTGGAAGAATTAAATCATCAAGGTTTTTAAGGGCTAGAGTTGAT  540
Ifos_S 481 AGCCATATAAAACTATTAGAAGAATTAAATCACCAAGGTTTTTAAGAGCTAGAGTTGAT  540
Apha_S 481 AGCCATACAAAACTATTGGAAGAATTAAATCACCAAGGTTTTTAAGGGCTAGAGTTGAT  540
Bsep_S 481 AGTCATGTCAAAATGTTAGAAGAACTTTCTCACCAGGGTTTTTGCGTGCCAGAGTCGAT  540
Akaw_S 161   S  H  V  Q  L  L  K  E  L  N  H  Q  G  F  L  R  A  R  I  D  180
Clau_S 161   S  H  V  Q  L  L  K  E  L  N  Q  Q  G  F  L  R  A  R  I  D  180
Pkil_S 161   S  H  V  Q  L  L  K  E  L  N  Q  Q  G  F  L  R  A  R  I  D  180
Psoy_S 161   S  H  V  Q  L  L  K  E  L  N  Q  Q  G  F  L  R  A  R  I  D  180
Vok    161   S  H  V  Q  L  L  K  E  L  N  Q  Q  G  F  L  R  A  R  I  D  180
Cpac_S 161   S  H  A  K  L  L  E  E  L  N  H  Q  G  F  L  R  A  R  V  N  180
Cfau_S 161   S  H  A  K  L  L  E  E  L  N  Y  Q  G  F  L  R  A  R  V  N  180
Cnau_S 161   S  H  A  K  L  L  E  E  L  N  H  Q  G  F  L  R  A  R  V  N  180
Pste_S 161   S  H  A  K  L  L  E  E  L  N  H  Q  G  F  L  R  A  R  V  N  180
Rma    161   S  H  A  K  L  L  E  E  L  N  H  Q  G  F  L  R  A  R  V  D  180
Ifos_S 161   S  H  I  K  L  L  E  E  L  N  H  Q  G  F  L  R  A  R  V  D  180
Apha_S 161   S  H  T  K  L  L  E  E  L  N  H  Q  G  F  L  R  A  R  V  D  180
Bsep_S 161   S  H  V  K  M  L  E  E  L  S  H  Q  G  F  L  R  A  R  V  D  180
```

```
Akaw_S 541 GGTGTGGTTGTTTATATAGATAAAATGGAAGTACTGAATAGTAATGTTAACCATAGTATT  600
Clau_S 541 GGTGTAGTTGTGTATATAGATGAAATGGAAGTGCTGAATAGCAAAGTTAACCATAGTATT  600
Pkil_S 541 GGTGTAGTTGTGTATATAGATGAAATGGAAGTACTGAATAGTAAGGTTAATCATAGTATT  600
Psoy_S 541 GGTGTAGTTGTGTATATAGATGAAATGGAAGTACTGAATAGTAAGGTTAATCATAGTATT  600
Vok    541 GGTGTAGTTGTGTATATAGATGAAATGGAAGTATTGAATAGTAAGGTTAATCATAGTATT  600
Cpac_S 541 GGCGTAGTCGTGTATATAGATGAAATGGAAGTACTTAATGGCAAAGTTAACCATACTATT  600
Cfau_S 541 GGCGTAGTCGTGTATATAGATGAAATGGAAATGCTTAATGGCAAGGTTAACCATACTATT  600
Cnau_S 541 GGCGTAGTCGTGTATATAGATGAAATGGAAGTGCTTAATGGCAAGGTTAACCATACTATT  600
Pste_S 541 GGCGTAGTCGTGTATATAGATGAAATGGAAGTGCTTAATGGCAAGGTTAACCATACTATT  600
Rma    541 GGCGTAGTCGTGTATATAGATGAAATGGAAGTGCTTAATGGCAAGGTTAACCATACTATT  600
Ifos_S 541 GGTGTAGTCGTGTATATAGATGAAATGGAAGTACTTAATGGCAAGGTTAACCATACTATT  600
Apha_S 541 GGTGTGGTTGTGTATATAGATGAAATGGAAGCACTTAATGGTAAGGTTAACCACAGTATT  600
Bsep_S 541 GGTGAGATTGTCTATTTAGATGATATGCAAGAATTAGAAGGTAAAACTCATCATACTATT  600
Akaw_S 181   G  V  V  V  Y  I  D  K  M  E  V  L  N  S  N  V  N  H  S  I  200
Clau_S 181   G  V  V  V  Y  I  D  E  M  E  V  L  N  S  K  V  N  H  S  I  200
Pkil_S 181   G  V  V  V  Y  I  D  E  M  E  V  L  N  S  K  V  N  H  S  I  200
Psoy_S 181   G  V  V  V  Y  I  D  E  M  E  V  L  N  S  K  V  N  H  S  I  200
Vok    181   G  V  V  V  Y  I  D  E  M  E  V  L  N  S  K  V  N  H  S  I  200
Cpac_S 181   G  V  V  V  Y  I  D  E  M  E  V  L  N  G  K  V  N  H  T  I  200
Cfau_S 181   G  V  V  V  Y  I  D  E  M  E  M  L  N  G  K  V  N  H  T  I  200
Cnau_S 181   G  V  V  V  Y  I  D  E  M  E  V  L  N  G  K  V  N  H  T  I  200
Pste_S 181   G  V  V  V  Y  I  D  E  M  E  V  L  N  G  K  V  N  H  T  I  200
Rma    181   G  V  V  V  Y  I  D  E  M  E  V  L  N  G  K  V  N  H  T  I  200
Ifos_S 181   G  V  V  V  Y  I  D  E  M  E  V  L  N  G  K  V  N  H  T  I  200
Apha_S 181   G  V  V  V  Y  I  D  E  M  E  A  L  N  G  K  V  N  H  S  I  200
Bsep_S 181   G  E  I  V  Y  L  D  D  M  Q  E  L  E  G  K  T  H  H  T  I  200
```

```
Akaw_S 601 GAAATTGTAGTTGATCGTTTAAAAATACGAGAAGGTATGATTTCACGTTTATCTGAATCA  660
Clau_S 601 GAAATTGTAGTTGATCGTTTAAAAATACGAGAAGATATGATTTCACGTTTATCTGAATCA  660
Pkil_S 601 GAAATTGTAGTTGATCGTTTAAAAATACGAGAAGATATGATTTCACGTTTATCTGAATCA  660
Psoy_S 601 GAAATTGTAGTTGATCGTTTAAAAATACGAGAAGATATGATTTCACGTTTATCTGAATCA  660
Vok    601 GAAATTGTAGTTGATCGTTTAAAAATACGAGAAGATATGATTTCACGTTTATCTGAATCA  660
Cpac_S 601 GAAATTATCATTGATCGTTTAAAAATACGAGAAGACATGATCTCACGTTTGTCTGAATCG  660
Cfau_S 601 GAAATTATCATTGATCGTTTAAAAATACGAGAAGACATGGCCTCACGCTTGTCTGAATCG  660
Cnau_S 601 GAAATTATCATTGATCGTTTAAAAATACGAGAAGGCATGGCCTCACGTTTGTCTGAATCG  660
Pste_S 601 GAAATTATCATTGATCGTTTAAAAATACGAGAAGACATGGCCTCACGTTTATCTGAATCG  660
Rma    601 GAAATTGTTATTGATCGTTTAAAAGTACGAGAAGACATGGCTTCACGCTTGTCTGAATCA  660
Ifos_S 601 GAAATTATCATTGATCGTTTAAAAATACGAGAAGATATGGCTTCACGCTTGTCTGAATCG  660
Apha_S 601 GAAATTATCATTGATCGTTTAAAAATACGAGAAGACATGGCTTCGCGTTTGTCTGAATCG  660
Bsep_S 601 GAGATTGTGATTGACCGTTTAAAAATCCGTAAAGACATTGCTTCTCGCCTATCTGAATCC  660
Akaw_S 201   E  I  V  V  D  R  L  K  I  R  E  G  M  I  S  R  L  S  E  S  220
Clau_S 201   E  I  V  V  D  R  L  K  I  R  E  D  M  I  S  R  L  S  E  S  220
Pkil_S 201   E  I  V  V  D  R  L  K  I  R  E  D  M  I  S  R  L  S  E  S  220
Psoy_S 201   E  I  V  V  D  R  L  K  I  R  E  D  M  I  S  R  L  S  E  S  220
Vok    201   E  I  V  V  D  R  L  K  I  R  E  D  M  I  S  R  L  S  E  S  220
Cpac_S 201   E  I  I  I  D  R  L  K  I  R  E  D  M  I  S  R  L  S  E  S  220
Cfau_S 201   E  I  I  I  D  R  L  K  I  R  E  D  M  A  S  R  L  S  E  S  220
Cnau_S 201   E  I  I  I  D  R  L  K  I  R  E  G  M  A  S  R  L  S  E  S  220
Pste_S 201   E  I  I  I  D  R  L  K  I  R  E  D  M  A  S  R  L  S  E  S  220
Rma    201   E  I  V  I  D  R  L  K  V  R  E  D  M  A  S  R  L  S  E  S  220
Ifos_S 201   E  I  I  I  D  R  L  K  I  R  E  D  M  A  S  R  L  S  E  S  220
Apha_S 201   E  I  I  I  D  R  L  K  I  R  E  D  M  A  S  R  L  S  E  S  220
Bsep_S 201   E  I  V  I  D  R  L  K  I  R  K  D  I  A  S  R  L  S  E  S  220
```

```
Akaw_S 661 TTAGAAACTGCACTTAATTTAAGTGCAGGTTTGGTACGTATTGCACCTATGGAAGATGAA  720
Clau_S 661 TTAGAAACTGCACTTAATTTAAGCGCAGGTTTGGTACGTATTGCACCTATGGAAGATGAA  720
Pkil_S 661 TTAGAAACTGCACTTAACTTAAGCGCAGGTTTGGTACGAATTGCACCTATGGAATGTGAA  720
Psoy_S 661 TTAGAAACTGCACTTAACTTAAGCGCAGGTTTGGTACGAATTGCACCTATGGAATGTGAA  720
Vok    661 TTAGAAACTGCACTTAATTTAAGCGCAGGTTTGGTACGCATTGCACCTATGGAATATGAA  720
Cpac_S 661 TTGGAAACTGCACTTAATTTGAGTGCTGGTTTGGTGCGAGTTGCACCTATGGAAGAAGAT  720
Cfau_S 661 TTGGAAACTGCACTTAATTTGAGCGCTGGTTTGGTGCGAGTTGTACCTATGGAAGAAGAT  720
Cnau_S 661 TTGGAAACTGCACTTAATTTGAGTGCTGGTTTGGTGCGAGTTGCATCTATGGAAGAAGAT  720
Pste_S 661 TTAGAAACTGCATTTAATTTGAGTGCTGGTTTGGTGCGAGTTGCACCTATGGAAGAAGAT  720
Rma    661 TTAGAAACTGCACTTAATTTGAGTGCTGGTTTAGTGCGAGTTGCATCTATGGAAGATGAG  720
Ifos_S 661 TTAGAAACTGCACTTAATTTAAGTGCAGGTTTGGTGCGAGTTGCATCTATGGATGATGAA  720
Apha_S 661 TTAGAAACTGCACTTAATCTAAGTGCAGGTTTGGTGCGAGTTGCATCTATGGAAGATGAG  720
Bsep_S 661 CTAGAGACCGCTTTAAATTTGAGTGCAGGATTGGTTAGAATTGCAGCCATAGAATTGGAT  720
Akaw_S 221   L  E  T  A  L  N  L  S  A  G  L  V  R  I  A  P  M  E  D  E  240
Clau_S 221   L  E  T  A  L  N  L  S  A  G  L  V  R  I  A  P  M  E  D  E  240
Pkil_S 221   L  E  T  A  L  N  L  S  A  G  L  V  R  I  A  P  M  E  C  E  240
Psoy_S 221   L  E  T  A  L  N  L  S  A  G  L  V  R  I  A  P  M  E  C  E  240
Vok    221   L  E  T  A  L  N  L  S  A  G  L  V  R  I  A  P  M  E  Y  E  240
Cpac_S 221   L  E  T  A  L  N  L  S  A  G  L  V  R  V  A  P  M  E  E  D  240
Cfau_S 221   L  E  T  A  L  N  L  S  A  G  L  V  R  V  V  P  M  E  E  D  240
Cnau_S 221   L  E  T  A  L  N  L  S  A  G  L  V  R  V  A  S  M  E  E  D  240
Pste_S 221   L  E  T  A  F  N  L  S  A  G  L  V  R  V  A  P  M  E  E  D  240
Rma    221   L  E  T  A  L  N  L  S  A  G  L  V  R  V  A  S  M  E  D  E  240
Ifos_S 221   L  E  T  A  L  N  L  S  A  G  L  V  R  V  A  S  M  D  D  E  240
Apha_S 221   L  E  T  A  L  N  L  S  A  G  L  V  R  V  A  S  M  E  D  E  240
Bsep_S 221   L  E  T  A  L  N  L  S  A  G  L  V  R  I  A  A  I  E  L  D  240
```

```
Akaw_S 721 TTTTCCTTGCAAGAAAAGGTATTTTCCGCTAAGTTTTCTTGTATTGAGTGTGGTTACTCA  780
Clau_S 721 TCATCCTTGCAAGAAAAGGTATTTTCCGATAAGTTTTCTTGTATTGAATGTGATTACTCA  780
Pkil_S 721 TCATCTTTACAAGAAAAGGTATTTTCCGCTAAGTTTTCTTGTATTGAGTGTGGTTACTCA  780
Psoy_S 721 TCATCTTTACAAGAAAAGGTATTTTCCGCTAAGTTTTCTTGTATTGAGTGTGGTTACTCA  780
Vok    721 TCATCTTTGCAAGAAAAGGTATTTTCCGCTAAGTTTTCTTGTATTGAATGCGGTTACTCA  780
Cpac_S 721 CCATCTTGGCAAGAAAAGGTGTTTTCTGCTAAATTTTCCTGCGTTGAGTGTGGCTACTCA  780
Cfau_S 721 CCATCTTGGCAAGAAAAGGTGTTTTCTGCTAAATTTTCTTGCGTTGAGTGTGGCTACTCA  780
Cnau_S 721 CCATTTTGGCAAGAAAAGGTGTTTTCTGCTAAATTTTCCTGCGTTGAATGTGGCTACTCA  780
Pste_S 721 CCATCTTGGCAAGAAAAGGTGTTTTCTGCTAAATTTTCTTGCGTTGAGTGTGGCTACTCA  780
Rma    721 CCTTCTTGGCAAGAAGGAGTGTTTTCTGCTAAATTTTCTTGTGTTGAATGTGGTTACTCA  780
Ifos_S 721 CCATCTTGGCAAGAAAAGGTGTTTTCTGCTAAATTTTCTTGTGTTGAGTGTGGCTACTCA  780
Apha_S 721 CCGTCTTGGCAAGACGAGGTGTTTTCTGCTAAATTTTCTTGTGTTGAGTGTGGCTACTCA  780
Bsep_S 721 TCAAGGCAAGAAGAATTGGTATTTTCTGCTAAATTTTCGTGCACTGAATGTGGCTACTCT  780
Akaw_S 241   F  S  L  Q  E  K  V  F  S  A  K  F  S  C  I  E  C  G  Y  S  260
Clau_S 241   S  S  L  Q  E  K  V  F  S  D  K  F  S  C  I  E  C  D  Y  S  260
Pkil_S 241   S  S  L  Q  E  K  V  F  S  A  K  F  S  C  I  E  C  G  Y  S  260
Psoy_S 241   S  S  L  Q  E  K  V  F  S  A  K  F  S  C  I  E  C  G  Y  S  260
Vok    241   S  S  L  Q  E  K  V  F  S  A  K  F  S  C  I  E  C  G  Y  S  260
Cpac_S 241   P  S  W  Q  E  K  V  F  S  A  K  F  S  C  V  E  C  G  Y  S  260
Cfau_S 241   P  S  W  Q  E  K  V  F  S  A  K  F  S  C  V  E  C  G  Y  S  260
Cnau_S 241   P  F  W  Q  E  K  V  F  S  A  K  F  S  C  V  E  C  G  Y  S  260
Pste_S 241   P  S  W  Q  E  K  V  F  S  A  K  F  S  C  V  E  C  G  Y  S  260
Rma    241   P  S  W  Q  E  G  V  F  S  A  K  F  S  C  V  E  C  G  Y  S  260
Ifos_S 241   P  S  W  Q  E  K  V  F  S  A  K  F  S  C  V  E  C  G  Y  S  260
Apha_S 241   P  S  W  Q  D  E  V  F  S  A  K  F  S  C  V  E  C  G  Y  S  260
Bsep_S 241   S  R  Q  E  E  L  V  F  S  A  K  F  S  C  T  E  C  G  Y  S  260
```

```
Akaw_S 781 TTAAGTGAATTGGAACCTAGACTTTTCTCATTTAATAATCCAGTTGGCGCTTGCCAAACT  840
Clau_S 781 TTAAGTGAATTGGAACCTAGACTTTTCTCATTTAATAATCCAGTTGGTGCTTGTCAAGCT  840
Pkil_S 781 TTAAGTGAATTGGAACCTAGGCTTTTCTCATTTAATAATCCAGTTGGTGCTTGCCAAACT  840
Psoy_S 781 TTAAGTGAATTGGAACCTAGGCTTTTCTCATTTAATAATCCAGTTGGTGCTTGCCAAACT  840
Vok    781 TTAAGTGAATTGGAGCCTAGGCTTTTCTCATTTAATAATCCAGTTGGTGCTTGCCAAACT  840
Cpac_S 781 TTAAGTGAATTAGAGCCTAGACTTTTCTCATTTAACAATCCAGTTGGCGCTTGTCAAACT  840
Cfau_S 781 TTAAGTGAATTAGAGCCTAGACTTTTCTCATTTAACAATCCAGTTGGTGCTTGTCAAACT  840
Cnau_S 781 TTAAGTGAATTGGAGCCTAGACTTTTCTCATTTAACAATCCAGTTGGCGCTTGTCAAACT  840
Pste_S 781 TTAAGTGAATTAGAGCCTAGACTTTTCTCATTTAACAATCCAGTTGGCGCTTGTCAAACT  840
Rma    781 TTAAGTGAATTGGAACCTAGGATTTTTTCATTTAACAATCCAGTTGGTGCTTGTCAAACT  840
Ifos_S 781 TTAAGTGAATTAGAGCCTAGACTTTTTTCATTTAACAATCCAGTTGGTGCTTGCCAAACT  840
Apha_S 781 TTGAGTGAATTGGAGCCTAGACTTTTCTCATTTAACAATCCAGTTGGTGCTTGTCAAACT  840
Bsep_S 781 TTAACTGAATTAGAGCCAAGATTATTTCGTTTAACAATCCAGTTGGCGCTTGTCAAAGT  840
Akaw_S 261   L  S  E  L  E  P  R  L  F  S  F  N  N  P  V  G  A  C  Q  T  280
Clau_S 261   L  S  E  L  E  P  R  L  F  S  F  N  N  P  V  G  A  C  Q  A  280
Pkil_S 261   L  S  E  L  E  P  R  L  F  S  F  N  N  P  V  G  A  C  Q  T  280
Psoy_S 261   L  S  E  L  E  P  R  L  F  S  F  N  N  P  V  G  A  C  Q  T  280
Vok    261   L  S  E  L  E  P  R  L  F  S  F  N  N  P  V  G  A  C  Q  T  280
Cpac_S 261   L  S  E  L  E  P  R  L  F  S  F  N  N  P  V  G  A  C  Q  T  280
Cfau_S 261   L  S  E  L  E  P  R  L  F  S  F  N  N  P  V  G  A  C  Q  T  280
Cnau_S 261   L  S  E  L  E  P  R  L  F  S  F  N  N  P  V  G  A  C  Q  T  280
Pste_S 261   L  S  E  L  E  P  R  L  F  S  F  N  N  P  V  G  A  C  Q  T  280
Rma    261   L  S  E  L  E  P  R  I  F  S  F  N  N  P  V  G  A  C  Q  T  280
Ifos_S 261   L  S  E  L  E  P  R  L  F  S  F  N  N  P  V  G  A  C  Q  T  280
Apha_S 261   L  S  E  L  E  P  R  L  F  S  F  N  N  P  V  G  A  C  Q  T  280
Bsep_S 261   L  T  E  L  E  P  R  L  F  S  F  N  N  P  V  G  A  C  Q  S  280
```

```
Akaw_S 841 TGTGATGGACTAGGTGTGAAAGATACATTTGATGAACAAAAAGTTGTAGCTAATCCTAGT  900
Clau_S 841 TGTAATGGACTGGGTGTAAAAGATATATTTGATGAACAAAAAGTTGTAGCTAGTCCTAGT  900
Pkil_S 841 TGTGATGGACTGGGTGTGAGAGATACATTTGATGAACAAAAAGTTGTAGTTAGTCCTAGT  900
Psoy_S 841 TGTGATGGACTGGGTGTGAGAGATACATTTGATGAACAAAAAGTTGTAGTTAGTCCTAGT  900
Vok    841 TGTGATGGACTGGGTGTGAGAGATACATTTGATGAACAAAAAGTTGTAGTTAGTCCTAGT  900
Cpac_S 841 TGCGATGGCTTAGGTGTAAAAGATATGTTTGATAAGCAAAAAGTTGTGGCCAATCCTAAC  900
Cfau_S 841 TGCGGTGGCTTGGGTGTAAAAGATACGTTTGATAAACAAAAAGTTGTGGCTAATCCTAAC  900
Cnau_S 841 TGTGATGGCTTGGGTGTAAAAGATACGTTTGATGAGCAAAAAGTTGTGGCCAATCCTAAC  900
Pste_S 841 TGCGATGGCTTGGGTGTAAAAGATACGTTTGATGAGCAAAAAGTTGTGGTTAGTCCTAAC  900
Rma    841 TGTGATGGCTTGGGTGTAAAAGATACATTTGATGAACAAAAAATCGTGGCTAATCCTAGT  900
Ifos_S 841 TGTGATGGATTGGGTGTAAAAGATACATTTGATGAGCAAAAAGTCGTGGTCAATCCTAGC  900
Apha_S 841 TGTGATGGATTAGGTGTAAAAGATACATTTGATGAGCAGAAAGTCGTGGCCAATCCTAGC  900
Bsep_S 841 TGCGATGGTTTGGGTATTAAAGAGATTTTTGATGACCACAAAGTCGTTAGCAATCCAACT  900
Akaw_S 281   C  D  G  L  G  V  K  D  T  F  D  E  Q  K  V  V  A  N  P  S  300
Clau_S 281   C  N  G  L  G  V  K  D  I  F  D  E  Q  K  V  V  A  S  P  S  300
Pkil_S 281   C  D  G  L  G  V  R  D  T  F  D  E  Q  K  V  V  V  S  P  S  300
Psoy_S 281   C  D  G  L  G  V  R  D  T  F  D  E  Q  K  V  V  V  S  P  S  300
Vok    281   C  D  G  L  G  V  R  D  T  F  D  E  Q  K  V  V  V  S  P  S  300
Cpac_S 281   C  D  G  L  G  V  K  D  M  F  D  K  Q  K  V  V  A  N  P  N  300
Cfau_S 281   C  G  G  L  G  V  K  D  T  F  D  K  Q  K  V  V  A  N  P  N  300
Cnau_S 281   C  D  G  L  G  V  K  D  T  F  D  E  Q  K  V  V  A  N  P  N  300
Pste_S 281   C  D  G  L  G  V  K  D  T  F  D  E  Q  K  V  V  V  S  P  N  300
Rma    281   C  D  G  L  G  V  K  D  T  F  D  E  Q  K  I  V  A  N  P  S  300
Ifos_S 281   C  D  G  L  G  V  K  D  T  F  D  E  Q  K  V  V  V  N  P  S  300
Apha_S 281   C  D  G  L  G  V  K  D  T  F  D  E  Q  K  V  V  A  N  P  S  300
Bsep_S 281   C  D  G  L  G  I  K  E  I  F  D  D  H  K  V  V  S  N  P  T  300
```

```
Akaw_S 901 ATGAGTTTAGCAGATGGGGCGGTTTATGGTTGGGGACGCTCAAATGCTTATTTTTATCAA  960
Clau_S 901 ATGAGTTTAGCAGATGGTGCAGTTTATGGTTGGGGACGCTCAAATGCTTATTTTTATCAA  960
Pkil_S 901 ATGAGTTTAGCAGATGGGGCAGTTTATGGTTGGGGACGCTCAAATGCTTATTTTTATCAA  960
Psoy_S 901 ATGAGTTTAGCAGATGGGGCAGTTTATGGTTGGGGACGCTCAAATGCTTATTTTTATCAA  960
Vok    901 ATGAGTTTAGCAGATGGGGCAGTTTATGGTTGGGGACGCTCAAATGCTTATTTTTATCAA  960
Cpac_S 901 ATGAGTTTAGCAGATGGTGCTATTTATGGTTGGGGGCGCTCAAATGCTTATTTTTATCAA  960
Cfau_S 901 ATGAGTTTGGCAGATGGTGCTGTTTATGGTTGGGGGCGCTCAAATGCTTATTTTTATCAA  960
Cnau_S 901 ATGAGTTTGGCAGATGGTGCTGTTTATGGTTGGGGGTGCTCAAATGCTTATTTTTATCAA  960
Pste_S 901 ATGAGTTTGGCAGATGGTGCTGTTTATGGTTGGGGGCGCTCAAATGCTTATTTTTATCAA  960
Rma    901 ATTAGTTTGGCAGATGGTGCTGTTTATGGCTGGGGACGTTCAAATGCTTATTTTTATCAA  960
Ifos_S 901 ATGAGTTTGGCGGATGGCGCTGTTTATGGCTGGGGACGCTCAAATGCTTATTTTTATCAA  960
Apha_S 901 ATGAGTTTGGCAGATGGTGCTGTTTATGGCTGGGGGCGCTCAAATGCTTATTTTTATCAA  960
Bsep_S 901 GCCAGTCTTGCCGAAGGTGCAATTTACGGTTGGGGTCGCTCAAATGCTTATTTCTACCAA  960
Akaw_S 301   M  S  L  A  D  G  A  V  Y  G  W  G  R  S  N  A  Y  F  Y  Q  320
Clau_S 301   M  S  L  A  D  G  A  V  Y  G  W  G  R  S  N  A  Y  F  Y  Q  320
Pkil_S 301   M  S  L  A  D  G  A  V  Y  G  W  G  R  S  N  A  Y  F  Y  Q  320
Psoy_S 301   M  S  L  A  D  G  A  V  Y  G  W  G  R  S  N  A  Y  F  Y  Q  320
Vok    301   M  S  L  A  D  G  A  V  Y  G  W  G  R  S  N  A  Y  F  Y  Q  320
Cpac_S 301   M  S  L  A  D  G  A  I  Y  G  W  G  R  S  N  A  Y  F  Y  Q  320
Cfau_S 301   M  S  L  A  D  G  A  V  Y  G  W  G  R  S  N  A  Y  F  Y  Q  320
Cnau_S 301   M  S  L  A  D  G  A  V  Y  G  W  G  C  S  N  A  Y  F  Y  Q  320
Pste_S 301   M  S  L  A  D  G  A  V  Y  G  W  G  R  S  N  A  Y  F  Y  Q  320
Rma    301   I  S  L  A  D  G  A  V  Y  G  W  G  R  S  N  A  Y  F  Y  Q  320
Ifos_S 301   M  S  L  A  D  G  A  V  Y  G  W  G  R  S  N  A  Y  F  Y  Q  320
Apha_S 301   M  S  L  A  D  G  A  V  Y  G  W  G  R  S  N  A  Y  F  Y  Q  320
Bsep_S 301   A  S  L  A  E  G  A  I  Y  G  W  G  R  S  N  A  Y  F  Y  Q  320
```

```
Akaw_S 961 ATATTAATTTTGGTAGGTAAGTATTACGGCTTTAGTATTGAAACACCTTATGAACAATTG 1020
Clau_S 961 ATATTAATTTTGGTAGGTAAGTATTACGGTTTTAGTATTGAAACACCTTATGAACAATTA 1020
Pkil_S 961 ATATTAATGTTAGTAGGTAAGTATTACGGCTTTAGTATTGAAACACCTTATGAACAATTA 1020
Psoy_S 961 ATATTAATGTTAGTAGGTAAGTATTACGGCTTTAGTATTGAAACACCTTATGAACAATTA 1020
Vok    961 ATATTAATGTTAGTAGGTAAGTATTACGGCTTTAGTATTGAAACACCTTATGAACAATTA 1020
Cpac_S 961 ATGCTAATGTTGGTAGGTAAGTATTACGGATTTAGTATTGAAACCCCTTATGAACAATTA 1020
Cfau_S 961 ATGCTAATGTTGGTAGGTAAGTATTACGGATTCAGTATTGAAACCCCTTATGAAGAATTA 1020
Cnau_S 961 ATGCTAATGTTGGTAGGTAAGTATTACGGATTTAGTATTGAAACCCCTTATGAACAATTA 1020
Pste_S 961 ATGCTAATCTTGGTAGGTAAGTATTACGGATTTAGTATTGAAACCCCTTATGAACAATTA 1020
Rma    961 ATGTTAATATTGGTAAGTAAGTATTACGACTTTAATATTGAAACTCCTTATGAACAATTA 1020
Ifos_S 961 ATGTTAATGTTGGTAGGTAAGTATTATGGCTTTAGTATTGAAACCCCTTATGAGCAATTA 1020
Apha_S 961 ATGCTAATGTTGGTAGGCAAGTATTACGGTTTTAGTATTGAAACCCCTTATGAACAATTA 1020
Bsep_S 961 ATCTTATTTCTAGTCGGGCAACATTACGGTTTTAGCGTTGATACCCCTTACGAAGAACTT 1020
Akaw_S 321   I  L  I  L  V  G  K  Y  Y  G  F  S  I  E  T  P  Y  E  Q  L  340
Clau_S 321   I  L  I  L  V  G  K  Y  Y  G  F  S  I  E  T  P  Y  E  Q  L  340
Pkil_S 321   I  L  M  L  V  G  K  Y  Y  G  F  S  I  E  T  P  Y  E  Q  L  340
Psoy_S 321   I  L  M  L  V  G  K  Y  Y  G  F  S  I  E  T  P  Y  E  Q  L  340
Vok    321   I  L  M  L  V  G  K  Y  Y  G  F  S  I  E  T  P  Y  E  Q  L  340
Cpac_S 321   M  L  M  L  V  G  K  Y  Y  G  F  S  I  E  T  P  Y  E  Q  L  340
Cfau_S 321   M  L  M  L  V  G  K  Y  Y  G  F  S  I  E  T  P  Y  E  E  L  340
Cnau_S 321   M  L  M  L  V  G  K  Y  Y  G  F  S  I  E  T  P  Y  E  Q  L  340
Pste_S 321   M  L  I  L  V  G  K  Y  Y  G  F  S  I  E  T  P  Y  E  Q  L  340
Rma    321   M  L  I  L  V  S  K  Y  Y  D  F  N  I  E  T  P  Y  E  Q  L  340
Ifos_S 321   M  L  M  L  V  G  K  Y  Y  G  F  S  I  E  T  P  Y  E  Q  L  340
Apha_S 321   M  L  M  L  V  G  K  Y  Y  G  F  S  I  E  T  P  Y  E  Q  L  340
Bsep_S 321   I  L  F  L  V  G  Q  H  Y  G  F  S  V  D  T  P  Y  E  E  L  340
```

```
Akaw_S 1021 AGTGATAAACACAAAAAAATTGTTCTTTATGGGAGTGGCACAGATGACATTGACTTTGCA 1080
Clau_S 1021 AGTGATAAACACAAAAAAATTGTCCTTTATGGGAGTGGCACAGATGACATTGACTTTTCA 1080
Pkil_S 1021 AGTGATAAGCACAAAAAAATTGTCCTTTATGGGAGTGGTACAGATGATATTGACTTTTCA 1080
Psoy_S 1021 AGTGATAAGCACAAAAAAATTGTCCTTTATGGGAGTGGTACAGATGATATTGACTTTTCA 1080
Vok    1021 AGTAATAAGCACAAAAAAATTGTCCTTTATGGGAGTGGCACAGATGATATTGACTTTTCA 1080
Cpac_S 1021 AGCGATAAGCACAAAAAAATTGTTCTTTATGGAAGTGGTACAGATGACATTGATTTTTCC 1080
Cfau_S 1021 AGCGATAAGCACAAAAAAATTGTTCTTTATGGAAGTGGTACAGATGACATTGATTTTTCC 1080
Cnau_S 1021 AGCGATAAGCATAAAAAAATTGTTCTTTATGGAAGTGGTACAGATGACATTGATTTTTCC 1080
Pste_S 1021 AGCGATAAGCACAAAAAAATTGTTCTTTACGGAAGTGGTACAGATGACATTGATTTTTCC 1080
Rma    1021 AGTGATAAGCACAAAAAAATTGTTCTTTATGGAAGTGGCACAGATGACATTGATTTTTCC 1080
Ifos_S 1021 AGTGATAAGCACAAAAAAATTGTCCTTTACGGGAGTGGTACAGATGACATTGACTTTTCT 1080
Apha_S 1021 AGTGATAAGCACAAAAAAATTGTCCTTTATGGGAGTGGTGTAGATGATATTGATTTTTCC 1080
Bsep_S 1021 AGTGAGAAACATCAAAAAATCATCCTCCACGGTAGTGGTAACGACGAAATTGACTTTTCA 1080
Akaw_S  341   S  D  K  H  K  K  I  V  L  Y  G  S  G  T  D  D  I  D  F  A  360
Clau_S  341   S  D  K  H  K  K  I  V  L  Y  G  S  G  T  D  D  I  D  F  S  360
Pkil_S  341   S  D  K  H  K  K  I  V  L  Y  G  S  G  T  D  D  I  D  F  S  360
Psoy_S  341   S  D  K  H  K  K  I  V  L  Y  G  S  G  T  D  D  I  D  F  S  360
Vok     341   S  N  K  H  K  K  I  V  L  Y  G  S  G  T  D  D  I  D  F  S  360
Cpac_S  341   S  D  K  H  K  K  I  V  L  Y  G  S  G  T  D  D  I  D  F  S  360
Cfau_S  341   S  D  K  H  K  K  I  V  L  Y  G  S  G  T  D  D  I  D  F  S  360
Cnau_S  341   S  D  K  H  K  K  I  V  L  Y  G  S  G  T  D  D  I  D  F  S  360
Pste_S  341   S  D  K  H  K  K  I  V  L  Y  G  S  G  T  D  D  I  D  F  S  360
Rma     341   S  D  K  H  K  K  I  V  L  Y  G  S  G  T  D  D  I  D  F  S  360
Ifos_S  341   S  D  K  H  K  K  I  V  L  Y  G  S  G  T  D  D  I  D  F  S  360
Apha_S  341   S  D  K  H  K  K  I  V  L  Y  G  S  G  V  D  D  I  D  F  S  360
Bsep_S  341   S  E  K  H  Q  K  I  I  L  H  G  S  G  N  D  E  I  D  F  S  360
```

```
Akaw_S 1081 AGAATCAAAGGACGTAATGGTTGGTCTAATAAAGCCAAGCCATTTGAGGGCATTATTCCA 1140
Clau_S 1081 AGAATCAAAGGACGTAAGGGTTGGTCTAATAAAGCTAAGCCATTTGAGGGCATCATTCCA 1140
Pkil_S 1081 AGAATTAAAGGACGTAAGGGTTGGTCTAATAAAGCCAAGCCATTTGAGGGTATTATTCCA 1140
Psoy_S 1081 AGAATTAAAGGACGTAAGGGTTGGTCTAATAAAGCCAAGCCATTTGAGGGTATTATTCCA 1140
Vok    1081 AGAATCAAAGGACGTAAGGGTTGGTCTAATAAAGCTAAGCCATTTGAGGGTATTATTCCA 1140
Cpac_S 1081 AAAATAAAAGGGCGTAAAAGTTGGTCTAATAAGGCTAAGCCATTTGAAGGCATTATTCCA 1140
Cfau_S 1081 AAAATAAAAGGACGTAAAGGCTGGTCTAATAAGGCTAAGCCATTTGAAGGCATTATTCCA 1140
Cnau_S 1081 AAAATAAAAGGGCGTAAAGGTTGGTCTAATAAGGCTAAGCCATTTGAAGGTATTATTCCC 1140
Pste_S 1081 AAAATAAAAGGACGTAAAGGCTGGTCTAATAAGGCTAAGCCGTTTGAAGGCATTATTCCA 1140
Rma    1081 AAAATAAAAGGACGTAAAGGTTGGTCTAATAAAGCCAAGCCATTTGAAGGTATTATTCCA 1140
Ifos_S 1081 AAAATAAAAGGACGTAAAGGCTGGTCTAATAAAGCCAAGCCATTTGAAGGCATTATTCCA 1140
Apha_S 1081 AAAATAAAAGGGCGTAAAGGCTGGTCTAATAAGGCTAAGCCATTTGAAGGCATTATTCCA 1140
Bsep_S 1081 AAAATCAAAGGACGCAAAGGCTGGTCAAACAAAGCCAAACCCTTTGAAGGTGTGATTCCA 1140
Akaw_S  361   R  I  K  G  R  N  G  W  S  N  K  A  K  P  F  E  G  I  I  P  380
Clau_S  361   R  I  K  G  R  K  G  W  S  N  K  A  K  P  F  E  G  I  I  P  380
Pkil_S  361   R  I  K  G  R  K  G  W  S  N  K  A  K  P  F  E  G  I  I  P  380
Psoy_S  361   R  I  K  G  R  K  G  W  S  N  K  A  K  P  F  E  G  I  I  P  380
Vok     361   R  I  K  G  R  K  G  W  S  N  K  A  K  P  F  E  G  I  I  P  380
Cpac_S  361   K  I  K  G  R  K  S  W  S  N  K  A  K  P  F  E  G  I  I  P  380
Cfau_S  361   K  I  K  G  R  K  G  W  S  N  K  A  K  P  F  E  G  I  I  P  380
Cnau_S  361   K  I  K  G  R  K  G  W  S  N  K  A  K  P  F  E  G  I  I  P  380
Pste_S  361   K  I  K  G  R  K  G  W  S  N  K  A  K  P  F  E  G  I  I  P  380
Rma     361   K  I  K  G  R  K  G  W  S  N  K  A  K  P  F  E  G  I  I  P  380
Ifos_S  361   K  I  K  G  R  K  G  W  S  N  K  A  K  P  F  E  G  I  I  P  380
Apha_S  361   K  I  K  G  R  K  G  W  S  N  K  A  K  P  F  E  G  I  I  P  380
Bsep_S  361   K  I  K  G  R  K  G  W  S  N  K  A  K  P  F  E  G  V  I  P  380
```

```
Akaw_S 1141 AGAATGATACGTCGTTATGAAACAAGTGAGATTCACAGCGTTAAAGAAGAGCTTTCTCGT 1200
Clau_S 1141 AGAATGATGCGTCGTTATGAAACAAGTGAGATTCATAGCGTTAGAGAAGAGCTTTCTCGT 1200
Pkil_S 1141 AGAATGATGCGTCGTTATGAAACAAGTGAGATTAATAGTGTTAGAGAAGAGCTTTCTCGT 1200
Psoy_S 1141 AGAATGATGCGTCGTTATGAAACAAGTGAGATTAATAGTGTTAGAGAAGAGCTTTCTCGT 1200
Vok    1141 AGAATGATGCGTCGTTATGAAACAAGTGAGATTAATAGCGTTAGAGAAGAGCTTTCTCGT 1200
Cpac_S 1141 AGAATGATGCGACGTTATGAAGAAAGTGAGATTCGTAGTGTTAGAGAAGAGCTTTCCCGT 1200
Cfau_S 1141 AGAATGATGCGACGTTATGAAGAAAGTGAGATTCGTAGTGTTAGAGAAGAGCTTTCTCGT 1200
Cnau_S 1141 AGAATGATGCGACGTTATGAAGAAAGTGAGATTCGTAGTGTTAGAGAAGAGCTTTCCCGT 1200
Pste_S 1141 AGAATGATGCGACGTTATGAAGAAAGTGAGATTCGTAGTGTTAGAGAAGAGCTTTCTCGT 1200
Rma    1141 AGAATGATACGTCGTTATGAAGAAAGTGATATTCGTAGTGTTAGAGAAGAACTTTCTCGT 1200
Ifos_S 1141 AGAATGATGCGCCGTTATGAAGAAAGTGAGATTCGTAGTGTTAGAGAAGAGCTTTCTCGT 1200
Apha_S 1141 AGAATGATGCGACGTTATGAAGAAAGTGAGATTCGTAATGTTAGAGAAGAGCTTTCTCGT 1200
Bsep_S 1141 AGAATGATGCGTCGCTACGAAGAAAGTGAAATCCGTTCCGTGCGTGAAGAATTATCTCGT 1200
Akaw_S  381   R  M  I  R  R  Y  E  T  S  E  I  H  S  V  K  E  E  L  S  R  400
Clau_S  381   R  M  M  R  R  Y  E  T  S  E  I  H  S  V  R  E  E  L  S  R  400
Pkil_S  381   R  M  M  R  R  Y  E  T  S  E  I  N  S  V  R  E  E  L  S  R  400
Psoy_S  381   R  M  M  R  R  Y  E  T  S  E  I  N  S  V  R  E  E  L  S  R  400
Vok     381   R  M  M  R  R  Y  E  T  S  E  I  N  S  V  R  E  E  L  S  R  400
Cpac_S  381   R  M  M  R  R  Y  E  E  S  E  I  R  S  V  R  E  E  L  S  R  400
Cfau_S  381   R  M  M  R  R  Y  E  E  S  E  I  R  S  V  R  E  E  L  S  R  400
Cnau_S  381   R  M  M  R  R  Y  E  E  S  E  I  R  S  V  R  E  E  L  S  R  400
Pste_S  381   R  M  M  R  R  Y  E  E  S  E  I  R  S  V  R  E  E  L  S  R  400
Rma     381   R  M  I  R  R  Y  E  E  S  D  I  R  S  V  R  E  E  L  S  R  400
Ifos_S  381   R  M  M  R  R  Y  E  E  S  E  I  R  S  V  R  E  E  L  S  R  400
Apha_S  381   R  M  M  R  R  Y  E  E  S  E  I  R  N  V  R  E  E  L  S  R  400
Bsep_S  381   R  M  M  R  R  Y  E  E  S  E  I  R  S  V  R  E  E  L  S  R  400
```

```
Akaw_S 1201 TATGTTGTTAGTAAGGATTGCACTGAATGTCATGGCGATAGATTAAATGAATCAGCTAGA 1260
Clau_S 1201 TATGTTGTTAGTAAAGATTGTACTGAATGTCATGGCGATAGATTAAATGAATCAGCTAGA 1260
Pkil_S 1201 TATGTTGTTAGTAAAAATTGCACTGAATGTCATGGCGATAGATTAAATGAATCAGCTAGA 1260
Psoy_S 1201 TATGTTGTTAGTAAAAATTGCACTGAATGTCATGGCGATAGATTAAATGAATCAGCTAGA 1260
Vok    1201 TATGTTGTTAGTAAGAATTGTACTGAATGTCATGGCGATAGATTAAATGAATCAGCTAGA 1260
Cpac_S 1201 TATGTAGTGAGCAAGGATTGTGGGCAATGTCGTGGTGATAGGTTGAATGAATCAGCTAGA 1260
Cfau_S 1201 TATGTAGTGAGCAAGGATTGTGAGCAATGTCATGGTGATAGGTTGAATGAATCAGCTAGA 1260
Cnau_S 1201 TATGTGGTGAGCAAAGATTGTGGGCAATGCCATGGTGATAGATTGAATGAATCAGCTAGA 1260
Pste_S 1201 TATGTAGTGAGCAAGGATTGTGGGCAATGCCATGGTGATAGGTTGAATGAATCAGCTAGA 1260
Rma    1201 TATGTGGTGAGCAAGGATTGTGTGCAATGTCATGGCGATAGATTGAATGAATCGGCTAGA 1260
Ifos_S 1201 TATGTGGTGAGCAAGGATTGCGGACAATGCCATGGCGATAGGTTGAATGAATCGGCTAGA 1260
Apha_S 1201 TATGTAGTGAGTAAGGATTGCGGACAATGCCATGGCGATAGGTTGAATGAATCGGCTAGA 1260
Bsep_S 1201 TATGTGGTTAGTAAAAATTGTGGCAGTTGTAACGGTAGTCGTTTAAATGAATCTGCCAGA 1260
Akaw_S  401   Y  V  V  S  K  D  C  T  E  C  H  G  D  R  L  N  E  S  A  R  420
Clau_S  401   Y  V  V  S  K  D  C  T  E  C  H  G  D  R  L  N  E  S  A  R  420
Pkil_S  401   Y  V  V  S  K  N  C  T  E  C  H  G  D  R  L  N  E  S  A  R  420
Psoy_S  401   Y  V  V  S  K  N  C  T  E  C  H  G  D  R  L  N  E  S  A  R  420
Vok     401   Y  V  V  S  K  N  C  T  E  C  H  G  D  R  L  N  E  S  A  R  420
Cpac_S  401   Y  V  V  S  K  D  C  G  Q  C  R  G  D  R  L  N  E  S  A  R  420
Cfau_S  401   Y  V  V  S  K  D  C  E  Q  C  H  G  D  R  L  N  E  S  A  R  420
Cnau_S  401   Y  V  V  S  K  D  C  G  Q  C  H  G  D  R  L  N  E  S  A  R  420
Pste_S  401   Y  V  V  S  K  D  C  G  Q  C  H  G  D  R  L  N  E  S  A  R  420
Rma     401   Y  V  V  S  K  D  C  V  Q  C  H  G  D  R  L  N  E  S  A  R  420
Ifos_S  401   Y  V  V  S  K  D  C  G  Q  C  H  G  D  R  L  N  E  S  A  R  420
Apha_S  401   Y  V  V  S  K  D  C  G  Q  C  H  G  D  R  L  N  E  S  A  R  420
Bsep_S  401   Y  V  V  S  K  N  C  G  S  C  N  G  S  R  L  N  E  S  A  R  420
```

```
Akaw_S 1261 AATGTGTTTATTGATGAGCAAAATTTATCAAATATTACTAAGCTTTCTATTGTTGATATT 1320
Clau_S 1261 AATGTGTTTATTGATGAGAAAAATTTATCAAATATTACTAAGCTTTCTATTGCTGATATT 1320
Pkil_S 1261 AATGTGTTTATTGATGAGCAAAATTTATCAAATATTACTAAGCTTTCTATTGCCGATATT 1320
Psoy_S 1261 AATGTGTTTATTGATGAGCAAAATTTATCAAATATTACTAAGCTTTCTATTGCCGATATT 1320
Vok    1261 AATGTGTTTATTGATGAGCAAAATTTATCAAATATTACTAAGCTTTCTATTGCTGATATT 1320
Cpac_S 1261 AATGTGTTTATTGATGGACAGAACTTATCAAATATCACCAAACTTTCTATTGCTGATATT 1320
Cfau_S 1261 AATGTGTTTATTGATGGGCAGAACTTATCAAATATCACCAAACTTTCTATTGCTGATATT 1320
Cnau_S 1261 AATGTGTTTATTGATGGGCAGAACTTATCAAATATCACCAAACTTTCTATTGCTGATATT 1320
Pste_S 1261 AATGTGTTTATTGATGGGCAGAACTTATCAAACATCATCAAACTTTCTATTGCTGATATT 1320
Rma    1261 AATGTATTTATTGATGGGAAAAACTTATCAAATATTACCAAACTTTCCATTGCTGATATT 1320
Ifos_S 1261 AATGTATTCATTGATGGGCAAAACTTATCAAACATCACCAAACTTTCCATTGCTGATATT 1320
Apha_S 1261 AATGTATTTATTGATGGGCAAAGCTTATCAAACATCACCAAACTTTCTATTGCTGATATT 1320
Bsep_S 1261 AATGTTTTTATCGGCGAATATAATTTATCCGATATCAGTAAACTCTCTATTGCTAATAGC 1320
Akaw_S  421   N  V  F  I  D  E  Q  N  L  S  N  I  T  K  L  S  I  V  D  I  440
Clau_S  421   N  V  F  I  D  E  K  N  L  S  N  I  T  K  L  S  I  A  D  I  440
Pkil_S  421   N  V  F  I  D  E  Q  N  L  S  N  I  T  K  L  S  I  A  D  I  440
Psoy_S  421   N  V  F  I  D  E  Q  N  L  S  N  I  T  K  L  S  I  A  D  I  440
Vok     421   N  V  F  I  D  E  Q  N  L  S  N  I  T  K  L  S  I  A  D  I  440
Cpac_S  421   N  V  F  I  D  G  Q  N  L  S  N  I  T  K  L  S  I  A  D  I  440
Cfau_S  421   N  V  F  I  D  G  Q  N  L  S  N  I  T  K  L  S  I  A  D  I  440
Cnau_S  421   N  V  F  I  D  G  Q  N  L  S  N  I  T  K  L  S  I  A  D  I  440
Pste_S  421   N  V  F  I  D  G  Q  N  L  S  N  I  I  K  L  S  I  A  D  I  440
Rma     421   N  V  F  I  D  G  K  N  L  S  N  I  T  K  L  S  I  A  D  I  440
Ifos_S  421   N  V  F  I  D  G  Q  N  L  S  N  I  T  K  L  S  I  A  D  I  440
Apha_S  421   N  V  F  I  D  G  Q  S  L  S  N  I  T  K  L  S  I  A  D  I  440
Bsep_S  421   N  V  F  I  G  E  Y  N  L  S  D  I  S  K  L  S  I  A  N  S  440
```

```
Akaw_S 1321 TATAGTTTTCTAAAAGAGGTAAAGCTGGAAGGAGTTCGTGGTCAAATTGCGGAAAAAATT 1380
Clau_S 1321 TATAGTTTTTTAAAAGAGGTAAAGCTGGAAGGAGTTCGTGGTCAAATTGCGGAAAAAATT 1380
Pkil_S 1321 TATAGTTTTTTAAAAGAGGTAAAGCTGGAAGGAGTTCGTGGTCAAATTGCGGAAAAAATT 1380
Psoy_S 1321 TATAGTTTTTTAAAAGAGGTAAAGCTGGAAGGAGTTCGTGGTCAAATTGCGGAAAAAATT 1380
Vok    1321 TATAGTTTTTTAAAAGAGGTAAAGCTGGAAGGAGTTCGTGGTCAAATTGCGGAAAAAATT 1380
Cpac_S 1321 TATGATTTTTTCAAAGAGTTGAAGTTGGAAGGCGCTCGTGGTCAAATTGCGGATAAGATT 1380
Cfau_S 1321 TATGATTTTCTCAAAGAGTTGAAGTTGGAAGGTGCTCGTGGTCAAATTGCGGATAAGATT 1380
Cnau_S 1321 TATGATTTTTTCAAAAAGTTGAAGTTGGAAGGTGCTCGTGGTCAAATTGCGGATAAGATT 1380
Pste_S 1321 TATGATTTTTTTAAAGAATTGAAGTTAGAAGGCACTCGTGGTCAAATTGCGGATAAGATT 1380
Rma    1321 TATGATTTTTTCAAAGAATTAAAGCTAGAAGGGGCTCGTGGTCAAATTGCAGATAAGATT 1380
Ifos_S 1321 TATGATTTTTTCAAAGAGTTGAAGTTGGAAGGAGCTCGTGGTCAAATTGCGGATAAGATT 1380
Apha_S 1321 TATGATTTTTTCAAAGAGTTGAAGTTGAAAGGCGCTCGTGGTCAAATTGCGGATAAGATT 1380
Bsep_S 1321 CATGATTTTTTCAAACATTTAAAACTAGATGGTGTACGAGGTGAGATTGCCGAAAAAATC 1380
Akaw_S  441   Y  S  F  L  K  E  V  K  L  E  G  V  R  G  Q  I  A  E  K  I  460
Clau_S  441   Y  S  F  L  K  E  V  K  L  E  G  V  R  G  Q  I  A  E  K  I  460
Pkil_S  441   Y  S  F  L  K  E  V  K  L  E  G  V  R  G  Q  I  A  E  K  I  460
Psoy_S  441   Y  S  F  L  K  E  V  K  L  E  G  V  R  G  Q  I  A  E  K  I  460
Vok     441   Y  S  F  L  K  E  V  K  L  E  G  V  R  G  Q  I  A  E  K  I  460
Cpac_S  441   Y  D  F  F  K  E  L  K  L  E  G  A  R  G  Q  I  A  D  K  I  460
Cfau_S  441   Y  D  F  L  K  E  L  K  L  E  G  A  R  G  Q  I  A  D  K  I  460
Cnau_S  441   Y  D  F  F  K  K  L  K  L  E  G  A  R  G  Q  I  A  D  K  I  460
Pste_S  441   Y  D  F  F  K  E  L  K  L  E  G  T  R  G  Q  I  A  D  K  I  460
Rma     441   Y  D  F  F  K  E  L  K  L  E  G  A  R  G  Q  I  A  D  K  I  460
Ifos_S  441   Y  D  F  F  K  E  L  K  L  E  G  A  R  G  Q  I  A  D  K  I  460
Apha_S  441   Y  D  F  F  K  E  L  K  L  K  G  A  R  G  Q  I  A  D  K  I  460
Bsep_S  441   H  D  F  F  K  H  L  K  L  D  G  V  R  G  E  I  A  E  K  I  460
```

```
Akaw_S 1381 TTAAAAGAAATTATTCAACGTTTAGAATTTTTAATTAATGTGGGGTTGGAGTATTTAAGT 1440
Clau_S 1381 TTAAAAGAAATTATTCAACGTTTAGAATTTTTAATTAATGTTGGATTGGAATATTTAAGT 1440
Pkil_S 1381 TTAAAAGAAATTATTCAACGTTTAGAATTTTTAATGAATGTGGGGTTGGAGTATTTAAGT 1440
Psoy_S 1381 TTAAAAGAAATTATTCAACGTTTAGAATTTTTAATGAATGTGGGGTTGGAGTATTTAAGT 1440
Vok    1381 TTAAAAGAAATTATTCAACGTTTAGAATTTTTAATTAATGTGGGGTTAGAGTATTTAAGT 1440
Cpac_S 1381 TTAAAAGAAACTGTTCAGCGTTTGGCATTTTTAATTAATGTAGGTTTAGAATATTTAACT 1440
Cfau_S 1381 TTAAAAGAAATTGTTCAGCGTTTGGCATTTTTAATTAATGTAGGTTTGGAATATTTAAGT 1440
Cnau_S 1381 TTAAAAGAAATTGTTCAGCGTTTGGCATTTCTAATTAATGTAGGTTTGGAATATTTAAGT 1440
Pste_S 1381 TTAAAAGAAATTGTTCAGCGTTTGGTATTTTTAATTAATGTAGGTTTGGAATATTTAAGT 1440
Rma    1381 TTAAAAGAAATTGTTCAGCGTTTGGCATTTCTAATTAATGTAGGGTTGGAATATTTAAGT 1440
Ifos_S 1381 TTAAAAGAAATTGTCCAACGTTTAGCGTTTTTAATTAACGTGGGTTTGGAATATTTAAGC 1440
Apha_S 1381 TTAAAAGAAATTGTTCAGCGTTTGGCATTTTTAATTAACGTAGGTTTGGAATATTTAAGT 1440
Bsep_S 1381 CTAAAAGAAATCATTCAACGCTTAGAATTTTTACTTAATGTTGGTTTAGAATATCTCAGT 1440
Akaw_S  461   L  K  E  I  I  Q  R  L  E  F  L  I  N  V  G  L  E  Y  L  S  480
Clau_S  461   L  K  E  I  I  Q  R  L  E  F  L  I  N  V  G  L  E  Y  L  S  480
Pkil_S  461   L  K  E  I  I  Q  R  L  E  F  L  M  N  V  G  L  E  Y  L  S  480
Psoy_S  461   L  K  E  I  I  Q  R  L  E  F  L  M  N  V  G  L  E  Y  L  S  480
Vok     461   L  K  E  I  I  Q  R  L  E  F  L  I  N  V  G  L  E  Y  L  S  480
Cpac_S  461   L  K  E  T  V  Q  R  L  A  F  L  I  N  V  G  L  E  Y  L  T  480
Cfau_S  461   L  K  E  I  V  Q  R  L  A  F  L  I  N  V  G  L  E  Y  L  S  480
Cnau_S  461   L  K  E  I  V  Q  R  L  A  F  L  I  N  V  G  L  E  Y  L  S  480
Pste_S  461   L  K  E  I  V  Q  R  L  V  F  L  I  N  V  G  L  E  Y  L  S  480
Rma     461   L  K  E  I  V  Q  R  L  A  F  L  I  N  V  G  L  E  Y  L  S  480
Ifos_S  461   L  K  E  I  V  Q  R  L  A  F  L  I  N  V  G  L  E  Y  L  S  480
Apha_S  461   L  K  E  I  V  Q  R  L  A  F  L  I  N  V  G  L  E  Y  L  S  480
Bsep_S  461   L  K  E  I  I  Q  R  L  E  F  L  L  N  V  G  L  E  Y  L  S  480
```

ABC_UvrA_I domain (464-576)

```
Akaw_S 1441 CTTGATCGTAGAGCAAATAGCCTGTCAGGTGGTGAAGCACAGCGTATTCGTCTAGCTAGT 1500
Clau_S 1441 CTTGATCGTAGAGCAAATAGCTTATCAGGTGGTGAAGCACAGCGTATTCGTTTAGCTAGT 1500
Pkil_S 1441 CTTGATCGTAGGGCAAATAGCTTATCAGGTGGTGAAGCACAGCGTATTCGCTTAGCTAGT 1500
Psoy_S 1441 CTTGATCGTAGGGCAAATAGCTTATCAGGTGGTGAAGCACAGCGTATTCGCTTAGCTAGT 1500
Vok    1441 CTTGATCGTAGAGCAAATAGCTTATCAGGTGGTGAGGCACAGCGTATTCGCTTAGCTAGT 1500
Cpac_S 1441 CTTGACCGTAGAGCAAATAGTTTGTCAGGTGGTGAAGCGCAACGTATTCGCTTGGCTAGT 1500
Cfau_S 1441 CTTGACCGTAGAGCAAATAGTTTGTCAGGTGGTGAAGCGCAGCGCATTCGCTTGGCTAGT 1500
Cnau_S 1441 CTTGACCGTAGAGCAAATAGTTTGTCAGGTGGTGAAGCGCAGCGTATTCGCTTGGCTAGT 1500
Pste_S 1441 CTTGACCGTAGAGCAAATAGTTTGTCAGGTGGTGAAGCACAGCGCATTCGCTTGGCTAGT 1500
Rma    1441 CTTAATCGTAGAGCAAATAGCTTGTCAGGTGGTGAAGCGCAGCGTATTCGCTTGGCCAGT 1500
Ifos_S 1441 CTTGACCGTAGAGCAAATAGTTTGTCAGGCGGTGAGGCGCAGCGCATTCGCTTAGCTAGT 1500
Apha_S 1441 CTTGACCGTAGAGCAAATAGTTTGTCAGGTGGTGAAGCGCAGCGCATTCGCTTGGCCAGC 1500
Bsep_S 1441 TTAGAGCGCCGTTCAGGTACTTTGTCAGGTGGAGAAGCACAACGCATTCGTCTTGCCAGT 1500
Akaw_S  481   L  D  R  R  A  N  S  L  S  G  G  E  A  Q  R  I  R  L  A  S  500
Clau_S  481   L  D  R  R  A  N  S  L  S  G  G  E  A  Q  R  I  R  L  A  S  500
Pkil_S  481   L  D  R  R  A  N  S  L  S  G  G  E  A  Q  R  I  R  L  A  S  500
Psoy_S  481   L  D  R  R  A  N  S  L  S  G  G  E  A  Q  R  I  R  L  A  S  500
Vok     481   L  D  R  R  A  N  S  L  S  G  G  E  A  Q  R  I  R  L  A  S  500
Cpac_S  481   L  D  R  R  A  N  S  L  S  G  G  E  A  Q  R  I  R  L  A  S  500
Cfau_S  481   L  D  R  R  A  N  S  L  S  G  G  E  A  Q  R  I  R  L  A  S  500
Cnau_S  481   L  D  R  R  A  N  S  L  S  G  G  E  A  Q  R  I  R  L  A  S  500
Pste_S  481   L  D  R  R  A  N  S  L  S  G  G  E  A  Q  R  I  R  L  A  S  500
Rma     481   L  N  R  R  A  N  S  L  S  G  G  E  A  Q  R  I  R  L  A  S  500
Ifos_S  481   L  D  R  R  A  N  S  L  S  G  G  E  A  Q  R  I  R  L  A  S  500
Apha_S  481   L  D  R  R  A  N  S  L  S  G  G  E  A  Q  R  I  R  L  A  S  500
Bsep_S  481   L  E  R  R  S  G  T  L  S  G  G  E  A  Q  R  I  R  L  A  S  500
```

ABC_UvrA_I domain (464-576)

```
Akaw_S 1501 CAGATTGGTGCAGGATTGATGGGTGTTTTGTATGTGCTTGATGAGCCATCAATAGGCTTA 1560
Clau_S 1501 CAGATTGGTGTAGGATTGATGGGTGTCTTGTATGTGCTTGATGAGCCATCAATAGGTTTA 1560
Pkil_S 1501 CAGATTGGTGCAGGATTGATGGGTGTCTTGTATGTGCTTGATGAACCATCAATAGGTTTA 1560
Psoy_S 1501 CAGATTGGTGCAGGATTGATGGGTGTCTTGTATGTGCTTGATGAACCATCAATAGGTTTA 1560
Vok    1501 CAGATTGGTGCAGGATTGATGGGTGTCTTGTATGTACTTGATGAACCATCAATAGGTTTA 1560
Cpac_S 1501 CAGATTGGTGCAGGGCTGATGGGTGTGCTGTATGTGCTTGATGAACCATCAATTGGTTTG 1560
Cfau_S 1501 CAGATTGGTGCGGGGCTGATGGGTGTGCTGTATGTGCTTGATGAACCATCAATCGGTTTG 1560
Cnau_S 1501 CAGATTGGTTCAGGGCTGATGGGTGTGCTGTATGTGCTTGATGAACCATCAATCGGTTTA 1560
Pste_S 1501 CAGATTGGTGCAGGGCTGATGGGTGTGCTGTATGTGCTTGATGAACCATCAATCGGTTTG 1560
Rma    1501 CAGATTGGTGCAGGACTGATGGGTGTGTTGTATGTGCTTGATGAACCATCAATCGGTTTA 1560
Ifos_S 1501 CAGATTGGTGCAGGATTGATGGGGGTGTTGTATGTGCTTGATGAACCATCAATCGGTCTG 1560
Apha_S 1501 CAGATTGGTGCAGGACTAATGGGCGTGCTGTATGTGCTTGATGAGCCATCAATTGGTTTG 1560
Bsep_S 1501 CAGATTGGTGCAGGACTGATGGGTGTGCTATATGTGTTAGATGAGCCCTCAATCGGCTTG 1560
Akaw_S  501   Q  I  G  A  G  L  M  G  V  L  Y  V  L  D  E  P  S  I  G  L  520
Clau_S  501   Q  I  G  V  G  L  M  G  V  L  Y  V  L  D  E  P  S  I  G  L  520
Pkil_S  501   Q  I  G  A  G  L  M  G  V  L  Y  V  L  D  E  P  S  I  G  L  520
Psoy_S  501   Q  I  G  A  G  L  M  G  V  L  Y  V  L  D  E  P  S  I  G  L  520
Vok     501   Q  I  G  A  G  L  M  G  V  L  Y  V  L  D  E  P  S  I  G  L  520
Cpac_S  501   Q  I  G  A  G  L  M  G  V  L  Y  V  L  D  E  P  S  I  G  L  520
Cfau_S  501   Q  I  G  A  G  L  M  G  V  L  Y  V  L  D  E  P  S  I  G  L  520
Cnau_S  501   Q  I  G  S  G  L  M  G  V  L  Y  V  L  D  E  P  S  I  G  L  520
Pste_S  501   Q  I  G  A  G  L  M  G  V  L  Y  V  L  D  E  P  S  I  G  L  520
Rma     501   Q  I  G  A  G  L  M  G  V  L  Y  V  L  D  E  P  S  I  G  L  520
Ifos_S  501   Q  I  G  A  G  L  M  G  V  L  Y  V  L  D  E  P  S  I  G  L  520
Apha_S  501   Q  I  G  A  G  L  M  G  V  L  Y  V  L  D  E  P  S  I  G  L  520
Bsep_S  501   Q  I  G  A  G  L  M  G  V  L  Y  V  L  D  E  P  S  I  G  L  520
```

ABC_UvrA_I domain (464-576)

```
Akaw_S 1561 CATCAAAGAGACAATCAAAAGCTATTAAACACACTTACCTATTTACGTGATATTGGCAAT 1620
Clau_S 1561 CATCAAAGAGACAATCAAAAGTTATTAAACACACTTGCCTATTTGCGTGATATTGGCAAT 1620
Pkil_S 1561 CATCAAAGAGACAATCAAAAGCTATTAAATACACTTGCCTATTTGCGTGATATTGGCAAT 1620
Psoy_S 1561 CATCAAAGAGACAATCAAAAGCTATTAAATACACTTGCCTATTTGCGTGATATTGGCAAT 1620
Vok    1561 CATCAAAGAGACAATCAAAAGCTATTAAACACACTTGCCTATTTGCGTGATATTGGAAAT 1620
Cpac_S 1561 CATCAAAGAGATAATCAAAAGTTACTAAATACACTGACCTATTTGCGTGATATTGGCAAT 1620
Cfau_S 1561 CATCAAAGAGATAATCAAAAGTTATTAAATACACTGATCTATTTACGTGATATTGGCAAT 1620
Cnau_S 1561 CATCAAAGAGATAATCAAAAGTTACTAAATACACTGACCTATTTGCGTGATATTGGCAAT 1620
Pste_S 1561 CATCAAAGAGATAATCAAAAATTACTAAATACACTGACCTATTTGCGTGATATTGGCAAC 1620
Rma    1561 CATCAAAGAGACAATCAAAAGTTACTAAATACACTGACATATTTGCGTGATATTGGTAAT 1620
Ifos_S 1561 CATCAAAGAGACAATCAAAAGTTACTAAATACACTGACTTATTTGCGTGATATTGGCAAT 1620
Apha_S 1561 CACCAAAGAGACAATCAAAAGTTATTAAATACACTGACCTATTTGCGTGATATTGGCAAT 1620
Bsep_S 1561 CACCAACGCGACAATCAAAAACTCTTAAACACTTTGATTTATCTCCGTGATATAGGCAAT 1620
Akaw_S  521   H  Q  R  D  N  Q  K  L  L  N  T  L  T  Y  L  R  D  I  G  N  540
Clau_S  521   H  Q  R  D  N  Q  K  L  L  N  T  L  A  Y  L  R  D  I  G  N  540
Pkil_S  521   H  Q  R  D  N  Q  K  L  L  N  T  L  A  Y  L  R  D  I  G  N  540
Psoy_S  521   H  Q  R  D  N  Q  K  L  L  N  T  L  A  Y  L  R  D  I  G  N  540
Vok     521   H  Q  R  D  N  Q  K  L  L  N  T  L  A  Y  L  R  D  I  G  N  540
Cpac_S  521   H  Q  R  D  N  Q  K  L  L  N  T  L  T  Y  L  R  D  I  G  N  540
Cfau_S  521   H  Q  R  D  N  Q  K  L  L  N  T  L  I  Y  L  R  D  I  G  N  540
Cnau_S  521   H  Q  R  D  N  Q  K  L  L  N  T  L  T  Y  L  R  D  I  G  N  540
Pste_S  521   H  Q  R  D  N  Q  K  L  L  N  T  L  T  Y  L  R  D  I  G  N  540
Rma     521   H  Q  R  D  N  Q  K  L  L  N  T  L  T  Y  L  R  D  I  G  N  540
Ifos_S  521   H  Q  R  D  N  Q  K  L  L  N  T  L  T  Y  L  R  D  I  G  N  540
Apha_S  521   H  Q  R  D  N  Q  K  L  L  N  T  L  T  Y  L  R  D  I  G  N  540
Bsep_S  521   H  Q  R  D  N  Q  K  L  L  N  T  L  I  Y  L  R  D  I  G  N  540
```

ABC_UvrA_I domain (464-576)

```
Akaw_S 1621 ACAGTGATTATGATAGAACACGATGAAGAAGCAATTAAACAGGCCGATTATGTAATTGAT 1680
Clau_S 1621 ACAGTGATTATGATAGAACACGATGAAGAAGCAATTAAACAGGCCGATTATGTAATTGAT 1680
Pkil_S 1621 ACAGTAATTATGATAGAACACGATGAAGAAGCAATTAAACAGGCCGATTATGTAATTGAT 1680
Psoy_S 1621 ACAGTAATTATGATAGAACACGATGAAGAAGCAATTAAACAGGCCGATTATGTAATTGAT 1680
Vok    1621 ACAGTGATTATGATAGAACACGATGAAGAAGCAATTAAACAGGCCGATTATGTAATTGAT 1680
Cpac_S 1621 ACAGTGGTTGTGGTAGAGCATGATGAAGAGACAATTAAACAGGCTGATTACGTGATTGAT 1680
Cfau_S 1621 ACAGTGATTGTGGTAGAGCATGATGAAGAGACAATTAAACAGGCTGATTATGTGATTGAT 1680
Cnau_S 1621 ACAGTGATTGTGGTAGAGCATGATGAAGAGACAATTAAACAGGCCGATTACGTGATTGAT 1680
Pste_S 1621 ACAGTGATTGTGGTAGAGCATGATGAAGAGACAATTAAACAGGCCGATTACGTGATTGAT 1680
Rma    1621 ACAGTGATTGTGGTAGAGCATGACAAAGAAGCAATTAAACAAGCCGATTACGTGATTGAT 1680
Ifos_S 1621 ACAGTAATTGTGGTAGAGCATGATGAGGAGGCAATTAAACAAGCTGATTACGTGATTGAT 1680
Apha_S 1621 ACAGTGATTGTGGTAGAGCACGACGAAGAGGCGATTAAACAAGCCGATTACGTGATTGAT 1680
Bsep_S 1621 ACTGTGATTGTCGTTGAGCACGATGAAGATGCTATTAAACAAGCCGATTTTGTTATCGAC 1680
Akaw_S  541   T  V  I  M  I  E  H  D  E  E  A  I  K  Q  A  D  Y  V  I  D  560
Clau_S  541   T  V  I  M  I  E  H  D  E  E  A  I  K  Q  A  D  Y  V  I  D  560
Pkil_S  541   T  V  I  M  I  E  H  D  E  E  A  I  K  Q  A  D  Y  V  I  D  560
Psoy_S  541   T  V  I  M  I  E  H  D  E  E  A  I  K  Q  A  D  Y  V  I  D  560
Vok     541   T  V  I  M  I  E  H  D  E  E  A  I  K  Q  A  D  Y  V  I  D  560
Cpac_S  541   T  V  V  V  V  E  H  D  E  E  T  I  K  Q  A  D  Y  V  I  D  560
Cfau_S  541   T  V  I  V  V  E  H  D  E  E  T  I  K  Q  A  D  Y  V  I  D  560
Cnau_S  541   T  V  I  V  V  E  H  D  E  E  T  I  K  Q  A  D  Y  V  I  D  560
Pste_S  541   T  V  I  V  V  E  H  D  E  E  T  I  K  Q  A  D  Y  V  I  D  560
Rma     541   T  V  I  V  V  E  H  D  K  E  A  I  K  Q  A  D  Y  V  I  D  560
Ifos_S  541   T  V  I  V  V  E  H  D  E  E  A  I  K  Q  A  D  Y  V  I  D  560
Apha_S  541   T  V  I  V  V  E  H  D  E  E  A  I  K  Q  A  D  Y  V  I  D  560
Bsep_S  541   T  V  I  V  V  E  H  D  E  D  A  I  K  Q  A  D  F  V  I  D  560
```

ABC_UvrA_I domain (464-576)

```
Akaw_S 1681 ATTGGTCCTGGTGCTGGTGTACATGGAGGTGAAATAGTTGCCATTGGTATACCTGAAGAT 1740
Clau_S 1681 ATTGGTCCTGGTGCTGGTGTACATGGAGGTGAAATAGTTGCTATTGGCATACCTGAAGAT 1740
Pkil_S 1681 ATTGGTCCTGGTGCTGGCGTACATGGAGGTGAAATAGTTGCCATTGGCATACCTAAAGAT 1740
Psoy_S 1681 ATTGGTCCTGGTGCTGGCGTACATGGAGGTGAAATAGTTGCCATTGGCATACCTAAAGAT 1740
Vok    1681 ATTGGGCCTGGTGCTGGTGTACATGGAGGTGAAATAGTTGCTATTGGTATACCTAAAGAT 1740
Cpac_S 1681 ATCGGTCCTGGTGCTGGTATTCATGGCGGTGAAATTGTTGCAATGGGCAGCCCTAAAGAC 1740
Cfau_S 1681 ATCGGTCCTGGTGCTGGTATTCATGGCGGTGAAATTGTTGCAATGGGCAGCCCTAAAGAC 1740
Cnau_S 1681 ATCGGTCCTGGTGCTGGTATTCATGGCGGTGAAATTGTTGCAATGGGCAGCCCTAAAGAC 1740
Pste_S 1681 ATCGGTCCTGGTGCTGGTATTCATGGCGGTGAAATTGTTGCAATGGGCAGCCCTAAAGAC 1740
Rma    1681 ATCGGTCCTGGTGCTGGTATTCATGGCGGTGAAATTGTTGCAATGGGTAGCCCTAAAGAC 1740
Ifos_S 1681 ATCGGTCCTGGCGCTGGTATTCATGGTGGTGAAATTGTTGCAATGGGCAGTCCTAAAGAC 1740
Apha_S 1681 ATTGGCCCTGGTGCTGGTATTCATGGCGGTGAAATTGTTGCAATGGGTAGCCCTAAAGAC 1740
Bsep_S 1681 ATTGGACCAGGCGCAGGCATTCATGGGGGTGAAATTATTGCCACTGGCACCCCGCAAGAG 1740
Akaw_S  561   I  G  P  G  A  G  V  H  G  G  E  I  V  A  I  G  I  P  E  D  580
Clau_S  561   I  G  P  G  A  G  V  H  G  G  E  I  V  A  I  G  I  P  E  D  580
Pkil_S  561   I  G  P  G  A  G  V  H  G  G  E  I  V  A  I  G  I  P  K  D  580
Psoy_S  561   I  G  P  G  A  G  V  H  G  G  E  I  V  A  I  G  I  P  K  D  580
Vok     561   I  G  P  G  A  G  V  H  G  G  E  I  V  A  I  G  I  P  K  D  580
Cpac_S  561   I  G  P  G  A  G  I  H  G  G  E  I  V  A  M  G  S  P  K  D  580
Cfau_S  561   I  G  P  G  A  G  I  H  G  G  E  I  V  A  M  G  S  P  K  D  580
Cnau_S  561   I  G  P  G  A  G  I  H  G  G  E  I  V  A  M  G  S  P  K  D  580
Pste_S  561   I  G  P  G  A  G  I  H  G  G  E  I  V  A  M  G  S  P  K  D  580
Rma     561   I  G  P  G  A  G  I  H  G  G  E  I  V  A  M  G  S  P  K  D  580
Ifos_S  561   I  G  P  G  A  G  I  H  G  G  E  I  V  A  M  G  S  P  K  D  580
Apha_S  561   I  G  P  G  A  G  I  H  G  G  E  I  V  A  M  G  S  P  K  D  580
Bsep_S  561   I  G  P  G  A  G  I  H  G  G  E  I  I  A  T  G  T  P  Q  E  580
```

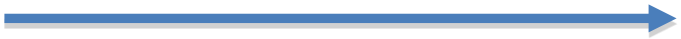

ABC_UvrA_I domain (464-576)

```
Akaw_S 1741 ATTGAAAATAACCTTAAATCTCTAACAGGTGATTATCTAAGCGGTCGTCAAAGTATTAAA 1800
Clau_S 1741 ATTAAAAACAACCTTAAGTCTCTAACAGGTGATTATCTAAGTGGTCGTCAAAGTATTAAA 1800
Pkil_S 1741 ATTGAAAACAATCTTAAGTCTTTAACAGGTGATTATCTAAGTGGTCGTCAAAGTATTAAA 1800
Psoy_S 1741 ATTGAAAACAATCTTAAGTCTTTAACAGGTGATTATCTAAGTGGTCGTCAAAGTATTAAA 1800
Vok    1741 ATTGAAAATAATCTTAAGTCTTTAACAGGTGATTATCTAAGTGGTCGTCAAAGTATTAAA 1800
Cpac_S 1741 ATTGAAAACAATCTTAAATCTTTAACGGGTGATTATTTAAGTGGTCGCCAAGGTATTGAA 1800
Cfau_S 1741 ATTGAAAACAACCTTAAGTCTTTAACGGGTGATTATTTAAGTGGTCGTCAAAGTATTGAA 1800
Cnau_S 1741 ATTGAAAATAATCTTAAGTCTTTAACGGGTGATTATTTAAGCGGTCGCCAAAGTATTGAA 1800
Pste_S 1741 ATTGAAAACAACCTTAAGTCTTTAACGGGTGATTATTTAAGTGGTCGCCAAAGTATTGAA 1800
Rma    1741 ATCGAAAATAACCTTAAATCTTTAACGGGTGATTATTTAAGTGGTCGTCAAAGTATTGAA 1800
Ifos_S 1741 ATTGAAAACAACCTTAAGTCTTTAACGGGTGATTATTTAAGTGGTCGTCAAAGTATTGAA 1800
Apha_S 1741 ATTGAAAACAACCTTAAGTCTTTAACGGGTGATTATTTAAGTGGCCGTCAAAGTATTGAA 1800
Bsep_S 1741 ATCAGTGAAAATCCAAAATCTATCACTGGTGACTACATTAGCGGTCGTCAAAGCATTGCC 1800
Akaw_S  581   I  E  N  N  L  K  S  L  T  G  D  Y  L  S  G  R  Q  S  I  K  600
Clau_S  581   I  K  N  N  L  K  S  L  T  G  D  Y  L  S  G  R  Q  S  I  K  600
Pkil_S  581   I  E  N  N  L  K  S  L  T  G  D  Y  L  S  G  R  Q  S  I  K  600
Psoy_S  581   I  E  N  N  L  K  S  L  T  G  D  Y  L  S  G  R  Q  S  I  K  600
Vok     581   I  E  N  N  L  K  S  L  T  G  D  Y  L  S  G  R  Q  S  I  K  600
Cpac_S  581   I  E  N  N  L  K  S  L  T  G  D  Y  L  S  G  R  Q  G  I  E  600
Cfau_S  581   I  E  N  N  L  K  S  L  T  G  D  Y  L  S  G  R  Q  S  I  E  600
Cnau_S  581   I  E  N  N  L  K  S  L  T  G  D  Y  L  S  G  R  Q  S  I  E  600
Pste_S  581   I  E  N  N  L  K  S  L  T  G  D  Y  L  S  G  R  Q  S  I  E  600
Rma     581   I  E  N  N  L  K  S  L  T  G  D  Y  L  S  G  R  Q  S  I  E  600
Ifos_S  581   I  E  N  N  L  K  S  L  T  G  D  Y  L  S  G  R  Q  S  I  E  600
Apha_S  581   I  E  N  N  L  K  S  L  T  G  D  Y  L  S  G  R  Q  S  I  E  600
Bsep_S  581   I  S  E  N  P  K  S  I  T  G  D  Y  I  S  G  R  Q  S  I  A  600
```

```
Akaw_S 1801 GTTCCTACTAAACGAAAAAGTGCTAATCAATGGATTCGTATTAAAGGAGCTAGAGGTAAT 1860
Clau_S 1801 GTTCCTACTAAACGAAAAAGTGCCAATCAATGGATTCGTATTAAAGGAGCTAAGGGTAAT 1860
Pkil_S 1801 GTTCCTACTAAACGAAAAAGTGCCAATCAATGGATTCGTATTAAAGGCGCTAAGGGTAAT 1860
Psoy_S 1801 GTTCCTACTAAACGAAAAAGTGCCAATCAATGGATTCGTATTAAAGGCGCTAAGGGTAAT 1860
Vok    1801 GTTCCTACTAAACGAAAAAGTGCCAATCAATGGATTCGTATTAAAGGCGCTAAGGGTAAT 1860
Cpac_S 1801 GTGCCTGCTAAACGTAAAGGTGCTAGTCAGTGGTTGTGTATTAAAGGTGCTAAGGGTAAC 1860
Cfau_S 1801 GTGCCTGCTAAACGTAAAAGTGCCAATCAGTGGTTGTGTATTAAAGGTGCTAAAGGTAAT 1860
Cnau_S 1801 GTACCTTCCAAACGTAAAAGTGCCAGTCAGTGGTTGTGTATTAAAGGTGCTAAGGGTAAC 1860
Pste_S 1801 GTGCCTGTTAAACGTAAAAGTGCCAGTCAGTGGTTGTGTATTAAAGGTGCTAAGGGCAAC 1860
Rma    1801 GTACCCACTAAACGTAAAAATGTCAGTCAGTGGTTGCGTATTAAAGGCGCTAAGGGTAAT 1860
Ifos_S 1801 GTGCCTGCTAAACGTAAAAGTGCCAGTCAGTGGTTGCGCATTAAAGGCGCTAGGGGTAAT 1860
Apha_S 1801 GTGCCTGCTAAACGTAAAAGTGCCAGTCAGTGGTTGCACATTAAAGGTGCTAGGGGTAAT 1860
Bsep_S 1801 GTGCCAAAACAACGCAAAACTGCAACCGATTGGCTACACATCAAAGGTGCAACGGGTAAC 1860
Akaw_S  601   V  P  T  K  R  K  S  A  N  Q  W  I  R  I  K  G  A  R  G  N  620
Clau_S  601   V  P  T  K  R  K  S  A  N  Q  W  I  R  I  K  G  A  K  G  N  620
Pkil_S  601   V  P  T  K  R  K  S  A  N  Q  W  I  R  I  K  G  A  K  G  N  620
Psoy_S  601   V  P  T  K  R  K  S  A  N  Q  W  I  R  I  K  G  A  K  G  N  620
Vok     601   V  P  T  K  R  K  S  A  N  Q  W  I  R  I  K  G  A  K  G  N  620
Cpac_S  601   V  P  A  K  R  K  G  A  S  Q  W  L  C  I  K  G  A  K  G  N  620
Cfau_S  601   V  P  A  K  R  K  S  A  N  Q  W  L  C  I  K  G  A  K  G  N  620
Cnau_S  601   V  P  S  K  R  K  S  A  S  Q  W  L  C  I  K  G  A  K  G  N  620
Pste_S  601   V  P  V  K  R  K  S  A  S  Q  W  L  C  I  K  G  A  K  G  N  620
Rma     601   V  P  T  K  R  K  N  V  S  Q  W  L  R  I  K  G  A  K  G  N  620
Ifos_S  601   V  P  A  K  R  K  S  A  S  Q  W  L  R  I  K  G  A  R  G  N  620
Apha_S  601   V  P  A  K  R  K  S  A  S  Q  W  L  H  I  K  G  A  R  G  N  620
Bsep_S  601   V  P  K  Q  R  K  T  A  T  D  W  L  H  I  K  G  A  T  G  N  620
```

ABC_UvrA_II domain (612-919)

```
Akaw_S 1861 AGCCTTAATAATGTTGATTTGTCTATTCCTATAGGTGTATTAACTTGTGTGACAGGTGTA 1920
Clau_S 1861 AGCCTTAATAATGTTGATTTGTCTATTCCTATAGGTGTATTAACTTGTGTGACAGGTGTA 1920
Pkil_S 1861 AGCCTTAATAATGTTGATTTGTCTATCCCTATAGGTGTATTAACTTGTGTGACAGGCGTG 1920
Psoy_S 1861 AGCCTTAATAATGTTGATTTGTCTATCCCTATAGGTGTATTAACTTGTGTGACAGGCGTG 1920
Vok    1861 AGCCTTAATAATGTTGATTTGTCTATTCCTATAGGTGTGTTAACTTGTGTGACAGGCGTA 1920
Cpac_S 1861 AATCTTAATAAAGTTGACTTGTCTATCCCTGTAGGTGTATTAACTTGTGTGACAGGTGTG 1920
Cfau_S 1861 AATCTTAATAAGGTTGACTTGTCTATCCCTGTAGGCGTATTAACTTGTGTGACGGGTGTG 1920
Cnau_S 1861 AATCTTAACAAGGTTGACTTGTCTATCCCTGTGGGCGTATTAACTTGTGTGACAGGCGTA 1920
Pste_S 1861 AATCTTAATAAGGTTGACTTGTCTATCCCTGTGGGTGTATTAACTTGTGTGACAGGTGTG 1920
Rma    1861 AATCTTAATAAGGTTGACCTGTCTATCCCTGTGGGTGTATTAACTTGTGTGACGGGTGTG 1920
Ifos_S 1861 AATCTTAACAAGGTTGACTTGTCTATCCCTGTGGGTGTATTGACTTGTGTGACAGGTGTA 1920
Apha_S 1861 AATCTTAACAAGGTTGACTTATCTATCCCTGTGGGTGTATTAACTTGTGTGACAGGTGTA 1920
Bsep_S 1861 AACCTTAATAAAGTAGACTTAGCTATCCCCATCGGTGTCCTGACTTGCGTTACAGGTGTT 1920
Akaw_S  621   S  L  N  N  V  D  L  S  I  P  I  G  V  L  T  C  V  T  G  V  640
Clau_S  621   S  L  N  N  V  D  L  S  I  P  I  G  V  L  T  C  V  T  G  V  640
Pkil_S  621   S  L  N  N  V  D  L  S  I  P  I  G  V  L  T  C  V  T  G  V  640
Psoy_S  621   S  L  N  N  V  D  L  S  I  P  I  G  V  L  T  C  V  T  G  V  640
Vok     621   S  L  N  N  V  D  L  S  I  P  I  G  V  L  T  C  V  T  G  V  640
Cpac_S  621   N  L  N  K  V  D  L  S  I  P  V  G  V  L  T  C  V  T  G  V  640
Cfau_S  621   N  L  N  K  V  D  L  S  I  P  V  G  V  L  T  C  V  T  G  V  640
Cnau_S  621   N  L  N  K  V  D  L  S  I  P  V  G  V  L  T  C  V  T  G  V  640
Pste_S  621   N  L  N  K  V  D  L  S  I  P  V  G  V  L  T  C  V  T  G  V  640
Rma     621   N  L  N  K  V  D  L  S  I  P  V  G  V  L  T  C  V  T  G  V  640
Ifos_S  621   N  L  N  K  V  D  L  S  I  P  V  G  V  L  T  C  V  T  G  V  640
Apha_S  621   N  L  N  K  V  D  L  S  I  P  V  G  V  L  T  C  V  T  G  V  640
Bsep_S  621   N  L  N  K  V  D  L  A  I  P  I  G  V  L  T  C  V  T  G  V  640

E. coli                                                       G  V

                                                    ATPase site II
```

ABC_UvrA_II domain (612-919)

```
Akaw_S 1921 TCTGGTTCTGGAAAATCAACCTTAATTAATGATACTTTGTATGCTTTGGCAGCAAGGGAG 1980
Clau_S 1921 TCTGGATCTGGAAAATCAACTTTAATTAATGATACTTTGTATGCTTTGGCAGCAAGAGAG 1980
Pkil_S 1921 TCTGGTTCTGGAAAATCAACCTTAATTAATGATACTTTGTATGCTTTGGCAGCAAGGGAA 1980
Psoy_S 1921 TCTGGTTCTGGAAAATCAACCTTAATTAATGATACTTTGTATGCTTTGGCAGCAAGGGAA 1980
Vok    1921 TCTGGTTCTGGAAAATCAACCTTAATTAATGATACTTTGTATGCTTTGGCAGCAAGGGAA 1980
Cpac_S 1921 TCTGGTTCTGGAAAATCAACCCTAATTAACGACACTTTGTATGCTTTAGCGGCTAGAGAA 1980
Cfau_S 1921 TCTGGTTCTGGGAAATCAACCTTAATTAACGACACTTTGTATGCTTTAGCGGCTAGAGAA 1980
Cnau_S 1921 TCTGGTTCTGGAAAATCAACCTTAATTAACGACACTTTGTATGCTTTAGCGGCTAGAGAA 1980
Pste_S 1921 TCTGGTTCTGGAAAATCAACCTTAATTAACAACACTTTGTATGCTTTAGCGGCTAGAGAA 1980
Rma    1921 TCTGGCTCTGGAAAATCAACCTTAATTAACGATACTTTGTATGCTTTAGTAGCTAGAGAG 1980
Ifos_S 1921 TCTGGCTCTGGAAAGTCAACTTTAATTAACGACACTTTATATGCTTTAGCAGCCAGGGAG 1980
Apha_S 1921 TCTGGCTCTGGAAAATCAACCTTAATTAACGACACCTTGTATGCTTTAGCGGCTAGAGAA 1980
Bsep_S 1921 TCGGGCTCGGGAAAATCCACTCTGATTAACGACACCCTGTATTCGCTCGCTGCTAGAGGT 1980
Akaw_S  641  S  G  S  G  K  S  T  L  I  N  D  T  L  Y  A  L  A  A  R  E  660
Clau_S  641  S  G  S  G  K  S  T  L  I  N  D  T  L  Y  A  L  A  A  R  E  660
Pkil_S  641  S  G  S  G  K  S  T  L  I  N  D  T  L  Y  A  L  A  A  R  E  660
Psoy_S  641  S  G  S  G  K  S  T  L  I  N  D  T  L  Y  A  L  A  A  R  E  660
Vok     641  S  G  S  G  K  S  T  L  I  N  D  T  L  Y  A  L  A  A  R  E  660
Cpac_S  641  S  G  S  G  K  S  T  L  I  N  D  T  L  Y  A  L  A  A  R  E  660
Cfau_S  641  S  G  S  G  K  S  T  L  I  N  D  T  L  Y  A  L  A  A  R  E  660
Cnau_S  641  S  G  S  G  K  S  T  L  I  N  D  T  L  Y  A  L  A  A  R  E  660
Pste_S  641  S  G  S  G  K  S  T  L  I  N  N  T  L  Y  A  L  A  A  R  E  660
Rma     641  S  G  S  G  K  S  T  L  I  N  D  T  L  Y  A  L  V  A  R  E  660
Ifos_S  641  S  G  S  G  K  S  T  L  I  N  D  T  L  Y  A  L  A  A  R  E  660
Apha_S  641  S  G  S  G  K  S  T  L  I  N  D  T  L  Y  A  L  A  A  R  E  660
Bsep_S  641  S  G  S  G  K  S  T  L  I  N  D  T  L  Y  S  L  A  A  R  G  660

E. coli      S  G  S  G  K
```

ATPase site II

ABC_UvrA_II domain (612-919)

```
Akaw_S 1981 CTTCATCATGCACAAATAACACCTGCTGAATATGAATCAGTTGAAGGATTAAATTATCTT 2040
Clau_S 1981 CTTCATCATACACAAATAATACCTGCTGAATATGAATCAGTTGAAGGGTTGAATTATCTT 2040
Pkil_S 1981 CTTCATCATGCACAAATAACACCTGCTGAATATGAATCAGTTGAAGGATTGAATTATCTT 2040
Psoy_S 1981 CTTCATCATGCACAAATAACACCTGCTGAATATGAATCAGTTGAAGGATTGAATTATCTT 2040
Vok    1981 CTTCATCATGCACAAATAACACCTGCTGAATATGAATCAGTCGAAGGATTGAATTATCTT 2040
Cpac_S 1981 GTTAATCGTGCACAGACAACGCCTGCTGAGTATGAATCAGTTGAAGGTTTAAATTATTTT 2040
Cfau_S 1981 GTTAATCGTGCACAGACAACGCCTGCTGAGTATGAATCAGTTGAAGGTTTAAATTATTTT 2040
Cnau_S 1981 CTTAATCGTGCACAGACAACGCCTGCTGAGTATGAATCAGTTGAAGGTTTAAATTATTTT 2040
Pste_S 1981 CTTAATCGTGCACAGACAACGCCTGCTGAGTATGAATCAGTTGAAGGTTTAAATTATTTT 2040
Rma    1981 CTTAATCATGCACAAACAACACCTGCTGAATATGAATCAATTGAAGGTTTGAATTATTTT 2040
Ifos_S 1981 CTTAATCGCGCACAAACAACGCCTGCTGAATATGAGTCAGTTGAAGGTTTGGATTATTTT 2040
Apha_S 1981 CTTAATCGTGCACAGACAACGCCTGCTGAGTATGAATCAGTTGAAGGTTTGGATTATTTT 2040
Bsep_S 1981 TTAAATCGTTCGCAAATCGTTCCCGCGCCACATGAATCCATTGAGGGTTTAGAATATTGC 2040
Akaw_S  661  L  H  H  A  Q  I  T  P  A  E  Y  E  S  V  E  G  L  N  Y  L  680
Clau_S  661  L  H  H  T  Q  I  I  P  A  E  Y  E  S  V  E  G  L  N  Y  L  680
Pkil_S  661  L  H  H  A  Q  I  T  P  A  E  Y  E  S  V  E  G  L  N  Y  L  680
Psoy_S  661  L  H  H  A  Q  I  T  P  A  E  Y  E  S  V  E  G  L  N  Y  L  680
Vok     661  L  H  H  A  Q  I  T  P  A  E  Y  E  S  V  E  G  L  N  Y  L  680
Cpac_S  661  V  N  R  A  Q  T  T  P  A  E  Y  E  S  V  E  G  L  N  Y  F  680
Cfau_S  661  V  N  R  A  Q  T  T  P  A  E  Y  E  S  V  E  G  L  N  Y  F  680
Cnau_S  661  L  N  R  A  Q  T  T  P  A  E  Y  E  S  V  E  G  L  N  Y  F  680
Pste_S  661  L  N  R  A  Q  T  T  P  A  E  Y  E  S  V  E  G  L  N  Y  F  680
Rma     661  L  N  H  A  Q  T  T  P  A  E  Y  E  S  I  E  G  L  N  Y  F  680
Ifos_S  661  L  N  R  A  Q  T  T  P  A  E  Y  E  S  V  E  G  L  D  Y  F  680
Apha_S  661  L  N  R  A  Q  T  T  P  A  E  Y  E  S  V  E  G  L  D  Y  F  680
Bsep_S  661  L  N  R  S  Q  I  V  P  A  P  H  E  S  I  E  G  L  E  Y  C  680
```

ABC_UvrA_II domain (612-919)

```
Akaw_S 2041 GATAAAATTGTTAATATTGATCAAAGACCAATTGGTCGTACGCCACGTTCTAATCCAGCT 2100
Clau_S 2041 GATAAAATTGTTAATATTAATCAAAGTCCAATTGGACGTACGCCACGTTCTAATCCAGCT 2100
Pkil_S 2041 GATAAAATTGTTAATATTGATCAAAGTCCAATTGGACGTACGCCACGTTCTAATCCAGCT 2100
Psoy_S 2041 GATAAAATTGTTAATATTGATCAAAGTCCAATTGGACGTACGCCACGTTCTAATCCAGCT 2100
Vok    2041 GATAAAATTGTTAATATTGATCAAAGTCCAATTGGACGTACACCACGTTCTAATCCAGCT 2100
Cpac_S 2041 GATAAGATTGTCAATATTGACCAAAGCCCAATTGGACGTACGCCACGCTCTAATCCAGCC 2100
Cfau_S 2041 GATAAGATTGTCAATATTGACCAAAGCCCAATTGGACGTACGCCACGTTCTAATCCAGCA 2100
Cnau_S 2041 GATAAGATTGTCAATATTGACCAAAGTCCAATTGGACGTACGCCACGTTCTAATCCAGCC 2100
Pste_S 2041 GATAAGATTGTCAATATTGACCAAAGCCCAATTGGACGTACGCCACGCTCTAATCCAGCC 2100
Rma    2041 GACAAGATTGTCAATATTGATCAAAGCCCAATTGGACGCACACCACGCTCTAATCCAGCT 2100
Ifos_S 2041 GACAAAATTGTCAATATTGACCAAAGCCCAATTGGACGCACACCACGCTCTAATCCAGCC 2100
Apha_S 2041 GATAAGATTGTCAATATTGACCAAAGCCCAATTGGACGTACGCCACGCTCTAATCCAGCC 2100
Bsep_S 2041 GACAAAGTTGTCAACATTGACCAAAGCCCTATTGGTCGCACCCCTCGTTCAAACCCAGCC 2100
Akaw_S  681   D  K  I  V  N  I  D  Q  R  P  I  G  R  T  P  R  S  N  P  A   700
Clau_S  681   D  K  I  V  N  I  N  Q  S  P  I  G  R  T  P  R  S  N  P  A   700
Pkil_S  681   D  K  I  V  N  I  D  Q  S  P  I  G  R  T  P  R  S  N  P  A   700
Psoy_S  681   D  K  I  V  N  I  D  Q  S  P  I  G  R  T  P  R  S  N  P  A   700
Vok     681   D  K  I  V  N  I  D  Q  S  P  I  G  R  T  P  R  S  N  P  A   700
Cpac_S  681   D  K  I  V  N  I  D  Q  S  P  I  G  R  T  P  R  S  N  P  A   700
Cfau_S  681   D  K  I  V  N  I  D  Q  S  P  I  G  R  T  P  R  S  N  P  A   700
Cnau_S  681   D  K  I  V  N  I  D  Q  S  P  I  G  R  T  P  R  S  N  P  A   700
Pste_S  681   D  K  I  V  N  I  D  Q  S  P  I  G  R  T  P  R  S  N  P  A   700
Rma     681   D  K  I  V  N  I  D  Q  S  P  I  G  R  T  P  R  S  N  P  A   700
Ifos_S  681   D  K  I  V  N  I  D  Q  S  P  I  G  R  T  P  R  S  N  P  A   700
Apha_S  681   D  K  I  V  N  I  D  Q  S  P  I  G  R  T  P  R  S  N  P  A   700
Bsep_S  681   D  K  V  V  N  I  D  Q  S  P  I  G  R  T  P  R  S  N  P  A   700
```

ABC_UvrA_II domain (612-919)

```
Akaw_S 2101 ACTTATACAGGTGTATTTTCGTTAGTACGTGATTTATTTTCACAAACTTTAGAGGCAAGG 2160
Clau_S 2101 ACTTATACAGGTGTATTTTCGTTAGTACGTGATTTATTTTCACAAACTTTAGAGGCAAGG 2160
Pkil_S 2101 ACTTATACAGGTGTATTTTCGTTAGTGCGTGATTTATTTTCACAAACTTTAGAGGCAAAG 2160
Psoy_S 2101 ACTTATACAGGTGTATTTTCGTTAGTGCGTGATTTATTTTCACAAACTTTAGAGGCAAAG 2160
Vok    2101 ACTTATACAGGTGTATTTTCGTTAGTGCGTGATTTATTTTCACAAACTTTAGAGGCAAAG 2160
Cpac_S 2101 ACTTATACGGGTGTGTTTTCGTTAGTACGTGATTTATTTTCACAAACTTTAGAGGCAAGG 2160
Cfau_S 2101 ACTTATACGGGCGTGTTTTCGTTAGTACGTGATTTATTTTCACAAACTTTAGAGGCAAGA 2160
Cnau_S 2101 ACTTATACGGGTGTGTTTTCATTAGTACGTGATTTATTTTCACAAACTTTAGAGGCAAGA 2160
Pste_S 2101 ACTTATACGGGTGTGTTTTCATTAGTACGTGATTTATTTTCACAAACTTTAGAGGCAAGA 2160
Rma    2101 ACTTATACAGGTGTATTTTCATTAGTACGTGATTTATTTTCACAAACTTTGGAAGCAAGA 2160
Ifos_S 2101 ACTTATACAGGCGTGTTCTCATTAATTCGTGATTTATTTTCACAAACTTTAGAAGCAAGA 2160
Apha_S 2101 ACTTATACGGGTGTGTTTTTATTAGTACGTGATTTATTTTCACAAACTTTAGAGGCAAGA 2160
Bsep_S 2101 ACTTATACGAGCGTGTTTACTCTTATTCGTGATTTATTTTCACAAACTTTAGAAGCCCGT 2160
Akaw_S  701   T  Y  T  G  V  F  S  L  V  R  D  L  F  S  Q  T  L  E  A  R   720
Clau_S  701   T  Y  T  G  V  F  S  L  V  R  D  L  F  S  Q  T  L  E  A  R   720
Pkil_S  701   T  Y  T  G  V  F  S  L  V  R  D  L  F  S  Q  T  L  E  A  K   720
Psoy_S  701   T  Y  T  G  V  F  S  L  V  R  D  L  F  S  Q  T  L  E  A  K   720
Vok     701   T  Y  T  G  V  F  S  L  V  R  D  L  F  S  Q  T  L  E  A  K   720
Cpac_S  701   T  Y  T  G  V  F  S  L  V  R  D  L  F  S  Q  T  L  E  A  R   720
Cfau_S  701   T  Y  T  G  V  F  S  L  V  R  D  L  F  S  Q  T  L  E  A  R   720
Cnau_S  701   T  Y  T  G  V  F  S  L  V  R  D  L  F  S  Q  T  L  E  A  R   720
Pste_S  701   T  Y  T  G  V  F  S  L  V  R  D  L  F  S  Q  T  L  E  A  R   720
Rma     701   T  Y  T  G  V  F  S  L  V  R  D  L  F  S  Q  T  L  E  A  R   720
Ifos_S  701   T  Y  T  G  V  F  S  L  I  R  D  L  F  S  Q  T  L  E  A  R   720
Apha_S  701   T  Y  T  G  V  F  L  L  V  R  D  L  F  S  Q  T  L  E  A  R   720
Bsep_S  701   T  Y  T  S  V  F  T  L  I  R  D  L  F  S  Q  T  L  E  A  R   720
```

ABC_UvrA_II domain (612-919)

```
Akaw_S 2161 TTACGTGGTTATAAAGCAGGTTGTTTTAGCTTTAATGTTAAAGGAGGTAGGTGTGAAGCG 2220
Clau_S 2161 TTACGTGGTTATAAAGCAGGTCGTTTTAGCTTTAATGTTAAAGGAGGTAGGTGTGAAGCA 2220
Pkil_S 2161 TTACGTGGTTATAAAGCAGGTCGTTTTAGTTTTAATGTTAAAGGAGGTAGGTGTGAAGCG 2220
Psoy_S 2161 TTACGTGGTTATAAAGCAGGTCGTTTTAGTTTTAATGTTAAAGGAGGTAGGTGTGAAGCG 2220
Vok    2161 TTACGTGGTTATAAAGCAGGTCGTTTTAGTTTTAATGTTAAAGGAGGTAGGTGTGAAGCG 2220
Cpac_S 2161 ATGCGCGGTTATAAAGCAGGACGTTTTAGTTTTAATGTTAAAGGCGGTAGATGCGAAGCA 2220
Cfau_S 2161 ACGCGCGGTTATAAAGCAGGGCGTTTTAGTTTTAATGTTAAAGGTGGTAGATGTGAAGCA 2220
Cnau_S 2161 ACGCGCGGTTATAAAGTAGGGCGTTTTAGTTTTAATGTTAAAGGTGGTAGATGTGAAGTA 2220
Pste_S 2161 ACGCGCGGTTATAAAGCGGGGCGTTTTAGTTTTAATGTTAAAGGCGGTAGATGTGAAGCA 2220
Rma    2161 ACGCGCGGTTATAAAGCAGGACGTTTTAGTTTTAATGTTAAAGGTGGTAGATGTGAGGCA 2220
Ifos_S 2161 ACATGTGGTTATAAAGCAGGGCGTTTTAGTTTTAATGTTAAAGGCGGTAGATGTGAAGCG 2220
Apha_S 2161 ACACGTGGTTATAAAGCAGGGCGTTTTAGTTTTAATGTTAAAGGTGGTAGATGTGAAGCA 2220
Bsep_S 2161 TCTCGTGGCTATAAAGCAGGGCGTTTCAGTTTTAATGTTAAAGGTGGCCGCTGCGAGGCG 2220
Akaw_S  721   L  R  G  Y  K  A  G  C  F  S  F  N  V  K  G  G  R  C  E  A   740
Clau_S  721   L  R  G  Y  K  A  G  R  F  S  F  N  V  K  G  G  R  C  E  A   740
Pkil_S  721   L  R  G  Y  K  A  G  R  F  S  F  N  V  K  G  G  R  C  E  A   740
Psoy_S  721   L  R  G  Y  K  A  G  R  F  S  F  N  V  K  G  G  R  C  E  A   740
Vok     721   L  R  G  Y  K  A  G  R  F  S  F  N  V  K  G  G  R  C  E  A   740
Cpac_S  721   M  R  G  Y  K  A  G  R  F  S  F  N  V  K  G  G  R  C  E  A   740
Cfau_S  721   T  R  G  Y  K  A  G  R  F  S  F  N  V  K  G  G  R  C  E  A   740
Cnau_S  721   T  R  G  Y  K  V  G  R  F  S  F  N  V  K  G  G  R  C  E  V   740
Pste_S  721   T  R  G  Y  K  A  G  R  F  S  F  N  V  K  G  G  R  C  E  A   740
Rma     721   T  R  G  Y  K  A  G  R  F  S  F  N  V  K  G  G  R  C  E  A   740
Ifos_S  721   T  C  G  Y  K  A  G  R  F  S  F  N  V  K  G  G  R  C  E  A   740
Apha_S  721   T  R  G  Y  K  A  G  R  F  S  F  N  V  K  G  G  R  C  E  A   740
Bsep_S  721   S  R  G  Y  K  A  G  R  F  S  F  N  V  K  G  G  R  C  E  A   740
```

ABC_UvrA_II domain (612-919)

```
Akaw_S 2221 TGCAAGGGTGATGGATTGGTTAAGGTAGAAATGCATTTTCTGCCAGATATTTATGTATCA 2280
Clau_S 2221 TGCAAGGGTGATGGATTGATTAAGGTAGAAATGCATTTTCTGCCAGATATTTATGTATCA 2280
Pkil_S 2221 TGCAAGGGTGATGGATTGATTAAGGTAGAAATGCATTTCCTGCCAGATATTTATGTATCA 2280
Psoy_S 2221 TGCAAGGGTGATGGATTGATTAAGGTAGAAATGCATTTCCTGCCAGATATTTATGTATCA 2280
Vok    2221 TGCAAGGGTGATGGATTGATTAAGGTAGAAATGCATTTCCTATCAGATATTTATGTATCA 2280
Cpac_S 2221 TGCAAGGGTGACGGGCTGATTAAGATGGAAATGCATTTTTTACCAGATGTTTATGTGTCA 2280
Cfau_S 2221 TGCAAGGGTGACGGGCTGATTAAGACGGAAATGCATTTTTTGCCAGATGTTTATGTGTCA 2280
Cnau_S 2221 TGCAAGGGTGACGGGCTGATTAAGGTAGAAATGCATTTTTTGCCAGATGTTTATGTGTCA 2280
Pste_S 2221 TGCAAGGGTGACGGGCTGATTAAGGTGGAAATGCATTTTTTGCCAGATGTTTATGTGTCA 2280
Rma    2221 TGTAAGGGTGATGGACTGATTAAGGTAGAAATGCATTTTTTACCAGATGTTTATGTGTTA 2280
Ifos_S 2221 TGCAAGGGCGATGGGCTAATCAAGGTAGAAATGCATTTTTTACCAGATGTTTATGTGTCA 2280
Apha_S 2221 TGCAAGGGTGATGGGCTAATTAAGGTAGAAATGCATTTTTTGCCAGATGTTTATGTGTCA 2280
Bsep_S 2221 TGTAAAGGCGATGGCTTGATTAAAGTTGAAATGCATTTCTTAGCTGATATCTATGTCCCT 2280
Akaw_S  741   C  K  G  D  G  L  V  K  V  E  M  H  F  L  P  D  I  Y  V  S   760
Clau_S  741   C  K  G  D  G  L  I  K  V  E  M  H  F  L  P  D  I  Y  V  S   760
Pkil_S  741   C  K  G  D  G  L  I  K  V  E  M  H  F  L  P  D  I  Y  V  S   760
Psoy_S  741   C  K  G  D  G  L  I  K  V  E  M  H  F  L  P  D  I  Y  V  S   760
Vok     741   C  K  G  D  G  L  I  K  V  E  M  H  F  L  S  D  I  Y  V  S   760
Cpac_S  741   C  K  G  D  G  L  I  K  M  E  M  H  F  L  P  D  V  Y  V  S   760
Cfau_S  741   C  K  G  D  G  L  I  K  T  E  M  H  F  L  P  D  V  Y  V  S   760
Cnau_S  741   C  K  G  D  G  L  I  K  V  E  M  H  F  L  P  D  V  Y  V  S   760
Pste_S  741   C  K  G  D  G  L  I  K  V  E  M  H  F  L  P  D  V  Y  V  S   760
Rma     741   C  K  G  D  G  L  I  K  V  E  M  H  F  L  P  D  V  Y  V  L   760
Ifos_S  741   C  K  G  D  G  L  I  K  V  E  M  H  F  L  P  D  V  Y  V  S   760
Apha_S  741   C  K  G  D  G  L  I  K  V  E  M  H  F  L  P  D  V  Y  V  S   760
Bsep_S  741   C  K  G  D  G  L  I  K  V  E  M  H  F  L  A  D  I  Y  V  P   760
```

ABC_UvrA_II domain (612-919)

```
Akaw_S 2281 TGTGATGTATGTCAAGGTCAGCGTTATAATCAACAAACTTTAGAAGTGTTTTATAAAGGA 2340
Clau_S 2281 TGTGATGTATGTCATGGTCAGCGTTATAATCAACAAACTTTAGAAGTATTTTATAAAGGA 2340
Pkil_S 2281 TGTGATATATGTCAAGGTCAGCGTTATAATCAACAAACTTTAGAAGTGTTTTATAAAGGA 2340
Psoy_S 2281 TGTGATATATGTCAAGGTCAGCGTTATAATCAACAAACTTTAGAAGTGTTTTATAAAGGA 2340
Vok    2281 TGTGATGTATGTCAAGGTCAGCGTTATAATCAACAAACTTTAGAAGTGTTTTATAAAGGA 2340
Cpac_S 2281 TGTGATGTGTGCCAAGGCCAGCGTTACAATCAACAAACTTTAGAAGTACTTTACAAGGGA 2340
Cfau_S 2281 TGTGATGTGTGCCAAGGCCAACGTTACAATCAACAAACTTTAGAAGTATTTTACAAGGGA 2340
Cnau_S 2281 TGTGATGTGTGCCGAGGCCAGCGTTACAATCAACAAACTTTAGAAGTACTTTACAAGGGA 2340
Pste_S 2281 TGTGATATGTGCCAAGGTCAGTGTTACAATCAACAAACTTTAGAAGTACTTTACAAGGGA 2340
Rma    2281 TGCGATGTGTGCCAAGGTCAGCGTTACAATCAACAAACTTTAAAAGTGTTTTACAAGGGA 2340
Ifos_S 2281 TGTGATGTGTGCAAAGGCCAGCGTTACAATCAACAAACTTTAGAAGTGCTTTACAAGGGG 2340
Apha_S 2281 TGTGACGTGTGCCAAGGCCAACGTTACAATCAACAAACTTTAGAAATACTTTACAAGGGA 2340
Bsep_S 2281 TGTGATGTTTGCAGCGGCGACCGCTATAATCGTGAAACTTTGGAAATCACCTATAAAGGA 2340
Akaw_S  761   C  D  V  C  Q  G  Q  R  Y  N  Q  Q  T  L  E  V  F  Y  K  G  780
Clau_S  761   C  D  V  C  H  G  Q  R  Y  N  Q  Q  T  L  E  V  F  Y  K  G  780
Pkil_S  761   C  D  I  C  Q  G  Q  R  Y  N  Q  Q  T  L  E  V  F  Y  K  G  780
Psoy_S  761   C  D  I  C  Q  G  Q  R  Y  N  Q  Q  T  L  E  V  F  Y  K  G  780
Vok     761   C  D  V  C  Q  G  Q  R  Y  N  Q  Q  T  L  E  V  F  Y  K  G  780
Cpac_S  761   C  D  V  C  Q  G  Q  R  Y  N  Q  Q  T  L  E  V  L  Y  K  G  780
Cfau_S  761   C  D  V  C  Q  G  Q  R  Y  N  Q  Q  T  L  E  V  F  Y  K  G  780
Cnau_S  761   C  D  V  C  R  G  Q  R  Y  N  Q  Q  T  L  E  V  L  Y  K  G  780
Pste_S  761   C  D  M  C  Q  G  Q  C  Y  N  Q  Q  T  L  E  V  L  Y  K  G  780
Rma     761   C  D  V  C  Q  G  Q  R  Y  N  Q  Q  T  L  K  V  F  Y  K  G  780
Ifos_S  761   C  D  V  C  K  G  Q  R  Y  N  Q  Q  T  L  E  V  L  Y  K  G  780
Apha_S  761   C  D  V  C  Q  G  Q  R  Y  N  Q  Q  T  L  E  I  L  Y  K  G  780
Bsep_S  761   C  D  V  C  S  G  D  R  Y  N  R  E  T  L  E  I  T  Y  K  G  780
```

ABC_UvrA_II domain (612-919)

```
Akaw_S 2341 AAAAGTATTTCACAGGTGCTTGGTATGACCGTAGAACAGGCAATTAAGTTTTTCCAACCT 2400
Clau_S 2341 AAAAGTATTTCACAGGTACTTGCTATGACCGTAGAACAGGCAATTAAGTTTTTCCAACCT 2400
Pkil_S 2341 AAAAGCATTTCGCAGGTACTTGGTATGACTGTAGAACAGGCAATTAGGTTTTTCCAATCT 2400
Psoy_S 2341 AAAAGCATTTCGCAGGTACTTGGTATGACTGTAGAACAGGCAATTAGGTTTTTCCAATCT 2400
Vok    2341 AAAAGCATTTCGCAGGTACTTGGTATGACTGTAGAACAGGCAATTAGGTTTTTCCAATCT 2400
Cpac_S 2341 AAGAGTATTGCACAAGTGCTTGATATGACTGTAGAACAGGCTTGTGAATTTTTCCAACCT 2400
Cfau_S 2341 AAGAGTATTGCACAAGTGCTTGATATGACCGTAGAACAGGCTTGTGAATTTTTTCAACCT 2400
Cnau_S 2341 AAGAGTATTGCACAAGTGCTTGATATGACCGTAGAACAGGCTTGTGAGTTTTTCCAACCT 2400
Pste_S 2341 AAGAGTATTGCACAGGTGCTTGATATGAGCGTAGAACAGGCTTGTGAATTTTTCCAACCT 2400
Rma    2341 AAAAGTATTGCACAGGTGCTTGACATGACTGTAGAACAGGCTTGTGAATTTTTCCAACCC 2400
Ifos_S 2341 AAAAGTATTGCACAGGTGCTTGACATGACCGTAGAACAGGCTTGTGAATTTTTCCAACCC 2400
Apha_S 2341 AAGAGTATTGCACAAGTGCTTGATATGACTGTAGAACAGGCTTGTGAATTTTTTCAACCC 2400
Bsep_S 2341 AAAACGATTGCTGAAATATTAAATATGACTGTTGAAATCGCCGTCAAATTTTTCGATCCC 2400
Akaw_S  781   K  S  I  S  Q  V  L  G  M  T  V  E  Q  A  I  K  F  F  Q  P  800
Clau_S  781   K  S  I  S  Q  V  L  A  M  T  V  E  Q  A  I  K  F  F  Q  P  800
Pkil_S  781   K  S  I  S  Q  V  L  G  M  T  V  E  Q  A  I  R  F  F  Q  S  800
Psoy_S  781   K  S  I  S  Q  V  L  G  M  T  V  E  Q  A  I  R  F  F  Q  S  800
Vok     781   K  S  I  S  Q  V  L  G  M  T  V  E  Q  A  I  R  F  F  Q  S  800
Cpac_S  781   K  S  I  A  Q  V  L  D  M  T  V  E  Q  A  C  E  F  F  Q  P  800
Cfau_S  781   K  S  I  A  Q  V  L  D  M  T  V  E  Q  A  C  E  F  F  Q  P  800
Cnau_S  781   K  S  I  A  Q  V  L  D  M  T  V  E  Q  A  C  E  F  F  Q  P  800
Pste_S  781   K  S  I  A  Q  V  L  D  M  S  V  E  Q  A  C  E  F  F  Q  P  800
Rma     781   K  S  I  A  Q  V  L  D  M  T  V  E  Q  A  C  E  F  F  Q  P  800
Ifos_S  781   K  S  I  A  Q  V  L  D  M  T  V  E  Q  A  C  E  F  F  Q  P  800
Apha_S  781   K  S  I  A  Q  V  L  D  M  T  V  E  Q  A  C  E  F  F  Q  P  800
Bsep_S  781   K  T  I  A  E  I  L  N  M  T  V  E  I  A  V  K  F  F  D  P  800
```

ABC_UvrA_II domain (612-919)

```
Akaw_S 2401 ATACCTAAAATTAAACAAAAACTACAAACTTTAATGGAAGTTGGACTCTCTTATATTACT 2460
Clau_S 2401 ATACCTAAAATTAAACAAAAACTACAAACTCTAATGGAGGTTGGACTTTCTTATATTACT 2460
Pkil_S 2401 ATGCCTAAAATTAAACAAAAACTACAAACTCTAATGGAGGTTGGACTCTCTTATATTACT 2460
Psoy_S 2401 ATGCCTAAAATTAAACAAAAACTACAAACTCTAATGGAGGTTGGACTCTCTTATATTACT 2460
Vok    2401 ATTTCTAAAATTAAACAAAAACTACAAACTCTAATGGAGGTTGGACTCTCTTATATTACT 2460
Cpac_S 2401 ATGCCCAAAATTAAACAAAAATTACAAACTTTAATGGATGTTGGACTTTCCTATATCACC 2460
Cfau_S 2401 ATGCCTAAAATTAAACAAAAATTACAAACTTTAATGGATGTTGGACTCTCCTATATCACC 2460
Cnau_S 2401 ATGCCCAAAATTAAACAAAAATTACAAACTTTAATGGATGTTGGACTTTCCTATATCACC 2460
Pste_S 2401 ATGCCTAAAATTAAACAAAAATTACAAACTTTAATGGATGTTGGGCTCTCCTATATCACC 2460
Rma    2401 ATGCCTAAAATTAAACGAAAATTACAAACTTTAATGGATGTTGGGCTTTCTTATATCATT 2460
Ifos_S 2401 ATGCCTAAAATTAAACAAAAATTACAAACTTTAATGGATGTTGGGCTTTCTTATATCACC 2460
Apha_S 2401 ATGCCCAAGATTAAACAAAAATTACAAACTTTAATGGATGTTGGACTTTCTTATATTACG 2460
Bsep_S 2401 ATTCCAAAAATTAAACAGAAACTGCAAACCCTAATGGATGTCGGTTTATCCTACATTACC 2460
Akaw_S  801   I  P  K  I  K  Q  K  L  Q  T  L  M  E  V  G  L  S  Y  I  T  820
Clau_S  801   I  P  K  I  K  Q  K  L  Q  T  L  M  E  V  G  L  S  Y  I  T  820
Pkil_S  801   M  P  K  I  K  Q  K  L  Q  T  L  M  E  V  G  L  S  Y  I  T  820
Psoy_S  801   M  P  K  I  K  Q  K  L  Q  T  L  M  E  V  G  L  S  Y  I  T  820
Vok     801   I  S  K  I  K  Q  K  L  Q  T  L  M  E  V  G  L  S  Y  I  T  820
Cpac_S  801   M  P  K  I  K  Q  K  L  Q  T  L  M  D  V  G  L  S  Y  I  T  820
Cfau_S  801   M  P  K  I  K  Q  K  L  Q  T  L  M  D  V  G  L  S  Y  I  T  820
Cnau_S  801   M  P  K  I  K  Q  K  L  Q  T  L  M  D  V  G  L  S  Y  I  T  820
Pste_S  801   M  P  K  I  K  Q  K  L  Q  T  L  M  D  V  G  L  S  Y  I  T  820
Rma     801   M  P  K  I  K  R  K  L  Q  T  L  M  D  V  G  L  S  Y  I  I  820
Ifos_S  801   M  P  K  I  K  Q  K  L  Q  T  L  M  D  V  G  L  S  Y  I  T  820
Apha_S  801   M  P  K  I  K  Q  K  L  Q  T  L  M  D  V  G  L  S  Y  I  T  820
Bsep_S  801   I  P  K  I  K  Q  K  L  Q  T  L  M  D  V  G  L  S  Y  I  T  820
```

ABC_UvrA_II domain (612-919)

```
Akaw_S 2461 CTTGGACAAAATGCAACTACTCTATCTGGTGGCGAAGCACAACGTATTAAATTAGCAAAG 2520
Clau_S 2461 CTTGGGCAAAATGCAACTACTTTATCTGGTGGTGAAGCACAACGTATTAAATTAGCAAAG 2520
Pkil_S 2461 CTTGGGCAAAATGCAACTACTTTATCTGGTGGTGAAGCACAACGTATTAAATTAGCAAAG 2520
Psoy_S 2461 CTTGGGCAAAATGCAACTACTTTATCTGGTGGTGAAGCACAACGTATTAAATTAGCAAAG 2520
Vok    2461 CTTGGGCAAAATGCAACTACTTTATCTGGTGGTGAAGCACAACGTATTAAATTAGCAAAG 2520
Cpac_S 2461 CTTGGACAAAATGCAATCACCTTGTCCGGCGGTGAAGCGCAACGCATTAAATTAGCAAAA 2520
Cfau_S 2461 CTTGGACAAAATGCAACCACCTTGTCTGGCGGTGAGGCGCAACGCATTAAATTAGCAAAA 2520
Cnau_S 2461 CTTGGACAAAATGCAACTACCTTGTCTGGCGGTGAGGCACAACGCATTAAATTAGCAAAA 2520
Pste_S 2461 CTTGGACAAAATGTAACTACCTTGTCTGGCGGTGAAGCGCAACGCATTAAATTAGCAAAA 2520
Rma    2461 CTTGGACAAAATGCAACTACCTTATCTGGTGGCGAGGCGCAACGTATTAAATTAGCAAAA 2520
Ifos_S 2461 CTTGGACAAAATGCAACCACCTTATCTGGTGGTGAGGCGCAACGCATCAAATTAGCAAAA 2520
Apha_S 2461 CTTGGACAAAATGCAACCACCTTATCTGGTGGTGAGGCACAACGCATTAAATTAGCAAAA 2520
Bsep_S 2461 CTCGGACAAAATGCTACAACGCTTTCAGGCGGTGAAGCACAACGCATTAAACTCGCCAAA 2520
Akaw_S  821   L  G  Q  N  A  T  T  L  S  G  G  E  A  Q  R  I  K  L  A  K  840
Clau_S  821   L  G  Q  N  A  T  T  L  S  G  G  E  A  Q  R  I  K  L  A  K  840
Pkil_S  821   L  G  Q  N  A  T  T  L  S  G  G  E  A  Q  R  I  K  L  A  K  840
Psoy_S  821   L  G  Q  N  A  T  T  L  S  G  G  E  A  Q  R  I  K  L  A  K  840
Vok     821   L  G  Q  N  A  T  T  L  S  G  G  E  A  Q  R  I  K  L  A  K  840
Cpac_S  821   L  G  Q  N  A  I  T  L  S  G  G  E  A  Q  R  I  K  L  A  K  840
Cfau_S  821   L  G  Q  N  A  T  T  L  S  G  G  E  A  Q  R  I  K  L  A  K  840
Cnau_S  821   L  G  Q  N  A  T  T  L  S  G  G  E  A  Q  R  I  K  L  A  K  840
Pste_S  821   L  G  Q  N  V  T  T  L  S  G  G  E  A  Q  R  I  K  L  A  K  840
Rma     821   L  G  Q  N  A  T  T  L  S  G  G  E  A  Q  R  I  K  L  A  K  840
Ifos_S  821   L  G  Q  N  A  T  T  L  S  G  G  E  A  Q  R  I  K  L  A  K  840
Apha_S  821   L  G  Q  N  A  T  T  L  S  G  G  E  A  Q  R  I  K  L  A  K  840
Bsep_S  821   L  G  Q  N  A  T  T  L  S  G  G  E  A  Q  R  I  K  L  A  K  840
```

ABC_UvrA_II domain (612-919)

```
Akaw_S 2521 GAATTGTCAAGAATGGATACTGGACAAACGCTTTATATTCTTGATGAGCCTACAACGGGT 2580
Clau_S 2521 GAATTGTCAAGAATAGATACTGGACAAACGCTTTATATTCTTGATGAACCGACAACTGGT 2580
Pkil_S 2521 GAATTGTCAAGAATGGATACTGGACAAACGCTTTATATTCTTGATGAGCCGACAACGGGT 2580
Psoy_S 2521 GAATTGTCAAGAATGGATACTGGACAAACGCTTTATATTCTTGATGAGCCGACAACGGGT 2580
Vok    2521 GAATTGTCAAGAATGGATACTGGACGAACGCTTTATATTCTTGATGAGCCGACAACGGGT 2580
Cpac_S 2521 GAGTTGTCAAAAATGGATACTGGACAAACACTCTACATTCTTGATGAACCGACAACAGGC 2580
Cfau_S 2521 GAATTGTCAAAAATGGATACTGGACAAACACTCTACATTCTTGATGAACCGACAACAGGT 2580
Cnau_S 2521 GAGTTGTCAAAAATGGATACTGGACAAACACTCTACATTCTTGATGAACCGACAACAGGT 2580
Pste_S 2521 GAGTTGTCAAAAATGGATACTGGACAAACACTCTACATTCTTGATGAACCGACAATAGGT 2580
Rma    2521 GAATTGTCAAAAATGGATACTGGACAAACACTTTATATTCTTGATGAACCAACAACAGGT 2580
Ifos_S 2521 GAGTTGTCAAAAATGGATACTGGACAAACGCTCTATATTCTTGATGAACCAACAACAGGC 2580
Apha_S 2521 GAGTTGTCAAAAATGGATACTGGACAAACGCTCTACATTCTTGACGAACCAACAACAGGC 2580
Bsep_S 2521 GAACTGTCAAAGCCTGACACTGGACAAACACTCTATATTCTTGATGAGCCAACCACTGGC 2580
Akaw_S  841  E  L  S  R  M  D  T  G  Q  T  L  Y  I  L  D  E  P  T  T  G  860
Clau_S  841  E  L  S  R  I  D  T  G  Q  T  L  Y  I  L  D  E  P  T  T  G  860
Pkil_S  841  E  L  S  R  M  D  T  G  Q  T  L  Y  I  L  D  E  P  T  T  G  860
Psoy_S  841  E  L  S  R  M  D  T  G  Q  T  L  Y  I  L  D  E  P  T  T  G  860
Vok     841  E  L  S  R  M  D  T  G  R  T  L  Y  I  L  D  E  P  T  T  G  860
Cpac_S  841  E  L  S  K  M  D  T  G  Q  T  L  Y  I  L  D  E  P  T  T  G  860
Cfau_S  841  E  L  S  K  M  D  T  G  Q  T  L  Y  I  L  D  E  P  T  T  G  860
Cnau_S  841  E  L  S  K  M  D  T  G  Q  T  L  Y  I  L  D  E  P  T  T  G  860
Pste_S  841  E  L  S  K  M  D  T  G  Q  T  L  Y  I  L  D  E  P  T  I  G  860
Rma     841  E  L  S  K  M  D  T  G  Q  T  L  Y  I  L  D  E  P  T  T  G  860
Ifos_S  841  E  L  S  K  M  D  T  G  Q  T  L  Y  I  L  D  E  P  T  T  G  860
Apha_S  841  E  L  S  K  M  D  T  G  Q  T  L  Y  I  L  D  E  P  T  T  G  860
Bsep_S  841  E  L  S  K  P  D  T  G  Q  T  L  Y  I  L  D  E  P  T  T  G  860
```

ABC_UvrA_II domain (612-919)

```
Akaw_S 2581 TTGCATTTTCATGATATTAAACTTTTGTTATCAGTTATTAATAGATTATGCGAGCGTAAC 2640
Clau_S 2581 TTGCATTTTCATGATATTAAACTTTTATTATCAGTTATTAATAGATTATGCGAACGTAAC 2640
Pkil_S 2581 TTGCATTTTCATGATATTAAACTTTTGTTATCAGTTATTAATAGATTATGCGAGCGTAAC 2640
Psoy_S 2581 TTGCATTTTCATGATATTAAACTTTTGTTATCAGTTATTAATAGATTATGCGAGCGTAAC 2640
Vok    2581 TTGCATTTTCATGATATTAAACTTTTGTTATCAGTTATTAATAGATTATGCGAGCGTAAC 2640
Cpac_S 2581 TTACATTTCCATGATATTAAACTTTTGTTATCAGTTATTAATAGATTACGCGAGCATAAC 2640
Cfau_S 2581 TTACATTTCCATGATATTAAACTTTTGTTATCAGTTATCAATAGATTACGCGAGCATAAC 2640
Cnau_S 2581 TTACATTTCCATGATATTAAACTTTTGTTATCAGTTATTAATAGATTACGCAAGCGTAAC 2640
Pste_S 2581 TTACATTTTCATGATATTAAACTTTTGTTATCAGTTATTAATAGATTACGCGAGCGTAAC 2640
Rma    2581 TTGCATTTTCATGATATTAAACTTCTACTGTCAGTTATTAATAGGTTGCGTGAGCGCAAC 2640
Ifos_S 2581 TTGCATTTTCATGATATTAAACTTTTGCTGTCAGTTATTAATAGGTTGCGTGAGCGCAAC 2640
Apha_S 2581 TTGCATTTTCATGATATTAAACTCTTGTTGTTAGTTATTAATAGGTTGCGTGAGCGCAAC 2640
Bsep_S 2581 CTACATTTTCACGACATCAAACAATTGTTATCTGTGATTATGCGTTTGCGAGAACGAGAA 2640
Akaw_S  861  L  H  F  H  D  I  K  L  L  L  S  V  I  N  R  L  C  E  R  N  880
Clau_S  861  L  H  F  H  D  I  K  L  L  L  S  V  I  N  R  L  C  E  R  N  880
Pkil_S  861  L  H  F  H  D  I  K  L  L  L  S  V  I  N  R  L  C  E  R  N  880
Psoy_S  861  L  H  F  H  D  I  K  L  L  L  S  V  I  N  R  L  C  E  R  N  880
Vok     861  L  H  F  H  D  I  K  L  L  L  S  V  I  N  R  L  C  E  R  N  880
Cpac_S  861  L  H  F  H  D  I  K  L  L  L  S  V  I  N  R  L  R  E  H  N  880
Cfau_S  861  L  H  F  H  D  I  K  L  L  L  S  V  I  N  R  L  R  E  H  N  880
Cnau_S  861  L  H  F  H  D  I  K  L  L  L  S  V  I  N  R  L  R  K  R  N  880
Pste_S  861  L  H  F  H  D  I  K  L  L  L  S  V  I  N  R  L  R  E  R  N  880
Rma     861  L  H  F  H  D  I  K  L  L  L  S  V  I  N  R  L  R  E  R  N  880
Ifos_S  861  L  H  F  H  D  I  K  L  L  L  S  V  I  N  R  L  R  E  R  N  880
Apha_S  861  L  H  F  H  D  I  K  L  L  L  L  V  I  N  R  L  R  E  R  N  880
Bsep_S  861  L  H  F  H  D  I  K  Q  L  L  S  V  I  M  R  L  R  E  R  E  880
```

ABC_UvrA_II domain (612-919)

```
Akaw_S 2641 AATACTATTGTTATTATTGAACATAACCTTGATGTTATTAAAACTGCAGATTGGATTATT 2700
Clau_S 2641 AATACCATTGTTATTATTGAGCATAACCTTGATGTTATTAAAACTGCAGATTGGATTATT 2700
Pkil_S 2641 AATACTATTGTTATTATTGAACATAACCTTGATGTTATTAAAACTGCAGATTGGATTATT 2700
Psoy_S 2641 AATACTATTGTTATTATTGAACATAACCTTGATGTTATTAAAACTGCAGATTGGATTATT 2700
Vok    2641 AATACTATTGTTATTATTGAACATAACCTTGATGTTATTAAAACTGCAGATTGGATTATT 2700
Cpac_S 2641 AATACCATTGTTATCATTGAACACAACCTTGATGTTATTAAAACTGCAGATTGGATTGTT 2700
Cfau_S 2641 AATACCATTGTTATCATTGAACACAACCTTGATGTTATTAAAACTGCAGATTGGATTGTT 2700
Cnau_S 2641 AATACCATTGTTATCATTGAACACAACCTTGATGTTATTAAAACTGCAGATTGGATTGTT 2700
Pste_S 2641 AATACCATTGTTATCATTGAACACAACCTTGATATTATTAAAACTGCAGATTGGATTGTT 2700
Rma    2641 AATACCATTGTGATTATTGAGCATAATCTTGATGTTATTAAAACTGCAGATTGGATTGTT 2700
Ifos_S 2641 AATACCATTGTGATTATTGAGCACAATCTTGATGTTATTAAAACTGCAGATTGGATTGTT 2700
Apha_S 2641 AATACCATTGTGATTATTGAGCACAACCTTGATGTTATTAAAACTGCAGATTGGATTGTT 2700
Bsep_S 2641 AACACGATTGTTATTATTGAGCATAATCTTGATGTTATTAAAACTGCCGATTGGGTTGTA 2700
Akaw_S  881   N  T  I  V  I  I  E  H  N  L  D  V  I  K  T  A  D  W  I  I  900
Clau_S  881   N  T  I  V  I  I  E  H  N  L  D  V  I  K  T  A  D  W  I  I  900
Pkil_S  881   N  T  I  V  I  I  E  H  N  L  D  V  I  K  T  A  D  W  I  I  900
Psoy_S  881   N  T  I  V  I  I  E  H  N  L  D  V  I  K  T  A  D  W  I  I  900
Vok     881   N  T  I  V  I  I  E  H  N  L  D  V  I  K  T  A  D  W  I  I  900
Cpac_S  881   N  T  I  V  I  I  E  H  N  L  D  V  I  K  T  A  D  W  I  V  900
Cfau_S  881   N  T  I  V  I  I  E  H  N  L  D  V  I  K  T  A  D  W  I  V  900
Cnau_S  881   N  T  I  V  I  I  E  H  N  L  D  V  I  K  T  A  D  W  I  V  900
Pste_S  881   N  T  I  V  I  I  E  H  N  L  D  I  I  K  T  A  D  W  I  V  900
Rma     881   N  T  I  V  I  I  E  H  N  L  D  V  I  K  T  A  D  W  I  V  900
Ifos_S  881   N  T  I  V  I  I  E  H  N  L  D  V  I  K  T  A  D  W  I  V  900
Apha_S  881   N  T  I  V  I  I  E  H  N  L  D  V  I  K  T  A  D  W  I  V  900
Bsep_S  881   N  T  I  V  I  I  E  H  N  L  D  V  I  K  T  A  D  W  V  V  900
```

ABC_UvrA_II domain (612-919)

```
Akaw_S 2701 GACTTAGGACCTGAAGGAGGTAACAAAGGTGGGCAGATTATTGCATTTGGTACACCAGAA 2760
Clau_S 2701 GACTTAGGACCTGAAGGGGGTAATAAGGGTGGGCAGATTATTGCATTTGGTACACCAGAG 2760
Pkil_S 2701 GACTTAGGACCTGAGGGGGGTAATAAGGGTGGGCAGATTATTGCATTTGGTACACCAGAA 2760
Psoy_S 2701 GACTTAGGACCTGAGGGGGGTAATAAGGGTGGGCAGATTATTGCATTTGGTACACCAGAA 2760
Vok    2701 GATTTAGGACCTGAGGGAGGTAATAAGGGTGGGCAGATTATTGCATTTGGTACACCAGAA 2760
Cpac_S 2701 GATTTAGGCCCTGAAGGGGGTAATAAGGGTGGGCAAATTATTGCGTTTGGTACGCCAGAA 2760
Cfau_S 2701 GATTTAGGCCCTGAAGGGGGTAATAAGGGTGGGCAAATTATTGCGTTTGGTACACCAGAA 2760
Cnau_S 2701 GATTTAGGCCCTGAAGGGGGTAATAAGGGTGGGAAAATTATTGCGTTTGGTACGCCAGAA 2760
Pste_S 2701 GATTTAGGCCCTGAAGGGGGTAATAAGGGTGGGCAAATTATTGCGTTTGGTACGCCAGAA 2760
Rma    2701 GATTTAGGCCCTGAAGGGGGTAATAAAGGTGGGCAAATTATTGCATTTGGCACACCAGAA 2760
Ifos_S 2701 GATTTAGGCCCTGAAGGGGGTAATAAAGGCGGGCAGATTATTGCATTCGGCACGCCAGAA 2760
Apha_S 2701 GATTTAGGCCCTGAAGGGGGTAATAAGGGTGGACAAATTATTGCGTTTGGTACGCCAGAA 2760
Bsep_S 2701 GATTTAGGCCCTGAAGGTGGCGATAAAGGCGGCAATATCATTGCCACTGGTACGCCAGAA 2760
Akaw_S  901   D  L  G  P  E  G  G  N  K  G  G  Q  I  I  A  F  G  T  P  E  920
Clau_S  901   D  L  G  P  E  G  G  N  K  G  G  Q  I  I  A  F  G  T  P  E  920
Pkil_S  901   D  L  G  P  E  G  G  N  K  G  G  Q  I  I  A  F  G  T  P  E  920
Psoy_S  901   D  L  G  P  E  G  G  N  K  G  G  Q  I  I  A  F  G  T  P  E  920
Vok     901   D  L  G  P  E  G  G  N  K  G  G  Q  I  I  A  F  G  T  P  E  920
Cpac_S  901   D  L  G  P  E  G  G  N  K  G  G  Q  I  I  A  F  G  T  P  E  920
Cfau_S  901   D  L  G  P  E  G  G  N  K  G  G  Q  I  I  A  F  G  T  P  E  920
Cnau_S  901   D  L  G  P  E  G  G  N  K  G  G  K  I  I  A  F  G  T  P  E  920
Pste_S  901   D  L  G  P  E  G  G  N  K  G  G  Q  I  I  A  F  G  T  P  E  920
Rma     901   D  L  G  P  E  G  G  N  K  G  G  Q  I  I  A  F  G  T  P  E  920
Ifos_S  901   D  L  G  P  E  G  G  N  K  G  G  Q  I  I  A  F  G  T  P  E  920
Apha_S  901   D  L  G  P  E  G  G  N  K  G  G  Q  I  I  A  F  G  T  P  E  920
Bsep_S  901   D  L  G  P  E  G  G  D  K  G  G  N  I  I  A  T  G  T  P  E  920
```

ABC_UvrA_II domain (612-919)

```
Akaw_S 2761 GAAGTGGCACAAGTTAAGGAATCATATACAGGAGGATATTTAAAAACTTATGTGTAA 2817
Clau_S 2761 GAAGTGGCACAAGTTAAGGAATCTTATACAGGAGAATATTTAAAAACTTACGTGTAA 2817
Pkil_S 2761 GAAGTGGCACAAGTTAAGGAATCGTATACAGGAGAATATTTAAAAACTTATGTGTAA 2817
Psoy_S 2761 GAAGTGGCACAAGTTAAGGAATCGTATACAGGAGAATATTTAAAAACTTATGTGTAA 2817
Vok    2761 GAAGTGGCACAAGTTAAGGAATCGTATACAGGAGAATATTTAAAAACTTACGTGTAA 2817
Cpac_S 2761 GAGGTGGTAAAATTTAAAGGCTCGTATACGGGTCAATATTTAAAAGCTTACTTGTAG 2817
Cfau_S 2761 GAGGTGGTAAAAGTTAAAGGCTCGTATACGGGTCAATATTTAAAAGCTTACTTGTAG 2817
Cnau_S 2761 GAGGTGGCAAAAGTTAAAGGCTCGTATACGGGTCAATATTTAAAAGCTTACTTGTAG 2817
Pste_S 2761 GAGGTGGTAAAAGTTAAAGGCTCGTATACGGGTCAATATTTAAAAGCTTACTTGTAG 2817
Rma    2761 GAAGTGGCTCAAGTTAAAGGGTCGTATACAGGTGAATATTTAAAAGCTTACTTGTAA 2817
Ifos_S 2761 GAGGTGGCTCAAGTTAAAGGGTCGTACACAGGCGAATATTTAAAAGCTTACTTGTAG 2817
Apha_S 2761 GAGGTGGCAAAAGTTAAAGGCTCGTATACGGGTCAATATTTAAAAGCTTACTTGTAG 2817
Bsep_S 2761 GAAGTTGCAGAGGTTAAAGGTTCTTACACAGGGCAATATTTGAAAGAAATGATTTAA 2817
Akaw_S  921   E  V  A  Q  V  K  E  S  Y  T  G  G  Y  L  K  T  Y  V  *   939
Clau_S  921   E  V  A  Q  V  K  E  S  Y  T  G  E  Y  L  K  T  Y  V  *   939
Pkil_S  921   E  V  A  Q  V  K  E  S  Y  T  G  E  Y  L  K  T  Y  V  *   939
Psoy_S  921   E  V  A  Q  V  K  E  S  Y  T  G  E  Y  L  K  T  Y  V  *   939
Vok     921   E  V  A  Q  V  K  E  S  Y  T  G  E  Y  L  K  T  Y  V  *   939
Cpac_S  921   E  V  V  K  F  K  G  S  Y  T  G  Q  Y  L  K  A  Y  L  *   939
Cfau_S  921   E  V  V  K  V  K  G  S  Y  T  G  Q  Y  L  K  A  Y  L  *   939
Cnau_S  921   E  V  A  K  V  K  G  S  Y  T  G  Q  Y  L  K  A  Y  L  *   939
Pste_S  921   E  V  V  K  V  K  G  S  Y  T  G  Q  Y  L  K  A  Y  L  *   939
Rma     921   E  V  A  Q  V  K  G  S  Y  T  G  E  Y  L  K  A  Y  L  *   939
Ifos_S  921   E  V  A  Q  V  K  G  S  Y  T  G  E  Y  L  K  A  Y  L  *   939
Apha_S  921   E  V  A  K  V  K  G  S  Y  T  G  Q  Y  L  K  A  Y  L  *   939
Bsep_S  921   E  V  A  E  V  K  G  S  Y  T  G  Q  Y  L  K  E  M  I  *   939
```

Reference

1. Thiagalingam S, Grossman L. Both ATPase sites of *Escherichia coli* UvrA have functional roles in nucleotide excision repair. The Journal of biological chemistry. 1991;266(17):11395-403. PubMed PMID: 1828249.
