# Supplementary material for: Loss of genes related to Nucleotide Excision Repair (NER) and implications for reductive genome evolution in symbionts of deep-sea vesicomyid clams
Source: PLoS One. 2017 Feb 15;12(2):e0171274. doi: 10.1371/journal.pone.0171274 (PMC5310779; doi:10.1371/journal.pone.0171274)
Supplement: S2 Fig — The arrangement of nucleotide and amino acid sequences is as described in S1 Fig. Although the ORFs of this gene were shown to be collapsed in clade I symbionts and do not code proteins, residual amino acid coding of nucleotide sequences were estimated and used for manual alignment. # indicates the gap of amino acid sequence where no corresponding nucleotide sequence exist. * indicates stop codon. Conserved domains of uvrB found in NCBI blast search are shown as bidirectional arrows. Specific conserved sequences, 7 consensus helicase motifs of E. coli, are shown below the alignment [2, 3]. Among them, motifs I, Ia, V and VI were well conserved, while motifs II, III and IV were moderately conserved. The conserved motifs suggested that the remaining uvrBs in clade II symbionts were functional. (PDF) [file pone.0171274.s005.pdf]

S2 Fig.

```
Akaw_S    1 GTGGACAATAAATTTCAACTGATATA-AAATTTACTCCTAGTGGAGGTTAG---------   50
Clau_S    1 GTGGACAATAAATTTAAACTGATATC-AAATCTACTTCTAGTGGAGATCAGTCAAAAGCG   59
Pkil_S    1 GTGGACAATAAATTTTAACTGATATC-AAAT-TACTTCTAGTGGAGATTAGCCAAAAGCG   58
Psoy_S    1 GTGGACAATAAATTTTAACTGATATC-AAAT-TACTTCTAGTGGAGATTAGCCAAAAGCG   58
Vok       1 GTGGACAATAAATTTTAACTGATATC-AAATT-ACTCATAGTGGAGATCAGCCAAAAGCG   58
Cpac_S    1 GTGAACAATAAATTTCAATTGATATCGAAATTTTTCCCTAGTGGTGATCAGCCAGAAGCG   60
Cfau_S    1 GTGGATAATAAATTTCAATTGATATCGAAATTTTTCCCTAGTGGCGATCAGCCAGAAGCG   60
Cnau_S    1 GTGGACAATAAATTTCAATTGATATCGAAATTTTCCCCTAGTGGCGATCAGCCAGAGGCG   60
Pste_S    1 GTGGACAATAAATTTCAATTGATATCGAAATTTTCCCCTAGTGGCGATCAGCCAGAAGCG   60
Rma       1 GTGAATAATAAATTTCAACTGATATCGAAATTTTCCCCTAGTGGTGATCAGCCAGAAGCG   60
Ifos_S    1 GTGGACAATAAATTTCAACTGATATCGAAATTTTCCCCTAGTGGGGATCAGCCAGAAGCG   60
Apha_S    1 GTGGACAATAGATTTCAACTGATATCGAAATTTTCCCCTAGTGGTGATCAGCCAGAAGCG   60
Bsep_S    1 ATGGGTAAGAAATTTCAATTAGAATCGCAATTTTCTCCAATGGGAGACCAGCCAAAGGCT   60
Akaw_S    1   M  D  N  K  F  Q  L  I  #  K  F  T  P  S  G  G  *  #  #  #   16
Clau_S    1   M  D  N  K  F  K  L  I  #  K  S  T  S  S  G  D  Q  S  K  A   19
Pkil_S    1   M  D  N  K  F  *  L  I  #  K  #  T  S  S  G  D  *  P  K  A   18
Psoy_S    1   M  D  N  K  F  *  L  I  #  K  #  T  S  S  G  D  *  P  K  A   18
Vok       1   M  D  N  K  F  *  L  I  #  K  #  T  H  S  G  D  Q  P  K  A   18
Cpac_S    1   M  N  N  K  F  Q  L  I  S  K  F  F  P  S  G  D  Q  P  E  A   20
Cfau_S    1   M  D  N  K  F  Q  L  I  S  K  F  F  P  S  G  D  Q  P  E  A   20
Cnau_S    1   M  D  N  K  F  Q  L  I  S  K  F  S  P  S  G  D  Q  P  E  A   20
Pste_S    1   M  D  N  K  F  Q  L  I  S  K  F  S  P  S  G  D  Q  P  E  A   20
Rma       1   M  N  N  K  F  Q  L  I  S  K  F  S  P  S  G  D  Q  P  E  A   20
Ifos_S    1   M  D  N  K  F  Q  L  I  S  K  F  S  P  S  G  D  Q  P  E  A   20
Apha_S    1   M  D  N  R  F  Q  L  I  S  K  F  S  P  S  G  D  Q  P  E  A   20
Bsep_S    1   M  G  K  K  F  Q  L  E  S  Q  F  S  P  M  G  D  Q  P  K  A   20
```

```
Akaw_S   51 ------------------------------------------------------------   50
Clau_S   60 ATTAAAATTTTGCTTGACAGCGTGAATTCT---ACTGCTAAGTTCCAAGCATTACTTGGT  116
Pkil_S   59 ATTAGAATTTTGGTTGATAACGTGAATTGT---GGTGATAAGTTCCAAGTATTACTTGGT  115
Psoy_S   59 ATTAGAATTTTGGTTGATAACGTGAATTGT---GGTGATAAGTTCCAAGTATTACTTGGT  115
Vok      59 ATTAGAATTTTGGTTGACAGCGTGAATTGA--TGGTGATAAGTTCCAAGTATTACTTGGT  116
Cpac_S   61 ATTAAAACTTTGGTTGATGGCGTGAATGCC---GGTGCTAAGTTCCAAACCTTGCTTGGC  117
Cfau_S   61 ATTAAAACTTTGGTTGATGGCGTGAATGCT---GGTGCTAAGTTTCAAACCTTGCTTGGC  117
Cnau_S   61 ATTAAAACTTTGGTTGATGGCGTGAATGCT---GGTGCTAAGTTTCAAACCTTGCTTGGC  117
Pste_S   61 ATTAAAACTTTGGTTGATGGTGTGAATGCT---GGTGCTAAGTTTCAAACCTTGCTTGGC  117
Rma      61 ATTAAAACTTTGATTGATAGTGTGAATGCT---GGCGTTAAGTTTCAAACCTTGCTTGGT  117
Ifos_S   61 ATTAAAACTTTGGTTGATGGTGTGAATGCT---GGTGCTAAGTTTCAAACTTTGCTTGGC  117
Apha_S   61 ATTAAAACTTTAGTTGATGGCGTGAATGCT---GGTGCTAAGTTTCAAACCTTGCTTGGC  117
Bsep_S   61 ATTAAGGCGTTGGTAGAAGGGATTAATGCG---GGTGAAAAATTTCAAACATTGCTTGGC  117
Akaw_S   17   #  #  #  #  #  #  #  #  #  #  #  #  #  #  #  #  #  #  #  #   16
Clau_S   20   I  K  I  L  L  D  S  V  N  S  #  T  A  K  F  Q  A  L  L  G   38
Pkil_S   19   I  R  I  L  V  D  N  V  N  C  #  G  D  K  F  Q  V  L  L  G   37
Psoy_S   19   I  R  I  L  V  D  N  V  N  C  #  G  D  K  F  Q  V  L  L  G   37
Vok      19   I  R  I  L  V  D  S  V  N  *  #  G  D  K  F  Q  V  L  L  G   37
Cpac_S   21   I  K  T  L  V  D  G  V  N  A  #  G  A  K  F  Q  T  L  L  G   39
Cfau_S   21   I  K  T  L  V  D  G  V  N  A  #  G  A  K  F  Q  T  L  L  G   39
Cnau_S   21   I  K  T  L  V  D  G  V  N  A  #  G  A  K  F  Q  T  L  L  G   39
Pste_S   21   I  K  T  L  V  D  G  V  N  A  #  G  A  K  F  Q  T  L  L  G   39
Rma      21   I  K  T  L  I  D  S  V  N  A  #  G  V  K  F  Q  T  L  L  G   39
Ifos_S   21   I  K  T  L  V  D  G  V  N  A  #  G  A  K  F  Q  T  L  L  G   39
Apha_S   21   I  K  T  L  V  D  G  V  N  A  #  G  A  K  F  Q  T  L  L  G   39
Bsep_S   21   I  K  A  L  V  E  G  I  N  A  #  G  E  K  F  Q  T  L  L  G   39

E. coli                                               L  A  H  Q  T  L  L  G
                                                             Motif I
```

DEAD-like helicases superfamily domain (37-99)

```
Akaw_S    51 ----------------------------------------------------------------  50
Clau_S   117 GTAACTGGTTCAGGAAAGATCTTC---AAACTAGCAAATGTTATTCACTAGACTAAACGC  173
Pkil_S   116 GTAACTGGTTCGGGAAGAACCTTTTC-ACCCTAGCTAATGTTGTTTACTAGATTAAATAC  174
Psoy_S   116 GTAACTGGTTCGGGAAGAACCTTTTC-ACCCTAGCTAATGTTGTTTACTAGATTAAATAC  174
Vok      117 GTAATTGGTTTAGGAAAGACCTTTTC-ACCATAGCTAATGTTATTTACTAGACTAAATAC  175
Cpac_S   118 GTAACTGGCTCGGGTAAAACCTTT---ACGCTGGCTAATGTTATTCAACAGACCAAACGC  174
Cfau_S   118 GTAACTGGCTCGGGTAAAACCTTT---ACACTAGCTAATGTTATTCAACAGACCAAACGC  174
Cnau_S   118 GTAACTGGCTCGGGTAAAACCTTT---ACACTGGCTAATGTTATTCAACAGACCAAACGC  174
Pste_S   118 GTAACTGGCTCGGGTAAAACCTTT---ACACTGGCTAATGTTATTCAACAGACCAAACGC  174
Rma      118 GTAACTGGTTCGGGTAAAACCTTT---ACACTGGCTAATGTTATTCAACAAACTAAACGT  174
Ifos_S   118 GTAACTGGCTCGGGTAAAACCTTT---ACATTGGCCAATGTTATTCAACAGACTAAGCGC  174
Apha_S   118 GTAACTGGATCGGGTAAAACCTTT---ACACTGGCTAATGTTATTCAACAGACCGAGCGC  174
Bsep_S   118 GTTACAGGGTCTGGTAAGACTTTT---ACCTTGGCAAATGTTATTCAACAGACTAAGCGT  174
Akaw_S    17  #  #  #  #  #  #  #  #  #  #  #  #  #  #  #  #  #  #  #  #   16
Clau_S    39  V  T  G  S  G  K  I  F  #  K  L  A  N  V  I  H  *  T  K  R   57
Pkil_S    38  V  T  G  S  G  R  T  F  #  T  L  A  N  V  V  Y  *  I  K  Y   56
Psoy_S    38  V  T  G  S  G  R  T  F  #  T  L  A  N  V  V  Y  *  I  K  Y   56
Vok       38  V  I  G  L  G  K  T  F  #  T  I  A  N  V  I  Y  *  T  K  Y   56
Cpac_S    40  V  T  G  S  G  K  T  F  #  T  L  A  N  V  I  Q  Q  T  K  R   58
Cfau_S    40  V  T  G  S  G  K  T  F  #  T  L  A  N  V  I  Q  Q  T  K  R   58
Cnau_S    40  V  T  G  S  G  K  T  F  #  T  L  A  N  V  I  Q  Q  T  K  R   58
Pste_S    40  V  T  G  S  G  K  T  F  #  T  L  A  N  V  I  Q  Q  T  K  R   58
Rma       40  V  T  G  S  G  K  T  F  #  T  L  A  N  V  I  Q  Q  T  K  R   58
Ifos_S    40  V  T  G  S  G  K  T  F  #  T  L  A  N  V  I  Q  Q  T  K  R   58
Apha_S    40  V  T  G  S  G  K  T  F  #  T  L  A  N  V  I  Q  Q  T  E  R   58
Bsep_S    40  V  T  G  S  G  K  T  F  #  T  L  A  N  V  I  Q  Q  T  K  R   58

E. coli       V  T  G  S  G  K  T  F     T                             R
                        Motif I
```

DEAD-like helicases superfamily domain (37-99)

```
Akaw_S    51 ----------------------------------------------------------------  50
Clau_S   174 CTTAGTTTAATTATGGTA---TCCAACAAA-CATTAGTCGCAGAATTGTATAATGAAATG  229
Pkil_S   175 CCTAATTTAATTACGGCACTCCCCAATAAAACATTAACAGCACAATTGTATAATGAAATG  234
Psoy_S   175 CCTAATTTAATTACGGCACTCCCCAATAAAACATTAACAGCACAATTGTATAATGAAATG  234
Vok      176 CCTAGTTTAATTATAGCC-----------------------------------------  193
Cpac_S   175 CCTAGTTTGATTATGGCA---CCCAACAAAACATTAGCAGCACAATTGTATAGTGAGATG  231
Cfau_S   175 CCCAGTTTGATTATGGCA---CCCAACAAAACATTAGCAGCACAATTGTATAGTGAGATG  231
Cnau_S   175 CCCAGCTTGATTATGGCG---CCCAACAAAACATTAGCAGCACAATTGTATAGTGAGATG  231
Pste_S   175 CCCAGTTTGATTATGGCG---CCCAACAAAACATTAGCAGCACAATTGTATAGTGAGATG  231
Rma      175 CCTAGTTTGATTATGGCG---CCTAATAAAACATTAGCAGCACAATTGTATAGTGAGATG  231
Ifos_S   175 CCCAGTTTGATTATGGCA---CCCAATAAAACATTAGCAGCACAATTGTATAGTGAGATG  231
Apha_S   175 TCCAGTTTGATTATGGCG---CCCAATAAAACATTAGCAGCACAATTATATAGTGAGATG  231
Bsep_S   175 GCAAGTTTAATTATAGCG---CCAAATAAAACGCTGGCGGCACAGTTATACAGTGAAATG  231
Akaw_S    17  #  #  #  #  #  #  #  #  #  #  #  #  #  #  #  #  #  #  #  #   16
Clau_S    58  L  S  L  I  M  V  #  S  N  K  #  L  V  A  E  L  Y  N  E  M   75
Pkil_S    57  P  N  L  I  T  A  L  P  N  K  T  L  T  A  Q  L  Y  N  E  M   76
Psoy_S    57  P  N  L  I  T  A  L  P  N  K  T  L  T  A  Q  L  Y  N  E  M   76
Vok       57  P  S  L  I  I  A  #  #  #  #  #  #  #  #  #  #  #  #  #  #   62
Cpac_S    59  P  S  L  I  M  A  #  P  N  K  T  L  A  A  Q  L  Y  S  E  M   77
Cfau_S    59  P  S  L  I  M  A  #  P  N  K  T  L  A  A  Q  L  Y  S  E  M   77
Cnau_S    59  P  S  L  I  M  A  #  P  N  K  T  L  A  A  Q  L  Y  S  E  M   77
Pste_S    59  P  S  L  I  M  A  #  P  N  K  T  L  A  A  Q  L  Y  S  E  M   77
Rma       59  P  S  L  I  M  A  #  P  N  K  T  L  A  A  Q  L  Y  S  E  M   77
Ifos_S    59  P  S  L  I  M  A  #  P  N  K  T  L  A  A  Q  L  Y  S  E  M   77
Apha_S    59  S  S  L  I  M  A  #  P  N  K  T  L  A  A  Q  L  Y  S  E  M   77
Bsep_S    59  A  S  L  I  I  A  #  P  N  K  T  L  A  A  Q  L  Y  S  E  M   77

E. coli       P  T  M  V  L  A     P  N  K  T  L  A  A  Q  L  Y  G  E  M
                                         Motif Ia
```

DEAD-like helicases superfamily domain (37-99)

```
Akaw_S   51 ------------------------------------------------------------  50
Clau_S  230 AAAAAGTTATTTTCATATAATTCAATGGAATATTTTGTTTCTTATTATGATTATTATCAA 289
Pkil_S  235 AAAGAGCTCTTTCTACATAATTCAGTTGAATATTTTGTTTCTTGTTATGATTATTATCAG 294
Psoy_S  235 AAAGAGCTCTTTCTACATAATTCAGTTGAATATTTTGTTTCTTGTTATGATTATTATCAG 294
Vok     194 ------------------------------------------------------------ 193
Cpac_S  232 AAAGAGTTTTTTCCACACAATTCGGTGGAATATTTTGTTTCTTATTATGATTATTATCAG 291
Cfau_S  232 AAAGAGTTTTTTCCACACAATTCGGTGGAATATTTTGTTTCTTATTATGATTATTACCAA 291
Cnau_S  232 AAAGAGTTTTTTCCACACAATTCGGTGGAATATTTTGTTTCTTATTATGATTATTATCAG 291
Pste_S  232 AAAGAGTTTTTTCCACACAATTCGGTGGAATATTTTGTTTCTTATTATGATTATTATCAG 291
Rma     232 AAAGAGTTTTTTCCGTATAATTCGGTGGAATATTTTGTTTCTTATTATGATTATTATCAG 291
Ifos_S  232 AAGGAGTTTTTTCCACACAATTCAGTGGAATATTTTGTTTCTTATTATGATTATTATCAG 291
Apha_S  232 AAAGAGTTTTTTCCACACAATTCAGTGGAATATTTTGTTTCTTATTATGACTATTATCAG 291
Bsep_S  232 AAAGCATTTTTCCCCAATAATGCGGTGGAATATTTTGTTTCTTATTATGATTATTACCAG 291
Akaw_S   17   #  #  #  #  #  #  #  #  #  #  #  #  #  #  #  #  #  #  #  #  16
Clau_S   76   K  K  L  F  S  Y  N  S  M  E  Y  F  V  S  Y  Y  D  Y  Y  Q  95
Pkil_S   77   K  E  L  F  L  H  N  S  V  E  Y  F  V  S  C  Y  D  Y  Y  Q  96
Psoy_S   77   K  E  L  F  L  H  N  S  V  E  Y  F  V  S  C  Y  D  Y  Y  Q  96
Vok      63   #  #  #  #  #  #  #  #  #  #  #  #  #  #  #  #  #  #  #  #  62
Cpac_S   78   K  E  F  F  P  H  N  S  V  E  Y  F  V  S  Y  Y  D  Y  Y  Q  97
Cfau_S   78   K  E  F  F  P  H  N  S  V  E  Y  F  V  S  Y  Y  D  Y  Y  Q  97
Cnau_S   78   K  E  F  F  P  H  N  S  V  E  Y  F  V  S  Y  Y  D  Y  Y  Q  97
Pste_S   78   K  E  F  F  P  H  N  S  V  E  Y  F  V  S  Y  Y  D  Y  Y  Q  97
Rma      78   K  E  F  F  P  Y  N  S  V  E  Y  F  V  S  Y  Y  D  Y  Y  Q  97
Ifos_S   78   K  E  F  F  P  H  N  S  V  E  Y  F  V  S  Y  Y  D  Y  Y  Q  97
Apha_S   78   K  E  F  F  P  H  N  S  V  E  Y  F  V  S  Y  Y  D  Y  Y  Q  97
Bsep_S   78   K  A  F  F  P  N  N  A  V  E  Y  F  V  S  Y  Y  D  Y  Y  Q  97

E. coli       K
```

DEAD-like helicases superfamily domain (37-99)

```
Akaw_S   51 ------------------------------------------------------------  50
Clau_S  290 CTAGAGATTTATGTTCCTGCTTTTGATACT---ATC------------------------ 322
Pkil_S  295 ATAGAGGCTTATATTCCTGTTTCTGATACTTATATCGAAAAGGATTCTTCTATTAATAAA 354
Psoy_S  295 ATAGAGGCTTATATTCCTGTTTCTGATACTTATATCGAAAAGGATTCTTCTATTAATAAA 354
Vok     194 ------------------------------------------------------------ 193
Cpac_S  292 CCAGAGGCTTATGTGCCTGCGTCTGATACTTATATTGAAAAAGATTCTTCTATTAATGAG 351
Cfau_S  292 CCAGAGGCTTATGTACCTGCGTCTGATACTTATATCGAAAAAGATTCTTCTATTAATGAG 351
Cnau_S  292 CCAGAGGCTTATGTGCCTGCGTCTGATACTTATATCGAAAAAGATTCTTCTATTAATGAG 351
Pste_S  292 CCAGAGGCTTATGTGCCTGTGTCTGATACTTATATCGAAAAAGATTCTTCTATTAATGAG 351
Rma     292 CCAGAAGCTTATGTACCTGCCTCTGATACTTATATCGAAAAAGACTCTTCTATTAATGAG 351
Ifos_S  292 CCAGAAGCTTATGTACCCGCCTCTGATACTTATATCGAAAAAGACTCTTCTATTAATGAG 351
Apha_S  292 CCAGAAGCTTATGTACCTGCTTCTGATACTTATATTGAAAAAGATTCTTCTATTAATGAA 351
Bsep_S  292 CCAGAAGCGTATGTGCCAGCCAGTGATACTTATATTGAAAAAGATTCGTCGATTAACGAG 351
Akaw_S   17   #  #  #  #  #  #  #  #  #  #  #  #  #  #  #  #  #  #  #  #  16
Clau_S   96   L  E  I  Y  V  P  A  F  D  T  #  I  #  #  #  #  #  #  #  # 106
Pkil_S   97   I  E  A  Y  I  P  V  S  D  T  Y  I  E  K  D  S  S  I  N  K 116
Psoy_S   97   I  E  A  Y  I  P  V  S  D  T  Y  I  E  K  D  S  S  I  N  K 116
Vok      63   #  #  #  #  #  #  #  #  #  #  #  #  #  #  #  #  #  #  #  #  62
Cpac_S   98   P  E  A  Y  V  P  A  S  D  T  Y  I  E  K  D  S  S  I  N  E 117
Cfau_S   98   P  E  A  Y  V  P  A  S  D  T  Y  I  E  K  D  S  S  I  N  E 117
Cnau_S   98   P  E  A  Y  V  P  A  S  D  T  Y  I  E  K  D  S  S  I  N  E 117
Pste_S   98   P  E  A  Y  V  P  V  S  D  T  Y  I  E  K  D  S  S  I  N  E 117
Rma      98   P  E  A  Y  V  P  A  S  D  T  Y  I  E  K  D  S  S  I  N  E 117
Ifos_S   98   P  E  A  Y  V  P  A  S  D  T  Y  I  E  K  D  S  S  I  N  E 117
Apha_S   98   P  E  A  Y  V  P  A  S  D  T  Y  I  E  K  D  S  S  I  N  E 117
Bsep_S   98   P  E  A  Y  V  P  A  S  D  T  Y  I  E  K  D  S  S  I  N  E 117
```

DEAD-like helicases superfamily domain (37-99)

```
Akaw_S   51 ------------------------------------------------------------  50
Clau_S  323 ---------------------------------------AAGGCTTTATTAGAGTGTGAT 343
Pkil_S  355 CAAATCGAGCAAATGCTTCTTGGGTCCGCCTATT--ACCAAGGCTCTATTAGAGCGTGA- 411
Psoy_S  355 CAAATCGAGCAAATGCTTCTTGGGTCCGCCTATT--ACCAAGGCTCTATTAGAGCGTGA- 411
Vok     194 ------------------------------------------------------------ 193
Cpac_S  352 CAGATTGAGCAAATGCGCCTA---TCTGCC------ACTAAAGCACTATTAGAGCGTGAT 402
Cfau_S  352 CAAATTGAGCAAATGCGCCTA---TCTGCC------ACCAAAGCACTATTAGAGCGTGAT 402
Cnau_S  352 CAAATTGAGCAAATGCGCCTA---TCTGCC------ACTAAAGCTCTATTAGAACGTGAT 402
Pste_S  352 CAAATTGAGCAAATGCGCCTA---TCTGCC------ACCAAAGCTCTATTAGAGCATGAT 402
Rma     352 CAAATAGAGCAAATGCGCTTA---TCTGCT------ACTAAGGCCTTATTAGAGCGTGAT 402
Ifos_S  352 CAAATTAAGCAAATGCGCCTA---TCTGCC------ACTAAGGCGTTATTAGAGCGTGAT 402
Apha_S  352 CAAATCGAGCAAATGCGTCTA---TCTGCT------ACCAAGGCACTATTAGAGCGTGAT 402
Bsep_S  352 CAGATTGAGCAGATGCGTTTG---AGTGCA------ACCAAAGCATTATTGGAGCGAGAT 402
Akaw_S   17  #  #  #  #  #  #  #  #  #  #  #  #  #  #  #  #  #  #  #  #   16
Clau_S  107  #  #  #  #  #  #  #  #  #  #  #  #  #  K  A  L  L  E  C  D  113
Pkil_S  117  Q  I  E  Q  M  L  L  G  S  A  Y  #  T  K  A  L  L  E  R  #  134
Psoy_S  117  Q  I  E  Q  M  L  L  G  S  A  Y  #  T  K  A  L  L  E  R  #  134
Vok      63  #  #  #  #  #  #  #  #  #  #  #  #  #  #  #  #  #  #  #  #   62
Cpac_S  118  Q  I  E  Q  M  R  L  #  S  A  #  #  T  K  A  L  L  E  R  D  134
Cfau_S  118  Q  I  E  Q  M  R  L  #  S  A  #  #  T  K  A  L  L  E  R  D  134
Cnau_S  118  Q  I  E  Q  M  R  L  #  S  A  #  #  T  K  A  L  L  E  R  D  134
Pste_S  118  Q  I  E  Q  M  R  L  #  S  A  #  #  T  K  A  L  L  E  H  D  134
Rma     118  Q  I  E  Q  M  R  L  #  S  A  #  #  T  K  A  L  L  E  R  D  134
Ifos_S  118  Q  I  K  Q  M  R  L  #  S  A  #  #  T  K  A  L  L  E  R  D  134
Apha_S  118  Q  I  E  Q  M  R  L  #  S  A  #  #  T  K  A  L  L  E  R  D  134
Bsep_S  118  Q  I  E  Q  M  R  L  #  S  A  #  #  T  K  A  L  L  E  R  D  134
```

```
Akaw_S   51 ------------------------------------------------------------  50
Clau_S  344 GATGTTATTATTATTGCTAGTGTATCTACTATTTATGGTTTG---AGGAATATTTAAATT 400
Pkil_S  412 GTTATTATTATTGTT---AGTATGTTTGCTATTTATGATTTG-GTCGG---ATC------ 458
Psoy_S  412 GTTATTATTATTGTT---AGTATGTTTGCTATTTATGATTTG-GTCGG---ATC------ 458
Vok     194 ------------------------------------------------------------ 193
Cpac_S  403 GATGTTATCATTATTGCCAGTGTATCTGCCATTTATGGCTTG---GGTAATCCTGAAAGT 459
Cfau_S  403 GATGTTATTATTATTGCCAGTGTATCTGCTATTTATGGCTTG---GGTAATCCTGAAAGT 459
Cnau_S  403 GATGTTATTATTATTGCCAGTGTATCTGCTATTTATGGCTTG---GGTAATCCTGAAAGT 459
Pste_S  403 GATGTTATTATTATTGCCAGTGTATCTGCTATTTACGGCTTG---GGTAATCCTGAAAGT 459
Rma     403 GATGTTATTATCATTGCTAGTGTGTCTGCTATTTATGGCTTG---GGTAACCCTGAAAGT 459
Ifos_S  403 GATGTTATTATTATTGCTAGTGTGTCTGCTATTTATGGCCTG---GGCAACCCTGAAAGT 459
Apha_S  403 GATGTGATTATTATTGCCAGTGTGTCTGCTATTTATGGCTTG---GGAAATCCTGAAAGT 459
Bsep_S  403 GATGTGATTATTATTGCCAGTGTGTCGGCAATTTATGGCTTG---GGAGACCCTGATAGC 459
Akaw_S   17  #  #  #  #  #  #  #  #  #  #  #  #  #  #  #  #  #  #  #  #   16
Clau_S  114  D  V  I  I  I  A  S  V  S  T  I  Y  G  L  #  R  N  I  *  I  132
Pkil_S  135  V  I  I  I  V  #  S  M  F  A  I  Y  D  L  #  R  #  I  #  #  149
Psoy_S  135  V  I  I  I  V  #  S  M  F  A  I  Y  D  L  #  R  #  I  #  #  149
Vok      63  #  #  #  #  #  #  #  #  #  #  #  #  #  #  #  #  #  #  #  #   62
Cpac_S  135  D  V  I  I  I  A  S  V  S  A  I  Y  G  L  #  G  N  P  E  S  153
Cfau_S  135  D  V  I  I  I  A  S  V  S  A  I  Y  G  L  #  G  N  P  E  S  153
Cnau_S  135  D  V  I  I  I  A  S  V  S  A  I  Y  G  L  #  G  N  P  E  S  153
Pste_S  135  D  V  I  I  I  A  S  V  S  A  I  Y  G  L  #  G  N  P  E  S  153
Rma     135  D  V  I  I  I  A  S  V  S  A  I  Y  G  L  #  G  N  P  E  S  153
Ifos_S  135  D  V  I  I  I  A  S  V  S  A  I  Y  G  L  #  G  N  P  E  S  153
Apha_S  135  D  V  I  I  I  A  S  V  S  A  I  Y  G  L  #  G  N  P  E  S  153
Bsep_S  135  D  V  I  I  I  A  S  V  S  A  I  Y  G  L  #  G  D  P  D  S  153
```

```
Akaw_S   51 ------------------------------------------------------------   50
Clau_S  401 TATATGATGATGCTACTACATTTTAGTATTGGGAAAATTATTAATCAGCGTAGAATAATT  460
Pkil_S  459 ------------------------------------------------------------  458
Psoy_S  459 ------------------------------------------------------------  458
Vok     194 ------------------------------------------------------------  193
Cpac_S  460 TATATGGCTATGTTGCTACATTTGAGCATTGGAGAAGTTACTAATCAGCGTGAGATACTT  519
Cfau_S  460 TATATGGCTATGTTACTACATTTGAGCATTGGAGAAGTTACTAATCAGCGTGAGATACTT  519
Cnau_S  460 TATATGGCTATGTTGCTACATTTGAGTATTGGGGAAGTTACTAATCAACGTGAGATACTT  519
Pste_S  460 TATATGGCTATGTTGCTACATTTGAGTATTGGGGAAGTTACTAATCAGCGTGAGATACTT  519
Rma     460 TATATGGCCATGTTGCTGCATTTGAGCGTTGGAGAAGTTACTAATCAGCGTGAGATACTT  519
Ifos_S  460 TATATGGCCATGTTGCTGCATTTGAGTGTCGGGGAAATTACTAATCAGCGTGAGATACTT  519
Apha_S  460 TATATGGCCATGTTGCTACATTTGAGTATTGGGGAAATTATTAATCAGCGCGAGATACTT  519
Bsep_S  460 TATATGCAAATGTTATTGCATTTGAGTGTTGGTGAAATTATTAATCAACGAGAAATCTTA  519
Akaw_S   17   #  #  #  #  #  #  #  #  #  #  #  #  #  #  #  #  #  #  #  #   16
Clau_S  133   Y  M  M  M  L  L  H  F  S  I  G  K  I  I  N  Q  R  R  I  I  152
Pkil_S  150   #  #  #  #  #  #  #  #  #  #  #  #  #  #  #  #  #  #  #  #  149
Psoy_S  150   #  #  #  #  #  #  #  #  #  #  #  #  #  #  #  #  #  #  #  #  149
Vok      63   #  #  #  #  #  #  #  #  #  #  #  #  #  #  #  #  #  #  #  #   62
Cpac_S  154   Y  M  A  M  L  L  H  L  S  I  G  E  V  T  N  Q  R  E  I  L  173
Cfau_S  154   Y  M  A  M  L  L  H  L  S  I  G  E  V  T  N  Q  R  E  I  L  173
Cnau_S  154   Y  M  A  M  L  L  H  L  S  I  G  E  V  T  N  Q  R  E  I  L  173
Pste_S  154   Y  M  A  M  L  L  H  L  S  I  G  E  V  T  N  Q  R  E  I  L  173
Rma     154   Y  M  A  M  L  L  H  L  S  V  G  E  V  T  N  Q  R  E  I  L  173
Ifos_S  154   Y  M  A  M  L  L  H  L  S  V  G  E  I  T  N  Q  R  E  I  L  173
Apha_S  154   Y  M  A  M  L  L  H  L  S  I  G  E  I  I  N  Q  R  E  I  L  173
Bsep_S  154   Y  M  Q  M  L  L  H  L  S  V  G  E  I  I  N  Q  R  E  I  L  173
```

```
Akaw_S   51 ------------------------------------------------------------   50
Clau_S  461 TCACGCCTATCACAAATAAAATACTCACCTAATGACATGATTTTAATACGTGGCTATTTT  520
Pkil_S  459 ------------------------------------------------------------  458
Psoy_S  459 ------------------------------------------------------------  458
Vok     194 ------------------------------------------------------------  193
Cpac_S  520 TCACGCTTGTCACAAATGCAATACTCGCGCAATGATATTACTTTGATGCGTGGCTATTTC  579
Cfau_S  520 TCACGCTTGTCACAAATGCAATACTCGCGCAATGATATTACTTTGATGCGTGGCCATTTT  579
Cnau_S  520 TCACGCTTGTCACAAATGCAATATTCGCGCAATGATATTACTTTGATGCGTGGCCATTTT  579
Pste_S  520 TCACGCTTGTCACAAATGCAATACTCGCGCAATGATATTACTTTGATGCGTGGCCATTTT  579
Rma     520 TTACGTTTGTCGCAAATGCAATATTCGCGTAATGACGTTACTTTAATACGTGGTCATTTT  579
Ifos_S  520 TCACGTTTGTCGCAAATGCAATATTCGCGTAGTAATGCTACTTTAATACGTGGTCATTTT  579
Apha_S  520 TCACGCTTGTCACAGATGCAATACTCGCGCAATGATATTACTTTGATGCGTGGCCATTTT  579
Bsep_S  520 TCTCGTTTGTCGCAGATGCAATACAGCCGCAATGATGTAAGTTTAATGCGTGGTAGTTTT  579
Akaw_S   17   #  #  #  #  #  #  #  #  #  #  #  #  #  #  #  #  #  #  #  #   16
Clau_S  153   S  R  L  S  Q  I  K  Y  S  P  N  D  M  I  L  I  R  G  Y  F  172
Pkil_S  150   #  #  #  #  #  #  #  #  #  #  #  #  #  #  #  #  #  #  #  #  149
Psoy_S  150   #  #  #  #  #  #  #  #  #  #  #  #  #  #  #  #  #  #  #  #  149
Vok      63   #  #  #  #  #  #  #  #  #  #  #  #  #  #  #  #  #  #  #  #   62
Cpac_S  174   S  R  L  S  Q  M  Q  Y  S  R  N  D  I  T  L  M  R  G  Y  F  193
Cfau_S  174   S  R  L  S  Q  M  Q  Y  S  R  N  D  I  T  L  M  R  G  H  F  193
Cnau_S  174   S  R  L  S  Q  M  Q  Y  S  R  N  D  I  T  L  M  R  G  H  F  193
Pste_S  174   S  R  L  S  Q  M  Q  Y  S  R  N  D  I  T  L  M  R  G  H  F  193
Rma     174   L  R  L  S  Q  M  Q  Y  S  R  N  D  V  T  L  I  R  G  H  F  193
Ifos_S  174   S  R  L  S  Q  M  Q  Y  S  R  S  N  A  T  L  I  R  G  H  F  193
Apha_S  174   S  R  L  S  Q  M  Q  Y  S  R  N  D  I  T  L  M  R  G  H  F  193
Bsep_S  174   S  R  L  S  Q  M  Q  Y  S  V  N  D  V  S  L  M  R  G  S  F  193
```

```
Akaw_S    51 ------------------------------------------------------------   50
Clau_S   521 TAGG--GTTAAAGGTTAAGTGATTGATATTTTCCCTGCAGATTCTAAAGAGCAAGGTATA  578
Pkil_S   459 ------------------------------------------------------------  458
Psoy_S   459 ------------------------------------------------------------  458
Vok      194 ------------------------------------------------------------  193
Cpac_S   580 CGA---GTTAAGGGCGAAGTGATTGATATTTTTCCTGCTGATTCTGAAGAGCAAGCCATA  636
Cfau_S   580 CGA---GTTAAGGGCGAAGTGATTGATATTTTCCCTGCTGATTCTGAAGAGCAAGCCATA  636
Cnau_S   580 CGA---GTTAAGGGTGAAGTGATTGATATTTTCCCTGCTGATTCTGAAAAGCAAGCCATA  636
Pste_S   580 CGA---GTTAAGGGCGAAGTGATTGATATTTTCCCTGCTGATTCTGAAGAGCAAGCCATA  636
Rma      580 AGA---GTTAAAGGTGAAGTGATTGATATTTTCCCTGCTGATTCTGAAGAACAGACCATA  636
Ifos_S   580 CAA---GTTAAAGGTGAAGTGATTGATATTTTTCCTGCTGATTCTGAAGAACAAGCCATA  636
Apha_S   580 CGA---GTTAAGGGTGAAGTGATTGATATTTTCCCTGCTGATTCTGAAGAGCAAGCCATA  636
Bsep_S   580 CGC---GTAAAAGGTGAGGTGATTGATATTTTCCCCGCAGATTCGGAAGAGCAGGCATTA  636
Akaw_S    17   #  #  #  #  #  #  #  #  #  #  #  #  #  #  #  #  #  #  #  #   16
Clau_S   173   *  #  V  K  G  *  V  I  D  I  F  P  A  D  S  K  E  Q  G  I  191
Pkil_S   150   #  #  #  #  #  #  #  #  #  #  #  #  #  #  #  #  #  #  #  #  149
Psoy_S   150   #  #  #  #  #  #  #  #  #  #  #  #  #  #  #  #  #  #  #  #  149
Vok       63   #  #  #  #  #  #  #  #  #  #  #  #  #  #  #  #  #  #  #  #   62
Cpac_S   194   R  #  V  K  G  E  V  I  D  I  F  P  A  D  S  E  E  Q  A  I  212
Cfau_S   194   R  #  V  K  G  E  V  I  D  I  F  P  A  D  S  E  E  Q  A  I  212
Cnau_S   194   R  #  V  K  G  E  V  I  D  I  F  P  A  D  S  E  K  Q  A  I  212
Pste_S   194   R  #  V  K  G  E  V  I  D  I  F  P  A  D  S  E  E  Q  A  I  212
Rma      194   R  #  V  K  G  E  V  I  D  I  F  P  A  D  S  E  E  Q  T  I  212
Ifos_S   194   Q  #  V  K  G  E  V  I  D  I  F  P  A  D  S  E  E  Q  A  I  212
Apha_S   194   R  #  V  K  G  E  V  I  D  I  F  P  A  D  S  E  E  Q  A  I  212
Bsep_S   194   R  #  V  K  G  E  V  I  D  I  F  P  A  D  S  E  E  Q  A  L  212
```

```
Akaw_S    51 ------------------------------------------------------------   50
Clau_S   579 CGTATTGAGATGTTTAATGAATAAATTGAACAACTTTATTGGTTTGATTCTTTAATAGGT  638
Pkil_S   459 ------------------------------------------------------------  458
Psoy_S   459 ------------------------------------------------------------  458
Vok      194 ------------------------------------------------------------  193
Cpac_S   637 CGCATTGAAATGTTTGATGAAGAAATTGAACAACTTTATTGGTTTGACCCTTTAACAGGG  696
Cfau_S   637 CGCATTGAAATGTTTGATGAAGAAATTGAACAACTTTATTGGTTTGATCCTTTAACAGGG  696
Cnau_S   637 CGCATTGAAATGTTTGATGAAGAAATTGAACAACTTTATTGGTTTGACCCTTTAACAGGG  696
Pste_S   637 CGCATTGAAATGTTTGATGAAGAAATTGAACAACTTTATTGGTTTGACCCTTTAACAGGG  696
Rma      637 CGGATTGAAATGTTTGATGAAGAAATTGAACAACTTTATTGGTTTGATCCTTTAACAGGG  696
Ifos_S   637 CGCATTGAAATGTTTGATGAAGAAATTGAACAACTTTATTGGTTTGATCCTTTAACAGGG  696
Apha_S   637 CGCATTGAAATGTTTGATGAAGAAATTGAACAACTTTATTGGTTTGACCCTTTAACAGGG  696
Bsep_S   637 CGCATTGAATTGTTTGATGAAGAGGTTGAGGCAATTTATTGGTTTGACCCGTTAACAGGC  696
Akaw_S    17   #  #  #  #  #  #  #  #  #  #  #  #  #  #  #  #  #  #  #  #   16
Clau_S   192   R  I  E  M  F  N  E  *  I  E  Q  L  Y  W  F  D  S  L  I  G  211
Pkil_S   150   #  #  #  #  #  #  #  #  #  #  #  #  #  #  #  #  #  #  #  #  149
Psoy_S   150   #  #  #  #  #  #  #  #  #  #  #  #  #  #  #  #  #  #  #  #  149
Vok       63   #  #  #  #  #  #  #  #  #  #  #  #  #  #  #  #  #  #  #  #   62
Cpac_S   213   R  I  E  M  F  D  E  E  I  E  Q  L  Y  W  F  D  P  L  T  G  232
Cfau_S   213   R  I  E  M  F  D  E  E  I  E  Q  L  Y  W  F  D  P  L  T  G  232
Cnau_S   213   R  I  E  M  F  D  E  E  I  E  Q  L  Y  W  F  D  P  L  T  G  232
Pste_S   213   R  I  E  M  F  D  E  E  I  E  Q  L  Y  W  F  D  P  L  T  G  232
Rma      213   R  I  E  M  F  D  E  E  I  E  Q  L  Y  W  F  D  P  L  T  G  232
Ifos_S   213   R  I  E  M  F  D  E  E  I  E  Q  L  Y  W  F  D  P  L  T  G  232
Apha_S   213   R  I  E  M  F  D  E  E  I  E  Q  L  Y  W  F  D  P  L  T  G  232
Bsep_S   213   R  I  E  L  F  D  E  E  V  E  A  I  Y  W  F  D  P  L  T  G  232
```

```
Akaw_S    51 ------------------------------------------------------------   50
Clau_S   639 AAAAAGCTACAATCTTTATAAATAATTATCATTTATTCAAAAATGCACTATATTG-CGCG  697
Pkil_S   459 ------------------------------------------------------------  458
Psoy_S   459 ------------------------------------------------------------  458
Vok      194 ------------------------------------------------------------  193
Cpac_S   697 AAAAAACTAAAATCTTTACAAAGAGTTACTATTTATCCACAAACGCACTACGTT---ACC  753
Cfau_S   697 GAAAAACTAAAATCTTTACAAAGAGTTACCATCTATCCACAAACGCACTACGTT---ACC  753
Cnau_S   697 GGGAAACTAAAATCTTTACAAAGAGTTACCATCTATCCACAAACGCACTACGTT---ACC  753
Pste_S   697 GAAAAACTAAAATTTTTACAAAGAGTTACCATCTATCCACAAACGCACTACGTT---ACC  753
Rma      697 GAAAAACTAAAGTCTTTACAAAGAGTTACCATCTATCCACAAACGCATTATGTT---ACC  753
Ifos_S   697 GAAAAACTAAAGTCTTTGCAAAGAATTACCATCTATCCACAAACACACTACGTT---ACA  753
Apha_S   697 GAAAAACTAAAATCTTTACAAAAAATTACCATTTATCCACAAACGCACTACGTT---ACC  753
Bsep_S   697 GAGAAATATAAACGCTTACAGCGTGTTAGTATTTACCCAAAAACGCATTATGTT---ACG  753
Akaw_S    17   #  #  #  #  #  #  #  #  #  #  #  #  #  #  #  #  #  #  #  #   16
Clau_S   212   K  K  L  Q  S  L  *  I  I  I  I  Y  S  K  M  H  Y  I  #  A  230
Pkil_S   150   #  #  #  #  #  #  #  #  #  #  #  #  #  #  #  #  #  #  #  #  149
Psoy_S   150   #  #  #  #  #  #  #  #  #  #  #  #  #  #  #  #  #  #  #  #  149
Vok       63   #  #  #  #  #  #  #  #  #  #  #  #  #  #  #  #  #  #  #  #   62
Cpac_S   233   K  K  L  K  S  L  Q  R  V  T  I  Y  P  Q  T  H  Y  V  #  T  251
Cfau_S   233   E  K  L  K  S  L  Q  R  V  T  I  Y  P  Q  T  H  Y  V  #  T  251
Cnau_S   233   G  K  L  K  S  L  Q  R  V  T  I  Y  P  Q  T  H  Y  V  #  T  251
Pste_S   233   E  K  L  K  F  L  Q  R  V  T  I  Y  P  Q  T  H  Y  V  #  T  251
Rma      233   E  K  L  K  S  L  Q  R  V  T  I  Y  P  Q  T  H  Y  V  #  T  251
Ifos_S   233   E  K  L  K  S  L  Q  R  I  T  I  Y  P  Q  T  H  Y  V  #  T  251
Apha_S   233   E  K  L  K  S  L  Q  K  I  T  I  Y  P  Q  T  H  Y  V  #  T  251
Bsep_S   233   E  K  Y  K  R  L  Q  R  V  S  I  Y  P  K  T  H  Y  V  #  T  251
```

```
Akaw_S    51 ------------------------------------------------------------   50
Clau_S   698 CTAAAATCTTAAATTTTGAATATATTAAATAATATTAAAGCAGATCTAAAAGATCAAAGA  757
Pkil_S   459 ------------------------------------------------------------  458
Psoy_S   459 ------------------------------------------------------------  458
Vok      194 ------------------------------------------------------------  193
Cpac_S   754 CAAAAATCTAAAATTTTGAATATGTTGGATGATATTAAGGCGGAGCTAAAAGATCGTAGA  813
Cfau_S   754 CCAAAATCTAAAATTTTGAATATGTTGGATGATATTAAAGCAGAGCTAAAAGATTGTAGA  813
Cnau_S   754 CAAAAATCTAAAATTTTGAATATGTTGGATGATATTAAAGCAGAGCTAAAAGATCGTAGA  813
Pste_S   754 CCAAAATCTAAAATTTTGAATATGTTAGATGATATTAAAGCAGAGCTAAAAGATCGTAGA  813
Rma      754 CCAAAATCTAAAATTTTGAATATGTTAGATGATATTAAAGCAGAACTAAAAGATCGAAGA  813
Ifos_S   754 CCAAAATCTAAAATTTTGAATATGTTAGATGATATTAAAGCAGAACTAAAAGATCGCAGA  813
Apha_S   754 CCAAAATCTAAAATTTTGAACATGTTGGATGATATTAAAGTGGAGCTAAAAGATCGTAGA  813
Bsep_S   754 CCAAAATCCAAGATTTTGAATATGTTAGAAGACATTAAGGCCGAGTTAAAAATTAGACAA  813
Akaw_S    17   #  #  #  #  #  #  #  #  #  #  #  #  #  #  #  #  #  #  #  #   16
Clau_S   231   L  K  S  *  I  L  N  I  L  N  N  I  K  A  D  L  K  D  Q  R  250
Pkil_S   150   #  #  #  #  #  #  #  #  #  #  #  #  #  #  #  #  #  #  #  #  149
Psoy_S   150   #  #  #  #  #  #  #  #  #  #  #  #  #  #  #  #  #  #  #  #  149
Vok       63   #  #  #  #  #  #  #  #  #  #  #  #  #  #  #  #  #  #  #  #   62
Cpac_S   252   Q  K  S  K  I  L  N  M  L  D  D  I  K  A  E  L  K  D  R  R  271
Cfau_S   252   P  K  S  K  I  L  N  M  L  D  D  I  K  A  E  L  K  D  C  R  271
Cnau_S   252   Q  K  S  K  I  L  N  M  L  D  D  I  K  A  E  L  K  D  R  R  271
Pste_S   252   P  K  S  K  I  L  N  M  L  D  D  I  K  A  E  L  K  D  R  R  271
Rma      252   P  K  S  K  I  L  N  M  L  D  D  I  K  A  E  L  K  D  R  R  271
Ifos_S   252   P  K  S  K  I  L  N  M  L  D  D  I  K  A  E  L  K  D  R  R  271
Apha_S   252   P  K  S  K  I  L  N  M  L  D  D  I  K  V  E  L  K  D  R  R  271
Bsep_S   252   P  K  S  K  I  L  N  M  L  E  D  I  K  A  E  L  K  I  R  Q  271
```

```
Akaw_S   51 ------------------------------------------------------------  50
Clau_S  758 AATGTTTTGTTGTTTGTTAATAAATTGGTTGAAGAGCAACGTCTAACACAACGTGTACAC 817
Pkil_S  459 ------------------------------------------------------------ 458
Psoy_S  459 ------------------------------------------------------------ 458
Vok     194 ------------------------------------------------------------ 193
Cpac_S  814 AGTGAGTTGCTATCCGTTAATAAATTGGTTGAGGAGCAACGTTTAACACAACGCGTACAC 873
Cfau_S  814 AGTGAGTTGCTATCCGTTAATAAATTGGTTGAGGAGCAACGTTTAACACAACGCGTACAC 873
Cnau_S  814 AGTGAGTTACTATTCGTTAATAAATTGGTTGAGGAGCAACGCTTAACACAACGCGTACAC 873
Pste_S  814 AGTGAGTTGCTATCCGTTAATAAATTGGTTGAGGAGCAACGCTTAACACAACGCGTACAC 873
Rma     814 AGCGAGTTATTATCTGTTAATAAATTAGTTGAGGAGCAGCGTTTAACACAACGCGTACAT 873
Ifos_S  814 AGTGAGTTATTGTCTGTGAATAAATTAGTTGAGGAACAGCGCTTAACACAACGCGTACAT 873
Apha_S  814 AGTGAGTTACTATCTGTTAATAAATTGGTTGAAGAACAACGCTTAACACAACGCGTACAC 873
Bsep_S  814 AAAGAACTGCTATCGGCAAACAAATTGGTGGAGGAGCAACGCTTAACACAAAGGGTACGG 873
Akaw_S   17  #  #  #  #  #  #  #  #  #  #  #  #  #  #  #  #  #  #  #  #   16
Clau_S  251  N  V  L  L  F  V  N  K  L  V  E  E  Q  R  L  T  Q  R  V  H  270
Pkil_S  150  #  #  #  #  #  #  #  #  #  #  #  #  #  #  #  #  #  #  #  #  149
Psoy_S  150  #  #  #  #  #  #  #  #  #  #  #  #  #  #  #  #  #  #  #  #  149
Vok      63  #  #  #  #  #  #  #  #  #  #  #  #  #  #  #  #  #  #  #  #   62
Cpac_S  272  S  E  L  L  S  V  N  K  L  V  E  E  Q  R  L  T  Q  R  V  H  291
Cfau_S  272  S  E  L  L  S  V  N  K  L  V  E  E  Q  R  L  T  Q  R  V  H  291
Cnau_S  272  S  E  L  L  F  V  N  K  L  V  E  E  Q  R  L  T  Q  R  V  H  291
Pste_S  272  S  E  L  L  S  V  N  K  L  V  E  E  Q  R  L  T  Q  R  V  H  291
Rma     272  S  E  L  L  S  V  N  K  L  V  E  E  Q  R  L  T  Q  R  V  H  291
Ifos_S  272  S  E  L  L  S  V  N  K  L  V  E  E  Q  R  L  T  Q  R  V  H  291
Apha_S  272  S  E  L  L  S  V  N  K  L  V  E  E  Q  R  L  T  Q  R  V  H  291
Bsep_S  272  K  E  L  L  S  A  N  K  L  V  E  E  Q  R  L  T  Q  R  V  R  291
```

```
Akaw_S   51 TTGGATATTAGAATGATGCGAAAATTAGGATATTGTACAGATATTGAG---AATTAATCA 107
Clau_S  818 ATGGATATTGAAATGATGCGAGAATTAGGATATTATATAGGTTTTGAGA--AATTACTCA 875
Pkil_S  459 ------------------------------------------------------------ 458
Psoy_S  459 ------------------------------------------------------------ 458
Vok     194 ------------------------------------------------------------ 193
Cpac_S  874 ATGGATATTGAAATGATGCGTGAGTTAGGGTATTGCACAGGTATTGAG---AATTACTCA 930
Cfau_S  874 ATGGATATTGAAATGATGCGTGAGTTAGGGTATTGCACAGGTATTGAG---AATTACTCA 930
Cnau_S  874 ATGGATATTGAAATGATGCGTGAGTTAGGGTATTGCACAGGTATTGAG---AATTATTCA 930
Pste_S  874 ATGGATATTGAAATGATGCGTGAGTTAGGGTATTGCACAGGTATTGAG---AATTACTCA 930
Rma     874 ATGGATATTGAAATGATGCGAGAGTTAGGGTACTGCACAGGTATTGAG---AATTACTCA 930
Ifos_S  874 ATGGATATCGAAATGATGCGCGAGTTAGGATACTGCACAGGTATTGAG---AATTACTCG 930
Apha_S  874 ATGGATATTGAAATGATGCGCGAGTTAGGGTATTGCACAGGTATTGAG---AATTACTCA 930
Bsep_S  874 ATGGATATTGAGATGATGAGCGAATTAGGATATTGCAATGGCATTGAA---AATTATTCG 930
Akaw_S   17  L  D  I  R  M  M  R  K  L  G  Y  C  T  D  I  E  #  N  *  S   35
Clau_S  271  M  D  I  E  M  M  R  E  L  G  Y  Y  I  G  F  E  #  N  Y  S  289
Pkil_S  150  #  #  #  #  #  #  #  #  #  #  #  #  #  #  #  #  #  #  #  #  149
Psoy_S  150  #  #  #  #  #  #  #  #  #  #  #  #  #  #  #  #  #  #  #  #  149
Vok      63  #  #  #  #  #  #  #  #  #  #  #  #  #  #  #  #  #  #  #  #   62
Cpac_S  292  M  D  I  E  M  M  R  E  L  G  Y  C  T  G  I  E  #  N  Y  S  310
Cfau_S  292  M  D  I  E  M  M  R  E  L  G  Y  C  T  G  I  E  #  N  Y  S  310
Cnau_S  292  M  D  I  E  M  M  R  E  L  G  Y  C  T  G  I  E  #  N  Y  S  310
Pste_S  292  M  D  I  E  M  M  R  E  L  G  Y  C  T  G  I  E  #  N  Y  S  310
Rma     292  M  D  I  E  M  M  R  E  L  G  Y  C  T  G  I  E  #  N  Y  S  310
Ifos_S  292  M  D  I  E  M  M  R  E  L  G  Y  C  T  G  I  E  #  N  Y  S  310
Apha_S  292  M  D  I  E  M  M  R  E  L  G  Y  C  T  G  I  E  #  N  Y  S  310
Bsep_S  292  M  D  I  E  M  M  S  E  L  G  Y  C  N  G  I  E  #  N  Y  S  310
```

```
Akaw_S  108 CGTTATTTATCTAGTCAAAAATCTGGGGACCCACCTTCGACTTTATTGGATTATTTGCCA  167
Clau_S  876 CGTTATTTATCTAGTCAAAAACCTGGTAATCTACCTTCGACTTTATTGGATTATTTGCCA  935
Pkil_S  459 ------------------------------------------------------------  458
Psoy_S  459 ------------------------------------------------------------  458
Vok     194 ------------------------------------------------------------  193
Cpac_S  931 CGCTATTTATCCAGTCAAAATCCTGGCGAGTCACCCTCAACATTGTTGGATTATTTGCCA  990
Cfau_S  931 CGCTATTTATCCAGTCAAAATCCTGGCGAGCCACCCTCAACATTGTTGGATTATTTGCCA  990
Cnau_S  931 CGCTATTTATCCAGTCAAAATCCTGGTGAGCCACCCTCAACATTGTTAGATTATTTGCCA  990
Pste_S  931 CGCTATTTATCCAGTCAAAATCCTGGCGAGCCACCTTCAACATTATTGGATTATTTGCCA  990
Rma     931 CGTTATTTATCCAGTCAAAATCCTGGAGATCCACCCTCAACATTGTTGGATTATTTGCCA  990
Ifos_S  931 CGCTATTTATCCAATCAAAACCCTGGCGAGCCACCCTCAACATTGTTGGATTATTTGCCC  990
Apha_S  931 CGCTATTTATCCAGTCAAAATCCTGGCGAGCCACCCTCAACATTGTTGGATTATTTGCCA  990
Bsep_S  931 CGTTATTTATCCAATAGAAAAGCCGGGCAACCGCCACCGACATTGTTGGATTATTTACCT  990
Akaw_S   36   R  Y  L  S  S  Q  K  S  G  D  P  P  S  T  L  L  D  Y  L  P   55
Clau_S  290   R  Y  L  S  S  Q  K  P  G  N  L  P  S  T  L  L  D  Y  L  P  309
Pkil_S  150   #  #  #  #  #  #  #  #  #  #  #  #  #  #  #  #  #  #  #  #  149
Psoy_S  150   #  #  #  #  #  #  #  #  #  #  #  #  #  #  #  #  #  #  #  #  149
Vok      63   #  #  #  #  #  #  #  #  #  #  #  #  #  #  #  #  #  #  #  #   62
Cpac_S  311   R  Y  L  S  S  Q  N  P  G  E  S  P  S  T  L  L  D  Y  L  P  330
Cfau_S  311   R  Y  L  S  S  Q  N  P  G  E  P  P  S  T  L  L  D  Y  L  P  330
Cnau_S  311   R  Y  L  S  S  Q  N  P  G  E  P  P  S  T  L  L  D  Y  L  P  330
Pste_S  311   R  Y  L  S  S  Q  N  P  G  E  P  P  S  T  L  L  D  Y  L  P  330
Rma     311   R  Y  L  S  S  Q  N  P  G  D  P  P  S  T  L  L  D  Y  L  P  330
Ifos_S  311   R  Y  L  S  N  Q  N  P  G  E  P  P  S  T  L  L  D  Y  L  P  330
Apha_S  311   R  Y  L  S  S  Q  N  P  G  E  P  P  S  T  L  L  D  Y  L  P  330
Bsep_S  311   R  Y  L  S  N  R  K  A  G  Q  P  P  P  T  L  L  D  Y  L  P  330

E. coli                                                          L  P
```

```
Akaw_S  168 GGATAATGCATTTGATCTTT-TTATATAAGTCACAAT--ATTACTGTTAGTCAAATCAGT  224
Clau_S  936 GATAATGCATTGATCATT---TTATATGAGTCACAT---ATTACTGTTAATCAAATTAGT  989
Pkil_S  459 ------------------------------------------------------------  458
Psoy_S  459 ------------------------------------------------------------  458
Vok     194 ------------------------------------------------------------  193
Cpac_S  991 AATAATGCATTGATAATT---TTAGATGAATCACAT---GTGACTGTTAGCCAAATTGGT 1044
Cfau_S  991 GATAATGCATTGGTAATT---CTAGATGAATCACAT---GTGACTGTTAGCCAAATTGGT 1044
Cnau_S  991 GATAATGCATTGGTAATT---TTAGATGAATCACAT---GTGACTGTTAGCCAAATTGGT 1044
Pste_S  991 GATAATGCATTGGTAATT---TTAGATGAATCACAT---GTGACTGTTAGCCAAATTGGT 1044
Rma     991 GATAATGCATTGGTAATT---TTAGATGAATCGCAT---GTGACTGTTAGCCAGATTGGT 1044
Ifos_S  991 GATAATGCATTGGTAATT---TTAGATGAATCGCAT---GTGACTGTTAGCCAAATTGGT 1044
Apha_S  991 GATAATGCATTGGTAATT---TTAGATGAATCACAT---GTGACTGTTAGCCAAATTGGT 1044
Bsep_S  991 GAGGATGCCCTGGTGATT---TTGGACGAGTCGCAT---GTGACAGCGAGTCAGATTGGC 1044
Akaw_S   56   G  *  C  I  *  S  #  L  Y  K  S  Q  Y  I  T  V  S  Q  I  S   74
Clau_S  310   D  N  A  L  I  I  #  L  Y  E  S  H  #  I  T  V  N  Q  I  S  327
Pkil_S  150   #  #  #  #  #  #  #  #  #  #  #  #  #  #  #  #  #  #  #  #  149
Psoy_S  150   #  #  #  #  #  #  #  #  #  #  #  #  #  #  #  #  #  #  #  #  149
Vok      63   #  #  #  #  #  #  #  #  #  #  #  #  #  #  #  #  #  #  #  #   62
Cpac_S  331   N  N  A  L  I  I  #  L  D  E  S  H  #  V  T  V  S  Q  I  G  348
Cfau_S  331   D  N  A  L  V  I  #  L  D  E  S  H  #  V  T  V  S  Q  I  G  348
Cnau_S  331   D  N  A  L  V  I  #  L  D  E  S  H  #  V  T  V  S  Q  I  G  348
Pste_S  331   D  N  A  L  V  I  #  L  D  E  S  H  #  V  T  V  S  Q  I  G  348
Rma     331   D  N  A  L  V  I  #  L  D  E  S  H  #  V  T  V  S  Q  I  G  348
Ifos_S  331   D  N  A  L  V  I  #  L  D  E  S  H  #  V  T  V  S  Q  I  G  348
Apha_S  331   D  N  A  L  V  I  #  L  D  E  S  H  #  V  T  V  S  Q  I  G  348
Bsep_S  331   E  D  A  L  V  I  #  L  D  E  S  H  #  V  T  A  S  Q  I  G  348

E. coli       A  D  G  L  L  V     V  D  E  S  H     V  T
                          Motif II
```

```
Akaw_S  225 GGTATGTATAAAGGCGATAGGGCTCGTAAAAAAACGCTTGTTGAATATGATTTCCGTTTA  284
Clau_S  990 GGT------------------------AAAAAAACGCTTGTTGAATTTGGGTTTCGTCTA 1025
Pkil_S  459 ------------------------------------------------------------  458
Psoy_S  459 ------------------------------------------------------------  458
Vok     194 ------------------------------------------------------------  193
Cpac_S 1045 GGCATGTATAAAGGCGATAGGGCGCGTAAAAAAACGCTTGTTGAGTTTGGTTTTCGCCTA 1104
Cfau_S 1045 AGCATGTATAAAGGCGATAGGGCGCGTAAAAAAACGCTTGTTGAGTTTGGTTTTCGCCTA 1104
Cnau_S 1045 GGCATGTATAAAGGCGATAGGGCGCGTAAAAAAACGCTTGTTGAGTTTGGCTTTCGCCTA 1104
Pste_S 1045 GGCATGTATAAAGGCGATAGGGCGCGTAAAAAAACGCTTGTTGAGTTTGGTTTTCGCCTA 1104
Rma    1045 GGCATGTATAAAGGTGATAGGGCGCGTAAAAAAACGCTTGTTGATTTTGGTTTTCGCCTA 1104
Ifos_S 1045 GGTATGTATAAAGGTGATAGGGCACGCAAAAAAACGCTTGTTGAGTTTGGTTTTCGCCTA 1104
Apha_S 1045 GGCATGTATAAAGGTGATAGGGCGCGTAAAAAAACGCTTGTTGAGTTTGGTTTTCGCTTA 1104
Bsep_S 1045 GCAATGTATAAGGGTGATAGGGCGCGTAAAACCACCTTGGTAGAGTTTGGTTTTCGCCTA 1104
Akaw_S   75   G  M  Y  K  G  D  R  A  R  K  K  T  L  V  E  Y  D  F  R  L   94
Clau_S  328   G  #  #  #  #  #  #  #  #  K  K  T  L  V  E  F  G  F  R  L  339
Pkil_S  150   #  #  #  #  #  #  #  #  #  #  #  #  #  #  #  #  #  #  #  #  149
Psoy_S  150   #  #  #  #  #  #  #  #  #  #  #  #  #  #  #  #  #  #  #  #  149
Vok      63   #  #  #  #  #  #  #  #  #  #  #  #  #  #  #  #  #  #  #  #   62
Cpac_S  349   G  M  Y  K  G  D  R  A  R  K  K  T  L  V  E  F  G  F  R  L  368
Cfau_S  349   S  M  Y  K  G  D  R  A  R  K  K  T  L  V  E  F  G  F  R  L  368
Cnau_S  349   G  M  Y  K  G  D  R  A  R  K  K  T  L  V  E  F  G  F  R  L  368
Pste_S  349   G  M  Y  K  G  D  R  A  R  K  K  T  L  V  D  F  G  F  R  L  368
Rma     349   G  M  Y  K  G  D  R  A  R  K  K  T  L  V  D  F  G  F  R  L  368
Ifos_S  349   G  M  Y  K  G  D  R  A  R  K  K  T  L  V  E  F  G  F  R  L  368
Apha_S  349   G  M  Y  K  G  D  R  A  R  K  K  T  L  V  E  F  G  F  R  L  368
Bsep_S  349   A  M  Y  K  G  D  R  A  R  K  T  T  L  V  E  F  G  F  R  L  368


Akaw_S  285 CTAAGTGCTTTAGATAATAGATCACTTCTATTTTCAGAATTTGAAGATAGAATTCATTAA  344
Clau_S 1026 TCAAGTACTTTAGATAATAGGCTACTTAGATTTT-AGAATTTGAAGATAGAGTTCATCAA 1084
Pkil_S  459 ------------------------------------------------------------  458
Psoy_S  459 ------------------------------------------------------------  458
Vok     194 ------------------------------------------------------------  193
Cpac_S 1105 CCAAGTGCTTTGGATAATCGACCGCTTAGGTTTTCAGAATTTGAAGACAGAGTTCGTCAA 1164
Cfau_S 1105 CCAAGTGCTTTGGATAATCGACCGCTTAGATTTTCAGAATTTGAAGACAGAGTTCATCAA 1164
Cnau_S 1105 CCAAGTGCTTTGGATAATCGACCGCTTAGATTTTCAGAATTTGAAGACAGAGTTCATCAA 1164
Pste_S 1105 CCAAGTGCTTTGGATAATCGACCGCTTAGATTTTCAGAATTTGAAGACAGGGTTCATCAA 1164
Rma    1105 CCAAGTGCTTTGGATAATCGGCCACTTAAATTTTCAGAATTTGAAGACAGAGTTCATCAA 1164
Ifos_S 1105 CCAAGTGCGTTGGATAATCGACCACTTAAATTTTCAGAATTTGAAGACAGAGTTCATCAA 1164
Apha_S 1105 CCAAGTGCTTTGGATAATCGACCGCTTAGATTTTCAGAATTTGAAGACAGAGTCCATCAG 1164
Bsep_S 1105 CCATCGGCACTTGATAATCGCCCATTGAGGTTTGAAGAATTTGAGGGAAAAATTCATCAA 1164
Akaw_S   95   L  S  A  L  D  N  R  S  L  L  F  S  E  F  E  D  R  I  H  *  114
Clau_S  340   S  S  T  L  D  N  R  L  L  R  F  #  E  F  E  D  R  V  H  Q  358
Pkil_S  150   #  #  #  #  #  #  #  #  #  #  #  #  #  #  #  #  #  #  #  #  149
Psoy_S  150   #  #  #  #  #  #  #  #  #  #  #  #  #  #  #  #  #  #  #  #  149
Vok      63   #  #  #  #  #  #  #  #  #  #  #  #  #  #  #  #  #  #  #  #   62
Cpac_S  369   P  S  A  L  D  N  R  P  L  R  F  S  E  F  E  D  R  V  R  Q  388
Cfau_S  369   P  S  A  L  D  N  R  P  L  R  F  S  E  F  E  D  R  V  H  Q  388
Cnau_S  369   P  S  A  L  D  N  R  P  L  R  F  S  E  F  E  D  R  V  H  Q  388
Pste_S  369   P  S  A  L  D  N  R  P  L  R  F  S  E  F  E  D  R  V  H  Q  388
Rma     369   P  S  A  L  D  N  R  P  L  K  F  S  E  F  E  D  R  V  H  Q  388
Ifos_S  369   P  S  A  L  D  N  R  P  L  K  F  S  E  F  E  D  R  V  H  Q  388
Apha_S  369   P  S  A  L  D  N  R  P  L  R  F  S  E  F  E  D  R  V  H  Q  388
Bsep_S  369   P  S  A  L  D  N  R  P  L  R  F  E  E  F  E  G  K  I  H  Q  388
```

```
Akaw_S   345 TGTATTTTGGCCTCTG--GCTACGCTTGCACGATATGAATTGGAAGTTTCTAGTATCATT  402
Clau_S  1085 TGTATTTTAGTATCT---TCTATGATTGTATGATATGAACTGTAAGTTTCAGTATCATTG 1141
Pkil_S   459 ---------------------------------------CTGGAAGTTTCTAGTATCATT  479
Psoy_S   459 ---------------------------------------CTGGAAGTTTCTAGTATCATT  479
Vok      194 ------------------------------------------------------------  193
Cpac_S  1165 TGTATTTTGGTATCT---GCCACGCCTGCACAATATGAGTTGGAAGTTTCTAGTGTGATT 1221
Cfau_S  1165 TGTATTTTGGTATCT---GCCACGCCTGCACAATATGAGTTGGAAGTTTCTAGTGTGATT 1221
Cnau_S  1165 TGTATTTTGGTATCT---GCCACGCCTGCACAATATGAGTTGGAAGTTTCTAGCGTGATT 1221
Pste_S  1165 TGTATTTTGGTATCT---GCCACGCCTGCAGAATATGAGCTGGAAGTTTCTAGTGTGATT 1221
Rma     1165 TGTATTTTAGTATCT---GCTACGCCTGCACAATATGAGTTGGAAGTTTCTAGTGTGATT 1221
Ifos_S  1165 TGTATCTTGGTGTCT---GCCACGCCTGCACAATATGAGTTGGAAGTTTCTAGTGTGATT 1221
Apha_S  1165 TGTATTTTGGTATCT---GCCACGCCTGCACAATATGAGTTGGAAGTTTCTAGTGTGATT 1221
Bsep_S  1165 TGTCTATTGGTATCT---GCTACGCCAGCACAGTATGAATTGGATATTTCCAGTAGGATT 1221
Akaw_S   115   C  I  L  A  S  #  A  T  L  A  R  Y  E  L  E  V  S  S  I  I  133
Clau_S   359   C  I  L  V  S  #  S  M  I  V  *  Y  E  L  *  V  S  V  S  L  377
Pkil_S   150   #  #  #  #  #  #  #  #  #  #  #  #  #  L  E  V  S  S  I  I  156
Psoy_S   150   #  #  #  #  #  #  #  #  #  #  #  #  #  L  E  V  S  S  I  I  156
Vok       63   #  #  #  #  #  #  #  #  #  #  #  #  #  #  #  #  #  #  #  #   62
Cpac_S   389   C  I  L  V  S  #  A  T  P  A  Q  Y  E  L  E  V  S  S  V  I  407
Cfau_S   389   C  I  L  V  S  #  A  T  P  A  Q  Y  E  L  E  V  S  S  V  I  407
Cnau_S   389   C  I  L  V  S  #  A  T  P  A  Q  Y  E  L  E  V  S  S  V  I  407
Pste_S   389   C  I  L  V  S  #  A  T  P  A  E  Y  E  L  E  V  S  S  V  I  407
Rma      389   C  I  L  V  S  #  A  T  P  A  Q  Y  E  L  E  V  S  S  V  I  407
Ifos_S   389   C  I  L  V  S  #  A  T  P  A  Q  Y  E  L  E  V  S  S  V  I  407
Apha_S   389   C  I  L  V  S  #  A  T  P  A  Q  Y  E  L  E  V  S  S  V  I  407
Bsep_S   389   C  L  L  V  S  #  A  T  P  A  Q  Y  E  L  D  I  S  S  R  I  407

E. coli           I  Y  V  S     A  T  P  G  N  Y  E  L  E  K
                             Motif III
```

```
Akaw_S   403 GCTGAGCAAGTGATAAGACCAACAGGATTATTGGATCCTGAAATTAATATTTGCTCTACG  462
Clau_S  1142 TTAAGCAAAGTGGTAAGAGCAACAGGATTATTAGATTCTGAAATTGATATTCGTTCTTTG 1201
Pkil_S   480 GCTGAGTAAGTGGTAAGA------------------------------------------  497
Psoy_S   480 GCTGAGTAAGTGGTAAGA------------------------------------------  497
Vok      194 ------------------------------------------------------------  193
Cpac_S  1222 GCTGAACAAGTAGTAAGACCAACAGGATTATTAGACCCTGAGATTGACATTCGTCCTGTG 1281
Cfau_S  1222 GCTGAACAAGTAGTAAGACCGACAGGATTGTTAGACCCTGAGATTGACATTCGCCCTGTG 1281
Cnau_S  1222 GCTGAACAAGTAGTAAGACCAACAGGATTGTTAGACCCTGAGATTGACATTCGCCCTGTG 1281
Pste_S  1222 GCTGAACAAGTAGTAAGACCAACAGGATTGTTAGACCCTGAGATTGACATTCGCCCTGTG 1281
Rma     1222 GTTGAACAAGTAGTAAGGCCAACAGGATTGTTAGACCCTGAGATTGATATTCGCCCTGTG 1281
Ifos_S  1222 GCTGAACAAGTAGTAAGGCCAACAGGATTGTTAGACCCTGAGATTGACATTCGACCTGTG 1281
Apha_S  1222 GCTGAACAAGTAGTAAGACCAACAGGATTATTAGACCCTGAGATTGACATTCGTCCTGTG 1281
Bsep_S  1222 GTTGAGCAAGTGGTACGCCCGACGGGTTTGTTAGACCCTGAAATTGAAGTGCGCCCCGTG 1281
Akaw_S   134   A  E  Q  V  I  R  P  T  G  L  L  D  P  E  I  N  I  C  S  T  153
Clau_S   378   L  S  K  V  V  R  A  T  G  L  L  D  S  E  I  D  I  R  S  L  397
Pkil_S   157   A  E  *  V  V  R  #  #  #  #  #  #  #  #  #  #  #  #  #  #  162
Psoy_S   157   A  E  *  V  V  R  #  #  #  #  #  #  #  #  #  #  #  #  #  #  162
Vok       63   #  #  #  #  #  #  #  #  #  #  #  #  #  #  #  #  #  #  #  #   62
Cpac_S   408   A  E  Q  V  V  R  P  T  G  L  L  D  P  E  I  D  I  R  P  V  427
Cfau_S   408   A  E  Q  V  V  R  P  T  G  L  L  D  P  E  I  D  I  R  P  V  427
Cnau_S   408   A  E  Q  V  V  R  P  T  G  L  L  D  P  E  I  D  I  R  P  V  427
Pste_S   408   A  E  Q  V  V  R  P  T  G  L  L  D  P  E  I  D  I  R  P  V  427
Rma      408   V  E  Q  V  V  R  P  T  G  L  L  D  P  E  I  D  I  R  P  V  427
Ifos_S   408   A  E  Q  V  V  R  P  T  G  L  L  D  P  E  I  D  I  R  P  V  427
Apha_S   408   A  E  Q  V  V  R  P  T  G  L  L  D  P  E  I  D  I  R  P  V  427
Bsep_S   408   V  E  Q  V  V  R  P  T  G  L  L  D  P  E  I  E  V  R  P  V  427
```

```
Akaw_S  463 AATGCAAAGGTAGATGATTTACTTAGTGAAATACGAACACGTGTTAAAGTGGATAAAAGT  522
Clau_S 1202 GATACACAGGTAGATGATTTACTTAGTGAAATACGAACACGTGTTAAA------------ 1249
Pkil_S  498 ------------------------------------------------------------  497
Psoy_S  498 ------------------------------------------------------------  497
Vok     194 ------------------------------------------------------------  193
Cpac_S 1282 GATACGCAGGTGGATGATTTACTTAGTGAAATACGAATGCGTGTTAAAGCGAATGAGCGT 1341
Cfau_S 1282 GATACGCAGGTGGATGATTTACTTAGTGAAATACGAATGCGCGTTAAAGCGAATGAGCGT 1341
Cnau_S 1282 GATACACAGGTGGATGATTTACTTAGTGAAATACGAATGCGTGTTAAAGCGAATGAGCGT 1341
Pste_S 1282 GATACGCAGGTGGATGATTTACTTAGTGAAATACGAATGCACGTTAAAGCGAATGAGCGT 1341
Rma    1282 GATACACAGGTGGATGATTTGCTGAGTGAAATACGAATGCGTGTTAAAGCAGGTGAACGA 1341
Ifos_S 1282 GATACTCAGGTGGATGATTTACTGAGTGAAATACGTATGCGCGTTAAAGCGGGCGAACGC 1341
Apha_S 1282 GATACGCAGGTGGATGATCTACTTAGTGAGATACGAATGCGTGTTAAAGTGAATGAGCGT 1341
Bsep_S 1282 GAAACGCAAGTCGATGATTTATTGAGTGAAATACACAAACGAGTTGAGATGGGGGATAGG 1341
Akaw_S  154  N  A  K  V  D  D  L  L  S  E  I  R  T  R  V  K  V  D  K  S   173
Clau_S  398  D  T  Q  V  D  D  L  L  S  E  I  R  T  R  V  K  #  #  #  #   413
Pkil_S  163  #  #  #  #  #  #  #  #  #  #  #  #  #  #  #  #  #  #  #  #   162
Psoy_S  163  #  #  #  #  #  #  #  #  #  #  #  #  #  #  #  #  #  #  #  #   162
Vok      63  #  #  #  #  #  #  #  #  #  #  #  #  #  #  #  #  #  #  #  #   62
Cpac_S  428  D  T  Q  V  D  D  L  L  S  E  I  R  M  R  V  K  A  N  E  R   447
Cfau_S  428  D  T  Q  V  D  D  L  L  S  E  I  R  M  R  V  K  A  N  E  R   447
Cnau_S  428  D  T  Q  V  D  D  L  L  S  E  I  R  M  R  V  K  A  N  E  R   447
Pste_S  428  D  T  Q  V  D  D  L  L  S  E  I  R  M  H  V  K  A  N  E  R   447
Rma     428  D  T  Q  V  D  D  L  L  S  E  I  R  M  R  V  K  A  G  E  R   447
Ifos_S  428  D  T  Q  V  D  D  L  L  S  E  I  R  M  R  V  K  A  G  E  R   447
Apha_S  428  D  T  Q  V  D  D  L  L  S  E  I  R  M  R  V  K  V  N  E  R   447
Bsep_S  428  E  T  Q  V  D  D  L  L  S  E  I  H  K  R  V  E  M  G  D  R   447

E. coli                                                     E  R
```

helicase superfamily c-terminal domain (429-549)

```
Akaw_S  523 GTATTGGTTACAACTTTGACTAAAAAAATAAGTGAGTAATTAAGTGATTATTTAAATGAC  582
Clau_S 1250 ------------------------------------------------------------ 1249
Pkil_S  498 ------------------------------------------------------------  497
Psoy_S  498 ------------------------------------------------------------  497
Vok     194 ------------------------------------------------------------  193
Cpac_S 1342 GTATTAGTGACAACTTTGACTAAAAAAATGAGTGAGCAGTTAAGTGATTATTTAAACGAC 1401
Cfau_S 1342 GTATTGGTGACAACTTTGACTAAAAAAATGAGTGAACAGTTAAGTGATTATTTAAACGAC 1401
Cnau_S 1342 GTACTGGTGACAACTTTGACTAAAAAAATGAGTGAGCAGTTAAGTGATTATTTAAACGAC 1401
Pste_S 1342 ATATTGGTGACAACTTTAACTAAAAAAATGAGTGAGCAGTTAAGTGATTATTTAAACGAC 1401
Rma    1342 GTATTGGTGACAACTTTGACTAAAAAAATGAGTGAGCAATTAAGTGATTATTTAAACGAC 1401
Ifos_S 1342 GTATTGGTGACAACTTTGACCAAAAAAATGAGTGAGCAGTTAAGTGATTACTTAAACGAC 1401
Apha_S 1342 GTATTGGTGACAACTTTGACTAAAAAAATGAGTGAGCGGTTAAGTGATTATCTAAACGAC 1401
Bsep_S 1342 GTGTTGGTTACTACTTTAACCAAGCGAATGAGCGAACAATTAAGTGATTATTTAAGTGAG 1401
Akaw_S  174  V  L  V  T  T  L  T  K  K  I  S  E  *  L  S  D  Y  L  N  D   193
Clau_S  414  #  #  #  #  #  #  #  #  #  #  #  #  #  #  #  #  #  #  #  #   413
Pkil_S  163  #  #  #  #  #  #  #  #  #  #  #  #  #  #  #  #  #  #  #  #   162
Psoy_S  163  #  #  #  #  #  #  #  #  #  #  #  #  #  #  #  #  #  #  #  #   162
Vok      63  #  #  #  #  #  #  #  #  #  #  #  #  #  #  #  #  #  #  #  #   62
Cpac_S  448  V  L  V  T  T  L  T  K  K  M  S  E  Q  L  S  D  Y  L  N  D   467
Cfau_S  448  V  L  V  T  T  L  T  K  K  M  S  E  Q  L  S  D  Y  L  N  D   467
Cnau_S  448  V  L  V  T  T  L  T  K  K  M  S  E  Q  L  S  D  Y  L  N  D   467
Pste_S  448  I  L  V  T  T  L  T  K  K  M  S  E  Q  L  S  D  Y  L  N  D   467
Rma     448  V  L  V  T  T  L  T  K  K  M  S  E  Q  L  S  D  Y  L  N  D   467
Ifos_S  448  V  L  V  T  T  L  T  K  K  M  S  E  Q  L  S  D  Y  L  N  D   467
Apha_S  448  V  L  V  T  T  L  T  K  K  M  S  E  R  L  S  D  Y  L  N  D   467
Bsep_S  448  V  L  V  T  T  L  T  K  R  M  S  E  Q  L  S  D  Y  L  S  E   467

E. coli      V  L  V  T  T  I  T  K  R  M  A  E  D  L  T  E  Y  L  E  E
                                   Motif IV
```

helicase superfamily c-terminal domain (429-549)

```
Akaw_S   583 TATTATGTTAAAGTTTGGTTATTTATAT--TCTGATATCGATGCGGCTAAAAGCGTGGAG  640
Clau_S  1250 ------------GTTTGCTATTTACAT---TCTGATATTGATACGGTTAAAAGAGTGGAA 1294
Pkil_S   498 -------------CTAATTTATTAGAT---CCTAAAATTGATACGGTTAAACGAGTGGAA  541
Psoy_S   498 -------------CTAATTTATTAGAT---CCTAAAATTGATACGGTTAAACGAGTGGAA  541
Vok      194 ------------------------------------------------------------  193
Cpac_S  1402 CATCATGTTAAAGTTCGTTACCTACAT---TCTGATATTGATACGGTTGAAAGGGTGGAA 1458
Cfau_S  1402 CATCATGTTAAAGTTCGTTACCTACAT---TCTGATATTGATACGGTTGAAAGGGTGGAA 1458
Cnau_S  1402 CATCATGTTAAAGTTCGTTACCTACAT---TCTGATATTGATACAGTTGAAAGGGTGGAA 1458
Pste_S  1402 CATCATGTTAAAGTTCGTTACCTACAT---TCTGATATTGATACGGTTGAAAGGGTGGAA 1458
Rma     1402 CATCATGTTAAAGTTCGTTACTTACAT---TCTGATATTGATACAATTGAAAGGGTGGAA 1458
Ifos_S  1402 CATCATGTTAAAGTTCGTTACTTACAT---TCTGATATTGATACGGTTGAAAGGGTGGAA 1458
Apha_S  1402 CATCATGTTAAAGTTCGTTACTTGCAT---TCTGATATTGATACGGTTGAAAGGGTGGAA 1458
Bsep_S  1402 CATAATGTTAAAGTTCGTTATTTACAC---TCTGATATTGATACGGTTGAGCGAGTAGAG 1458
Akaw_S   194   Y  Y  V  K  V  W  L  F  I  #  S  D  I  D  A  A  K  S  V  E   212
Clau_S   414   #  #  #  #  V  C  Y  L  H  #  S  D  I  D  T  V  K  R  V  E   428
Pkil_S   163   #  #  #  #  #  N  L  L  D  #  P  K  I  D  T  V  K  R  V  E   176
Psoy_S   163   #  #  #  #  #  N  L  L  D  #  P  K  I  D  T  V  K  R  V  E   176
Vok       63   #  #  #  #  #  #  #  #  #  #  #  #  #  #  #  #  #  #  #  #    62
Cpac_S   468   H  H  V  K  V  R  Y  L  H  #  S  D  I  D  T  V  E  R  V  E   486
Cfau_S   468   H  H  V  K  V  R  Y  L  H  #  S  D  I  D  T  V  E  R  V  E   486
Cnau_S   468   H  H  V  K  V  R  Y  L  H  #  S  D  I  D  T  V  E  R  V  E   486
Pste_S   468   H  H  V  K  V  R  Y  L  H  #  S  D  I  D  T  V  E  R  V  E   486
Rma      468   H  H  V  K  V  R  Y  L  H  #  S  D  I  D  T  I  E  R  V  E   486
Ifos_S   468   H  H  V  K  V  R  Y  L  H  #  S  D  I  D  T  V  E  R  V  E   486
Apha_S   468   H  H  V  K  V  R  Y  L  H  #  S  D  I  D  T  V  E  R  V  E   486
Bsep_S   468   H  N  V  K  V  R  Y  L  H  #  S  D  I  D  T  V  E  R  V  E   486
```

helicase superfamily c-terminal domain (429-549)

```
Akaw_S   641 ATTATTCGTGATTTTTGGTCAGATGTTTTTGATGTACTTGTTGGTATCAATTTGATACGT  700
Clau_S  1295 ATTATTCGTGATTTTAGTTTAGGTGTTTTTAATGTACTTATTGGTATTAATCTGTTACAT 1354
Pkil_S   542 ATTATTCGTGATTTTAGCTTAGGTGTTTTTGATGTACTTATTGGTACCAATCTGGTACGT  601
Psoy_S   542 ATTATTCGTGATTTTAGCTTAGGTGTTTTTGATGTACTTATTGGTACCAATCTGGTACGT  601
Vok      194 ------------------------------------------------------------  193
Cpac_S  1459 ATTATTCGTGATTTGCGCTTAGGCATATTTGATGTACTTGTTGGTATCAATCTATTACGT 1518
Cfau_S  1459 ATTATTCGTGATTTGCGCTTAGGTATGTTTGATGTGCTTGTTGGTATCAACCTATTACGC 1518
Cnau_S  1459 ATTATTCGTGATTTGCGCTTAGGCGTGTTTGATGTGCTTGTTGGTATCAATCTATTACGC 1518
Pste_S  1459 ATTATTCGTGATTTGCGCTTAGGCGTGTTTGATGTGCTTGTTGGTATCAACCTATTACGC 1518
Rma     1459 ATTATTCGTGATTTGCGCTTAGGTATATTTGATGTGCTAGTTGGTATTAACCTATTACGT 1518
Ifos_S  1459 ATTATTCGTGATTTGCGCTTAGGTATATTTGATGTTCTGGTTGGTATTAATCTATTACGT 1518
Apha_S  1459 ATTATTCGTGATTTGCGCTTAGGCGTATTTGATGTGCTTGTTGGTATCAACCTATTACGT 1518
Bsep_S  1459 ATTATTCGCGATTTGCGTTTGGGTATTTTTGATGTTTTAGTGGGTATTAATTTACTTAGA 1518
Akaw_S   213   I  I  R  D  F  W  S  D  V  F  D  V  L  V  G  I  N  L  I  R   232
Clau_S   429   I  I  R  D  F  S  L  G  V  F  N  V  L  I  G  I  N  L  L  H   448
Pkil_S   177   I  I  R  D  F  S  L  G  V  F  D  V  L  I  G  T  N  L  V  R   196
Psoy_S   177   I  I  R  D  F  S  L  G  V  F  D  V  L  I  G  T  N  L  V  R   196
Vok       63   #  #  #  #  #  #  #  #  #  #  #  #  #  #  #  #  #  #  #  #    62
Cpac_S   487   I  I  R  D  L  R  L  G  I  F  D  V  L  V  G  I  N  L  L  R   506
Cfau_S   487   I  I  R  D  L  R  L  G  M  F  D  V  L  V  G  I  N  L  L  R   506
Cnau_S   487   I  I  R  D  L  R  L  G  V  F  D  V  L  V  G  I  N  L  L  R   506
Pste_S   487   I  I  R  D  L  R  L  G  V  F  D  V  L  V  G  I  N  L  L  R   506
Rma      487   I  I  R  D  L  R  L  G  I  F  D  V  L  V  G  I  N  L  L  R   506
Ifos_S   487   I  I  R  D  L  R  L  G  I  F  D  V  L  V  G  I  N  L  L  R   506
Apha_S   487   I  I  R  D  L  R  L  G  V  F  D  V  L  V  G  I  N  L  L  R   506
Bsep_S   487   I  I  R  D  L  R  L  G  I  F  D  V  L  V  G  I  N  L  L  R   506

E. coli                    L  R  L  G  E  F  D  V  L  V  G  I  N  L  L  R
                                    Motif V
```

helicase superfamily c-terminal domain (429-549)

```
Akaw_S   701 GAAGGTTTAAATATTCCAGAG------GTATCTTTAGTGGGACT-ATTTTATG-GATGCA  752
Clau_S  1355 AAAGGTTTGGATATTCGAGAGGCA--TCTTTCTTTAGTGGCT---ATTTTA---GATGCG 1406
Pkil_S   602 GAAGGTTCGAATATTCTAGAGACA------TCTTTAGTGACT----TTTTA---GATGCA  648
Psoy_S   602 GAAGGTTCGAATATTCTAGAGACA------TCTTTAGTGACT----TTTTA---GATGCA  648
Vok      194 ------------------------------------------------------------  193
Cpac_S  1519 GAAGGCTTGGATATTCCCGAA------GTGTCTTTGGTGGCT---ATTTTA---GATGCA 1566
Cfau_S  1519 GAAGGTTTGGATATTCCCGAA------GTGTCTTTAGTGGCT---ATTTTA---GATGCA 1566
Cnau_S  1519 GAAGGTTTGGATATTCCCGAA------GTGTCTTTGGTGGCT---ATTTTA---GATGCA 1566
Pste_S  1519 GAAGGTTTGGATATTCCTGAA------GTGTCTTTGGTGGCT---ATTTTA---GATGCA 1566
Rma     1519 GAAGGTTTGGATATTCCTGAG------GTATCTTTGGTGGCT---ATTTTA---GATGCA 1566
Ifos_S  1519 GAAGGTTTGGATATTCCTGAG------GTGTCTTTGGTGGCT---ATTTTA---GATGCG 1566
Apha_S  1519 GAAGGCTTGGATATTCCCGAA------GTGTCTTTGGTGGCT---ATTTTA---GATGCA 1566
Bsep_S  1519 GAGGGCTTAGACATTCCAGAA------GTCTCGCTTGTTGCT---ATTTTA---GATGCT 1566
Akaw_S   233   E  G  L  N  I  P  E  #  #  V  S  L  V  G  #  #  L  #  D  A  247
Clau_S   449   K  G  L  D  I  R  E  A  #  L  S  L  V  A  #  I  L  #  D  A  465
Pkil_S   197   E  G  S  N  I  L  E  T  #  #  S  L  V  T  #  #  L  #  D  A  211
Psoy_S   197   E  G  S  N  I  L  E  T  #  #  S  L  V  T  #  #  L  #  D  A  211
Vok       63   #  #  #  #  #  #  #  #  #  #  #  #  #  #  #  #  #  #  #  #   62
Cpac_S   507   E  G  L  D  I  P  E  #  #  V  S  L  V  A  #  I  L  #  D  A  522
Cfau_S   507   E  G  L  D  I  P  E  #  #  V  S  L  V  A  #  I  L  #  D  A  522
Cnau_S   507   E  G  L  D  I  P  E  #  #  V  S  L  V  A  #  I  L  #  D  A  522
Pste_S   507   E  G  L  D  I  P  E  #  #  V  S  L  V  A  #  I  L  #  D  A  522
Rma      507   E  G  L  D  I  P  E  #  #  V  S  L  V  A  #  I  L  #  D  A  522
Ifos_S   507   E  G  L  D  I  P  E  #  #  V  S  L  V  A  #  I  L  #  D  A  522
Apha_S   507   E  G  L  D  I  P  E  #  #  V  S  L  V  A  #  I  L  #  D  A  522
Bsep_S   507   E  G  L  D  I  P  E  #  #  V  S  L  V  A  #  I  L  #  D  A  522

E. coli        E  G  L  D  M  P
```

helicase superfamily c-terminal domain (429-549)

```
Akaw_S   753 GATAAAGAAGGATTTTTACGCTCTGAATACTCGTTAATTCAAACCATGGGACGGAAAAGG  812
Clau_S  1407 TATAAAGAAGT-TTTTTACGCTCTGAACTCTCGATAATTCAATCTATAGGATGAA----- 1460
Pkil_S   649 GATAAAGAAGGTTTTTTACGCTCTGAACGCTTGCTAATTTAACCCATGGGA-CAA-GCCT  706
Psoy_S   649 GATAAAGAAGGTTTTTTACGCTCTGAACGCTTGCTAATTTAACCCATGGGA-CAA-GCCC  706
Vok      194 ------------------------------------------------------------  193
Cpac_S  1567 GATAAAGAAGGATTTTTACGCTCTGAACGCTCGCTGATTCAAACCATGGGGCGAG----- 1621
Cfau_S  1567 GATAAAGAAGGATTTTTACGTTCTGAACGCTCTCTGATTCAAACCATGGGGCGAG----- 1621
Cnau_S  1567 GATAAAGAAGGATTTTTACGCTCTGAACGTTCGCTAATTCAAACCATGGGGCGAG----- 1621
Pste_S  1567 GATAAAGAAGGATTTTTACGCTCTGAACGCTCGCTGATTCAAACCATGGGGCGAG----- 1621
Rma     1567 GATAAAGAAGGTTTTTTACGTTCTGAACGCTCGTTGATTCAAACCATGGGGCGAG----- 1621
Ifos_S  1567 GATAAAGAAGGTTTTTTACGCTCTGAACGCTCATTAATTCAAACCATGGGGCGAG----- 1621
Apha_S  1567 GATAAAGAAGGGTTTTTACGCTCTGAACGCTCGCTGATTCAAACCATGGGGCGAG----- 1621
Bsep_S  1567 GATAAAGAAGGTTTTTTGCGATCCGAACGCGCTTTAATTCAAACCATGGGTAGGG----- 1621
Akaw_S   248   D  K  E  G  F  L  R  S  E  Y  S  L  I  Q  T  M  G  R  K  R  267
Clau_S   466   Y  K  E  #  F  L  R  S  E  L  S  I  I  Q  S  I  G  *  #  #  482
Pkil_S   212   D  K  E  G  F  L  R  S  E  R  L  L  I  *  P  M  G  #  #  P  229
Psoy_S   212   D  K  E  G  F  L  R  S  E  R  L  L  I  *  P  M  G  #  #  P  229
Vok       63   #  #  #  #  #  #  #  #  #  #  #  #  #  #  #  #  #  #  #  #   62
Cpac_S   523   D  K  E  G  F  L  R  S  E  R  S  L  I  Q  T  M  G  R  A  #  541
Cfau_S   523   D  K  E  G  F  L  R  S  E  R  S  L  I  Q  T  M  G  R  #  #  540
Cnau_S   523   D  K  E  G  F  L  R  S  E  R  S  L  I  Q  T  M  G  R  #  #  540
Pste_S   523   D  K  E  G  F  L  R  S  E  R  S  L  I  Q  T  M  G  R  #  #  540
Rma      523   D  K  E  G  F  L  R  S  E  R  S  L  I  Q  T  M  G  R  #  #  540
Ifos_S   523   D  K  E  G  F  L  R  S  E  R  S  L  I  Q  T  M  G  R  #  #  540
Apha_S   523   D  K  E  G  F  L  R  S  E  R  S  L  I  Q  T  M  G  R  #  #  540
Bsep_S   523   D  K  E  G  F  L  R  S  E  R  A  L  I  Q  T  M  G  R  #  #  540

E. coli                       L  R  S  E  R  S  L  I  Q  T  I  G  R
                              Motif VI.
```

helicase superfamily c-terminal domain (429-549)

```
Akaw_S   813 CT-GCTAGAAATATAAATGGACACGTAATTTTATACGC-GCTACCAACATAGCCAAATCA  870
Clau_S  1461 --GGCTAGATATATAAATGTGCACATAATTTTACAT---GTTGCCAGCATAATTACATCG 1515
Pkil_S   707 CCGGCTAG-TATAAGAATTGGTACGTAATTTTATAT---GTTACCTACATAACCAAATCG  762
Psoy_S   707 CCGGCTAG-TATAAGAATTGGTACGTAATTTTATAT---GTTACCTACATAACCAAATCG  762
Vok      194 ------------------------------------------------------------  193
Cpac_S  1622 -CGGCTAGGAATATAAATGGACACGTAATTTTATAC---GCTGCTCGCATAACCAAATCA 1677
Cfau_S  1622 -CGGCTAGGAATATAAATGGGCACGTAATTTTATAC---GCTGCTCGCATAACCAAATCG 1677
Cnau_S  1622 -CGGCTAGAAATATAAATGGGCACGTAATTTTATAC---GCTGCTCGCATAACCAAATCG 1677
Pste_S  1622 -CGGCTAGAAATATAAATGGGCACGTAATTTTATAC---GCTGCTCGCATAACCAAATCG 1677
Rma     1622 -CAGCTAGAAATATAAATGGGCACGTCATTTTATAT---GCTGCTCGCATAACTAAATCG 1677
Ifos_S  1622 -CAGCTAGAAATATAAATGGACACGTCATTTTATAC---GTTGCTCGCATAACCAAATCG 1677
Apha_S  1622 -CGGCTAGAAATATAAATGGGCACGTAATTTTATAC---GCTGCCCGCATAACCAAATCG 1677
Bsep_S  1622 -CGGCTAGAAATATCAATGGGCATGTGATTTTATAC---GCCGATAAAATTACCAAATCC 1677
Akaw_S   268   #  A  R  N  I  N  G  H  V  I  L  Y  #  A  T  N  I  A  K  S  285
Clau_S   483   #  A  R  Y  I  N  V  H  I  I  L  H  #  V  A  S  I  I  T  S  500
Pkil_S   230   P  A  #  Y  K  N  W  Y  V  I  L  Y  #  V  T  Y  I  T  K  S  247
Psoy_S   230   P  A  #  Y  K  N  W  Y  V  I  L  Y  #  V  T  Y  I  T  K  S  247
Vok       63   #  #  #  #  #  #  #  #  #  #  #  #  #  #  #  #  #  #  #  #   62
Cpac_S   542   #  A  R  N  I  N  G  H  V  I  L  Y  #  A  A  R  I  T  K  S  559
Cfau_S   541   #  A  R  N  I  N  G  H  V  I  L  Y  #  A  A  R  I  T  K  S  558
Cnau_S   541   #  A  R  N  I  N  G  H  V  I  L  Y  #  A  A  R  I  T  K  S  558
Pste_S   541   #  A  R  N  I  N  G  H  V  I  L  Y  #  A  A  R  I  T  K  S  558
Rma      541   #  A  R  N  I  N  G  H  V  I  L  Y  #  A  A  R  I  T  K  S  558
Ifos_S   541   #  A  R  N  I  N  G  H  V  I  L  Y  #  V  A  R  I  T  K  S  558
Apha_S   541   #  A  R  N  I  N  G  H  V  I  L  Y  #  A  A  R  I  T  K  S  558
Bsep_S   541   #  A  R  N  I  N  G  H  V  I  L  Y  #  A  D  K  I  T  K  S  558

E. coli        A  A  R  N
```

helicase superfamily c-terminal domain (429-549) UvrB domain (550-591)

```
Akaw_S   871 ATGCAAAAA---G----AAATGAA--TAAAAT---------------------------  893
Clau_S  1516 ATGCAAAGA---GCAATGAATGAAATTAAAAGAGGT------CGGATTAAATAGGAGGTT 1566
Pkil_S   763 ATGTAAAGAT--CAAATAAATGAAACTAAAAGACGTTCTC--CGGGTTAAACAGGAGACT  818
Psoy_S   763 ATGTAAAGAT--CAAATAAATGAAACTAAAAGACGTTCTC--CGGGTTAAACAGGAGACT  818
Vok      194 ------------------------------------------------------------  193
Cpac_S  1678 ATGCAAAAG---GCGATGGATGAAACCAAAAGACGT------CGGGTTAAGCAGCAGGCC 1728
Cfau_S  1678 ATGCAAAAG---GCGATGGATGAAACCAAAAGACGT------CGGGTTAAGCAGCAGGCC 1728
Cnau_S  1678 ATGCAAAAG---GCGATGGATGAAACCAAAAGACGT------CGGGTTAAGCAGCAGGCC 1728
Pste_S  1678 ATGCAAAAG---GCGATGGATGAAACCAAAAGACGT------CGGGTTAAGCAGCAGGCC 1728
Rma     1678 ATGCAAAAA---GCAATGGATGAAACCAAAAGACGT------CGAATTAAACAGCAAGCT 1728
Ifos_S  1678 ATGCAAAAA---GCGATGGATGAAACCAAAAGACGT------CGGATTAAACAGCAAGCT 1728
Apha_S  1678 ATGCAAAAG---GCGATGGATGAAACCAAAAGACGT------CGGGTTAAGCAGCAGGAC 1728
Bsep_S  1678 ATGCAAAAA---GCAATGGATGTTACTACTGATCGC------CGTGAAAAACAGCGAGCG 1728
Akaw_S   286   M  Q  K  #  #  #  N  E  #  K  #  #  #  #  #  #  #  #  #  #  291
Clau_S   501   M  Q  R  #  A  M  N  E  I  K  R  G  #  #  R  I  K  *  E  V  517
Pkil_S   248   M  *  R  #  Q  I  N  E  T  K  R  R  S  #  R  V  K  Q  E  T  265
Psoy_S   248   M  *  R  #  Q  I  N  E  T  K  R  R  S  #  R  V  K  Q  E  T  265
Vok       63   #  #  #  #  #  #  #  #  #  #  #  #  #  #  #  #  #  #  #  #   62
Cpac_S   560   M  Q  K  #  A  M  D  E  T  K  R  R  #  #  R  V  K  Q  Q  A  576
Cfau_S   559   M  Q  K  #  A  M  D  E  T  K  R  R  #  #  R  V  K  Q  Q  A  575
Cnau_S   559   M  Q  K  #  A  M  D  E  T  K  R  R  #  #  R  V  K  Q  Q  A  575
Pste_S   559   M  Q  K  #  A  M  D  E  T  K  R  R  #  #  R  V  K  Q  Q  A  575
Rma      559   M  Q  K  #  A  M  D  E  T  K  R  R  #  #  R  I  K  Q  Q  A  575
Ifos_S   559   M  Q  K  #  A  M  D  E  T  K  R  R  #  #  R  I  K  Q  Q  A  575
Apha_S   559   M  Q  K  #  A  M  D  E  T  K  R  R  #  #  R  V  K  Q  Q  D  575
Bsep_S   559   M  Q  K  #  A  M  D  V  T  T  D  R  #  #  R  E  K  Q  R  A  575
```

UvrB domain (550-591)

```
Akaw_S  894 --------------------------TAAAAGTATTACTAAACTTATTATTAATATTCTT  927
Clau_S 1567 TTTAATTTAAAAAATGACATCACACCTAAAAGTATTTTCAAGCCTATTATCAATATTCTT 1626
Pkil_S  819 TATAATTTAAAAAATGATATCACGCCTAAAAGTATTACTAAACCTATTATTAATATTCTT  878
Psoy_S  819 TATAATTTAAAAAATGATATCACGCCTAAAAGTATTACTAAACCTATTATTAATATTCTT  878
Vok     194 ---------------------------------------AAACCTATTATCAATACTCTT  214
Cpac_S 1729 TATAATCTGAAAAACAATATTACACCCAAAGGTATTGCCAAACCTATTATCAATATCCTT 1788
Cfau_S 1729 TATAATCTGAAAAACAATATCACACCCAAAGGTATTACTAAACCTATTATCAATATCCTT 1788
Cnau_S 1729 TATAATCTGAGAAACAATATCACACCCAAAGGTATTGCTAAACCTATTATCAATATTCTT 1788
Pste_S 1729 TATAATCTGAAAAACAATATCACACCAAAAGGTATTGCTAAACCTATTATCAATATCCTT 1788
Rma    1729 TATAATCTGAAAAACAACATCACACCTGAAGGTATTGCTAAGCCTATTATCAATATCCTT 1788
Ifos_S 1729 TATAATTTGAAAAACAACATCACTCCTAAAGGCATTGATAAGCCTATTATTAATATCCTT 1788
Apha_S 1729 TATAATCTAAAGAACAACATCACACCCAAAGGTATTGCCAAACCTATTATCAATATCCTT 1788
Bsep_S 1729 CACAATAAAGCTAACGGCATCACCCCAAAAAGTGTATCCCGCCCAATCGTCAATATTTTA 1788
Akaw_S  292   #  #  #  #  #  #  #  #  #  K  S  I  T  K  L  I  I  N  I  L  302
Clau_S  518   F  N  L  K  N  D  I  T  P  K  S  I  F  K  P  I  I  N  I  L  537
Pkil_S  266   Y  N  L  K  N  D  I  T  P  K  S  I  T  K  P  I  I  N  I  L  285
Psoy_S  266   Y  N  L  K  N  D  I  T  P  K  S  I  T  K  P  I  I  N  I  L  285
Vok      63   #  #  #  #  #  #  #  #  #  #  #  #  #  K  P  I  I  N  T  L   69
Cpac_S  577   Y  N  L  K  N  N  I  T  P  K  G  I  A  K  P  I  I  N  I  L  596
Cfau_S  576   Y  N  L  K  N  N  I  T  P  K  G  I  T  K  P  I  I  N  I  L  595
Cnau_S  576   Y  N  L  R  N  N  I  T  P  K  G  I  A  K  P  I  I  N  I  L  595
Pste_S  576   Y  N  L  K  N  N  I  T  P  K  G  I  A  K  P  I  I  N  I  L  595
Rma     576   Y  N  L  K  N  N  I  T  P  E  G  I  A  K  P  I  I  N  I  L  595
Ifos_S  576   Y  N  L  K  N  N  I  T  P  K  G  I  D  K  P  I  I  N  I  L  595
Apha_S  576   Y  N  L  K  N  N  I  T  P  K  G  I  A  K  P  I  I  N  I  L  595
Bsep_S  576   H  N  K  A  N  G  I  T  P  K  S  V  S  R  P  I  V  N  I  L  595
```

UvrB domain (550-591)

```
Akaw_S  928 TATACAAATTTAACCAAT---AAT---ATA-----------CATAGTA------GTTCTC  964
Clau_S 1627 GATATAAATTTAACTAATA--ACATACACAGAA---------------------GTTCTT 1663
Pkil_S  879 GATATAAATTTAACCAAT---AATAAAATAGAAAAATATGACATATATACA---GTGCTT  932
Psoy_S  879 GATATAAATTTAACCAAT---AATAAAATAGAAAAATATGACATATATACA---GTGCTT  932
Vok     215 GATATAAATTTAACCAAT---AATAAAATAGAAAAA-ATGACATATATACA---GTGCTT  267
Cpac_S 1789 GATACGGATTTAATCAGT---AGCCAGACAGAAGAGAATGGTGCTCAGAAA---GTGTTT 1842
Cfau_S 1789 GATACGGATTTAACCAGT---AGCCAGACAGAAGAGAATGGTGCTCAGAAA---GTGTTT 1842
Cnau_S 1789 GATACGGATTTAACCAGT---AGCCAGACAGAAGAGGATGGTGTTCAGAAA---GTGTTT 1842
Pste_S 1789 GATACGGATTTAACCAGT---AGCCAGACAGAAGAGGATGGTGCTCAGAAA---GTGTTT 1842
Rma    1789 GATACAGATTTAACTAGC---AACCAAATAGAAGAAGATAATGTTCAGAAA---GTTTTT 1842
Ifos_S 1789 GATACAGATTTAACCAGT---AACCAGATCGAGGAAGATGGTACTCAGAAA---GTGTTT 1842
Apha_S 1789 GATACGGATTTAACCAGT---AGCCAGAAAAAAGAGAATGGCGTTCAGAAA---GTGTTT 1842
Bsep_S 1789 GACACCGATGATGCTATG---GTTGCCGATCCAGAGGGTGAATACGATACCCAGGTCATA 1845
Akaw_S  303   Y  T  N  L  T  N  #  N  #  I  #  #  #  #  I  V  #  #  V  L  314
Clau_S  538   D  I  N  L  T  N  #  T  Y  T  E  #  #  #  #  #  #  #  V  L  549
Pkil_S  286   D  I  N  L  T  N  #  N  K  I  E  K  Y  D  I  Y  T  #  V  L  303
Psoy_S  286   D  I  N  L  T  N  #  N  K  I  E  K  Y  D  I  Y  T  #  V  L  303
Vok      70   D  I  N  L  T  N  #  N  K  I  E  K  #  D  I  Y  T  #  V  L   86
Cpac_S  597   D  T  D  L  I  S  #  S  Q  T  E  E  N  G  A  Q  K  #  V  F  614
Cfau_S  596   D  T  D  L  T  S  #  S  Q  T  E  E  N  G  A  Q  K  #  V  F  613
Cnau_S  596   D  T  D  L  T  S  #  S  Q  T  E  E  D  G  V  Q  K  #  V  F  613
Pste_S  596   D  T  D  L  T  S  #  S  Q  T  E  E  D  G  A  Q  K  #  V  F  613
Rma     596   D  T  D  L  T  S  #  N  Q  I  E  E  D  N  V  Q  K  #  V  F  613
Ifos_S  596   D  T  D  L  T  S  #  N  Q  I  E  E  D  G  T  Q  K  #  V  F  613
Apha_S  596   D  T  D  L  T  S  #  S  Q  K  K  E  N  G  V  Q  K  #  V  F  613
Bsep_S  596   D  T  D  D  A  M  #  V  A  D  P  E  G  E  Y  D  T  Q  V  I  614
```

```
Akaw_S   965 ATTCAACTTTCACCAGTGCAATTAGCAAAATAA---ATTAAAATATTAGAAAAGAAAATG 1021
Clau_S  1664 AGTCAATTTTAGCCAGTACATTTAGCAATATAA--ATATAAAATATTAGAAGAAAAAATA 1721
Pkil_S   933 ATTTAACTTTCACCGATGCAATTAGTAAAAGAA--ATATAAAGTATTAGAAGAGAAAATG  990
Psoy_S   933 ATTTAACTTTCACCGATGCAATTAGTAAAAGAA--ATATAAAGTATTAGAAGAGAAAATG  990
Vok      268 ATTCAACTTTTACTGGTGCAATTATTAAAATAA--ATATAAAATATTAGAAGAGAAAATG  325
Cpac_S  1843 ACTCAACTTTCACCAGTACAATTAGCAAAAGAA---ATTAAGATGCTAGAAAAGCAAATG 1899
Cfau_S  1843 ACTCAACTTTCACCAGTACAATTAGCAAAAGAA---ATTAAGATACTAGAAAAGAAAATG 1899
Cnau_S  1843 ACTCAACTTTCACCAGTACAATTAGCAAAAGAA---ATTAAAATGCTAGAAAAGCAAATG 1899
Pste_S  1843 ACTCAACTTTCACCAGTACAATTAGCAAAAGAA---ATTAAAATACTAGAAAAGAAAATG 1899
Rma     1843 ATCCAACTTTCACCTGTACAATTAGCAAAAAAA---ATTAAAATATTAGAAAAGCAAATG 1899
Ifos_S  1843 ACCCAACTTTTACCCGTGCAATTAGCAAAAGAA---ATTAAAATATTAGAAAAGAAAATG 1899
Apha_S  1843 ACTCAACTTTCACCAGTACAATTAGCAAAAGAA---ATTAAAGTGCTAGAAAAGCAAATG 1899
Bsep_S  1846 CAGCAACTATCCCCTGTGCAACTTGCTAAAGCG---TTAAAGGCATTAGAAAAAGAGATG 1902
Akaw_S   315   I  Q  L  S  P  V  Q  L  A  K  *  #  I  K  I  L  E  K  K  M  333
Clau_S   550   S  Q  F  *  P  V  H  L  A  I  *  #  Y  K  I  L  E  E  K  I  568
Pkil_S   304   I  *  L  S  P  M  Q  L  V  K  E  #  Y  K  V  L  E  E  K  M  322
Psoy_S   304   I  *  L  S  P  M  Q  L  V  K  E  #  Y  K  V  L  E  E  K  M  322
Vok       87   I  Q  L  L  L  V  Q  L  L  K  *  #  Y  K  I  L  E  E  K  M  105
Cpac_S   615   T  Q  L  S  P  V  Q  L  A  K  E  #  I  K  M  L  E  K  Q  M  633
Cfau_S   614   T  Q  L  S  P  V  Q  L  A  K  E  #  I  K  I  L  E  K  K  M  632
Cnau_S   614   T  Q  L  S  P  V  Q  L  A  K  E  #  I  K  M  L  E  K  Q  M  632
Pste_S   614   T  Q  L  S  P  V  Q  L  A  K  E  #  I  K  I  L  E  K  K  M  632
Rma      614   I  Q  L  S  P  V  Q  L  A  K  K  #  I  K  I  L  E  K  Q  M  632
Ifos_S   614   T  Q  L  L  P  V  Q  L  A  K  E  #  I  K  I  L  E  K  K  M  632
Apha_S   614   T  Q  L  S  P  V  Q  L  A  K  E  #  I  K  V  L  E  K  Q  M  632
Bsep_S   615   Q  Q  L  S  P  V  Q  L  A  K  A  A  L  K  A  L  E  K  E  M  634
```

```
Akaw_S  1022 TATGGTTTTGCTACCGATTTAGAGTCTGAATATGTAGTAGATATGTGTAATAAGATTAAA 1081
Clau_S  1722 TATAGTTTTGTTACTGATTTAGAGTTTGAATATGTAGTAGATATACGTAATCAGATTAAA 1781
Pkil_S   991 TATAA-TTTGTTACTGATTTATAGTTTGAATATGCAGTGGATATGCGTAATCAGATTAAA 1049
Psoy_S   991 TATAA-TTTGTTACTGATTTATAGTTTGAATATGCAGTGGATATGCGTAATCAGATTAAA 1049
Vok      326 TATAG-TTTGCTACTGATTTAGAGTTTGAATATGCAGTAGATATGCGTAATCAGATTAAA  384
Cpac_S  1900 TATAGTTTTGCTAGCGATTTGGCGTTTGAACGTGCAGCAGATATGCGTAATCAGATTAAA 1959
Cfau_S  1900 TATAGTTTTGCTAGCGATTTAGCGTTTGAACGTGCAGCAGATATGCGTAATCAGATTAAA 1959
Cnau_S  1900 TATAGTTTTGCTAGCGATTTGGCGTTTGAACGTGCAGCAGATATGCGTAATCAAATTAAA 1959
Pste_S  1900 TATAGTTTTGCTAGCGATTTGGCGTTTGAACGTGCAGCAGATATGCGTAATCAGATTAAA 1959
Rma     1900 TATAGTTTTGCCAACGATTTGGCGTTTGAACATGCAGCAGATATGCGTAATCAGATTAAA 1959
Ifos_S  1900 TATGGTTTTGCTAGCGATTTGGCGTTTGAGCGTGCAGCAGATGTGCGTAATCAGATTAAA 1959
Apha_S  1900 TATAGTTTTGCTAGCGATTTAGCGTTTGAACATGCGGCAGATATGCGTAATCAGATTAAA 1959
Bsep_S  1903 ATTGGTTTTGCAGAGGAGTTAAAATTTGAACAGGCAGCTGACATTCGTAATAAAATTAAA 1962
Akaw_S   334   Y  G  F  A  T  D  L  E  S  E  Y  V  V  D  M  C  N  K  I  K  353
Clau_S   569   Y  S  F  V  T  D  L  E  F  E  Y  V  V  D  I  R  N  Q  I  K  588
Pkil_S   323   Y  #  F  V  T  D  L  *  F  E  Y  A  V  D  M  R  N  Q  I  K  341
Psoy_S   323   Y  #  F  V  T  D  L  *  F  E  Y  A  V  D  M  R  N  Q  I  K  341
Vok      106   Y  #  F  A  T  D  L  E  F  E  Y  A  V  D  M  R  N  Q  I  K  124
Cpac_S   634   Y  S  F  A  S  D  L  A  F  E  R  A  A  D  M  R  N  Q  I  K  653
Cfau_S   633   Y  S  F  A  S  D  L  A  F  E  R  A  A  D  M  R  N  Q  I  K  652
Cnau_S   633   Y  S  F  A  S  D  L  A  F  E  R  A  A  D  M  R  N  Q  I  K  652
Pste_S   633   Y  S  F  A  S  D  L  A  F  E  R  A  A  D  M  R  N  Q  I  K  652
Rma      633   Y  S  F  A  N  D  L  A  F  E  H  A  A  D  M  R  N  Q  I  K  652
Ifos_S   633   Y  G  F  A  S  D  L  A  F  E  R  A  A  D  V  R  N  Q  I  K  652
Apha_S   633   Y  S  F  A  S  D  L  A  F  E  H  A  A  D  M  R  N  Q  I  K  652
Bsep_S   635   I  G  F  A  E  E  L  K  F  E  Q  A  A  D  I  R  N  K  I  K  654
```

```
Akaw_S 1082 CAACTCAAAGACGCTCAA---T---AAAAA---ATATTATGA 1114
Clau_S 1782 CAACTCAAAGACACTCAAT--TTTAAAAAA---ATATTATGA 1818
Pkil_S 1050 AAACTCTAAAAACATTTAG--TTTAAAAAA---ATATTATGA 1086
Psoy_S 1050 AAACTCTAAAAACATTTAG--TTTAAAAAA---ATATTATGA 1086
Vok     385 CAACTCAAAGACACTTAA---TTTAAAAAA---ATATTATGA  420
Cpac_S 1960 CAACTCAAGGACACTCAA---TTTAAAAAA---GCATTATGA 1995
Cfau_S 1960 CAACTCAAGGACACTCAA---TTTAAAAAA---GCATTATGA 1995
Cnau_S 1960 CAACTCAAGGACACTCAA---TTTAAAAAA---GCATTATGA 1995
Pste_S 1960 CAACTCAAGGACACTCAA---TTTAAAAAA---GCATTATGA 1995
Rma    1960 CAGCTGAAAGACACTCAA---TTTAAAAAA---ATATTTTGA 1995
Ifos_S 1960 CAGCTCAAAGACACTCAA---TTTAAAAAGCATTATGATTAA 1998
Apha_S 1960 CAACTCAAGGATACTCAA---TTTAAAAAA---GCATTATGA 1995
Bsep_S 1963 AAATTAAAAGAGACGCAA---TTCAAATCA---GGACTATGA 1998
Akaw_S  354   Q  L  K  D  A  Q  #  #  #  K  #  I  L  *  363
Clau_S  589   Q  L  K  D  T  Q  #  F  K  K  #  I  L  *  600
Pkil_S  342   K  L  *  K  H  L  #  F  K  K  #  I  L  *  353
Psoy_S  342   K  L  *  K  H  L  #  F  K  K  #  I  L  *  353
Vok     125   Q  L  K  D  T  *  #  F  K  K  #  I  L  *  136
Cpac_S  654   Q  L  K  D  T  Q  #  F  K  K  #  A  L  *  665
Cfau_S  653   Q  L  K  D  T  Q  #  F  K  K  #  A  L  *  664
Cnau_S  653   Q  L  K  D  T  Q  #  F  K  K  #  A  L  *  664
Pste_S  653   Q  L  K  D  T  Q  #  F  K  K  #  A  L  *  664
Rma     653   Q  L  K  D  T  Q  #  F  K  K  #  I  F  *  664
Ifos_S  653   Q  L  K  D  T  Q  #  F  K  K  H  Y  D  *  665
Apha_S  653   Q  L  K  D  T  Q  #  F  K  K  #  A  L  *  664
Bsep_S  655   K  L  K  E  T  Q  #  F  K  S  #  G  L  *  666
```

References

2. Gorbalenya AE, Koonin EV, Donchenko AP, Blinov VM. Two related superfamilies of putative helicases involved in replication, recombination, repair and expression of DNA and RNA genomes. Nucleic Acids Res. 1989;17(12):4713-30. PubMed PMID: 2546125; PubMed Central PMCID: PMC318027.

3. Theis K, Chen PJ, Skorvaga M, Van Houten B, Kisker C. Crystal structure of UvrB, a DNA helicase adapted for nucleotide excision repair. EMBO J. 1999;18(24):6899-907. doi: 10.1093/emboj/18.24.6899. PubMed PMID: 10601012; PubMed Central PMCID: PMC1171753.
