# Supplementary material for: Loss of genes related to Nucleotide Excision Repair (NER) and implications for reductive genome evolution in symbionts of deep-sea vesicomyid clams
Source: PLoS One. 2017 Feb 15;12(2):e0171274. doi: 10.1371/journal.pone.0171274 (PMC5310779; doi:10.1371/journal.pone.0171274)
Supplement: S3 Fig — The arrangement of nucleotide and amino acid sequences is as described in S1 Fig. Conserved domains of mfd found in an NCBI blast search are shown as bidirectional arrows. Seven helicase motifs of Mfd from Escherichia coli are shown below the alignment, and the corresponding sequences of the symbionts are underlined [4]. # indicates the gap of amino acid sequence where no corresponding nucleotide sequence exist. * indicates stop codon. (PDF) [file pone.0171274.s006.pdf]

S3 Fig.

```
Akaw_S     1 ATGCCATACCTCTATTCCAACAAAATTAAATGCTTTGAATTAGGTCGAAAAATGTATTGG   60
Clau_S     1 ATGCCATATCTCTATTCCAACAAAGTTAAATGCTTTAAATTAGGTCGAAAAATGTATTGG   60
Pkil_S     1 ATGCCATATCTCTATTCCAAAAAAATTAAATGCTTTGAATTAGGTCGAAAAATGTATTGG   60
Psoy_S     1 ATGCCATATCTCTATTCCAAAAAAATTAAATGCTTTGAATTAGGTCGAAAAATGTATTGG   60
Vok        1 ATGCCATATCTCTATTCCAACAAAGTTAAATGCTTTGAATTAGGTCGAAAAATGTATTGG   60
Cpac_S     1 ATGCAATATCTTTATCCTAGTAAGCTCAAGTGCTTTCAATCGGGTCAGAAAACTTATTGG   60
Cfau_S     1 ATGCAATATCTTTATCCTTATAAGCCTAAATGCTTTCAATCGGGTCAAAAAACTTATTGG   60
Cnau_S     1 ATGCAATATCTTTATCCTGATAAGCTCAAGTGCTTTCAATCGGGTCAAAAAACTTATTGG   60
Pste_S     1 ATGCAATATCTTTATCCTGATAAGCTCAAGTACTTTCAATCGGGTCAAAAAACTTATTGG   60
Rma        1 ATGCAATATCTCTATCCTGATAAGCTCAAATGCTTTCAATCGAATCAAAAAACCTATTGG   60
Ifos_S     1 ATGTTATATCTCTATCCTGATAAGCTTAAATGCTTTCAATCAGGTCAAAAAACTTATTGG   60
Apha_S     1 ATGCCATATCTCTATCCTGATAAGCTTAAACGCTTTCAATCAGGTCAAAAAACTTATTGG   60
Bsep_S     1 ATGCAGTCCTTTTATCCACAGGGTGTAACGCCTTTCGAGTCCGGGCAAAAATTTTATTGG   60
Akaw_S     1   M  P  Y  L  Y  S  N  K  I  K  C  F  E  L  G  R  K  M  Y  W   20
Clau_S     1   M  P  Y  L  Y  S  N  K  V  K  C  F  K  L  G  R  K  M  Y  W   20
Pkil_S     1   M  P  Y  L  Y  S  K  K  I  K  C  F  E  L  G  R  K  M  Y  W   20
Psoy_S     1   M  P  Y  L  Y  S  K  K  I  K  C  F  E  L  G  R  K  M  Y  W   20
Vok        1   M  P  Y  L  Y  S  N  K  V  K  C  F  E  L  G  R  K  M  Y  W   20
Cpac_S     1   M  Q  Y  L  Y  P  S  K  L  K  C  F  Q  S  G  Q  K  T  Y  W   20
Cfau_S     1   M  Q  Y  L  Y  P  Y  K  P  K  C  F  Q  S  G  Q  K  T  Y  W   20
Cnau_S     1   M  Q  Y  L  Y  P  D  K  L  K  C  F  Q  S  G  Q  K  T  Y  W   20
Pste_S     1   M  Q  Y  L  Y  P  D  K  L  K  Y  F  Q  S  G  Q  K  T  Y  W   20
Rma        1   M  Q  Y  L  Y  P  D  K  L  K  C  F  Q  S  N  Q  K  T  Y  W   20
Ifos_S     1   M  L  Y  L  Y  P  D  K  L  K  C  F  Q  S  G  Q  K  T  Y  W   20
Apha_S     1   M  P  Y  L  Y  P  D  K  L  K  R  F  Q  S  G  Q  K  T  Y  W   20
Bsep_S     1   M  Q  S  F  Y  P  Q  G  V  T  P  F  E  S  G  Q  K  F  Y  W   20
```

```
Akaw_S    61 GGGTCACTACATGGTAGTGCAGATGCGTTGGCATTAATTGAATTCGCCAACCAACAA---  117
Clau_S    61 GGTTCACTATATGGTAGTGCAGATGCATTGGCATTAATTGAATTCGCCAACCAACAA---  117
Pkil_S    61 GGTTCACTATATGGTAGTGCAGATGCATTGGCATTAATTGAATTCGCCAACCAACAA---  117
Psoy_S    61 GGTTCACTATATGGTAGTGCAGATGCATTGGCATTAATTGAATTCGCCAACCAACAA---  117
Vok       61 GGTTCACTATATGGTAGTGCAGATGCATTGGCATTAATTGAATTCGCCAACCAACAA---  117
Cpac_S    61 GGCTCACTGTACGGCAGTGCAGATGCACTGGCATTAATTGAATTTACCAATCAACAACAG  120
Cfau_S    61 GGCTCACTGTACGGCAGTGCAGATGCACTGGCATTAATTGAATTTGCCAATCAACAACAG  120
Cnau_S    61 GGCTCACTGTACGGCAGTGCAGATGCACTGGCATTAATTGAATTTGCCAATCAACAACAG  120
Pste_S    61 GGCTCACTGTACGGCAGTGCAGATGCACTAGCATTAATTGAATTTGCCAATCAACAACAG  120
Rma       61 GGCTCACTGTACGGTAGTGCAGATGCACTAGCATTAATTGAATTTACAAATCAACAACAG  120
Ifos_S    61 GGCTCACTATACGGCAGTGCAGATGCACTGGCATTAATTGAATTTGCCAATCAACAACAG  120
Apha_S    61 GGCTCACTATACGGCAGTGCAGATGCACTGGCATTAATTGAATTTTCCAATCAACAACAG  120
Bsep_S    61 GGGTCGCTTTACGGCAGCGCTCAGGCGTTAGCGTTGATTGAGTTTGCTCGGCAGCAAGAG  120
Akaw_S    21   G  S  L  H  G  S  A  D  A  L  A  L  I  E  F  A  N  Q  Q  #   39
Clau_S    21   G  S  L  Y  G  S  A  D  A  L  A  L  I  E  F  A  N  Q  Q  #   39
Pkil_S    21   G  S  L  Y  G  S  A  D  A  L  A  L  I  E  F  A  N  Q  Q  #   39
Psoy_S    21   G  S  L  Y  G  S  A  D  A  L  A  L  I  E  F  A  N  Q  Q  #   39
Vok       21   G  S  L  Y  G  S  A  D  A  L  A  L  I  E  F  A  N  Q  Q  #   39
Cpac_S    21   G  S  L  Y  G  S  A  D  A  L  A  L  I  E  F  T  N  Q  Q  Q   40
Cfau_S    21   G  S  L  Y  G  S  A  D  A  L  A  L  I  E  F  A  N  Q  Q  Q   40
Cnau_S    21   G  S  L  Y  G  S  A  D  A  L  A  L  I  E  F  A  N  Q  Q  Q   40
Pste_S    21   G  S  L  Y  G  S  A  D  A  L  A  L  I  E  F  A  N  Q  Q  Q   40
Rma       21   G  S  L  Y  G  S  A  D  A  L  A  L  I  E  F  T  N  Q  Q  Q   40
Ifos_S    21   G  S  L  Y  G  S  A  D  A  L  A  L  I  E  F  A  N  Q  Q  Q   40
Apha_S    21   G  S  L  Y  G  S  A  D  A  L  A  L  I  E  F  S  N  Q  Q  Q   40
Bsep_S    21   G  S  L  Y  G  S  A  Q  A  L  A  L  I  E  F  A  R  Q  Q  E   40
```

```
Akaw_S  118 CAAGTTATTTTAGTAATTGCTAATGATGTTGTACATTTTGATAATTTTTATAAATCTTTA  177
Clau_S  118 CAAGTCATTTTAGTAATTGCTAATGATATTGTACATTTTAATAATTTTTATAAATCTTTA  177
Pkil_S  118 CAAGTCATTTTAGTAATTGCTAATGATATTGTACATTTTAATAATTTTTATAAATCTTTA  177
Psoy_S  118 CAAGTCATTTTAGTAATTGCTAATGATATTGTACATTTTAATAATTTTTATAAATCTTTA  177
Vok     118 CATATCATTTTAGTAATTGCTAATGATATTGTACATTTTGATAATTTTTATAAATCTTTA  177
Cpac_S  121 CAAGTTATTTTAGTCATTGCTAATGACATTGCACATTTTGACAGTCTTTATAAATCCTTA  180
Cfau_S  121 CAAGTTATTTTAGTCATTGCTAATGACATTGCACATTTTGACAGTCTTTATAAATCTTTA  180
Cnau_S  121 CAAGTTATTTTAGTCATTGCTAATGACATTGCACATTTTGACAGTCTTTATAAATCTTTA  180
Pste_S  121 CAAGTTATTTTAGTCATTGCTAATGACATTGCACATTTTGACAGTCTTTATAAATCTTTA  180
Rma     121 CAAGTTATTTTAGTCATTACTGATGACACTGGACATTTTGACAATCTTTATAAATCTTTA  180
Ifos_S  121 CAAGTTATTTTAGTCATTGCTAATGACATCACACATTTTGACAGTCTTTATAAATCTTTA  180
Apha_S  121 CAAGTTATTTTAGTCATTGCTAATGACATTACCCATTTTGACAGTCTTTATAAATCTTTA  180
Bsep_S  121 CGGGTGATTTTGGTGGTGGCAAATGATATTAGCCACTTTGAGCAATTGTTTAAATCGCTC  180
Akaw_S   40   Q  V  I  L  V  I  A  N  D  V  V  H  F  D  N  F  Y  K  S  L   59
Clau_S   40   Q  V  I  L  V  I  A  N  D  I  V  H  F  N  N  F  Y  K  S  L   59
Pkil_S   40   Q  V  I  L  V  I  A  N  D  I  V  H  F  N  N  F  Y  K  S  L   59
Psoy_S   40   Q  V  I  L  V  I  A  N  D  I  V  H  F  N  N  F  Y  K  S  L   59
Vok      40   H  I  I  L  V  I  A  N  D  I  V  H  F  D  N  F  Y  K  S  L   59
Cpac_S   41   Q  V  I  L  V  I  A  N  D  I  A  H  F  D  S  L  Y  K  S  L   60
Cfau_S   41   Q  V  I  L  V  I  A  N  D  I  A  H  F  D  S  L  Y  K  S  L   60
Cnau_S   41   Q  V  I  L  V  I  A  N  D  I  A  H  F  D  S  L  Y  K  S  L   60
Pste_S   41   Q  V  I  L  V  I  A  N  D  I  A  H  F  D  S  L  Y  K  S  L   60
Rma      41   Q  V  I  L  V  I  T  D  D  T  G  H  F  D  N  L  Y  K  S  L   60
Ifos_S   41   Q  V  I  L  V  I  A  N  D  I  T  H  F  D  S  L  Y  K  S  L   60
Apha_S   41   Q  V  I  L  V  I  A  N  D  I  T  H  F  D  S  L  Y  K  S  L   60
Bsep_S   41   R  V  I  L  V  V  A  N  D  I  S  H  F  E  Q  L  F  K  S  L   60
```

```
Akaw_S  178 AAGTTTTATAACACTGACTTAGAAATTTTAAAATTTGATAATTGGGAGGTTCTCGCCTAT  237
Clau_S  178 AAGTTTTATAACACTGACTTAGAAATTTTAAAATTTGATAATTGGGAAGTTCTCGCTTAT  237
Pkil_S  178 AAGTTTTATAACACCGACCTAGAAATTTTAAAATTTGATAATTGGGAAGTTCTTGCCTAT  237
Psoy_S  178 AAGTTTTATAACACCGACCTAGAAATTTTAAAATTTGATAATTGGGAAGTTCTTGCCTAT  237
Vok     178 AAGTTTTATAACACTGACCTAGAAATTTTAAAATTTGATAATTGGGAAGTTCTTGCCTAT  237
Cpac_S  181 AATTTCTACAACACAGACTTAGAAATTTTAAAATTTGATAATTGGGAAGTGCTCGCCTAT  240
Cfau_S  181 AATTTCTACAACACAGATTTAGAAATTTTAAAATTTGATAACTGGGAAGTGCTCGCCTAT  240
Cnau_S  181 AACTTCTACAATACAGACCTAGAAATTTTAAAATTTGATAACTGGGAAGTGCTCGCCTAT  240
Pste_S  181 AACTTCTACAACACAGACTTAGAAATTTTAAAATTTGATAACTGGGAAGTGCTCGCCTAT  240
Rma     181 AACTTCTACAACACAAACTTAGAAATTTTAAAATTTGATAGTTGGGAAGTGCTCGCCTAT  240
Ifos_S  181 AACTTCTACAACACAGACTTAGAAATTTTAAAATTTGATAACTGGGAAGTGCTCGCCTAT  240
Apha_S  181 AACTTCTACAATACAGACTTAGAAATTTTAAAATTTGATAGCTGGGAGGTACTCGCCTAT  240
Bsep_S  181 AATTTTTATGGCAGTGACTTGGCAGTTTTACGCTTTGATAATTGGGAGGTGTTGCCGTTT  240
Akaw_S   60   K  F  Y  N  T  D  L  E  I  L  K  F  D  N  W  E  V  L  A  Y   79
Clau_S   60   K  F  Y  N  T  D  L  E  I  L  K  F  D  N  W  E  V  L  A  Y   79
Pkil_S   60   K  F  Y  N  T  D  L  E  I  L  K  F  D  N  W  E  V  L  A  Y   79
Psoy_S   60   K  F  Y  N  T  D  L  E  I  L  K  F  D  N  W  E  V  L  A  Y   79
Vok      60   K  F  Y  N  T  D  L  E  I  L  K  F  D  N  W  E  V  L  A  Y   79
Cpac_S   61   N  F  Y  N  T  D  L  E  I  L  K  F  D  N  W  E  V  L  A  Y   80
Cfau_S   61   N  F  Y  N  T  D  L  E  I  L  K  F  D  N  W  E  V  L  A  Y   80
Cnau_S   61   N  F  Y  N  T  D  L  E  I  L  K  F  D  N  W  E  V  L  A  Y   80
Pste_S   61   N  F  Y  N  T  D  L  E  I  L  K  F  D  N  W  E  V  L  A  Y   80
Rma      61   N  F  Y  N  T  N  L  E  I  L  K  F  D  S  W  E  V  L  A  Y   80
Ifos_S   61   N  F  Y  N  T  D  L  E  I  L  K  F  D  N  W  E  V  L  A  Y   80
Apha_S   61   N  F  Y  N  T  D  L  E  I  L  K  F  D  S  W  E  V  L  A  Y   80
Bsep_S   61   N  F  Y  G  S  D  L  A  V  L  R  F  D  N  W  E  V  L  P  F   80
```

```
Akaw_S  238 GATCACTTCTCACCTCATCCTGATATTACCTCAAATAGATTAAAAACTTTATCAAAACTT  297
Clau_S  238 GATTACTTCTCACCTCATCCTGATATTACCTCAAGTAGATTAAAAACCTTATCAAAACTT  297
Pkil_S  238 GATTACTTCTCACCTCATCCTGATATTACCTCCAGTAGATTAAAAACCTTATCAAAACTT  297
Psoy_S  238 GATTACTTCTCACCTCATCCTGATATTACCTCCAGTAGATTAAAAACCTTATCAAAACTT  297
Vok     238 GATTACTTCTCACCTCATCCTGATATTACCTCCAGTAGATTAAAAACCTTATCAAAACTT  297
Cpac_S  241 GATCACTTTTCGCCTCATCCTGATATCACCTCAAGCCGACTAAAAACCTTATCAAAACTT  300
Cfau_S  241 GATCACTTTTCGCCTCATCCTGATATCACCTCAAGTCGACTAAAAACCTTATCAAAACTT  300
Cnau_S  241 GATCACTTTTCACCTCATCCTGATATCACATCAAACCGACTAAAAACCTTATCAAAACTT  300
Pste_S  241 GATCACTTTTCGCCTCATCCTGATATCACCTCAAGCCGACTAAAAACCTTATCAAAACTT  300
Rma     241 GATCACTTTTCACCTCATCCTGATATCACATCAAGTCGATTAAAAACCTTATCAAAACTT  300
Ifos_S  241 GATCACTTTTCGCCTCATCCTGATATCACCTCCAGTCGATTAGCAGCCCTATCAAAACTT  300
Apha_S  241 GATCACTTTTCACCTCATCCTGATATCATCTCAAGTCGATTAAAAACCCTATCAAAACTT  300
Bsep_S  241 GACCATTTTTCTCCGCATCCGGATATCACCTCTAGTCGCCTTGATACGCTGTCTAAATTA  300
Akaw_S   80   D  H  F  S  P  H  P  D  I  T  S  N  R  L  K  T  L  S  K  L    99
Clau_S   80   D  Y  F  S  P  H  P  D  I  T  S  S  R  L  K  T  L  S  K  L    99
Pkil_S   80   D  Y  F  S  P  H  P  D  I  T  S  S  R  L  K  T  L  S  K  L    99
Psoy_S   80   D  Y  F  S  P  H  P  D  I  T  S  S  R  L  K  T  L  S  K  L    99
Vok      80   D  Y  F  S  P  H  P  D  I  T  S  S  R  L  K  T  L  S  K  L    99
Cpac_S   81   D  H  F  S  P  H  P  D  I  T  S  S  R  L  K  T  L  S  K  L   100
Cfau_S   81   D  H  F  S  P  H  P  D  I  T  S  S  R  L  K  T  L  S  K  L   100
Cnau_S   81   D  H  F  S  P  H  P  D  I  T  S  N  R  L  K  T  L  S  K  L   100
Pste_S   81   D  H  F  S  P  H  P  D  I  T  S  S  R  L  K  T  L  S  K  L   100
Rma      81   D  H  F  S  P  H  P  D  I  T  S  S  R  L  K  T  L  S  K  L   100
Ifos_S   81   D  H  F  S  P  H  P  D  I  T  S  S  R  L  A  A  L  S  K  L   100
Apha_S   81   D  H  F  S  P  H  P  D  I  I  S  S  R  L  K  T  L  S  K  L   100
Bsep_S   81   D  H  F  S  P  H  P  D  I  T  S  S  R  L  D  T  L  S  K  L   100
```

```
Akaw_S  298 AGAAGTCTTAAACGTGGTATTGTTATCACCACGTTAGAGTCATTATTTTCACACTTATGC  357
Clau_S  298 AAAAGTCTTAAACGTGGTATTATTATCACCACGTTAGAGTCGTTATTTTCACACTTATGC  357
Pkil_S  298 AAAAGCCTTAAACGGGGTATTGTTATCACCACGTTAGAGTCGTTATTTTCACACTTATGC  357
Psoy_S  298 AAAAGCCTTAAACGGGGTATTGTTATCACCACGTTAGAGTCGTTATTTTCACACTTATGC  357
Vok     298 AAAAGTCTTAAACGAGGTATTGTTATCACCACGTTAGAGTCGTTATTTTCACACTTATGT  357
Cpac_S  301 AAAAATCTTAAATGCGGTATTGTTCTCACCACACTAGAATCATTGTTTTCACACTTATGC  360
Cfau_S  301 AAAAATCTTAAACGCGGTATTGTTCTCACCACACTAGAATCATTATTTTCACACTTATGC  360
Cnau_S  301 AAAAATCTTAAACGCGGTATTGTTCTCACCACACTAGAATCATTATTCTCACACTTATGC  360
Pste_S  301 AAAAATCTTAAACGCGGTATTGTTCTCACCACACTAGAATCATTATTTTCACACTTATGC  360
Rma     301 AAAAATCTTAAAAATGGTATTATTATCACTACATTAGAATCATTATTTTCATACTTATGC  360
Ifos_S  301 AAAAGTCTTAAACGTGGTATTGTTATCACCACATTAGAATCATTATTCTCACGTCTATGC  360
Apha_S  301 AAAAGTCTTAAACGTGGTATTGTTATCACCACATTAGAATCATTATTCTCACGTTTATGC  360
Bsep_S  301 CCGACGCTAAAACGCGGTATTGTTATTACCACTTTGGAGTCTCTGTCTCAGAAGTTGTGT  360
Akaw_S  100   R  S  L  K  R  G  I  V  I  T  T  L  E  S  L  F  S  H  L  C   119
Clau_S  100   K  S  L  K  R  G  I  I  I  T  T  L  E  S  L  F  S  H  L  C   119
Pkil_S  100   K  S  L  K  R  G  I  V  I  T  T  L  E  S  L  F  S  H  L  C   119
Psoy_S  100   K  S  L  K  R  G  I  V  I  T  T  L  E  S  L  F  S  H  L  C   119
Vok     100   K  S  L  K  R  G  I  V  I  T  T  L  E  S  L  F  S  H  L  C   119
Cpac_S  101   K  N  L  K  C  G  I  V  L  T  T  L  E  S  L  F  S  H  L  C   120
Cfau_S  101   K  N  L  K  R  G  I  V  L  T  T  L  E  S  L  F  S  H  L  C   120
Cnau_S  101   K  N  L  K  R  G  I  V  L  T  T  L  E  S  L  F  S  H  L  C   120
Pste_S  101   K  N  L  K  R  G  I  V  L  T  T  L  E  S  L  F  S  H  L  C   120
Rma     101   K  N  L  K  N  G  I  I  I  T  T  L  E  S  L  F  S  Y  L  C   120
Ifos_S  101   K  S  L  K  R  G  I  V  I  T  T  L  E  S  L  F  S  R  L  C   120
Apha_S  101   K  S  L  K  R  G  I  V  I  T  T  L  E  S  L  F  S  R  L  C   120
Bsep_S  101   P  T  L  K  R  G  I  V  I  T  T  L  E  S  L  S  Q  K  L  C   120
```

```
Akaw_S  358 CCACTAGAGTTTAGCGAAAAATATATCTTAAATATTCGTATTAATGACAAAATTAATTCT  417
Clau_S  358 CCACTAGAGTTTAGCGAAAAATATAGCTTAAATATTCGTATTAATGACAAAATTAATTCT  417
Pkil_S  358 CCACTAGAGTTTAGCAAAAAATATAGCTTAAATATTCATATTAATGACAAAATTAATCCT  417
Psoy_S  358 CCACTAGAGTTTAGCAAAAAATATAGCTTAAATATTCATATTAATGACAAAATTAATCCT  417
Vok     358 CCACTAGAGTTTAGCAAAAAATATAGCTTAAATATTCGTATTAATGACAAAATTAATCCT  417
Cpac_S  361 CCGTTAGAGTTTGGCGAAGAATATAGCTTTAATATTGGCATTAATGACAATATTAATCTT  420
Cfau_S  361 CCGTTAGAGTTTGGCGAAAAATATAGCTTTAATATTGGTATTAATGACAATATTAATCTT  420
Cnau_S  361 CCGTTAAAGTTTGGCGAAAAATATAGCTTTAATATTGGTATTAATGACAATATTAATCTT  420
Pste_S  361 CCGTTAGAGTTTGGCGAAAAATATAGCTTTAATATTGGTATTAATGATAATATTAATTTT  420
Rma     361 CCGTTAGAGTTTAGCGAAAAATATAGCTTTAATATTAATATTAATGACAATATTAATATT  420
Ifos_S  361 CCGTTAGAGTTTGTAGAAAAATACAGCTTCAATATCGATATTAATGACAACATTAATATT  420
Apha_S  361 CCATTAGAGTTTGGCGAAAAATATAGCTTCAATATTAATATTAATGACAACATTAATATT  420
Bsep_S  361 CCAATTGAGTTTAGCAAGCAATACAGTTTTAGTCTGAAAAGTGGCGATGATTTGGAGATA  420
Akaw_S  120   P  L  E  F  S  E  K  Y  I  L  N  I  R  I  N  D  K  I  N  S  139
Clau_S  120   P  L  E  F  S  E  K  Y  S  L  N  I  R  I  N  D  K  I  N  S  139
Pkil_S  120   P  L  E  F  S  K  K  Y  S  L  N  I  H  I  N  D  K  I  N  P  139
Psoy_S  120   P  L  E  F  S  K  K  Y  S  L  N  I  H  I  N  D  K  I  N  P  139
Vok     120   P  L  E  F  S  K  K  Y  S  L  N  I  R  I  N  D  K  I  N  P  139
Cpac_S  121   P  L  E  F  G  E  E  Y  S  F  N  I  G  I  N  D  N  I  N  L  140
Cfau_S  121   P  L  E  F  G  E  K  Y  S  F  N  I  G  I  N  D  N  I  N  L  140
Cnau_S  121   P  L  K  F  G  E  K  Y  S  F  N  I  G  I  N  D  N  I  N  L  140
Pste_S  121   P  L  E  F  G  E  K  Y  S  F  N  I  G  I  N  D  N  I  N  F  140
Rma     121   P  L  E  F  S  E  K  Y  S  F  N  I  N  I  N  D  N  I  N  I  140
Ifos_S  121   P  L  E  F  V  E  K  Y  S  F  N  I  D  I  N  D  N  I  N  I  140
Apha_S  121   P  L  E  F  G  E  K  Y  S  F  N  I  N  I  N  D  N  I  N  I  140
Bsep_S  121   P  I  E  F  S  K  Q  Y  S  F  S  L  K  S  G  D  D  L  E  I  140
```

```
Akaw_S  418 GATACTTTTAATAAAGAATTACTAAAAATTGGATACAAGCATGTAATTACAGTAATGGAG  477
Clau_S  418 GATACTTTTAGTGAAAAATTACTAAAAATTGGATACAAAAATGTAATCACAGTAATGGAG  477
Pkil_S  418 GATAATTTTAGTGAAAAATTACTAAAAATTGGATACAAGCATGTAATCACAGTAATGGAG  477
Psoy_S  418 GATAATTTTAGTGAAAAATTACTAAAAATTGGATACAAGCATGTAATCACAGTAATGGAG  477
Vok     418 GATACTTTTAGTGAAAAATTACTAAAAATTGGATACAAGCATGTAATCACAGTAATGGAG  477
Cpac_S  421 GATACTTTTAGTGAAAGACTACTAAAAATTGGCTACAACCGCGTAACTACAGTGATGGAA  480
Cfau_S  421 GGTACTTTTAGTGAAAGACTACTAAAAATTGGCTACAACCGCGTAACCACAGTGATGGAG  480
Cnau_S  421 GATACTTTTAGTGAAAAACTACTAAGAATTGGCTACAACCGCGTAACCATAGTAATGGAG  480
Pste_S  421 GATACTTTTAGTGAAAAACTACTAAAAATTGGCTACAACCGCGTAACCACAGTGATGGAA  480
Rma     421 AAAGCTTTTAGTAAAAAACTACTAAAAATTGGCTACAACCGTGTAACCACCGTAATGGAA  480
Ifos_S  421 GAAGCCTTTAGTGAAAAACTACTAAAAATTGGCTACAACCGTGTAACCACCGTCATGGGG  480
Apha_S  421 GAAGCCTTTAGTGAAAAACTACTCAAAATTGGCTACAACCGTGTAACCACCGTAATGGAG  480
Bsep_S  421 CAGTCATTCTCTCAACGCCTGTTGAAAATCGGCTATAACCGCGTGAATACAGTTAGAGAG  480
Akaw_S  140   D  T  F  N  K  E  L  L  K  I  G  Y  K  H  V  I  T  V  M  E  159
Clau_S  140   D  T  F  S  E  K  L  L  K  I  G  Y  K  N  V  I  T  V  M  E  159
Pkil_S  140   D  N  F  S  E  K  L  L  K  I  G  Y  K  H  V  I  T  V  M  E  159
Psoy_S  140   D  N  F  S  E  K  L  L  K  I  G  Y  K  H  V  I  T  V  M  E  159
Vok     140   D  T  F  S  E  K  L  L  K  I  G  Y  K  H  V  I  T  V  M  E  159
Cpac_S  141   D  T  F  S  E  R  L  L  K  I  G  Y  N  R  V  T  T  V  M  E  160
Cfau_S  141   G  T  F  S  E  R  L  L  K  I  G  Y  N  R  V  T  T  V  M  E  160
Cnau_S  141   D  T  F  S  E  K  L  L  R  I  G  Y  N  R  V  T  I  V  M  E  160
Pste_S  141   D  T  F  S  E  K  L  L  K  I  G  Y  N  R  V  T  T  V  M  E  160
Rma     141   K  A  F  S  K  K  L  L  K  I  G  Y  N  R  V  T  T  V  M  E  160
Ifos_S  141   E  A  F  S  E  K  L  L  K  I  G  Y  N  R  V  T  T  V  M  G  160
Apha_S  141   E  A  F  S  E  K  L  L  K  I  G  Y  N  R  V  T  T  V  M  E  160
Bsep_S  141   Q  S  F  S  Q  R  L  L  K  I  G  Y  N  R  V  N  T  V  R  E  160
```

```
Akaw_S  478 CGTGGTGAGTTTAATATACGTAGCTCATTGATTGATCTATACCCGATGGGTACAAAAACA  537
Clau_S  478 CATGGTGAGTTTAATATACGTAACTCATTGATTGATCTATACCCGATGGGTGCAAAAACA  537
Pkil_S  478 CATGGTGAGTTTAATATGCGTAATTCATTGATTGATCTATACCCAATGGGTGCAAAAACA  537
Psoy_S  478 CATGGTGAGTTTAATATGCGTAATTCATTGATTGATCTATACCCAATGGGTGCAAAAACA  537
Vok     478 CATGGTGAGTTTAATATGCGTAATTCATTGATTGATCTATACCCGATGGGTACAAAAACA  537
Cpac_S  481 CATGGTGAGTTTAATATACGTGGCTCATTGATTGATTTATACCCCATGGGTTCAAGAACA  540
Cfau_S  481 CATGGTGAGTTTAATGTACGTAGCTCATTGATTGATTTATACCCAATGGGTGCAAAAACA  540
Cnau_S  481 CATGGTGAGTTTAATATACGTGGCTCATTGATTGATTTATACCCAATGGGTGCAAAAACA  540
Pste_S  481 CATGGTGAGTTTAATGTACGTGGCTCATTGATTGATTTATACCCAATGGGTGCAAAAACA  540
Rma     481 CATGGTGAATTTAATATTCATGGCTCATTGATTGATTTATACCCAATGGGTGCAAAAACA  540
Ifos_S  481 CATGGTGAGTTTAATATTCGTGGCTCATTGATTGATTTATACCCCATGGGCGCAAAAACA  540
Apha_S  481 CATGGTGAGTTTAATATTCATGGCTCATTGATTGATTTATACCCAATAGGCGCAAAAACA  540
Bsep_S  481 CGCGGAGAATTTAGCGTTAAAGGCTCATTGATTGATCTGTTTCCAATGGGTGTGAATTTA  540
Akaw_S  160   R  G  E  F  N  I  R  S  S  L  I  D  L  Y  P  M  G  T  K  T  179
Clau_S  160   H  G  E  F  N  I  R  N  S  L  I  D  L  Y  P  M  G  A  K  T  179
Pkil_S  160   H  G  E  F  N  M  R  N  S  L  I  D  L  Y  P  M  G  A  K  T  179
Psoy_S  160   H  G  E  F  N  M  R  N  S  L  I  D  L  Y  P  M  G  A  K  T  179
Vok     160   H  G  E  F  N  M  R  N  S  L  I  D  L  Y  P  M  G  T  K  T  179
Cpac_S  161   H  G  E  F  N  I  R  G  S  L  I  D  L  Y  P  M  G  S  R  T  180
Cfau_S  161   H  G  E  F  N  V  R  S  S  L  I  D  L  Y  P  M  G  A  K  T  180
Cnau_S  161   H  G  E  F  N  I  R  G  S  L  I  D  L  Y  P  M  G  A  K  T  180
Pste_S  161   H  G  E  F  N  V  R  G  S  L  I  D  L  Y  P  M  G  A  K  T  180
Rma     161   H  G  E  F  N  I  H  G  S  L  I  D  L  Y  P  M  G  A  K  T  180
Ifos_S  161   H  G  E  F  N  I  R  G  S  L  I  D  L  Y  P  M  G  A  K  T  180
Apha_S  161   H  G  E  F  N  I  H  G  S  L  I  D  L  Y  P  I  G  A  K  T  180
Bsep_S  161   R  G  E  F  S  V  K  G  S  L  I  D  L  F  P  M  G  V  N  L  180
```

```
Akaw_S  538 GCTTATCGAATTAATTTATTTGACCAAAAAGTAGAATCCATTCGCTCCTTTAACACTTCA  597
Clau_S  538 GCTTATCGAATTAATTTATTTGACCAAAAAGTAGAATCCATTCGCGCCTTTAACACTTCA  597
Pkil_S  538 GCTTATCGAATTAATTTATTTAACCAAAAAGTAGAATCCATTCGTGCCTTTAACACTTCA  597
Psoy_S  538 GCTTATCGAATTAATTTATTTAACCAAAAAGTAGAATCCATTCGTGCCTTTAACACTTCA  597
Vok     538 GCTTATCGAATTAATTTATTTAACCAAAAAGTGGAATCCATTCGCGCCTTTAACACTTCA  597
Cpac_S  541 CCCTATCGAATTGATTTATTTGACCAAGAAGTAGAGTCTATCCGCACCTTTGATACCTCA  600
Cfau_S  541 CCCTACCGAATTGATTTATTTGACCAAGAAGTAGAGTCTATTCGCACCTTTGATACCTCA  600
Cnau_S  541 CCCTACCGAATTGATTTGTTTGACCAAGAAGTAGAGTCTATTCGTACCTTTGATACCTCA  600
Pste_S  541 CCCTACCGAATTGATTTGTTTGACCAAGAAGTAGAGTCTATTCGCACCTTTGATGCCTCA  600
Rma     541 CCTTACAGAATTGATTTGTTTGATCAAGAAGTAGAGTCTATTCGCACCTTTGACACCTCA  600
Ifos_S  541 CCTTACCGAATTGATTTGTTTGGCCAAGAAGTGGAGTCTATTCGCACCTTTGATACCTCA  600
Apha_S  541 CCCTACCGAATTGATTTGTTTGACCAAGAAGTAGAGTCTATTCGCACCTTTGATACTTCA  600
Bsep_S  541 CCGTATCGGATTGATTTGTTTGACCAAGAAATTGAATCAATTCGTATTTTTGACGCATCA  600
Akaw_S  180   A  Y  R  I  N  L  F  D  Q  K  V  E  S  I  R  S  F  N  T  S  199
Clau_S  180   A  Y  R  I  N  L  F  D  Q  K  V  E  S  I  R  A  F  N  T  S  199
Pkil_S  180   A  Y  R  I  N  L  F  N  Q  K  V  E  S  I  R  A  F  N  T  S  199
Psoy_S  180   A  Y  R  I  N  L  F  N  Q  K  V  E  S  I  R  A  F  N  T  S  199
Vok     180   A  Y  R  I  N  L  F  N  Q  K  V  E  S  I  R  A  F  N  T  S  199
Cpac_S  181   P  Y  R  I  D  L  F  D  Q  E  V  E  S  I  R  T  F  D  T  S  200
Cfau_S  181   P  Y  R  I  D  L  F  D  Q  E  V  E  S  I  R  T  F  D  T  S  200
Cnau_S  181   P  Y  R  I  D  L  F  D  Q  E  V  E  S  I  R  T  F  D  T  S  200
Pste_S  181   P  Y  R  I  D  L  F  D  Q  E  V  E  S  I  R  T  F  D  A  S  200
Rma     181   P  Y  R  I  D  L  F  D  Q  E  V  E  S  I  R  T  F  D  T  S  200
Ifos_S  181   P  Y  R  I  D  L  F  G  Q  E  V  E  S  I  R  T  F  D  T  S  200
Apha_S  181   P  Y  R  I  D  L  F  D  Q  E  V  E  S  I  R  T  F  D  T  S  200
Bsep_S  181   P  Y  R  I  D  L  F  D  Q  E  I  E  S  I  R  I  F  D  A  S  200
```

```
Akaw_S  598 ACTCAACGTTCTAAAACTAAAATACCTGAAATTACACTATTACCTGCTAGAGAGTTCGCA  657
Clau_S  598 AATCAACGTTCTAAAACTAAAACAGCTGAAATTACACTATTGCCTGCTAAAGAGTTCCCA  657
Pkil_S  598 ACTCAACGCTCTAAAACTAAAATACCTGAAATTACGCTATTGCCTGCTAGAGAGTTCGCG  657
Psoy_S  598 ACTCAACGCTCTAAAACTAAAATACCTGAAATTACGCTATTGCCTGCTAGAGAGTTCGCG  657
Vok     598 ACTCAACGTTCTAAAACTAAAATACCTGAAATTACACTATTGCCTGCTAGAGAGTTTGCA  657
Cpac_S  601 ACTCAACGCTCCAAGATGCAAGTATCTGAAATTGCGCTACTGCCTGCCAGAGAATTTGCC  660
Cfau_S  601 ACTCAACGCTCCAAGGTGCAAGTGTCTGAAATTGCGCTACTGCCTGCCAGAGAATTTCCC  660
Cnau_S  601 ACTCAACGCTCCAAGGTGCAAGTGTCTGAAATTGCGCTACTGCCTGCCAGAGAATTTGCC  660
Pste_S  601 ACTCAACGCTCCAAGGCGCAAGTGTCTGAAATTGCGCTACTGCCTGCCAGAGAATTTGCC  660
Rma     601 ACTCAACGCTCTAAAATGGAAGTGTCTAAAATTATGCTATTGCCTGCCAGGGAGTTTGCC  660
Ifos_S  601 ACTCAACGCTCTAAAGTACAAGTGCCTGAAATTGTGCTACTGCCTACTAGAGAATTTGCC  660
Apha_S  601 ACTCAACGCTCCAAGGTGCAAGTGTCTGAAATTGCGCTATTGCCTGCCAGAGAATTTGCC  660
Bsep_S  601 ACTCAGCGCTCTATTGAGGCGGTGGAACAGGTGTCATTGTTGCCAGCGAGAGAGTTTGCG  660
Akaw_S  200  T  Q  R  S  K  T  K  I  P  E  I  T  L  L  P  A  R  E  F  A   219
Clau_S  200  N  Q  R  S  K  T  K  T  A  E  I  T  L  L  P  A  K  E  F  P   219
Pkil_S  200  T  Q  R  S  K  T  K  I  P  E  I  T  L  L  P  A  R  E  F  A   219
Psoy_S  200  T  Q  R  S  K  T  K  I  P  E  I  T  L  L  P  A  R  E  F  A   219
Vok     200  T  Q  R  S  K  T  K  I  P  E  I  T  L  L  P  A  R  E  F  A   219
Cpac_S  201  T  Q  R  S  K  M  Q  V  S  E  I  A  L  L  P  A  R  E  F  A   220
Cfau_S  201  T  Q  R  S  K  V  Q  V  S  E  I  A  L  L  P  A  R  E  F  P   220
Cnau_S  201  T  Q  R  S  K  V  Q  V  S  E  I  A  L  L  P  A  R  E  F  A   220
Pste_S  201  T  Q  R  S  K  A  Q  V  S  E  I  A  L  L  P  A  R  E  F  A   220
Rma     201  T  Q  R  S  K  M  E  V  S  K  I  M  L  L  P  A  R  E  F  A   220
Ifos_S  201  T  Q  R  S  K  V  Q  V  P  E  I  V  L  L  P  T  R  E  F  A   220
Apha_S  201  T  Q  R  S  K  V  Q  V  S  E  I  A  L  L  P  A  R  E  F  A   220
Bsep_S  201  T  Q  R  S  I  E  A  V  E  Q  V  S  L  L  P  A  R  E  F  A   220
```

```
Akaw_S  658 ACAGATAATACCAGTATTGAATACTTTAAAACCAATTATAAAAAAACATTT---AATGAT  714
Clau_S  658 ACAGATAATACTAGTATTGAATACTTTAAAACCAATTATAAAAAAACATTT---AATGAT  714
Pkil_S  658 ACAGATAATACCAGTATTGAATACTTTAAAACCAATTATAAAAAAACATTT---AATGAT  714
Psoy_S  658 ACAGATAATACCAGTATTGAATACTTTAAAACCAATTATAAAAAAACATTT---AATGAT  714
Vok     658 ACAGATAATACTAGTATTGAATACTTTAAAACTAATTATAAAAAAATATTT---AATGAT  714
Cpac_S  661 ACAGATAATGCTAGCATTGAGCGGTTCAAAACCAATTATCAAAAAGCGTTT---AATAGC  717
Cfau_S  661 ACAGATAATGCTAGTATTGAGCGTTTCAAAACCAATTATCAAAAAGTGTTT---AATGAT  717
Cnau_S  661 ACAGATAATACTAGCATTGAGCGTTTCAAAACCAATTATCAAAAAGCGTTT---AATGAT  717
Pste_S  661 ACAGATAACGCTAGTATTGAGCGTTTCAAAACCAATTATCAAAAAGCGTTT---AATGAT  717
Rma     661 ACAGATAGTACTAGTATTGAACGCTTTAAAGTCAATTATCAAAAAGCATTT---AATGAC  717
Ifos_S  661 ACAGATAATACCAGTATTGAGCGCTTTAAAACTAATTATCAAAAAGCATTT---AATGAC  717
Apha_S  661 ACAGATAATGCTAGCATTGAGCGTTTCAAAACCAATTATCAAAAAGCGTTT---AATGAC  717
Bsep_S  661 ACTGACCAGAGCAGTATTGAGGTTTTTAAATCTAACTATCAAAAAGCATTTGGCAATACC  720
Akaw_S  220  T  D  N  T  S  I  E  Y  F  K  T  N  Y  K  K  T  F  #  N  D   238
Clau_S  220  T  D  N  T  S  I  E  Y  F  K  T  N  Y  K  K  T  F  #  N  D   238
Pkil_S  220  T  D  N  T  S  I  E  Y  F  K  T  N  Y  K  K  T  F  #  N  D   238
Psoy_S  220  T  D  N  T  S  I  E  Y  F  K  T  N  Y  K  K  T  F  #  N  D   238
Vok     220  T  D  N  T  S  I  E  Y  F  K  T  N  Y  K  K  I  F  #  N  D   238
Cpac_S  221  T  D  N  A  S  I  E  R  F  K  T  N  Y  Q  K  A  F  #  N  S   239
Cfau_S  221  T  D  N  A  S  I  E  R  F  K  T  N  Y  Q  K  V  F  #  N  D   239
Cnau_S  221  T  D  N  T  S  I  E  R  F  K  T  N  Y  Q  K  A  F  #  N  D   239
Pste_S  221  T  D  N  A  S  I  E  R  F  K  T  N  Y  Q  K  A  F  #  N  D   239
Rma     221  T  D  S  T  S  I  E  R  F  K  V  N  Y  Q  K  A  F  #  N  D   239
Ifos_S  221  T  D  N  T  S  I  E  R  F  K  T  N  Y  Q  K  A  F  #  N  D   239
Apha_S  221  T  D  N  A  S  I  E  R  F  K  T  N  Y  Q  K  A  F  #  N  D   239
Bsep_S  221  T  D  Q  S  S  I  E  V  F  K  S  N  Y  Q  K  A  F  G  N  T   240
```

```
Akaw_S  715 AATGGCTTTATTTATACCGAGGTTAGTGAAGGAAGATTACCAGGTGGTATTGAATTCTAT  774
Clau_S  715 AATGATTTTATTTATACCGAGGTTAGTGAAGGAAGATTACCTGGTGGTATTGAGTTCTAT  774
Pkil_S  715 AATGGTTTTATTTATACCGAAGTTAGTGAAGGAAGATTACCAGGTGGTATTGAGTTCTAT  774
Psoy_S  715 AATGGTTTTATTTATACCGAAGTTAGTGAAGGAAGATTACCAGGTGGTATTGAGTTCTAT  774
Vok     715 AATGGTTTTATTTATACCGAAGTTAGTGAAGGAAGATTACCAGGTGGTATTGAGTTCTAT  774
Cpac_S  718 CATGGTTTCATTTATACCGAAGTTAGTGAAGGTAGACTACCAGGCGGTATTGAATTCTAT  777
Cfau_S  718 CATAGTTTCATTTATACCGAAGTTAGTGAAGGCAGGCTACCAGGCGGTATTGAATTCTAT  777
Cnau_S  718 CATGGTTTCATTTATACCGAAGTTAGTGAAGGCAGGCTACCAGGCGGTATTGAATTCTAT  777
Pste_S  718 TATGGTTTCATTTATACCGAAGTTAGTGAAGGCAGACTACCAGGCGGTATTGAATTCTAT  777
Rma     718 GATGGCTTTATTTATACTGAAGTTAGTGAAGGTAGGTTACCAAGTGGTATTGAATTTTAT  777
Ifos_S  718 AATGGCTTTATCTATACCGAAGTTAGTGAAGGTAGGCTGCCAGGTGGCATTGAATTTTAT  777
Apha_S  718 AATGGCTTTATTTATACCGAAGTTAGCGAAGGTAGGCTACCAGGTGGTATTGAATTTTAT  777
Bsep_S  721 AATGGGTTTATTTTTGATGAGGTTAGCGAGTTGCGATTGCCGAATGGCATTGAATTTTAT  780
Akaw_S  239   N  G  F  I  Y  T  E  V  S  E  G  R  L  P  G  G  I  E  F  Y  258
Clau_S  239   N  D  F  I  Y  T  E  V  S  E  G  R  L  P  G  G  I  E  F  Y  258
Pkil_S  239   N  G  F  I  Y  T  E  V  S  E  G  R  L  P  G  G  I  E  F  Y  258
Psoy_S  239   N  G  F  I  Y  T  E  V  S  E  G  R  L  P  G  G  I  E  F  Y  258
Vok     239   N  G  F  I  Y  T  E  V  S  E  G  R  L  P  G  G  I  E  F  Y  258
Cpac_S  240   H  G  F  I  Y  T  E  V  S  E  G  R  L  P  G  G  I  E  F  Y  259
Cfau_S  240   H  S  F  I  Y  T  E  V  S  E  G  R  L  P  G  G  I  E  F  Y  259
Cnau_S  240   H  G  F  I  Y  T  E  V  S  E  G  R  L  P  G  G  I  E  F  Y  259
Pste_S  240   Y  G  F  I  Y  T  E  V  S  E  G  R  L  P  G  G  I  E  F  Y  259
Rma     240   D  G  F  I  Y  T  E  V  S  E  G  R  L  P  S  G  I  E  F  Y  259
Ifos_S  240   N  G  F  I  Y  T  E  V  S  E  G  R  L  P  G  G  I  E  F  Y  259
Apha_S  240   N  G  F  I  Y  T  E  V  S  E  G  R  L  P  G  G  I  E  F  Y  259
Bsep_S  241   N  G  F  I  F  D  E  V  S  E  L  R  L  P  N  G  I  E  F  Y  260
```

```
Akaw_S  775 TTACCATTATTTTTTAACACAACAAATACCTTATTTGACTATCTAGCGAATAACACTATT  834
Clau_S  775 TTACCATTGTTTTTTCACACAACAAATACCTTATTTGACTATCTAGCGAATAACACTATT  834
Pkil_S  775 TTACCATTATTTTTTCACACAACAAATACCTTATTTGACTATCTAGCGAATAATACTATT  834
Psoy_S  775 TTACCATTATTTTTTCACACAACAAATACCTTATTTGACTATCTAGCGAATAATACTATT  834
Vok     775 TTACCATTGTTTTTTCACACAACAAATACCTTATTTGACTATCTAGCAGATAATACTATT  834
Cpac_S  778 TTACCACTATTTTTTAACACAACCAACACCTTGTTTGACTATCTAGTAAATAACGCCATG  837
Cfau_S  778 TTACCACTATTTTTTAACACAACCAACACCTTGTTTGACTATCTAGTAAATAACGCCGTG  837
Cnau_S  778 TTACCACTGTTTTTTAACACAACCAACACCTTGTTTGACTATCTAGTGAATAACGCTATG  837
Pste_S  778 TTACCACTGTTTTTTAACACAACCAACACCTTATTTGACTATCTAGTGAATAACGTCATA  837
Rma     778 TTACCACTCTTTTTTAACACAACCAATACCTTGTTTGATTATCTAGTGGATAACGCCATT  837
Ifos_S  778 TTACCGCTGTTTTTTAACACAACCAACACCTTGTTTGATTATCTAGTGGATAACACTATT  837
Apha_S  778 TTACCGCTGTTTTTTAACACAACCAACACCTTATTTGATTATCTAGTGGATAACACTATT  837
Bsep_S  781 TTATCATTATTTTTTACCCATACCAACAGCCTATTTGACTATTTGCCCAAGGATACAATT  840
Akaw_S  259   L  P  L  F  F  N  T  T  N  T  L  F  D  Y  L  A  N  N  T  I  278
Clau_S  259   L  P  L  F  F  H  T  T  N  T  L  F  D  Y  L  A  N  N  T  I  278
Pkil_S  259   L  P  L  F  F  H  T  T  N  T  L  F  D  Y  L  A  N  N  T  I  278
Psoy_S  259   L  P  L  F  F  H  T  T  N  T  L  F  D  Y  L  A  N  N  T  I  278
Vok     259   L  P  L  F  F  H  T  T  N  T  L  F  D  Y  L  A  D  N  T  I  278
Cpac_S  260   L  P  L  F  F  N  T  T  N  T  L  F  D  Y  L  V  N  N  A  M  279
Cfau_S  260   L  P  L  F  F  N  T  T  N  T  L  F  D  Y  L  V  N  N  A  V  279
Cnau_S  260   L  P  L  F  F  N  T  T  N  T  L  F  D  Y  L  V  N  N  A  M  279
Pste_S  260   L  P  L  F  F  N  T  T  N  T  L  F  D  Y  L  V  N  N  V  I  279
Rma     260   L  P  L  F  F  N  T  T  N  T  L  F  D  Y  L  V  D  N  A  I  279
Ifos_S  260   L  P  L  F  F  N  T  T  N  T  L  F  D  Y  L  V  D  N  T  I  279
Apha_S  260   L  P  L  F  F  N  T  T  N  T  L  F  D  Y  L  V  D  N  T  I  279
Bsep_S  261   L  S  L  F  F  T  H  T  N  S  L  F  D  Y  L  P  K  D  T  I  280
```

```
Akaw_S  835 ATTGCTACGTCAAAAGAATTTTCGAGCTTAGTAGACAAAACTTATAGCGAAATACGCGAA  894
Clau_S  835 ATTGCCACATCAAAAGAATTTTCGAGCTTAGTGGATAAAACTTATAGCGAAATATGTGAA  894
Pkil_S  835 ATTGCCACGTCAAAAAAATTTTCGAGCTTAGTGGATAAAACGTATAACGAAATACGTGAA  894
Psoy_S  835 ATTGCCACGTCAAAAAAATTTTCGAGCTTAGTGGATAAAACGTATAACGAAATACGTGAA  894
Vok     835 ATTGCCACGTCAAAAGAATTTTCGAACTTAGTGGATAAAACTTATAACGAAATACGTGAA  894
Cpac_S  838 ATTGCCACATCAAAAGGGTTCTCAAACTTGGTAGATAAGACCTATAGCGAGATACGTGAA  897
Cfau_S  838 ATTGCCACATCAAAAGGGTTTTCAAACTTGGTAGATAAGACCTATAGCGAGATACGCGAA  897
Cnau_S  838 ATTGCCACATCAAAAGGGTTTTCAACCTTAGTAGATAAGACTTATAGCGAGATACGTGAA  897
Pste_S  838 ATTGCCACATCAAAAGGGTTTTCAAACTTGGTAGATAAGACCTATAGCGAGATACGCGAA  897
Rma     838 ATTGTCACATCAAAAGGGTTTTCAAGTTTAGTGGATAAGACCTATAGCGAGATACATGAA  897
Ifos_S  838 ATTGTCATATCAAAAGGATTTTCAGACTTGGTGGATAAGACCTATAGCGAGATACGTGAA  897
Apha_S  838 GTTGCCACATCAAAAGGATTTTCAGACTTAGTGGATAAGACTTATAGCGAGATACGTGAA  897
Bsep_S  841 ATTGCCACCAGTCAAGGGTTTAGCGAGTTGTTAGAGGTAAATTATGACGAGATTGACGCG  900
Akaw_S  279   I  A  T  S  K  E  F  S  S  L  V  D  K  T  Y  S  E  I  R  E  298
Clau_S  279   I  A  T  S  K  E  F  S  S  L  V  D  K  T  Y  S  E  I  C  E  298
Pkil_S  279   I  A  T  S  K  K  F  S  S  L  V  D  K  T  Y  N  E  I  R  E  298
Psoy_S  279   I  A  T  S  K  K  F  S  S  L  V  D  K  T  Y  N  E  I  R  E  298
Vok     279   I  A  T  S  K  E  F  S  N  L  V  D  K  T  Y  N  E  I  R  E  298
Cpac_S  280   I  A  T  S  K  G  F  S  N  L  V  D  K  T  Y  S  E  I  R  E  299
Cfau_S  280   I  A  T  S  K  G  F  S  N  L  V  D  K  T  Y  S  E  I  R  E  299
Cnau_S  280   I  A  T  S  K  G  F  S  T  L  V  D  K  T  Y  S  E  I  R  E  299
Pste_S  280   I  A  T  S  K  G  F  S  N  L  V  D  K  T  Y  S  E  I  R  E  299
Rma     280   I  V  T  S  K  G  F  S  S  L  V  D  K  T  Y  S  E  I  H  E  299
Ifos_S  280   I  V  I  S  K  G  F  S  D  L  V  D  K  T  Y  S  E  I  R  E  299
Apha_S  280   V  A  T  S  K  G  F  S  D  L  V  D  K  T  Y  S  E  I  R  E  299
Bsep_S  281   I  A  T  S  Q  G  F  S  E  L  L  E  V  N  Y  D  E  I  D  A  300
```

```
Akaw_S  895 CGATTTAAAAATGCTAAAAAATCATTAGAAAGAGTACCACTTGACATTCAACGAGTTTTC  954
Clau_S  895 CGATTTAAAAATGCTAAAAAATCATTAGACAGAGCACCACTTGACATTCAACGAGTTTTC  954
Pkil_S  895 CGATTTGAAAATGCTAAAAAATCATTAGACAGAGCACCACTTGACATTCAACGAGTTTTC  954
Psoy_S  895 CGATTTGAAAATGCTAAAAAATCATTAGACAGAGCACCACTTGACATTCAACGAGTTTTC  954
Vok     895 CGATTTGAAAATGCTAAAAAATCATTAGACAGAGCACCACTTGACATTCAACGAGTTTTC  954
Cpac_S  898 CGATTTGAAAATGCCAAAAAAATATTAGACAGAGCGCCACTTGATATTCACCAAGTTTTC  957
Cfau_S  898 CGATTTGAAAATGCCAAAAAAACATTAGATAGAGCGCCACTTGATATTCACCAAGTTTTC  957
Cnau_S  898 CGATTTGAAAATGCTAAAAAAACATTAGACAGAGCACCGCTTGATATTCACCAAGTTTTC  957
Pste_S  898 CGATTTGAAAATGCCAAAAAAACATTAGACAGAGCACCGTTTGATATTCACCAAGTTTTC  957
Rma     898 CGATTTGAAAATGCTAAAAAATCATTAGATAGGGCGCCACTTGATATTCATAAAGTTTTT  957
Ifos_S  898 CGATTTGAGCGTGCTAAAAAATCATTAGATAGAGCACCCCTTGATATTCCCCAAGTTTTC  957
Apha_S  898 CGATTTGAAAATGCTAAAAAATCATTAGATAGAGCTCCACTTGATATTCCCCAAGTTTTC  957
Bsep_S  901 CGTCATCAGCAAGCGGAATGCAATTATGAGCGTCTGCCATTGCCGATTAATCAGGTATTT  960
Akaw_S  299   R  F  K  N  A  K  K  S  L  E  R  V  P  L  D  I  Q  R  V  F  318
Clau_S  299   R  F  K  N  A  K  K  S  L  D  R  A  P  L  D  I  Q  R  V  F  318
Pkil_S  299   R  F  E  N  A  K  K  S  L  D  R  A  P  L  D  I  Q  R  V  F  318
Psoy_S  299   R  F  E  N  A  K  K  S  L  D  R  A  P  L  D  I  Q  R  V  F  318
Vok     299   R  F  E  N  A  K  K  S  L  D  R  A  P  L  D  I  Q  R  V  F  318
Cpac_S  300   R  F  E  N  A  K  K  I  L  D  R  A  P  L  D  I  H  Q  V  F  319
Cfau_S  300   R  F  E  N  A  K  K  T  L  D  R  A  P  L  D  I  H  Q  V  F  319
Cnau_S  300   R  F  E  N  A  K  K  T  L  D  R  A  P  L  D  I  H  Q  V  F  319
Pste_S  300   R  F  E  N  A  K  K  T  L  D  R  A  P  F  D  I  H  Q  V  F  319
Rma     300   R  F  E  N  A  K  K  S  L  D  R  A  P  L  D  I  H  K  V  F  319
Ifos_S  300   R  F  E  R  A  K  K  S  L  D  R  A  P  L  D  I  P  Q  V  F  319
Apha_S  300   R  F  E  N  A  K  K  S  L  D  R  A  P  L  D  I  P  Q  V  F  319
Bsep_S  301   R  H  Q  Q  A  E  C  N  Y  E  R  L  P  L  P  I  N  Q  V  F  320
```

```
Akaw_S  955 CTATCCAAAGAGTTGTTCTTTAGTGAAATCAAAAAAAAATCACAGCTTATACTTAGTGCA 1014
Clau_S  955 CTATCCAAAGAGTTGCTCTTTAGTGAAGTCAAAAAAAAATCACAGCTTATCCTTAGTGCT 1014
Pkil_S  955 CTATCCAAAGAGTTGCTCTTTAGTGAAGTCAAAAAAAAATCACAAATTATAATTAGTACA 1014
Psoy_S  955 CTATCCAAAGAGTTGCTCTTTAGTGAAGTCAAAAAAAAATCACAAATTATAATTAGTACA 1014
Vok     955 CTATCCAAAGAGTTGCTCTTTAGTGAAGTCAAGAAAAAATCACAGATTATAATTAGTACA 1014
Cpac_S  958 CTGTCCAAAGAGTTGTTATTTAGTGAAATCAAGCAAAAATCACAACTCATCATTAGCACG 1017
Cfau_S  958 CTGTCCAAAGAGTTGTTGTTTAGTGAAATCAAGCAAAAATCACAACTCATCATTAGCACG 1017
Cnau_S  958 CTGTCCAAAGAGTTGTTGTTTAGTAAAATCAAGCAAAAATCACAACTCATCATTAGCACG 1017
Pste_S  958 CTGTCCAAAGAGTTGTTGTTTAGTGAAATCAAGCAAAAATCACAACTCATCATTAGCACG 1017
Rma     958 CTGTCCAAAGAATTACTATTTAGAGAAATCAAACAAAAATCACAGCATATCATTAGCACA 1017
Ifos_S  958 CTGTCCAAAGAATTGCTGTTTAGTGAAATTAAGAAAAAACCACAGCTCATCATCAGTACA 1017
Apha_S  958 CTGTCCAAAGAGTTGTTGTTTAGTGAAATCAAGCAAAAATCACAACTCATCATTAGCACG 1017
Bsep_S  961 GTGGATAAAGATGTGCTGTTTGGGCAGATAAAACAACGCCAGCAGCTGGTGACTGGCAGC 1020
Akaw_S  319   L  S  K  E  L  F  F  S  E  I  K  K  K  S  Q  L  I  L  S  A  338
Clau_S  319   L  S  K  E  L  L  F  S  E  V  K  K  K  S  Q  L  I  L  S  A  338
Pkil_S  319   L  S  K  E  L  L  F  S  E  V  K  K  K  S  Q  I  I  I  S  T  338
Psoy_S  319   L  S  K  E  L  L  F  S  E  V  K  K  K  S  Q  I  I  I  S  T  338
Vok     319   L  S  K  E  L  L  F  S  E  V  K  K  K  S  Q  I  I  I  S  T  338
Cpac_S  320   L  S  K  E  L  L  F  S  E  I  K  Q  K  S  Q  L  I  I  S  T  339
Cfau_S  320   L  S  K  E  L  L  F  S  E  I  K  Q  K  S  Q  L  I  I  S  T  339
Cnau_S  320   L  S  K  E  L  L  F  S  K  I  K  Q  K  S  Q  L  I  I  S  T  339
Pste_S  320   L  S  K  E  L  L  F  S  E  I  K  Q  K  S  Q  L  I  I  S  T  339
Rma     320   L  S  K  E  L  L  F  R  E  I  K  Q  K  S  Q  H  I  I  S  T  339
Ifos_S  320   L  S  K  E  L  L  F  S  E  I  K  K  K  P  Q  L  I  I  S  T  339
Apha_S  320   L  S  K  E  L  L  F  S  E  I  K  Q  K  S  Q  L  I  I  S  T  339
Bsep_S  321   V  D  K  D  V  L  F  G  Q  I  K  Q  R  Q  Q  L  V  T  G  S  340
```

```
Akaw_S 1015 TCCAAACTAGAAAATAAAAATCAGTATTTTAATTTTAAATCAAGCCTACCGCCCTCAGTA 1074
Clau_S 1015 TCTAAACTAGAAAATAAAAATCAGTATTTTAATTTTAACTCAAGCCTATCACCGTCAGTA 1074
Pkil_S 1015 TTTAAACTAGAAAATAAAAATCAGCATTTTAATTTTAACTCAAGCCTACCACCACCAGTA 1074
Psoy_S 1015 TTTAAACTAGAAAATAAAAATCAGCATTTTAATTTTAACTCAAGCCTACCACCACCAGTA 1074
Vok    1015 TCTAAACTAGAAAATAAAAAGCAACATTTTAATTTTAACTCAAGTCTACCACCGCCAGTA 1074
Cpac_S 1018 TCAAAACTAGAAAACAAAAATCAACACTTTAATTTTAATTCAAGCCTATTGCCTCCCATA 1077
Cfau_S 1018 TCAAAACTAGAAGAAAAAAACCAACACTTTAATTTTAATTCAAGCCTATTGTCTCCCATA 1077
Cnau_S 1018 TCAAAACTAGAAGACAAAAACCAACACTTTAATTTTAATTCAAGCCTATTGCCTCCCATA 1077
Pste_S 1018 TCAAAACTAGAAGACAAAAACCAACACTTTAATTTTAATTCAAGCCTATTGCCTCCCATA 1077
Rma    1018 TCAAAACTAGAAAATAAAAACCAGAATTTTAATTTTAACTCAAGCTTATTGCCCTCAATA 1077
Ifos_S 1018 TCAAAACTAGAAGATAAAAACCAACACTTTAATTTCAACTCAAGCTTATTACCCCCAATA 1077
Apha_S 1018 TCAAAACTAGAAAATAAAAACCAACACTTTAATTTTAATTCAAGCCTATTGCCTTCCATA 1077
Bsep_S 1021 TCGAAACAGGAAGAGAAAGTTGGGCGCTTGAATTTTGGTAGTCAGTTGTTGCCACCGCTG 1080
Akaw_S  339   S  K  L  E  N  K  N  Q  Y  F  N  F  K  S  S  L  P  P  S  V  358
Clau_S  339   S  K  L  E  N  K  N  Q  Y  F  N  F  N  S  S  L  S  P  S  V  358
Pkil_S  339   F  K  L  E  N  K  N  Q  H  F  N  F  N  S  S  L  P  P  P  V  358
Psoy_S  339   F  K  L  E  N  K  N  Q  H  F  N  F  N  S  S  L  P  P  P  V  358
Vok     339   S  K  L  E  N  K  K  Q  H  F  N  F  N  S  S  L  P  P  P  V  358
Cpac_S  340   S  K  L  E  N  K  N  Q  H  F  N  F  N  S  S  L  L  P  P  I  359
Cfau_S  340   S  K  L  E  E  K  N  Q  H  F  N  F  N  S  S  L  L  S  P  I  359
Cnau_S  340   S  K  L  E  D  K  N  Q  H  F  N  F  N  S  S  L  L  P  P  I  359
Pste_S  340   S  K  L  E  D  K  N  Q  H  F  N  F  N  S  S  L  L  P  P  I  359
Rma     340   S  K  L  E  N  K  N  Q  N  F  N  F  N  S  S  L  L  P  S  I  359
Ifos_S  340   S  K  L  E  D  K  N  Q  H  F  N  F  N  S  S  L  L  P  P  I  359
Apha_S  340   S  K  L  E  N  K  N  Q  H  F  N  F  N  S  S  L  L  P  S  I  359
Bsep_S  341   S  K  Q  E  E  K  V  G  R  L  N  F  G  S  Q  L  L  P  P  L  360
```

```
Akaw_S 1075 CGCATTGAACTACAAACAAACAATCCGTTAAGAAAATTTTCAAAATTTGTTAAAAAATTT 1134
Clau_S 1075 CGCATTGAACTACAAACAAATAATCCGTTAAGCAAATTTTCAACATTTGTTGAAAAATTT 1134
Pkil_S 1075 CGCATTGAACTACAAACAAACAATCCGTTAAGAAAATTTTCAACATTTGTTAAAAGATTT 1134
Psoy_S 1075 CGCATTGAACTACAAACAAACAATCCGTTAAGAAAATTTTCAACATTTGTTAAAAGATTT 1134
Vok    1075 CGCATTGAACTACAAACGAACAATCCGTTAAGCAAATTTTCAACATTTGTTAAAAGATTT 1134
Cpac_S 1078 TGCATTGAGCCACAAACAAAGAATCCATTAAACAAGTTTTCAGTATTTGTTAAAAGATTT 1137
Cfau_S 1078 CGCATTGAGCCAAAAACAAAAAATCCATTAAACAAATTTTTAATATTTGTTAAAAGATTT 1137
Cnau_S 1078 CGCATTGAGCCACAAACAAAAAATCCATTAAACAAATTTTTAGCATTTATTAAAAGATTT 1137
Pste_S 1078 CGCATTGAGCCACAAACAAAAAACCCATTAAACAAATTTTCAGCATTTGTTAAAAGATTT 1137
Rma    1078 CGTATTGAACCACAAACAAAAAACCCATTAAGAAAATTTTTAGCCTTTGTTAAAAAATTC 1137
Ifos_S 1078 CGCATTGAACCACAAACGAAAAACCCATTAAGCAAATTTTCAGCTTTTGTTAAAAGATTT 1137
Apha_S 1078 CACATTGAGCCACAAACAAAAAATCCATTAAGCAAATTTTCAGCATTTGTTAAAAGATTT 1137
Bsep_S 1081 ACGATTAGTGCGCAAAATAAAAATCCACTGAGTAAATTTCTAAATTTTGAGAAAAAGTTT 1140
Akaw_S  359   R  I  E  L  Q  T  N  N  P  L  R  K  F  S  K  F  V  K  K  F  378
Clau_S  359   R  I  E  L  Q  T  N  N  P  L  S  K  F  S  T  F  V  E  K  F  378
Pkil_S  359   R  I  E  L  Q  T  N  N  P  L  R  K  F  S  T  F  V  K  R  F  378
Psoy_S  359   R  I  E  L  Q  T  N  N  P  L  R  K  F  S  T  F  V  K  R  F  378
Vok     359   R  I  E  L  Q  T  N  N  P  L  S  K  F  S  T  F  V  K  R  F  378
Cpac_S  360   C  I  E  P  Q  T  K  N  P  L  N  K  F  S  V  F  V  K  R  F  379
Cfau_S  360   R  I  E  P  K  T  K  N  P  L  N  K  F  L  I  F  V  K  R  F  379
Cnau_S  360   R  I  E  P  Q  T  K  N  P  L  N  K  F  L  A  F  I  K  R  F  379
Pste_S  360   R  I  E  P  Q  T  K  N  P  L  N  K  F  S  A  F  V  K  R  F  379
Rma     360   R  I  E  P  Q  T  K  N  P  L  R  K  F  L  A  F  V  K  K  F  379
Ifos_S  360   R  I  E  P  Q  T  K  N  P  L  S  K  F  S  A  F  V  K  R  F  379
Apha_S  360   H  I  E  P  Q  T  K  N  P  L  S  K  F  S  A  F  V  K  R  F  379
Bsep_S  361   T  I  S  A  Q  N  K  N  P  L  S  K  F  L  N  F  E  K  K  F  380
```

```
Akaw_S 1135 ACTAACAAGCAAAACAAAAAAATATTAATTGTTTGTAAATCACTCGATAGACAAAACGTA 1194
Clau_S 1135 ACTAACCAGCAAAACAAAAAAATATTAATTGTTTGTGAATCACTCGATAGACAAAATGTA 1194
Pkil_S 1135 ACCAACAAACCAAACAAAAAAATATTAATTGTTTGTGAATCGCTCGATAGACAAAATGTA 1194
Psoy_S 1135 ACCAACAAACCAAACAAAAAAATATTAATTGTTTGTGAATCGCTCGATAGACAAAATGTA 1194
Vok    1135 ACTAACAAACAAAACAAAAAAATATTAATTGTTTGTGAATCACTCGATAGACAAAATGTA 1194
Cpac_S 1138 ACCGACAAGCAAAGCAAGAAAATATTAATTGTTTGTGAGTCGCGCGGCAGGCAAGATGTG 1197
Cfau_S 1138 ACCAACAAGAAAAGCAAAAAAATATTAATTGTTTGTGAGTCGTACGGCAGGCAAGATGTG 1197
Cnau_S 1138 AACAACAAGCAAAGTAAAAAAATATTAATTGTTTGTGAATCGCGCGGCAGGCAAGATGTG 1197
Pste_S 1138 ACCAACAAGCGAAGTAAAAAAATATTAATTGTTTGTGAGTCGCGTGGCAGGCAAGATGTG 1197
Rma    1138 ACTAAC---------AAAAAAATATTAGTTGTTTGTGAATCGCGTAGCAGGCAAAATGTG 1188
Ifos_S 1138 ACCAAC---------AAAAAAATATTAGTTGTTTGTGAATCGCGTGGCAGGCAAGATGTG 1188
Apha_S 1138 ACCAACAAGCAAAGCAAAAAAATATTAATTGTTTGTGGGTCGCGTGGTAGGCAAGATGTG 1197
Bsep_S 1141 ACAGGG------------AAAATCCTGATTGTTTGCGAGTCCGAAGGTAGGCAGAGTGTG 1188
Akaw_S  379   T  N  K  Q  N  K  K  I  L  I  V  C  K  S  L  D  R  Q  N  V  398
Clau_S  379   T  N  Q  Q  N  K  K  I  L  I  V  C  E  S  L  D  R  Q  N  V  398
Pkil_S  379   T  N  K  P  N  K  K  I  L  I  V  C  E  S  L  D  R  Q  N  V  398
Psoy_S  379   T  N  K  P  N  K  K  I  L  I  V  C  E  S  L  D  R  Q  N  V  398
Vok     379   T  N  K  Q  N  K  K  I  L  I  V  C  E  S  L  D  R  Q  N  V  398
Cpac_S  380   T  D  K  Q  S  K  K  I  L  I  V  C  E  S  R  G  R  Q  D  V  399
Cfau_S  380   T  N  K  K  S  K  K  I  L  I  V  C  E  S  Y  G  R  Q  D  V  399
Cnau_S  380   N  N  K  Q  S  K  K  I  L  I  V  C  E  S  R  G  R  Q  D  V  399
Pste_S  380   T  N  K  R  S  K  K  I  L  I  V  C  E  S  R  G  R  Q  D  V  399
Rma     380   T  N  #  #  #  K  K  I  L  V  V  C  E  S  R  S  R  Q  N  V  396
Ifos_S  380   T  N  #  #  #  K  K  I  L  V  V  C  E  S  R  G  R  Q  D  V  396
Apha_S  380   T  N  K  Q  S  K  K  I  L  I  V  C  G  S  R  G  R  Q  D  V  399
Bsep_S  381   T  G  #  #  #  #  K  I  L  I  V  C  E  S  E  G  R  Q  S  V  396
```

```
Akaw_S 1195 CTCAGTGAATTACTAATCAATCATAATCTTAATACTTATAGCGTTAAGAGCTGGCATGAA 1254
Clau_S 1195 CTCAATGATTTACTTATCGATAATAATCTTGATACTTATAGCGTTAAGAGCTGGCATGAA 1254
Pkil_S 1195 CTCAGTGATTTACTTATCAATCATAATCTTGATACTTGTAGCGTTAAGAGCTGGCATGAA 1254
Psoy_S 1195 CTCAGTGATTTACTTATCAATCATAATCTTGATACTTGTAGCGTTAAGAGCTGGCATGAA 1254
Vok    1195 CTAAGTGATTTACTCATCAATCATAATCTTAATACTTATAGCGTTAAGAGCTGGCATGAA 1254
Cpac_S 1198 CTCAGCGATTTACTCATCAGTCATAACCTTGATATTCATAGTGTTAAAAACTGGCATGAA 1257
Cfau_S 1198 CTCAGCGATTTACTCATCAAGCATAACCTTGATATTCATAGTGTTAAAAACTGGCATGAA 1257
Cnau_S 1198 CTCAGCGATTTACTCATCAGTCATAACCTTGATATTCATAGTGTTAAAAACTGGAATGAA 1257
Pste_S 1198 CTCAGCGATTTACTCATCAGCCATAACCTTGATATTCATAGTGTTAAAAACTGGCATGAA 1257
Rma    1189 CTCAGAGATTTATTCACTAGTCATCAACTTGATACCCATAACGTTAAAAACTGGTACGAA 1248
Ifos_S 1189 CTCAGCGATTTGTTCACCAGCCATCAACTTGATACCCATAGCGTTAAAAACTGGCGTGAA 1248
Apha_S 1198 CTCAGTGATTTACTCATCAGCCATAACCTTGATGTTCATAGTGTTAAAAACTGGCATGAA 1257
Bsep_S 1189 TTGAGTGATTTTCTCATTAATCACAACCGCAATCCGCTGCCAGTGGCACATTGGCAAGAT 1248
Akaw_S  399   L  S  E  L  L  I  N  H  N  L  N  T  Y  S  V  K  S  W  H  E  418
Clau_S  399   L  N  D  L  L  I  D  N  N  L  D  T  Y  S  V  K  S  W  H  E  418
Pkil_S  399   L  S  D  L  L  I  N  H  N  L  D  T  C  S  V  K  S  W  H  E  418
Psoy_S  399   L  S  D  L  L  I  N  H  N  L  D  T  C  S  V  K  S  W  H  E  418
Vok     399   L  S  D  L  L  I  N  H  N  L  N  T  Y  S  V  K  S  W  H  E  418
Cpac_S  400   L  S  D  L  L  I  S  H  N  L  D  I  H  S  V  K  N  W  H  E  419
Cfau_S  400   L  S  D  L  L  I  K  H  N  L  D  I  H  S  V  K  N  W  H  E  419
Cnau_S  400   L  S  D  L  L  I  S  H  N  L  D  I  H  S  V  K  N  W  N  E  419
Pste_S  400   L  S  D  L  L  I  S  H  N  L  D  I  H  S  V  K  N  W  H  E  419
Rma     397   L  R  D  L  F  T  S  H  Q  L  D  T  H  N  V  K  N  W  Y  E  416
Ifos_S  397   L  S  D  L  F  T  S  H  Q  L  D  T  H  S  V  K  N  W  R  E  416
Apha_S  400   L  S  D  L  L  I  S  H  N  L  D  V  H  S  V  K  N  W  H  E  419
Bsep_S  397   L  S  D  F  L  I  N  H  N  R  N  P  L  P  V  A  H  W  Q  D  416
```

```
Akaw_S 1255 TTTACTACTAGCAACAAATCGCTTAACATTCTCCATGAGAAACTTATCCATGGTTTTCTT 1314
Clau_S 1255 TTTACTACTAGTAGTAAATCGCTTAATATTATCCATGATAATCTTATCCATGGTTTGCTT 1314
Pkil_S 1255 TTTACTACTAGTAGTAAATCGCTTAACATTATCCATGAGAATCTTATCCATGGTTTACTT 1314
Psoy_S 1255 TTTACTACTAGTAGTAAATCGCTTAACATTATCCATGAGAATCTTATCCATGGTTTACTT 1314
Vok    1255 TTTACTACTAGTAGTAAATCACTTAACATTATCCATGAAGATCTTATCCATGGTTTGCTT 1314
Cpac_S 1258 TTTAGCAAGAGTAACAAGCCGCTTAACATTACCCATGAAGACCTTACCCATGGCTTATTC 1317
Cfau_S 1258 TTTAGCAAGAGTAACAAGCCACTTAACATTACCCATGAAGACCTTACCCATGGCTTATTC 1317
Cnau_S 1258 TTTAGCAAGAGTAGCAAACCACTTAACATTACCCATAAAAACCTTACCCATAGCTTATTC 1317
Pste_S 1258 TTTAGCAAGAGTAGCAAGCCACTTAACATTACCCATGAAAACCTTATCCATGGCTTATTC 1317
Rma    1249 TTTAGTAAAAGTAGCAAACTGCTTAACATTACTAATGAAAATCTTACCAATGGCTTACTC 1308
Ifos_S 1249 TTTAATAAGAGCAGCAAACTGCTCAATATTACCCATGAAAATCTTGCCCATGGCTTGCTC 1308
Apha_S 1258 TTTAGCAAAAATAACAAGCCACTTAACATTACCCATGAAAACATTACCCATGGCTTATTC 1317
Bsep_S 1249 TTTATTGAGAGCGAGCAAAGCCTTTGCATTACCAATGATGAGCTGACCGAAGGCTTGCTA 1308
Akaw_S  419   F  T  T  S  N  K  S  L  N  I  L  H  E  K  L  I  H  G  F  L  438
Clau_S  419   F  T  T  S  S  K  S  L  N  I  I  H  D  N  L  I  H  G  L  L  438
Pkil_S  419   F  T  T  S  S  K  S  L  N  I  I  H  E  N  L  I  H  G  L  L  438
Psoy_S  419   F  T  T  S  S  K  S  L  N  I  I  H  E  N  L  I  H  G  L  L  438
Vok     419   F  T  T  S  S  K  S  L  N  I  I  H  E  D  L  I  H  G  L  L  438
Cpac_S  420   F  S  K  S  N  K  P  L  N  I  T  H  E  D  L  T  H  G  L  F  439
Cfau_S  420   F  S  K  S  N  K  P  L  N  I  T  H  E  D  L  T  H  G  L  F  439
Cnau_S  420   F  S  K  S  S  K  P  L  N  I  T  H  K  N  L  T  H  S  L  F  439
Pste_S  420   F  S  K  S  S  K  P  L  N  I  T  H  E  N  L  I  H  G  L  F  439
Rma     417   F  S  K  S  S  K  L  L  N  I  T  N  E  N  L  T  N  G  L  L  436
Ifos_S  417   F  N  K  S  S  K  L  L  N  I  T  H  E  N  L  A  H  G  L  L  436
Apha_S  420   F  S  K  N  N  K  P  L  N  I  T  H  E  N  I  T  H  G  L  F  439
Bsep_S  417   F  I  E  S  E  Q  S  L  C  I  T  N  D  E  L  T  E  G  L  L  436
```

```
Akaw_S 1315 ATCCATAATATTGCCATTATTACTGAAGAAGATTTATTTGGACAAGAAGTAGTTCAACAA 1374
Clau_S 1315 ATCCATAATATTGCTATTATTACTGAAGAAGATTTATTTGGACAAGAAGTAGTTCAACAA 1374
Pkil_S 1315 ATCCATAATATTGCTATTATTACTGAAGAAGATTTATTTGGACAAGAAGTAGTTCAACAA 1374
Psoy_S 1315 ATCCATAATATTGCTATTATTACTGAAGAAGATTTATTTGGACAAGAAGTAGTTCAACAA 1374
Vok    1315 ATCCATAATATTGCTATTATTACTGAAGAAGATTTATTTGGGCAAGAAGTAGTTCAACAA 1374
Cpac_S 1318 ACTAATGATATCGCCATTGTTACTGAAGAGGATTTATTCGGTCAAGAAGTGGTTCAGCAA 1377
Cfau_S 1318 ACTAATGATATCGCCATTGTTACTGAAGAGGATTTATTCGGTCAAGAAGTGGTTCAGCAA 1377
Cnau_S 1318 ACTAATGATATCGCCATTGTTACTGAAGAGGATTTATTCGGTCAAGAAGTGGTTCAGCAA 1377
Pste_S 1318 ACTAATGATATCGCCATTGTTACTGAAGAGGATTTATTCGGTCAAGAAGTGGTTCAGCAA 1377
Rma    1309 ACTGATGATATTGCCATTATTACTGAAGAGAATTTATTCGGTCAAGAAGTGGTTCAACAA 1368
Ifos_S 1309 ACTAGTGATATTGCCATTGTTACTGAAGAAGATTTATTCGGTCAAGAAGTGGTTCAGCAA 1368
Apha_S 1318 ACTAATGGTATCGCCATTGTTACTGAAGAGGATTTATTCGGTCAAGAAGTGGTTCAGCAA 1377
Bsep_S 1309 ACTAAGGACATTGCGGTCATTACCGAAGTCAATTTGTTTGGCGCTGATGTGGTTAAACAG 1368
Akaw_S  439   I  H  N  I  A  I  I  T  E  E  D  L  F  G  Q  E  V  V  Q  Q  458
Clau_S  439   I  H  N  I  A  I  I  T  E  E  D  L  F  G  Q  E  V  V  Q  Q  458
Pkil_S  439   I  H  N  I  A  I  I  T  E  E  D  L  F  G  Q  E  V  V  Q  Q  458
Psoy_S  439   I  H  N  I  A  I  I  T  E  E  D  L  F  G  Q  E  V  V  Q  Q  458
Vok     439   I  H  N  I  A  I  I  T  E  E  D  L  F  G  Q  E  V  V  Q  Q  458
Cpac_S  440   T  N  D  I  A  I  V  T  E  E  D  L  F  G  Q  E  V  V  Q  Q  459
Cfau_S  440   T  N  D  I  A  I  V  T  E  E  D  L  F  G  Q  E  V  V  Q  Q  459
Cnau_S  440   T  N  D  I  A  I  V  T  E  E  D  L  F  G  Q  E  V  V  Q  Q  459
Pste_S  440   T  N  D  I  A  I  V  T  E  E  D  L  F  G  Q  E  V  V  Q  Q  459
Rma     437   T  D  D  I  A  I  I  T  E  E  N  L  F  G  Q  E  V  V  Q  Q  456
Ifos_S  437   T  S  D  I  A  I  V  T  E  E  D  L  F  G  Q  E  V  V  Q  Q  456
Apha_S  440   T  N  G  I  A  I  V  T  E  E  D  L  F  G  Q  E  V  V  Q  Q  459
Bsep_S  437   T  K  D  I  A  V  I  T  E  V  N  L  F  G  A  D  V  V  K  Q  456
```

```
Akaw_S 1375 ---CGCCAT---TATGCTAAACATAAAGATTTTGACGTGGGCATTACAAGTTTAGAAGAG 1428
Clau_S 1375 CAACGTCGT---CATGCCAAACATAAAGATTTTGACGTGGCTATTAAAAGCTTAGTAGAG 1431
Pkil_S 1375 CAACGTCGT---CATACCAAACATAAAGATTTTGACATAGCTATTAAGAGCTTAATAGAA 1431
Psoy_S 1375 CAACGTCGT---CATACCAAACATAAAGATTTTGACATAGCTATTAAGAGCTTAATAGAA 1431
Vok    1375 CAACGTCGT---CATACCAAACATAAATATTTTGACGTAGCTATTAAGAGCTTAGTAGAG 1431
Cpac_S 1378 CAGCGCCGTCGCCGTGCTAAACATAAAGATTTTGATGAGGCAATTAAAAGCTTGGTTGAG 1437
Cfau_S 1378 CAGCGCCGTCGCCGTGCCAAACATAAAGATTTTGATGAGGCAATTAAAAGCTTGGTTGAG 1437
Cnau_S 1378 CAGCGCCGGCACCGTGCCAAACATAAAGATTTTGATGAGGCAATTAAAAGCTTGGTTGAG 1437
Pste_S 1378 CAGCGCCGTCGCCGTGCCAAACATAAAAATTTTGATGAGGCAATTAAAAGCTTGATTGAG 1437
Rma    1369 CAACGTCGT---CGTGTCAAGCACAAAGATTTTGATGAAGCAATTAAAAGCCTAGTTGAG 1425
Ifos_S 1369 CAGCGTCGT---CGTGCTAATCACAAAGATTTTGATGAAGCAATTAAAAGCCTAGTTGAA 1425
Apha_S 1378 CAGCGCCGTCGCCGTGCCAAACATAAAGATTTTAATGAGGCAATTAAAAGCCTAGTTGAA 1437
Bsep_S 1369 CAACGACGCAGACGGGCGAAGCATAAGGATTTTGATGAGGCGATTAAGAGCCTGGTGGAA 1428
Akaw_S  459   #  R  H  #  Y  A  K  H  K  D  F  D  V  G  I  T  S  L  E  E  476
Clau_S  459   Q  R  R  #  H  A  K  H  K  D  F  D  V  A  I  K  S  L  V  E  477
Pkil_S  459   Q  R  R  #  H  T  K  H  K  D  F  D  I  A  I  K  S  L  I  E  477
Psoy_S  459   Q  R  R  #  H  T  K  H  K  D  F  D  I  A  I  K  S  L  I  E  477
Vok     459   Q  R  R  #  H  T  K  H  K  Y  F  D  V  A  I  K  S  L  V  E  477
Cpac_S  460   Q  R  R  R  R  A  K  H  K  D  F  D  E  A  I  K  S  L  V  E  479
Cfau_S  460   Q  R  R  R  R  A  K  H  K  D  F  D  E  A  I  K  S  L  V  E  479
Cnau_S  460   Q  R  R  H  R  A  K  H  K  D  F  D  E  A  I  K  S  L  V  E  479
Pste_S  460   Q  R  R  R  R  A  K  H  K  N  F  D  E  A  I  K  S  L  I  E  479
Rma     457   Q  R  R  #  R  V  K  H  K  D  F  D  E  A  I  K  S  L  V  E  475
Ifos_S  457   Q  R  R  #  R  A  N  H  K  D  F  D  E  A  I  K  S  L  V  E  475
Apha_S  460   Q  R  R  R  R  A  K  H  K  D  F  N  E  A  I  K  S  L  V  E  479
Bsep_S  457   Q  R  R  R  R  A  K  H  K  D  F  D  E  A  I  K  S  L  V  E  476
```

CarD-like/TRCF domain (475-560)

```
Akaw_S 1429 ATTAAAATAGGCGATGCTATTGTGCATGAAAATTATGGCGTAGGTAGATATTTAGGACTC 1488
Clau_S 1432 ATTAAAATAGGTGATGCGATTGTGCATGAAAATTATGGCGTGGGTAGATATTTAGGGCTC 1491
Pkil_S 1432 ATTAAAATAGGCGATGCTATTGTGCATGAAAATTATGGCGTGGGTAGATATTTAGGGCTC 1491
Psoy_S 1432 ATTAAAATAGGCGATGCTATTGTGCATGAAAATTATGGCGTGGGTAGATATTTAGGGCTC 1491
Vok    1432 ATTAAAATAGGCGATGCTATTGTGCATGAAAATTATGGCGTGGGTAGATATTTAGGGCTC 1491
Cpac_S 1438 ATTAAAATAGGTGATGCTATTGTGCATGAAAACTATGGCGTGGGCAAATATTTAGGGCTT 1497
Cfau_S 1438 ATTAGAATGGATGATGCTATTGTGCATGAAAACTATGGTGTGGGCAGATATTTAGGGCTT 1497
Cnau_S 1438 ATTAAAATGGGTGATGCTATTGTGCATGAAAACTATGGTGTGGGAAGATATTTAGGGCTT 1497
Pste_S 1438 ATTAAAATGGGTGATGCTATTGTGCATGAAAACTATGGTGTGGGCAGATATTTAGGGCTT 1497
Rma    1426 ATTAAAATGGGTGATGCTATTGTGCATGAAAATTATGGTGTAGGTAGGTATTTAGGGCTT 1485
Ifos_S 1426 ATTAAAATGGGTGATGCGATTGTGCATGAAAACTATGGTGTGGGTAGGTATTTAGGGCTT 1485
Apha_S 1438 ATTAAAATGGGTGATGCGATTGTGCATGAAAACTATGGTGTGGGCAGATATTTAGGGCTT 1497
Bsep_S 1429 GTCCAAATCGGCGACCCAATTGTGCATGAGAGTTATGGTGTGGGGCGTTATTTGGGTTTG 1488
Akaw_S  477   I  K  I  G  D  A  I  V  H  E  N  Y  G  V  G  R  Y  L  G  L  496
Clau_S  478   I  K  I  G  D  A  I  V  H  E  N  Y  G  V  G  R  Y  L  G  L  497
Pkil_S  478   I  K  I  G  D  A  I  V  H  E  N  Y  G  V  G  R  Y  L  G  L  497
Psoy_S  478   I  K  I  G  D  A  I  V  H  E  N  Y  G  V  G  R  Y  L  G  L  497
Vok     478   I  K  I  G  D  A  I  V  H  E  N  Y  G  V  G  R  Y  L  G  L  497
Cpac_S  480   I  K  I  G  D  A  I  V  H  E  N  Y  G  V  G  K  Y  L  G  L  499
Cfau_S  480   I  R  M  D  D  A  I  V  H  E  N  Y  G  V  G  R  Y  L  G  L  499
Cnau_S  480   I  K  M  G  D  A  I  V  H  E  N  Y  G  V  G  R  Y  L  G  L  499
Pste_S  480   I  K  M  G  D  A  I  V  H  E  N  Y  G  V  G  R  Y  L  G  L  499
Rma     476   I  K  M  G  D  A  I  V  H  E  N  Y  G  V  G  R  Y  L  G  L  495
Ifos_S  476   I  K  M  G  D  A  I  V  H  E  N  Y  G  V  G  R  Y  L  G  L  495
Apha_S  480   I  K  M  G  D  A  I  V  H  E  N  Y  G  V  G  R  Y  L  G  L  499
Bsep_S  477   V  Q  I  G  D  P  I  V  H  E  S  Y  G  V  G  R  Y  L  G  L  496
```

CarD-like/TRCF domain (475-560)

```
Akaw_S 1489 AAAACCAAAATTTTTGATAGACAATCACAAGATTTTCTTGTCCTAAAATATGCCAATAAC 1548
Clau_S 1492 AAAACCAAAATTTTTGATAAACAATCACAAGATTTTCTTGTCCTAAAATATGCCAATAAC 1551
Pkil_S 1492 AAAACCAAAATTTTTGATAGACAATCACAAGATTTTCTTGTCCTAAAATATGCCAATAAC 1551
Psoy_S 1492 AAAACCAAAATTTTTGATAGACAATCACAAGATTTTCTTGTCCTAAAATATGCCAATAAC 1551
Vok    1492 AAAACCAAAATTTTTGATAAACAATCACAAGATTTTCTTGTCCTAAAATATGCCAATAAC 1551
Cpac_S 1498 AAAACCCAAATTTTTGATGGTCAATCACAAGATTTTCTTGTCCTGGAATATGCTGATAAG 1557
Cfau_S 1498 AAAACCCAAATCTTTGATGGTCAATCACAAGATTTTCTTGCCCTGGAATATGCTGATAAG 1557
Cnau_S 1498 AAAACCCAAACCTTTGATGGTCAATCACAAGATTTTCTTGCCCTGGAATATGCTGATAAG 1557
Pste_S 1498 AAAACCCAAACCTTTGATGGTCAATCACAAGATTTTCTTACCCTGGAATATGCTGATAAG 1557
Rma    1486 AAACCCCAAATCTTTGATGATCAATCACAAGATTTTCTTACCCTAGAATATGCTGATAAT 1545
Ifos_S 1486 AAAACCCAAACTTTTGATGGACAATCACAAGATTTCCTTGCCCTAGAATATGCTAATAAC 1545
Apha_S 1498 AAAACCCAAACCTTTGATGGACAATCACAAGATTTTATTGCCCTAGAATATGCTGATAAC 1557
Bsep_S 1489 AAAACACAGACTTTTGATGAAATGACGCAAGATTTTTTGGTGTTAGAGTATGCTGATGAA 1548
Akaw_S  497   K  T  K  I  F  D  R  Q  S  Q  D  F  L  V  L  K  Y  A  N  N  516
Clau_S  498   K  T  K  I  F  D  K  Q  S  Q  D  F  L  V  L  K  Y  A  N  N  517
Pkil_S  498   K  T  K  I  F  D  R  Q  S  Q  D  F  L  V  L  K  Y  A  N  N  517
Psoy_S  498   K  T  K  I  F  D  R  Q  S  Q  D  F  L  V  L  K  Y  A  N  N  517
Vok     498   K  T  K  I  F  D  K  Q  S  Q  D  F  L  V  L  K  Y  A  N  N  517
Cpac_S  500   K  T  Q  I  F  D  G  Q  S  Q  D  F  L  V  L  E  Y  A  D  K  519
Cfau_S  500   K  T  Q  I  F  D  G  Q  S  Q  D  F  L  A  L  E  Y  A  D  K  519
Cnau_S  500   K  T  Q  T  F  D  G  Q  S  Q  D  F  L  A  L  E  Y  A  D  K  519
Pste_S  500   K  T  Q  T  F  D  G  Q  S  Q  D  F  L  T  L  E  Y  A  D  K  519
Rma     496   K  P  Q  I  F  D  D  Q  S  Q  D  F  L  T  L  E  Y  A  D  N  515
Ifos_S  496   K  T  Q  T  F  D  G  Q  S  Q  D  F  L  A  L  E  Y  A  N  N  515
Apha_S  500   K  T  Q  T  F  D  G  Q  S  Q  D  F  I  A  L  E  Y  A  D  N  519
Bsep_S  497   K  T  Q  T  F  D  E  M  T  Q  D  F  L  V  L  E  Y  A  D  E  516
```

CarD-like/TRCF domain (475-560)

```
Akaw_S 1549 GCAAAACTAATGGTACCAATCATCTCATTTAATCTAATTTCTAGATATGCTGGAATTTCA 1608
Clau_S 1552 GCAAAATTAATGGTGCCAATCATCTCATTTAATCTAATCTCTAGATATGCTGGAATTTCA 1611
Pkil_S 1552 GCAAAATTAATGGTGCCAATAATCTCATTTAATCTAATCTCTAGATATGCTGGAATTTCG 1611
Psoy_S 1552 GCAAAATTAATGGTGCCAATAATCTCATTTAATCTAATCTCTAGATATGCTGGAATTTCG 1611
Vok    1552 GCAAAATTAATGGTGCCAATCATCTCATTTAATCTAATCTCTAGATATGCTGGAATTTCG 1611
Cpac_S 1558 GCAAAGTTAATGGTGCCAATCACTTCGCTTAATTTAATCTCTAGGTACGCTGGCATTTCA 1617
Cfau_S 1558 GCAAAATTAATGGTGCCAATCACCTCGCTTAATTTAATCTCTAGGTACGCTGGCATTTCA 1617
Cnau_S 1558 GCAAAGTTAATGGTGCCAATCACCTCGCTTAATCTAATCTCTAGGTACGCTGGCATTTCA 1617
Pste_S 1558 GCAAAGTTAATGGTGCCAATCACCTCGCTTAATTTAATCTCTAGGTACGCTGGTATTTCA 1617
Rma    1546 GCAAAATTAATGGTGCCAATAACCTCACTCAACCTAATCTCCAGATACGCTGGAATCTCA 1605
Ifos_S 1546 GCAAAGTTGATGGTACCAATCACCTCGCTTAATCTAATCTCCAGATATGCTGGTATTTCA 1605
Apha_S 1558 GCAAAGTTAATGGTACCAATCACCTCGCTTAATTTAATCTCTAGGTACGCTGGCATTTCA 1617
Bsep_S 1549 TCTAAATTAATGGTGCCGATGACTTCGTTGAATCTGATTTCTCGCTATTCGGGGGCATCG 1608
Akaw_S  517   A  K  L  M  V  P  I  I  S  F  N  L  I  S  R  Y  A  G  I  S  536
Clau_S  518   A  K  L  M  V  P  I  I  S  F  N  L  I  S  R  Y  A  G  I  S  537
Pkil_S  518   A  K  L  M  V  P  I  I  S  F  N  L  I  S  R  Y  A  G  I  S  537
Psoy_S  518   A  K  L  M  V  P  I  I  S  F  N  L  I  S  R  Y  A  G  I  S  537
Vok     518   A  K  L  M  V  P  I  I  S  F  N  L  I  S  R  Y  A  G  I  S  537
Cpac_S  520   A  K  L  M  V  P  I  T  S  L  N  L  I  S  R  Y  A  G  I  S  539
Cfau_S  520   A  K  L  M  V  P  I  T  S  L  N  L  I  S  R  Y  A  G  I  S  539
Cnau_S  520   A  K  L  M  V  P  I  T  S  L  N  L  I  S  R  Y  A  G  I  S  539
Pste_S  520   A  K  L  M  V  P  I  T  S  L  N  L  I  S  R  Y  A  G  I  S  539
Rma     516   A  K  L  M  V  P  I  T  S  L  N  L  I  S  R  Y  A  G  I  S  535
Ifos_S  516   A  K  L  M  V  P  I  T  S  L  N  L  I  S  R  Y  A  G  I  S  535
Apha_S  520   A  K  L  M  V  P  I  T  S  L  N  L  I  S  R  Y  A  G  I  S  539
Bsep_S  517   S  K  L  M  V  P  M  T  S  L  N  L  I  S  R  Y  S  G  A  S  536
```

CarD-like/TRCF domain (475-560)

```
Akaw_S 1609 CTAGAAAGTACGCCATTACACAAACTAGGAACCAACCAGTGGGCTAAGGCTAAGAAAAAA 1668
Clau_S 1612 CTAGAAAATACGCCATTACACAAACTAGGAACCAACCAATGGGCTAAGGCTAAGAAAAAA 1671
Pkil_S 1612 CTAAAAAGTACGCCATTACACAAACTAGGAACCAACCAATGGGCTAAGGCTAAGAAAAAA 1671
Psoy_S 1612 CTAAAAAGTACGCCATTACACAAACTAGGAACCAACCAATGGGCTAAGGCTAAGAAAAAA 1671
Vok    1612 CTAAAAAGTACGCCATTACACAAACTAGGAACCAACCAATGGGCCAAGGCTAAGAAAAAA 1671
Cpac_S 1618 CCAGATAGTGCGCCATTACACAAACTAGGTACAAATCAATGGAGCGAGGCTAAGAAAAAA 1677
Cfau_S 1618 CCAGATAGTGCACCATTACACAAGCTAGGTACAAATCAATGGAGCAAGGCTAAGAAAAAA 1677
Cnau_S 1618 TCAGATAGTGCACCATTACACAAGCTAGGTACAAATCAATGGAGCAAGGCTAAGAAAAAA 1677
Pste_S 1618 CCAGATAGTGCACCATTACACAAGCTAGGTACAAACCAATGGAGCAAGGCTAAGAAAAAA 1677
Rma    1606 TCGGAGAATGCTCCATTACACAAGCTAGGAACAAATCAATGGAGCAAGGCTAAGAAAAAA 1665
Ifos_S 1606 ---GATAGTGCACCATTACACAAACTAGGCACAAATCAATGGAGCAAGGCTAAGAAAAAA 1662
Apha_S 1618 CTAGATAGTGCACCATTACACAAGCTAGGCACAAATCAATGGAGTAAGGCTAAGAAAAAA 1677
Bsep_S 1609 CCTGATATTGCACCTCTACACAAATTAGGCTCTAATCAATGGAGTAAGGCAAAGCAAAAA 1668
Akaw_S  537   L  E  S  T  P  L  H  K  L  G  T  N  Q  W  A  K  A  K  K  K  556
Clau_S  538   L  E  N  T  P  L  H  K  L  G  T  N  Q  W  A  K  A  K  K  K  557
Pkil_S  538   L  K  S  T  P  L  H  K  L  G  T  N  Q  W  A  K  A  K  K  K  557
Psoy_S  538   L  K  S  T  P  L  H  K  L  G  T  N  Q  W  A  K  A  K  K  K  557
Vok     538   L  K  S  T  P  L  H  K  L  G  T  N  Q  W  A  K  A  K  K  K  557
Cpac_S  540   P  D  S  A  P  L  H  K  L  G  T  N  Q  W  S  E  A  K  K  K  559
Cfau_S  540   P  D  S  A  P  L  H  K  L  G  T  N  Q  W  S  K  A  K  K  K  559
Cnau_S  540   S  D  S  A  P  L  H  K  L  G  T  N  Q  W  S  K  A  K  K  K  559
Pste_S  540   P  D  S  A  P  L  H  K  L  G  T  N  Q  W  S  K  A  K  K  K  559
Rma     536   S  E  N  A  P  L  H  K  L  G  T  N  Q  W  S  K  A  K  K  K  555
Ifos_S  536   #  D  S  A  P  L  H  K  L  G  T  N  Q  W  S  K  A  K  K  K  554
Apha_S  540   L  D  S  A  P  L  H  K  L  G  T  N  Q  W  S  K  A  K  K  K  559
Bsep_S  537   P  D  I  A  P  L  H  K  L  G  S  N  Q  W  S  K  A  K  Q  K  556
```

CarD-like/TRCF domain (475-560)

```
Akaw_S 1669 GTAAGTGAAGCTTTATTTGATGTAGCAGTTGAATTATTAAAAATTTCTGCCAAACGATCT 1728
Clau_S 1672 GCAAGTGAATCTTTATTTGATGTAGCAGTTGAATTATTAAAAATTTCTGCCAAACGATCC 1731
Pkil_S 1672 GCAGGTGAAGCTTTATTTGATGTAGCAGTTGAATTATTAAAAATTTCTGCCAAGCGATCC 1731
Psoy_S 1672 GCAGGTGAAGCTTTATTTGATGTAGCAGTTGAATTATTAAAAATTTCTGCCAAGCGATCC 1731
Vok    1672 GCAGGTGAAGCTTTATTTGATATAGCAGTTGAATTATTAAAAATTTCTGCCAAGCGATCC 1731
Cpac_S 1678 TCAGGCGAGGCTTTGTTTGATGTGGCGGCTGAATTATTAGAAATTTATGCCAAGCGAGCC 1737
Cfau_S 1678 TCAGGCGAGGCTTTGTTTGATGTGGCGGCTGAATTATTAGAAATTTATGCCAAGCGAGCC 1737
Cnau_S 1678 TCAGGCGAGGCTTTGTTTGATGTGGCGGCTGAATTATTAGAAATTTATGCCAAACGAGCC 1737
Pste_S 1678 TCAAGCGAGGCTTTGTTTGATGTGGCGGCTGAATTATTAGAAATTTATGCCAAGCGAGCC 1737
Rma    1666 GCAGGTGAGGCTTTGTTTGATGTGGCAGCTGAATTATTAGAAATTTATGCCAAACGAGCC 1725
Ifos_S 1663 GCAGGTGAAGCTTTGTTTGACGTGGCAGCTGAATTATTAGAAATTTATGCCAAACGAGCC 1722
Apha_S 1678 GCGGGTGAGGCTTTGTTTGATGTGGCAGCTGAATTATTAGAAATTTATGCCAAACGAGTC 1737
Bsep_S 1669 GCCGTCGACGCCTTGCACGATATTGCCGCAGAATTATTGGAAATCTACGCCAAACGACAG 1728
Akaw_S  557   V  S  E  A  L  F  D  V  A  V  E  L  L  K  I  S  A  K  R  S  576
Clau_S  558   A  S  E  S  L  F  D  V  A  V  E  L  L  K  I  S  A  K  R  S  577
Pkil_S  558   A  G  E  A  L  F  D  V  A  V  E  L  L  K  I  S  A  K  R  S  577
Psoy_S  558   A  G  E  A  L  F  D  V  A  V  E  L  L  K  I  S  A  K  R  S  577
Vok     558   A  G  E  A  L  F  D  I  A  V  E  L  L  K  I  S  A  K  R  S  577
Cpac_S  560   S  G  E  A  L  F  D  V  A  A  E  L  L  E  I  Y  A  K  R  A  579
Cfau_S  560   S  G  E  A  L  F  D  V  A  A  E  L  L  E  I  Y  A  K  R  A  579
Cnau_S  560   S  G  E  A  L  F  D  V  A  A  E  L  L  E  I  Y  A  K  R  A  579
Pste_S  560   S  S  E  A  L  F  D  V  A  A  E  L  L  E  I  Y  A  K  R  A  579
Rma     556   A  G  E  A  L  F  D  V  A  A  E  L  L  E  I  Y  A  K  R  A  575
Ifos_S  555   A  G  E  A  L  F  D  V  A  A  E  L  L  E  I  Y  A  K  R  A  574
Apha_S  560   A  G  E  A  L  F  D  V  A  A  E  L  L  E  I  Y  A  K  R  V  579
Bsep_S  557   A  V  D  A  L  H  D  I  A  A  E  L  L  E  I  Y  A  K  R  Q  576
```

CarD-like/TRCF domain (475-560)

```
Akaw_S 1729 TCCCAAACAGGCTTTTCCTTTCCTAAACCTAATGATGATTACTCTTCATTTGTTGCTAAT 1788
Clau_S 1732 TCCCAAACAGGATTTTCTTTTCCTAAACCTAATGATGATTACTCTTCATTTGTTGCCAAA 1791
Pkil_S 1732 TCACAAACAGGCTTTTCTTTCCCTAAGCCTAATGATGATTACTCTTCATTTGTTGCTAAT 1791
Psoy_S 1732 TCACAAACAGGCTTTTCTTTCCCTAAGCCTAATGATGATTACTCTTCATTTGTTGCTAAT 1791
Vok    1732 TCTCAAACAGGCTTTTCTTTCCCTAAGCCTAATGATGATTACTCTTCATTTGTTGCCAAT 1791
Cpac_S 1738 TCTCAAACAGGCTTTTCTTTTCCTGAGCCGAATGATGCTTATTCCTCATTCGTTGCCAAT 1797
Cfau_S 1738 TCTCAAACAGGCTTTGCTTTTCCTGAACCGAATGATGCTTATTCCTCATTCGTTGCCAGT 1797
Cnau_S 1738 TCTCAAACAGGCTTTGCTTTTCCTGAACCGAATGATGCTTATTCCTCATTCGTTGCCAGT 1797
Pste_S 1738 TCTCAAACAGGTTTTGCTTTTCCTGAGCCGAATGATGCTTATTCCTCATTCGTTACCAGT 1797
Rma    1726 TCTAAAATAAGCTTTGCTTTCCCTAAGCCGAATGATGCTTACTCCTCATTTGTTGCTAGT 1785
Ifos_S 1723 TCTCAAACAGGTTTTGCTTTCCCTAAGCCGAATGATGCTTATTCCTCATTTGTTGCCAGC 1782
Apha_S 1738 TCTCAAACAGGCTTTGCTTTCCCTGAACCGAATGATGCTTATTCCTCATTTGTTGCCAGC 1797
Bsep_S 1729 GCACAAGTTGGCTTTGCGTTTCCAGAGCCGAATGATGCGTATTCGTCGTTTGTTGCCAGT 1788
Akaw_S  577   S  Q  T  G  F  S  F  P  K  P  N  D  D  Y  S  S  F  V  A  N  596
Clau_S  578   S  Q  T  G  F  S  F  P  K  P  N  D  D  Y  S  S  F  V  A  K  597
Pkil_S  578   S  Q  T  G  F  S  F  P  K  P  N  D  D  Y  S  S  F  V  A  N  597
Psoy_S  578   S  Q  T  G  F  S  F  P  K  P  N  D  D  Y  S  S  F  V  A  N  597
Vok     578   S  Q  T  G  F  S  F  P  K  P  N  D  D  Y  S  S  F  V  A  N  597
Cpac_S  580   S  Q  T  G  F  S  F  P  E  P  N  D  A  Y  S  S  F  V  A  N  599
Cfau_S  580   S  Q  T  G  F  A  F  P  E  P  N  D  A  Y  S  S  F  V  A  S  599
Cnau_S  580   S  Q  T  G  F  A  F  P  E  P  N  D  A  Y  S  S  F  V  A  S  599
Pste_S  580   S  Q  T  G  F  A  F  P  E  P  N  D  A  Y  S  S  F  V  T  S  599
Rma     576   S  K  I  S  F  A  F  P  K  P  N  D  A  Y  S  S  F  V  A  S  595
Ifos_S  575   S  Q  T  G  F  A  F  P  K  P  N  D  A  Y  S  S  F  V  A  S  594
Apha_S  580   S  Q  T  G  F  A  F  P  E  P  N  D  A  Y  S  S  F  V  A  S  599
Bsep_S  577   A  Q  V  G  F  A  F  P  E  P  N  D  A  Y  S  S  F  V  A  S  596
```

```
Akaw_S 1789 TTTCCCTTTGAAGAAACTCCAGATCAGATAAAAACCATGAATGAAGTACTAGCAGATATG 1848
Clau_S 1792 TTTCCATTTGAGGAAACTCCAGATCAAATAAAAACCATGAATGAAGTACTAGCAGATATG 1851
Pkil_S 1792 TTCCCCTTTGAGGAAACTCCAGATCAGATAAAAACCATGAATGAGGTACTAGCAGATATG 1851
Psoy_S 1792 TTCCCCTTTGAGGAAACTCCAGATCAGATAAAAACCATGAATGAGGTACTAGCAGATATG 1851
Vok    1792 TTTCCCTTTGAGGAAACTCCAGATCAGATAAAAACCATGAATGAAGTACTAGCAGATATG 1851
Cpac_S 1798 TTTCCCTTTGAGGAAACACCAGACCAGCTCAAAACCATGGGCGAAGTGTTAGCAGATATG 1857
Cfau_S 1798 TTTCCCTTTGAGGAAACACCGGACCAGCTCAAAACCATGGGTGAAGTGTTAGCAGATATG 1857
Cnau_S 1798 TTTCCCTTTGAGGAAACACCAGACCAGCTCAAAACCATGGACGAAGTGTTAGCAGATATG 1857
Pste_S 1798 TTTCCCTTTGAGGAAACACCAGACCAGCTCAAAACCATGGGCGAAGTGTTAGCAGATATG 1857
Rma    1786 TTCCCTTTTGAGGAAACACCAGACCAACTAAAAACCATGGATGAAGTTTTAGCAGATATG 1845
Ifos_S 1783 TTTCCCTTTGAGGAAACACCAGACCAACTAAAAACCATGGGCGAAGTCTTAGCAGATATG 1842
Apha_S 1798 TTTCCCTTTGAAGAAACACCAGATCAGCTAAAAACTATGGGCGAAGTTTTAGCAGATATG 1857
Bsep_S 1789 TTCCCTTTTGAAGAAACGCCCGACCAACTGAAAACCATGGGCGAGGTGTTGGCAGATATG 1848
Akaw_S  597   F  P  F  E  E  T  P  D  Q  I  K  T  M  N  E  V  L  A  D  M  616
Clau_S  598   F  P  F  E  E  T  P  D  Q  I  K  T  M  N  E  V  L  A  D  M  617
Pkil_S  598   F  P  F  E  E  T  P  D  Q  I  K  T  M  N  E  V  L  A  D  M  617
Psoy_S  598   F  P  F  E  E  T  P  D  Q  I  K  T  M  N  E  V  L  A  D  M  617
Vok     598   F  P  F  E  E  T  P  D  Q  I  K  T  M  N  E  V  L  A  D  M  617
Cpac_S  600   F  P  F  E  E  T  P  D  Q  L  K  T  M  G  E  V  L  A  D  M  619
Cfau_S  600   F  P  F  E  E  T  P  D  Q  L  K  T  M  G  E  V  L  A  D  M  619
Cnau_S  600   F  P  F  E  E  T  P  D  Q  L  K  T  M  D  E  V  L  A  D  M  619
Pste_S  600   F  P  F  E  E  T  P  D  Q  L  K  T  M  G  E  V  L  A  D  M  619
Rma     596   F  P  F  E  E  T  P  D  Q  L  K  T  M  D  E  V  L  A  D  M  615
Ifos_S  595   F  P  F  E  E  T  P  D  Q  L  K  T  M  G  E  V  L  A  D  M  614
Apha_S  600   F  P  F  E  E  T  P  D  Q  L  K  T  M  G  E  V  L  A  D  M  619
Bsep_S  597   F  P  F  E  E  T  P  D  Q  L  K  T  M  G  E  V  L  A  D  M  616
```

```
Akaw_S 1849 CAATCACAACAACCAATGGACAGATTGATATGTGGAGACGTTGGTTTTGGAAAAACTGAG 1908
Clau_S 1852 CAATCACAACAACCAATGGACAGATTAGTATGTGGAGACGTTGGTTTTGGAAAAACTGAA 1911
Pkil_S 1852 CAATCACAACAACCAATGGACAGATTGGTATGTGGAGATGTTGGTTTTGGAAAAACTGAG 1911
Psoy_S 1852 CAATCACAACAACCAATGGACAGATTGGTATGTGGAGATGTTGGTTTTGGAAAAACTGAG 1911
Vok    1852 CAATCACAACAACCAATGGACAGATTGGTATGTGGAGACGTTGGTTTTGGAAAAACTGAG 1911
Cpac_S 1858 CAATCACACAAACCAATGGACAGATTGGTTTGTGGCGATGTTGGTTTTGGAAAAACTGAA 1917
Cfau_S 1858 CAATCACACAAACCAATGGACAGATTGGTTTGTGGCGATGTTGGTTTTGGTAAAACTGAA 1917
Cnau_S 1858 CAATCACACAAACCAATGGACAGATTGATTTGTGGCGATGTTGGTTTTGGCAAAACTGAA 1917
Pste_S 1858 CAATCACACAAACCAATGGACAGATTGGTTTGTGGAGATGTTGGTTTTGGTAAAACTGAA 1917
Rma    1846 CAATCATGCAGGCCAATGGACAGATTGGTCTGTGGCGATGTTGGTTTTGGTAAAACTGAA 1905
Ifos_S 1843 CAATCACACAGACCAATGGACAGATTAGTCTGTGGCGATGTTGGTTTTGGCAAAACTGAA 1902
Apha_S 1858 CAATCACACAAACCAATGGACAGATTGGTCTGTGGCGATGTTGGTTTTGGCAAAACTGAA 1917
Bsep_S 1849 CAATCGCAAAAACCAATGGACAGACTGGTCTGTGGTGATGTGGGCTTCGGTAAAACTGAA 1908
Akaw_S  617   Q  S  Q  Q  P  M  D  R  L  I  C  G  D  V  G  F  G  K  T  E  636
Clau_S  618   Q  S  Q  Q  P  M  D  R  L  V  C  G  D  V  G  F  G  K  T  E  637
Pkil_S  618   Q  S  Q  Q  P  M  D  R  L  V  C  G  D  V  G  F  G  K  T  E  637
Psoy_S  618   Q  S  Q  Q  P  M  D  R  L  V  C  G  D  V  G  F  G  K  T  E  637
Vok     618   Q  S  Q  Q  P  M  D  R  L  V  C  G  D  V  G  F  G  K  T  E  637
Cpac_S  620   Q  S  H  K  P  M  D  R  L  V  C  G  D  V  G  F  G  K  T  E  639
Cfau_S  620   Q  S  H  K  P  M  D  R  L  V  C  G  D  V  G  F  G  K  T  E  639
Cnau_S  620   Q  S  H  K  P  M  D  R  L  I  C  G  D  V  G  F  G  K  T  E  639
Pste_S  620   Q  S  H  K  P  M  D  R  L  V  C  G  D  V  G  F  G  K  T  E  639
Rma     616   Q  S  C  R  P  M  D  R  L  V  C  G  D  V  G  F  G  K  T  E  635
Ifos_S  615   Q  S  H  R  P  M  D  R  L  V  C  G  D  V  G  F  G  K  T  E  634
Apha_S  620   Q  S  H  K  P  M  D  R  L  V  C  G  D  V  G  F  G  K  T  E  639
Bsep_S  617   Q  S  Q  K  P  M  D  R  L  V  C  G  D  V  G  F  G  K  T  E  636

E. coli                          L  V  C  G  D  V  G  F  G  K  T
```

Motif I

```
Akaw_S 1909 ATTGCCATGCGTGCAGCTTTTTTAGCAGTTAAAGCAGGTAAACAAGTTGCAATTTTAGTA 1968
Clau_S 1912 ATTGCCATGCGTGCAGCTTTTTTAGCAGTTAAAGCAGGTAAACAAGTTGCAATTTTAGTG 1971
Pkil_S 1912 ATTGCCATGCGTGCAGCTTTTTTAGCAGTTAAAGCAGGTAAACAAGTTGCAATTTTAGTA 1971
Psoy_S 1912 ATTGCCATGCGTGCAGCTTTTTTAGCAGTTAAAGCAGGTAAACAAGTTGCAATTTTAGTA 1971
Vok    1912 ATTGCCATGCGTGCAGCTTTTTTAGCAGTTAAAGCAGGTAAACAAGTTGCAATTTTAGTA 1971
Cpac_S 1918 ATTGCCATGCGTGCAGCGTTTTTAGCAGTTGAGGCAGGCAAGCAAGTGGCAATTTTGGTA 1977
Cfau_S 1918 ATTGCCATGCGTGCAGCGTTTTTAGCAGTTGAGGCAGGCAAGCAAGTGGCAATTCTGGTA 1977
Cnau_S 1918 ATTGCCATGCGTGCAGCGTTTTTAGCAGTTGAGGCAGGCAAGCAAGTGGCAATTTTGGTA 1977
Pste_S 1918 ATTGCCATGCGTGCAGCGTTTTTAGCAGTTGAATCAGGCAAGCAAGTGGTAATTTTGGTA 1977
Rma    1906 ATTGCTATGCGTGCGGCATTCTTAGCGGTTGAAGCGGGGAAGCAAGTGGCAATTTTGGTT 1965
Ifos_S 1903 ATTGCCATGCGTGCGGCATTTTTAGCAGTTGAAGCAGGAAAGCAAGTGGCAATTTTGGTA 1962
Apha_S 1918 ATTGCCATGCGTGCGGCATTCTTAGCGGTTGAAGCAGGCAAACAAGTGGCAATTTTGGTA 1977
Bsep_S 1909 ATTGCCATGCGAGCAGCATTTTTAGCCGTTGAAGCGGGTAAACAGGTGGCAATTTTGGTG 1968
Akaw_S  637  I  A  M  R  A  A  F  L  A  V  K  A  G  K  Q  V  A  I  L  V  656
Clau_S  638  I  A  M  R  A  A  F  L  A  V  K  A  G  K  Q  V  A  I  L  V  657
Pkil_S  638  I  A  M  R  A  A  F  L  A  V  K  A  G  K  Q  V  A  I  L  V  657
Psoy_S  638  I  A  M  R  A  A  F  L  A  V  K  A  G  K  Q  V  A  I  L  V  657
Vok     638  I  A  M  R  A  A  F  L  A  V  K  A  G  K  Q  V  A  I  L  V  657
Cpac_S  640  I  A  M  R  A  A  F  L  A  V  E  A  G  K  Q  V  A  I  L  V  659
Cfau_S  640  I  A  M  R  A  A  F  L  A  V  E  A  G  K  Q  V  A  I  L  V  659
Cnau_S  640  I  A  M  R  A  A  F  L  A  V  E  A  G  K  Q  V  A  I  L  V  659
Pste_S  640  I  A  M  R  A  A  F  L  A  V  E  S  G  K  Q  V  V  I  L  V  659
Rma     636  I  A  M  R  A  A  F  L  A  V  E  A  G  K  Q  V  A  I  L  V  655
Ifos_S  635  I  A  M  R  A  A  F  L  A  V  E  A  G  K  Q  V  A  I  L  V  654
Apha_S  640  I  A  M  R  A  A  F  L  A  V  E  A  G  K  Q  V  A  I  L  V  659
Bsep_S  637  I  A  M  R  A  A  F  L  A  V  E  A  G  K  Q  V  A  I  L  V  656

E. coli                                                     V  A  V  L  V
                                                               Motif IA
```

```
Akaw_S 1969 CCAACCACACTATTATCTAACCAACACTATCGATCTTTTATCGATCGTTTCACTGATGAT 2028
Clau_S 1972 CCAACTACACTATTATCCAACCAACACTATCGATCTTTTATCGATCGTTTCAATAATGAT 2031
Pkil_S 1972 CCAACCACACTATTATCCAACCAACACTATCGATCTTTTATCGATCGTTTCACTAATGAT 2031
Psoy_S 1972 CCAACCACACTATTATCCAACCAACACTATCGATCTTTTATCGATCGTTTCACTAATGAT 2031
Vok    1972 CCAACCACACTATTATCCAACCAACACTATCGATCTTTTATCGATCGTTTCACTAATGAT 2031
Cpac_S 1978 CCAACCACGTTATTATCTAACCAACATTATCAATCGTTTGTTGACCGTTTTGCTAGCCAC 2037
Cfau_S 1978 CCAACCACGCTATTATCTAACCAACATTACCAATCATTTGTTGACCGTTTTACTAGCTAC 2037
Cnau_S 1978 CCAACCACGCTATTATCTAACCAACATTACCAATCGTTTGTTAACCGTTTTGCTAACCAC 2037
Pste_S 1978 CCAACCACGCTATTATCTAACCAACATTATCAATCATTTGTTGACCGTTTTGCTAGCCAC 2037
Rma    1966 CCAACCACGCTATTATCTAACCAGCATTATCAATCATTTGTTGACCGCTTTATTAACCAC 2025
Ifos_S 1963 CCAACCACGCTATTATCTAACCAGCACCATCAATCGTTTATTGACCGTTTTGCCAACCAC 2022
Apha_S 1978 CCAACCACGCTATTATCTAACCAGCACTATCAATCGTTTGTTGACCGTTTTGCCAACCAC 2037
Bsep_S 1969 CCGACGACCTTGTTGGCAAATCAGCATTTTGAGTCCTTTAAAGATAGGTTTGTTAAATAT 2028
Akaw_S  657  P  T  T  L  L  S  N  Q  H  Y  R  S  F  I  D  R  F  T  D  D  676
Clau_S  658  P  T  T  L  L  S  N  Q  H  Y  R  S  F  I  D  R  F  N  N  D  677
Pkil_S  658  P  T  T  L  L  S  N  Q  H  Y  R  S  F  I  D  R  F  T  N  D  677
Psoy_S  658  P  T  T  L  L  S  N  Q  H  Y  R  S  F  I  D  R  F  T  N  D  677
Vok     658  P  T  T  L  L  S  N  Q  H  Y  R  S  F  I  D  R  F  T  N  D  677
Cpac_S  660  P  T  T  L  L  S  N  Q  H  Y  Q  S  F  V  D  R  F  A  S  H  679
Cfau_S  660  P  T  T  L  L  S  N  Q  H  Y  Q  S  F  V  D  R  F  T  S  Y  679
Cnau_S  660  P  T  T  L  L  S  N  Q  H  Y  Q  S  F  V  N  R  F  A  N  H  679
Pste_S  660  P  T  T  L  L  S  N  Q  H  Y  Q  S  F  V  D  R  F  A  S  H  679
Rma     656  P  T  T  L  L  S  N  Q  H  Y  Q  S  F  V  D  R  F  I  N  H  675
Ifos_S  655  P  T  T  L  L  S  N  Q  H  H  Q  S  F  I  D  R  F  A  N  H  674
Apha_S  660  P  T  T  L  L  S  N  Q  H  Y  Q  S  F  V  D  R  F  A  N  H  679
Bsep_S  657  P  T  T  L  L  A  N  Q  H  F  E  S  F  K  D  R  F  V  K  Y  676

E. coli      P  T  T
```

```
Akaw_S 2029 CCTACAAAAATTGCAACATTATCAAGATTTCAAACCCCAAAAGAGCAAAAAATTATAATT 2088
Clau_S 2032 CCTACAAAAATTGCAACATTATCAAGATTTCAAACCCCAAAAGAGCAAAAAATAATTATT 2091
Pkil_S 2032 CCTACAAAAATTGCAACATTATCAAGATTCCAAACCCCAAAAGAGCAAAAAATAATTATT 2091
Psoy_S 2032 CCTACAAAAATTGCAACATTATCAAGATTCCAAACCCCAAAAGAGCAAAAAATAATTATT 2091
Vok    2032 CCTACAAAAATTGCAACATTATCAAGATTCCAAACCCCAAAAGAGCAAAAAATAATTATT 2091
Cpac_S 2038 CCTGTTGAAATTGCAGCGCTTTCAAGGCTTCAAACCCCAAAAGAGCAAAGAATAATCATT 2097
Cfau_S 2038 CCTGTTGAAATTGCAGCGCTTTCAAGGTTTCAAACTCCAAAAGAGCAAAGAATAATCATT 2097
Cnau_S 2038 CCTGTTGAAATTGCAGCGCTCTCAAGGTTTCAAACCCCAAAAGAGCAAGAAATAATCATT 2097
Pste_S 2038 CCTGTTGAAATTGCAGCGCTTTCAAGGTTTCAAACCCCAAAAGAGCAAAAAATAATCATT 2097
Rma    2026 CCTGTTGAAATTGCAGCGCTTTCAAGGTTTCAAACTCAAAAAGAGAAAAAACTAATTATT 2085
Ifos_S 2023 CCTGTTGAAATTGCAGCGCTTTCAAGGTTTCAAACCCCAAAAGAGCAAAAACTAATTATT 2082
Apha_S 2038 CCTGTTGAAATTGCAGCGCTTTCAAGGTTTCAAACCCAAAAAGAGCAAAAACTAATTATT 2097
Bsep_S 2029 CCAGTGGAAATTGCAGCAATGTCACGCTTTCAAACTACTAAGGAGCAAACACGCATTAAG 2088
Akaw_S  677   P  T  K  I  A  T  L  S  R  F  Q  T  P  K  E  Q  K  I  I  I  696
Clau_S  678   P  T  K  I  A  T  L  S  R  F  Q  T  P  K  E  Q  K  I  I  I  697
Pkil_S  678   P  T  K  I  A  T  L  S  R  F  Q  T  P  K  E  Q  K  I  I  I  697
Psoy_S  678   P  T  K  I  A  T  L  S  R  F  Q  T  P  K  E  Q  K  I  I  I  697
Vok     678   P  T  K  I  A  T  L  S  R  F  Q  T  P  K  E  Q  K  I  I  I  697
Cpac_S  680   P  V  E  I  A  A  L  S  R  L  Q  T  P  K  E  Q  R  I  I  I  699
Cfau_S  680   P  V  E  I  A  A  L  S  R  F  Q  T  P  K  E  Q  R  I  I  I  699
Cnau_S  680   P  V  E  I  A  A  L  S  R  F  Q  T  P  K  E  Q  E  I  I  I  699
Pste_S  680   P  V  E  I  A  A  L  S  R  F  Q  T  P  K  E  Q  K  I  I  I  699
Rma     676   P  V  E  I  A  A  L  S  R  F  Q  T  Q  K  E  K  K  L  I  I  695
Ifos_S  675   P  V  E  I  A  A  L  S  R  F  Q  T  P  K  E  Q  K  L  I  I  694
Apha_S  680   P  V  E  I  A  A  L  S  R  F  Q  T  Q  K  E  Q  K  L  I  I  699
Bsep_S  677   P  V  E  I  A  A  M  S  R  F  Q  T  T  K  E  Q  T  R  I  K  696
```

```
Akaw_S 2089 GAACAACTAAAACAAGGAAACATTGATATCATTATTGGCACACACAAAATTATTCAAAAT 2148
Clau_S 2092 GAACAACTAAAAGAAGGAACCATTAATATTATTATCGGTACACACAAAATTATTCAAAAT 2151
Pkil_S 2092 GAACAACTAAAGCAAGGAACCATTGATATCATTATCGGTACACACAAAATTATTCAGAAT 2151
Psoy_S 2092 GAACAACTAAAGCAAGGAACCATTGATATCATTATCGGTACACACAAAATTATTCAGAAT 2151
Vok    2092 GAACAACTAAAGCAAGGAACCATTGATATCATTATCGGTACACACAAAATTATTCAAAAT 2151
Cpac_S 2098 GAAAAATTAAACCAAGGAAAAGTTGACATCGTCATTGGTACACACAAAATTATTCAAGAC 2157
Cfau_S 2098 GAAAAATTAAACCAAGGAAAAATTGACATCGTCATTGGTACACACAAAATTATTCAAGGC 2157
Cnau_S 2098 GAAAAATTAAACCAAGGGAAAATTGACATCGTCATTGGTACACACAAAATTATTCAAGGC 2157
Pste_S 2098 GAAAAATTAAACCAAGGAAAAATTGACATCGTCATTGGTACACACAAAATTATTCAAGAC 2157
Rma    2086 AAAAAATTAAATCAAGGAACAATTGACATTATCATTGGTACTCACACAATTATTCAAAGC 2145
Ifos_S 2083 GAAAAATTAAACCAAGGAACAATTGACATTGTCATTGGCACGCACAAAATTATTCAAGGC 2142
Apha_S 2098 GAAAAATTAAACCAAGGAACAATTGACATTGTCATTGGCACACACAAAATTATTCAAGGT 2157
Bsep_S 2089 CAACAACTGCTTGAAGGAAAAGTGGACATCGTTATCGGCACGCACAAGCTCATTCACGGC 2148
Akaw_S  697   E  Q  L  K  Q  G  N  I  D  I  I  I  G  T  H  K  I  I  Q  N  716
Clau_S  698   E  Q  L  K  E  G  T  I  N  I  I  I  G  T  H  K  I  I  Q  N  717
Pkil_S  698   E  Q  L  K  Q  G  T  I  D  I  I  I  G  T  H  K  I  I  Q  N  717
Psoy_S  698   E  Q  L  K  Q  G  T  I  D  I  I  I  G  T  H  K  I  I  Q  N  717
Vok     698   E  Q  L  K  Q  G  T  I  D  I  I  I  G  T  H  K  I  I  Q  N  717
Cpac_S  700   E  K  L  N  Q  G  K  V  D  I  V  I  G  T  H  K  I  I  Q  D  719
Cfau_S  700   E  K  L  N  Q  G  K  I  D  I  V  I  G  T  H  K  I  I  Q  G  719
Cnau_S  700   E  K  L  N  Q  G  K  I  D  I  V  I  G  T  H  K  I  I  Q  G  719
Pste_S  700   E  K  L  N  Q  G  K  I  D  I  V  I  G  T  H  K  I  I  Q  D  719
Rma     696   K  K  L  N  Q  G  T  I  D  I  I  I  G  T  H  T  I  I  Q  S  715
Ifos_S  695   E  K  L  N  Q  G  T  I  D  I  V  I  G  T  H  K  I  I  Q  G  714
Apha_S  700   E  K  L  N  Q  G  T  I  D  I  V  I  G  T  H  K  I  I  Q  G  719
Bsep_S  697   Q  Q  L  L  E  G  K  V  D  I  V  I  G  T  H  K  L  I  H  G  716
```

```
Akaw_S 2149 AACATTAAATACAAAAACCTTAGCTTGATTATTATTGATGAAGAACATCGCTTTGGAGTT 2208
Clau_S 2152 AATATTAAATACAAAAACCTCAGCTTGATTATTATTGATGAAGAACACCGCTTTGGGGTT 2211
Pkil_S 2152 AATATTAAATACAAAAGCCTTAGCTTGATTATTATTGATGAAGAACATCGCTTTGGAGTT 2211
Psoy_S 2152 AATATTAAATACAAAAGCCTTAGCTTGATTATTATTGATGAAGAACATCGCTTTGGAGTT 2211
Vok    2152 AATATTAAATATAAAAACCTTAGCTTGATTATTATTGATGAAGAACATCGCTTTGGAGTT 2211
Cpac_S 2158 GCTATTAAATACAAAAACCTTGGCTTGATTATTATTGACGAAGAGCATCGCTTCGGCGTT 2217
Cfau_S 2158 ACTATTAAATACAAAAACCTTGGCTTGCTTATTATTGACGAAGAGCATCGCTTCGGCGTT 2217
Cnau_S 2158 ACTATTAAATACAAAAACCTTGGTTTGATTATTATTGACGAAGAGCATCGCTTCGGCGTT 2217
Pste_S 2158 ACTATTAAATACAAAAACCTTGGCTTGATTATTATTGACGAAGAGCATCGCTTCGGCGTT 2217
Rma    2146 ACTATTAAATACAAAGATCTTGGCTTGATTATTATTGACGAAGAGCATCGTTTCGGTGTT 2205
Ifos_S 2143 ACTATTAAATACAAAAACCTTGGCTTAATTATTATTGACGAAGAACATCGCTTCGGCGTT 2202
Apha_S 2158 ACTATTAAATACAAAAACCTTGGCTTGATTATTATTGACGAAGAGCATCGCTTCGGCGTT 2217
Bsep_S 2149 AGTATTAAGTATAAAAATCTGGGGCTGATTATCATTGATGAAGAGCATAGATTTGGCGTT 2208
Akaw_S  717  N  I  K  Y  K  N  L  S  L  I  I  I  D  E  E  H  R  F  G  V  736
Clau_S  718  N  I  K  Y  K  N  L  S  L  I  I  I  D  E  E  H  R  F  G  V  737
Pkil_S  718  N  I  K  Y  K  S  L  S  L  I  I  I  D  E  E  H  R  F  G  V  737
Psoy_S  718  N  I  K  Y  K  S  L  S  L  I  I  I  D  E  E  H  R  F  G  V  737
Vok     718  N  I  K  Y  K  N  L  S  L  I  I  I  D  E  E  H  R  F  G  V  737
Cpac_S  720  A  I  K  Y  K  N  L  G  L  I  I  I  D  E  E  H  R  F  G  V  739
Cfau_S  720  T  I  K  Y  K  N  L  G  L  L  I  I  D  E  E  H  R  F  G  V  739
Cnau_S  720  T  I  K  Y  K  N  L  G  L  I  I  I  D  E  E  H  R  F  G  V  739
Pste_S  720  T  I  K  Y  K  N  L  G  L  I  I  I  D  E  E  H  R  F  G  V  739
Rma     716  T  I  K  Y  K  D  L  G  L  I  I  I  D  E  E  H  R  F  G  V  735
Ifos_S  715  T  I  K  Y  K  N  L  G  L  I  I  I  D  E  E  H  R  F  G  V  734
Apha_S  720  T  I  K  Y  K  N  L  G  L  I  I  I  D  E  E  H  R  F  G  V  739
Bsep_S  717  S  I  K  Y  K  N  L  G  L  I  I  I  D  E  E  H  R  F  G  V  736

E. coli                                L  I  V  D  E  E  H
```

Motif II

```
Akaw_S 2209 AAACAAAAAGAAGCCTTGAAAAAACTAAGAGGGCAAAGTGATATTCTAACCATGACTGCC 2268
Clau_S 2212 AGACAAAAAGAAGCCTTGAAAAAACTACGAGGGCAAAGTGATATTTTAACTATGACTGCC 2271
Pkil_S 2212 AAACAAAAAGAAGCCTTAAAAAAACTACGAGGACAAAGTGATATTCTAACCATGACTGCT 2271
Psoy_S 2212 AAACAAAAAGAAGCCTTAAAAAAACTACGAGGACAAAGTGATATTCTAACCATGACTGCT 2271
Vok    2212 AAACAAAAAGAAGCTTTAAAAAAACTACGAGGACAAAGTGATATTCTAACCATGACTGCC 2271
Cpac_S 2218 AAACAAAAAGAGGCGTTGAAAAAACTACGGGGCGAGGGTGACATTTTGACCATGACTGCC 2277
Cfau_S 2218 AAACAAAAAGAGGCGTTGAAAAAACTACGGGGCGAGAGTGACGTTCTGACCATGACTGCC 2277
Cnau_S 2218 AAACAAAAAGAGGCGTTGAAAAAACTACGGGGCGAGAGTGACATTCTGACCATGACTGCC 2277
Pste_S 2218 AAACAAAAAGAGGCGTTGAAAAAGCTACGGGGCGAGAGTGACATTCTGACCATGACTGCC 2277
Rma    2206 AAACAAAAAGAGGCATTGAAAAAAATACGGGGAGAAAGTGACATTCTGACCATGACTGCC 2265
Ifos_S 2203 AAACAAAAAGAGGCGTTGAAAAAACTACGGGGTGAAAGTGACATTCTGACCATGACTGCC 2262
Apha_S 2218 AAACAAAAAGAGGCATTGAAAAAACTACGGGGCGAGAGCGACATTCTAACCATGACTGCC 2277
Bsep_S 2209 AAACAAAAGGAGTCTCTGAAAAAAATTCGGGGTCAGAGTGATATTCTAACAATGACAGCA 2268
Akaw_S  737  K  Q  K  E  A  L  K  K  L  R  G  Q  S  D  I  L  T  M  T  A  756
Clau_S  738  R  Q  K  E  A  L  K  K  L  R  G  Q  S  D  I  L  T  M  T  A  757
Pkil_S  738  K  Q  K  E  A  L  K  K  L  R  G  Q  S  D  I  L  T  M  T  A  757
Psoy_S  738  K  Q  K  E  A  L  K  K  L  R  G  Q  S  D  I  L  T  M  T  A  757
Vok     738  K  Q  K  E  A  L  K  K  L  R  G  Q  S  D  I  L  T  M  T  A  757
Cpac_S  740  K  Q  K  E  A  L  K  K  L  R  G  E  G  D  I  L  T  M  T  A  759
Cfau_S  740  K  Q  K  E  A  L  K  K  L  R  G  E  S  D  V  L  T  M  T  A  759
Cnau_S  740  K  Q  K  E  A  L  K  K  L  R  G  E  S  D  I  L  T  M  T  A  759
Pste_S  740  K  Q  K  E  A  L  K  K  L  R  G  E  S  D  I  L  T  M  T  A  759
Rma     736  K  Q  K  E  A  L  K  K  I  R  G  E  S  D  I  L  T  M  T  A  755
Ifos_S  735  K  Q  K  E  A  L  K  K  L  R  G  E  S  D  I  L  T  M  T  A  754
Apha_S  740  K  Q  K  E  A  L  K  K  L  R  G  E  S  D  I  L  T  M  T  A  759
Bsep_S  737  K  Q  K  E  S  L  K  K  I  R  G  Q  S  D  I  L  T  M  T  A  756

E. coli                                         I  L  T  L  T  A
```

Motif III

```
Akaw_S 2269 ACTCCTATTCCACGTACATTAAATATGGCGCTAGGTTCATTAAAAGAATTATCTATTATT 2328
Clau_S 2272 ACCCCTATTCCACGTACATTAAATATGGCACTAGGTTCATTAAGAGAATTATCTATTATT 2331
Pkil_S 2272 ACTCCTATTCCACGTACATTAAATATGGCACTAAGTTCATTAAGAGAATTATCTATTATT 2331
Psoy_S 2272 ACTCCTATTCCACGTACATTAAATATGGCACTAAGTTCATTAAGAGAATTATCTATTATT 2331
Vok    2272 ACCCCTATTCCACGTACATTAAATATGGCACTAGGTTCATTAAGAGAATTATCTATTATT 2331
Cpac_S 2278 ACCCCTATTCCACGCACATTGAATATGGCGCTAGGCTCATTAAGAGAATTGTCTATTATT 2337
Cfau_S 2278 ACCCCTATTCCACGTACATTGAATATGGCGCTAGGCTCATTAAGAGAATTGTCTATTATT 2337
Cnau_S 2278 ACCCCTGTTCCACGCACATTGAATATGGCGCTAGGCTCATTAAGAGAATTGTCTATTATT 2337
Pste_S 2278 ACCCCTATTCCACGCACATTGAATATGGCGCTAGGCTCATTAAGAGAATTGTCTATTATT 2337
Rma    2266 ACCCCTATTCCACGCACATTGAATATGGCGCTAGGTTCATTAAGAGAGCTGTCCATTATT 2325
Ifos_S 2263 ACCCCTATTCCGCGCACATTAAATATGGCGCTAGGCTCATTAAGAGAATTGTCCATTATT 2322
Apha_S 2278 ACCCCTATTCCACGCACATTAAGTATGGCGCTAGGCTCATTAAGAGAGTTATCCATTATT 2337
Bsep_S 2269 ACCCCGATTCCACGCACGCTGAATATGGCACTCGGCTCGCTCAGAGAGTTGTCCATTATT 2328
Akaw_S  757  T  P  I  P  R  T  L  N  M  A  L  G  S  L  K  E  L  S  I  I  776
Clau_S  758  T  P  I  P  R  T  L  N  M  A  L  G  S  L  R  E  L  S  I  I  777
Pkil_S  758  T  P  I  P  R  T  L  N  M  A  L  S  S  L  R  E  L  S  I  I  777
Psoy_S  758  T  P  I  P  R  T  L  N  M  A  L  S  S  L  R  E  L  S  I  I  777
Vok     758  T  P  I  P  R  T  L  N  M  A  L  G  S  L  R  E  L  S  I  I  777
Cpac_S  760  T  P  I  P  R  T  L  N  M  A  L  G  S  L  R  E  L  S  I  I  779
Cfau_S  760  T  P  I  P  R  T  L  N  M  A  L  G  S  L  R  E  L  S  I  I  779
Cnau_S  760  T  P  V  P  R  T  L  N  M  A  L  G  S  L  R  E  L  S  I  I  779
Pste_S  760  T  P  I  P  R  T  L  N  M  A  L  G  S  L  R  E  L  S  I  I  779
Rma     756  T  P  I  P  R  T  L  N  M  A  L  G  S  L  R  E  L  S  I  I  775
Ifos_S  755  T  P  I  P  R  T  L  N  M  A  L  G  S  L  R  E  L  S  I  I  774
Apha_S  760  T  P  I  P  R  T  L  S  M  A  L  G  S  L  R  E  L  S  I  I  779
Bsep_S  757  T  P  I  P  R  T  L  N  M  A  L  G  S  L  R  E  L  S  I  I  776

E. coli      T  P  I  P
```

```
Akaw_S 2329 GCAACGCCACCTGCTAAGCGCAGCACTATCCAAACTTTTGTACAAGAATGGCATAACAAT 2388
Clau_S 2332 GCAACGCCACCTGCTAAGCGCAGTACTATCCAAACTTTTGTACAAGAATGGCATAACAAT 2391
Pkil_S 2332 GCAACGCCACCCGCTAAGCGCAGTACTATCCAAACTTTTGTACAAGAATGGCATAACAAT 2391
Psoy_S 2332 GCAACGCCACCCGCTAAGCGCAGTACTATCCAAACTTTTGTACAAGAATGGCATAACAAT 2391
Vok    2332 GCAACGCCACCAGCTAAGCGCAGTACTATCCAAACTTTTGTACAAGAATGGCATAACGAT 2391
Cpac_S 2338 GCCACGCCACCTGCCAAACGTAACGCCATTCAAACATTTGTACAAGAGTGGAGTAACAAT 2397
Cfau_S 2338 GCCACGCCACCTGCCAAACGTAGCGCCATCCAAACGTTTGTACAAGAGTGGAGTAACAAT 2397
Cnau_S 2338 GCCACGCCACCTGCCAAACGTAGCGCCATTCAAACGTTTGTACAAGAGTGGAGTAACAAC 2397
Pste_S 2338 GCCACGCCACCTGCCAAACGTAGCGCCATTCAAACGTTTGTACAAGAGTGGAATAACAAC 2397
Rma    2326 GCCACCCCCCCTGCCAAACGTAGTGCCATTCAAACGTTTGTACAAGAGTGGAATAACAAC 2385
Ifos_S 2323 GCCACGCCACCTGCCAAACGCAGCGCCATCCAAACGTTTGTACAAGAGTGGAATGACAAC 2382
Apha_S 2338 GCCACGCCACCTGCCAAACGTAGTGCCATCCAAACGTTTGTGCAAGAGTGGGATAACAAC 2397
Bsep_S 2329 GCCACGCCACCGCAAGGGCGGAGCGCAATTCAGACTTTTGTCAACGAGTGGAGTGATGCC 2388
Akaw_S  777  A  T  P  P  A  K  R  S  T  I  Q  T  F  V  Q  E  W  H  N  N  796
Clau_S  778  A  T  P  P  A  K  R  S  T  I  Q  T  F  V  Q  E  W  H  N  N  797
Pkil_S  778  A  T  P  P  A  K  R  S  T  I  Q  T  F  V  Q  E  W  H  N  N  797
Psoy_S  778  A  T  P  P  A  K  R  S  T  I  Q  T  F  V  Q  E  W  H  N  N  797
Vok     778  A  T  P  P  A  K  R  S  T  I  Q  T  F  V  Q  E  W  H  N  D  797
Cpac_S  780  A  T  P  P  A  K  R  N  A  I  Q  T  F  V  Q  E  W  S  N  N  799
Cfau_S  780  A  T  P  P  A  K  R  S  A  I  Q  T  F  V  Q  E  W  S  N  N  799
Cnau_S  780  A  T  P  P  A  K  R  S  A  I  Q  T  F  V  Q  E  W  S  N  N  799
Pste_S  780  A  T  P  P  A  K  R  S  A  I  Q  T  F  V  Q  E  W  N  N  N  799
Rma     776  A  T  P  P  A  K  R  S  A  I  Q  T  F  V  Q  E  W  N  N  N  795
Ifos_S  775  A  T  P  P  A  K  R  S  A  I  Q  T  F  V  Q  E  W  N  D  N  794
Apha_S  780  A  T  P  P  A  K  R  S  A  I  Q  T  F  V  Q  E  W  D  N  N  799
Bsep_S  777  A  T  P  P  Q  G  R  S  A  I  Q  T  F  V  N  E  W  S  D  A  796
```

```
Akaw_S 2389 AATATCAAAGAAGCAATTACAAGAGAAATACACCGTGGTGGACAGGTTTTTGTACTGCAT 2448
Clau_S 2392 AATATCAAAGAGGCAATTACAAGAGAAATACACCGCGGTGGACAGGTTTTTGTACTGCAT 2451
Pkil_S 2392 AATATCAAAGAGGCAATTACACGAGAAATACACCGTGGTGGGCAGGTTTTCGTACTGCAT 2451
Psoy_S 2392 AATATCAAAGAGGCAATTACACGAGAAATACACCGTGGTGGGCAGGTTTTCGTACTGCAT 2451
Vok    2392 AATATCAAAGAGGCAATTACACGAGAAATACACCGTGGTGGGCAGGTTTTTATACTGCAT 2451
Cpac_S 2398 AACATCAAAGAAGCAATCACACGTGAAATGCATCGTGGTGGTCAGATTTTTGTGCTACAT 2457
Cfau_S 2398 AACATCAAAGAGGCAATCACACGTGAAATACACCGTGGTGGTCAGATTTTTGTGTTACAT 2457
Cnau_S 2398 AACATCAAAGAGGCAATCACACGTGAAATGCACCGTGGTGGTCAGGTTTTTGTACTACAT 2457
Pste_S 2398 AACATCAAAGAGGCAATCACACGTGAAATGCACCGTGGTGGTCAGGTTTTTGTGCTACAT 2457
Rma    2386 AACATCAAAGAGGCAATTACACGTGAAATGCACCGTGGCGGTCAGGTATTTGTACTACAT 2445
Ifos_S 2383 AACATCAAAGAGGCAATCACACGTGAAATGCACCGTGGTGGTCAGGTGTTTGTACTACAC 2442
Apha_S 2398 AACATCAAAGAGGCAATCACACGTGAAATGCACCGTGGCGGTCAGGTATTTGTACTACAC 2457
Bsep_S 2389 ACTATTAAAGAGGTGTGTTCGCGTGAGCTACATCGTGGGGGGCAAATATTTGTCCTGCAT 2448
Akaw_S  797  N  I  K  E  A  I  T  R  E  I  H  R  G  G  Q  V  F  V  L  H  816
Clau_S  798  N  I  K  E  A  I  T  R  E  I  H  R  G  G  Q  V  F  V  L  H  817
Pkil_S  798  N  I  K  E  A  I  T  R  E  I  H  R  G  G  Q  V  F  V  L  H  817
Psoy_S  798  N  I  K  E  A  I  T  R  E  I  H  R  G  G  Q  V  F  V  L  H  817
Vok     798  N  I  K  E  A  I  T  R  E  I  H  R  G  G  Q  V  F  I  L  H  817
Cpac_S  800  N  I  K  E  A  I  T  R  E  M  H  R  G  G  Q  I  F  V  L  H  819
Cfau_S  800  N  I  K  E  A  I  T  R  E  I  H  R  G  G  Q  I  F  V  L  H  819
Cnau_S  800  N  I  K  E  A  I  T  R  E  M  H  R  G  G  Q  V  F  V  L  H  819
Pste_S  800  N  I  K  E  A  I  T  R  E  M  H  R  G  G  Q  V  F  V  L  H  819
Rma     796  N  I  K  E  A  I  T  R  E  M  H  R  G  G  Q  V  F  V  L  H  815
Ifos_S  795  N  I  K  E  A  I  T  R  E  M  H  R  G  G  Q  V  F  V  L  H  814
Apha_S  800  N  I  K  E  A  I  T  R  E  M  H  R  G  G  Q  V  F  V  L  H  819
Bsep_S  797  T  I  K  E  V  C  S  R  E  L  H  R  G  G  Q  I  F  V  L  H  816

E. coli                                                          Y  Y  L  Y

                                                              Motif IV
```

```
Akaw_S 2449 AATGATATTAATTCAATTGACAATATGGCAGAAAATCTAAAACAAATTATACCAAAATTA 2508
Clau_S 2452 AATGATATTAATTCAATTGACAATATGGCAGAAAATCTAAAACAAATTATACCAAAATTA 2511
Pkil_S 2452 AATGATATTAATTCAATTGACAATATGGCAGAAAATCTAAAACAAATTATACCAAAATTA 2511
Psoy_S 2452 AATGATATTAATTCAATTGACAATATGGCAGAAAATCTAAAACAAATTATACCAAAATTA 2511
Vok    2452 AATGATATTAATTCAATTGACAATATGGCAGAAAATCTAAAACAAATTATACCAAAATTA 2511
Cpac_S 2458 AACGACATTGATTCAATTGACAATATGGCAGAAAATCTCGAACAAATTATGCCAAAAGCC 2517
Cfau_S 2458 AACGACATTGATTCAATTGACAATATGGCAAAAAATCTTGAACAAATTATACCAAAAGCC 2517
Cnau_S 2458 AACGACATTGATTCAATTGACAATATGGCAGAAAATCTCAAACAAATTATGCCAAAAGCC 2517
Pste_S 2458 AACAACATTGATTCAATTGACAATATGGCAGAAAATCTCAAACAAATTATGCCAAAAGCC 2517
Rma    2446 AACGATATTGATTCAATTGACAAAATGGTAGAAAATCTCAAACAAATTATGCCAAAAATT 2505
Ifos_S 2443 AACGATATTGATTCAATTGACAATATGGCAGAAAATCTCAAACAAATTATGCCAAAAGTT 2502
Apha_S 2458 AACGATATTGACTCAATTGATAATATGGCAGAAAATCTCAAACAAATTATACCAAAAGTT 2517
Bsep_S 2449 AATGACATTGATAGTATTGATAATATGGCAGAAAAGCTAACATCGCTGATACCCAATGCC 2508
Akaw_S  817  N  D  I  N  S  I  D  N  M  A  E  N  L  K  Q  I  I  P  K  L  836
Clau_S  818  N  D  I  N  S  I  D  N  M  A  E  N  L  K  Q  I  I  P  K  L  837
Pkil_S  818  N  D  I  N  S  I  D  N  M  A  E  N  L  K  Q  I  I  P  K  L  837
Psoy_S  818  N  D  I  N  S  I  D  N  M  A  E  N  L  K  Q  I  I  P  K  L  837
Vok     818  N  D  I  N  S  I  D  N  M  A  E  N  L  K  Q  I  I  P  K  L  837
Cpac_S  820  N  D  I  D  S  I  D  N  M  A  E  N  L  E  Q  I  M  P  K  A  839
Cfau_S  820  N  D  I  D  S  I  D  N  M  A  K  N  L  E  Q  I  I  P  K  A  839
Cnau_S  820  N  D  I  D  S  I  D  N  M  A  E  N  L  K  Q  I  M  P  K  A  839
Pste_S  820  N  N  I  D  S  I  D  N  M  A  E  N  L  K  Q  I  M  P  K  A  839
Rma     816  N  D  I  D  S  I  D  K  M  V  E  N  L  K  Q  I  M  P  K  I  835
Ifos_S  815  N  D  I  D  S  I  D  N  M  A  E  N  L  K  Q  I  M  P  K  V  834
Apha_S  820  N  D  I  D  S  I  D  N  M  A  E  N  L  K  Q  I  I  P  K  V  839
Bsep_S  817  N  D  I  D  S  I  D  N  M  A  E  K  L  T  S  L  I  P  N  A  836

E. coli      N  D  V  E
```

Nucleotide binding region (816-869)

```
Akaw_S 2509 CAGGTTCGTATTGCCCACGGAAAAATTCCAACACGTGAGTTAGAACAAATCATGAGTGAT 2568
Clau_S 2512 CAGATTCGTATCGCCCACGGAAAAATTCCAACACGTGAGTTAGAACAAATCATGAGTGAT 2571
Pkil_S 2512 CAGATTCGTATCGCCCACGGAAAAATTCCAACACGTGAATTAGAACAAATCATGAGTGAT 2571
Psoy_S 2512 CAGATTCGTATCGCCCACGGAAAAATTCCAACACGTGAATTAGAACAAATCATGAGTGAT 2571
Vok    2512 CAGATTCGTATCGCCCATGGAAAAATTCCAACACGTGAGTTAGAACAAATCATGAGTGAT 2571
Cpac_S 2518 CACGTTCGTATTGCCCACGGACAAATGCCAACACGTGAGTTAGAAAGAGTCATGAGTGAC 2577
Cfau_S 2518 CACGTTCGTATTGCCCACGGACAAATGCCAACACGTGAATTAGAAAGAATCATGAGTGAC 2577
Cnau_S 2518 CACGTTCGTATCGCTCACGGACAAATGCCAACACATGAATTAGAAAGAATCATGAGTGAC 2577
Pste_S 2518 CACGTTTGTATCGCCCACGGACAAATGCCAACACGTGAATTAGAAAGAATCATGAGTGAC 2577
Rma    2506 CACGTTCGTATTGCCCACGGACAAATGCCAACGCGTGAACTAAAAAAAATTATGAGTGAT 2565
Ifos_S 2503 CACGTTCGCATCGCCCACGGACAAATACCAACACGTGAACTAGAAAGAATCATGAGTGAT 2562
Apha_S 2518 CACGTTCGTATCGCCCACGGACAAATGCCGACACGTGAACTAGAAAGAGTCATGAGTGAT 2577
Bsep_S 2509 CGTGTGCGTATTGCCCACGGGCAAATGCCAACCCGTGAATTAGAGCAAATTATGAGTGAT 2568
Akaw_S  837   Q  V  R  I  A  H  G  K  I  P  T  R  E  L  E  Q  I  M  S  D  856
Clau_S  838   Q  I  R  I  A  H  G  K  I  P  T  R  E  L  E  Q  I  M  S  D  857
Pkil_S  838   Q  I  R  I  A  H  G  K  I  P  T  R  E  L  E  Q  I  M  S  D  857
Psoy_S  838   Q  I  R  I  A  H  G  K  I  P  T  R  E  L  E  Q  I  M  S  D  857
Vok     838   Q  I  R  I  A  H  G  K  I  P  T  R  E  L  E  Q  I  M  S  D  857
Cpac_S  840   H  V  R  I  A  H  G  Q  M  P  T  R  E  L  E  R  V  M  S  D  859
Cfau_S  840   H  V  R  I  A  H  G  Q  M  P  T  R  E  L  E  R  I  M  S  D  859
Cnau_S  840   H  V  R  I  A  H  G  Q  M  P  T  H  E  L  E  R  I  M  S  D  859
Pste_S  840   H  V  C  I  A  H  G  Q  M  P  T  R  E  L  E  R  I  M  S  D  859
Rma     836   H  V  R  I  A  H  G  Q  M  P  T  R  E  L  K  K  I  M  S  D  855
Ifos_S  835   H  V  R  I  A  H  G  Q  I  P  T  R  E  L  E  R  I  M  S  D  854
Apha_S  840   H  V  R  I  A  H  G  Q  M  P  T  R  E  L  E  R  V  M  S  D  859
Bsep_S  837   R  V  R  I  A  H  G  Q  M  P  T  R  E  L  E  Q  I  M  S  D  856
```

Nucleotide binding region (816-869)

```
Akaw_S 2569 TTTTACCACGCACGTTTCCACATTCTAGTTTGCACCACCATTATTGAAACAGGAATTGAT 2628
Clau_S 2572 TTTTACCACGCACGTTTCCATATTCTAGTTTGCACCACGATTATTGAAACAGGGATTGAT 2631
Pkil_S 2572 TTTTACCACGCACGTTTCCACATTCTAGTTTGCACCACGATTATTGAAACAGGGATTGAT 2631
Psoy_S 2572 TTTTACCACGCACGTTTCCACATTCTAGTTTGCACCACGATTATTGAAACAGGGATTGAT 2631
Vok    2572 TTTTACCACGCACGTTTCCACATTCTAGTTTGCACCACGATTATTGAAACAGGGATTGAT 2631
Cpac_S 2578 TTTTATCATGCGTGTTTCCAAATTTTGGTTTGCACCACAATTATTGAAACAGGGATTGAC 2637
Cfau_S 2578 TTTTATCATGCGTGTTTCCAAATTTTGGTTTGCACCACAATTATTGAAACAGGGATTGAC 2637
Cnau_S 2578 TTCTATCATTCGCGTTTCCAAATTTTGGTTTGCACCACAATTATTGAAACAGGGATTGAC 2637
Pste_S 2578 TTTTATCATGCGCGTTTTCAAATTTTGGTTTGCACCACAATTATTGAAACAGGGATTGAC 2637
Rma    2566 TTTTATCACGCACGTTTCCAAATTTTGGTTTGCACCACAATTATTGAAACAGGGATTGAC 2625
Ifos_S 2563 TTTTATCACGCACGTTTCCAAATTTTGGTTTGCACCACGATTATCGAAACAGGGATTGAC 2622
Apha_S 2578 TTTTATCACGCACGTTTCCAAATTTTAGTTTGCACCACGATTATCGAAACAGGGATTGAC 2637
Bsep_S 2569 TTTTATCATGGTCGCTTCCAAATTCTAGTCTGCACCACCATTATTGAGACAGGCATTGAC 2628
Akaw_S  857   F  Y  H  A  R  F  H  I  L  V  C  T  T  I  I  E  T  G  I  D  876
Clau_S  858   F  Y  H  A  R  F  H  I  L  V  C  T  T  I  I  E  T  G  I  D  877
Pkil_S  858   F  Y  H  A  R  F  H  I  L  V  C  T  T  I  I  E  T  G  I  D  877
Psoy_S  858   F  Y  H  A  R  F  H  I  L  V  C  T  T  I  I  E  T  G  I  D  877
Vok     858   F  Y  H  A  R  F  H  I  L  V  C  T  T  I  I  E  T  G  I  D  877
Cpac_S  860   F  Y  H  A  C  F  Q  I  L  V  C  T  T  I  I  E  T  G  I  D  879
Cfau_S  860   F  Y  H  A  C  F  Q  I  L  V  C  T  T  I  I  E  T  G  I  D  879
Cnau_S  860   F  Y  H  S  R  F  Q  I  L  V  C  T  T  I  I  E  T  G  I  D  879
Pste_S  860   F  Y  H  A  R  F  Q  I  L  V  C  T  T  I  I  E  T  G  I  D  879
Rma     856   F  Y  H  A  R  F  Q  I  L  V  C  T  T  I  I  E  T  G  I  D  875
Ifos_S  855   F  Y  H  A  R  F  Q  I  L  V  C  T  T  I  I  E  T  G  I  D  874
Apha_S  860   F  Y  H  A  R  F  Q  I  L  V  C  T  T  I  I  E  T  G  I  D  879
Bsep_S  857   F  Y  H  G  R  F  Q  I  L  V  C  T  T  I  I  E  T  G  I  D  876

E. coli                              V  C  T  T  I  I  E  T  G  I  D
```

Motif V

Nucleotide binding region (816-869)

```
Akaw_S 2629 ATTCCTAACGCCAATACCATTATTATCAATAATGCACAAAATTTTGGGCTAGCGCAATTA 2688
Clau_S 2632 ATTCCTAACGCCAATACCATCATTATCAATAATGCACAAAATTTTGGTCTAGCACAATTA 2691
Pkil_S 2632 ATTCCTAACGCCAATACTATCATTATCAATAATGCACAAAATTTTGGTCTAGCACAATTA 2691
Psoy_S 2632 ATTCCTAACGCCAATACTATCATTATCAATAATGCACAAAATTTTGGTCTAGCACAATTA 2691
Vok    2632 ATTCCTAACGCCAATACTATCATTATCAATAATGCACAAAATTTTGGTCTAGCACAATTA 2691
Cpac_S 2638 ATTCCCAATGCTAATACTATCATTATCAATAATGCGCAAAAATTTGGTCTGGCACAATTA 2697
Cfau_S 2638 ATTCCCAATGCTAACACTATTATTATCAATAATGCGCAAAAATTTGGTCTGGCACAATTA 2697
Cnau_S 2638 ATTCCCAATGCTAATACTATTATTATCAATAATGCGCAAAATTTTGGTCTGGCACAATTA 2697
Pste_S 2638 ATTCCCAATGCTAATACTATTATTATCAATAATGCGCAAAATTTTGGTCTGGCACAATTA 2697
Rma    2626 ATTCCAAATGCTAATACCATCATTATCAATAATGCACAAAATTTTGGTCTGGCACAATTA 2685
Ifos_S 2623 ATTCCCAATGCTAATACCATCATTATTAATAATGCACAAAATTTTGGTTTGGCACAACTA 2682
Apha_S 2638 ATTCCCAATGCCAATACCATTATTATTATTAATGCACAAAATTTTGGTCTGGCACAACTA 2697
Bsep_S 2629 ATTCCTAATGCCAATACCATTATTATTAACAATGCACAAAATTTTGGCTTGGCGCAATTG 2688
Akaw_S  877   I  P  N  A  N  T  I  I  I  N  N  A  Q  N  F  G  L  A  Q  L  896
Clau_S  878   I  P  N  A  N  T  I  I  I  N  N  A  Q  N  F  G  L  A  Q  L  897
Pkil_S  878   I  P  N  A  N  T  I  I  I  N  N  A  Q  N  F  G  L  A  Q  L  897
Psoy_S  878   I  P  N  A  N  T  I  I  I  N  N  A  Q  N  F  G  L  A  Q  L  897
Vok     878   I  P  N  A  N  T  I  I  I  N  N  A  Q  N  F  G  L  A  Q  L  897
Cpac_S  880   I  P  N  A  N  T  I  I  I  N  N  A  Q  K  F  G  L  A  Q  L  899
Cfau_S  880   I  P  N  A  N  T  I  I  I  N  N  A  Q  K  F  G  L  A  Q  L  899
Cnau_S  880   I  P  N  A  N  T  I  I  I  N  N  A  Q  N  F  G  L  A  Q  L  899
Pste_S  880   I  P  N  A  N  T  I  I  I  N  N  A  Q  N  F  G  L  A  Q  L  899
Rma     876   I  P  N  A  N  T  I  I  I  N  N  A  Q  N  F  G  L  A  Q  L  895
Ifos_S  875   I  P  N  A  N  T  I  I  I  N  N  A  Q  N  F  G  L  A  Q  L  894
Apha_S  880   I  P  N  A  N  T  I  I  I  I  N  A  Q  N  F  G  L  A  Q  L  899
Bsep_S  877   I  P  N  A  N  T  I  I  I  N  N  A  Q  N  F  G  L  A  Q  L  896

E. coli       I  P  T  A  N
```

```
Akaw_S 2689 CATCAACTTAGAGGTCGTGTTGGTCGTTCTCACCACAGGGCTTATGCTTATTTAATTGTT 2748
Clau_S 2692 CATCAACTTAGAGGCCGTGTTGGTCGTTCTCACCACAGGGCTTATGCTTATTTAATTGTT 2751
Pkil_S 2692 CATCAACTCAGAGGTCGTGTTGGTCGTTCTCACCACAGAGCTTATGCTTATTTAATTATT 2751
Psoy_S 2692 CATCAACTCAGAGGTCGTGTTGGTCGTTCTCACCACAGAGCTTATGCTTATTTAATTATT 2751
Vok    2692 CATCAACTTAGAGGTCGTGTTGGTCGTTCTCACCACAGGGCTTATGCTTATTTAATTGTT 2751
Cpac_S 2698 CACCAACTACGGGGTCGTGTTGGTCGCTCCCACCATAGGGCTTACGCTTATTTGATTGTC 2757
Cfau_S 2698 CATCAACTACGAGGTCGTGTTGGTCGTTCCCACCATAGGGCCTACGCTTATTTGATTGTC 2757
Cnau_S 2698 CATCAACTACGAGGTCGTGTTGGTCGCTCCCACCATAGGGCTTACGCTTATTTGATTGTC 2757
Pste_S 2698 CATCAACTACGAGGTCGTGTTGGTCGCTCCCACCATAGGGCTTACGCTTATTTGATTGTC 2757
Rma    2686 CACCAGCTACGAGGTCGTGTTGGTCGTTCCCATCATAGGGCTTATGCCTATTTAATTATT 2745
Ifos_S 2683 CACCAACTACGAGGCCGTGTTGGTCGCTCCCACCATAGAGCTTATGCCTATTTAATTATT 2742
Apha_S 2698 CACCAACTGCGGGGCCGTGTTGGTCGCTCCCACCATAGGGCTTATGCCTATTTAATTATT 2757
Bsep_S 2689 CACCAATTGCGTGGGCGAGTTGGTCGTTCACATCATCGGGCGTATGCGTATTTGGTGATT 2748
Akaw_S  897   H  Q  L  R  G  R  V  G  R  S  H  H  R  A  Y  A  Y  L  I  V  916
Clau_S  898   H  Q  L  R  G  R  V  G  R  S  H  H  R  A  Y  A  Y  L  I  V  917
Pkil_S  898   H  Q  L  R  G  R  V  G  R  S  H  H  R  A  Y  A  Y  L  I  I  917
Psoy_S  898   H  Q  L  R  G  R  V  G  R  S  H  H  R  A  Y  A  Y  L  I  I  917
Vok     898   H  Q  L  R  G  R  V  G  R  S  H  H  R  A  Y  A  Y  L  I  V  917
Cpac_S  900   H  Q  L  R  G  R  V  G  R  S  H  H  R  A  Y  A  Y  L  I  V  919
Cfau_S  900   H  Q  L  R  G  R  V  G  R  S  H  H  R  A  Y  A  Y  L  I  V  919
Cnau_S  900   H  Q  L  R  G  R  V  G  R  S  H  H  R  A  Y  A  Y  L  I  V  919
Pste_S  900   H  Q  L  R  G  R  V  G  R  S  H  H  R  A  Y  A  Y  L  I  V  919
Rma     896   H  Q  L  R  G  R  V  G  R  S  H  H  R  A  Y  A  Y  L  I  I  915
Ifos_S  895   H  Q  L  R  G  R  V  G  R  S  H  H  R  A  Y  A  Y  L  I  I  914
Apha_S  900   H  Q  L  R  G  R  V  G  R  S  H  H  R  A  Y  A  Y  L  I  I  919
Bsep_S  897   H  Q  L  R  G  R  V  G  R  S  H  H  R  A  Y  A  Y  L  V  I  916

E.coli           Q  L  R  G  R  V  G  R

                 Motif VI
```

```
Akaw_S 2749 AAGTCTAATCAGTCTTTATCTAAAATAGCTAAAAATAGACTTGATGTAATTAAGTCTTTA 2808
Clau_S 2752 AATTCTCATCAATCTTTATCTAAAACAGCTAAAAATAGACTTGATGTAATTAAGTCTTTA 2811
Pkil_S 2752 AAGTCTCATCAATCTTTATCTAAAACAGCTAAAGATAGACTTGATGTAATTAAGTCTTTA 2811
Psoy_S 2752 AAGTCTCATCAATCTTTATCTAAAACAGCTAAAGATAGACTTGATGTAATTAAGTCTTTA 2811
Vok    2752 AAGTCTCATCTATCTTTATCTAAAACAGCTAAAGATAGACTTGATGTAATTAAGTCTTTA 2811
Cpac_S 2758 AAGTCTCATCAATCACTATCCAAAACAGCCAAAAAACGTCTCGATGCCATTGAATCTTTA 2817
Cfau_S 2758 AAGTCTCATCAATCACTATCCAGAACAGCCAAAAAACGTCTCGATGCCATTGAATCTTTA 2817
Cnau_S 2758 AAATCTCATCAATCACTATCCAAAACAGCCAAAAAACGCCTCGATGCCATTGAATCTTTA 2817
Pste_S 2758 AAGTCTCATCAATCACTATCCAAAACAGCCAAAAAACGCCTCGATTCCATTGAATCTTTA 2817
Rma    2746 AAATCTTATCAATTATTATCCAAAACAGCCAAAAAACGTCTAGATGTCATTGAATCTTTA 2805
Ifos_S 2743 AAATCCCATCAATCGTTATCTAAAACAGCCAAAAAACGCCTTGATACCATTGAGTCTTTA 2802
Apha_S 2758 AAATCTCATCAATCACTATCTAAAACAGCCAAAAAACGCCTTGATGCCGTTGAGTCTTTA 2817
Bsep_S 2749 AAATCGCACCAGTCACTGAGTAAAAATGCCAAAAAACGCTTAGATGCCGTCGAGTCTTTA 2808
Akaw_S  917   K  S  N  Q  S  L  S  K  I  A  K  N  R  L  D  V  I  K  S  L  936
Clau_S  918   N  S  H  Q  S  L  S  K  T  A  K  N  R  L  D  V  I  K  S  L  937
Pkil_S  918   K  S  H  Q  S  L  S  K  T  A  K  D  R  L  D  V  I  K  S  L  937
Psoy_S  918   K  S  H  Q  S  L  S  K  T  A  K  D  R  L  D  V  I  K  S  L  937
Vok     918   K  S  H  L  S  L  S  K  T  A  K  D  R  L  D  V  I  K  S  L  937
Cpac_S  920   K  S  H  Q  S  L  S  K  T  A  K  K  R  L  D  A  I  E  S  L  939
Cfau_S  920   K  S  H  Q  S  L  S  R  T  A  K  K  R  L  D  A  I  E  S  L  939
Cnau_S  920   K  S  H  Q  S  L  S  K  T  A  K  K  R  L  D  A  I  E  S  L  939
Pste_S  920   K  S  H  Q  S  L  S  K  T  A  K  K  R  L  D  S  I  E  S  L  939
Rma     916   K  S  Y  Q  L  L  S  K  T  A  K  K  R  L  D  V  I  E  S  L  935
Ifos_S  915   K  S  H  Q  S  L  S  K  T  A  K  K  R  L  D  T  I  E  S  L  934
Apha_S  920   K  S  H  Q  S  L  S  K  T  A  K  K  R  L  D  A  V  E  S  L  939
Bsep_S  917   K  S  H  Q  S  L  S  K  N  A  K  K  R  L  D  A  V  E  S  L  936
```

```
Akaw_S 2809 ACAAAACTTGGTTCTGGGTTTATGTTAGCTAATCACGATCTTGAAATTCGTGGTGCAGGT 2868
Clau_S 2812 ACAAAACTTGGCTCTGGGTTTATTTTAGCTAATCACGATCTTGAAATTCGTGGTGCAGGT 2871
Pkil_S 2812 ACAGAACTTGGTTCTGGGTTTATGTTGGCTAATCACGATCTTGAAATTCGTGGCGCAGGT 2871
Psoy_S 2812 ACAGAACTTGGTTCTGGGTTTATGTTGGCTAATCACGATCTTGAAATTCGTGGCGCAGGT 2871
Vok    2812 ACAGAACTTGGTTCTGGGTTTATGTTGGCTAATCACGATCTTGAAATTCGTGGTGCAGGT 2871
Cpac_S 2818 AAAGATCTTGGTACTGGATTTATGCTGGCTAATCACGACCTTGAAATTCGTGGTGCGGGT 2877
Cfau_S 2818 AAAGAGCTTGGTACTGGATTTATGCTGGCTAATCACGACCTTGAAATTCGTGGTGCGGGT 2877
Cnau_S 2818 AAAGAGCTTGGGGCTGGATTTATGCTGGCTAATCACGACCTTGAAATTCGTGGTGCGGGT 2877
Pste_S 2818 GAAGAGCTTGGTGCTGGATTTATGCTGGCTAATCACGACCTTGAAATTCGTGGTGCGGGT 2877
Rma    2806 GAAGAGCTTGGTGCTGGATTTATACTAGCCAATCACGACCTTGAAATTCGTGGTGCGGGT 2865
Ifos_S 2803 GAGGGGCTTGGTGCTGGATTTATGCTGGCTAATCACGACCTTGAAATTCGTGGTGCGGGT 2862
Apha_S 2818 GAAGAGCTTGGTGCTGGATTTATGCTAGCTAATCACGACCTTGAAATTCGTGGTGCGGGT 2877
Bsep_S 2809 GAGGAGTTAGGTGCAGGCTTTATGCTCGCCAATCATGACTTAGAAATCCGTGGTGCGGGC 2868
Akaw_S  937   T  K  L  G  S  G  F  M  L  A  N  H  D  L  E  I  R  G  A  G  956
Clau_S  938   T  K  L  G  S  G  F  I  L  A  N  H  D  L  E  I  R  G  A  G  957
Pkil_S  938   T  E  L  G  S  G  F  M  L  A  N  H  D  L  E  I  R  G  A  G  957
Psoy_S  938   T  E  L  G  S  G  F  M  L  A  N  H  D  L  E  I  R  G  A  G  957
Vok     938   T  E  L  G  S  G  F  M  L  A  N  H  D  L  E  I  R  G  A  G  957
Cpac_S  940   K  D  L  G  T  G  F  M  L  A  N  H  D  L  E  I  R  G  A  G  959
Cfau_S  940   K  E  L  G  T  G  F  M  L  A  N  H  D  L  E  I  R  G  A  G  959
Cnau_S  940   K  E  L  G  A  G  F  M  L  A  N  H  D  L  E  I  R  G  A  G  959
Pste_S  940   E  E  L  G  A  G  F  M  L  A  N  H  D  L  E  I  R  G  A  G  959
Rma     936   E  E  L  G  A  G  F  I  L  A  N  H  D  L  E  I  R  G  A  G  955
Ifos_S  935   E  G  L  G  A  G  F  M  L  A  N  H  D  L  E  I  R  G  A  G  954
Apha_S  940   E  E  L  G  A  G  F  M  L  A  N  H  D  L  E  I  R  G  A  G  959
Bsep_S  937   E  E  L  G  A  G  F  M  L  A  N  H  D  L  E  I  R  G  A  G  956
```

```
Akaw_S 2869 GATTTACTCGGTGATAATCAATCTGGACAAATCAGCGAGATTGGTTTTAACCTTTACCAT 2928
Clau_S 2872 GATTTACTCGGTGATAATCAATCTGGACAAATTAGAGAAATTGGTTTTAACCTTTACCAT 2931
Pkil_S 2872 GATTTACTCGGTGATAATCAATCTGGACAAATCAGCGAAATTGGTTTTAACCTTTACCAT 2931
Psoy_S 2872 GATTTACTCGGTGATAATCAATCTGGACAAATCAGCGAAATTGGTTTTAACCTTTACCAT 2931
Vok    2872 GATCTACTCGGTGATAATCAATCTGGACAAATCAGCGAAATTGGTTTTAACCTTTACCAT 2931
Cpac_S 2878 GATTTATTGGGTGACAATCAGTCTGGAAGAATTAGCGAAATTGGTTTTAACCTTTATCAT 2937
Cfau_S 2878 GATTTATTGGGTGACAATCAGTCTGGAAAAATTAGCGAAATTGGTTTTAATCTTTATCAT 2937
Cnau_S 2878 GATTTATTGGGTGACAATCAATCTGGAAAAATTAGCGAAATTGGTTTTAATCTTTATCAT 2937
Pste_S 2878 GATTTATTGGGTGACAATCAATCTGGAAAAATTAGCGAAATTGGTTTTAATCTTTATCAT 2937
Rma    2866 GATTTATTGGGCGATAATCAATCTGGAAAAATTAACGAAATTGGCTTTAATCTTTATCAT 2925
Ifos_S 2863 GATTTATTGGGTGATAATCAATCTGGAAAAATTAGCGAAATTGGCTTTAATCTTTATCAT 2922
Apha_S 2878 GATTTATTGGGTGATAATCAATCTGGAAAAATTAGCGAAATTGGCTTTAATCTTTACCAT 2937
Bsep_S 2869 GATTTGCTTGGCGATAATCAGTCGGGAAAAATCAGCGAAATCGGTTTTAATATGTATCAT 2928
Akaw_S  957   D  L  L  G  D  N  Q  S  G  Q  I  S  E  I  G  F  N  L  Y  H  976
Clau_S  958   D  L  L  G  D  N  Q  S  G  Q  I  R  E  I  G  F  N  L  Y  H  977
Pkil_S  958   D  L  L  G  D  N  Q  S  G  Q  I  S  E  I  G  F  N  L  Y  H  977
Psoy_S  958   D  L  L  G  D  N  Q  S  G  Q  I  S  E  I  G  F  N  L  Y  H  977
Vok     958   D  L  L  G  D  N  Q  S  G  Q  I  S  E  I  G  F  N  L  Y  H  977
Cpac_S  960   D  L  L  G  D  N  Q  S  G  R  I  S  E  I  G  F  N  L  Y  H  979
Cfau_S  960   D  L  L  G  D  N  Q  S  G  K  I  S  E  I  G  F  N  L  Y  H  979
Cnau_S  960   D  L  L  G  D  N  Q  S  G  K  I  S  E  I  G  F  N  L  Y  H  979
Pste_S  960   D  L  L  G  D  N  Q  S  G  K  I  S  E  I  G  F  N  L  Y  H  979
Rma     956   D  L  L  G  D  N  Q  S  G  K  I  N  E  I  G  F  N  L  Y  H  975
Ifos_S  955   D  L  L  G  D  N  Q  S  G  K  I  S  E  I  G  F  N  L  Y  H  974
Apha_S  960   D  L  L  G  D  N  Q  S  G  K  I  S  E  I  G  F  N  L  Y  H  979
Bsep_S  957   D  L  L  G  D  N  Q  S  G  K  I  S  E  I  G  F  N  M  Y  H  976
```

```
Akaw_S 2929 GATTTATTAAAACGCACCATTAATTCCATAAACTCTGGTAAAAAAATTAACCTTAATAAT 2988
Clau_S 2932 GATTTATTGAAACGTACTATTAATGCCATACACTCTGGTAACAAAATTAACCTTAATGAT 2991
Pkil_S 2932 GATTTATTGAAACGCACCATTGATGCCATATACTCTGGTAAAAAAATTAACCTTAATGAT 2991
Psoy_S 2932 GATTTATTGAAACGCACCATTGATGCCATATACTCTGGTAAAAAAATTAACCTTAATGAT 2991
Vok    2932 GATTTATTGAAACGCACCATTGATGCCGTGTACTCTGGTAAAAAAATTAACCTTAATGAT 2991
Cpac_S 2938 GATTTATTAAAACGCACTATTGATGCCATGCGCTCTGGTAGAAAAATTAACCTTAACGAC 2997
Cfau_S 2938 GATTTATTAAAACGCACTATTGATGCCATGCGCTCTGGTAGAAAAATTAACCTTAACGAC 2997
Cnau_S 2938 GATTTATTAAAACGCACTATTGATGCCATACGCTCTGGTAGAAAAATTAACCTTAACGAC 2997
Pste_S 2938 GATTTATTAAAACGCACTATTGATGCCATGCGCTCTGGTAGAAAAATTAACCTTAACGAC 2997
Rma    2926 GATTTATTAAAACGTACCATTGACACCCTGCGTTCTGGCAGGAAAATTAACTTTGATGAT 2985
Ifos_S 2923 GATTTATTAAAACGCACTATTGATGCCATGCGCTCTGGTAGAAAAATTAACCTTGATGAC 2982
Apha_S 2938 GATTTATTAAAACGCACCATTGATGCCATGCGCTCTGGTAGAAAAATTAACCTTAACGAC 2997
Bsep_S 2929 GACCTGCTCAAACGCACCGTTGATGCAATGCGCGTGGGCAGAAAAATAAACCTCAACAAC 2988
Akaw_S  977   D  L  L  K  R  T  I  N  S  I  N  S  G  K  K  I  N  L  N  N  996
Clau_S  978   D  L  L  K  R  T  I  N  A  I  H  S  G  N  K  I  N  L  N  D  997
Pkil_S  978   D  L  L  K  R  T  I  D  A  I  Y  S  G  K  K  I  N  L  N  D  997
Psoy_S  978   D  L  L  K  R  T  I  D  A  I  Y  S  G  K  K  I  N  L  N  D  997
Vok     978   D  L  L  K  R  T  I  D  A  V  Y  S  G  K  K  I  N  L  N  D  997
Cpac_S  980   D  L  L  K  R  T  I  D  A  M  R  S  G  R  K  I  N  L  N  D  999
Cfau_S  980   D  L  L  K  R  T  I  D  A  M  R  S  G  R  K  I  N  L  N  D  999
Cnau_S  980   D  L  L  K  R  T  I  D  A  I  R  S  G  R  K  I  N  L  N  D  999
Pste_S  980   D  L  L  K  R  T  I  D  A  M  R  S  G  R  K  I  N  L  N  D  999
Rma     976   D  L  L  K  R  T  I  D  T  L  R  S  G  R  K  I  N  F  D  D  995
Ifos_S  975   D  L  L  K  R  T  I  D  A  M  R  S  G  R  K  I  N  L  D  D  994
Apha_S  980   D  L  L  K  R  T  I  D  A  M  R  S  G  R  K  I  N  L  N  D  999
Bsep_S  977   D  L  L  K  R  T  V  D  A  M  R  V  G  R  K  I  N  L  N  N  996
```

```
Akaw_S 2989 CCAATTAACCATGAAGTACAAATTGATTCTGGCTTACCATCAATTATTCCAGAAACCTAT 3048
Clau_S 2992 TCAATTAACCATGAAGTACAAATTGATTCTGGTTTACCATCAATTATTCCAGAAACCTAT 3051
Pkil_S 2992 CCAATTAACCATGAAGTACAAATTGATTCTGGCCTACCATCAATTATTCCAGAAACCTAT 3051
Psoy_S 2992 CCAATTAACCATGAAGTACAAATTGATTCTGGCCTACCATCAATTATTCCAGAAACCTAT 3051
Vok    2992 CCAATTAACCATGAAGTACAAATTGATTCTGGCTTACCATCAATTATTCCAGAAACCTAT 3051
Cpac_S 2998 CCAATTAATCATGAAGTGAAAATTGATTCTGGCTTGCCATCCATTATTCCAGAAACCTAT 3057
Cfau_S 2998 CCAATTAATCATGAAGTGAAAATTAATTCTGGCTTGCCATCCATTATTCCAGAAACCTAT 3057
Cnau_S 2998 CCAATTAATCATGAAGTGCAAATTGATTCTGGCTTGCCATCCATTATTCCAGAAACCTAT 3057
Pste_S 2998 CCAATTAATCATGAAGTGCAAATTGATTCTGGCTTACCATCCATTATTCCAGAAACCTAT 3057
Rma    2986 CCAATCAGTCATGAAGTGAAAATTGATTCTGGTTTGCCATCTATCATTCCAGAGACGTAT 3045
Ifos_S 2983 CCCATCAATCATGAAGTACAAATTGATTCTGGCTTGCCATCTATCATTCCAGAGGCATAT 3042
Apha_S 2998 CCAATTAATCATGAAGTGAAAATTGATTCTGGCTTGCCATCTATCATTCCAGAGGCGTAT 3057
Bsep_S 2989 CCACTCAACCACGAAATTGAAATTGATACAGGGCTCCCCTGCCTTATCCCCGAAAGTTAT 3048
Akaw_S  997   P  I  N  H  E  V  Q  I  D  S  G  L  P  S  I  I  P  E  T  Y 1016
Clau_S  998   S  I  N  H  E  V  Q  I  D  S  G  L  P  S  I  I  P  E  T  Y 1017
Pkil_S  998   P  I  N  H  E  V  Q  I  D  S  G  L  P  S  I  I  P  E  T  Y 1017
Psoy_S  998   P  I  N  H  E  V  Q  I  D  S  G  L  P  S  I  I  P  E  T  Y 1017
Vok     998   P  I  N  H  E  V  Q  I  D  S  G  L  P  S  I  I  P  E  T  Y 1017
Cpac_S 1000   P  I  N  H  E  V  K  I  D  S  G  L  P  S  I  I  P  E  T  Y 1019
Cfau_S 1000   P  I  N  H  E  V  K  I  N  S  G  L  P  S  I  I  P  E  T  Y 1019
Cnau_S 1000   P  I  N  H  E  V  Q  I  D  S  G  L  P  S  I  I  P  E  T  Y 1019
Pste_S 1000   P  I  N  H  E  V  Q  I  D  S  G  L  P  S  I  I  P  E  T  Y 1019
Rma     996   P  I  S  H  E  V  K  I  D  S  G  L  P  S  I  I  P  E  T  Y 1015
Ifos_S  995   P  I  N  H  E  V  Q  I  D  S  G  L  P  S  I  I  P  E  A  Y 1014
Apha_S 1000   P  I  N  H  E  V  K  I  D  S  G  L  P  S  I  I  P  E  A  Y 1019
Bsep_S  997   P  L  N  H  E  I  E  I  D  T  G  L  P  C  L  I  P  E  S  Y 1016
```

transcription-repair-coupling factor (TRCF) (1004-1104)

```
Akaw_S 3049 ATTTTTGATGTTCATGAACGACTCGTTCTTTATAAACGCATTGCCAATTGTCAAAATAAC 3108
Clau_S 3052 ATTTTTGATGTTCATGAACGGCTTGTGATTTATAAACGCATTGCTAATTGTCAAAACAAC 3111
Pkil_S 3052 ATTTTTGATGTTCATGAACGACTTGTGCTTTATAAACGCATTGCCAATTGTCAAAACAAC 3111
Psoy_S 3052 ATTTTTGATGTTCATGAACGACTTGTGCTTTATAAACGCATTGCCAATTGTCAAAACAAC 3111
Vok    3052 ATTTTTGATGTTCATGAACGGCTTGTGCTTTATAAACGCATTGCCAATTGTCAAAACAAC 3111
Cpac_S 3058 ATTTTTGATGTGCACGAACGACTGGTACTCTACAAGCGTATTGCCAATTGTCAAAACAAT 3117
Cfau_S 3058 ATTTTTGATGTGCACGAACGACTGGTATTCTACAAACGCATTGCCAATTGCCAAAACAAT 3117
Cnau_S 3058 ATTTTTGATGTGCACGAACGACTGGTACTCTACAAGCGCATTGCCAATTGCCAAAACAAT 3117
Pste_S 3058 ATTTTTGATGTACACGAACGACTGGTACTCTACAAACGCATTGCCAATTGCCAAAACAAT 3117
Rma    3046 ATTTTTGATGTACACCAACGACTTGTACTCTATAAACGTATTGCAAGTTGTCAAAACAAT 3105
Ifos_S 3043 ATTTTTGACGTACACGAACGACTTGTACTCTACAAGCGTATTGCCAGTTGTCAAAACAAT 3102
Apha_S 3058 ATTTTTGACGTGCACGAACGACTCGTACTCTACAAGCGCATTGCCAGTTGCCAAAACAAT 3117
Bsep_S 3049 CTTGGTGATGTCCACGAACGCTTGGTGCTTTACAAGCGCATCGCCAGCGCCAAAAATAAT 3108
Akaw_S 1017   I  F  D  V  H  E  R  L  V  L  Y  K  R  I  A  N  C  Q  N  N 1036
Clau_S 1018   I  F  D  V  H  E  R  L  V  I  Y  K  R  I  A  N  C  Q  N  N 1037
Pkil_S 1018   I  F  D  V  H  E  R  L  V  L  Y  K  R  I  A  N  C  Q  N  N 1037
Psoy_S 1018   I  F  D  V  H  E  R  L  V  L  Y  K  R  I  A  N  C  Q  N  N 1037
Vok    1018   I  F  D  V  H  E  R  L  V  L  Y  K  R  I  A  N  C  Q  N  N 1037
Cpac_S 1020   I  F  D  V  H  E  R  L  V  L  Y  K  R  I  A  N  C  Q  N  N 1039
Cfau_S 1020   I  F  D  V  H  E  R  L  V  F  Y  K  R  I  A  N  C  Q  N  N 1039
Cnau_S 1020   I  F  D  V  H  E  R  L  V  L  Y  K  R  I  A  N  C  Q  N  N 1039
Pste_S 1020   I  F  D  V  H  E  R  L  V  L  Y  K  R  I  A  N  C  Q  N  N 1039
Rma    1016   I  F  D  V  H  Q  R  L  V  L  Y  K  R  I  A  S  C  Q  N  N 1035
Ifos_S 1015   I  F  D  V  H  E  R  L  V  L  Y  K  R  I  A  S  C  Q  N  N 1034
Apha_S 1020   I  F  D  V  H  E  R  L  V  L  Y  K  R  I  A  S  C  Q  N  N 1039
Bsep_S 1017   L  G  D  V  H  E  R  L  V  L  Y  K  R  I  A  S  A  K  N  N 1036
```

transcription-repair-coupling factor (TRCF) (1004-1104)

```
Akaw_S 3109 AATGAACTTAAAGCCCTGCAAATTGAGATGATTGATCGTTTCGGCTTATTGCCAGATTCA 3168
Clau_S 3112 AATGAACTTAAAGCCTTGCAAATTGAGATGATTGACCGTTTTGGTCTATTACCAGATTCA 3171
Pkil_S 3112 AATGAACTCAAAGCCTTGCAAATTGAAATGATTGATCGTTTTGGTCTATTGCCAGATTCA 3171
Psoy_S 3112 AATGAACTCAAAGCCTTGCAAATTGAAATGATTGATCGTTTTGGTCTATTGCCAGATTCA 3171
Vok    3112 AATGAACTTAAAGCCTTGCAAATTGAAATGATTGATCGTTTTGGTCTGTTGCCAGATTCA 3171
Cpac_S 3118 AAGGAGCTTAAGGACTTGAAAATTGAGATGATTGACCGCTTTGGATTATTACCAAATTCA 3177
Cfau_S 3118 AAGGAGCTTAAAGCCTTGCAAATTGAGATGATTGACCGTTTTGGATTATTACCAGATTCA 3177
Cnau_S 3118 AAGGAGCTTAAAGCCTTGCAAATTGAGATGATTGACCGCTTTGGATTATTACCAGATTCA 3177
Pste_S 3118 AAGGAGCTTAAAGCCTTGCAAATTGAGATGATTGACCGCTTTGGATTATTACCAGATTCA 3177
Rma    3106 AAAGAACTTAAAGCATTACAAATTGAGATGATTGATCGTTTTGGATTATTACCAGATTCA 3165
Ifos_S 3103 AAAGAACTTAAAGCTCTACAGATTGAGATGATTGACCGCTTTGGATTATTACCAGATTCA 3162
Apha_S 3118 AAAGAACTTAGAGCTCTACAAATTGAGATGATTGACCGTTTTGGATTATTACCAAATTCA 3177
Bsep_S 3109 ACCGAATTAAAAGACCTGCAAATAGAAATGATTGACCGCTTCGGACTGCTCAAAGATGCC 3168
Akaw_S 1037   N  E  L  K  A  L  Q  I  E  M  I  D  R  F  G  L  L  P  D  S 1056
Clau_S 1038   N  E  L  K  A  L  Q  I  E  M  I  D  R  F  G  L  L  P  D  S 1057
Pkil_S 1038   N  E  L  K  A  L  Q  I  E  M  I  D  R  F  G  L  L  P  D  S 1057
Psoy_S 1038   N  E  L  K  A  L  Q  I  E  M  I  D  R  F  G  L  L  P  D  S 1057
Vok    1038   N  E  L  K  A  L  Q  I  E  M  I  D  R  F  G  L  L  P  D  S 1057
Cpac_S 1040   K  E  L  K  D  L  K  I  E  M  I  D  R  F  G  L  L  P  N  S 1059
Cfau_S 1040   K  E  L  K  A  L  Q  I  E  M  I  D  R  F  G  L  L  P  D  S 1059
Cnau_S 1040   K  E  L  K  A  L  Q  I  E  M  I  D  R  F  G  L  L  P  D  S 1059
Pste_S 1040   K  E  L  K  A  L  Q  I  E  M  I  D  R  F  G  L  L  P  D  S 1059
Rma    1036   K  E  L  K  A  L  Q  I  E  M  I  D  R  F  G  L  L  P  D  S 1055
Ifos_S 1035   K  E  L  K  A  L  Q  I  E  M  I  D  R  F  G  L  L  P  D  S 1054
Apha_S 1040   K  E  L  R  A  L  Q  I  E  M  I  D  R  F  G  L  L  P  N  S 1059
Bsep_S 1037   T  E  L  K  D  L  Q  I  E  M  I  D  R  F  G  L  L  K  D  A 1056
```

transcription-repair-coupling factor (TRCF) (1004-1104)

```
Akaw_S 3169 ACTAAAAACTTACTTTACAATACTAAACTAAAACTCTTTTCCCAAATAATTGGGGTTAAC 3228
Clau_S 3172 ACTAAAAACTTACTTTATAATACCAAACTAAAGCTCTTTTCCCAAATAATTGGGGTTAAC 3231
Pkil_S 3172 ACTAAAAACTTACTTTACAATACCAAGTTGAAACTCTTTTCCCAAATAATTGGGGTTAAC 3231
Psoy_S 3172 ACTAAAAACTTACTTTACAATACCAAGTTGAAACTCTTTTCCCAAATAATTGGGGTTAAC 3231
Vok    3172 ACTAAAAACTTACTTTACAATACCAAACTAAAACTCTTTTCCCAAATAATTGGGGTGAAC 3231
Cpac_S 3178 ACCAAGAACTTATTTGTCAATACCAAACTCAAGCTCTTTTCACAAAAAATTGGCATTGAT 3237
Cfau_S 3178 ACTAAAAACTTATTTGTCAATATCAAACTCAAACTCTTTTCACAAAAAATTGGCATTGAT 3237
Cnau_S 3178 ACCAAAAACTTATTTTCCAATACCAAACTCAAACTCTTTTCACAAAAAATTGGCATTGAT 3237
Pste_S 3178 ACCAAAAACTTATTTGCCAATAACAAACTCAAGCTCTTTTCACAAAAAATTGGCATTGAT 3237
Rma    3166 ACCAAAAATTTATTTGCCAATACTAAACTCAAGCTCTTTTCACAAAAAATTGGCATTGAT 3225
Ifos_S 3163 ACCAAAAATTTATTTGCCAATACCAAACTTAAGCTCTTTTCAAAAAAAATTGGCATTGAT 3222
Apha_S 3178 ACCAAAAACTTATTTGCTAATACCAAACTCAAGCTCTTTTCACAAAAAATTGGTATTGAT 3237
Bsep_S 3169 AGCAAAAACCTATTCGCCGTTACCCAACTCAAACTATTCTGTGAAAAAATCGGCATTAAC 3228
Akaw_S 1057   T  K  N  L  L  Y  N  T  K  L  K  L  F  S  Q  I  I  G  V  N 1076
Clau_S 1058   T  K  N  L  L  Y  N  T  K  L  K  L  F  S  Q  I  I  G  V  N 1077
Pkil_S 1058   T  K  N  L  L  Y  N  T  K  L  K  L  F  S  Q  I  I  G  V  N 1077
Psoy_S 1058   T  K  N  L  L  Y  N  T  K  L  K  L  F  S  Q  I  I  G  V  N 1077
Vok    1058   T  K  N  L  L  Y  N  T  K  L  K  L  F  S  Q  I  I  G  V  N 1077
Cpac_S 1060   T  K  N  L  F  V  N  T  K  L  K  L  F  S  Q  K  I  G  I  D 1079
Cfau_S 1060   T  K  N  L  F  V  N  I  K  L  K  L  F  S  Q  K  I  G  I  D 1079
Cnau_S 1060   T  K  N  L  F  S  N  T  K  L  K  L  F  S  Q  K  I  G  I  D 1079
Pste_S 1060   T  K  N  L  F  A  N  N  K  L  K  L  F  S  Q  K  I  G  I  D 1079
Rma    1056   T  K  N  L  F  A  N  T  K  L  K  L  F  S  Q  K  I  G  I  D 1075
Ifos_S 1055   T  K  N  L  F  A  N  T  K  L  K  L  F  S  K  K  I  G  I  D 1074
Apha_S 1060   T  K  N  L  F  A  N  T  K  L  K  L  F  S  Q  K  I  G  I  D 1079
Bsep_S 1057   S  K  N  L  F  A  V  T  Q  L  K  L  F  C  E  K  I  G  I  N 1076
```

transcription-repair-coupling factor (TRCF) (1004-1104)

```
Akaw_S 3229 AAAATAATCCTTTATGATGACAAAGCCATCATTACCTTTAACAAAAAAAACACTATTGAT 3288
Clau_S 3232 AAAATAATACTTTATGAAGACAAAGCTATAATTACCTTTAACAAAAAAAATACCATTGAT 3291
Pkil_S 3232 AAAATAATCCTTTATGAAGACAAGGCTATCATAACCTTTAACAAAAAAAACACCATTGAT 3291
Psoy_S 3232 AAAATAATCCTTTATGAAGACAAGGCTATCATAACCTTTAACAAAAAAAACACCATTGAT 3291
Vok    3232 AAAATAATCCTTTATGAAGACAAGGCCATCATTACCTTTAACAAAAAAAACACCATTGAT 3291
Cpac_S 3238 GAAATCAACCTCTATGAAGACAAAGCTATCATCACCTTTGGTAGTAAAAATACCATTGAA 3297
Cfau_S 3238 GAAATCAACCTCTATGAAGACAAAGCCATCATCACCTTTGGCAGTAAAAATACCATTAAG 3297
Cnau_S 3238 GAAATCAACCTCTATGAAGACAAAGCCATCATCACCTTTGGCAGTAAAAATACCATTGAG 3297
Pste_S 3238 GAAATCAACCTCTATGAAGACAAGGCCATCATCACCTTTGGCAGTAAAAATACCATTGAG 3297
Rma    3226 AAAATCAGTCTCTATGAAAACAAAGCTATCATCACCTTTGGCAATAAAAATACCATCGAA 3285
Ifos_S 3223 AAAATTAACCTTTATGAAAACAAAGCCATTATCACCTTTGGTAATAAGAATACCATTGAG 3282
Apha_S 3238 GAAATCAACCTCTATAAAGACAAAGCCATCATTACCTTTGGTAATAAAAATGCCATTGAG 3297
Bsep_S 3229 AAAGTCTCAATCTTTGAGGGCAAAGTTCATCTAACCTTTGCCGACAAAACTACTATTGAG 3288
Akaw_S 1077  K  I  I  L  Y  D  D  K  A  I  I  T  F  N  K  K  N  T  I  D 1096
Clau_S 1078  K  I  I  L  Y  E  D  K  A  I  I  T  F  N  K  K  N  T  I  D 1097
Pkil_S 1078  K  I  I  L  Y  E  D  K  A  I  I  T  F  N  K  K  N  T  I  D 1097
Psoy_S 1078  K  I  I  L  Y  E  D  K  A  I  I  T  F  N  K  K  N  T  I  D 1097
Vok    1078  K  I  I  L  Y  E  D  K  A  I  I  T  F  N  K  K  N  T  I  D 1097
Cpac_S 1080  E  I  N  L  Y  E  D  K  A  I  I  T  F  G  S  K  N  T  I  E 1099
Cfau_S 1080  E  I  N  L  Y  E  D  K  A  I  I  T  F  G  S  K  N  T  I  K 1099
Cnau_S 1080  E  I  N  L  Y  E  D  K  A  I  I  T  F  G  S  K  N  T  I  E 1099
Pste_S 1080  E  I  N  L  Y  E  D  K  A  I  I  T  F  G  S  K  N  T  I  E 1099
Rma    1076  K  I  S  L  Y  E  N  K  A  I  I  T  F  G  N  K  N  T  I  E 1095
Ifos_S 1075  K  I  N  L  Y  E  N  K  A  I  I  T  F  G  N  K  N  T  I  E 1094
Apha_S 1080  E  I  N  L  Y  K  D  K  A  I  I  T  F  G  N  K  N  A  I  E 1099
Bsep_S 1077  K  V  S  I  F  E  G  K  V  H  L  T  F  A  D  K  T  T  I  E 1096
```

transcription-repair-coupling factor (TRCF) (1004-1104)

```
Akaw_S 3289 CCTACAAAAATTATCAACCTTATCCAAAAACAAGCAAAAAAAATATCAGTTAAAAGATCAA 3348
Clau_S 3292 CCTATCAAAATCACCAACCTTATCCAAAAACAAGAAAAAAAAATATCAACTAAAAGATCAA 3351
Pkil_S 3292 CCTATAAAAATCACCAACCTTATCCAAAAACAAGCAAAAAAAATATCAGCTAAAAAATCAA 3351
Psoy_S 3292 CCTATAAAAATCACCAACCTTATCCAAAAACAAGCAAAAAAAATATCAGCTAAAAAATCAA 3351
Vok    3292 CCTATAAAAATCACCAACCTTATCCAAAAACAAGCAAAAAAAATATCAGCTAAAAGATCAA 3351
Cpac_S 3298 CCAATCAAAATCATCAATCTTATTCAAAAACAAGCGAAAAAAATATCAGTTAAAGGGTCAA 3357
Cfau_S 3298 CCAATCAAAATCATCAATCTTATTCAAAAACAAGCGAAAAAAATATCAATTAAAGGGTCAA 3357
Cnau_S 3298 CCAATCAAAATCATCAATCTTATTCAAAAACAAGCGAAAAAAATATCAGTTAAAGGGTCAA 3357
Pste_S 3298 CCAATCAAAATCATCAATCTTATTCAAAAACAAGCGAAAAAAATATCAGTTAAAGGGTCAA 3357
Rma    3286 CCAATAAAAATAATCAACCTTATTCAAAAGCAAACGAAAAAAATATCAGCTAAAGGATCAA 3345
Ifos_S 3283 CCGATAAAAATCATCAACCTTATTCAAAAACAAGCAAAAAAAATATCAGCTAAAGGGTCAA 3342
Apha_S 3298 CCGATAAGAATCATCAACCTTATTCAAAAGCAAGCAAAAAAAATATCAGCTAAAGGGTCAA 3357
Bsep_S 3289 CCAATAACAATCATCAATTTAATACAAACCCAACCACGAACTTATCGAATTAAAGGACAA 3348
Akaw_S 1097  P  T  K  I  I  N  L  I  Q  K  Q  A  K  K  Y  Q  L  K  D  Q 1116
Clau_S 1098  P  I  K  I  T  N  L  I  Q  K  Q  E  K  K  Y  Q  L  K  D  Q 1117
Pkil_S 1098  P  I  K  I  T  N  L  I  Q  K  Q  A  K  K  Y  Q  L  K  N  Q 1117
Psoy_S 1098  P  I  K  I  T  N  L  I  Q  K  Q  A  K  K  Y  Q  L  K  N  Q 1117
Vok    1098  P  I  K  I  T  N  L  I  Q  K  Q  A  K  K  Y  Q  L  K  D  Q 1117
Cpac_S 1100  P  I  K  I  I  N  L  I  Q  K  Q  A  K  K  Y  Q  L  K  G  Q 1119
Cfau_S 1100  P  I  K  I  I  N  L  I  Q  K  Q  A  K  K  Y  Q  L  K  G  Q 1119
Cnau_S 1100  P  I  K  I  I  N  L  I  Q  K  Q  A  K  K  Y  Q  L  K  G  Q 1119
Pste_S 1100  P  I  K  I  I  N  L  I  Q  K  Q  A  K  K  Y  Q  L  K  G  Q 1119
Rma    1096  P  I  K  I  I  N  L  I  Q  K  Q  T  K  K  Y  Q  L  K  D  Q 1115
Ifos_S 1095  P  I  K  I  I  N  L  I  Q  K  Q  A  K  K  Y  Q  L  K  G  Q 1114
Apha_S 1100  P  I  R  I  I  N  L  I  Q  K  Q  A  K  K  Y  Q  L  K  G  Q 1119
Bsep_S 1097  P  I  T  I  I  N  L  I  Q  T  Q  P  R  T  Y  R  I  K  G  Q 1116
```

transcription-repair-coupling factor (TRCF) (1004-1104)

```
Akaw_S 3349 AATCAATTAATTATCAAAGAACAAATGCCCAAAAATATTAAACGAATTAAATTAATAGAA 3408
Clau_S 3352 AATCAATTAATTATCAAAGAACAAATGCCAAAAAATATTAAACGAATCAAATTAATAGAA 3411
Pkil_S 3352 AATCAATTAATTATCAAAGAACAAATGCCTAAAAATATTAGACGAATCAAATTAATAGAA 3411
Psoy_S 3352 AATCAATTAATTATCAAAGAACAAATGCCTAAAAATATTAGACGAATCAAATTAATAGAA 3411
Vok    3352 AATCAATTAATTATCAAAGAACAAATGCCTAAAAATATTAGACGAATCAAATTAATAGAA 3411
Cpac_S 3358 AACCAATTAATCGTTAAAGAGGAGATGCCTGAAGATATTAGACGAATCGAGTTGATAGAA 3417
Cfau_S 3358 AACCAATTAATCATTAAAGAAGAGATGCCTGAAGATATTAGACGAATCGAGTTGATAGAA 3417
Cnau_S 3358 AACCAATTAATCGTTAAAGAAGAGATGCCTGAAGATATTAGACGAATCGAGTTGATAGAA 3417
Pste_S 3358 AACCAATTAATCGTTAAAGAAGAGATGCCTGAAGATATTAGACGAATCGAGTTGATAGAA 3417
Rma    3346 AACCAATTAATTATTAAAGAAGAGATGCCTGAAGATATTAGACGAATCGAGTTGATAGAA 3405
Ifos_S 3343 AATCAATTAATTGTTAAACAGGAGATGCCTGAAGATATTAGACGAATTGAATTGATAGAA 3402
Apha_S 3358 AATCAATTAATTGTTAAACAAGAGATGCCTGAAGATATTAGACGAATTGAATTGATAGAA 3417
Bsep_S 3349 AATCAACTCATTATCACCCGCGAAATGCCCGAAGATATCGGGCGCATTGAATGGGTTGAA 3408
Akaw_S 1117   N  Q  L  I  I  K  E  Q  M  P  K  N  I  K  R  I  K  L  I  E 1136
Clau_S 1118   N  Q  L  I  I  K  E  Q  M  P  K  N  I  K  R  I  K  L  I  E 1137
Pkil_S 1118   N  Q  L  I  I  K  E  Q  M  P  K  N  I  R  R  I  K  L  I  E 1137
Psoy_S 1118   N  Q  L  I  I  K  E  Q  M  P  K  N  I  R  R  I  K  L  I  E 1137
Vok    1118   N  Q  L  I  I  K  E  Q  M  P  K  N  I  R  R  I  K  L  I  E 1137
Cpac_S 1120   N  Q  L  I  V  K  E  E  M  P  E  D  I  R  R  I  E  L  I  E 1139
Cfau_S 1120   N  Q  L  I  I  K  E  E  M  P  E  D  I  R  R  I  E  L  I  E 1139
Cnau_S 1120   N  Q  L  I  V  K  E  E  M  P  E  D  I  R  R  I  E  L  I  E 1139
Pste_S 1120   N  Q  L  I  V  K  E  E  M  P  E  D  I  R  R  I  E  L  I  E 1139
Rma    1116   N  Q  L  I  I  K  E  E  M  P  E  D  I  R  R  I  E  L  I  E 1135
Ifos_S 1115   N  Q  L  I  V  K  Q  E  M  P  E  D  I  R  R  I  E  L  I  E 1134
Apha_S 1120   N  Q  L  I  V  K  Q  E  M  P  E  D  I  R  R  I  E  L  I  E 1139
Bsep_S 1117   N  Q  L  I  I  T  R  E  M  P  E  D  I  G  R  I  E  W  V  E 1136
```

```
Akaw_S 3409 TATCTATTAAAAATATTA---AATTAA 3432
Clau_S 3412 TATCTATTAAAAATGTTA---AATTAA 3435
Pkil_S 3412 TACCTATTAAAAATGTTA---AATTAA 3435
Psoy_S 3412 TACCTATTAAAAATGTTA---AATTAA 3435
Vok    3412 TACCTATTAAAAATGTTA---AATTAA 3435
Cpac_S 3418 AACCTACTAAAAACATTA---AGTTAA 3441
Cfau_S 3418 AACCTACTAAAAACATTA---AGTTAA 3441
Cnau_S 3418 AACCTACTAAAAACATTA---AGTTAA 3441
Pste_S 3418 AACCTGCTAAAAACATTA---AGTTAA 3441
Rma    3406 AACCTATTAAAAACACTA---AGTTAA 3429
Ifos_S 3403 AATCTACTAAAAACATTA---AATTAG 3426
Apha_S 3418 AACCTACTAAAAACATTA---AATTAA 3441
Bsep_S 3409 GATTTTCTAAAAACTTTAGGAGCTTAA 3435
Akaw_S 1137   Y  L  L  K  I  L  #  N  * 1144
Clau_S 1138   Y  L  L  K  M  L  #  N  * 1145
Pkil_S 1138   Y  L  L  K  M  L  #  N  * 1145
Psoy_S 1138   Y  L  L  K  M  L  #  N  * 1145
Vok    1138   Y  L  L  K  M  L  #  N  * 1145
Cpac_S 1140   N  L  L  K  T  L  #  S  * 1147
Cfau_S 1140   N  L  L  K  T  L  #  S  * 1147
Cnau_S 1140   N  L  L  K  T  L  #  S  * 1147
Pste_S 1140   N  L  L  K  T  L  #  S  * 1147
Rma    1136   N  L  L  K  T  L  #  S  * 1143
Ifos_S 1135   N  L  L  K  T  L  #  N  * 1142
Apha_S 1140   N  L  L  K  T  L  #  N  * 1147
Bsep_S 1137   D  F  L  K  T  L  G  A  * 1145
```

Reference.

4. Selby CP, Sancar A. Molecular mechanism of transcription-repair coupling. Science. 1993;260(5104):53-8. PubMed PMID: 8465200.
