# Supplementary material for: Loss of genes related to Nucleotide Excision Repair (NER) and implications for reductive genome evolution in symbionts of deep-sea vesicomyid clams
Source: PLoS One. 2017 Feb 15;12(2):e0171274. doi: 10.1371/journal.pone.0171274 (PMC5310779; doi:10.1371/journal.pone.0171274)
Supplement: S4 Fig — The arrangement of nucleotide and amino acid sequences is as described in S1 Fig. Although the ORFs of this gene were collapsed in clade I symbionts and do not code proteins, the remnant amino acid sequences for coding nucleotide sequences were estimated as was possible and used for the alignment. Conserved domains of uvrC found in an NCBI blast search are shown as bidirectional arrows. Specific conserved sequences found in uvrC from E. coli are shown below the alignment [5, 6]. Bold-face, underlined letters indicate the conserved amino acid residues. The conserved hydrophobic amino acid residues in the HhH (helix-hairpin-helix) domain are shown with a red background. The conserved glycine residues in the HhH domain have a yellow background. # indicates the gap of amino acid sequence where no corresponding nucleotide sequence exist. * indicates stop codon. (PDF) [file pone.0171274.s007.pdf]

S4 Fig.

```
Akaw_S     1 ATGTCAATTAAA---GAAAAGA--TTAAAGAATTTAACTACCAACTATAGCGTTTAT---   52
Clau_S     1 ATGTCAATTAAA---GAAAAAA--TTAAAGAATCTAACTAACAACTCTGACGTTTATA--   53
Pkil_S     1 ATACCAATTAAAA--GAAAAGA-CTAAAGAATCTAACTACCAACTTTGGTGTTTATT---   54
Psoy_S     1 ATACCAATTAAAA--GAAAAGA-CTAAAGAATCTAACTACCAACTTTGGTGTTTATT---   54
Vok        1 ATACCAATTAAA---GAAAAGA--CTAAAGAATCTAACTACCAACTCTGGTGTTTAT---   52
Cpac_S     1 ATGCCAATTAAA---GAAAAG---CTAAAGAATTTAACCACTAATCCTGGCGTTTAT---   51
Cfau_S     1 ATGCCAATTAAA---GAAAAG---CTAAAGAATTTAACCACCAATCCTGGCGTTTAT---   51
Cnau_S     1 ATGCCAATTAAA---GAAAAG---CTAAAGAATTTAACCACCAATTCTGGCGTTTAT---   51
Pste_S     1 ATGCCAATTAAA---GAAAAG---CTAAAGAATTTGACCACCAATCCTGGCGTTTAT---   51
Rma        1 ATGCTAATTCAA---GAAAAG---CTAAAGAATTTAACTACTAATCCTGGTGTTTAT---   51
Ifos_S     1 ATGCTAATTAAA---GAAAAG---CTAAAGAATTTAACCACCCATCCTGGCGTTTAT---   51
Apha_S     1 ATGCCAATTAAA---GAAAAG---CTACAATTTTTAACCACCAATCCTGGTGTTTAT---   51
Bsep_S     1 ATGTCAATTAAA---GAAAAA---TTAAAAAATTTAACCACGCAACCTGGTGTGTAT---   51
Akaw_S     1   M  S  I  K  #  E  K  #  L  K  N  L  T  T  N  Y  S  V  Y  #   17
Clau_S     1   M  S  I  K  #  E  K  #  L  K  N  L  T  N  N  S  D  V  Y  #   17
Pkil_S     1   I  P  I  K  #  E  K  #  L  K  N  L  T  T  N  F  G  V  Y  #   17
Psoy_S     1   I  P  I  K  #  E  K  #  L  K  N  L  T  T  N  F  G  V  Y  #   17
Vok        1   I  P  I  K  #  E  K  #  L  K  N  L  T  T  N  S  G  V  Y  #   17
Cpac_S     1   M  P  I  K  #  E  K  #  L  K  N  L  T  T  N  P  G  V  Y  #   17
Cfau_S     1   M  P  I  K  #  E  K  #  L  K  N  L  T  T  N  P  G  V  Y  #   17
Cnau_S     1   M  P  I  K  #  E  K  #  L  K  N  L  T  T  N  S  G  V  Y  #   17
Pste_S     1   M  P  I  K  #  E  K  #  L  K  N  L  T  T  N  P  G  V  Y  #   17
Rma        1   M  L  I  Q  #  E  K  #  L  K  N  L  T  T  N  P  G  V  Y  #   17
Ifos_S     1   M  L  I  K  #  E  K  #  L  K  N  L  T  T  H  P  G  V  Y  #   17
Apha_S     1   M  P  I  K  #  E  K  #  L  Q  F  L  T  T  N  P  G  V  Y  #   17
Bsep_S     1   M  S  I  K  #  E  K  #  L  K  N  L  T  T  Q  P  G  V  Y  #   17

E. coli                                            T  S  Q  P  G  V  Y
```

GIY-YIG_UvrC_Cho domain (10-90)

```
Akaw_S    53 CAAATG-----------------------------------------------------   58
Clau_S    54 -AAATATTTGATAAACATGGTCAAATAACCTATGTAGGTAAAGCTAACAATTAAAAAGA  112
Pkil_S    55 -AAATATTTGATAAACAGGATTAAATAATTTATGTAGATAAAGCTAAAAACTTAAAAAGT  113
Psoy_S    55 -AAATATTTGATAAACAGGATTAAATAATTTATGTAGATAAAGCTAAAAACTTAAAAAGT  113
Vok       53 CAAATGTTTGATAAACAGGATTAAATAATCTATGTAGGTAAAGCTAAAAACTTAAAAAGT  112
Cpac_S    52 CAAATGTTTAATAAGCAAGATCAAGTGATTTATGTAGGTAAGGCTAGAAACTTAAAAAAT  111
Cfau_S    52 CAAATGTTTGATAAGCAAGACCAAGTGATTTATGTGGGCAAGGCTAAAAACCTAAAAAAT  111
Cnau_S    52 CAAATGCTTGATAAGCAAGATCAAGTGATTTATGTGGGTAAGGCTAAAAACCTAAAAAAT  111
Pste_S    52 CAAATGTTTGATAGGCAAGACCAAGTGATTTATGTGGGTAAGGCTAAAAACCTAAAAAAT  111
Rma       52 CAAATGTTTGATAAACAAGATCAAGTGATTTATGTCGGTAAGGCTAAAAATCTAAAAAAT  111
Ifos_S    52 CAAATGTTTGATAAACAAGACCGGGTGATTTATGTGGGCAAGGCTAAAAATCTAAAAAAT  111
Apha_S    52 CAAATGTTTGATAAGCAAGACCAAGTGATTTATGTAGGTAAGGCTAAAAACCTAAAAAAT  111
Bsep_S    52 CAAATGCTAGACAAGCAGGGTCTAGTGATTTATGTTGGTAAGGCAAAAAACCTAAAAAAA  111
Akaw_S    18   Q  M  #  #  #  #  #  #  #  #  #  #  #  #  #  #  #  #  #  #   19
Clau_S    18   #  I  F  D  K  H  G  Q  I  T  Y  V  G  K  A  N  K  L  K  R   36
Pkil_S    18   #  I  F  D  K  Q  D  *  I  I  Y  V  D  K  A  K  N  L  K  S   36
Psoy_S    18   #  I  F  D  K  Q  D  *  I  I  Y  V  D  K  A  K  N  L  K  S   36
Vok       18   Q  M  F  D  K  Q  D  *  I  I  Y  V  G  K  A  K  N  L  K  S   37
Cpac_S    18   Q  M  F  N  K  Q  D  Q  V  I  Y  V  G  K  A  R  N  L  K  N   37
Cfau_S    18   Q  M  F  D  K  Q  D  Q  V  I  Y  V  G  K  A  K  N  L  K  N   37
Cnau_S    18   Q  M  L  D  K  Q  D  Q  V  I  Y  V  G  K  A  K  N  L  K  N   37
Pste_S    18   Q  M  F  D  R  Q  D  Q  V  I  Y  V  G  K  A  K  N  L  K  N   37
Rma       18   Q  M  F  D  K  Q  D  Q  V  I  Y  V  G  K  A  K  N  L  K  N   37
Ifos_S    18   Q  M  F  D  K  Q  D  R  V  I  Y  V  G  K  A  K  N  L  K  N   37
Apha_S    18   Q  M  F  D  K  Q  D  Q  V  I  Y  V  G  K  A  K  N  L  K  N   37
Bsep_S    18   Q  M  L  D  K  Q  G  L  V  I  Y  V  G  K  A  K  N  L  K  K   37

E. coli        R  M  Y  D  A  G  G  T  V  I  Y  V  G  K  A  K  D  L  K  K
```

GIY-YIG_UvrC_Cho domain (10-90)

```
Akaw_S    59 ------------------------------------------------------------  58
Clau_S   113 AAAGTGTAAAACTATTTTCTTAGAACACATAAACAGAACAAAGCTAGAGTTCTAGTGGTA  172
Pkil_S   114 AGAGTGTTAAACTATTTTGTTAGAACACATAAATAGAATAAAACTAGAGTTCTC-TGGTG  172
Psoy_S   114 AGAGTGTTAAACTATTTTGTTAGAACACATAAATAGAATAAAACTAGAGTTCTC-TGGTG  172
Vok      113 AGAGTGTTAAACTATTTTGTTAGAACACATAAACAGAACAAAACTAGAGTCC---TGGTG  169
Cpac_S   112 AGAGTATCAAGCTATTTTGTTAAAACACATGAGCAAAAGAAGGCTAGAGTTTTAGTTGCA  171
Cfau_S   112 AGAGTATCAAGCTATTTTGTTAAAACACATGAGCAGAATAAGACTAGAGTTTTAGTTGCA  171
Cnau_S   112 AGAGTATCAAGCTATTTTGTTAAAACACATGAGCAGAATAAGACTAGAGTTTTAGTTGCA  171
Pste_S   112 AGAGTATCAAGCTATTTTGTTAAAACATATGAGCAGAACAAGACTAGAGTTTTAGTTGCA  171
Rma      112 AGAGTATCAAGCTACTTTGTTAAAACACATGAGCAGAACAAGGCCAGGGTTTTAGTTGCA  171
Ifos_S   112 AGAGTGTCAAGCTACTTTGTTAAAACACATGAGCAGAATAAGACCAGAGTTTTAGTTGCC  171
Apha_S   112 AGAGTATCAAGCTACTTTGTGAAAACACATGAGCAGAATAAGACTAGGGTTTTAGTTGCA  171
Bsep_S   112 CGCGTGTCTAATTATTTTTTTAAAAATCATCAAGACGATAAAACTAGGGTATTAGTGAGT  171
Akaw_S    20  #  #  #  #  #  #  #  #  #  #  #  #  #  #  #  #  #  #  #  #   19
Clau_S    37  K  V  *  N  Y  F  L  R  T  H  K  Q  N  K  A  R  V  L  V  V   56
Pkil_S    37  R  V  L  N  Y  F  V  R  T  H  K  *  N  K  T  R  V  L  #  V   55
Psoy_S    37  R  V  L  N  Y  F  V  R  T  H  K  *  N  K  T  R  V  L  #  V   55
Vok       38  R  V  L  N  Y  F  V  R  T  H  K  Q  N  K  T  R  V  #  #  V   55
Cpac_S    38  R  V  S  S  Y  F  V  K  T  H  E  Q  K  K  A  R  V  L  V  A   57
Cfau_S    38  R  V  S  S  Y  F  V  K  T  H  E  Q  N  K  T  R  V  L  V  A   57
Cnau_S    38  R  V  S  S  Y  F  V  K  T  H  E  Q  N  K  T  R  V  L  V  A   57
Pste_S    38  R  V  S  S  Y  F  V  K  T  Y  E  Q  N  K  T  R  V  L  V  A   57
Rma       38  R  V  S  S  Y  F  V  K  T  H  E  Q  N  K  A  R  V  L  V  A   57
Ifos_S    38  R  V  S  S  Y  F  V  K  T  H  E  Q  N  K  T  R  V  L  V  A   57
Apha_S    38  R  V  S  S  Y  F  V  K  T  H  E  Q  N  K  T  R  V  L  V  A   57
Bsep_S    38  R  V  S  N  Y  F  F  K  N  H  Q  D  D  K  T  R  V  L  V  S   57

E. coli       R  L  S  S  Y  F  R  S  N  L  A  S  R  K  T  E  A  L  V  A
```

GIY-YIG_UvrC_Cho domain (10-90)

```
Akaw_S    59 ------------------------------------------------------------  58
Clau_S   173 ---AATATTAAAATTTTTAAAGTT-------------------------------------  193
Pkil_S   173 GTAAATATTAAAATTTTTAAAGTT-------------------------------------  196
Psoy_S   173 GTAAATATTAAAATTTTTAAAGTT-------------------------------------  196
Vok      170 GTAAATATTAAAATTTTTAAAGTT-------------------------------------  193
Cpac_S   172 ---AATACTAAAGACTTTAAAGTAATCGTTACTGCAACGGAAACTCAGGCTTTGTTATTA  228
Cfau_S   172 ---AATACTAAAGACTTTAAAGTAATCGTTACTGCAACGGAAACTCAGGCTTTGTTATTA  228
Cnau_S   172 ---AATATTAAAGACTTTAAAGTAATCGTTACTGCAACGGAAACTCAGGCTTTGTTATTA  228
Pste_S   172 ---AATATTAAAGACTTTAAAGTAATCGTTACTGCAACGGAAACTCAGGCTTTGTTATTA  228
Rma      172 ---AATATTAAAGATTTTAAAGTAATCGTTACTGCAACGGAAACTCAGGCTTTATTATTA  228
Ifos_S   172 ---AACATTAAAGATTTTAGAGTAATCGTTACTGCAACGGAAACTCAGGCTTTGTTATTA  228
Apha_S   172 ---AATATTAAGGACTTTAAAGTAATCGTTACTGCAACAGAAACTCAGGCTTTGTTGTTA  228
Bsep_S   172 ---AATATTGACACTTTTGAAACTGTGATTACCGACACTGAAACTCAAGCGTTATTACTA  228
Akaw_S    20  #  #  #  #  #  #  #  #  #  #  #  #  #  #  #  #  #  #  #  #   19
Clau_S    57  #  N  I  K  I  F  K  V  #  #  #  #  #  #  #  #  #  #  #  #   63
Pkil_S    56  V  N  I  K  I  F  K  V  #  #  #  #  #  #  #  #  #  #  #  #   63
Psoy_S    56  V  N  I  K  I  F  K  V  #  #  #  #  #  #  #  #  #  #  #  #   63
Vok       56  V  N  I  K  I  F  K  V  #  #  #  #  #  #  #  #  #  #  #  #   63
Cpac_S    58  #  N  T  K  D  F  K  V  I  V  T  A  T  E  T  Q  A  L  L  L   76
Cfau_S    58  #  N  T  K  D  F  K  V  I  V  T  A  T  E  T  Q  A  L  L  L   76
Cnau_S    58  #  N  I  K  D  F  K  V  I  V  T  A  T  E  T  Q  A  L  L  L   76
Pste_S    58  #  N  I  K  D  F  K  V  I  V  T  A  T  E  T  Q  A  L  L  L   76
Rma       58  #  N  I  K  D  F  K  V  I  V  T  A  T  E  T  Q  A  L  L  L   76
Ifos_S    58  #  N  I  K  D  F  R  V  I  V  T  A  T  E  T  Q  A  L  L  L   76
Apha_S    58  #  N  I  K  D  F  K  V  I  V  T  A  T  E  T  Q  A  L  L  L   76
Bsep_S    58  #  N  I  D  T  F  E  T  V  I  T  D  T  E  T  Q  A  L  L  L   76

E. coli       L  V  A  Q  I  Q  Q  I  D  V  T  V  T  E  T  E  A  L  L  L
```

GIY-YIG_UvrC_Cho domain (10-90)

```
Akaw_S   59 ------------------------------------------------------------  58
Clau_S  194 ------------------------------------------------------------ 193
Pkil_S  197 ------------------------------------------------------------ 196
Psoy_S  197 ------------------------------------------------------------ 196
Vok     194 ------------------------------------------------------------ 193
Cpac_S  229 GAATCTGAACTAATTAAGCAGCACATGCCAAGATATAACATCTTGTTAAAAGATTCAAAA 288
Cfau_S  229 GAATCTGAACTAATTAAGCAGCACATGCCAAGATATAACATCTTGTTAAAAGATTCAAAA 288
Cnau_S  229 GAATCTGAACTAATTAAGCAGCACATGCCAAGATATAACATTTTGTTAAAAGATTCAAAA 288
Pste_S  229 GAATCTGAACTAATTAAGCAGCACATGCCAAGATATAACATCTTGTTAAAAGATTCAAAA 288
Rma     229 GAATCTGAACTAATTAAGCAACATATGCCAAGATATAATATTTTATTAAAAGATTCAAAA 288
Ifos_S  229 GAATCTGAACTGATTAAGCAACACATGCCAAGATATAACATCTTGTTAAAGGATTCAAAA 288
Apha_S  229 GAGTCTGAACTAATTAAGCAGTACATGCCAAGATATAACATCTTGTTGAAAGATTCAAAA 288
Bsep_S  229 GAAAACGAATTGATTAAACAGCACAAACCCAGATATAACATTTTGTTGAAAGACGCAAAA 288
Akaw_S   20   #  #  #  #  #  #  #  #  #  #  #  #  #  #  #  #  #  #  #  #   19
Clau_S   64   #  #  #  #  #  #  #  #  #  #  #  #  #  #  #  #  #  #  #  #   63
Pkil_S   64   #  #  #  #  #  #  #  #  #  #  #  #  #  #  #  #  #  #  #  #   63
Psoy_S   64   #  #  #  #  #  #  #  #  #  #  #  #  #  #  #  #  #  #  #  #   63
Vok      64   #  #  #  #  #  #  #  #  #  #  #  #  #  #  #  #  #  #  #  #   63
Cpac_S   77   E  S  E  L  I  K  Q  H  M  P  R  Y  N  I  L  L  K  D  S  K   96
Cfau_S   77   E  S  E  L  I  K  Q  H  M  P  R  Y  N  I  L  L  K  D  S  K   96
Cnau_S   77   E  S  E  L  I  K  Q  H  M  P  R  Y  N  I  L  L  K  D  S  K   96
Pste_S   77   E  S  E  L  I  K  Q  H  M  P  R  Y  N  I  L  L  K  D  S  K   96
Rma      77   E  S  E  L  I  K  Q  H  M  P  R  Y  N  I  L  L  K  D  S  K   96
Ifos_S   77   E  S  E  L  I  K  Q  H  M  P  R  Y  N  I  L  L  K  D  S  K   96
Apha_S   77   E  S  E  L  I  K  Q  Y  M  P  R  Y  N  I  L  L  K  D  S  K   96
Bsep_S   77   E  N  E  L  I  K  Q  H  K  P  R  Y  N  I  L  L  K  D  A  K   96

E. coli       E  H  N  Y  I  K  L  Y  Q  P  R  Y  N  V  L
```

GIY-YIG_UvrC_Cho domain (10-90)

```
Akaw_S   59 ------------------------------------------------------------  58
Clau_S  194 ---------------------------------------AGAG----------------- 197
Pkil_S  197 ------------------------------------------------------------ 196
Psoy_S  197 ------------------------------------------------------------ 196
Vok     194 ------------------------------------------------------------ 193
Cpac_S  289 AGTTACCCCTATATCTTTATCAGTCACGACAAACATCCTAGAGTGGATTTTTATCGAGGC 348
Cfau_S  289 AGTTATCCCTATATCTTTATCAGTCATGACAAACATCCTAGAGTGGATTTTTATCGAGGC 348
Cnau_S  289 AGTTATCCCTATATCTTTATTAGTCATGACAAGCATCCTAAAGTGGGTTTTTATCGAGGT 348
Pste_S  289 AGTTATCCCTATATCTTTATCAGTCATGACAAACATCCTAGAGTGGGGTTTTATCGAGGC 348
Rma     289 AGTTATCCCTATATCTTTATCAGCCATGACAAACATCCTAGAGTGGGTTTTTATCGAGGA 348
Ifos_S  289 AGTTATCCTTATATCTTTATTAGTCATGACAAGCATCCTAGAGTGGGTTTTTATCGAGGT 348
Apha_S  289 AGCTATCCTTATATTTTTATCAGCCATGACAAGCATCCTAGAGTGGGTTTTTATCGAGGT 348
Bsep_S  289 AGTTACCCCTACATTTATATCAGCAATGACAAACATCCGCGCGTGGGCTTTTATCGCGGC 348
Akaw_S   20   #  #  #  #  #  #  #  #  #  #  #  #  #  #  #  #  #  #  #  #   19
Clau_S   64   #  #  #  #  #  #  #  #  #  #  #  #  #  R  #  #  #  #  #  #   64
Pkil_S   64   #  #  #  #  #  #  #  #  #  #  #  #  #  #  #  #  #  #  #  #   63
Psoy_S   64   #  #  #  #  #  #  #  #  #  #  #  #  #  #  #  #  #  #  #  #   63
Vok      64   #  #  #  #  #  #  #  #  #  #  #  #  #  #  #  #  #  #  #  #   63
Cpac_S   97   S  Y  P  Y  I  F  I  S  H  D  K  H  P  R  V  D  F  Y  R  G  116
Cfau_S   97   S  Y  P  Y  I  F  I  S  H  D  K  H  P  R  V  D  F  Y  R  G  116
Cnau_S   97   S  Y  P  Y  I  F  I  S  H  D  K  H  P  K  V  G  F  Y  R  G  116
Pste_S   97   S  Y  P  Y  I  F  I  S  H  D  K  H  P  R  V  G  F  Y  R  G  116
Rma      97   S  Y  P  Y  I  F  I  S  H  D  K  H  P  R  V  G  F  Y  R  G  116
Ifos_S   97   S  Y  P  Y  I  F  I  S  H  D  K  H  P  R  V  G  F  Y  R  G  116
Apha_S   97   S  Y  P  Y  I  F  I  S  H  D  K  H  P  R  V  G  F  Y  R  G  116
Bsep_S   97   S  Y  P  Y  I  Y  I  S  N  D  K  H  P  R  V  G  F  Y  R  G  116
```

```
Akaw_S   59 ------------------------------------------------------------  58
Clau_S  198 ------------AATG-T------------------------------------------ 202
Pkil_S  197 ------------------------------------------------------------ 196
Psoy_S  197 ------------------------------------------------------------ 196
Vok     194 ------------------------------------------------------------ 193
Cpac_S  349 AGAAGGCATCAGAATGCTCAATATTTTGGCCCCTACCCTTCGGCTCATGTGGTCAGAGAT 408
Cfau_S  349 AAAAGGCATCAGAATGCTCAATATTTTGGCCCCTACCCTTCAGCTCATATGGTCAGAGAT 408
Cnau_S  349 AAAAAGTATCAGAATGCTCAATATTTTGGCCCCTACCATTCGGCTCATGTGGTCAGAGAT 408
Pste_S  349 AAAAAGCATCAGAATGCTCAATATTTTGGCCCCTACCCTTCGGCTCATGTGGTCAGAGAT 408
Rma     349 AAAAAGTGTCAGAGTGCTCAATATTTCGGTCCCTACCCTTCGGCTCATGTGGTTAGAGAT 408
Ifos_S  349 AAAAAACATCAGAGTGCTCAATATTTCGGTCCCTATCCTTCGGCTCATGTGGTTAGGGAT 408
Apha_S  349 AAAAAGCATCAGAGTGCTCAATATTTTGGCCCCTACCCTTCGGCTCATGTGGTTAGGGAT 408
Bsep_S  349 ACCAAAAATAATAAATACCAATATTTTGGGCCGTATCCATCTGCACATATTGTGCGTGAT 408
Akaw_S   20  #  #  #  #  #  #  #  #  #  #  #  #  #  #  #  #  #  #  #  #   19
Clau_S   65  #  #  #  #  N  #  #  #  #  #  #  #  #  #  #  #  #  #  #  #   65
Pkil_S   64  #  #  #  #  #  #  #  #  #  #  #  #  #  #  #  #  #  #  #  #   63
Psoy_S   64  #  #  #  #  #  #  #  #  #  #  #  #  #  #  #  #  #  #  #  #   63
Vok      64  #  #  #  #  #  #  #  #  #  #  #  #  #  #  #  #  #  #  #  #   63
Cpac_S  117  R  R  H  Q  N  A  Q  Y  F  G  P  Y  P  S  A  H  V  V  R  D  136
Cfau_S  117  K  R  H  Q  N  A  Q  Y  F  G  P  Y  P  S  A  H  M  V  R  D  136
Cnau_S  117  K  K  Y  Q  N  A  Q  Y  F  G  P  Y  H  S  A  H  V  V  R  D  136
Pste_S  117  K  K  H  Q  N  A  Q  Y  F  G  P  Y  P  S  A  H  V  V  R  D  136
Rma     117  K  K  C  Q  S  A  Q  Y  F  G  P  Y  P  S  A  H  V  V  R  D  136
Ifos_S  117  K  K  H  Q  S  A  Q  Y  F  G  P  Y  P  S  A  H  V  V  R  D  136
Apha_S  117  K  K  H  Q  S  A  Q  Y  F  G  P  Y  P  S  A  H  V  V  R  D  136
Bsep_S  117  T  K  N  N  K  Y  Q  Y  F  G  P  Y  P  S  A  H  I  V  R  D  136
```

```
Akaw_S   59 ------------------------------------------------------------  58
Clau_S  203 ---------------------------------------TGT---AATATAACTTATTGA 220
Pkil_S  197 ---------------------------------AAAGAGTGTCCTAATAGAACTTACTGA 223
Psoy_S  197 ---------------------------------AAAGAGTGTCCTAATAGAACTTACTGA 223
Vok     194 ---------------------------------AGAGAATGCTCTAATAGAACTTACTGA 220
Cpac_S  409 TCGCTTAATTTACTTAAAAAAATCTTTAAAGTTAGGCAGTGTACTAATACTACTTACCGA 468
Cfau_S  409 TCGCTTAATTTACTTAAAAAAATCTTTAAAGTTAGACAATGTACTAATAGTACTTACCAA 468
Cnau_S  409 TCGCTTAATTTACTTAAAAAAATCTTTAAAGTTAGGCAATGTACTAATAGTACTTACCGA 468
Pste_S  409 TCGCTTAATTTACTTAAAAAAATTTTTAAAGTTAGGCAATGTACTAATAGTACTTACCGA 468
Rma     409 TCGCTCAGTCTACTTAAAAAAATCTTTAAAGTTAGACAATGTACCAATAGCACTTATCGC 468
Ifos_S  409 TCACTCAATTTACTTAAAAAAATCTTTAAAGTTAGGCAATGCAGTAATAGCACCTACCGA 468
Apha_S  409 TCGCTTAATTTACTTAAAAAAATCTTTAAAGTTAGACAATGTACTAATAGTACTTACCGA 468
Bsep_S  409 TCTCTCAATTTACTAAAGAAAGTTTTTAAAGTTAGGCAATGTGCAAATGCTACCTATCGC 468
Akaw_S   20  #  #  #  #  #  #  #  #  #  #  #  #  #  #  #  #  #  #  #  #   19
Clau_S   66  #  #  #  #  #  #  #  #  #  #  #  #  #  C  #  N  I  T  Y  *   71
Pkil_S   64  #  #  #  #  #  #  #  #  #  #  #  K  E  C  P  N  R  T  Y  *   72
Psoy_S   64  #  #  #  #  #  #  #  #  #  #  #  K  E  C  P  N  R  T  Y  *   72
Vok      64  #  #  #  #  #  #  #  #  #  #  #  R  E  C  S  N  R  T  Y  *   72
Cpac_S  137  S  L  N  L  L  K  K  I  F  K  V  R  Q  C  T  N  T  T  Y  R  156
Cfau_S  137  S  L  N  L  L  K  K  I  F  K  V  R  Q  C  T  N  S  T  Y  Q  156
Cnau_S  137  S  L  N  L  L  K  K  I  F  K  V  R  Q  C  T  N  S  T  Y  R  156
Pste_S  137  S  L  N  L  L  K  K  I  F  K  V  R  Q  C  T  N  S  T  Y  R  156
Rma     137  S  L  S  L  L  K  K  I  F  K  V  R  Q  C  T  N  S  T  Y  R  156
Ifos_S  137  S  L  N  L  L  K  K  I  F  K  V  R  Q  C  S  N  S  T  Y  R  156
Apha_S  137  S  L  N  L  L  K  K  I  F  K  V  R  Q  C  T  N  S  T  Y  R  156
Bsep_S  137  S  L  N  L  L  K  K  V  F  K  V  R  Q  C  A  N  A  T  Y  R  156
```

```
Akaw_S   59 -----------------------------------------------------------  58
Clau_S  221 -AA---TCTCGTTTAGATC--TTATTTA--GAATATCAAATATGGTTATGTAGTGCACCT  272
Pkil_S  224 --A---TCTCGTTTAGACC--TTATTTA--TAATATCAAATGTGGCTATGTAGTGCACCT  274
Psoy_S  224 --A---TCTCGTTTAGACC--TTATTTA--TAATATCAAATGTGGCTATGTAGTGCACCT  274
Vok     221 --A---TCTCGTTTAGAC--TTTGTTTA--TAATATCAAATATGGCTATGTAGTACACCT  271
Cpac_S  469 TCACGCTCCAGACCTTGT---TTA------GAATATCAAATAGGGCTGTGTAGCGCACCT  519
Cfau_S  469 TCACGCTCCAAGCCTTGT---TTA------GAATATCAAATAGGGCTGTGTAGTGCACCT  519
Cnau_S  469 TCACGCTCCAGCCCTTGT---TTA------GAATATCAAATAGGGCTGTGTAGTGCACCT  519
Pste_S  469 TCACGCTCCAGACCTTGT---TTA------GAATATCAAATAGGGCTGTGTAGTGCACCT  519
Rma     469 TCACGTTCCAGACCTTGT---TTA------GAATATCAAATAGGGCTATGTAGTGCACCT  519
Ifos_S  469 TCACGCTCCAGACCTTGT---TTA------GAATATCAAATAGGACTATGTAGTGCACCT  519
Apha_S  469 TCACGCTCCAGACCTTGT---TTA------GAATATCAAATAGGGCTATGTAGCGCACCT  519
Bsep_S  469 TCAAGGTCTAGGCCTTGC---TTG------GAATATCAAATTGGCTTGTGTAGTGCGCCC  519
Akaw_S   20   #  #  #  #  #  #  #  #  #  #  #  #  #  #  #  #  #  #  #  #    19
Clau_S   72   #  #  S  R  L  D  #  L  F  #  E  Y  Q  I  W  L  C  S  A  P    87
Pkil_S   73   #  #  S  R  L  D  #  L  F  #  *  Y  Q  M  W  L  C  S  A  P    88
Psoy_S   73   #  #  S  R  L  D  #  L  F  #  *  Y  Q  M  W  L  C  S  A  P    88
Vok      73   #  #  S  R  L  D  #  L  F  #  *  Y  Q  I  W  L  C  S  T  P    88
Cpac_S  157   S  R  S  R  P  C  #  L  #  #  E  Y  Q  I  G  L  C  S  A  P   173
Cfau_S  157   S  R  S  K  P  C  #  L  #  #  E  Y  Q  I  G  L  C  S  A  P   173
Cnau_S  157   S  R  S  S  P  C  #  L  #  #  E  Y  Q  I  G  L  C  S  A  P   173
Pste_S  157   S  R  S  R  P  C  #  L  #  #  E  Y  Q  I  G  L  C  S  A  P   173
Rma     157   S  R  S  R  P  C  #  L  #  #  E  Y  Q  I  G  L  C  S  A  P   173
Ifos_S  157   S  R  S  R  P  C  #  L  #  #  E  Y  Q  I  G  L  C  S  A  P   173
Apha_S  157   S  R  S  R  P  C  #  L  #  #  E  Y  Q  I  G  L  C  S  A  P   173
Bsep_S  157   S  R  S  R  P  C  #  L  #  #  E  Y  Q  I  G  L  C  S  A  P   173
```

```
Akaw_S   59 -----------------------------------------------------------  58
Clau_S  273 TGTGTGAGAAAAGTTAACCATGT-GAAA--CATTATCAATCAGATGTACCAATGATGTCT  329
Pkil_S  275 TGTGTGGGAAAAAATCAGTTATAT-GAA---AACTATCAATCAGATGTACCAATGATGTTT  330
Psoy_S  275 TGTGTGGGAAAAAATCAGTTATAT-GAA---AACTATCAATCAGATGTACCAATGATGTTT  330
Vok     272 TGTGTGGGAAAAAATCAGCCATAT-GAA---AATTATCAATTAGATGTACCAATGATGTCT  327
Cpac_S  520 TGTGTTGGCAAAATTAGCGAT---GAA---AATTATCAATCAGATGTATTAATGATGTCT  573
Cfau_S  520 TGTGTTAGCAAAATTAGCGAT---GAA---AATTATCAATCAGATGTATTAATGATGTCT  573
Cnau_S  520 TGTGTTAGCAAAATTAGCGAT---GAA---AATTATCAATCAGATGTATTAATGATGTCT  573
Pste_S  520 TGTGTTAGCAAAATTAGTGAT---GAA---AATTATCAATCAGATGTATTAATGATGTCT  573
Rma     520 TGCGTTAACAAAATTAGCGAT---GAA---AATTATCAATCAGATGTATTGATGATGTCT  573
Ifos_S  520 TGCGTTAGCAAAATTAGCGAT---GAA---AATTATCAATCAGACGTGTTGATGATGTCT  573
Apha_S  520 TGCGTTAACAAAATTAGCGAT---GAA---AATTATCAATCAGATGTGTTGATGATGTCT  573
Bsep_S  520 TGTGTTAACAAAATAAGCAAC---GAA---GATTATGTGCAAGATGTCAAAATGATGGGG  573
Akaw_S   20   #  #  #  #  #  #  #  #  E  #  #  #  #  #  #  #  #  #  #  #    20
Clau_S   88   C  V  R  K  V  N  H  #  E  #  H  Y  Q  S  D  V  P  M  M  S   105
Pkil_S   89   C  V  G  K  I  S  Y  #  E  #  N  Y  Q  S  D  V  P  M  M  F   106
Psoy_S   89   C  V  G  K  I  S  Y  #  E  #  N  Y  Q  S  D  V  P  M  M  F   106
Vok      89   C  V  G  K  I  S  H  #  E  #  N  Y  Q  L  D  V  P  M  M  S   106
Cpac_S  174   C  V  G  K  I  S  D  #  E  #  N  Y  Q  S  D  V  L  M  M  S   191
Cfau_S  174   C  V  S  K  I  S  D  #  E  #  N  Y  Q  S  D  V  L  M  M  S   191
Cnau_S  174   C  V  S  K  I  S  D  #  E  #  N  Y  Q  S  D  V  L  M  M  S   191
Pste_S  174   C  V  S  K  I  S  D  #  E  #  N  Y  Q  S  D  V  L  M  M  S   191
Rma     174   C  V  N  K  I  S  D  #  E  #  N  Y  Q  S  D  V  L  M  M  S   191
Ifos_S  174   C  V  S  K  I  S  D  #  E  #  N  Y  Q  S  D  V  L  M  M  S   191
Apha_S  174   C  V  N  K  I  S  D  #  E  #  N  Y  Q  S  D  V  L  M  M  S   191
Bsep_S  174   C  V  N  K  I  S  N  #  E  #  D  Y  V  Q  D  V  K  M  M  G   191
```

```
Akaw_S   59 ------------------------------------------------------------  58
Clau_S  330 TTGCTTTTATCTAGCAAAGACAAGAAAACCTTATAAAAGGTATCAAAAAAAA--ATGCAA 387
Pkil_S  331 TTATTCTTATCTGGCAAAGACAATAAAACTCTAGAAAAGGTGTTAAAAAAA---ATGCAA 387
Psoy_S  331 TTATTCTTATCTGGCAAAGACAATAAAACTCTAGAAAAGGTGTTAAAAAAA---ATGCAA 387
Vok     328 TTATTCTTATCTGACAAAGACAAGAAAACTCTAGAAAAGGTGTCAAAAAAA---ATGCAA 384
Cpac_S  574 TTGTTTTTATCTGGCAAAGGTAAAAAAACCCTAGAAAAGATGTCAAAGAAA---ATGCAA 630
Cfau_S  574 TTGTTTTTATCTGGCAAAGGTAGGAAAACCCTAGAAAAGGTGTCAAAGAAA---ATGCAA 630
Cnau_S  574 TTGTTTTTATCTGGCAAAGGTAGGGAGACCCTAGAAAAAGTGTCAAAAAAA---ATGCAA 630
Pste_S  574 TTGTTTTTATCTGGTAAAGGTAGGGAAACCCTAGAAAAAGTGTCAAAGAAA---ATGCAA 630
Rma     574 TTATTTTTATCTGGTAAAGGCAGAGAAACCCTAGAAAGAGTGTCAAAAAAA---ATGCAA 630
Ifos_S  574 TTGTTTTTATCTGGCAAAGGCAGAGAAGCCCTAGAGAGAGTGTCAAAGAAA---ATGCAA 630
Apha_S  574 TTGTTTTTGTCTGGCAAAGGTAGGGAAACCCTGGAAAAGGTGTCAAAGAAA---ATGCAA 630
Bsep_S  574 TTATTTTTATCAGGAAAAGGCGTGCAAATTTTAGAAGATGTGTCGCAAAAA---ATGCAA 630
Akaw_S   21   #  #  #  #  #  #  #  #  #  #  #  #  #  #  #  #  #  #  #  #   20
Clau_S  106   L  L  L  S  S  K  D  K  K  T  L  *  K  V  S  K  K  #  M  Q  124
Pkil_S  107   L  F  L  S  G  K  D  N  K  T  L  E  K  V  L  K  K  #  M  Q  125
Psoy_S  107   L  F  L  S  G  K  D  N  K  T  L  E  K  V  L  K  K  #  M  Q  125
Vok     107   L  F  L  S  D  K  D  K  K  T  L  E  K  V  S  K  K  #  M  Q  125
Cpac_S  192   L  F  L  S  G  K  G  K  K  T  L  E  K  M  S  K  K  #  M  Q  210
Cfau_S  192   L  F  L  S  G  K  G  R  K  T  L  E  K  V  S  K  K  #  M  Q  210
Cnau_S  192   L  F  L  S  G  K  G  R  E  T  L  E  K  V  S  K  K  #  M  Q  210
Pste_S  192   L  F  L  S  G  K  G  R  E  T  L  E  K  V  S  K  K  #  M  Q  210
Rma     192   L  F  L  S  G  K  G  R  E  T  L  E  R  V  S  K  K  #  M  Q  210
Ifos_S  192   L  F  L  S  G  K  G  R  E  A  L  E  R  V  S  K  K  #  M  Q  210
Apha_S  192   L  F  L  S  G  K  G  R  E  T  L  E  K  V  S  K  K  #  M  Q  210
Bsep_S  192   L  F  L  S  G  K  G  V  Q  I  L  E  D  V  S  Q  K  #  M  Q  210
```

UvrB/uvrC motif (200-234)

```
Akaw_S   59 ------------------------------------------------------------  58
Clau_S  388 CTTACTTCTCAAAATTTAGAGTTTGAATTAGCGGCGTGATTACAGGACCAATTAATTGAT 447
Pkil_S  388 CTTGCTTCT-AAAATTTAGAGTTTGAATTAGCGGCGTGATTACATTACTAATTAATTGAT 446
Psoy_S  388 CTTGCTTCT-AAAATTTAGAGTTTGAATTAGCGGCGTGATTACATTACTAATTAATTGAT 446
Vok     385 CTTGCTTCTCAAAATTTAGAGTTTGAATTAGCGGCGCGATTATATTACCAATTAATTGAT 444
Cpac_S  631 CTTGCCTCTCAAAATTTAGAGTTTGAATTGGCAGCACGCTTGCGCGATCAATTAATTGAT 690
Cfau_S  631 CTTGCCTCTCAAAATTTAGAGTTTGAATTGGCAGCACGCTTGCGTGATCAATTAATTGAT 690
Cnau_S  631 CTTGCCTCTCAAAATTTAGAGTTTGAATTGGCAGCACGCTTGCGTGATCAATTGATTGAT 690
Pste_S  631 CTTGCCTCTCAAAATTTAGAGTTTGAATTGGCAGCACGCTTGCGTGATCAATTGATTGAT 690
Rma     631 CTTGCCTCTAAAAAATCTAGAGTTTGAATTGGCAGCGCGTTTACGTGACCAATTGATTGAT 690
Ifos_S  631 CTTGCCTCTCAAAATTTAGAGTTTGAATTGGCAGCGCGTTTACGCGACCAGTTGATTGAT 690
Apha_S  631 TTTGCCTCTCAAAATTTAGAGTTTGAATTGGCAGCACGCTTGCGTGATCAATTGATTGAT 690
Bsep_S  631 GCGGCTGCTCAAAATAAAAACTACGAATTGGCAGCGCACTTGCGTGATCAAATGATTGGT 690
Akaw_S   21   #  #  #  #  #  #  #  #  #  #  #  #  #  #  #  #  #  #  #  #   20
Clau_S  125   L  T  S  Q  N  L  E  F  E  L  A  A  *  L  Q  D  Q  L  I  D  144
Pkil_S  126   L  A  S  #  N  L  E  F  E  L  A  A  *  L  H  Y  *  L  I  D  144
Psoy_S  126   L  A  S  #  N  L  E  F  E  L  A  A  *  L  H  Y  *  L  I  D  144
Vok     126   L  A  S  Q  N  L  E  F  E  L  A  A  R  L  Y  Y  Q  L  I  D  145
Cpac_S  211   L  A  S  Q  N  L  E  F  E  L  A  A  R  L  R  D  Q  L  I  D  230
Cfau_S  211   L  A  S  Q  N  L  E  F  E  L  A  A  R  L  R  D  Q  L  I  D  230
Cnau_S  211   L  A  S  Q  N  L  E  F  E  L  A  A  R  L  R  D  Q  L  I  D  230
Pste_S  211   L  A  S  Q  N  L  E  F  E  L  A  A  R  L  R  D  Q  L  I  D  230
Rma     211   L  A  S  K  N  L  E  F  E  L  A  A  R  L  R  D  Q  L  I  D  230
Ifos_S  211   L  A  S  Q  N  L  E  F  E  L  A  A  R  L  R  D  Q  L  I  D  230
Apha_S  211   F  A  S  Q  N  L  E  F  E  L  A  A  R  L  R  D  Q  L  I  D  230
Bsep_S  211   A  A  A  Q  N  K  N  Y  E  L  A  A  H  L  R  D  Q  M  I  G  230
```

UvrB/uvrC motif (200-234)

```
Akaw_S   59 -----------------------------------------------------------------  58
Clau_S  448 TTACACGTCATTCAAGAGCAATATATCTCAAGTTT-AGTAGTAAC---TTAGATGTGGTT 503
Pkil_S  447 TTACACGTCATTCAAGAACAACATATCTCAAGTTTCAGTAGTAAC---TTAGATGTGGTT 503
Psoy_S  447 TTACACGTCATTCAAGAACAACATATCTCAAGTTTCAGTAGTAAC---TTAGATGTGGTT 503
Vok     445 TTACACGTTATTCAATAACAAC-TATATCAAGTTTTAGTAGTAAC---TTATATGTGGTT 500
Cpac_S  691 TTGCGCACCATTCAAGAACAACACACTTCAAGT---GCCAGTAATGACTTGGATGTGGTT 747
Cfau_S  691 TTGCGTACCATTCAAGAACAACACACTTCAAGT---GCCAGTAATGACTTGGATGTGGTT 747
Cnau_S  691 TTGCGCACCATTCAAGAACAACACGCTTCAAGT---GCCAGTAATGACTTAGATGTGGTT 747
Pste_S  691 TTACGCACTATTCAAGAACAACACACTTCAAGT---GCCATTAATGACTTGGATGTGGTT 747
Rma     691 TTACGCACCATCCAAGAACAACACACCTCAAGT---TCTAGTAATGATTTAGATGTGGTT 747
Ifos_S  691 TTACGCACCATCCAAGAGCAACACACCTCAAGT---GCTAGTAATGACTTAGATGTGGTT 747
Apha_S  691 TTGCGCACCATTCAAGAACAACACACTTCAAGT---GCCAGTAATGACTTAGATGTGGTT 747
Bsep_S  691 TTGCGCACCATTCAAGAGCAGCATAGTTCGCAA---ACTTTGGGTGATATGGATGTGGTG 747
Akaw_S   21  #  #  #  #  #  #  #  #  #  #  #  #  #  #  #  #  #  #  #  #   20
Clau_S  145  L  H  V  I  Q  E  Q  Y  I  S  S  #  S  S  N  #  L  D  V  V  162
Pkil_S  145  L  H  V  I  Q  E  Q  H  I  S  S  F  S  S  N  #  L  D  V  V  163
Psoy_S  145  L  H  V  I  Q  E  Q  H  I  S  S  F  S  S  N  #  L  D  V  V  163
Vok     146  L  H  V  I  Q  *  Q  #  I  S  S  F  S  S  N  #  L  Y  V  V  163
Cpac_S  231  L  R  T  I  Q  E  Q  H  T  S  S  #  A  S  N  D  L  D  V  V  249
Cfau_S  231  L  R  T  I  Q  E  Q  H  T  S  S  #  A  S  N  D  L  D  V  V  249
Cnau_S  231  L  R  T  I  Q  E  Q  H  A  S  S  #  A  S  N  D  L  D  V  V  249
Pste_S  231  L  R  T  I  Q  E  Q  H  T  S  S  #  A  I  N  D  L  D  V  V  249
Rma     231  L  R  T  I  Q  E  Q  H  T  S  S  #  S  S  N  D  L  D  V  V  249
Ifos_S  231  L  R  T  I  Q  E  Q  H  T  S  S  #  A  S  N  D  L  D  V  V  249
Apha_S  231  L  R  T  I  Q  E  Q  H  T  S  S  #  A  S  N  D  L  D  V  V  249
Bsep_S  231  L  R  T  I  Q  E  Q  H  S  S  Q  #  T  L  G  D  M  D  V  V  249
```

UvrB/uvrC motif (200-234)

```
Akaw_S   59 -----------------------------------------------------------------  58
Clau_S  504 AGTATTGATCAATAATGAGAAGTAAGTGGTATTAAGG----------ACGTTATGGTAAA 553
Pkil_S  504 AGTATTGATCAATAATGAGGAGTAAGTGGTATTGAGG--------AT--GTTCTGGTAAA 553
Psoy_S  504 AGTATTGATCAATAATGAGGAGTAAGTGGTATTGAGG--------AT--GTTCTGGTAAA 553
Vok     501 AGTATTGATCAACAATGAGGAGTAAGTGGTATTAAGG----------ACGTTCTGGTAAA 550
Cpac_S  748 AGTATCACTCAACAAGATGGGGTAAGTGGTGTTGAGGTGTTATTTATACGCTCTGGCAAG 807
Cfau_S  748 AGTATCGCTCAACAAGATGGGGTAAGTGGTGTTGAGGTGTTATTTATACGCTCTGGTAAG 807
Cnau_S  748 AGTATCGTTCAACAAGATGGGGTAAGTGGTGTTGAGGTGTTATTTATACGCTCTGGTAAG 807
Pste_S  748 AGTATCGCTCAACAAGATGGGGTAAGTGGTGTTGAGGTGTTATTTATACGCTCTGGTAAG 807
Rma     748 AGTATCGCTCAACAAGATGGGGTAAACGGTGTTGAGGTGTTATTTATACGCTCTGGTAAG 807
Ifos_S  748 AGTATCGCCCAGCAAGATGGGGTAAGTGGTATTGAGGTGTTATTCATACGTTCTGGCAAG 807
Apha_S  748 AGTATCGCCCAGCAAGATGGAGTAAGTGGTATTGAGGTGTTATTCATACGTTCTGGTAAG 807
Bsep_S  748 AGCATTGCCACGCAGGAGGGCGCGCATGCTGTTGAAGTACTGTTTGTGCGTTCAGGTAAG 807
Akaw_S   21  #  #  #  #  #  #  #  #  #  #  #  #  #  #  #  #  #  #  #  #   20
Clau_S  163  S  I  D  Q  *  *  E  V  S  G  I  K  #  #  #  #  R  Y  G  K  178
Pkil_S  164  S  I  D  Q  *  *  G  V  S  G  I  E  #  #  #  #  #  S  G  K  178
Psoy_S  164  S  I  D  Q  *  *  G  V  S  G  I  E  #  #  #  #  #  S  G  K  178
Vok     164  S  I  D  Q  Q  *  G  V  S  G  I  K  #  #  #  #  R  S  G  K  179
Cpac_S  250  S  I  T  Q  Q  D  G  V  S  G  V  E  V  L  F  I  R  S  G  K  269
Cfau_S  250  S  I  A  Q  Q  D  G  V  S  G  V  E  V  L  F  I  R  S  G  K  269
Cnau_S  250  S  I  V  Q  Q  D  G  V  S  G  V  E  V  L  F  I  R  S  G  K  269
Pste_S  250  S  I  A  Q  Q  D  G  V  S  G  V  E  V  L  F  I  R  S  G  K  269
Rma     250  S  I  A  Q  Q  D  G  V  N  G  V  E  V  L  F  I  R  S  G  K  269
Ifos_S  250  S  I  A  Q  Q  D  G  V  S  G  I  E  V  L  F  I  R  S  G  K  269
Apha_S  250  S  I  A  Q  Q  D  G  V  S  G  I  E  V  L  F  I  R  S  G  K  269
Bsep_S  250  S  I  A  T  Q  E  G  A  H  A  V  E  V  L  F  V  R  S  G  K  269
```

```
Akaw_S    59 ------------------------------------------------------------  58
Clau_S   554 TAAATTGGATAGGTATTTATTTTACTAAAAAACTCGAATAATCTAGATATTAAACAAATC 613
Pkil_S   554 CAAATTGAATTGGTATTAATTTTACCAAAAAACTCAAATAATCTAGATATTAAACAAATC 613
Psoy_S   554 CAAATTGAATTGGTATTAATTTTACCAAAAAACTCAAATAATCTAGATATTAAACAAATC 613
Vok      551 CAAATTGAATAGGTATTTATTTTACCAAAAAACTCAAATAATCTAGATTTTAAACAAATT 610
Cpac_S   808 CAAATTGGACAAGTGTTTATTTTGCCTAAAAACTCAAATAACCAAGACATTAAACAAGTT 867
Cfau_S   808 CAAATTGGACAAGTGTTTATTTTGCCTAAAAACTCAAACAACCAAGACATTAAACAAGTT 867
Cnau_S   808 CAAATTGGACAAGTGTTTATTTTGCCTAAAAACTCAAATAACCAAGACATTAAACAAGTT 867
Pste_S   808 CAAATTGGACAAGTGTTTATTTTGCCTAAAAACTCAAACAACCAAGACATTAAACAAGTT 867
Rma      808 CAAATTGGGCAAGTATTTATTTTACCTAAAAACTCAAATAACCAAGATATTAAGCAAGTT 867
Ifos_S   808 CAAATTGGACAAGTATTTATTTTGCCTAAAAACGCCAATAACCAAGACATTAAACAAGTT 867
Apha_S   808 CAAATTGGGCAAGTATTTATTTTGCCTAAAAACTCAAATAACCAAGATATTAAACAAGTT 867
Bsep_S   808 CAAATCGGACAAGAATGTATTTTTCCAAAACACAGTAAGGGTAAGGCGCTCAAAGAAGTT 867
Akaw_S    21   #  #  #  #  #  #  #  #  #  #  #  #  #  #  #  #  #  #  #  #  20
Clau_S   179   *  I  G  *  V  F  I  L  L  K  N  S  N  N  L  D  I  K  Q  I 198
Pkil_S   179   Q  I  E  L  V  L  I  L  P  K  N  S  N  N  L  D  I  K  Q  I 198
Psoy_S   179   Q  I  E  L  V  L  I  L  P  K  N  S  N  N  L  D  I  K  Q  I 198
Vok      180   Q  I  E  *  V  F  I  L  P  K  N  S  N  N  L  D  F  K  Q  I 199
Cpac_S   270   Q  I  G  Q  V  F  I  L  P  K  N  S  N  N  Q  D  I  K  Q  V 289
Cfau_S   270   Q  I  G  Q  V  F  I  L  P  K  N  S  N  N  Q  D  I  K  Q  V 289
Cnau_S   270   Q  I  G  Q  V  F  I  L  P  K  N  S  N  N  Q  D  I  K  Q  V 289
Pste_S   270   Q  I  G  Q  V  F  I  L  P  K  N  S  N  N  Q  D  I  K  Q  V 289
Rma      270   Q  I  G  Q  V  F  I  L  P  K  N  S  N  N  Q  D  I  K  Q  V 289
Ifos_S   270   Q  I  G  Q  V  F  I  L  P  K  N  A  N  N  Q  D  I  K  Q  V 289
Apha_S   270   Q  I  G  Q  V  F  I  L  P  K  N  S  N  N  Q  D  I  K  Q  V 289
Bsep_S   270   Q  I  G  Q  E  C  I  F  P  K  H  S  K  G  K  A  L  K  E  V 289
```

```
Akaw_S    59 ------------------------------------------------------------  58
Clau_S   614 TTATCTACTTTT-TGACTTTATACTATT--TTAGATAAAAAT--CCCT--AAAATAAATA 666
Pkil_S   614 TTATCTGCTTTTTTGCCTTTATACTAT---TTAGATAAAAATATCCCTAA---------- 660
Psoy_S   614 TTATCTGCTTTTTTGCCTTTATACTAT---TTAGATAAAAATATCCCTAA---------- 660
Vok      611 TTATCTGCTTTTTTGCTTTTATACTAT---TTAGATAAAAATATCCCT---AAACAAATA 664
Cpac_S   868 TTATCTGCTTTTTTGCCTTTATATTAT---TTGGGTAAAAATATCCCA---AAACAAATA 921
Cfau_S   868 TTATCTGCTTTTTTGCCTTTGTATTAT---TTGGATAAAAACATCCCA---AAACAAATA 921
Cnau_S   868 TTATCTGCTTTTTTGCCTTTGTATTAT---TTGGGCAAAAATATTCCA---AAACAAATA 921
Pste_S   868 TTATCTGCTTTTTTGCCTTTGTATTAT---TTGGGCAAAAATATCCCA---AAAAAAATA 921
Rma      868 TTATCTGCGTTTTTGCCTTTATATTAT---TTGGGTAAAAATACCCCA---AAACAAATA 921
Ifos_S   868 TTATCTGCTTTTTTGCCTTTATATTAT---TTGGGTAAAAACAACCCA---AAACAAATA 921
Apha_S   868 TTATCTGCTTTTTTGCCTTTGTATTAT---TTGGGCAAAAACACCCCA---AAACAAATA 921
Bsep_S   868 TTATCCGCTTTTTTGCCGTTATATTAT---TTAGGCAAACAGACGCCA---AAACAATTA 921
Akaw_S    21   #  #  #  #  #  #  #  #  #  #  #  #  #  #  #  #  #  #  #  #  20
Clau_S   199   L  S  T  F  #  T  L  Y  Y  #  L  D  K  N  #  P  #  K  *  I 214
Pkil_S   199   L  S  A  F  L  P  L  Y  Y  #  L  D  K  N  I  P  #  #  #  # 213
Psoy_S   199   L  S  A  F  L  P  L  Y  Y  #  L  D  K  N  I  P  #  #  #  # 213
Vok      200   L  S  A  F  L  L  L  Y  Y  #  L  D  K  N  I  P  #  K  Q  I 217
Cpac_S   290   L  S  A  F  L  P  L  Y  Y  #  L  G  K  N  I  P  #  K  Q  I 307
Cfau_S   290   L  S  A  F  L  P  L  Y  Y  #  L  D  K  N  I  P  #  K  Q  I 307
Cnau_S   290   L  S  A  F  L  P  L  Y  Y  #  L  G  K  N  I  P  #  K  Q  I 307
Pste_S   290   L  S  A  F  L  P  L  Y  Y  #  L  G  K  N  I  P  #  K  K  I 307
Rma      290   L  S  A  F  L  P  L  Y  Y  #  L  G  K  N  T  P  #  K  Q  I 307
Ifos_S   290   L  S  A  F  L  P  L  Y  Y  #  L  G  K  N  N  P  #  K  Q  I 307
Apha_S   290   L  S  A  F  L  P  L  Y  Y  #  L  G  K  N  T  P  #  K  Q  I 307
Bsep_S   290   L  S  A  F  L  P  L  Y  Y  #  L  G  K  Q  T  P  #  K  Q  L 307
```

```
Akaw_S   59 TTAAATAGTCATAAATTGAATGATAAACATATAATTTCCTCAGCTCTAAATACGCATATT  118
Clau_S  667 TTAATTAGTCATAATTTAAATTATAAACATATAATTACCTCAGCTTTAAACACGCATATT  726
Pkil_S  661 ------------------------------------------------------------  660
Psoy_S  661 ------------------------------------------------------------  660
Vok     665 TTAATTAGTTATAAATTGAATGATAAACATATAATTTCTTCAGCTTTAAATACGCATATT  724
Cpac_S  922 TTAATTAGCCACAAGTTGAGTGATAAGAAAACAATTGCTTCGGCTTTAAATACGCACATT  981
Cfau_S  922 TTAATTAGCCAGAAGTTGAGTGATAAAAAAACAATTGCTTCGGCTTTAAATACGCACATT  981
Cnau_S  922 TTAATTAGCCTCAAGTTGAGTGATAAAAAAACAATTGCTTCGGCTTTAAATACGCACATT  981
Pste_S  922 TTAATTAGCCACAAGTTGAGTGATAAAAAAACAATTACTTCGGCTTTAAATACGCACATT  981
Rma     922 TTAATTAGCCATAAATTGAGTGATAAAAAAATAATTGCTTCGGCTTTAAATACACATATT  981
Ifos_S  922 TTAATTAGCCATAAGTTGAGCGATAAAAAAACAATTGCCTCAGCTTTAAATACGCACATT  981
Apha_S  922 TTAATTAGCCATAAGTTGAGTGATAAAAAAACAATTGCCTCAGTTTTAAATACGCACATT  981
Bsep_S  922 TTACTCAACGAAAAATTAGTAGATAAAAAAATCATTGCCTCTGCCTTATCCACACAAATC  981
Akaw_S   21   L  N  S  H  K  L  N  D  K  H  I  I  S  S  A  L  N  T  H  I   40
Clau_S  215   L  I  S  H  N  L  N  Y  K  H  I  I  T  S  A  L  N  T  H  I  234
Pkil_S  214   #  #  #  #  #  #  #  #  #  #  #  #  #  #  #  #  #  #  #  #  213
Psoy_S  214   #  #  #  #  #  #  #  #  #  #  #  #  #  #  #  #  #  #  #  #  213
Vok     218   L  I  S  Y  K  L  N  D  K  H  I  I  S  S  A  L  N  T  H  I  237
Cpac_S  308   L  I  S  H  K  L  S  D  K  K  T  I  A  S  A  L  N  T  H  I  327
Cfau_S  308   L  I  S  Q  K  L  S  D  K  K  T  I  A  S  A  L  N  T  H  I  327
Cnau_S  308   L  I  S  L  K  L  S  D  K  K  T  I  A  S  A  L  N  T  H  I  327
Pste_S  308   L  I  S  H  K  L  S  D  K  K  T  I  T  S  A  L  N  T  H  I  327
Rma     308   L  I  S  H  K  L  S  D  K  K  I  I  A  S  A  L  N  T  H  I  327
Ifos_S  308   L  I  S  H  K  L  S  D  K  K  T  I  A  S  A  L  N  T  H  I  327
Apha_S  308   L  I  S  H  K  L  S  D  K  K  T  I  A  S  V  L  N  T  H  I  327
Bsep_S  308   L  L  N  E  K  L  V  D  K  K  I  I  A  S  A  L  S  T  Q  I  327
```

```
Akaw_S  119 ATTGATAGACTATATTAAGATAAAAGACATTATCTTAATATAGCTAATTTAACTGTTAAA  178
Clau_S  727 ATTGATAGACTAAATAAAGATAAAGTACATTATCTTAATATAGCTAATTTAACCGTTAAA  786
Pkil_S  661 ------------------------------------------------------------  660
Psoy_S  661 ------------------------------------------------------------  660
Vok     725 ATTGACAGACTAAATAAATATAAA-GACATTATCTTAATATAGCTAATTTAACCGTTAAG  783
Cpac_S  982 ATTGATACGCTAAATAAGGACAAACAGCATTATCTAAATGTTGCTAATTTAACCGCCAAG 1041
Cfau_S  982 ATTGACACGTCAAATAAGGATAAACAGCATTATCTAAATATTGCTAATTTAACTGCCAAG 1041
Cnau_S  982 ATTGACACGCCAAATAAGGACAAACAGCATTATCTAAATATTGCTAATTTAACTGCCAAG 1041
Pste_S  982 ATTGACACGCCAAATAAGGACAAACAGCATTATCTAAATATTGCTAATTTAACTGCCAAG 1041
Rma     982 ATTGATACACCGAACAAAGACAAAAAGCATTATCTAAATATTGCCAATTTAACCGCTAAG 1041
Ifos_S  982 ATTGATACACCAAATAAAGACAAAAAGCATTATCTTAATATTGCTAATTTAACCGCCAAG 1041
Apha_S  982 ATTGATACACCAAATAAAGACAAAAAGTATTATCTAAATATTGCTAATTTAACCGCTAAG 1041
Bsep_S  982 ATTGACACACCGCAAAAAGACAAACGCCATTTTTTAAAAATTGCCAGTCTGACTGCCGAA 1041
Akaw_S   41   I  D  R  L  Y  *  D  K  R  H  Y  L  N  I  A  N  L  T  V  K   60
Clau_S  235   I  D  R  L  N  K  D  K  V  H  Y  L  N  I  A  N  L  T  V  K  254
Pkil_S  214   #  #  #  #  #  #  #  #  #  #  #  #  #  #  #  #  #  #  #  #  213
Psoy_S  214   #  #  #  #  #  #  #  #  #  #  #  #  #  #  #  #  #  #  #  #  213
Vok     238   I  D  R  L  N  K  Y  K  #  H  Y  L  N  I  A  N  L  T  V  K  256
Cpac_S  328   I  D  T  L  N  K  D  K  Q  H  Y  L  N  V  A  N  L  T  A  K  347
Cfau_S  328   I  D  T  S  N  K  D  K  Q  H  Y  L  N  I  A  N  L  T  A  K  347
Cnau_S  328   I  D  T  P  N  K  D  K  Q  H  Y  L  N  I  A  N  L  T  A  K  347
Pste_S  328   I  D  T  P  N  K  D  K  Q  H  Y  L  N  I  A  N  L  T  A  K  347
Rma     328   I  D  T  P  N  K  D  K  K  H  Y  L  N  I  A  N  L  T  A  K  347
Ifos_S  328   I  D  T  P  N  K  D  K  K  H  Y  L  N  I  A  N  L  T  A  K  347
Apha_S  328   I  D  T  P  N  K  D  K  K  Y  Y  L  N  I  A  N  L  T  A  K  347
Bsep_S  328   I  D  T  P  Q  K  D  K  R  H  F  L  K  I  A  S  L  T  A  E  347
```

```
Akaw_S   179 GAAAATCTCAATCAAT--AAT--G-TATTATCAAGATTTA--AAAAAATCAACACTTATA  231
Clau_S   787 GAAAATCTTAATCAAT--AAT---ATATTATCAAGATTTA-AAAAAAATCAACACTTGTA  840
Pkil_S   661 ------------------------------------------------------------  660
Psoy_S   661 ------------------------------------------------------------  660
Vok      784 GAAAATCTTAATCAA---AAT--GTTATTATCAAGATTT---AAAAAATCAAGACTTGTG  835
Cpac_S  1042 GAAAATCTTAATCAA---AAT---TTATTATCAAAATTTAGAAAAAAATCAACACTTGTT 1095
Cfau_S  1042 GAAAATCTTAATCAA---AAT---TTATTATCAAGATTTAGAAAAAAATCAACACTTGTT 1095
Cnau_S  1042 GAAAATCTTAATCAA---AAT---TTATTATCAAGATTTAGAAAAAAATCAACACTTGTT 1095
Pste_S  1042 GAAAATCTTAATCAA---AAT---TTATTATCAAAATTTAGAAAAAAATCAACACTTGTT 1095
Rma     1042 GAAAATCTTAATCAA---AAT---TTATTATCAAAATTTAGAAAAAAATCAACACTTGTT 1095
Ifos_S  1042 GAAAATCTTAATCAA---AAT---TTATTATCAAGATTTAGAAAAAAATCAACACTTGTG 1095
Apha_S  1042 GAAAATCTTAATCAA---AGT---TTATTATCAAGATTTAGAAAAAAATCAACACTTGTG 1095
Bsep_S  1042 GAAAATCTAAAGCAA---TAT---TTAAACTCAAGATTCACCAAACGCTCACAATTAGAA 1095
Akaw_S    61   E  N  L  N  Q  #  N  #  #  L  S  R  F  #  K  K  S  T  L  I   76
Clau_S   255   E  N  L  N  Q  #  N  #  I  L  S  R  F  #  K  K  S  T  L  V  271
Pkil_S   214   #  #  #  #  #  #  #  #  #  #  #  #  #  #  #  #  #  #  #  #  213
Psoy_S   214   #  #  #  #  #  #  #  #  #  #  #  #  #  #  #  #  #  #  #  #  213
Vok      257   E  N  L  N  Q  #  N  #  L  L  S  R  F  #  K  K  S  R  L  V  273
Cpac_S   348   E  N  L  N  Q  #  N  #  L  L  S  K  F  R  K  K  S  T  L  V  365
Cfau_S   348   E  N  L  N  Q  #  N  #  L  L  S  R  F  R  K  K  S  T  L  V  365
Cnau_S   348   E  N  L  N  Q  #  N  #  L  L  S  R  F  R  K  K  S  T  L  V  365
Pste_S   348   E  N  L  N  Q  #  N  #  L  L  S  K  F  R  K  K  S  T  L  V  365
Rma      348   E  N  L  N  Q  #  N  #  L  L  S  K  F  R  K  K  S  T  L  V  365
Ifos_S   348   E  N  L  N  Q  #  N  #  L  L  S  R  F  R  K  K  S  T  L  V  365
Apha_S   348   E  N  L  N  Q  #  S  #  L  L  S  R  F  R  K  K  S  T  L  V  365
Bsep_S   348   E  N  L  K  Q  #  Y  #  L  N  S  R  F  T  K  R  S  Q  L  E  365
```

```
Akaw_S   232 CACTTACAA---GTTATT--AGAATGAGAGAAGTTACCTAATTATATTTAATGTTTTGAT  286
Clau_S   841 GACTTACAA---GCTATTTTAGAATTAGATAAGTTGCTTAATTATATTGAATATTTTGAT  897
Pkil_S   661 ------------------------------------------------------------  660
Psoy_S   661 ------------------------------------------------------------  660
Vok      836 CACCTACAAA--GTTATTTTAGAGTTAGACAAGTTGCCTAACCATATTCTTACTTTT---  890
Cpac_S  1096 CACTTGCAA---GTTATTTTAGGATTAGACAAATTACCTAATTATATTGAATGCTTTGAT 1152
Cfau_S  1096 CACTTGCAA---GTTACTTTAGGATTGGATAAACTACCTAATTATATTGAATGTTTTGAT 1152
Cnau_S  1096 CACTTGCAA---GTTATTTTAGGATTGGGTAAATTACCTAATTATATTGAATGCTTTGAT 1152
Pste_S  1096 CACTTGCAA---GTTATTTTAGGATTGGGTAAATTACCTAATTATATTGAATGCTTTGAT 1152
Rma     1096 CATTTGCAA---GTTATTTTAGGATTGGGCAAATTACCTAATTATATTGAATGCTTTGAT 1152
Ifos_S  1096 CATTTGCAA---GTTATTTTGGGATTAGGTAAATTGCCTAATTATATTGAATGCTTTGAT 1152
Apha_S  1096 CATTTGCAA---GTTATTTTGGAATTGGGCAAATTTCCTAATTATATTGAATGCTTTGAT 1152
Bsep_S  1096 CAACTACAA---AAAATCCTCAATCTTAAATCCCTGCCTAATTATATGGAGTGCTTTGAT 1152
Akaw_S    77   H  L  Q  #  V  I  #  E  *  E  K  L  P  N  Y  I  *  C  F  D   94
Clau_S   272   D  L  Q  #  A  I  L  E  L  D  K  L  L  N  Y  I  E  Y  F  D  290
Pkil_S   214   #  #  #  #  #  #  #  #  #  #  #  #  #  #  #  #  #  #  #  #  213
Psoy_S   214   #  #  #  #  #  #  #  #  #  #  #  #  #  #  #  #  #  #  #  #  213
Vok      274   H  L  Q  #  V  I  L  E  L  D  K  L  P  N  H  I  L  T  F  #  291
Cpac_S   366   H  L  Q  #  V  I  L  G  L  D  K  L  P  N  Y  I  E  C  F  D  384
Cfau_S   366   H  L  Q  #  V  T  L  G  L  D  K  L  P  N  Y  I  E  C  F  D  384
Cnau_S   366   H  L  Q  #  V  I  L  G  L  G  K  L  P  N  Y  I  E  C  F  D  384
Pste_S   366   H  L  Q  #  V  I  L  G  L  G  K  L  P  N  Y  I  E  C  F  D  384
Rma      366   H  L  Q  #  V  I  L  G  L  G  K  L  P  N  Y  I  E  C  F  D  384
Ifos_S   366   H  L  Q  #  V  I  L  G  L  G  K  L  P  N  Y  I  E  C  F  D  384
Apha_S   366   H  L  Q  #  V  I  L  E  L  G  K  F  P  N  Y  I  E  C  F  D  384
Bsep_S   366   Q  L  Q  #  K  I  L  N  L  K  S  L  P  N  Y  M  E  C  F  D  384
E. coli                                                  E  C  F  D
```

UvrC_HhH_N domain (367-526)

```
Akaw_S   287 ATTAGTCATATAATGGATGAG-ATAATATTGCCTTATGTGTGGTAT--TTTTAAA-GGGA  342
Clau_S   898 ATTAGTCATATAATGGGTGAAGTAAATGTTGCTTTATGTGTGGTAT--TTTTAAAAAGAA  955
Pkil_S   661 ------------------------------------------------------------  660
Psoy_S   661 ------------------------------------------------------------  660
Vok      891 ------------------------------------------------------------  890
Cpac_S  1153 ATTAGCCATATGATGGGCGAAGCAACAGTTGCCTCGTGCGTAGTG---TTTGAAAAAGGC 1209
Cfau_S  1153 ATTAGCCATATGATGGGCGAGGCAACAGTTGCTTCGTGCGTAGTG---TTTGAAAAAGGC 1209
Cnau_S  1153 ATTAGCCATATGATGGGCGAAGCAACAGTTGCCTCGTGCATAGTA---TTTGAAAAAGGC 1209
Pste_S  1153 ATTAGCCATATGATGGGCGAAGCAACGGTTGCCTCGTGCGTAGTG---TTTGAAAAAGGT 1209
Rma     1153 ATTAGCCATATGATGGGTGAGGCAACTGTTGCCTCATGCGTGGTG---TTTGAAAAAGGT 1209
Ifos_S  1153 ATTAGCCATATGATGGGTGAGGCAACTGTTGCCTCGTGCGTGGTG---TTTGAAAAAGGT 1209
Apha_S  1153 ATTAGCCATATGATGGGCGAGGCAACTGTTGCCTCGTGCGTAGTA---TTTGAAAAAGGC 1209
Bsep_S  1153 ATCAGCCATACAATGGGTGAGGCAACAGTTGCATCGTGTGTGGTG---TTTGAAAAAGGA 1209
Akaw_S    95   I  S  H  I  M  D  E  #  N  I  A  L  C  V  V  #  F  *  #  G  111
Clau_S   291   I  S  H  I  M  G  E  V  N  V  A  L  C  V  V  #  F  *  K  E  309
Pkil_S   214   #  #  #  #  #  #  #  #  #  #  #  #  #  #  #  #  #  #  #  #  213
Psoy_S   214   #  #  #  #  #  #  #  #  #  #  #  #  #  #  #  #  #  #  #  #  213
Vok      292   #  #  #  #  #  #  #  #  #  #  #  #  #  #  #  #  #  #  #  #  291
Cpac_S   385   I  S  H  M  M  G  E  A  T  V  A  S  C  V  V  #  F  E  K  G  403
Cfau_S   385   I  S  H  M  M  G  E  A  T  V  A  S  C  V  V  #  F  E  K  G  403
Cnau_S   385   I  S  H  M  M  G  E  A  T  V  A  S  C  I  V  #  F  E  K  G  403
Pste_S   385   I  S  H  M  M  G  E  A  T  V  A  S  C  V  V  #  F  E  K  G  403
Rma      385   I  S  H  M  M  G  E  A  T  V  A  S  C  V  V  #  F  E  K  G  403
Ifos_S   385   I  S  H  M  M  G  E  A  T  V  A  S  C  V  V  #  F  E  K  G  403
Apha_S   385   I  S  H  M  M  G  E  A  T  V  A  S  C  V  V  #  F  E  K  G  403
Bsep_S   385   I  S  H  T  M  G  E  A  T  V  A  S  C  V  V  #  F  E  K  G  403
E. coli        I  S  H
```

UvrC_HhH_N domain (367-526)

```
Akaw_S   343 GTCCAAAAAGTGAGCTAATATTTTCAATTTGGTA--A-T---AAAAATATTACTACTAAT  396
Clau_S   956 TCT-CAGAAGTGAGCTAATATC-----TTCGAGT--ATT-GT-AAAATACTACTACTAGT 1005
Pkil_S   661 ------------------------------------------------------------  660
Psoy_S   661 ------------------------------------------------------------  660
Vok      891 ------------------------------------------------------------  890
Cpac_S  1210 ATGCCAAAAGTGAGTCAATATCGCCAATTTGAC---ATT---AAAAATATTATCCCTGGT 1263
Cfau_S  1210 GTGCTAGAAGTGAGTCAATATTGTCAATTTGAC---ATT---AAAAATATTACCTCTGGT 1263
Cnau_S  1210 GTTCCCAAAGTAAGTCAATATCGCCAATTTGAC---ATT---AAAGATATTACCCCTGGT 1263
Pste_S  1210 GTGCCAAAAGTGAGTCAATATCGCCAATTTGAC---ATT---AAAAATATTACCCCTGGT 1263
Rma     1210 GTACCAAAAGCGAGTCAATATCGCCAATTTGAT---ATT---AAAAACATTATCCCTGGT 1263
Ifos_S  1210 ATACCCAAAGTGAGTCAATATCGCCAGTTTGAC---ATT---AAAAATATTACCTCTGGT 1263
Apha_S  1210 GTACCAAAAGTGAGCCAATATTGCCAATTTGAC---ATT---AAAAATATTACCCCTGGT 1263
Bsep_S  1210 TTGCCAAAAGTCAAAAGATACCGTCAATTTAAT---ATT---AACAATATCACCCCAGGT 1263
Akaw_S   112   V  Q  K  V  S  *  Y  F  Q  F  G  #  #  #  K  N  I  T  T  N  128
Clau_S   310   S  #  E  V  S  *  Y  #  #  F  E  #  I  #  #  N  T  T  T  S  323
Pkil_S   214   #  #  #  #  #  #  #  #  #  #  #  #  #  #  #  #  #  #  #  #  213
Psoy_S   214   #  #  #  #  #  #  #  #  #  #  #  #  #  #  #  #  #  #  #  #  213
Vok      292   #  #  #  #  #  #  #  #  #  #  #  #  #  #  #  #  #  #  #  #  291
Cpac_S   404   M  P  K  V  S  Q  Y  R  Q  F  D  #  I  #  K  N  I  I  P  G  421
Cfau_S   404   V  L  E  V  S  Q  Y  C  Q  F  D  #  I  #  K  N  I  T  S  G  421
Cnau_S   404   V  P  K  V  S  Q  Y  R  Q  F  D  #  I  #  K  D  I  T  P  G  421
Pste_S   404   V  P  K  V  S  Q  Y  R  Q  F  D  #  I  #  K  N  I  T  P  G  421
Rma      404   V  P  K  A  S  Q  Y  R  Q  F  D  #  I  #  K  N  I  I  P  G  421
Ifos_S   404   I  P  K  V  S  Q  Y  R  Q  F  D  #  I  #  K  N  I  T  S  G  421
Apha_S   404   V  P  K  V  S  Q  Y  C  Q  F  D  #  I  #  K  N  I  T  P  G  421
Bsep_S   404   L  P  K  V  K  R  Y  R  Q  F  N  #  I  #  N  N  I  T  P  G  421
E. coli                                                            P  G
```

UvrC_HhH_N domain (367-526)

```
Akaw_S   397 GATGATTATAGTGCGATGAATAAAGTTATATATCAATGTTATTCTAAACTATGGAAAAAT  456
Clau_S  1006 GATGATTATAGTACGATGAATCAAG----------------------------------- 1030
Pkil_S   661 ------------------------------------------------------------  660
Psoy_S   661 ------------------------------------------------------------  660
Vok      891 ------------------------------------------------------------  890
Cpac_S  1264 GATGACTATGCTGCAATGAATCAAGTCGTGTATCGGCGTTACTCTAAACTACTAAAGGAT 1323
Cfau_S  1264 GATGACTATGCTGCAATGAATCAAGTCGTGTATCGGCGTTATTCTAAACTACTAAAGGAT 1323
Cnau_S  1264 GATGATTATGCTGCAATGAATCAAGTCGTGTATCGGCGTTATTCTAAACTACTAAAGGAT 1323
Pste_S  1264 GATGATTATGCTGCAATGAATCAAGTCGTGTATCGGCGTTATTCTAAACTACTAAAGGAT 1323
Rma     1264 GACGATTATGCTGCAATGAATCAAGTTGTGTATAGGCGTTATTCTAAACTACTAAAGGAT 1323
Ifos_S  1264 GATGATTATGCTGCAATGAACCAAGTTGTGTATCGGCGTTATTCTAAACTATTAAAAGAT 1323
Apha_S  1264 GATGATTATGCCGCAATGAATCAAGTTGTGTACCGGCGTTATTCTAAATTACTAAAGGAT 1323
Bsep_S  1264 GATGATTATGCGGCAATGAATCAAGTCGTTTTCCGACGCTATTCTCGTTTACTCAAAGAT 1323
Akaw_S   129   D  D  Y  S  A  M  N  K  V  I  Y  Q  C  Y  S  K  L  W  K  N  148
Clau_S   324   D  D  Y  S  T  M  N  Q  #  #  #  #  #  #  #  #  #  #  #  #  331
Pkil_S   214   #  #  #  #  #  #  #  #  #  #  #  #  #  #  #  #  #  #  #  #  213
Psoy_S   214   #  #  #  #  #  #  #  #  #  #  #  #  #  #  #  #  #  #  #  #  213
Vok      292   #  #  #  #  #  #  #  #  #  #  #  #  #  #  #  #  #  #  #  #  291
Cpac_S   422   D  D  Y  A  A  M  N  Q  V  V  Y  R  R  Y  S  K  L  L  K  D  441
Cfau_S   422   D  D  Y  A  A  M  N  Q  V  V  Y  R  R  Y  S  K  L  L  K  D  441
Cnau_S   422   D  D  Y  A  A  M  N  Q  V  V  Y  R  R  Y  S  K  L  L  K  D  441
Pste_S   422   D  D  Y  A  A  M  N  Q  V  V  Y  R  R  Y  S  K  L  L  K  D  441
Rma      422   D  D  Y  A  A  M  N  Q  V  V  Y  R  R  Y  S  K  L  L  K  D  441
Ifos_S   422   D  D  Y  A  A  M  N  Q  V  V  Y  R  R  Y  S  K  L  L  K  D  441
Apha_S   422   D  D  Y  A  A  M  N  Q  V  V  Y  R  R  Y  S  K  L  L  K  D  441
Bsep_S   422   D  D  Y  A  A  M  N  Q  V  V  F  R  R  Y  S  R  L  L  K  D  441
E. coli        D  D  Y  A  A  M
```

UvrC_HhH_N domain (367-526)

```
Akaw_S   457 AAAAAA-CTTTACCATATATTATTTTTATCGATGATAGGTTGGGATAACTGAGTCAAGCT  515
Clau_S  1031 ----------------------------------------------------------CT 1032
Pkil_S   661 ------TCTTTACCAGATATTATTTTTATCGATGGTAGGTTGGGACAATTCAATCAAGCT  714
Psoy_S   661 ------TCTTTACCAGATATTATTTTTATCGATGGTAGGTTGGGACAATTCAATCAAGCT  714
Vok      891 ------------------------------------------------------------  890
Cpac_S  1324 AAAAAGCCTTTACCAGATATTATTTTTATTGATGGCGGACTAGGGCAGTTTAATCAGGCT 1383
Cfau_S  1324 AAAAAGCCTTTACCAGACATTATCTTTATTGATGGGGGATTAGGGCAGTTCAATCAGGTT 1383
Cnau_S  1324 AAAAAGCCTTTACCAGACATTATCTTTATTGATGGCGGATTAGGGCAGCTCAATCAGGCT 1383
Pste_S  1324 AAAAAGCCTTTACCAGACATTATCTTTATTGATGGCGGATTAGGGCAGTTCAATCAGGCT 1383
Rma     1324 AAAAAGCCTTTACCAGATATTATCTTTATTGATGGCGGATTAGGACAGTTTAATCAGGCT 1383
Ifos_S  1324 AAAAAGCCTTTACCAGACATTATCTTTATTGATGGCGGACTGGGGCAGCTCAATCAGGCT 1383
Apha_S  1324 AAAAAGCCTTTACCAGACATTATTTTTATTGATGGCGGATTGGGGCAGCTCAATCAAGCC 1383
Bsep_S  1324 AAGCAGCCATTGCCAGATATTGTTTTCATTGACGGTGGCTTAGGGCAACTTAATCAAGCG 1383
Akaw_S   149   K  K  #  L  P  Y  I  I  F  I  D  D  R  L  G  *  L  S  Q  A  167
Clau_S   332   #  #  #  #  #  #  #  #  #  #  #  #  #  #  #  #  #  #  #  #  331
Pkil_S   214   #  #  S  L  P  D  I  I  F  I  D  G  R  L  G  Q  F  N  Q  A  231
Psoy_S   214   #  #  S  L  P  D  I  I  F  I  D  G  R  L  G  Q  F  N  Q  A  231
Vok      292   #  #  #  #  #  #  #  #  #  #  #  #  #  #  #  #  #  #  #  #  291
Cpac_S   442   K  K  P  L  P  D  I  I  F  I  D  G  G  L  G  Q  F  N  Q  A  461
Cfau_S   442   K  K  P  L  P  D  I  I  F  I  D  G  G  L  G  Q  F  N  Q  V  461
Cnau_S   442   K  K  P  L  P  D  I  I  F  I  D  G  G  L  G  Q  L  N  Q  A  461
Pste_S   442   K  K  P  L  P  D  I  I  F  I  D  G  G  L  G  Q  F  N  Q  A  461
Rma      442   K  K  P  L  P  D  I  I  F  I  D  G  G  L  G  Q  F  N  Q  A  461
Ifos_S   442   K  K  P  L  P  D  I  I  F  I  D  G  G  L  G  Q  L  N  Q  A  461
Apha_S   442   K  K  P  L  P  D  I  I  F  I  D  G  G  L  G  Q  L  N  Q  A  461
Bsep_S   442   K  Q  P  L  P  D  I  V  F  I  D  G  G  L  G  Q  L  N  Q  A  461
E. coli                                 L  I  D  G  G  K  G
```

UvrC_HhH_N domain (367-526)

```
Akaw_S   516 T--ATTACATCGATTCATTTAATTAGTTTGG--AATAATGTGCAATTAGTAGGTAACATT  571
Clau_S  1033 ---ATTACGTCGATTAATTCAATTAGTTTGGATAAT---GTGTAATTGGTAGGT---ATT 1083
Pkil_S   715 ---ATTACGTCGATTAATTCAATTGGTTTGGATAAT--TGTACAATTGGTAGGT---ATT  766
Psoy_S   715 ---ATTACGTCGATTAATTCAATTGGTTTGGATAAT--TGTACAATTGGTAGGT---ATT  766
Vok      891 ------------------------------------------------------------  890
Cpac_S  1384 ---ATTATGGTGATGGATTCAATCGGCATGGATGAT---GTACAATTGGTAGGC---ATT 1434
Cfau_S  1384 ---ATTATGGTGATGAATTCAATCGGCGTGGATGAT---GTACAATTGGTAGGC---ATT 1434
Cnau_S  1384 ---ATTATGGTGATGAATTCAATTGGTGTGGATGAT---GTACAATTGGTAGGC---GTT 1434
Pste_S  1384 ---ATTATGGTGATGAATTCAATCGGCATGGATGGT---GTACAATTGGTAGGC---ATT 1434
Rma     1384 ---ATTATGGTGATGAATTCAATTGGCGTGGATGAT---GTGCAATTGGTAGGC---ATT 1434
Ifos_S  1384 ---ATTATGGTAATGAATTCAATCGGCGTGGATGAT---GTGCAATTGGTAGGT---ATT 1434
Apha_S  1384 ---ATCATGGTGATGAATTCAATCGGTGTGGATGAT---GTACAATTGGTAGGC---ATT 1434
Bsep_S  1384 ---ATTATGGTGATGGACTCCATCGGCATTGAATCC---ATTCAATTGGTTGGC---GTG 1434
Akaw_S   168   #  I  T  S  I  H  L  I  S  L  #  N  N  V  Q  L  V  G  N  I   185
Clau_S   332   #  I  T  S  I  N  S  I  S  L  D  N  #  V  *  L  V  G  #  I   348
Pkil_S   232   #  I  T  S  I  N  S  I  G  L  D  N  #  V  Q  L  V  G  #  I   248
Psoy_S   232   #  I  T  S  I  N  S  I  G  L  D  N  #  V  Q  L  V  G  #  I   248
Vok      292   #  #  #  #  #  #  #  #  #  #  #  #  #  #  #  #  #  #  #  #   291
Cpac_S   462   #  I  M  V  M  D  S  I  G  M  D  D  #  V  Q  L  V  G  #  I   478
Cfau_S   462   #  I  M  V  M  N  S  I  G  V  D  D  #  V  Q  L  V  G  #  I   478
Cnau_S   462   #  I  M  V  M  N  S  I  G  V  D  D  #  V  Q  L  V  G  #  V   478
Pste_S   462   #  I  M  V  M  N  S  I  G  M  D  G  #  V  Q  L  V  G  #  I   478
Rma      462   #  I  M  V  M  N  S  I  G  V  D  D  #  V  Q  L  V  G  #  I   478
Ifos_S   462   #  I  M  V  M  N  S  I  G  V  D  D  #  V  Q  L  V  G  #  I   478
Apha_S   462   #  I  M  V  M  N  S  I  G  V  D  D  #  V  Q  L  V  G  #  I   478
Bsep_S   462   #  I  M  V  M  D  S  I  G  I  E  S  #  I  Q  L  V  G  #  V   478
```

UvrC_HhH_N domain (367-526)

```
Akaw_S   572 GCCAAGGGGAAAAATCAAAAAGTAGGGATTGAGATTTTAATTATAGTTAGAGATAGTAAA  631
Clau_S  1084 GCCAAGGGTAAAAATCAAAAAGTAGGGCTTGAGATTTTAATTATAGTTAGAGATAGTAAA 1143
Pkil_S   767 ATCAAGAGGGAAAGTCAAAAAGAAGGGCTTGAAATTTTAATTATAGTTAGAGATAGTAAA  826
Psoy_S   767 ATCAAGAGGGAAAGTCAAAAAGAAGGGCTTGAAATTTTAATTATAGTTAGAGATAGTAAA  826
Vok      891 ------------------------------------------------------------  890
Cpac_S  1435 GCCAAGGGAGAGAATCGAAAGGCAGGGCTTGAGACTTTAATTACGGTTAAAGATGATAAG 1494
Cfau_S  1435 GCCAAGGGAGAGAATCTAAAGACAGGGCTTGAGACCTTAATTACGGTTAAAGATGATAAG 1494
Cnau_S  1435 GCCAAGGGAGAGAATCGAAAGGCAGGGCTTGAGACTTTAATTACGGTTAAAGATGATAAG 1494
Pste_S  1435 GCCAAGGGAGAGAATCGAAAGGCAGGGCTTGAGACTTTAATTACGGTTAAAGATGATAAG 1494
Rma     1435 GCCAAGGGAGAAAATAGAAAGGCAGGGCTTGAAACTTTAATTACGATTAAAGATGATAAT 1494
Ifos_S  1435 GCCAAGGGAGAGAATCGAAAAGCAGGGCTTGAAACTTTAATTATGGTTAAAGACGACAAG 1494
Apha_S  1435 GCCAAAGGAGAGAGGCGAAAAGCAGGGCTTGAGACTTTAATTACAGTTAAAGATGATAAA 1494
Bsep_S  1435 GCAAAAGGCGAGGGAAGGAAAGCTGGACTTGAGACTTTGATTATGGTCACAGATGGCAAA 1494
Akaw_S   186   A  K  G  K  N  Q  K  V  G  I  E  I  L  I  I  V  R  D  S  K   205
Clau_S   349   A  K  G  K  N  Q  K  V  G  L  E  I  L  I  I  V  R  D  S  K   368
Pkil_S   249   I  K  R  E  S  Q  K  E  G  L  E  I  L  I  I  V  R  D  S  K   268
Psoy_S   249   I  K  R  E  S  Q  K  E  G  L  E  I  L  I  I  V  R  D  S  K   268
Vok      292   #  #  #  #  #  #  #  #  #  #  #  #  #  #  #  #  #  #  #  #   291
Cpac_S   479   A  K  G  E  N  R  K  A  G  L  E  T  L  I  T  V  K  D  D  K   498
Cfau_S   479   A  K  G  E  N  L  K  T  G  L  E  T  L  I  T  V  K  D  D  K   498
Cnau_S   479   A  K  G  E  N  R  K  A  G  L  E  T  L  I  T  V  K  D  D  K   498
Pste_S   479   A  K  G  E  N  R  K  A  G  L  E  T  L  I  T  V  K  D  D  K   498
Rma      479   A  K  G  E  N  R  K  A  G  L  E  T  L  I  T  I  K  D  D  N   498
Ifos_S   479   A  K  G  E  N  R  K  A  G  L  E  T  L  I  M  V  K  D  D  K   498
Apha_S   479   A  K  G  E  R  R  K  A  G  L  E  T  L  I  T  V  K  D  D  K   498
Bsep_S   479   A  K  G  E  G  R  K  A  G  L  E  T  L  I  M  V  T  D  G  K   498
```

UvrC_HhH_N domain (367-526)

```
Akaw_S  632 GTTAATAAAATCAACCCACTAGCTCACGACCCTGCTTTTATATTAGTGAATCGTATC---  688
Clau_S 1144 GTTAATAAAATCAACATACTATCTCACGACCATGCTTTTATGTTAGTGAATCGT-TTTC- 1201
Pkil_S  827 GTTAATAAAATGAACCTACTACCTTACGATCATACTTTTATGTTAGTGAATCGCATC---  883
Psoy_S  827 GTTAATAAAATGAACCTACTACCTTACGATCATACTTTTATGTTAGTGAATCGCATC---  883
Vok     891 --------------------------------------ATGTTAGTGAATCGAATC---  908
Cpac_S 1495 ATCAATAAAATTGATCTACCACCTTACGACCCTGTGCTTATGTTAGTCAACCGTATT--- 1551
Cfau_S 1495 GTCAATAAAATTAATCTACCACCTTACGACCCTGCGCTTATGTTAGTCAAGCGCATT--- 1551
Cnau_S 1495 GTCAATAAAATCAATCTACCACCTTACGATCCTGCGCTTATGTTAGTTAACCGCATT--- 1551
Pste_S 1495 GTCAATAAAATCAATCTACCACCTTACGACCCTGCGCTTATGTTAGTCAACCGCATT--- 1551
Rma    1495 GTCAATAAAATCAATCTACTACCTTATGACCCTGCGCTTATGTTAGTCAATCACATT--- 1551
Ifos_S 1495 GTTAATAAAATCAGTCTACCACCTTACGACCCTGCGCTTATGCTGGTCAACCACATT--- 1551
Apha_S 1495 GTTAATAAAATCAATCTACCACCTCACGATCCTGCACTTATGTTGGTCAACCACATT--- 1551
Bsep_S 1495 ACACAAAAAATCAACCTACATCCACAGGATCAGGCTTTAATGTTGGTCAACCATATC--- 1551
Akaw_S  206   V  N  K  I  N  P  L  A  H  D  P  A  F  I  L  V  N  R  I  #  224
Clau_S  369   V  N  K  I  N  I  L  S  H  D  H  A  F  M  L  V  N  R  #  #  386
Pkil_S  269   V  N  K  M  N  L  L  P  Y  D  H  T  F  M  L  V  N  R  I  #  287
Psoy_S  269   V  N  K  M  N  L  L  P  Y  D  H  T  F  M  L  V  N  R  I  #  287
Vok     292   #  #  #  #  #  #  #  #  #  #  #  #  #  M  L  V  N  R  I  #  297
Cpac_S  499   I  N  K  I  D  L  P  P  Y  D  P  V  L  M  L  V  N  R  I  #  517
Cfau_S  499   V  N  K  I  N  L  P  P  Y  D  P  A  L  M  L  V  K  R  I  #  517
Cnau_S  499   V  N  K  I  N  L  P  P  Y  D  P  A  L  M  L  V  N  R  I  #  517
Pste_S  499   V  N  K  I  N  L  P  P  Y  D  P  A  L  M  L  V  N  R  I  #  517
Rma     499   V  N  K  I  N  L  L  P  Y  D  P  A  L  M  L  V  N  H  I  #  517
Ifos_S  499   V  N  K  I  S  L  P  P  Y  D  P  A  L  M  L  V  N  H  I  #  517
Apha_S  499   V  N  K  I  N  L  P  P  H  D  P  A  L  M  L  V  N  H  I  #  517
Bsep_S  499   T  Q  K  I  N  L  H  P  Q  D  Q  A  L  M  L  V  N  H  I  #  517
```

UvrC_HhH_N domain (367-526)

```
Akaw_S  689 CGGGATGAATTGCCCCGTTTTGTAACGAAGATTTATCTAAAAAAC-TT-GTTTTTAAGAT  746
Clau_S 1202 AGA-ATGAATCA-------TT---ATC-----TT----AAAAAAC-TT-GTTTTTAATAT 1239
Pkil_S  884 CGGGATGAATCAC-CAGTTTTGTAATGAAGAATTATCGAAAAAAC-TT-GTTTTTAAGAT  940
Psoy_S  884 CGGGATGAATCAC-CAGTTTTGTAATGAAGAATTATCGAAAAAAC-TT-GTTTTTAAGAT  940
Vok     909 CGGAATGAATCA-TCAGTTTTGTAATGAAGAATTATCGAAAAAAC-TT-GTTTTTAAGAT  965
Cpac_S 1552 CGAGACGAATCACACCGTTTTGCGATT------------AAAAATCATCGAAAAAAACGT 1599
Cfau_S 1552 CGAGACGAATCACACCGTTTTGCGATT------------AAAAATTATCGAAAGAAATGT 1599
Cnau_S 1552 CGAGACGAATCACACCGTTTTGCGATT------------AAAAATCATCGAAAGAAACGT 1599
Pste_S 1552 CGAGACGAATCACACCGTTTTGCGATT------------AAAAATCATCGAAAGAAACGT 1599
Rma    1552 CGAAACGAATCACACCGTTTTGCGATT------------AAAAATCATCGAAAGAAACGC 1599
Ifos_S 1552 CGAGACGAATCACACCGTTTTGCGATT------------AAAAATCATCGAAAAAAACGT 1599
Apha_S 1552 CGAGACGAGTCACATCGTTTTGCAATT------------AAAAATCATCGAAAGAAACGC 1599
Bsep_S 1552 CGCGATGAGTCTCACCGTTTTGCCATT------------AAAAACCATCGTAAAAAACGC 1599
Akaw_S  225   R  D  E  L  P  R  F  V  T  K  I  Y  L  K  N  #  #  F  *  D  242
Clau_S  387   R  #  E  S  #  #  #  #  I  #  #  #  L  K  N  #  #  F  *  Y  396
Pkil_S  288   R  D  E  S  #  S  F  V  M  K  N  Y  R  K  N  #  #  F  *  D  304
Psoy_S  288   R  D  E  S  #  S  F  V  M  K  N  Y  R  K  N  #  #  F  *  D  304
Vok     298   R  N  E  S  #  S  F  V  M  K  N  Y  R  K  N  #  #  F  *  D  314
Cpac_S  518   R  D  E  S  H  R  F  A  I  #  #  #  #  K  N  H  R  K  K  R  533
Cfau_S  518   R  D  E  S  H  R  F  A  I  #  #  #  #  K  N  Y  R  K  K  C  533
Cnau_S  518   R  D  E  S  H  R  F  A  I  #  #  #  #  K  N  H  R  K  K  R  533
Pste_S  518   R  D  E  S  H  R  F  A  I  #  #  #  #  K  N  H  R  K  K  R  533
Rma     518   R  N  E  S  H  R  F  A  I  #  #  #  #  K  N  H  R  K  K  R  533
Ifos_S  518   R  D  E  S  H  R  F  A  I  #  #  #  #  K  N  H  R  K  K  R  533
Apha_S  518   R  D  E  S  H  R  F  A  I  #  #  #  #  K  N  H  R  K  K  R  533
Bsep_S  518   R  D  E  S  H  R  F  A  I  #  #  #  #  K  N  H  R  K  K  R  533
E. coli          D  E  S  H  D  H
```

UvrC_HhH_N domain (367-526)

```
Akaw_S   747 --GT--------ACAACTTTAATATTAGAATCTATTGTA---GAAGTAGGAAAGTTACGT  793
Clau_S  1240 --GT--------ACAACTTCAATATTATAATCTATTATTA--GGAGTAATAAAGTTATGT 1287
Pkil_S   941 --GT--------ACAACTTCAATATTAGAATCTATTATA---GGAGTAGGAAAGTTACGT  987
Psoy_S   941 --GT--------ACAACTTCAATATTAGAATCTATTATA---GGAGTAGGAAAGTTACGT  987
Vok      966 --GT--------ATAACTTCAATATTAGAATCTATTATA---GGAGTAGTAAAGTTACGT 1012
Cpac_S  1600 GCGTCAAGACGCACAATCTCAACACTAGAATCTATTATA---GGTGTAGGAAGATTACGT 1656
Cfau_S  1600 GCGTCAAGACGCACAATCTCAATACTAGAATCTATTATA---GGTGTAGGAAAATTACGT 1656
Cnau_S  1600 GCCTCAAGACGTACAATCTCAATACTAGAATCTATTATA---GGTGTAGGAAAATTACGT 1656
Pste_S  1600 GCGTCAAGACGCACAATCTCAATACTAGAATCTATTATA---GGTGTAGGAAAATTACGT 1656
Rma     1600 GCTTCAAGACGTACAACCTCAATATTAGAATCTATTAAA---GGTGTAGGAAAATTACGC 1656
Ifos_S  1600 GCTTCAAGACGTACAACTTCAATACTAGAATCCATTACA---GGTGTAGGAAAATTACGC 1656
Apha_S  1600 GCTTCAAAACGTACAACTTCAATACTAGAATCCATTATA---GGCGTGGGAAAGTTACGC 1656
Bsep_S  1600 GGAAAAAAACGCACAACCTCTCCATTAGAGGGTATTGAA---GGCGTAGGAAAGCAGCGT 1656
Akaw_S   243   #  #  #  #  T  T  L  I  L  E  S  I  V  #  E  V  G  K  L  R  257
Clau_S   397   #  #  #  #  T  T  S  I  L  *  S  I  I  #  G  V  I  K  L  C  411
Pkil_S   305   #  #  #  #  T  T  S  I  L  E  S  I  I  #  G  V  G  K  L  R  319
Psoy_S   305   #  #  #  #  T  T  S  I  L  E  S  I  I  #  G  V  G  K  L  R  319
Vok      315   #  #  #  #  I  T  S  I  L  E  S  I  I  #  G  V  V  K  L  R  329
Cpac_S   534   A  S  R  R  T  I  S  T  L  E  S  I  I  #  G  V  G  R  L  R  552
Cfau_S   534   A  S  R  R  T  I  S  I  L  E  S  I  I  #  G  V  G  K  L  R  552
Cnau_S   534   A  S  R  R  T  I  S  I  L  E  S  I  I  #  G  V  G  K  L  R  552
Pste_S   534   A  S  R  R  T  I  S  I  L  E  S  I  I  #  G  V  G  K  L  R  552
Rma      534   A  S  R  R  T  T  S  I  L  E  S  I  K  #  G  V  G  K  L  R  552
Ifos_S   534   A  S  R  R  T  T  S  I  L  E  S  I  T  #  G  V  G  K  L  R  552
Apha_S   534   A  S  K  R  T  T  S  I  L  E  S  I  I  #  G  V  G  K  L  R  552
Bsep_S   534   G  K  K  R  T  T  S  P  L  E  G  I  E  #  G  V  G  K  Q  R  552

E. coli                    T  S  S  L  E  T  I  E  #  G  V  G
```

HhH_5 domain (536-593)

```
Akaw_S   794 AGAATACGT--TCTACTTAATTAT----------TACAAGAAGTAAAGAAAGCATAGTTT  841
Clau_S  1288 AGAATA---GCTCTATTTAATTATTTTGCTGGTTTACAAGAAGTAAAGAAAG-ATCGATT 1343
Pkil_S   988 AGAATA---GCTCTACTTAATTATTTTGTTGGTTTACAAGAAGTAAAGAAAGCATCTATT 1044
Psoy_S   988 AGAATA---GCTCTACTTAATTATTTTGTTGGTTTACAAGAAGTAAAGAAAGCATCTATT 1044
Vok     1013 AGAATA-G-G-TCTACTTAATTATTTTGTTGGTTTACAAGAAGTAAAGAAAGCATCTATT 1069
Cpac_S  1657 AGAATG---GCATTGCTGAATTACTTTGGTGGTTTGCAAGAAGTGAAAAAAGCATCGATT 1713
Cfau_S  1657 AGAATG---ACATTGCTGAATTACTTTGGTGGTTTTCAAGAAGTGAAAAAAGCATCGATT 1713
Cnau_S  1657 AGAATG---GCACTACTGAATTACTTTGGTGGTTTGCAAGAAGTGAAAAAAGCATCGATT 1713
Pste_S  1657 AGAATG---GCACTGCTGAATTACTTTGGTGGTTTGCAAGAAGTGAAAAAAGCATCGATT 1713
Rma     1657 AGAATG---GCGTTACTTAATTATTTTGGTGGTTTGCAAGAAATAAAAAAAGCATCTATT 1713
Ifos_S  1657 AGAATA---GCGCTACTTAATTATTTTGGTGGTTTGCAAGAAGTTAAAAAAGCATCGATT 1713
Apha_S  1657 AGAATG---GCACTGCTTAATTATTTTGGCGGTTTGCAAGAAGTGAAGAAGGCATCGATT 1713
Bsep_S  1657 CGCAGC---GCTTTACTTAATCATTTTGGTGGTTTGCAAGAAATACAAAAAGCATCAGTT 1713
Akaw_S   258   R  I  R  #  L  L  N  Y  #  #  #  #  Q  E  V  K  K  A  *  F  272
Clau_S   412   R  I  #  A  L  F  N  Y  F  A  G  L  Q  E  V  K  K  #  S  I  429
Pkil_S   320   R  I  #  A  L  L  N  Y  F  V  G  L  Q  E  V  K  K  A  S  I  338
Psoy_S   320   R  I  #  A  L  L  N  Y  F  V  G  L  Q  E  V  K  K  A  S  I  338
Vok      330   R  I  #  #  L  L  N  Y  F  V  G  L  Q  E  V  K  K  A  S  I  347
Cpac_S   553   R  M  #  A  L  L  N  Y  F  G  G  L  Q  E  V  K  K  A  S  I  571
Cfau_S   553   R  M  #  T  L  L  N  Y  F  G  G  F  Q  E  V  K  K  A  S  I  571
Cnau_S   553   R  M  #  A  L  L  N  Y  F  G  G  L  Q  E  V  K  K  A  S  I  571
Pste_S   553   R  M  #  A  L  L  N  Y  F  G  G  L  Q  E  V  K  K  A  S  I  571
Rma      553   R  M  #  A  L  L  N  Y  F  G  G  L  Q  E  I  K  K  A  S  I  571
Ifos_S   553   R  I  #  A  L  L  N  Y  F  G  G  L  Q  E  V  K  K  A  S  I  571
Apha_S   553   R  M  #  A  L  L  N  Y  F  G  G  L  Q  E  V  K  K  A  S  I  571
Bsep_S   553   R  S  #  A  L  L  N  H  F  G  G  L  Q  E  I  Q  K  A  S  V  571

E. coli           R  Q  M  L  L  K  Y  M  G  G  L  Q  G  L  R  N  A  S  V
```

HhH_5 domain (536-593)

```
Akaw_S   842 -ATGAAATGCAAAAAGTTAGTTGTATTAATTTAATATTAGCGACTAAAATAGTCGAAAAA  900
Clau_S  1344 CATGAAATTCAAAAAGTTAGTTGTATTAATTTAATATTAGCGACTAAAATAGTTGAAAAA 1403
Pkil_S  1045 CATGAAACTCAAAAAGTTAGTTGTATTAATTTAATATTAGCGACTAAAATAGTTGAAAAA 1104
Psoy_S  1045 CATGAAACTCAAAAAGTTAGTTGTATTAATTTAATATTAGCGACTAAAATAGTTGAAAAA 1104
Vok     1070 CATGAAATTCAAAAAGTTAGTTGTATTAATTTAATATTAGCGACTAAAATAGTTGAAAAA 1129
Cpac_S  1714 CATGAAATTCAAAAAGTCAGTGGTATTAATTTAACATTAGCGATTAAAATAGTTGAAAAA 1773
Cfau_S  1714 CATGAAATTCAAAAAGTCAGTGGTATTAATTTAACATTAGCGACTAAAATAGTTGAAAAA 1773
Cnau_S  1714 CATGAAATTCAAAAAGTCAGTGGTATTAATTTAACATTAGCGACTAAAATAGTTGAAAAA 1773
Pste_S  1714 CATGAAATTCAAAAAGTCAGTGGTATTAATTTAACATTAGCGACTAAAATAGTTGAAAAA 1773
Rma     1714 CATGAAATTCAAAAAGTCAGCGGTATTAATTTAGTATTAGCGACTAAAATAGTTAAAAAA 1773
Ifos_S  1714 CATGAAATTCGAAAAGTCAGTGGTATTAATTTAGTATTAGCGACTAAAATAGTTGAAAAA 1773
Apha_S  1714 CATGAAATTCAAAAAGTCAGTGGTATTAATTTAGCATTAGCGACTAAAATAGTTGAAAAA 1773
Bsep_S  1714 AACGAAATTCAAAAAGTTAATGGCATCAGTCTTGCATTAGCGACTAAAATTGTAGAAAAA 1773
Akaw_S   273   #  E  M  Q  K  V  S  C  I  N  L  I  L  A  T  K  I  V  E  K  291
Clau_S   430   H  E  I  Q  K  V  S  C  I  N  L  I  L  A  T  K  I  V  E  K  449
Pkil_S   339   H  E  T  Q  K  V  S  C  I  N  L  I  L  A  T  K  I  V  E  K  358
Psoy_S   339   H  E  T  Q  K  V  S  C  I  N  L  I  L  A  T  K  I  V  E  K  358
Vok      348   H  E  I  Q  K  V  S  C  I  N  L  I  L  A  T  K  I  V  E  K  367
Cpac_S   572   H  E  I  Q  K  V  S  G  I  N  L  T  L  A  I  K  I  V  E  K  591
Cfau_S   572   H  E  I  Q  K  V  S  G  I  N  L  T  L  A  T  K  I  V  E  K  591
Cnau_S   572   H  E  I  Q  K  V  S  G  I  N  L  T  L  A  T  K  I  V  E  K  591
Pste_S   572   H  E  I  Q  K  V  S  G  I  N  L  T  L  A  T  K  I  V  E  K  591
Rma      572   H  E  I  Q  K  V  S  G  I  N  L  V  L  A  T  K  I  V  K  K  591
Ifos_S   572   H  E  I  R  K  V  S  G  I  N  L  V  L  A  T  K  I  V  E  K  591
Apha_S   572   H  E  I  Q  K  V  S  G  I  N  L  A  L  A  T  K  I  V  E  K  591
Bsep_S   572   N  E  I  Q  K  V  N  G  I  S  L  A  L  A  T  K  I  V  E  K  591

E.coli         E  E  I  A  K  V  P  G  I  S  Q  G  L  A  E  K  I  F  W
```

HhH_5 domain (536-593)

```
Akaw_S   901 CTAAAAGATTAA  912
Clau_S  1404 CTTAAAGATTAA 1415
Pkil_S  1105 CTAAAAGATTAA 1116
Psoy_S  1105 CTAAAAGATTAA 1116
Vok     1130 CTAAAAGATTAA 1141
Cpac_S  1774 CTAAAAGATTAA 1785
Cfau_S  1774 CTAAAAGATTAA 1785
Cnau_S  1774 CTTAAAGATTAA 1785
Pste_S  1774 CTAAAAGATTAA 1785
Rma     1774 ATAAAAAGTTAA 1785
Ifos_S  1774 CTAAAGGGTTAA 1785
Apha_S  1774 CTTAAAGGTTAA 1785
Bsep_S  1774 TTACGAGGTTAA 1785
Akaw_S   292   L  K  D  *  295
Clau_S   450   L  K  D  *  453
Pkil_S   359   L  K  D  *  362
Psoy_S   359   L  K  D  *  362
Vok      368   L  K  D  *  371
Cpac_S   592   L  K  D  *  595
Cfau_S   592   L  K  D  *  595
Cnau_S   592   L  K  D  *  595
Pste_S   592   L  K  D  *  595
Rma      592   I  K  S  *  595
Ifos_S   592   L  K  G  *  595
Apha_S   592   L  K  G  *  595
Bsep_S   592   L  R  G  *  595
```

HHH_5 domain (536-593)

References.

5. Verhoeven EE, van Kesteren M, Moolenaar GF, Visse R, Goosen N. Catalytic sites for 3' and 5' incision of Escherichia coli nucleotide excision repair are both located in UvrC. The Journal of biological chemistry. 2000;275(7):5120-3. PubMed PMID: 10671556.

6. Verhoeven EE, van Kesteren M, Turner JJ, van der Marel GA, van Boom JH, Moolenaar GF, et al. The C-terminal region of *Escherichia coli* UvrC contributes to the flexibility of the UvrABC nucleotide excision repair system. Nucleic Acids Res. 2002;30(11):2492-500. PubMed PMID: 12034838; PubMed Central PMCID: PMC117173.
