# Supplementary material for: Loss of genes related to Nucleotide Excision Repair (NER) and implications for reductive genome evolution in symbionts of deep-sea vesicomyid clams
Source: PLoS One. 2017 Feb 15;12(2):e0171274. doi: 10.1371/journal.pone.0171274 (PMC5310779; doi:10.1371/journal.pone.0171274)
Supplement: S5 Fig — ORFs of uvrD of all clade I and II symbionts except Abyssogena phaseoliformis and Isorropodon fossajaponica were intact. Although the ORFs of this gene in symbionts of A. phaseoliformis and I. fossajaponica were collapsed and do not code proteins, remnant amino acid coding nucleotide sequences were estimated as was possible. Conserved domains of uvrD found in an NCBI blast search are shown as bidirectional arrows. Conserved helicase sequences (motifs Ia-VI) of uvrD from Escherichia coli are shown below the alignment [7]. Variation in their length seems to have resulted from small deletions. # indicates the gap of amino acid sequence where no corresponding nucleotide sequence exist. * indicates stop codon. (PDF) [file pone.0171274.s008.pdf]

S5 Fig.

```
Akaw_S     1 ATGAATTTATCTGAAATAACAAATGGTCTAAATGACAAACAATGCCAATCAGTTACACTA   60
Clau_S     1 ATGAATTTATCTGAAATAACAAATGGTCTAAATGACAAACAATGCCAATCAGTTACACTA   60
Pkil_S     1 ATGAATTTATCTGAAATAACAAATGGTCTAAATGACAAACAATACCAATCAGTTACACTA   60
Psoy_S     1 ATGAATTTATCTGAAATAACAAATGGTCTAAATGACAAACAATACCAATCAGTTACACTA   60
Vok        1 ATGAATTTATCTGAAATAACAAATGGTCTAAATGACAAACAATGCCAATCAGTTACACTA   60
Cpac_S     1 ATGAATTTATCTGAAATAACGAATGGCCTAAACGACAAACAATGCCAATCAGTCATACTC   60
Cfau_S     1 ATGAATTTATCTGAAATAACGAATGGCCTAAACGACAAACAATGCCAATCAGTCACACTC   60
Cnau_S     1 ATGAATTTATCTGAAATAACGAATGGCCTAAACGACAAACAATGCCAACCAGTCACACTC   60
Pste_S     1 ATGAATTTATCTAAAATAACGAATGGCCTAAACGACAAACAATGCCAATCAGTCACACTC   60
Rma        1 ATGAATTTATCCGAAATAACAAACGACCTAAATGACAAACAATGCCAATCAGTCACATTC   60
Ifos_S     1 ATGAATTTATCTGAAATAACAAGCGGCCTAAACGACAAACAATGCCAATCAGTCACATTA   60
Apha_S     1 ATGAATTTATCTGAAATAACGAACGGCCTAAACGCCAAACAATGCCAATTAGTCACACTC   60
Bsep_S     1 ATGCATTTATCTGAAATAATCGACGGACTTAACGACAATCAATCTCAATCAGTTGTCTTG   60
Akaw_S     1   M  N  L  S  E  I  T  N  G  L  N  D  K  Q  C  Q  S  V  T  L   20
Clau_S     1   M  N  L  S  E  I  T  N  G  L  N  D  K  Q  C  Q  S  V  T  L   20
Pkil_S     1   M  N  L  S  E  I  T  N  G  L  N  D  K  Q  Y  Q  S  V  T  L   20
Psoy_S     1   M  N  L  S  E  I  T  N  G  L  N  D  K  Q  Y  Q  S  V  T  L   20
Vok        1   M  N  L  S  E  I  T  N  G  L  N  D  K  Q  C  Q  S  V  T  L   20
Cpac_S     1   M  N  L  S  E  I  T  N  G  L  N  D  K  Q  C  Q  S  V  I  L   20
Cfau_S     1   M  N  L  S  E  I  T  N  G  L  N  D  K  Q  C  Q  S  V  T  L   20
Cnau_S     1   M  N  L  S  E  I  T  N  G  L  N  D  K  Q  C  Q  P  V  T  L   20
Pste_S     1   M  N  L  S  K  I  T  N  G  L  N  D  K  Q  C  Q  S  V  T  L   20
Rma        1   M  N  L  S  E  I  T  N  D  L  N  D  K  Q  C  Q  S  V  T  F   20
Ifos_S     1   M  N  L  S  E  I  T  S  G  L  N  D  K  Q  C  Q  S  V  T  L   20
Apha_S     1   M  N  L  S  E  I  T  N  G  L  N  A  K  Q  C  Q  L  V  T  L   20
Bsep_S     1   M  H  L  S  E  I  I  D  G  L  N  D  N  Q  S  Q  S  V  V  L   20
```

UvrD domain (6-716)

```
Akaw_S    61 AATAATGAAAAAAACGCTTTAATACTAGCAGGTGCTGGA---AGTGGTAAAACCAAGGTT  117
Clau_S    61 AATAATAAAAAAAACGCTTTAATACTAGCAGGTGCTGGA---AGTGGTAAAACCAAAGTT  117
Pkil_S    61 AATAATGAAAAAAACGCTTTAATACTAGCAGGTGCGGGA---AGTGGTAAAACCAAAGTT  117
Psoy_S    61 AATAATGAAAAAAACGCTTTAATACTAGCAGGTGCGGGA---AGTGGTAAAACCAAAGTT  117
Vok       61 AATAATGAAAAAAACGCTTTAATACTAGCAGGTGCTGGA---AGTGGTAAAACCAAAGTT  117
Cpac_S    61 AATAATGAAAAAAATGTTTTAATACTAGCAGGTGCAGGC---AGTGGCAAGACCAGAGTT  117
Cfau_S    61 AATAATGAAAAAAATGTTTTGATACTAGCAGGTGCAGGC---AGTGGCAAGACCAGAGTT  117
Cnau_S    61 AATAATGAAAAAAATGCTTTAATACTAGCAGGTGCAGGC---AGTGGCAAGACCAGAGTT  117
Pste_S    61 AATAATGAAAAAAATGCTTTAATACTAGCAGGTGCAGGC---AGTGGCAAGACCAGAGTT  117
Rma       61 AATAATGAAAAAAATGCTTTAATACTAGCAGGTGCAGGT---AGTGGTAAAACTAGAGTT  117
Ifos_S    61 AGTAATGAAAAAAATGCTTTAATACTAGCGGGTGCAGGC---AGTGGCAAAACCAGAGTG  117
Apha_S    61 AATAATGAAAAAAATGCTTTAATACTAGCAGGTGCAGGGT--AGTGGCAAGACCAGAGTT  118
Bsep_S    61 GACAACACTCAAAATGCCTTAATACTCGCAGGTGCGGGC---TCTGGCAAGACCCGTGTG  117
Akaw_S    21   N  N  E  K  N  A  L  I  L  A  G  A  G  #  S  G  K  T  K  V   39
Clau_S    21   N  N  K  K  N  A  L  I  L  A  G  A  G  #  S  G  K  T  K  V   39
Pkil_S    21   N  N  E  K  N  A  L  I  L  A  G  A  G  #  S  G  K  T  K  V   39
Psoy_S    21   N  N  E  K  N  A  L  I  L  A  G  A  G  #  S  G  K  T  K  V   39
Vok       21   N  N  E  K  N  A  L  I  L  A  G  A  G  #  S  G  K  T  K  V   39
Cpac_S    21   N  N  E  K  N  V  L  I  L  A  G  A  G  #  S  G  K  T  R  V   39
Cfau_S    21   N  N  E  K  N  V  L  I  L  A  G  A  G  #  S  G  K  T  R  V   39
Cnau_S    21   N  N  E  K  N  A  L  I  L  A  G  A  G  #  S  G  K  T  R  V   39
Pste_S    21   N  N  E  K  N  A  L  I  L  A  G  A  G  #  S  G  K  T  R  V   39
Rma       21   N  N  E  K  N  A  L  I  L  A  G  A  G  #  S  G  K  T  R  V   39
Ifos_S    21   S  N  E  K  N  A  L  I  L  A  G  A  G  #  S  G  K  T  R  V   39
Apha_S    21   N  N  E  K  N  A  L  I  L  A  G  A  G  *  S  G  K  T  R  V   40
Bsep_S    21   D  N  T  Q  N  A  L  I  L  A  G  A  G  #  S  G  K  T  R  V   39

E. coli                       L  V  L  A  G  A  G     S  G  K  T  R  V
```

**Helicase motif Ia**

UvrD domain (6-716)

```
Akaw_S  118 CTCATACATAGAATTGCTTACTTAATCACACAAAAAGATATTCACATTGATGCTATTCTA  177
Clau_S  118 CTCATACATAGAATCGCTTACTTAATCACACAAAAAAATATTCACATTAATGCTATTCTA  177
Pkil_S  118 CTCATACATAGAATTGCTTACTTAATCACACAAAAAGATATTCATATTGATTCTATTCTA  177
Psoy_S  118 CTCATACATAGAATTGCTTACTTAATCACACAAAAAGATATTCATATTGATTCTATTCTA  177
Vok     118 CTCATACATAGAATTGCCTACTTAATCACACAAAAAGATATTCACATTGATGCTATTCTA  177
Cpac_S  118 CTAACCCGTAGAATTGCCTATTTAATCACACAAAAAAATATTCGCATTAATGCCATTCTA  177
Cfau_S  118 TTAACCCATAGAATTGTCTATTTAATCACACAAAAAGATATTCGCATTGATGCCATTCTA  177
Cnau_S  118 TTAACCCATAGAATTGCCTATTTAATCACACAAAAAGATATTCACATTGATGCCATTCTG  177
Pste_S  118 TTAACCCATAGAATTGCCTATTTAATCACACAAAAAAATATTCGCATTGATGCCATTCTG  177
Rma     118 TTAACCCATAGAATTGCCTATTTAATTACACAAAAAGATATTCGTATTGATGCCATTTTA  177
Ifos_S  118 CTCACACATAGAATCGCTTATTTAATCACACAAAAAGATATTCGAACCGATGCTATTTTA  177
Apha_S  119 TTAACCCATAGAATTGCTTATTTAGTCACACAAAAAGATATTCGCATTGATGCCATTCTG  178
Bsep_S  118 CTGACACATCGCATCGCCTATCTGGTTACACAAAAAAATGTACCGATTGATGCAATTTTA  177
Akaw_S   40  L  I  H  R  I  A  Y  L  I  T  Q  K  D  I  H  I  D  A  I  L   59
Clau_S   40  L  I  H  R  I  A  Y  L  I  T  Q  K  N  I  H  I  N  A  I  L   59
Pkil_S   40  L  I  H  R  I  A  Y  L  I  T  Q  K  D  I  H  I  D  S  I  L   59
Psoy_S   40  L  I  H  R  I  A  Y  L  I  T  Q  K  D  I  H  I  D  S  I  L   59
Vok      40  L  I  H  R  I  A  Y  L  I  T  Q  K  D  I  H  I  D  A  I  L   59
Cpac_S   40  L  T  R  R  I  A  Y  L  I  T  Q  K  N  I  R  I  N  A  I  L   59
Cfau_S   40  L  T  H  R  I  V  Y  L  I  T  Q  K  D  I  R  I  D  A  I  L   59
Cnau_S   40  L  T  H  R  I  A  Y  L  I  T  Q  K  D  I  H  I  D  A  I  L   59
Pste_S   40  L  T  H  R  I  A  Y  L  I  T  Q  K  N  I  R  I  D  A  I  L   59
Rma      40  L  T  H  R  I  A  Y  L  I  T  Q  K  D  I  R  I  D  A  I  L   59
Ifos_S   40  L  T  H  R  I  A  Y  L  I  T  Q  K  D  I  R  T  D  A  I  L   59
Apha_S   41  L  T  H  R  I  A  Y  L  V  T  Q  K  D  I  R  I  D  A  I  L   60
Bsep_S   40  L  T  H  R  I  A  Y  L  V  T  Q  K  N  V  P  I  D  A  I  L   59

E. coli      L  V  H  R  I  A  W  L  M                             M
```

**Helicase motif Ia** **Helicase motif Ib**

```
Akaw_S  178 GCCGTTACTTTCACCAATAAAGCCGCTGCTGAAATGTGTGAAAGATTAAGTACACTATTA  237
Clau_S  178 GCCGTTACTTTTACTAATAAAGCCGCTACTGAAATGTGTGAAAGATTAAGTATGCTATTA  237
Pkil_S  178 GCTGTTACTTTTACCAATAAAGCCGCTACTGAAATGTGTGAAAGATTAAGTAAGCTATTA  237
Psoy_S  178 GCTGTTACTTTTACCAATAAAGCCGCTACTGAAATGTGTGAAAGATTAAGTAAGCTATTA  237
Vok     178 GCCGTTACTTTTACCAATAAAGCCGCTACTGAAATGTGTGAAAGATTAAGTAAGCTATTA  237
Cpac_S  178 GCGGTTACTTTTACCAATAAAGCTGCTGTTGAAATGCGCGGAAAGTTAAGTATTCTATTA  237
Cfau_S  178 GCGGTTACTTTTACCAATAAAGCTGTTGCTGCAATACGTGAAAAGTTAAGCATTCTATTA  237
Cnau_S  178 GTGGCTACTTTTACCAATAAAGCTGCTGCTGAAATGCGCAAAAGGTTAAGCATTCTATTA  237
Pste_S  178 GCGGTTACTTTTACCAATAAAGCTGTTGCTGAAATGCGCGAAAGGTTAAGCATTCTATTA  237
Rma     178 GCAGTTACTTTTACCAATAAAGCTGCTACTGAAATGCGTGAAAGGCTAAGTACTTTATTA  237
Ifos_S  178 GCGGTTACTTTTACTAATAAAGCCGCTGCTGAGATGCGTGAAAGGCTAAGTACTCTATTA  237
Apha_S  179 GCGCTTACTTTTACCAATAAAGCTGCTGCTGAAATGCGCGAAAGATTAAGCATTCTATTA  238
Bsep_S  178 GCGGTAACCTTTACCAATAAAGCATCTAAAGAAATGCGTGAACGCCTTGAAGTGTTGTTG  237
Akaw_S   60  A  V  T  F  T  N  K  A  A  A  E  M  C  E  R  L  S  T  L  L   79
Clau_S   60  A  V  T  F  T  N  K  A  A  T  E  M  C  E  R  L  S  M  L  L   79
Pkil_S   60  A  V  T  F  T  N  K  A  A  T  E  M  C  E  R  L  S  K  L  L   79
Psoy_S   60  A  V  T  F  T  N  K  A  A  T  E  M  C  E  R  L  S  K  L  L   79
Vok      60  A  V  T  F  T  N  K  A  A  T  E  M  C  E  R  L  S  K  L  L   79
Cpac_S   60  A  V  T  F  T  N  K  A  A  V  E  M  R  G  K  L  S  I  L  L   79
Cfau_S   60  A  V  T  F  T  N  K  A  V  A  A  I  R  E  K  L  S  I  L  L   79
Cnau_S   60  V  A  T  F  T  N  K  A  A  A  E  M  R  K  R  L  S  I  L  L   79
Pste_S   60  A  V  T  F  T  N  K  A  V  A  E  M  R  E  R  L  S  I  L  L   79
Rma      60  A  V  T  F  T  N  K  A  A  T  E  M  R  E  R  L  S  T  L  L   79
Ifos_S   60  A  V  T  F  T  N  K  A  A  A  E  M  R  E  R  L  S  T  L  L   79
Apha_S   61  A  L  T  F  T  N  K  A  A  A  E  M  R  E  R  L  S  I  L  L   80
Bsep_S   60  A  V  T  F  T  N  K  A  S  K  E  M  R  E  R  L  E  V  L  L   79

E. coli      A  V  T  F  T  N  K  A  A  A  E  M  R  H  R  I  G
```

**Helicase motif Ib**

UvrD domain (6-716)

```
Akaw_S  238 AGGCGTTCTATTACAAGCATGTGGACTGGAACATTTCATAGCTTGGCACAC---CGATTA  294
Clau_S  238 AGGCGTCCTATTACAAGTATGTGGATTGGAACATTTCATAGCTTGGCACAC---CGATTA  294
Pkil_S  238 AGGCGTCCTATTACAAGTATGTGGACTGGAACATTTCATAGCTTGGCACAC---CGATTA  294
Psoy_S  238 AGGCGTCCTATTACAAGTATGTGGACTGGAACATTTCATAGCTTGGCACAC---CGATTA  294
Vok     238 AGGCATCCTATTACAAGTATGTGGACTGGAACATTTCATAGTTTGGCACAC---CGATTA  294
Cpac_S  238 AGGCGTCCTATTCAAAGTATGTGGGTGGGAACATTTCATGGCTTGGCACAC---CGATTA  294
Cfau_S  238 AGGCGTCCTATTCAAAGTATGTGGGTGGGAACATTTCATGGCTTGGCACAC---CGATTA  294
Cnau_S  238 AGGCGTCCTATTCAAAGTATGTGGATGGGAACATTTCATGGCTTGACACAC---CGATTA  294
Pste_S  238 AGGCGTCCTATTCAAAGTATGTGGGTGGGAACATTTCATGGCTTTGCACAC---CGATTA  294
Rma     238 AGGCGCCCTATTCAAAGTATGTGGATGGGAACATTTCATAGCTTGGCACAC---CGATTA  294
Ifos_S  238 AGACGCCCTATTCAAAGCATGTGAGTGGAAATATTTCATAGCTTGGCACAC---CGATTA  294
Apha_S  239 AGGCGCCCTATTTAAAGTATGTGGGTGGGAACATTTCATGGCTTGGCACACCC-CGATTA  297
Bsep_S  238 AGGCGACCAATTAGAACAATGTGGGTTGGCACTTTCCATGGTTTGGCACAC---CGATTA  294
Akaw_S   80   R  R  S  I  T  S  M  W  T  G  T  F  H  S  L  A  H  #  R  L   98
Clau_S   80   R  R  P  I  T  S  M  W  I  G  T  F  H  S  L  A  H  #  R  L   98
Pkil_S   80   R  R  P  I  T  S  M  W  T  G  T  F  H  S  L  A  H  #  R  L   98
Psoy_S   80   R  R  P  I  T  S  M  W  T  G  T  F  H  S  L  A  H  #  R  L   98
Vok      80   R  H  P  I  T  S  M  W  T  G  T  F  H  S  L  A  H  #  R  L   98
Cpac_S   80   R  R  P  I  Q  S  M  W  V  G  T  F  H  G  L  A  H  #  R  L   98
Cfau_S   80   R  R  P  I  Q  S  M  W  V  G  T  F  H  G  L  A  H  #  R  L   98
Cnau_S   80   R  R  P  I  Q  S  M  W  M  G  T  F  H  G  L  T  H  #  R  L   98
Pste_S   80   R  R  P  I  Q  S  M  W  V  G  T  F  H  G  F  A  H  #  R  L   98
Rma      80   R  R  P  I  Q  S  M  W  M  G  T  F  H  S  L  A  H  #  R  L   98
Ifos_S   80   R  R  P  I  Q  S  M  *  V  E  I  F  H  S  L  A  H  #  R  L   98
Apha_S   81   R  R  P  I  *  S  M  W  V  G  T  F  H  G  L  A  H  #  R  L   99
Bsep_S   80   R  R  P  I  R  T  M  W  V  G  T  F  H  G  L  A  H  #  R  L   98
```

UvrD domain (6-716)

```
Akaw_S  295 TTACGTACTCATTATGAACAAGCTCAATTAACTTCTAACTTTCAAATACTAGATGTACAA  354
Clau_S  295 TTACGTACTCATTATGAACAAGTTCAATTAACTTCTAACTTTCAAATAATAGATGCACAA  354
Pkil_S  295 TTACGCACTCATTATGAACAAGCTCAATTAACTTATAACTTTCAAATCCTAGATGCACAG  354
Psoy_S  295 TTACGCACTCATTATGAACAAGCTCAATTAACTTATAACTTTCAAATCCTAGATGCACAG  354
Vok     295 TTACGCACTCATTATGAACAAGCTCAATTAACTTATAACTTTCAAATCCTAGATGCACAA  354
Cpac_S  295 TTGCGCACTCATTATGAAAAAGCTAGGTTGACTTCTGGTTTTCAAATCCTAGATGCACAA  354
Cfau_S  295 TTGCGTGCTCATTATGAAAAAGCTAGGTTGACTTCTGGCTTTCAAATCTTAGATGCACAA  354
Cnau_S  295 TTGCGCACTCACTATGAAAAAGCTAGGTTGACTTCTGGCTTTCAAATCCTAGATGCACAA  354
Pste_S  295 TTACGCACTCATTATAAAAAAGCTAGGTTGACTTCTGGCTTTCAAATCCTAGATGCACAA  354
Rma     295 TTACGCACTCATTACGAAAAGTCTAAGTTGACTTCTGGATTTCAAATTTTAGATGCACAA  354
Ifos_S  295 TTACGCACTCATTATGAAAAAGCTAAGTTGACTTCTGGCTTTCAAATTTTAGATGCACAA  354
Apha_S  298 TTACGCACTCATTATGAGAAAGCAGGGCTAACTTCTGGCTTTAAAATCCTAGATGCACAA  357
Bsep_S  295 TTACGCACCCACGCTGCCGAAGCCAACCTCAGTCCGCAATTTCAGATTCTTGATCAGCAA  354
Akaw_S   99   L  R  T  H  Y  E  Q  A  Q  L  T  S  N  F  Q  I  L  D  V  Q  118
Clau_S   99   L  R  T  H  Y  E  Q  V  Q  L  T  S  N  F  Q  I  I  D  A  Q  118
Pkil_S   99   L  R  T  H  Y  E  Q  A  Q  L  T  Y  N  F  Q  I  L  D  A  Q  118
Psoy_S   99   L  R  T  H  Y  E  Q  A  Q  L  T  Y  N  F  Q  I  L  D  A  Q  118
Vok      99   L  R  T  H  Y  E  Q  A  Q  L  T  Y  N  F  Q  I  L  D  A  Q  118
Cpac_S   99   L  R  T  H  Y  E  K  A  R  L  T  S  G  F  Q  I  L  D  A  Q  118
Cfau_S   99   L  R  A  H  Y  E  K  A  R  L  T  S  G  F  Q  I  L  D  A  Q  118
Cnau_S   99   L  R  T  H  Y  E  K  A  R  L  T  S  G  F  Q  I  L  D  A  Q  118
Pste_S   99   L  R  T  H  Y  K  K  A  R  L  T  S  G  F  Q  I  L  D  A  Q  118
Rma      99   L  R  T  H  Y  E  K  S  K  L  T  S  G  F  Q  I  L  D  A  Q  118
Ifos_S   99   L  R  T  H  Y  E  K  A  K  L  T  S  G  F  Q  I  L  D  A  Q  118
Apha_S  100   L  R  T  H  Y  E  K  A  G  L  T  S  G  F  K  I  L  D  A  Q  119
Bsep_S   99   L  R  T  H  A  A  E  A  N  L  S  P  Q  F  Q  I  L  D  Q  Q  118
```

UvrD domain (6-716)

```
Akaw_S  355 GACCAATTTCGTATTATCAAAAACCTCATGAAAGAAAATAATATTGATGAAACTAAGTTT  414
Clau_S  355 GACCAATTCCGTATTATCAAAAATCTCATGAAAGAAAATAATATTGATGAATCTAAGTTT  414
Pkil_S  355 GACCAATTCCGCATTATCAAAAACCTAATGAAAGAAAATAATATAGATGAATCTAAGTTT  414
Psoy_S  355 GACCAATTCCGCATTATCAAAAACCTAATGAAAGAAAATAATATAGATGAATCTAAGTTT  414
Vok     355 GACCAATTCCGCATTATCAAAAACCTAATGAAAGAAAATAATATAGATGAATCTAAGTTT  414
Cpac_S  355 GACCAATTTTGCATTGTCAAACGTTTAATGAAAGAAAATAACATTGACGAATCTAAGCTT  414
Cfau_S  355 GACCAATTTCGCATTATCAAACGTTTAATGAAAGAAAATAACATTGACGAATCTAAGTTT  414
Cnau_S  355 GACCAATTTCGTATTATCAAACGTTTAATGAAAGAAAATAACATTGATGAATCTAAGTTT  414
Pste_S  355 GACCAATTTAGCATTGTCAAACGTTTAATGAAAGAAAATAACATTGATGAATCTAAGTTT  414
Rma     355 GACCAGTTTCGTATTGTCAAACGTCTAATGAAAGAAAATAGCATTGATGAATCTAAGTTT  414
Ifos_S  355 GACCAGTTTCGCATTGTCAAACGTCTAATGAAAGAAAATCACATTGATGAATCTAAGTTC  414
Apha_S  358 GACCAGTTTCGCATT------------------------------GATAAATCTAAGTTT  387
Bsep_S  355 GACCAGTATCGCATTGTTAAACGCTTAATGAAAGAAAATAGCATTGATGAATCCAAGTTT  414
Akaw_S  119  D  Q  F  R  I  I  K  N  L  M  K  E  N  N  I  D  E  T  K  F  138
Clau_S  119  D  Q  F  R  I  I  K  N  L  M  K  E  N  N  I  D  E  S  K  F  138
Pkil_S  119  D  Q  F  R  I  I  K  N  L  M  K  E  N  N  I  D  E  S  K  F  138
Psoy_S  119  D  Q  F  R  I  I  K  N  L  M  K  E  N  N  I  D  E  S  K  F  138
Vok     119  D  Q  F  R  I  I  K  N  L  M  K  E  N  N  I  D  E  S  K  F  138
Cpac_S  119  D  Q  F  C  I  V  K  R  L  M  K  E  N  N  I  D  E  S  K  L  138
Cfau_S  119  D  Q  F  R  I  I  K  R  L  M  K  E  N  N  I  D  E  S  K  F  138
Cnau_S  119  D  Q  F  R  I  I  K  R  L  M  K  E  N  N  I  D  E  S  K  F  138
Pste_S  119  D  Q  F  S  I  V  K  R  L  M  K  E  N  N  I  D  E  S  K  F  138
Rma     119  D  Q  F  R  I  V  K  R  L  M  K  E  N  S  I  D  E  S  K  F  138
Ifos_S  119  D  Q  F  R  I  V  K  R  L  M  K  E  N  H  I  D  E  S  K  F  138
Apha_S  120  D  Q  F  R  I  #  #  #  #  #  #  #  #  #  #  D  K  S  K  F  129
Bsep_S  119  D  Q  Y  R  I  V  K  R  L  M  K  E  N  S  I  D  E  S  K  F  138
```

UvrD domain (6-716)

```
Akaw_S  415 CCTATTAAAAAGGTCCAGCAATTTATTAACAATCAAAAAAACAAAGGTATTCACTTACAG  474
Clau_S  415 CCTATTAAAAAGGTTCAGCAGTTTATTAACAATCAAAAAAACAAAGGTATTCGCCTACAA  474
Pkil_S  415 CCTATTAAAAAGGTTCAGCAGTTTATTAACAATCAAAAAAACAAAGGTATTCGCCTACAG  474
Psoy_S  415 CCTATTAAAAAGGTTCAGCAGTTTATTAACAATCAAAAAAACAAAGGTATTCGCCTACAG  474
Vok     415 CCTATTAAAAAGGTTCAGCAGTTTATTAACAATCAAAAAAATAAAGGTATTCACTTACAT  474
Cpac_S  415 CCCATTAAAAAAGTGCAATGGTTTATTAACAATCAAAAAGACGAAGGTATTCGCGCACAA  474
Cfau_S  415 CCCATTAGAAAAGTGCAGTGGTTTATTAACAATCAAAAAGACGAAGGTATTCGCGCACAA  474
Cnau_S  415 CCCATTAGAAAAGTGCAGTGGTTTATTAACAATCAAAAAGACAGAGGTATTCGCGCACAA  474
Pste_S  415 CCCATTAAAAAAGTACAGTGGTTTTTTAACAATCAAAAAGACGAAGGTATTCGCGCACAA  474
Rma     415 CCCATTAAAAAAGTGCAGTGGTTTATTAATAATCAAAAAAACGAAGATATTCGCGCACAA  474
Ifos_S  415 CCCATTAGAAAGGTTCAGTGGTTTATTAACAATCAAAAAGGCGAAGGTATTCGCCCGCAA  474
Apha_S  388 CCCATTAGAAAGGTTCAGTGGTTTATTAACAATCAAAAAGATGAGGGTATTCGCGTACAG  447
Bsep_S  415 CCCGTCCGTAAAGTGCAGTGGTTTATTAATCAGCAAAAAGATGAAGGTATTCGCCCAGGT  474
Akaw_S  139  P  I  K  K  V  Q  Q  F  I  N  N  Q  K  N  K  G  I  H  L  Q  158
Clau_S  139  P  I  K  K  V  Q  Q  F  I  N  N  Q  K  N  K  G  I  R  L  Q  158
Pkil_S  139  P  I  K  K  V  Q  Q  F  I  N  N  Q  K  N  K  G  I  R  L  Q  158
Psoy_S  139  P  I  K  K  V  Q  Q  F  I  N  N  Q  K  N  K  G  I  R  L  Q  158
Vok     139  P  I  K  K  V  Q  Q  F  I  N  N  Q  K  N  K  G  I  H  L  H  158
Cpac_S  139  P  I  K  K  V  Q  W  F  I  N  N  Q  K  D  E  G  I  R  A  Q  158
Cfau_S  139  P  I  R  K  V  Q  W  F  I  N  N  Q  K  D  E  G  I  R  A  Q  158
Cnau_S  139  P  I  R  K  V  Q  W  F  I  N  N  Q  K  D  R  G  I  R  A  Q  158
Pste_S  139  P  I  K  K  V  Q  W  F  F  N  N  Q  K  D  E  G  I  R  A  Q  158
Rma     139  P  I  K  K  V  Q  W  F  I  N  N  Q  K  N  E  D  I  R  A  Q  158
Ifos_S  139  P  I  R  K  V  Q  W  F  I  N  N  Q  K  G  E  G  I  R  P  Q  158
Apha_S  130  P  I  R  K  V  Q  W  F  I  N  N  Q  K  D  E  G  I  R  V  Q  149
Bsep_S  139  P  V  R  K  V  Q  W  F  I  N  Q  Q  K  D  E  G  I  R  P  G  158
```

UvrD domain (6-716)

```
Akaw_S  475 GATATTGACTCTAGTTACAACTATTTTATTAAAAAAAGTCTTAAAGTATTT---AAGCTT  531
Clau_S  475 GATATTGACCCTAGTTACAACTATTTTATTAAAAAAAGTCTTAAAGTATTT---GAGCTT  531
Pkil_S  475 GATATTGACTCTAGTTACAACTATTTTATTAAAAAAAGTCTTAAAGTATTT---GAGCTT  531
Psoy_S  475 GATATTGACTCTAGTTACAACTATTTTATTAAAAAAAGTCTTAAAGTATTT---GAGCTT  531
Vok     475 GATATTGACTCTAGTTACAACTATTTTATTAAAAAAAGTCTTAAAGTATTT---GAGCTT  531
Cpac_S  475 AACATTGATCCTGGTTATAACTATTTTGTTAAAAAAAATCTTGAAGTGTTT---GAACTT  531
Cfau_S  475 GACATTGACCCTGGTTATAACTATTTTATTAAAAATAATCTTGAAGTGTTT---GAACTC  531
Cnau_S  475 GACATTGACCCTAGTTATAACTATTTTGTTAAAAAAAGTCTTGAAGTGTTT---GAGCTT  531
Pste_S  475 GACATTGACCCTGGTTATAACTATTTTGTTAAAAAAAGTCTTGAAGTGTTT---GAGCTT  531
Rma     475 GATATTGACCCTGATTATAACTATTTTATTAAAAAAAATCTTGAAGTGTTT---GAACTT  531
Ifos_S  475 GACATTGATCCTGGTTATAACTATTTTGTTAAAA--AGTCTTGAAGTGTTTT--GAACTT  530
Apha_S  448 GATGTTGATCCTGGTTATAACTATTTTATTAAGAAAAGCCTTGAGGTATTT---GAACTT  504
Bsep_S  475 GATATCGATGCAGGACATAATTATTTTGTTAAGAAAAGCCTTGAAGTCTTT---GGTTTT  531
Akaw_S  159   D  I  D  S  S  Y  N  Y  F  I  K  K  S  L  K  V  F  #  K  L  177
Clau_S  159   D  I  D  P  S  Y  N  Y  F  I  K  K  S  L  K  V  F  #  E  L  177
Pkil_S  159   D  I  D  S  S  Y  N  Y  F  I  K  K  S  L  K  V  F  #  E  L  177
Psoy_S  159   D  I  D  S  S  Y  N  Y  F  I  K  K  S  L  K  V  F  #  E  L  177
Vok     159   D  I  D  S  S  Y  N  Y  F  I  K  K  S  L  K  V  F  #  E  L  177
Cpac_S  159   N  I  D  P  G  Y  N  Y  F  V  K  K  N  L  E  V  F  #  E  L  177
Cfau_S  159   D  I  D  P  G  Y  N  Y  F  I  K  N  N  L  E  V  F  #  E  L  177
Cnau_S  159   D  I  D  P  S  Y  N  Y  F  V  K  K  S  L  E  V  F  #  E  L  177
Pste_S  159   D  I  D  P  G  Y  N  Y  F  V  K  K  S  L  E  V  F  #  E  L  177
Rma     159   D  I  D  P  D  Y  N  Y  F  I  K  K  N  L  E  V  F  #  E  L  177
Ifos_S  159   D  I  D  P  G  Y  N  Y  F  V  K  #  S  L  E  V  F  #  E  L  176
Apha_S  150   D  V  D  P  G  Y  N  Y  F  I  K  K  S  L  E  V  F  #  E  L  168
Bsep_S  159   D  I  D  A  G  H  N  Y  F  V  K  K  S  L  E  V  F  #  G  F  177
```

UvrD domain (6-716)

```
Akaw_S  532 TATGAAGTACACTGCAAAACAAATAACTTGGTTGATTTTGCAGAACTGCTGATACGTAGT  591
Clau_S  532 TATGAAGTACACTGCAAAACAAATGACTTGATTGATTTTGCAGAACTGCTGATACGTAGT  591
Pkil_S  532 TATGAAGTACACTGCAAAACAAATGACTTGGTTGATTTTGCAGAACTGCTGATACGTAGT  591
Psoy_S  532 TATGAAGTACACTGCAAAACAAATGACTTGGTTGATTTTGCAGAACTGCTGATACGTAGT  591
Vok     532 TATGAAGTACACTGCAAAACAAATGAATTGATTGATTTTGCAGAACTGTTGATACGTAGT  591
Cpac_S  532 TATGAGGCACACTGTCAAATAAATGACTTAATTGATTTTGCAGAACTACTGATACGTAGT  591
Cfau_S  532 TATGAGGCACACTGTCAAACAAATGACTTAATTGATTTTTCAGAACTACTGATACGTAGT  591
Cnau_S  532 TATGAGGCACACTGCCAAACAAATGACTTAATTGATTTTTCAGAACTACTGATACGTAGT  591
Pste_S  532 TATGAAGCACACTGCCAAACAAATGACTTAATTGATTTTGCAGAACTACTGATACGTAGT  591
Rma     532 TATGAAGCGTATTGCCAAGAAAACGACTTAATTGATTTTGCAGAACTACTGATACGTAGT  591
Ifos_S  531 TATGAAGCGCACTGCCAAGCAAATGACCTAGTTGATTTTGCAGAACTACTGATACGTAGT  590
Apha_S  505 TATGAGACTCACTACCAAGCAAATGACTTAATTGATTTTGC-------------------  545
Bsep_S  532 TATGAAAATCATTGCAACGCCAACGGCTTAATCGATTTTGCCGAATTATTAGTTCGCAGT  591
Akaw_S  178   Y  E  V  H  C  K  T  N  N  L  V  D  F  A  E  L  L  I  R  S  197
Clau_S  178   Y  E  V  H  C  K  T  N  D  L  I  D  F  A  E  L  L  I  R  S  197
Pkil_S  178   Y  E  V  H  C  K  T  N  D  L  V  D  F  A  E  L  L  I  R  S  197
Psoy_S  178   Y  E  V  H  C  K  T  N  D  L  V  D  F  A  E  L  L  I  R  S  197
Vok     178   Y  E  V  H  C  K  T  N  E  L  I  D  F  A  E  L  L  I  R  S  197
Cpac_S  178   Y  E  A  H  C  Q  I  N  D  L  I  D  F  A  E  L  L  I  R  S  197
Cfau_S  178   Y  E  A  H  C  Q  T  N  D  L  I  D  F  S  E  L  L  I  R  S  197
Cnau_S  178   Y  E  A  H  C  Q  T  N  D  L  I  D  F  S  E  L  L  I  R  S  197
Pste_S  178   Y  E  A  H  C  Q  T  N  D  L  I  D  F  A  E  L  L  I  R  S  197
Rma     178   Y  E  A  Y  C  Q  E  N  D  L  I  D  F  A  E  L  L  I  R  S  197
Ifos_S  177   Y  E  A  H  C  Q  A  N  D  L  V  D  F  A  E  L  L  I  R  S  196
Apha_S  169   Y  E  T  H  Y  Q  A  N  D  L  I  D  F  #  #  #  #  #  #  #  181
Bsep_S  178   Y  E  N  H  C  N  A  N  G  L  I  D  F  A  E  L  L  V  R  S  197
```

UvrD domain (6-716)

```
Akaw_S  592 TATGAGTTATTAAAAAATAACGTAAACTTACTTAATCATTATAAAGATCGCTTTAAGCAC  651
Clau_S  592 TATGAGTTATTAAAAAATAACGTAAACTTACTTAATTATTATAAAGATCTCTTTAAGCAC  651
Pkil_S  592 TATGATTTATTAAAAAATAACATAAACTTACTTAACCATTATAAAGATCGCTTTGAACAC  651
Psoy_S  592 TATGATTTATTAAAAAATAACATAAACTTACTTAACCATTATAAAGATCGCTTTGAACAC  651
Vok     592 TATGAGTTATTAAAAAATAACGTAAACTTACTTAACCATTATAAAAATCGCTTTAAGCAC  651
Cpac_S  592 TATGAGTTATTAAAAAATAACACGGATTTGCTTAATCATTATCAAGCGCGCTTTGAGCAT  651
Cfau_S  592 TATGAGTTATTAAAAAATAACACGGACTTGCTTAATCATTATCAAGCGCGCTTTAAGCAC  651
Cnau_S  592 TATGAGTTATTAAAAAATAACACGGACTTACTTAATCATTATCAAACGCGCTTTGAATAT  651
Pste_S  592 TATGAGTTATTAAAAAATAACACGGACTTGCTTAATCATTATCAAGCGCGCTTTGAGCAT  651
Rma     592 TATGAGTTGTTAAAAAATAACACAGACTTGCTTAATCATTATCAAGCGCGCTTTGAGCAT  651
Ifos_S  591 TATGAGCTATTAAAAAATAACACAGACTTGCTTAATCATTATCAAGCGCGCTTTGAGCAT  650
Apha_S  546 ------------------------------------------------------------  545
Bsep_S  592 TACGAGCTATTAAGAAACAATTCTGAATTGTTGACTTACTACCAAACGCGCTTTCAACAT  651
Akaw_S  198   Y  E  L  L  K  N  N  V  N  L  L  N  H  Y  K  D  R  F  K  H  217
Clau_S  198   Y  E  L  L  K  N  N  V  N  L  L  N  Y  Y  K  D  L  F  K  H  217
Pkil_S  198   Y  D  L  L  K  N  N  I  N  L  L  N  H  Y  K  D  R  F  E  H  217
Psoy_S  198   Y  D  L  L  K  N  N  I  N  L  L  N  H  Y  K  D  R  F  E  H  217
Vok     198   Y  E  L  L  K  N  N  V  N  L  L  N  H  Y  K  N  R  F  K  H  217
Cpac_S  198   Y  E  L  L  K  N  N  T  D  L  L  N  H  Y  Q  A  R  F  E  H  217
Cfau_S  198   Y  E  L  L  K  N  N  T  D  L  L  N  H  Y  Q  A  R  F  K  H  217
Cnau_S  198   Y  E  L  L  K  N  N  T  D  L  L  N  H  Y  Q  T  R  F  E  Y  217
Pste_S  198   Y  E  L  L  K  N  N  T  D  L  L  N  H  Y  Q  A  R  F  E  H  217
Rma     198   Y  E  L  L  K  N  N  T  D  L  L  N  H  Y  Q  A  R  F  E  H  217
Ifos_S  197   Y  E  L  L  K  N  N  T  D  L  L  N  H  Y  Q  A  R  F  E  H  216
Apha_S  182   #  #  #  #  #  #  #  #  #  #  #  #  #  #  #  #  #  #  #  #  181
Bsep_S  198   Y  E  L  L  R  N  N  S  E  L  L  T  Y  Y  Q  T  R  F  Q  H  217
```

UvrD domain (6-716)

```
Akaw_S  652 ATTTTAATTGATGAATTTCAAGACACTAGCATAATC---CAATACAAATGGATTAAATTA  708
Clau_S  652 ATTTTAATTGATGAATTTCAAGACACTAACATGATC---CAATACAAATGGATTAAATTA  708
Pkil_S  652 ATTTTAATTGATGAATTTCAAGACACTAACATGATC---CAATATAAATGGATTAAATTA  708
Psoy_S  652 ATTTTAATTGATGAATTTCAAGACACTAACATGATC---CAATATAAATGGATTAAATTA  708
Vok     652 ATTTTAATTGATGAATTTCAAGACACTAATATGATC---CAATACAAATGGATTAAATTA  708
Cpac_S  652 ATTTTAGTGGATGAGTTTCAAGACACCAATACGGTG---CAATATAAATGGATTAAATTA  708
Cfau_S  652 ATTTTAGTGGACGAGTTTCAAGACACCAATACGGTG---CAATATAAATGGATTAAATTA  708
Cnau_S  652 ATTTTAGTGGATGAGTTTCAAGACACCAATACGATG---CAATATGAATGGATTAAATTA  708
Pste_S  652 ATTTTAGTGGATGAGTTTCAAGACACCAATACGGTG---CAATTTAAATGGATTAAATTA  708
Rma     652 ATTTTAGTGGATGAGTTTCAAGATACCAATACGGTG---CAGTACAAATGGATTAAATTA  708
Ifos_S  651 ATTTTAGTGGATGAATTTCAAGATACCAATACGGTGC--CAATACAAATGGATTAAATTA  708
Apha_S  546 ------------------------------------------------------------  545
Bsep_S  652 ATCTTAGTTGATGAATTTCAAGACACCAATACCGTA---CAATACCGATGGATTCAGCAG  708
Akaw_S  218   I  L  I  D  E  F  Q  D  T  S  I  I  #  Q  Y  K  W  I  K  L  236
Clau_S  218   I  L  I  D  E  F  Q  D  T  N  M  I  #  Q  Y  K  W  I  K  L  236
Pkil_S  218   I  L  I  D  E  F  Q  D  T  N  M  I  #  Q  Y  K  W  I  K  L  236
Psoy_S  218   I  L  I  D  E  F  Q  D  T  N  M  I  #  Q  Y  K  W  I  K  L  236
Vok     218   I  L  I  D  E  F  Q  D  T  N  M  I  #  Q  Y  K  W  I  K  L  236
Cpac_S  218   I  L  V  D  E  F  Q  D  T  N  T  V  #  Q  Y  K  W  I  K  L  236
Cfau_S  218   I  L  V  D  E  F  Q  D  T  N  T  V  #  Q  Y  K  W  I  K  L  236
Cnau_S  218   I  L  V  D  E  F  Q  D  T  N  T  M  #  Q  Y  E  W  I  K  L  236
Pste_S  218   I  L  V  D  E  F  Q  D  T  N  T  V  #  Q  F  K  W  I  K  L  236
Rma     218   I  L  V  D  E  F  Q  D  T  N  T  V  #  Q  Y  K  W  I  K  L  236
Ifos_S  217   I  L  V  D  E  F  Q  D  T  N  T  V  #  Q  Y  K  W  I  K  L  235
Apha_S  182   #  #  #  #  #  #  #  #  #  #  #  #  #  #  #  #  #  #  #  #  181
Bsep_S  218   I  L  V  D  E  F  Q  D  T  N  T  V  #  Q  Y  R  W  I  Q  Q  236

E. coli          L  V  D  E  F  Q  D  T  N  N  I     Q  Y
```

**Helicase motif II**

UvrD domain (6-716)

```
Akaw_S  709 TTATTTAGTGGCAACAATAAAGTATTCTGTGTAGGAGATGATGATCAATCTATTTATGGC  768
Clau_S  709 TTATTTAGTGGCAGTAATCAAGTATTCTGTGTGGGCGATGATGACCAATCTATTTATGGC  768
Pkil_S  709 TTATTTAGTGGCAATAATAAAGTATTCTGCGTGGGTGATGATGACCAATCTATTTATGGA  768
Psoy_S  709 TTATTTAGTGGCAATAATAAAGTATTCTGCGTGGGTGATGATGACCAATCTATTTATGGA  768
Vok     709 TTATTTAGTGGCAATAATAAAGTATTCTGTGTGGGCGATGATGACCAATCTATTTATGGC  768
Cpac_S  709 TTGTTTAGTGGCAATAATAGAGTATTTTGTGTGGGTGATGACGACCAATCTATTTATAGC  768
Cfau_S  709 TTGTTTAGTGGTAATAATAGAGTATTTTGTGTGGGTGATGACGACCAATCTATTTATGGC  768
Cnau_S  709 TTGTTTGGTGGTAATAATAGAGTATTTTTTGTGGGTGATGACGACCAATCTATTTATGGC  768
Pste_S  709 TTGTTTAGTGGTAATAATAGAGTATTTTGTGTGGGCGATGACGACCAATCTATTTATGGC  768
Rma     709 TTGTTTAATGGTCATAATAAAATATTTTGTGTGGGCGATGATGATCAATCTATTTATGGA  768
Ifos_S  709 TTGTTTAGTGGCAATAATTAAGTATTTTGTGTGGGTGGTGATGACCAATCTATTTATGGC  768
Apha_S  546 ------------------------------------------------------------  545
Bsep_S  709 TTGTTCAATGGCAAAAATAATGTCTTTTGCGTTGGCGATGACGACCAATCTATTTACGGT  768
Akaw_S  237   L  F  S  G  N  N  K  V  F  C  V  G  D  D  D  Q  S  I  Y  G  256
Clau_S  237   L  F  S  G  S  N  Q  V  F  C  V  G  D  D  D  Q  S  I  Y  G  256
Pkil_S  237   L  F  S  G  N  N  K  V  F  C  V  G  D  D  D  Q  S  I  Y  G  256
Psoy_S  237   L  F  S  G  N  N  K  V  F  C  V  G  D  D  D  Q  S  I  Y  G  256
Vok     237   L  F  S  G  N  N  K  V  F  C  V  G  D  D  D  Q  S  I  Y  G  256
Cpac_S  237   L  F  S  G  N  N  R  V  F  C  V  G  D  D  D  Q  S  I  Y  S  256
Cfau_S  237   L  F  S  G  N  N  R  V  F  C  V  G  D  D  D  Q  S  I  Y  G  256
Cnau_S  237   L  F  G  G  N  N  R  V  F  F  V  G  D  D  D  Q  S  I  Y  G  256
Pste_S  237   L  F  S  G  N  N  R  V  F  C  V  G  D  D  D  Q  S  I  Y  G  256
Rma     237   L  F  N  G  H  N  K  I  F  C  V  G  D  D  D  Q  S  I  Y  G  256
Ifos_S  236   L  F  S  G  N  N  *  V  F  C  V  G  G  D  D  Q  S  I  Y  G  255
Apha_S  182   #  #  #  #  #  #  #  #  #  #  #  #  #  #  #  #  #  #  #  #  181
Bsep_S  237   L  F  N  G  K  N  N  V  F  C  V  G  D  D  D  Q  S  I  Y  G  256

E. coli                           V  M  I  V  G  D  D  D  Q  S  I  Y  G

                                          Helicase motif III
```

UvrD domain (6-716)

```
Akaw_S  769 TGGCGCGGTGCAAATATTGAAAATATTACTAAACTATGTACAGATTTTGCTCCTATTAAA  828
Clau_S  769 TGGCGTGGTGCAAATATTGAAAATATTACTAAATTATACACAGATTTTGTTCCCATTAAA  828
Pkil_S  769 TGGCGCGGTGCAAATATTGAAAATATTACTAAATTATGCACAGATTTTGTTCCCATTAAA  828
Psoy_S  769 TGGCGCGGTGCAAATATTGAAAATATTACTAAATTATGCACAGATTTTGTTCCCATTAAA  828
Vok     769 TGGCGCGGTGCAAATATTGAGAATATTACTAAATTATGCACAGATTTTGTTCCCATTAAA  828
Cpac_S  769 TGGCGCGGGGCAAAAATTGAAAATATCACCAAACTATGCACAGATTTTGCTCCCATTGAA  828
Cfau_S  769 TGGCGCGGGGCAAAAATAGAAAATATCACCAAACTATACACAGATTTTTTTCCCATTGAA  828
Cnau_S  769 TGGCGCGGGGCAAAAATTGAAAATATCACCAAACTATACACAGATTTTGCCCCCATTGAA  828
Pste_S  769 TGGCGCGGGGCAAAAATTGAAAATATCACCAAACTATGCACAGATTTTGCCCCTATTAAA  828
Rma     769 TGGCGCGGTGCAAAAATTGAAAATATTACCAAACTATGCACAGACTTTACTCCCATTGAA  828
Ifos_S  769 TGGCGCGGTGCAAAAATTGAAAATATCACCAAACTATGTACAGGTTTTACCCCTATTGAA  828
Apha_S  546 --------------------------------------------------CCCTATTGAA  555
Bsep_S  769 TGGCGTGGTGCAAAAATTGAAAATATCACCAAGTTAAGCACTGATTTTGCACCGATTCAA  828
Akaw_S  257   W  R  G  A  N  I  E  N  I  T  K  L  C  T  D  F  A  P  I  K  276
Clau_S  257   W  R  G  A  N  I  E  N  I  T  K  L  Y  T  D  F  V  P  I  K  276
Pkil_S  257   W  R  G  A  N  I  E  N  I  T  K  L  C  T  D  F  V  P  I  K  276
Psoy_S  257   W  R  G  A  N  I  E  N  I  T  K  L  C  T  D  F  V  P  I  K  276
Vok     257   W  R  G  A  N  I  E  N  I  T  K  L  C  T  D  F  V  P  I  K  276
Cpac_S  257   W  R  G  A  K  I  E  N  I  T  K  L  C  T  D  F  A  P  I  E  276
Cfau_S  257   W  R  G  A  K  I  E  N  I  T  K  L  Y  T  D  F  F  P  I  E  276
Cnau_S  257   W  R  G  A  K  I  E  N  I  T  K  L  Y  T  D  F  A  P  I  E  276
Pste_S  257   W  R  G  A  K  I  E  N  I  T  K  L  C  T  D  F  A  P  I  K  276
Rma     257   W  R  G  A  K  I  E  N  I  T  K  L  C  T  D  F  T  P  I  E  276
Ifos_S  256   W  R  G  A  K  I  E  N  I  T  K  L  C  T  G  F  T  P  I  E  275
Apha_S  182   #  #  #  #  #  #  #  #  #  #  #  #  #  #  #  #  #  P  I  E  184
Bsep_S  257   W  R  G  A  K  I  E  N  I  T  K  L  S  T  D  F  A  P  I  Q  276

E. coli       W  R  G  A

            Helicase motif III
```

UvrD domain (6-716)

```
Akaw_S  829 ACCATTCGTTTAGAACAAAATTATCGTTCAACAAATAACATTCTAACAGCTTCTAACGGC  888
Clau_S  829 ACTATCCGTTTAGAACAAAACTATCGTTCAACAAATAATATTCTAACGGCTTCTAACACC  888
Pkil_S  829 ACCATACGTTTAGAACAAAATTATCGTTCAACAAATAATATTCTAACAGCTTCTAACGCC  888
Psoy_S  829 ACCATACGTTTAGAACAAAATTATCGTTCAACAAATAATATTCTAACAGCTTCTAACGCC  888
Vok     829 ACCATCCGTTTAGAACAAAATTATCGTTCAACAAATAATATTCTAACAGCTTCTAACGCC  888
Cpac_S  829 ACCATCCACTTAGAACAAAATTATCGTTCAACTGGTAATATTCTAAACGCTTCTAACGCC  888
Cfau_S  829 ACCATCCACTTAGAACAAAATTATCGTTCAACTGGTAATATTCTAAACGCTTCTAACGCC  888
Cnau_S  829 ACCATCCGCTTAGAACAAAATTATCGTTCAACTGGTAATATTCTAAACGCCTCTAACGCC  888
Pste_S  829 ATCATCCGCTTAGAACAAAATTATCGTTCAACTGGTAATATTCTAAACGCCTCTAACGCC  888
Rma     829 ACCATCCGCTTAAAACAAAATTATCGTTCAACTGGTAATATTCTAAACGCCTCTAATGCC  888
Ifos_S  829 ACCATCCGCTTAGAACAAAATTATCGCTCAACTGGTAATATTCTAAACGCCTCTAACGTC  888
Apha_S  556 ATCATCCGCTTAGAACAAAATTATCGCTCAACTGCTAATATTCTAAACGCCTCTAACGCC  615
Bsep_S  829 ACTATTCGCCTTGAGCAAAATTATCGCTCAACAGGCTATATTCTAAAAGCCTCAAATGCA  888
Akaw_S  277   T  I  R  L  E  Q  N  Y  R  S  T  N  N  I  L  T  A  S  N  G  296
Clau_S  277   T  I  R  L  E  Q  N  Y  R  S  T  N  N  I  L  T  A  S  N  T  296
Pkil_S  277   T  I  R  L  E  Q  N  Y  R  S  T  N  N  I  L  T  A  S  N  A  296
Psoy_S  277   T  I  R  L  E  Q  N  Y  R  S  T  N  N  I  L  T  A  S  N  A  296
Vok     277   T  I  R  L  E  Q  N  Y  R  S  T  N  N  I  L  T  A  S  N  A  296
Cpac_S  277   T  I  H  L  E  Q  N  Y  R  S  T  G  N  I  L  N  A  S  N  A  296
Cfau_S  277   T  I  H  L  E  Q  N  Y  R  S  T  G  N  I  L  N  A  S  N  A  296
Cnau_S  277   T  I  R  L  E  Q  N  Y  R  S  T  G  N  I  L  N  A  S  N  A  296
Pste_S  277   I  I  R  L  E  Q  N  Y  R  S  T  G  N  I  L  N  A  S  N  A  296
Rma     277   T  I  R  L  K  Q  N  Y  R  S  T  G  N  I  L  N  A  S  N  A  296
Ifos_S  276   T  I  R  L  E  Q  N  Y  R  S  T  G  N  I  L  N  A  S  N  V  295
Apha_S  185   I  I  R  L  E  Q  N  Y  R  S  T  A  N  I  L  N  A  S  N  A  204
Bsep_S  277   T  I  R  L  E  Q  N  Y  R  S  T  G  Y  I  L  K  A  S  N  A  296

E. coli          I  R  L  E  Q  N  Y  R  S  T

                     Helicase motif IV
```

UvrD domain (6-716)

```
Akaw_S  889 TTGATTGCTCATAATGCCAGTCGTATGGGTAAATCTCTTTGGACTAATGCTAGCGATGGT  948
Clau_S  889 TTGATTGCTCATAATACCAGTCGTATGGGTAAATCTCTTTGGACTAATGCTAGCGATGGT  948
Pkil_S  889 TTAATTGCTCATAATACCAGTCGTATGGGTAAATCTCTTTGGACTAGTGCTAGCGATGGT  948
Psoy_S  889 TTAATTGCTCATAATACCAGTCGTATGGGTAAATCTCTTTGGACTAGTGCTAGCGATGGT  948
Vok     889 TTGATTGCTCATAATACCAGTCGCATGGGTAAATCTCTTTGGACTAATGCTATCGATGGT  948
Cpac_S  889 TTAATTGCCCATAACACCAATCGCATGGGCAAATCTCTTTGGACTAATGCTAGCAATGGT  948
Cfau_S  889 TTAATTGCCCATAACACCAATCGTATGGGTAAATCTCTTTGGACTGATGCTGGCAATGGT  948
Cnau_S  889 TTAATTGCCCATAACACCAATCGCATGGGTAAATCTCTTTGGACTGATGCTGGTAATGGT  948
Pste_S  889 TTAATTGCTCATAACACCAATCGTATGGATAAATCTCTTTGGACTGATTCTGACAATGGT  948
Rma     889 TTGATTACTCATAACGTCAATCGTATGGGTAAATCTCTTTGGACTGATGCTGGTAATGGT  948
Ifos_S  889 TTGATTACCCATAACGCCAATCGTATGGGTAAGTCTCTTTGGACTGATGCTGGCAATGGT  948
Apha_S  616 TTGATTGCCCATAACGCCAATCGTATGGGTAAGTCTCTTTGGACTGATGCTGGCAATGGT  675
Bsep_S  889 CTCATTGCCAATAATAAAAACCGTATGGGTAAATCACTATGGACCGATTCAGGTGATGGC  948
Akaw_S  297   L  I  A  H  N  A  S  R  M  G  K  S  L  W  T  N  A  S  D  G  316
Clau_S  297   L  I  A  H  N  T  S  R  M  G  K  S  L  W  T  N  A  S  D  G  316
Pkil_S  297   L  I  A  H  N  T  S  R  M  G  K  S  L  W  T  S  A  S  D  G  316
Psoy_S  297   L  I  A  H  N  T  S  R  M  G  K  S  L  W  T  S  A  S  D  G  316
Vok     297   L  I  A  H  N  T  S  R  M  G  K  S  L  W  T  N  A  I  D  G  316
Cpac_S  297   L  I  A  H  N  T  N  R  M  G  K  S  L  W  T  N  A  S  N  G  316
Cfau_S  297   L  I  A  H  N  T  N  R  M  G  K  S  L  W  T  D  A  G  N  G  316
Cnau_S  297   L  I  A  H  N  T  N  R  M  G  K  S  L  W  T  D  A  G  N  G  316
Pste_S  297   L  I  A  H  N  T  N  R  M  D  K  S  L  W  T  D  S  D  N  G  316
Rma     297   L  I  T  H  N  V  N  R  M  G  K  S  L  W  T  D  A  G  N  G  316
Ifos_S  296   L  I  T  H  N  A  N  R  M  G  K  S  L  W  T  D  A  G  N  G  315
Apha_S  205   L  I  A  H  N  A  N  R  M  G  K  S  L  W  T  D  A  G  N  G  224
Bsep_S  297   L  I  A  N  N  K  N  R  M  G  K  S  L  W  T  D  S  G  D  G  316
```

UvrD domain (6-716)

```
Akaw_S  949 GAATTAATTGATATATATAAGGCAAAAACAGAAATAGATGAAGCAAACTATGTTGTCAGT 1008
Clau_S  949 GAATTAATTGATATATATAAAGCGAAAACTGAAATAGATGAAGCAAACTATGTCATCAGT 1008
Pkil_S  949 GAATTAATTGATATATATGAAGCGCAAACTGAAATAGATGAAGCAGACTATGTCGTCAGT 1008
Psoy_S  949 GAATTAATTGATATATATGAAGCGCAAACTGAAATAGATGAAGCAGACTATGTCGTCAGT 1008
Vok     949 GAATTAATTGATATGTATGAGGCGCAAACTGAAATAGACGAAGCAGACTATGTCGTCAGT 1008
Cpac_S  949 GAATTGATTGATATTTATGAAGCACGAACTGAAACAGATGAGGCAGATTATGTCATCAGC 1008
Cfau_S  949 GAATTGATTGATGTTTATAAAGCACGAACTGAAACAGATGAGGCAGATTATGTCATCAGC 1008
Cnau_S  949 AAATTGATTGATGTTTATGAAGCACGAACTGAAACAAATGAGGCAGATTATGTCATCAGC 1008
Pste_S  949 GAATTGATTGATGTTTATGAAGCACGAACTGAAACAGATGAGGCGGATTATGTCATTAGC 1008
Rma     949 GAATTGATTGATGTCTATGAAGCGCGAACTGAAACGGATGAGGCGAACTATGTTATCAGC 1008
Ifos_S  949 GAATTGATTGATGCCTATGAAGCGCGAACTGAAACAGATGAGGCAGATTATGTCATCAGT 1008
Apha_S  676 GAATTGATTGATGTCTATGAAGCGCAAACTGAAACAGATGAGGTAGACTATGTCACCAGT  735
Bsep_S  949 GATTTGATTGATTTATACGAAGCACGCACCGAAATAGACGAGGCTGATTATGTAGTTGGC 1008
Akaw_S  317   E  L  I  D  I  Y  K  A  K  T  E  I  D  E  A  N  Y  V  V  S  336
Clau_S  317   E  L  I  D  I  Y  K  A  K  T  E  I  D  E  A  N  Y  V  I  S  336
Pkil_S  317   E  L  I  D  I  Y  E  A  Q  T  E  I  D  E  A  D  Y  V  V  S  336
Psoy_S  317   E  L  I  D  I  Y  E  A  Q  T  E  I  D  E  A  D  Y  V  V  S  336
Vok     317   E  L  I  D  M  Y  E  A  Q  T  E  I  D  E  A  D  Y  V  V  S  336
Cpac_S  317   E  L  I  D  I  Y  E  A  R  T  E  T  D  E  A  D  Y  V  I  S  336
Cfau_S  317   E  L  I  D  V  Y  K  A  R  T  E  T  D  E  A  D  Y  V  I  S  336
Cnau_S  317   K  L  I  D  V  Y  E  A  R  T  E  T  N  E  A  D  Y  V  I  S  336
Pste_S  317   E  L  I  D  V  Y  E  A  R  T  E  T  D  E  A  D  Y  V  I  S  336
Rma     317   E  L  I  D  V  Y  E  A  R  T  E  T  D  E  A  N  Y  V  I  S  336
Ifos_S  316   E  L  I  D  A  Y  E  A  R  T  E  T  D  E  A  D  Y  V  I  S  335
Apha_S  225   E  L  I  D  V  Y  E  A  Q  T  E  T  D  E  V  D  Y  V  T  S  244
Bsep_S  317   D  L  I  D  L  Y  E  A  R  T  E  I  D  E  A  D  Y  V  V  G  336
```

UvrD domain (6-716)

```
Akaw_S 1009 AGCATTCAGAAATTAATCACTAATGGCTCATTACCCAATGATTGCGCTATTCTATATCGC 1068
Clau_S 1009 AGCATTCAGAAATTAATCACTAATGGCTCATTACCCAATGATTGTGCTATTCTATATCGC 1068
Pkil_S 1009 AGTATTCAGAAATTAATCGCTAATGGCGCATTACCCAATGATTGTGCTATTCTGTATCGC 1068
Psoy_S 1009 AGTATTCAGAAATTAATCGCTAATGGCGCATTACCCAATGATTGTGCTATTCTGTATCGC 1068
Vok    1009 AGTATTCAGAAATTAATCACTAATGGCGTATTACCCAATGATTGTGCTATTCTGTATCGC 1068
Cpac_S 1009 AGTATTCAAAAATTGATCACTGATAATGCATCACCTAGTGATTGTGCCATTTTATATCGG 1068
Cfau_S 1009 AGTATTCAAAAATTAATCACTGATGATGCATCACCTAGTGATTGTGCCATTTTGTATCGG 1068
Cnau_S 1009 AGTATTCAAAAATTAATTACCGATGATGCATCACCTAGTGATTGTGCCATTTTGTATCGG 1068
Pste_S 1009 AGTATTCAAAAATTAATCACTGATGATGTATCACCTAGTGATTGTGCCATTTTGTATCGG 1068
Rma    1009 AATATTCAAAAATTAATCACTAATGGTGCATCGCCCAGTGATTGCTCTATTTTGTACCGA 1068
Ifos_S 1009 AGTATTCAAAAATTAATCACTGATGGTGCATCACCTAGTGATTGTGCTATTTTGTATCGC 1068
Apha_S  736 AGTATTCAAAAATTAATCACTGATGGTGCATCACCTAGTGATTGCTCGATTTTGTACCGG  795
Bsep_S 1009 AGTATTCAAAAAATCATTAATAATGGCATTCCTGCTAATCAATGTGCTATTCTTTATCGT 1068
Akaw_S  337   S  I  Q  K  L  I  T  N  G  S  L  P  N  D  C  A  I  L  Y  R  356
Clau_S  337   S  I  Q  K  L  I  T  N  G  S  L  P  N  D  C  A  I  L  Y  R  356
Pkil_S  337   S  I  Q  K  L  I  A  N  G  A  L  P  N  D  C  A  I  L  Y  R  356
Psoy_S  337   S  I  Q  K  L  I  A  N  G  A  L  P  N  D  C  A  I  L  Y  R  356
Vok     337   S  I  Q  K  L  I  T  N  G  V  L  P  N  D  C  A  I  L  Y  R  356
Cpac_S  337   S  I  Q  K  L  I  T  D  N  A  S  P  S  D  C  A  I  L  Y  R  356
Cfau_S  337   S  I  Q  K  L  I  T  D  D  A  S  P  S  D  C  A  I  L  Y  R  356
Cnau_S  337   S  I  Q  K  L  I  T  D  D  A  S  P  S  D  C  A  I  L  Y  R  356
Pste_S  337   S  I  Q  K  L  I  T  D  D  V  S  P  S  D  C  A  I  L  Y  R  356
Rma     337   N  I  Q  K  L  I  T  N  G  A  S  P  S  D  C  S  I  L  Y  R  356
Ifos_S  336   S  I  Q  K  L  I  T  D  G  A  S  P  S  D  C  A  I  L  Y  R  355
Apha_S  245   S  I  Q  K  L  I  T  D  G  A  S  P  S  D  C  S  I  L  Y  R  264
Bsep_S  337   S  I  Q  K  I  I  N  N  G  I  P  A  N  Q  C  A  I  L  Y  R  356
```

UvrD domain (6-716)

```
Akaw_S 1069 CTTAATACCCAATCTCGTATCTTTGAAGAGGTGCTCATAAAATACAATATTCCTTATATT 1128
Clau_S 1069 CTTAATACCCAATCTCGTATCTTTGAAGAGGAGCTCATAAAATACAATATTCCTTATATT 1128
Pkil_S 1069 CTTAATACCCAATCTCGTATCTTTGAAGAGGCGCTCATAAAATACAATATTCCTTATATT 1128
Psoy_S 1069 CTTAATACCCAATCTCGTATCTTTGAAGAGGCGCTCATAAAATACAATATTCCTTATATT 1128
Vok    1069 CTTAATACCCAATCTCGTATCTTTGAAGAGGTTCTCATAAAATACAATATTCCTTATATT 1128
Cpac_S 1069 TCTAATGCTCAATCTCGCATCTTTGAAGAAGCGCTAATAAAATACAACATTCCTTATATT 1128
Cfau_S 1069 TCTAATGCTCAATCTCGCATCTTTGAAGAAGCACTAATAAAATACAACATTCCTTATATT 1128
Cnau_S 1069 TCTAATGCTCAATCTCGCATCTTTGAAGATGCGCTAATAAGATACAACATTCCTTATATT 1128
Pste_S 1069 TCCAATGCTCAATCTCGCATCTTTGAAGAAGCGCTAATAAAATGCAACATTCCTTATATT 1128
Rma    1069 TTTAATGCTCAATCTCGTATCTTTGAAGAGGCACTAATAAAATACAATATTCCTTATATT 1128
Ifos_S 1069 TCCAATGCCCAATCTCGTAACTTTGAAGAAGCGCTCATAAAATACAACATTCCTTATATT 1128
Apha_S  796 TCTAATGCTCAATCTCGCATCTTTAAAGAGGCGCTAATAAAATACAACATTCCTTATATT  855
Bsep_S 1069 TCCAATGCACAATCTAGGGCCTTTGAAGAGCGCTTAATTAAATACAATGTACCTTATATT 1128
Akaw_S  357   L  N  T  Q  S  R  I  F  E  E  V  L  I  K  Y  N  I  P  Y  I  376
Clau_S  357   L  N  T  Q  S  R  I  F  E  E  E  L  I  K  Y  N  I  P  Y  I  376
Pkil_S  357   L  N  T  Q  S  R  I  F  E  E  A  L  I  K  Y  N  I  P  Y  I  376
Psoy_S  357   L  N  T  Q  S  R  I  F  E  E  A  L  I  K  Y  N  I  P  Y  I  376
Vok     357   L  N  T  Q  S  R  I  F  E  E  V  L  I  K  Y  N  I  P  Y  I  376
Cpac_S  357   S  N  A  Q  S  R  I  F  E  E  A  L  I  K  Y  N  I  P  Y  I  376
Cfau_S  357   S  N  A  Q  S  R  I  F  E  E  A  L  I  K  Y  N  I  P  Y  I  376
Cnau_S  357   S  N  A  Q  S  R  I  F  E  D  A  L  I  R  Y  N  I  P  Y  I  376
Pste_S  357   S  N  A  Q  S  R  I  F  E  E  A  L  I  K  C  N  I  P  Y  I  376
Rma     357   F  N  A  Q  S  R  I  F  E  E  A  L  I  K  Y  N  I  P  Y  I  376
Ifos_S  356   S  N  A  Q  S  R  N  F  E  E  A  L  I  K  Y  N  I  P  Y  I  375
Apha_S  265   S  N  A  Q  S  R  I  F  K  E  A  L  I  K  Y  N  I  P  Y  I  284
Bsep_S  357   S  N  A  Q  S  R  A  F  E  E  R  L  I  K  Y  N  V  P  Y  I  376
```

UvrD domain (6-716)

```
Akaw_S 1129 ATTTATGGTGGTTTAAAATTTTTTGAACGTGCTGAAATTAAATACGCTTTAAGTTATTTG 1188
Clau_S 1129 ATTTATGGTGGTTTAAAATTTTTTGAACGTGCTGAAATTAAATACGCTTTAAGTTATTTA 1188
Pkil_S 1129 ATTTATGGTGGTTTAAAATTTTTTGAACGTGCTGAAATTAAACACGCTTTAAGTTATTTA 1188
Psoy_S 1129 ATTTATGGTGGTTTAAAATTTTTTGAACGTGCTGAAATTAAACACGCTTTAAGTTATTTA 1188
Vok    1129 ATTTATGGTGGTTTAAAATTTTTTGAACGTGCTGAAATTAAACACGCTTTAAGTTATTTA 1188
Cpac_S 1129 ATCTATGGTGGTTTAAGGTTTTTAGAGCGTGCTGAGATTAAAGATGCTTTAAGCTATCTG 1188
Cfau_S 1129 ATCTATGGTGGTTTAAGGTTTTTAGAGCGTGCTGAGATTAAAGACGCTTTAGGCTATCTG 1188
Cnau_S 1129 ATCTATGGTGGTTTAAGGTTTTTCGAGCGTGCTGAAATTAAATACGCTTTAGGCTATCTG 1188
Pste_S 1129 ATCTATGGTGGTTTAAGGTTTTTCGAGCGTGCTGAGATTAAAGAAGCTTTAGGCTATCTG 1188
Rma    1129 ATCTATGGTGGTTTAAAATTTTTTGAGTATACCGAGATTAAAGACGCTTTAAGTTATTTA 1188
Ifos_S 1129 ATCTATGGTGGTTTTAGGTTTTTCGAGCGTGCTGAGATTAAAGATGCTTTAAGTTATTTG 1188
Apha_S  856 ATCTATGGCAGTTTAAGATTTTTCGAGCATTCTGAGATTAAAGACGCTTTAGGCTATTTG  915
Bsep_S 1129 ATCTACGGCGGTTTGCGATTTTTTGAGCGCGCGGAAATTAAAGATGCTTTGTGTTATTTG 1188
Akaw_S  377   I  Y  G  G  L  K  F  F  E  R  A  E  I  K  Y  A  L  S  Y  L  396
Clau_S  377   I  Y  G  G  L  K  F  F  E  R  A  E  I  K  Y  A  L  S  Y  L  396
Pkil_S  377   I  Y  G  G  L  K  F  F  E  R  A  E  I  K  H  A  L  S  Y  L  396
Psoy_S  377   I  Y  G  G  L  K  F  F  E  R  A  E  I  K  H  A  L  S  Y  L  396
Vok     377   I  Y  G  G  L  K  F  F  E  R  A  E  I  K  H  A  L  S  Y  L  396
Cpac_S  377   I  Y  G  G  L  R  F  L  E  R  A  E  I  K  D  A  L  S  Y  L  396
Cfau_S  377   I  Y  G  G  L  R  F  L  E  R  A  E  I  K  D  A  L  G  Y  L  396
Cnau_S  377   I  Y  G  G  L  R  F  F  E  R  A  E  I  K  Y  A  L  G  Y  L  396
Pste_S  377   I  Y  G  G  L  R  F  F  E  R  A  E  I  K  E  A  L  G  Y  L  396
Rma     377   I  Y  G  G  L  K  F  F  E  Y  T  E  I  K  D  A  L  S  Y  L  396
Ifos_S  376   I  Y  G  G  F  R  F  F  E  R  A  E  I  K  D  A  L  S  Y  L  395
Apha_S  285   I  Y  G  S  L  R  F  F  E  H  S  E  I  K  D  A  L  G  Y  L  304
Bsep_S  377   I  Y  G  G  L  R  F  F  E  R  A  E  I  K  D  A  L  C  Y  L  396
```

UvrD domain (6-716)

```
Akaw_S 1189 CGTTTGATAGAAAACTCAGATGATAATCTTGCCTTTGAACGTGTGGTTAATTTCCCAACT 1248
Clau_S 1189 CGTTTAATAGAAAACTCAGATGATAATCTTGCCTTTGAACGGGTGGTTAATTTTCCAACT 1248
Pkil_S 1189 CGTTTGATAGAAAACTCAGATGATAATCTTGCCTTTGAACGTGTGGTTAATTTTCCAACT 1248
Psoy_S 1189 CGTTTGATAGAAAACTCAGATGATAATCTTGCCTTTGAACGTGTGGTTAATTTTCCAACT 1248
Vok    1189 CGTTTGATAGAAAACTCAGATGATAATCTTGCCTTTGAACGTGTGGTTAATTTTCCAACT 1248
Cpac_S 1189 CGTTTGATAGAAAATTCAGCTGACAATGTTGCCTTTGAGCGTGTGGTTAATTCTCCAACT 1248
Cfau_S 1189 CGTTTGATAGAAAATTCAGCTGACAATGTTGCCTTTGAGCGTGTGGTTAATTTTCCAACT 1248
Cnau_S 1189 CGTTTGATAGAAAATTCAGCTGACAATGTTGCCTTTGATCGTGTGGTTAATTTCCCAACT 1248
Pste_S 1189 CGTTTGATAGAAAATTCAGCTGACAATGTTGCCTTTGAGCGTGTGGTTAATTTCCCAACT 1248
Rma    1189 CGTTTAATAGAGAACTCGGCTGATAATGTTGCCTTTGAACGCGTAGTTAATTTTCCAACT 1248
Ifos_S 1189 CGTTTGATAGAAAATTCTGCTGACAATGTTGCCTTTGAACGTGTGATTAATTTCCCAACT 1248
Apha_S  916 CGTTTGATAGAAAATTCAGCTGATAATGTTGCTTTTGAGCGTGTAGTTAATTTCCAGACT  975
Bsep_S 1189 CGCTTAATGGAAAATACCGAAGACAGTGTTGCTTTTGAGCGCATCGTCAATTTCCCAACG 1248
Akaw_S  397  R  L  I  E  N  S  D  D  N  L  A  F  E  R  V  V  N  F  P  T  416
Clau_S  397  R  L  I  E  N  S  D  D  N  L  A  F  E  R  V  V  N  F  P  T  416
Pkil_S  397  R  L  I  E  N  S  D  D  N  L  A  F  E  R  V  V  N  F  P  T  416
Psoy_S  397  R  L  I  E  N  S  D  D  N  L  A  F  E  R  V  V  N  F  P  T  416
Vok     397  R  L  I  E  N  S  D  D  N  L  A  F  E  R  V  V  N  F  P  T  416
Cpac_S  397  R  L  I  E  N  S  A  D  N  V  A  F  E  R  V  V  N  S  P  T  416
Cfau_S  397  R  L  I  E  N  S  A  D  N  V  A  F  E  R  V  V  N  F  P  T  416
Cnau_S  397  R  L  I  E  N  S  A  D  N  V  A  F  D  R  V  V  N  F  P  T  416
Pste_S  397  R  L  I  E  N  S  A  D  N  V  A  F  E  R  V  V  N  F  P  T  416
Rma     397  R  L  I  E  N  S  A  D  N  V  A  F  E  R  V  V  N  F  P  T  416
Ifos_S  396  R  L  I  E  N  S  A  D  N  V  A  F  E  R  V  I  N  F  P  T  415
Apha_S  305  R  L  I  E  N  S  A  D  N  V  A  F  E  R  V  V  N  F  Q  T  324
Bsep_S  397  R  L  M  E  N  T  E  D  S  V  A  F  E  R  I  V  N  F  P  T  416
```

UvrD domain (6-716)

```
Akaw_S 1249 CGTGGTATTGGAAATAAAACAGTTAACAAAATACATACATTTGCACAAAATAACCGTGTC 1308
Clau_S 1249 CGTGGTATTGGAAATAAAACAGTTAACAAAATACGTACATTTGCACAAAATAATCATGTC 1308
Pkil_S 1249 CGTGGTATTGGAAATACAACAATTAACAAAATACGTATATTTGCACAAAATAACCATGTC 1308
Psoy_S 1249 CGTGGTATTGGAAATACAACAATTAACAAAATACGTATATTTGCACAAAATAACCATGTC 1308
Vok    1249 CGTGGTATTGGAAATACAACAATTAACAAAATACGTATATTTGCACAAAATAACCATGTC 1308
Cpac_S 1249 CGCGGTATTGGCAATGTAACGGTTGAAAAAATACGCACATTTGCACAAGACAACCACACC 1308
Cfau_S 1249 CGCGGTATTGGAAATACAACAGTTGAAAAAATACGCACATTTGCACAAGACAACCACACC 1308
Cnau_S 1249 CGCGGTATTGGCAATGCAACAGTTGAAAAAATACGCACATTTTCACAAAACAACTACACC 1308
Pste_S 1249 CGCGGTATTGGCAATATAACGGTTGAAAAAATACGTACATTTGCACAAGACAACCACACC 1308
Rma    1249 CGCGGTATTGGCAATGTAACGGTTAAAAAAATACGTACATTTGCACAAGACAACCACACC 1308
Ifos_S 1249 CGCGGTATTGGCAATGCAACGGTTGAAAAAATACGCACCTTTGCACAAGATAACCACACC 1308
Apha_S  976 CGCAGTATTGGCAATGCAACGGTTGAAAAAATACGCACATTTACACAAGACAACCATACC 1035
Bsep_S 1249 CGAGGTATTGGCGCTGCCACCATCGAAAAAGTGCGAGAGCATGCGTTAATGAACCAAACA 1308
Akaw_S  417  R  G  I  G  N  K  T  V  N  K  I  H  T  F  A  Q  N  N  R  V  436
Clau_S  417  R  G  I  G  N  K  T  V  N  K  I  R  T  F  A  Q  N  N  H  V  436
Pkil_S  417  R  G  I  G  N  T  T  I  N  K  I  R  I  F  A  Q  N  N  H  V  436
Psoy_S  417  R  G  I  G  N  T  T  I  N  K  I  R  I  F  A  Q  N  N  H  V  436
Vok     417  R  G  I  G  N  T  T  I  N  K  I  R  I  F  A  Q  N  N  H  V  436
Cpac_S  417  R  G  I  G  N  V  T  V  E  K  I  R  T  F  A  Q  D  N  H  T  436
Cfau_S  417  R  G  I  G  N  T  T  V  E  K  I  R  T  F  A  Q  D  N  H  T  436
Cnau_S  417  R  G  I  G  N  A  T  V  E  K  I  R  T  F  S  Q  N  N  Y  T  436
Pste_S  417  R  G  I  G  N  I  T  V  E  K  I  R  T  F  A  Q  D  N  H  T  436
Rma     417  R  G  I  G  N  V  T  V  K  K  I  R  T  F  A  Q  D  N  H  T  436
Ifos_S  416  R  G  I  G  N  A  T  V  E  K  I  R  T  F  A  Q  D  N  H  T  435
Apha_S  325  R  S  I  G  N  A  T  V  E  K  I  R  T  F  T  Q  D  N  H  T  344
Bsep_S  417  R  G  I  G  A  A  T  I  E  K  V  R  E  H  A  L  M  N  Q  T  436
```

UvrD domain (6-716)

```
Akaw_S 1309 AGTCTTTTTCAAGCAACCATACAAATATCATCTACACTATCAACTCGTACTGCGAATGCA 1368
Clau_S 1309 AGTCTTTTTCAATCAACTATACAAGTATCATCTACATTATCAACTCGTACTGCAAATGCA 1368
Pkil_S 1309 AGTCTTTTTCAAGCAACCATACAAGTATCATCTACACTGTCAACTCGTACTACTAATGCA 1368
Psoy_S 1309 AGTCTTTTTCAAGCAACCATACAAGTATCATCTACACTGTCAACTCGTACTACTAATGCA 1368
Vok    1309 AGTCTTTTTCAAGCAACCATACAAGTATCATCTACACTGTCAACTCGTACTACTAATGCA 1368
Cpac_S 1309 AGTCTTTTTCAAGCAGCCATACAAATATCATCCACCCTACCAACTCGTTCTGCCAACGCA 1368
Cfau_S 1309 AGTCTTTTTCAAGCAGCCATACAAATATCATCCACCCTACCAACTCGTTCTGCTAACGCA 1368
Cnau_S 1309 AGTCTTTTTCAAGCAGCCATACAAATATCATCCACCTTACCAACTCGTTCTGCCAACGCA 1368
Pste_S 1309 AGTCTTTTTCAAGCAGCCATACAAATATCATCCATCCTATCAACTCGTACTGCCAACGCA 1368
Rma    1309 AGTCTTTTTCAAGCAGCCATACAAATATCATCCATCCTGCCAACTCGCGCTGCCAATGCA 1368
Ifos_S 1309 AGTCTTTTTCAAGCAGCCATACAAATGTCATCCACCCTACCAACTCGCGCTGCCAATGCA 1368
Apha_S 1036 AGTCTTTTTCAAGCAGCCATACAAATATCATCCACCCTACCAACTCTTGCTGCCAATGTA 1095
Bsep_S 1309 GGCTTATTTCAAGCGGCTATCAGTATGGCACCTACTTTACCAACCCGCGCTGCCAATGCC 1368
Akaw_S  437   S  L  F  Q  A  T  I  Q  I  S  S  T  L  S  T  R  T  A  N  A  456
Clau_S  437   S  L  F  Q  S  T  I  Q  V  S  S  T  L  S  T  R  T  A  N  A  456
Pkil_S  437   S  L  F  Q  A  T  I  Q  V  S  S  T  L  S  T  R  T  T  N  A  456
Psoy_S  437   S  L  F  Q  A  T  I  Q  V  S  S  T  L  S  T  R  T  T  N  A  456
Vok     437   S  L  F  Q  A  T  I  Q  V  S  S  T  L  S  T  R  T  T  N  A  456
Cpac_S  437   S  L  F  Q  A  A  I  Q  I  S  S  T  L  P  T  R  S  A  N  A  456
Cfau_S  437   S  L  F  Q  A  A  I  Q  I  S  S  T  L  P  T  R  S  A  N  A  456
Cnau_S  437   S  L  F  Q  A  A  I  Q  I  S  S  T  L  P  T  R  S  A  N  A  456
Pste_S  437   S  L  F  Q  A  A  I  Q  I  S  S  I  L  S  T  R  T  A  N  A  456
Rma     437   S  L  F  Q  A  A  I  Q  I  S  S  I  L  P  T  R  A  A  N  A  456
Ifos_S  436   S  L  F  Q  A  A  I  Q  M  S  S  T  L  P  T  R  A  A  N  A  455
Apha_S  345   S  L  F  Q  A  A  I  Q  I  S  S  T  L  P  T  L  A  A  N  V  364
Bsep_S  437   G  L  F  Q  A  A  I  S  M  A  P  T  L  P  T  R  A  A  N  A  456
```

UvrD domain (6-716)

```
Akaw_S 1369 TTAAATGTATTTACCAACCTAATTAAGAAAATAAAAGATGATACTAAAAACTTAGATTTA 1428
Clau_S 1369 TTAAATGGATTTACCAACCTAATTGAGAAAATGAAAAATGATACAAAGAACTTAGATTTA 1428
Pkil_S 1369 TTAAATGGATTTACCAACCTAATTAAGAAAATGAAAGATGATACAAAGAACTTAGATTTA 1428
Psoy_S 1369 TTAAATGGATTTACCAACCTAATTAAGAAAATGAAAGATGATACAAAGAACTTAGATTTA 1428
Vok    1369 TTAAATGGATTTACCAACCTAATTGAGAAAATGAAAGATGATACAAAAAACTTAAATTTA 1428
Cpac_S 1369 TTAAATGGATTTACCAAGCTAATTGAGAAAATGATAGATGAGACAAAGTACCTAGGTTTA 1428
Cfau_S 1369 TTAAATGGATTTATCAAGCTAATTGAGCAAATGACAGATAACACAAAGTACCTAGGTTTA 1428
Cnau_S 1369 TTAACTGGATTTACCAACCTAATTGAACAAATGACAGATGACACAAAGCACCTAGATTTA 1428
Pste_S 1369 TTAAATGGATTTACCAACCTAATTGAGCAAATGACAGATGACACAAAGCACCTAGATTTA 1428
Rma    1369 TTAAGTAGCTTTATTAATCTAATTAAGCAAATAACAGACGACACAAAGCACCTAAATTTA 1428
Ifos_S 1369 TTAAGTAGCTTTGTTAACCTAATTGAGCAAATGACAGACGACACAAAGAACCTAGATTTA 1428
Apha_S 1096 TTAAATGGATTTACCAACCTAATTGAACAAATGACAGATGACACAAAGCACCTAGATTTA 1155
Bsep_S 1369 TTAATCGGATTTATGCAATTGATTGAAGAAATGCAAGACCATGCCCAGAATTTGGATTTA 1428
Akaw_S  457   L  N  V  F  T  N  L  I  K  K  I  K  D  D  T  K  N  L  D  L  476
Clau_S  457   L  N  G  F  T  N  L  I  E  K  M  K  N  D  T  K  N  L  D  L  476
Pkil_S  457   L  N  G  F  T  N  L  I  K  K  M  K  D  D  T  K  N  L  D  L  476
Psoy_S  457   L  N  G  F  T  N  L  I  K  K  M  K  D  D  T  K  N  L  D  L  476
Vok     457   L  N  G  F  T  N  L  I  E  K  M  K  D  D  T  K  N  L  N  L  476
Cpac_S  457   L  N  G  F  T  K  L  I  E  K  M  I  D  E  T  K  Y  L  G  L  476
Cfau_S  457   L  N  G  F  I  K  L  I  E  Q  M  T  D  N  T  K  Y  L  G  L  476
Cnau_S  457   L  T  G  F  T  N  L  I  E  Q  M  T  D  D  T  K  H  L  D  L  476
Pste_S  457   L  N  G  F  T  N  L  I  E  Q  M  T  D  D  T  K  H  L  D  L  476
Rma     457   L  S  S  F  I  N  L  I  K  Q  I  T  D  D  T  K  H  L  N  L  476
Ifos_S  456   L  S  S  F  V  N  L  I  E  Q  M  T  D  D  T  K  N  L  D  L  475
Apha_S  365   L  N  G  F  T  N  L  I  E  Q  M  T  D  D  T  K  H  L  D  L  384
Bsep_S  457   L  I  G  F  M  Q  L  I  E  E  M  Q  D  H  A  Q  N  L  D  L  476
```

UvrD domain (6-716)

```
Akaw_S 1429 TCTGAAAAAATAGTAAACTTACTTAATATATCAGGATTAATGGCGCATTATTCTAATGAT 1488
Clau_S 1429 TCTGAAAAAATAGTAAACTTACTTAATATATCAGGATTAATGGCGCATTATTCTAATGAT 1488
Pkil_S 1429 TCTGAAAAAATAGTAAACTTACTTAATATATCAGGATTAATGGCGCATTATTCTAATGAT 1488
Psoy_S 1429 TCTGAAAAAATAGTAAACTTACTTAATATATCAGGATTAATGGCGCATTATTCTAATGAT 1488
Vok    1429 TCTGAAAAAATAGTAAACTTACTTAATATGTCAGGATTAATGGCGCATTATTCTAATGAT 1488
Cpac_S 1429 TCTGAAAAAGTAGCCAATTTGCTTAACACATCAGGATTAATGGCGCATTATTCTAATGAT 1488
Cfau_S 1429 TCTGAAAAAGTAGCCAACTTGCTTAACACATCAGGATTAATGGTGCATTATTCTAATGAC 1488
Cnau_S 1429 TCTGAAAAAGTAGCTAACTTGCTTAACACATCAGGATTAATGGCGCATTATTCTAATGAC 1488
Pste_S 1429 TCTGAAAAAGTAGCCAACTTGCTTAACACATCAGGATTAATGGCTCATTATTCTAATAAC 1488
Rma    1429 TCTGAAAAAGTAACTAACTTGCTTAATACATCAGGATTAATTATGCATTATTCTAATGAT 1488
Ifos_S 1429 TCTGAAGAAGTAGCCAACTTGCTTAATACATCAGAATTAATGATGCATTATTCTAATGAT 1488
Apha_S 1156 TCTGAAAAAGTAGCTAACTTGCTTAACACATCAGGATTAATGATGCATTATTCTAATGAT 1215
Bsep_S 1429 TCTGAAAAAATTGGCTATTTAATTAAAAAATCAGGCTTATTTGACTATTATTCCAACGAT 1488
Akaw_S  477   S  E  K  I  V  N  L  L  N  I  S  G  L  M  A  H  Y  S  N  D  496
Clau_S  477   S  E  K  I  V  N  L  L  N  I  S  G  L  M  A  H  Y  S  N  D  496
Pkil_S  477   S  E  K  I  V  N  L  L  N  I  S  G  L  M  A  H  Y  S  N  D  496
Psoy_S  477   S  E  K  I  V  N  L  L  N  I  S  G  L  M  A  H  Y  S  N  D  496
Vok     477   S  E  K  I  V  N  L  L  N  M  S  G  L  M  A  H  Y  S  N  D  496
Cpac_S  477   S  E  K  V  A  N  L  L  N  T  S  G  L  M  A  H  Y  S  N  D  496
Cfau_S  477   S  E  K  V  A  N  L  L  N  T  S  G  L  M  V  H  Y  S  N  D  496
Cnau_S  477   S  E  K  V  A  N  L  L  N  T  S  G  L  M  A  H  Y  S  N  D  496
Pste_S  477   S  E  K  V  A  N  L  L  N  T  S  G  L  M  A  H  Y  S  N  N  496
Rma     477   S  E  K  V  T  N  L  L  N  T  S  G  L  I  M  H  Y  S  N  D  496
Ifos_S  476   S  E  E  V  A  N  L  L  N  T  S  E  L  M  M  H  Y  S  N  D  495
Apha_S  385   S  E  K  V  A  N  L  L  N  T  S  G  L  M  M  H  Y  S  N  D  404
Bsep_S  477   S  E  K  I  G  Y  L  I  K  K  S  G  L  F  D  Y  Y  S  N  D  496
```

UvrD domain (6-716)

```
Akaw_S 1489 AAAACTAGCAAC---------AAAAAAGAAAATCTAGAAGAGTTAATA---ACTGCAGCT 1536
Clau_S 1489 AAAACTAGCAAC---------AAAAAAGAAAATCTAAAAGAATTAATA---ACTGCAGCT 1536
Pkil_S 1489 AAAACTAGCAAC---------AAAAAAGAAAATCTAGAAGAATTAATA---ACTGCAGCT 1536
Psoy_S 1489 AAAACTAGCAAC---------AAAAAAGAAAATCTAGAAGAATTAATA---ACTGCAGCT 1536
Vok    1489 AAAACTAGCAAC---------AAAAAAGAAAATCTAGAAGAATTAATA---ACTGCGGCT 1536
Cpac_S 1489 AAAACTGATAAAGCTGGTAGCAAGAAAGAAAATCTAAAAGAGTTAATC---ACCGCAGCG 1545
Cfau_S 1489 AAAACTGATAAAGCTGGTAGCAAGAAAGAAAATCTAGAAGAGCTAATC---ACCGCAGCG 1545
Cnau_S 1489 AAAACTGATAAAGCTGGTAGCAAGAAAGAAAATCTAGAAAAGCTAATAATCACCGCAGCA 1548
Pste_S 1489 AAAACTGATAAAACTGGTAGCAAGAAAGAAAATCTAGAAGAGCTAATT---ACCACAGCG 1545
Rma    1489 AAAATCGGCAGT---------AAAAAAGAAAATCTAAAAAAGTTAATC---ACCATAACG 1536
Ifos_S 1489 AAAACCGATAAGGCTGGCAGTAAAAAAGAAAATCTAGAAGAACTAATC---ACCGCAGCG 1545
Apha_S 1216 AAAACCGATAAGGCCGGCAGTAAAAAAGAAAATCTAGAAGAGTTAATC---ACCGCAGCA 1272
Bsep_S 1489 AAAACCGATAAAGCTGGCAGCAAACCCGCCAACCTTGAAGAACTCATT---GCCGCTGCC 1545
Akaw_S  497   K  T  S  N  #  #  #  K  K  E  N  L  E  E  L  I  #  T  A  A  512
Clau_S  497   K  T  S  N  #  #  #  K  K  E  N  L  K  E  L  I  #  T  A  A  512
Pkil_S  497   K  T  S  N  #  #  #  K  K  E  N  L  E  E  L  I  #  T  A  A  512
Psoy_S  497   K  T  S  N  #  #  #  K  K  E  N  L  E  E  L  I  #  T  A  A  512
Vok     497   K  T  S  N  #  #  #  K  K  E  N  L  E  E  L  I  #  T  A  A  512
Cpac_S  497   K  T  D  K  A  G  S  K  K  E  N  L  K  E  L  I  #  T  A  A  515
Cfau_S  497   K  T  D  K  A  G  S  K  K  E  N  L  E  E  L  I  #  T  A  A  515
Cnau_S  497   K  T  D  K  A  G  S  K  K  E  N  L  E  K  L  I  I  T  A  A  516
Pste_S  497   K  T  D  K  T  G  S  K  K  E  N  L  E  E  L  I  #  T  T  A  515
Rma     497   K  I  G  S  #  #  #  K  K  E  N  L  K  K  L  I  #  T  I  T  512
Ifos_S  496   K  T  D  K  A  G  S  K  K  E  N  L  E  E  L  I  #  T  A  A  514
Apha_S  405   K  T  D  K  A  G  S  K  K  E  N  L  E  E  L  I  #  T  A  A  423
Bsep_S  497   K  T  D  K  A  G  S  K  P  A  N  L  E  E  L  I  #  A  A  A  515
```

UvrD domain (6-716)

```
Akaw_S 1537 AAACAATATAAA---------------CAAAATAATGATATGAATGAAGTAATAGGATTT 1581
Clau_S 1537 AAACAATATAAA---------------CAGAATAATAATATGAATGAAGTAATAGGATTT 1581
Pkil_S 1537 AAACAATATAAA---------------CAAAATAATGATATGAATGAAGTAATAGGATTT 1581
Psoy_S 1537 AAACAATATAAA---------------CAAAATAATGATATGAATGAAGTAATAGGATTT 1581
Vok    1537 AAACAATATAAA---------------CAGAACAATGATATGAATGAAATAATAGGATTT 1581
Cpac_S 1546 CAACAATATAACCAT---------GAACAAGATAGCGAGATGAATGAAGTGCTAGGATTT 1596
Cfau_S 1546 CAACAATATAACCAT---------GAACAAGATAGCGAGATGAACGAAGTGGTAGGATTT 1596
Cnau_S 1549 CAACAATATAACCAT---------GAACAAGATAGCGAGATGAACGAAGTGGTAGGATTT 1599
Pste_S 1546 CAACAATATAACCAT---------GAACAAGATAGCGAGATGAACGAAGTGGTAGGATTT 1596
Rma    1537 AAACAATACAACCAT---------GAACAAGATAATAAGATGAGCGAAGTTATGGGATTT 1587
Ifos_S 1546 CAACAATACAACCAT---------GAACAAGATAGCGGGATGAACGAAGTGGTGGGGTTT 1596
Apha_S 1273 CAACCATACAACCATACAACCAT-GAACAAGATAGCGAGATGAACGAGGTGGTGGGGTTT 1331
Bsep_S 1546 GAACAATACACACAT---------GAAGAAGACAGTGAAATGAACGAAGTTACAGGCTTC 1596
Akaw_S  513   K  Q  Y  K  #  #  #  #  #  Q  N  N  D  M  N  E  V  I  G  F  527
Clau_S  513   K  Q  Y  K  #  #  #  #  #  Q  N  N  N  M  N  E  V  I  G  F  527
Pkil_S  513   K  Q  Y  K  #  #  #  #  #  Q  N  N  D  M  N  E  V  I  G  F  527
Psoy_S  513   K  Q  Y  K  #  #  #  #  #  Q  N  N  D  M  N  E  V  I  G  F  527
Vok     513   K  Q  Y  K  #  #  #  #  #  Q  N  N  D  M  N  E  I  I  G  F  527
Cpac_S  516   Q  Q  Y  N  H  #  #  #  E  Q  D  S  E  M  N  E  V  L  G  F  532
Cfau_S  516   Q  Q  Y  N  H  #  #  #  E  Q  D  S  E  M  N  E  V  V  G  F  532
Cnau_S  517   Q  Q  Y  N  H  #  #  #  E  Q  D  S  E  M  N  E  V  V  G  F  533
Pste_S  516   Q  Q  Y  N  H  #  #  #  E  Q  D  S  E  M  N  E  V  V  G  F  532
Rma     513   K  Q  Y  N  H  #  #  #  E  Q  D  N  K  M  S  E  V  M  G  F  529
Ifos_S  515   Q  Q  Y  N  H  #  #  #  E  Q  D  S  G  M  N  E  V  V  G  F  531
Apha_S  424   Q  P  Y  N  H  T  T  #  E  Q  D  S  E  M  N  E  V  V  G  F  442
Bsep_S  516   E  Q  Y  T  H  #  #  #  E  E  D  S  E  M  N  E  V  T  G  F  532
```

UvrD domain (6-716)

```
Akaw_S 1582 ATTTCTTTAGCATCAATAGATTCTGGGGGTAATACTAATGCACTTATTAATCAAAATGTA 1641
Clau_S 1582 ATTTCTTTAGCATCAATAGATTCTGACAATAATACTAATGCACTTAATAATCAAAATGTA 1641
Pkil_S 1582 ATTTCTTTAGCATCAATAGATTCTGGGGGTAATAATAATGCACTTATTAATCAAAATGTA 1641
Psoy_S 1582 ATTTCTTTAGCATCAATAGATTCTGGGGGTAATAATAATGCACTTATTAATCAAAATGTA 1641
Vok    1582 ATTTCTTTAGCATCAATAGATTCTGGGGGTAATACTAATACACTTACTAATCAAAATGTA 1641
Cpac_S 1597 ATCTCTTTAGCATCGCTAGATTCTAGTGGCGATACCAATGCACTTATTAACCAAAATATA 1656
Cfau_S 1597 ATCTCTTTAGCATCACTAGATTCTAGTGGCGATACC---------ATTAACCAAAATGTG 1647
Cnau_S 1600 ATCTCTTTAGCATCGCTAGATTCTAGTGGCGATACCAATGCACCTATTAACCAAAATGTG 1659
Pste_S 1597 ATCTCTTTAGCATTGCTAGATTCTAATGGCGATACCAATGCACCTATTAATCAAAATGTG 1656
Rma    1588 ATTTCTTTAACATCGCTAGATTCTAGTGGCAATACCAATGCGTCTATTAACCAAAATGTA 1647
Ifos_S 1597 ATCTCTTT---------------------------------------------------- 1604
Apha_S 1332 ATCTCTTTAGCATCGCTGGATTCTAGTGGTGACACCAATGCACCTATTAACCAAAATATA 1391
Bsep_S 1597 ATCTCACTCGCTTCGCTTGATTCTAGTGGCGACAGCAGTGCACCACCGACGCAAAATGTG 1656
Akaw_S  528   I  S  L  A  S  I  D  S  G  G  N  T  N  A  L  I  N  Q  N  V  547
Clau_S  528   I  S  L  A  S  I  D  S  D  N  N  T  N  A  L  N  N  Q  N  V  547
Pkil_S  528   I  S  L  A  S  I  D  S  G  G  N  N  N  A  L  I  N  Q  N  V  547
Psoy_S  528   I  S  L  A  S  I  D  S  G  G  N  N  N  A  L  I  N  Q  N  V  547
Vok     528   I  S  L  A  S  I  D  S  G  G  N  T  N  T  L  T  N  Q  N  V  547
Cpac_S  533   I  S  L  A  S  L  D  S  S  G  D  T  N  A  L  I  N  Q  N  I  552
Cfau_S  533   I  S  L  A  S  L  D  S  S  G  D  T  #  #  #  I  N  Q  N  V  549
Cnau_S  534   I  S  L  A  S  L  D  S  S  G  D  T  N  A  P  I  N  Q  N  V  553
Pste_S  533   I  S  L  A  L  L  D  S  N  G  D  T  N  A  P  I  N  Q  N  V  552
Rma     530   I  S  L  T  S  L  D  S  S  G  N  T  N  A  S  I  N  Q  N  V  549
Ifos_S  532   I  S  L  #  #  #  #  #  #  #  #  #  #  #  #  #  #  #  #  #  534
Apha_S  443   I  S  L  A  S  L  D  S  S  G  D  T  N  A  P  I  N  Q  N  I  462
Bsep_S  533   I  S  L  A  S  L  D  S  S  G  D  S  S  A  P  P  T  Q  N  V  552
```

UvrD domain (6-716)

```
Akaw_S 1642 CAACTTATGACTATGCATTCAGCTAAAGGATTAGAGTTTCCTTATGTATTCTTAAGCGGC 1701
Clau_S 1642 CAACTTATGACTATGCATTCAGCTAAGGGCTTAGAATTTCCTTATGTATTCTTAAGCGGA 1701
Pkil_S 1642 CAACTTATGACTATGCATTCAGCCAAGGGGTTAGAATTTCCTTATGTATTCTTAAGCGGC 1701
Psoy_S 1642 CAACTTATGACTATGCATTCAGCCAAGGGGTTAGAATTTCCTTATGTATTCTTAAGCGGC 1701
Vok    1642 CAACTTATGACTATGCATTCAGCCAAAGGGTTAGAATTTCCTTATGTATTCTTAAGCGGC 1701
Cpac_S 1657 CAACTCATGACCATACATTCAGCCAAAGGGCTAGAGTTTCCCTATGTATTTTTGGTGGGC 1716
Cfau_S 1648 CAACTCATGACTATACATTCAGCCAAAGGGCTAGAATTTCCATATGTATTTTTGGTCGGC 1707
Cnau_S 1660 CAACTCATGACCATACATTCAGCCAAAGGGCTAGAGTTTCCCTATGTATTTTTGGTCGGC 1719
Pste_S 1657 CAACTCACGACCATGCATTCAGCCAAAGGGCTAGAGTTTCCCTATGTATTTTTGGTCGGC 1716
Rma    1648 CAACTCATGACTATACATTCAGCTAAAGGTCTAGAGTTTCCCTGTGTATTTTTGGTTGGC 1707
Ifos_S 1605 ------------------------------------------------------------ 1604
Apha_S 1392 CAACTCATGACCATACATTCAGCCAAAGGGTTAGATTTTCCTTATGTGTTTTTGGTTGAT 1451
Bsep_S 1657 CAATTAATGACCATCCACTCTGCCAAAGGCTTAGAGTTTCCGTATGTATTTTTAGCAGGC 1716
Akaw_S  548   Q  L  M  T  M  H  S  A  K  G  L  E  F  P  Y  V  F  L  S  G  567
Clau_S  548   Q  L  M  T  M  H  S  A  K  G  L  E  F  P  Y  V  F  L  S  G  567
Pkil_S  548   Q  L  M  T  M  H  S  A  K  G  L  E  F  P  Y  V  F  L  S  G  567
Psoy_S  548   Q  L  M  T  M  H  S  A  K  G  L  E  F  P  Y  V  F  L  S  G  567
Vok     548   Q  L  M  T  M  H  S  A  K  G  L  E  F  P  Y  V  F  L  S  G  567
Cpac_S  553   Q  L  M  T  I  H  S  A  K  G  L  E  F  P  Y  V  F  L  V  G  572
Cfau_S  550   Q  L  M  T  I  H  S  A  K  G  L  E  F  P  Y  V  F  L  V  G  569
Cnau_S  554   Q  L  M  T  I  H  S  A  K  G  L  E  F  P  Y  V  F  L  V  G  573
Pste_S  553   Q  L  T  T  M  H  S  A  K  G  L  E  F  P  Y  V  F  L  V  G  572
Rma     550   Q  L  M  T  I  H  S  A  K  G  L  E  F  P  C  V  F  L  V  G  569
Ifos_S  535   #  #  #  #  #  #  #  #  #  #  #  #  #  #  #  #  #  #  #  #  534
Apha_S  463   Q  L  M  T  I  H  S  A  K  G  L  D  F  P  Y  V  F  L  V  D  482
Bsep_S  553   Q  L  M  T  I  H  S  A  K  G  L  E  F  P  Y  V  F  L  A  G  572

E. coli          L  M  T  L  H  S  A  K  G  L  E  F  P

                       Helicase motif V
```

UvrD domain (6-716)

```
Akaw_S 1702 ATGGAAGAAGGTTTATTCCCCTCT---AGGCAAAGCCTAGATAAACCCCATTTAATAAGT 1758
Clau_S 1702 ATGGAAGAAGGTTTATTCCCCTCT---AGGCAAAGCAAAAATGAACCACATTTAATAGAT 1758
Pkil_S 1702 ATGGAAGAAGGTTTATCCCCCTACTACAGGCAAAGCAAAAATAAACCACATTTAATAGAT 1761
Psoy_S 1702 ATGGAAGAAGGTTTATCCCCCTACTACAGGCAAAGCAAAAATAAACCACATTTAATAGAT 1761
Vok    1702 ATGGAAGAAGGTTTATCCCCCTAC---AGGCAAAGCAAAAATAAACCACATTTAATAGAT 1758
Cpac_S 1717 ATGGAAGAGGATTTGTTTCCTTCC---AGACAAAGCAAGGATGAACCACATTTAATGGAT 1773
Cfau_S 1708 ATGGAAGAGGACTTGTTTCCTTCC---AGACAAAGCAAGGATGAACCACATTTAATGGAT 1764
Cnau_S 1720 ATGGAAGAGGACTTGTTTCCTTCC---AGACAAAGCAAAGATGAACCACATTTAATGGAT 1776
Pste_S 1717 ATGGAAGAGGACTTGTTTCCTTGC---AGACAAAGCAAGGATGAACCACATTTAATGGAT 1773
Rma    1708 ATGGAAGAAGACCTATTCCCTTCT---AGACAAAGTAAGAATGAACCTCATTTAACGGAT 1764
Ifos_S 1605 ------------------------------------------------------------ 1604
Apha_S 1452 ACGGAAGAGGACTTATTCCCCTCT---AGACAAAGCAAGGATGAGCCTCATTTAATGGAT 1508
Bsep_S 1717 TTAGAAGAAGACCTATTTCCATCT---AGGCAGTCCAAAGAGGAGCCGCATTTATTAGAT 1773
Akaw_S  568   M  E  E  G  L  F  P  S  #  R  Q  S  L  D  K  P  H  L  I  S  586
Clau_S  568   M  E  E  G  L  F  P  S  #  R  Q  S  K  N  E  P  H  L  I  D  586
Pkil_S  568   M  E  E  G  L  S  P  Y  Y  R  Q  S  K  N  K  P  H  L  I  D  587
Psoy_S  568   M  E  E  G  L  S  P  Y  Y  R  Q  S  K  N  K  P  H  L  I  D  587
Vok     568   M  E  E  G  L  S  P  Y  #  R  Q  S  K  N  K  P  H  L  I  D  586
Cpac_S  573   M  E  E  D  L  F  P  S  #  R  Q  S  K  D  E  P  H  L  M  D  591
Cfau_S  570   M  E  E  D  L  F  P  S  #  R  Q  S  K  D  E  P  H  L  M  D  588
Cnau_S  574   M  E  E  D  L  F  P  S  #  R  Q  S  K  D  E  P  H  L  M  D  592
Pste_S  573   M  E  E  D  L  F  P  C  #  R  Q  S  K  D  E  P  H  L  M  D  591
Rma     570   M  E  E  D  L  F  P  S  #  R  Q  S  K  N  E  P  H  L  T  D  588
Ifos_S  535   #  #  #  #  #  #  #  #  #  #  #  #  #  #  #  #  #  #  #  #  534
Apha_S  483   T  E  E  D  L  F  P  S  #  R  Q  S  K  D  E  P  H  L  M  D  501
Bsep_S  573   L  E  E  D  L  F  P  S  #  R  Q  S  K  E  E  P  H  L  L  D  591

E. coli                                                          L  E

                                                   Helicase motif VI
```

UvrD domain (6-716)

```
Akaw_S 1759 GAAGAAAGACGATTATGCTACGTTGGCATGACCCGTGCTATGAAAAAACTGTCACTATCT 1818
Clau_S 1759 GAAGAAAGACGATTATGCTACGTTGGCATGACCCGTGCCATGAAAAAACTATCACTATCT 1818
Pkil_S 1762 GAAGAAAGACGATTATGCTACGTTGGCATGACACGTGCCATGAAAAAACTGTCGCTATCT 1821
Psoy_S 1762 GAAGAAAGACGATTATGCTACGTTGGCATGACACGTGCCATGAAAAAACTGTCGCTATCT 1821
Vok    1759 GAAGAAAGACGATTATGCTACGTTGGCATGACCCGTGCCATGAAAAAACTGTCACTATCT 1818
Cpac_S 1774 GAAGAAAGACGGTTGTGCTACGTGGGCATGACTCGCGTTATAAAAAAATTATCACTTTCT 1833
Cfau_S 1765 GAAGAAAGACGGTTGTGCTACGTGGGCATGACTCGCGTCATGAAAAAATTATCACTTTCT 1824
Cnau_S 1777 GAAGAAAGACGGTTGTGCTACGTGGGCATGACTCGCGCCATGAAAAAATTATCACTTTCT 1836
Pste_S 1774 GAAGAAAGACGGTTATGCTACATGGGCATGACTCGTGCCATGAAAAAATTATCACTTTCT 1833
Rma    1765 GAAGAAAGACGATTATGCTACGTGGGTATGACTCGCGCTATGAAAAAACTATCACTCTCT 1824
Ifos_S 1605 ------------------------------------------------------------ 1604
Apha_S 1509 GAAGAAAGACGATTATGCTACGTGGGTATGACTCGTGCTATGAAAAAACTACCACTCTCT 1568
Bsep_S 1774 GAAGAAAGGCGTCTATGCTATGTTGGTATGACCCGTGCAATGAAAAAACTCACTCTGTCA 1833
Akaw_S  587  E  E  R  R  L  C  Y  V  G  M  T  R  A  M  K  K  L  S  L  S  606
Clau_S  587  E  E  R  R  L  C  Y  V  G  M  T  R  A  M  K  K  L  S  L  S  606
Pkil_S  588  E  E  R  R  L  C  Y  V  G  M  T  R  A  M  K  K  L  S  L  S  607
Psoy_S  588  E  E  R  R  L  C  Y  V  G  M  T  R  A  M  K  K  L  S  L  S  607
Vok     587  E  E  R  R  L  C  Y  V  G  M  T  R  A  M  K  K  L  S  L  S  606
Cpac_S  592  E  E  R  R  L  C  Y  V  G  M  T  R  V  I  K  K  L  S  L  S  611
Cfau_S  589  E  E  R  R  L  C  Y  V  G  M  T  R  V  M  K  K  L  S  L  S  608
Cnau_S  593  E  E  R  R  L  C  Y  V  G  M  T  R  A  M  K  K  L  S  L  S  612
Pste_S  592  E  E  R  R  L  C  Y  M  G  M  T  R  A  M  K  K  L  S  L  S  611
Rma     589  E  E  R  R  L  C  Y  V  G  M  T  R  A  M  K  K  L  S  L  S  608
Ifos_S  535  #  #  #  #  #  #  #  #  #  #  #  #  #  #  #  #  #  #  #  #  534
Apha_S  502  E  E  R  R  L  C  Y  V  G  M  T  R  A  M  K  K  L  P  L  S  521
Bsep_S  592  E  E  R  R  L  C  Y  V  G  M  T  R  A  M  K  K  L  T  L  S  611

E. coli      E  E  R  R  L  A  Y  V  G  V  T  R  A

                 Helicase motif VI
```

UvrD domain (6-716)

```
Akaw_S 1819 TACGCCATAAAAAGATTTTTACACGGACATTTTTTATACACTAATCCTTCACGCTTCCTA 1878
Clau_S 1819 TACACCATAAAACGATTTTTACACGGACATTTTTTATACGCTGATCCTTCACGTTTCCTA 1878
Pkil_S 1822 TACACCATGAAACGATTTTTACACGGACATTTTTTGTACGCTGATCCTTCACGTTTCCTA 1881
Psoy_S 1822 TACACCATGAAACGATTTTTACACGGACATTTTTTGTACGCTGATCCTTCACGTTTCCTA 1881
Vok    1819 TACACCATGAAACGATTTTTACACGGACATTTTTTGTACGCTGATCCTTCACGTTTCCTA 1878
Cpac_S 1834 TATGCCATTAAACGCTTTTTGCACGGAGAATCCTTATACCCTTGTCCTTCACGTTTTTTA 1893
Cfau_S 1825 TATGCCATTAAACGCTTTTTGCACGGAGAGTTTTTACACGCTTATCCTTCACGTTTTTTA 1884
Cnau_S 1837 TATGCCATTAAACGCTTTTTGCACGGACAATCCTTATACGCTTATCCTTCGCGTTTTTTA 1896
Pste_S 1834 TATGCCATTAAACGCTTTTTGTACAGACAATCCTTATACGCTCATCCTTCACGTTTTTTA 1893
Rma    1825 TATGCCATTAAACGCTTTTTGTACGGACAATCTTTATACGCTTATCCTTCACGTTTTTTA 1884
Ifos_S 1605 ------------------------------------------------------------ 1604
Apha_S 1569 TATGCTATTAAACGCTTTTTACACGGACAGTCTTTATACGCTTATCCTTCACGTTTTTTA 1628
Bsep_S 1834 TTCGCCATTAAACGCTTCTTGCATGGACAATCCAACTACGCCTACCCATCTCGATTCTTG 1893
Akaw_S  607  Y  A  I  K  R  F  L  H  G  H  F  L  Y  T  N  P  S  R  F  L  626
Clau_S  607  Y  T  I  K  R  F  L  H  G  H  F  L  Y  A  D  P  S  R  F  L  626
Pkil_S  608  Y  T  M  K  R  F  L  H  G  H  F  L  Y  A  D  P  S  R  F  L  627
Psoy_S  608  Y  T  M  K  R  F  L  H  G  H  F  L  Y  A  D  P  S  R  F  L  627
Vok     607  Y  T  M  K  R  F  L  H  G  H  F  L  Y  A  D  P  S  R  F  L  626
Cpac_S  612  Y  A  I  K  R  F  L  H  G  E  S  L  Y  P  C  P  S  R  F  L  631
Cfau_S  609  Y  A  I  K  R  F  L  H  G  E  F  L  H  A  Y  P  S  R  F  L  628
Cnau_S  613  Y  A  I  K  R  F  L  H  G  Q  S  L  Y  A  Y  P  S  R  F  L  632
Pste_S  612  Y  A  I  K  R  F  L  Y  R  Q  S  L  Y  A  H  P  S  R  F  L  631
Rma     609  Y  A  I  K  R  F  L  Y  G  Q  S  L  Y  A  Y  P  S  R  F  L  628
Ifos_S  535  #  #  #  #  #  #  #  #  #  #  #  #  #  #  #  #  #  #  #  #  534
Apha_S  522  Y  A  I  K  R  F  L  H  G  Q  S  L  Y  A  Y  P  S  R  F  L  541
Bsep_S  612  F  A  I  K  R  F  L  H  G  Q  S  N  Y  A  Y  P  S  R  F  L  631
```

UvrD domain (6-716)

```
Akaw_S 1879 AGCGAAATTCCAAATGAATATTTAAACAGGATTAAAATTAAA------------------ 1920
Clau_S 1879 AGCGAGATTCCAGATGAATATTTAAATAGGATTAAAGTTAAATCCGATGTAATAATAAAA 1938
Pkil_S 1882 AATGAAATTCCAGATGAATGTTTAAATAAAATTAAAGTTAAATTTGATATAAAAATAAAA 1941
Psoy_S 1882 AATGAAATTCCAGATGAATGTTTAAATAAAATTAAAGTTAAATTTGATATAAAAATAAAA 1941
Vok    1879 AATGAGATTCCAGATGAATATTTAAATAAAATTAAAGTTAAATTTGATATAACAATAAAA 1938
Cpac_S 1894 GACGAAATTCCAAGTGAATATTTAAATAAAATTAAAGCTAAATTTAGTGCAACAAAACAA 1953
Cfau_S 1885 GACGAAATTCCAAGTAGATATTTAAATAAAATTAAGGCTAAATTTAGTGCAACAATACAA 1944
Cnau_S 1897 GACGAAATCCCAAGTGAATATTTAAATAAAATTAAAGCTAAATTTGGTGCAACAATACAA 1956
Pste_S 1894 GACGAAATCCCAAGTGAATATTTAAATAAAATTAAAGCTAAATTTGGTACAACAATACAA 1953
Rma    1885 AACGAAATTCCAAGTAAATATTTAAATAAGATTAAAGCTAAATTTGGTGCAACAATACAG 1944
Ifos_S 1605 ------------------------------------------------------------ 1604
Apha_S 1629 GACGAAATTCTAAGTGAATATTTAAATAAAATTAAGGCTAAATTTGGTGCAACAATACAA 1688
Bsep_S 1894 TCAGAATTTCCAGAAAAACATCTTAATCAGATTAAACAAAAATATGGCGCAACACCGCAA 1953
Akaw_S  627   S  E  I  P  N  E  Y  L  N  R  I  K  I  K  #  #  #  #  #  #  640
Clau_S  627   S  E  I  P  D  E  Y  L  N  R  I  K  V  K  S  D  V  I  I  K  646
Pkil_S  628   N  E  I  P  D  E  C  L  N  K  I  K  V  K  F  D  I  K  I  K  647
Psoy_S  628   N  E  I  P  D  E  C  L  N  K  I  K  V  K  F  D  I  K  I  K  647
Vok     627   N  E  I  P  D  E  Y  L  N  K  I  K  V  K  F  D  I  T  I  K  646
Cpac_S  632   D  E  I  P  S  E  Y  L  N  K  I  K  A  K  F  S  A  T  K  Q  651
Cfau_S  629   D  E  I  P  S  R  Y  L  N  K  I  K  A  K  F  S  A  T  I  Q  648
Cnau_S  633   D  E  I  P  S  E  Y  L  N  K  I  K  A  K  F  G  A  T  I  Q  652
Pste_S  632   D  E  I  P  S  E  Y  L  N  K  I  K  A  K  F  G  T  T  I  Q  651
Rma     629   N  E  I  P  S  K  Y  L  N  K  I  K  A  K  F  G  A  T  I  Q  648
Ifos_S  535   #  #  #  #  #  #  #  #  #  #  #  #  #  #  #  #  #  #  #  #  534
Apha_S  542   D  E  I  L  S  E  Y  L  N  K  I  K  A  K  F  G  A  T  I  Q  561
Bsep_S  632   S  E  F  P  E  K  H  L  N  Q  I  K  Q  K  Y  G  A  T  P  Q  651
```

UvrD domain (6-716)

```
Akaw_S 1921 ------------------------------------------TCTGATAATCAATTA 1935
Clau_S 1939 AATTACAATAATAATAATTTTTTT---CAAAGTATTACATCTAAATCTGATAATCAATTA 1995
Pkil_S 1942 AATTACAATAATAATATTTTTAAT---CAAAGCATTACACCTAAATCTGATAATCAATTA 1998
Psoy_S 1942 AATTACAATAATAATATTTTTAAT---CAAAGCATTACACCTAAATCTGATAATCAATTA 1998
Vok    1939 AATTACAATAATAATAATATTTTTAATCAAAGCATTACACCTAAATCTGATAATCAATTA 1998
Cpac_S 1954 AATTATAATGCTGATAATACTTTTAATCAAAATATCACACTCAAATCTGATGGCCAATTA 2013
Cfau_S 1945 AATTACAATACTGATAATACTTTTAATCAAAATATCACACCCAAATCTAATGGCCAATTA 2004
Cnau_S 1957 AATTATAATGCTGATAATACTTTTAATCAAAATATCATGCCCAAATCTGATGGCCAATTA 2016
Pste_S 1954 AATTACAATGCTGATAATACTTTTAATCAAAATATCACACCCAAATCTGATGGCCAATTA 2013
Rma    1945 AATTACAATGATGACAATACTTTTAATAAAAATATTACACCAAAATCTGATGACCAAATA 2004
Ifos_S 1605 ------------------------------------------------------------ 1604
Apha_S 1689 AATTACAATGATGATGACACTTTTAATCAAGGTATTACACCCAAATCCGATGGTCAATTA 1748
Bsep_S 1954 AATTACCAAAAAGATGATGTATTTAATCAAGATATTAAACCACAAGCAAATGGCGATTTA 2013
Akaw_S  641   #  #  #  #  #  #  #  #  #  #  #  #  #  #  #  S  D  N  Q  L  645
Clau_S  647   N  Y  N  N  N  N  F  F  #  Q  S  I  T  S  K  S  D  N  Q  L  665
Pkil_S  648   N  Y  N  N  N  I  F  N  #  Q  S  I  T  P  K  S  D  N  Q  L  666
Psoy_S  648   N  Y  N  N  N  I  F  N  #  Q  S  I  T  P  K  S  D  N  Q  L  666
Vok     647   N  Y  N  N  N  N  I  F  N  Q  S  I  T  P  K  S  D  N  Q  L  666
Cpac_S  652   N  Y  N  A  D  N  T  F  N  Q  N  I  T  L  K  S  D  G  Q  L  671
Cfau_S  649   N  Y  N  T  D  N  T  F  N  Q  N  I  T  P  K  S  N  G  Q  L  668
Cnau_S  653   N  Y  N  A  D  N  T  F  N  Q  N  I  M  P  K  S  D  G  Q  L  672
Pste_S  652   N  Y  N  A  D  N  T  F  N  Q  N  I  T  P  K  S  D  G  Q  L  671
Rma     649   N  Y  N  D  D  N  T  F  N  K  N  I  T  P  K  S  D  D  Q  I  668
Ifos_S  535   #  #  #  #  #  #  #  #  #  #  #  #  #  #  #  #  #  #  #  #  534
Apha_S  562   N  Y  N  D  D  D  T  F  N  Q  G  I  T  P  K  S  D  G  Q  L  581
Bsep_S  652   N  Y  Q  K  D  D  V  F  N  Q  D  I  K  P  Q  A  N  G  D  L  671
```

UvrD domain (6-716)

```
Akaw_S 1936 CACATTGGTGCTTTAGTTAACCACACCAAATTCGGATTAGGAACGGTGTTAAATTTTGAA 1995
Clau_S 1996 CATATTGGTGCTTTAGTTAAACATACTAAATTCGGATTAGGAACGGTATTAAATTTTGAA 2055
Pkil_S 1999 CATATTGGTGCTTTAGTCAATCACCCCAAATTCGGATTAGGAACGGTATTAAATTTTGAA 2058
Psoy_S 1999 CATATTGGTGCTTTAGTCAATCACCCCAAATTCGGATTAGGAACGGTATTAAATTTTGAA 2058
Vok    1999 CATATTGGTGCTTTAGTCAACCACCCTAAATTCGGATTAGGAACAGTATTAAATTTTGAA 2058
Cpac_S 2014 TCCATCGGTACTTCAGTTAAACATGCCAAATTTGGATTAGGAACAGTGTTGAACTTCGAA 2073
Cfau_S 2005 TTCATCGGTGCTCCAGTTAAACATGCCAAATTTGGATTAGGAACAGTGTTGAACTTCGAA 2064
Cnau_S 2017 TCCATCGGTGCTTCAGTTAAACACGCCAAATTTGGATTAGGAATAGTGTTGAACTTCGAA 2076
Pste_S 2014 TCCATCGGTGCTTCAGTTAAACATGCCAAATTTGGATTAGGAACAGTGTTGAACTTCGAA 2073
Rma    2005 TCTATTGGCGCTTTGGTTAAACATGCCAAATTTGGATTAGGTACAGTGCTAAATTTTGAA 2064
Ifos_S 1605 ---------------------------------------------------------GAA 1607
Apha_S 1749 TCCATTAGTGCTTTGGTTAAACACGCCAAATTTGGCGTGGGTATGGTGCTAAATTTTAAA 1808
Bsep_S 2014 TCCATTGGCAGAACTGTTAAACATGCCAAATTTGGCTTTGGCACTGTGCTTAATTTTGAA 2073
Akaw_S  646   H  I  G  A  L  V  N  H  T  K  F  G  L  G  T  V  L  N  F  E  665
Clau_S  666   H  I  G  A  L  V  K  H  T  K  F  G  L  G  T  V  L  N  F  E  685
Pkil_S  667   H  I  G  A  L  V  N  H  P  K  F  G  L  G  T  V  L  N  F  E  686
Psoy_S  667   H  I  G  A  L  V  N  H  P  K  F  G  L  G  T  V  L  N  F  E  686
Vok     667   H  I  G  A  L  V  N  H  P  K  F  G  L  G  T  V  L  N  F  E  686
Cpac_S  672   S  I  G  T  S  V  K  H  A  K  F  G  L  G  T  V  L  N  F  E  691
Cfau_S  669   F  I  G  A  P  V  K  H  A  K  F  G  L  G  T  V  L  N  F  E  688
Cnau_S  673   S  I  G  A  S  V  K  H  A  K  F  G  L  G  I  V  L  N  F  E  692
Pste_S  672   S  I  G  A  S  V  K  H  A  K  F  G  L  G  T  V  L  N  F  E  691
Rma     669   S  I  G  A  L  V  K  H  A  K  F  G  L  G  T  V  L  N  F  E  688
Ifos_S  535   #  #  #  #  #  #  #  #  #  #  #  #  #  #  #  #  #  #  #  E  535
Apha_S  582   S  I  S  A  L  V  K  H  A  K  F  G  V  G  M  V  L  N  F  K  601
Bsep_S  672   S  I  G  R  T  V  K  H  A  K  F  G  F  G  T  V  L  N  F  E  691
```

UvrD domain (6-716)

```
Akaw_S 1996 GGACAAGGGGATAGTTCCAGA---GTACAGATAAAATTTAAACAAACAGGTAGTATAAAA 2052
Clau_S 2056 GGGCAAGGGAATACTTCCAGA---GTACAGATAAAATTTAAACAAGCAGGCAGTACAAAA 2112
Pkil_S 2059 GGACAAGGGGATAGTTCTAGA---ATACAGATAAAATTTAAACAAGCAGGCAGTATAAAA 2115
Psoy_S 2059 GGACAAGGGGATAGTTCTAGA---ATACAGATAAAATTTAAACAAGCAGGCAGTATAAAA 2115
Vok    2059 GGACAAGGGGATAGTTCTAGA---ATACAGATAAAATTTAAACAAGCAGGCAGTATAAAA 2115
Cpac_S 2074 GGTCAAGGTGATTCTGCCAGG---ATACAAATAAAATTTAAGCAAGTGGGC---ACAAAA 2127
Cfau_S 2065 GGTCAAGACGATTCTGTCAGG---ATACAAATAAAATTTAAGCAAGTGGGC---ACAAAA 2118
Cnau_S 2077 GGTCAAGGTGATTCTGCCAGG---GTACAAATAAAATTTAAGCAAGTGGGC---ACAAAA 2130
Pste_S 2074 GGTCAAGGCGATTCTGCCAGG---GTACAAATAAAATTTAAGCAAGTGGGC---ATAAAA 2127
Rma    2065 GGGCAAGGCGATTCTACTAGA---GTGCAAATAAAATTTAAGCAAGTGGGT---ACAAAA 2118
Ifos_S 1608 GGGCAAGGCGATTCTACTAGG---GTGCAAATAAAATTTAAACAAGTAGGC---ACAAAA 1661
Apha_S 1809 GGGCAAGGCGATTCTGATAGGG--GTACAAATAAAATTTAAACAAGCAGAC---ACAAAA 1863
Bsep_S 2074 GGCGATGGCGAGAGTGCCAGA---GTGCAAGTGAAATTCAAAACCGCAGGC---ACAAAA 2127
Akaw_S  666   G  Q  G  D  S  S  R  #  V  Q  I  K  F  K  Q  T  G  S  I  K  684
Clau_S  686   G  Q  G  N  T  S  R  #  V  Q  I  K  F  K  Q  A  G  S  T  K  704
Pkil_S  687   G  Q  G  D  S  S  R  #  I  Q  I  K  F  K  Q  A  G  S  I  K  705
Psoy_S  687   G  Q  G  D  S  S  R  #  I  Q  I  K  F  K  Q  A  G  S  I  K  705
Vok     687   G  Q  G  D  S  S  R  #  I  Q  I  K  F  K  Q  A  G  S  I  K  705
Cpac_S  692   G  Q  G  D  S  A  R  #  I  Q  I  K  F  K  Q  V  G  #  T  K  709
Cfau_S  689   G  Q  D  D  S  V  R  #  I  Q  I  K  F  K  Q  V  G  #  T  K  706
Cnau_S  693   G  Q  G  D  S  A  R  #  V  Q  I  K  F  K  Q  V  G  #  T  K  710
Pste_S  692   G  Q  G  D  S  A  R  #  V  Q  I  K  F  K  Q  V  G  #  I  K  709
Rma     689   G  Q  G  D  S  T  R  #  V  Q  I  K  F  K  Q  V  G  #  T  K  706
Ifos_S  536   G  Q  G  D  S  T  R  #  V  Q  I  K  F  K  Q  V  G  #  T  K  553
Apha_S  602   G  Q  G  D  S  D  R  #  V  Q  I  K  F  K  Q  A  D  #  T  K  619
Bsep_S  692   G  D  G  E  S  A  R  #  V  Q  V  K  F  K  T  A  G  #  T  K  709
```

UvrD domain (6-716)

```
Akaw_S 2053 TGGTTGATTAGTTCCTATGCAAATTTAGAATTTATATAA 2091
Clau_S 2113 TGGTTGATCAGTTCCTATGCAAATTTAGAATTTATATAG 2151
Pkil_S 2116 TGGTTGATTAGTTCCTATGCAAATTTAGAATTTATATAA 2154
Psoy_S 2116 TGGTTGATTAGTTCCTATGCAAATTTAGAATTTATATAA 2154
Vok    2116 TGGTTGATTAGTTCTTATGCAAATTTAGAATTTATATAG 2154
Cpac_S 2128 TGGCTGATTAGTTCGTATGCAAATTTAGAATTTGTATAA 2166
Cfau_S 2119 TGGCTGATTAGTTCGTATGCAAATTTAGAATTTGTATAA 2157
Cnau_S 2131 TGGCTAATTAGTGCGTATGCAAATTTAGAATTTGTATAA 2169
Pste_S 2128 TGGCTGATTAGTTCGTATGCAAATTTAGAATTTGTATAA 2166
Rma    2119 TGGCTGATTAATTCATATGCTAATTTAGAATTTATTTAA 2157
Ifos_S 1662 TGGCTGATTAGTTCGTATGCAAACCTAGGATTCGTTTAA 1700
Apha_S 1864 TAGCTGATTAGTTCGTATGCAAGCTTATAAGTTATATAG 1902
Bsep_S 2128 TGGCTGATTAGTGCTTACGCTAATTTAGAATTTATTTAA 2166
Akaw_S  685   W  L  I  S  S  Y  A  N  L  E  F  I  *  697
Clau_S  705   W  L  I  S  S  Y  A  N  L  E  F  I  *  717
Pkil_S  706   W  L  I  S  S  Y  A  N  L  E  F  I  *  718
Psoy_S  706   W  L  I  S  S  Y  A  N  L  E  F  I  *  718
Vok     706   W  L  I  S  S  Y  A  N  L  E  F  I  *  718
Cpac_S  710   W  L  I  S  S  Y  A  N  L  E  F  V  *  722
Cfau_S  707   W  L  I  S  S  Y  A  N  L  E  F  V  *  719
Cnau_S  711   W  L  I  S  A  Y  A  N  L  E  F  V  *  723
Pste_S  710   W  L  I  S  S  Y  A  N  L  E  F  V  *  722
Rma     707   W  L  I  N  S  Y  A  N  L  E  F  I  *  719
Ifos_S  554   W  L  I  S  S  Y  A  N  L  G  F  V  *  566
Apha_S  620   *  L  I  S  S  Y  A  S  L  *  V  I  *  632
Bsep_S  710   W  L  I  S  A  Y  A  N  L  E  F  I  *  722
```

uvrD domain (6-716)

Reference.


7. Curti E, Smerdon SJ, Davis EO. Characterization of the helicase activity and substrate specificity of Mycobacterium tuberculosis UvrD. J Bacteriol. 2007;189(5):1542-55. doi: 10.1128/JB.01421-06. PubMed PMID: 17158674; PubMed Central PMCID: PMC1855738.
