# Supplementary material for: Loss of genes related to Nucleotide Excision Repair (NER) and implications for reductive genome evolution in symbionts of deep-sea vesicomyid clams
Source: PLoS One. 2017 Feb 15;12(2):e0171274. doi: 10.1371/journal.pone.0171274 (PMC5310779; doi:10.1371/journal.pone.0171274)
Supplement: S6 Fig — The arrangement of nucleotide and amino acid sequences is as described in S1 Fig. Conserved domains of uvrD found in an NCBI blast search are shown as bidirectional arrows. # indicates the gap of amino acid sequence where no corresponding nucleotide sequence exist. * indicates stop codon. (PDF) [file pone.0171274.s009.pdf]

S6 Fig.

```
Akaw_S     1 GTGAATGATCAAAAACAAAGGCTACAAGCGTTAGACGTTAGTCAATCTTTTATTGTTCAA   60
Clau_S     1 GTGAATGACCAAAAACAAAGGCGAGAAGCATTAGACGTTAGTCAATCTTTTATTATTCAA   60
Pkil_S     1 GTGAATGACCAAAAACAAAGGCGACAAGCATTAGACGTTAGTCAATCTTTTATTATTCAA   60
Psoy_S     1 GTGAATGACCAAAAACAAAGGCGACAAGCATTAGACGTTAGTCAATCTTTTATTATTCAA   60
Vok        1 GTGAATGACCAAAAACAAAGGCGACAAGCATTAGACGTTAGTCAATCTTTTATTATTCAA   60
Cpac_S     1 ATGAATGACCAAGCTCAACGCCAACAGGCCTTAGATACTAGCCGGTCTTTTATCGTTCAA   60
Cfau_S     1 ATGAATGACCAAGTCCAACGCCAACAAGCTTTAGATATTAGCCGGTCTTTTATCGTTCAA   60
Cnau_S     1 GTGAATGATCAAGCCCAACGCCAACAAGCCTTAGATATTAGCCAGTCTTTTATCGTTCAA   60
Pste_S     1 GTGAATGACCAAGCCCAACGCCAACAAGCCTTAGATATTAGCCGGTCTTTTATCGTTCAA   60
Rma        1 GTGAATGATCAAACACAAAGGCGACAAGCATTAGATGTTAGTCAATCTTTTATTGTTCAA   60
Ifos_S     1 GTGAATGACCAAACGCAAAGGCGACAAGCATTAGATGTTAGTCAATCTTTTATTGTTCAA   60
Apha_S     1 GTGAATGACCAAACACAAAGGCGACAAGCATTAGACGTTAGTCAATCTTTTATCGTTCAA   60
Bsep_S     1 GTGAATGACCAAAAACAAAGAAATCAAGCCTTAGATATTAGCCAGTCTTTTATCGTGCAA   60
Akaw_S     1   M  N  D  Q  K  Q  R  L  Q  A  L  D  V  S  Q  S  F  I  V  Q   20
Clau_S     1   M  N  D  Q  K  Q  R  R  E  A  L  D  V  S  Q  S  F  I  I  Q   20
Pkil_S     1   M  N  D  Q  K  Q  R  R  Q  A  L  D  V  S  Q  S  F  I  I  Q   20
Psoy_S     1   M  N  D  Q  K  Q  R  R  Q  A  L  D  V  S  Q  S  F  I  I  Q   20
Vok        1   M  N  D  Q  K  Q  R  R  Q  A  L  D  V  S  Q  S  F  I  I  Q   20
Cpac_S     1   M  N  D  Q  A  Q  R  Q  Q  A  L  D  T  S  R  S  F  I  V  Q   20
Cfau_S     1   M  N  D  Q  V  Q  R  Q  Q  A  L  D  I  S  R  S  F  I  V  Q   20
Cnau_S     1   M  N  D  Q  A  Q  R  Q  Q  A  L  D  I  S  Q  S  F  I  V  Q   20
Pste_S     1   M  N  D  Q  A  Q  R  Q  Q  A  L  D  I  S  R  S  F  I  V  Q   20
Rma        1   M  N  D  Q  T  Q  R  R  Q  A  L  D  V  S  Q  S  F  I  V  Q   20
Ifos_S     1   M  N  D  Q  T  Q  R  R  Q  A  L  D  V  S  Q  S  F  I  V  Q   20
Apha_S     1   M  N  D  Q  T  Q  R  R  Q  A  L  D  V  S  Q  S  F  I  V  Q   20
Bsep_S     1   M  N  D  Q  K  Q  R  N  Q  A  L  D  I  S  Q  S  F  I  V  Q   20
```

AAA_19 domain (17-76)

```
Akaw_S    61 GCGCCTGCAGGCTCAGGGAAAACAGAGTTATTAACGCAACGTTATTTGAAGTTGTTATTA  120
Clau_S    61 GCGCCTGCTGGTTCAGGGAAAACAGAGTTATTAACGCAACGTTATTTGAAATTATTATTA  120
Pkil_S    61 GCACCTGCTGGTTCAGGGAAAACAGAGTTATTAACGCAACGTTATTTGAAATTGTTATCA  120
Psoy_S    61 GCACCTGCTGGTTCAGGGAAAACAGAGTTATTAACGCAACGTTATTTGAAATTGTTATCA  120
Vok       61 GCGCCTGCTGGTTCAGGAAAAACAGAGTTATTAACGCAACGTTATTTGAAATTGTTATCA  120
Cpac_S    61 GCACCTGCGGGCTCGGGCAAAACGGAATTATTGACTCAGCGTTATTTAAAATTGTTATCA  120
Cfau_S    61 GCGCCTGCGGGCTCGGGCAAAACGGAGCTATTGACTCAGCGTTATTTGAAATTGTTATCA  120
Cnau_S    61 GCGCCTGCGGGCTCGGGTAAAACGGAGTTATTGACTCAGCGTTATTTGAAATTGTTATCA  120
Pste_S    61 GCGCCTGCGGGCTCGGGTAAAACAGAGTTATTGACTCAGCGTTATTTGAAATTGTTATCG  120
Rma       61 GCGCCTGCGGGTTCTGGGAAAACAGAGTTATTAACGCAACGTTATTTGAAATTATTATCA  120
Ifos_S    61 GCACCTGCAGGCTCGGGGAAGACAGAGTTATTAACGCAGCGTTATTTGAAATCGTTATCA  120
Apha_S    61 GCGCCTGCGGGTTCGGGGAAAACAGAGTTATTAACGCAGCGTTATTTGAAATTGTTATCA  120
Bsep_S    61 GCGCCAGCAGGATCGGGAAAAACAGAGTTATTAACGCAACGCTATTTGAAATTATTGGCA  120
Akaw_S    21   A  P  A  G  S  G  K  T  E  L  L  T  Q  R  Y  L  K  L  L  L   40
Clau_S    21   A  P  A  G  S  G  K  T  E  L  L  T  Q  R  Y  L  K  L  L  L   40
Pkil_S    21   A  P  A  G  S  G  K  T  E  L  L  T  Q  R  Y  L  K  L  L  S   40
Psoy_S    21   A  P  A  G  S  G  K  T  E  L  L  T  Q  R  Y  L  K  L  L  S   40
Vok       21   A  P  A  G  S  G  K  T  E  L  L  T  Q  R  Y  L  K  L  L  S   40
Cpac_S    21   A  P  A  G  S  G  K  T  E  L  L  T  Q  R  Y  L  K  L  L  S   40
Cfau_S    21   A  P  A  G  S  G  K  T  E  L  L  T  Q  R  Y  L  K  L  L  S   40
Cnau_S    21   A  P  A  G  S  G  K  T  E  L  L  T  Q  R  Y  L  K  L  L  S   40
Pste_S    21   A  P  A  G  S  G  K  T  E  L  L  T  Q  R  Y  L  K  L  L  S   40
Rma       21   A  P  A  G  S  G  K  T  E  L  L  T  Q  R  Y  L  K  L  L  S   40
Ifos_S    21   A  P  A  G  S  G  K  T  E  L  L  T  Q  R  Y  L  K  S  L  S   40
Apha_S    21   A  P  A  G  S  G  K  T  E  L  L  T  Q  R  Y  L  K  L  L  S   40
Bsep_S    21   A  P  A  G  S  G  K  T  E  L  L  T  Q  R  Y  L  K  L  L  A   40
```

AAA_19 domain (17-76)

```
Akaw_S  121 GTCAGCACTTCACCTGAGAGTGTGATTGTGATGACCTTTACTAAAAAAGCGGTGAGTGCG  180
Clau_S  121 GTCAGTGATTCTCCTGAGAGTGTGATTGTAATGACTTTTACTAAAAAAGCTGTTAGTGAG  180
Pkil_S  121 GTCAGTACTTCGCCTGAGAGTGTTATTGTGATGACCTTTACTAAAAAAGCGGTGAGTGAG  180
Psoy_S  121 GTCAGTACTTCGCCTGAGAGTGTTATTGTGATGACCTTTACTAAAAAAGCGGTGAGTGAG  180
Vok     121 GTCAGTACTTCGCCTGAGAGTGTTATTGTGATGACCTTTACTAAAAAAGCGGTAAGTGAA  180
Cpac_S  121 GTTGGCGCCTCACCTGAGAGTGTAATTGTAATGACCTTTACTAAAAAGGCAGTAAGTGAG  180
Cfau_S  121 GTTAGTGCTTCACCTGAGAGTGTAATTGTAATGACCTTTACTAAAAAGGCAGTAAGTGAG  180
Cnau_S  121 GTTAGTGCTTCGCCTGAGAGTGTAATTGCAATGACCTTTACTAAAAAGGCAGTAAGTGAA  180
Pste_S  121 GTTAGTGCTTCGCCTGAGAGTGTAATTGTAATGACCTTTACTAAAAAGGCAGTAAGTGAG  180
Rma     121 GTTAGTGCTTCACCTGAGAGTGTGATTGCAATGACTTTTACTAAAAAGGCGGTGAGCGAG  180
Ifos_S  121 GTGAGTGCTTCGCCTGAGAGTGTGATTGCAATGACCTTTACTAAAAAGGCGGTGAGTGAG  180
Apha_S  121 GTGAGTGCTTCGCCTGAAAGTGTGATTGCAATGACCTTTACTAAAAAGGCGGTGAGTGAG  180
Bsep_S  121 CATTGTGTAGAACCTGAGAGTGTCATTGCGATGACTTTTACCAATAAAGCGGTGGATGAG  180
Akaw_S   41   V  S  T  S  P  E  S  V  I  V  M  T  F  T  K  K  A  V  S  A   60
Clau_S   41   V  S  D  S  P  E  S  V  I  V  M  T  F  T  K  K  A  V  S  E   60
Pkil_S   41   V  S  T  S  P  E  S  V  I  V  M  T  F  T  K  K  A  V  S  E   60
Psoy_S   41   V  S  T  S  P  E  S  V  I  V  M  T  F  T  K  K  A  V  S  E   60
Vok      41   V  S  T  S  P  E  S  V  I  V  M  T  F  T  K  K  A  V  S  E   60
Cpac_S   41   V  G  A  S  P  E  S  V  I  V  M  T  F  T  K  K  A  V  S  E   60
Cfau_S   41   V  S  A  S  P  E  S  V  I  V  M  T  F  T  K  K  A  V  S  E   60
Cnau_S   41   V  S  A  S  P  E  S  V  I  A  M  T  F  T  K  K  A  V  S  E   60
Pste_S   41   V  S  A  S  P  E  S  V  I  V  M  T  F  T  K  K  A  V  S  E   60
Rma      41   V  S  A  S  P  E  S  V  I  A  M  T  F  T  K  K  A  V  S  E   60
Ifos_S   41   V  S  A  S  P  E  S  V  I  A  M  T  F  T  K  K  A  V  S  E   60
Apha_S   41   V  S  A  S  P  E  S  V  I  A  M  T  F  T  K  K  A  V  S  E   60
Bsep_S   41   H  C  V  E  P  E  S  V  I  A  M  T  F  T  N  K  A  V  D  E   60
```

AAA_19 domain (17-76)

```
Akaw_S  181 TTGACCACTCGAGTGATTGAATCATTAGGTTTGGCTCAAGGAAATCGACCAAAAGATCCA  240
Clau_S  181 TTGACTACTAGAGTGATTAAATCATTAGGATTAGCTCAAGGAAATCGACCAAAAGATCCA  240
Pkil_S  181 TTGACCACCCGAGTGATTGAATCATTAGGATTGGCTCAAGGAAATAGACCAAAAGATCCA  240
Psoy_S  181 TTGACCACCCGAGTGATTGAATCATTAGGATTGGCTCAAGGAAATAGACCAAAAGATCCA  240
Vok     181 TTGACCACTCGAGTGATTGAATCATTAGAATTGGCTCAAGGAAATAGACCAAAAGATCCA  240
Cpac_S  181 TTGACCACTCGAGTGATTGAGTCTTTAAAATCGGCTCAAGGCAATCGACCAAACGAGCCA  240
Cfau_S  181 TTGACTACTCGAGTGATTGGGTCTTTAAAATCGGCTCAAGGCAATCGACCAAACGAGCCA  240
Cnau_S  181 TTGACCACTCGAGTTATTGAGTCTTTAAAATCAGCTCAAGGTAACCGACCAAACGAGCCA  240
Pste_S  181 TTGACCACTCGAGTGATTGAGTCTTTAAAATCGGCTCAAGGCAATCGACCAAACGAGTCA  240
Rma     181 TTGACCACTCGAGTGATTGAGTCTTTAAAATTAGCCCAAGGTAATCGACCAAAAGAATCG  240
Ifos_S  181 TTGACCACTCGGGTGATTGAGTCTTTAAAATTAGCCCAAGGCAATCGACCAAAAGAGTTA  240
Apha_S  181 TTGACCACTCGGGTGATTGAGTCTCTAAAATTAGTCCAAGGCAATCGACCAAAAGAGTTA  240
Bsep_S  181 ATGACGCATCGGGTGTTATCGGCGTTGAAATCGACAGTGCAATCACGACCTAGTGAGCCA  240
Akaw_S   61   L  T  T  R  V  I  E  S  L  G  L  A  Q  G  N  R  P  K  D  P   80
Clau_S   61   L  T  T  R  V  I  K  S  L  G  L  A  Q  G  N  R  P  K  D  P   80
Pkil_S   61   L  T  T  R  V  I  E  S  L  G  L  A  Q  G  N  R  P  K  D  P   80
Psoy_S   61   L  T  T  R  V  I  E  S  L  G  L  A  Q  G  N  R  P  K  D  P   80
Vok      61   L  T  T  R  V  I  E  S  L  E  L  A  Q  G  N  R  P  K  D  P   80
Cpac_S   61   L  T  T  R  V  I  E  S  L  K  S  A  Q  G  N  R  P  N  E  P   80
Cfau_S   61   L  T  T  R  V  I  G  S  L  K  S  A  Q  G  N  R  P  N  E  P   80
Cnau_S   61   L  T  T  R  V  I  E  S  L  K  S  A  Q  G  N  R  P  N  E  P   80
Pste_S   61   L  T  T  R  V  I  E  S  L  K  S  A  Q  G  N  R  P  N  E  S   80
Rma      61   L  T  T  R  V  I  E  S  L  K  L  A  Q  G  N  R  P  K  E  S   80
Ifos_S   61   L  T  T  R  V  I  E  S  L  K  L  A  Q  G  N  R  P  K  E  L   80
Apha_S   61   L  T  T  R  V  I  E  S  L  K  L  V  Q  G  N  R  P  K  E  L   80
Bsep_S   61   M  T  H  R  V  L  S  A  L  K  S  T  V  Q  S  R  P  S  E  P   80
```

AAA_19 domain (17-76)

```
Akaw_S  241 CATAAACAAATTATTTATGATTTAGCTTTTCAAGTATTAGAAAGATCTAAAGCGCTTGAT  300
Clau_S  241 CATAAACAAATTATTTATGATTTAGCTTTTCAAGTATTAGAAAGATCTAAGACACTTGAT  300
Pkil_S  241 CATAAACAAATTATTTATGATTTAGCTTCTCAAGTATTAGAAAAATCTAAGGAGCTTGAT  300
Psoy_S  241 CATAAACAAATTATTTATGATTTAGCTTCTCAAGTATTAGAAAAATCTAAGGAGCTTGAT  300
Vok     241 CATAAACAAATTATTTATGATTTAGCTTTTCAAGTATTAGAAAGATCTAAGGAGCTTGAT  300
Cpac_S  241 CATAAGCAAATTACTTACGATTTGGCT---AAAGTATTAAAAAAATTTAAGGCATTTGAT  297
Cfau_S  241 CATAAGCAAATTACTTACGATTTGGCT---AAAGTATTAGGAAAATCTAAGGCATCTGAT  297
Cnau_S  241 CATAAGCAAATTACTTACGATTTGGCT---AAAGTATTAGAAAAATCTAAGGCATTCGAT  297
Pste_S  241 CATAAGCAAATTACTTACGATTTAGCT---AAAGTATTAGAAAAATCTAAGGCATTTGAT  297
Rma     241 TATAAACAAACTACTTATGACTTGGCTCTTAAAGTATTAGAAAAATCCAAAACACTTGAT  300
Ifos_S  241 CATAAGCAAATTACTTATGATTTGGCTCTTAAAGTATTAGAAAAATCCAAGACATTTGAT  300
Apha_S  241 CATAAGCAAATTACTTATGACTTGGCTCTTAAAGTATTAGAAAAATCCAAGATACTTGAT  300
Bsep_S  241 CATAAACAAACGACTTATGATTTGGCAGCAGCGGCAATGAGGCACTCGGATGAGCAAGGG  300
Akaw_S   81   H  K  Q  I  I  Y  D  L  A  F  Q  V  L  E  R  S  K  A  L  D  100
Clau_S   81   H  K  Q  I  I  Y  D  L  A  F  Q  V  L  E  R  S  K  T  L  D  100
Pkil_S   81   H  K  Q  I  I  Y  D  L  A  S  Q  V  L  E  K  S  K  E  L  D  100
Psoy_S   81   H  K  Q  I  I  Y  D  L  A  S  Q  V  L  E  K  S  K  E  L  D  100
Vok      81   H  K  Q  I  I  Y  D  L  A  F  Q  V  L  E  R  S  K  E  L  D  100
Cpac_S   81   H  K  Q  I  T  Y  D  L  A  #  K  V  L  K  K  F  K  A  F  D   99
Cfau_S   81   H  K  Q  I  T  Y  D  L  A  #  K  V  L  G  K  S  K  A  S  D   99
Cnau_S   81   H  K  Q  I  T  Y  D  L  A  #  K  V  L  E  K  S  K  A  F  D   99
Pste_S   81   H  K  Q  I  T  Y  D  L  A  #  K  V  L  E  K  S  K  A  F  D   99
Rma      81   Y  K  Q  T  T  Y  D  L  A  L  K  V  L  E  K  S  K  T  L  D  100
Ifos_S   81   H  K  Q  I  T  Y  D  L  A  L  K  V  L  E  K  S  K  T  F  D  100
Apha_S   81   H  K  Q  I  T  Y  D  L  A  L  K  V  L  E  K  S  K  I  L  D  100
Bsep_S   81   H  K  Q  T  T  Y  D  L  A  A  A  A  M  R  H  S  D  E  Q  G  100
```

```
Akaw_S  301 TGGCAATTATTAAACACACCTGAGCGATTTAAGATTTTAACGATTGATAGTTTGGCAGGT  360
Clau_S  301 TGGCAATTATTAAATACACCTGAGCGATTTAAGATTTTAACGATTGATAGTTTGTCACGT  360
Pkil_S  301 TGGCAATTATTAAACACACCTGAACGATTTAAGATTTTAACGATTGATAGTTTGTCAAGT  360
Psoy_S  301 TGGCAATTATTAAACACACCTGAACGATTTAAGATTTTAACGATTGATAGTTTGTCAAGT  360
Vok     301 TGGCAATTATTAAATACACCTGAACGATTTAAGATTTTAACGATTGATAGTTTATCAAGT  360
Cpac_S  298 TGGCAATTATTAAACACGCCTGAGCGACTTAAAATTTCAACGATTGATGGTTTGTCAAGT  357
Cfau_S  298 TGGCAATTACTAAACACGCCTGAACGGCTTAAAATTTCAACGATTGATGGTTTGTCAAGT  357
Cnau_S  298 TGGCAATTATTAAACACGCCTGAGCGGCTTAAAATTTCAACGATTGATAGTTTGTCAAGT  357
Pste_S  298 TGGCAATTATTAAACACGCCTGAGCGGCTTAAAATTTCAACGATTGATGGTTTGTCAAGT  357
Rma     301 TGGCAATTATTAAATATGTATGAACGGTTTAAAATCTTAACGATTGACGGTTTATCAGGT  360
Ifos_S  301 TGGCAATTATTAAACATGCCTGAGCGGTTTAAAATCTCAACGATTGATGGTCTGTCAAGT  360
Apha_S  301 TGGCAATTATTAAACACGCCTGAGCGGTTTAAAATCTCAACGATTGATGGTCTGTCAAGT  360
Bsep_S  301 TGGCAGTTGTTGCAAAATCCGAAACGCTTAAAAATCTCAACCATTGATGGGCTTTATAGT  360
Akaw_S  101   W  Q  L  L  N  T  P  E  R  F  K  I  L  T  I  D  S  L  A  G  120
Clau_S  101   W  Q  L  L  N  T  P  E  R  F  K  I  L  T  I  D  S  L  S  R  120
Pkil_S  101   W  Q  L  L  N  T  P  E  R  F  K  I  L  T  I  D  S  L  S  S  120
Psoy_S  101   W  Q  L  L  N  T  P  E  R  F  K  I  L  T  I  D  S  L  S  S  120
Vok     101   W  Q  L  L  N  T  P  E  R  F  K  I  L  T  I  D  S  L  S  S  120
Cpac_S  100   W  Q  L  L  N  T  P  E  R  L  K  I  S  T  I  D  G  L  S  S  119
Cfau_S  100   W  Q  L  L  N  T  P  E  R  L  K  I  S  T  I  D  G  L  S  S  119
Cnau_S  100   W  Q  L  L  N  T  P  E  R  L  K  I  S  T  I  D  S  L  S  S  119
Pste_S  100   W  Q  L  L  N  T  P  E  R  L  K  I  S  T  I  D  G  L  S  S  119
Rma     101   W  Q  L  L  N  M  Y  E  R  F  K  I  L  T  I  D  G  L  S  G  120
Ifos_S  101   W  Q  L  L  N  M  P  E  R  F  K  I  S  T  I  D  G  L  S  S  120
Apha_S  101   W  Q  L  L  N  T  P  E  R  F  K  I  S  T  I  D  G  L  S  S  120
Bsep_S  101   W  Q  L  L  Q  N  P  K  R  L  K  I  S  T  I  D  G  L  Y  S  120
```

```
Akaw_S  361 TTTATTACCAGTCGTTATCCAATTAAAGATCAACTAGTACCTAAGAAAGTTATATCGCAA  420
Clau_S  361 TTGATTACCAGTCGTTATCCAAGTAAAAATAAATTAGTGCCTAAGAAAGTTATATCGCAA  420
Pkil_S  361 TTGATTACCAGTCGTTATCCAAGTAAAAATCAATTAGTGCCTAAAAAAGTTATATCGCAA  420
Psoy_S  361 TTGATTACCAGTCGTTATCCAAGTAAAAATCAATTAGTGCCTAAAAAAGTTATATCGCAA  420
Vok     361 TTGATTACCAGTCACTATCCAAGTAAAAATCAATTAGTGCCTAAGAAAGTTATATCGCAA  420
Cpac_S  358 TTGATTACCAGTCGTTATCCAAGTAAGAATCAATTAGTGCCCAAGCAAGTCATAGCGCAA  417
Cfau_S  358 TTAATTACCAGTCGTTATCCAAGTAAGAATCAATTAGTGCCCAAGCAAATCATAGCTCAA  417
Cnau_S  358 TTGATTACCAGTCGTTATCCAAGTAAGAATCAATTAGTACCCAAGCAAATCATAGCGCAA  417
Pste_S  358 TTGATTACCAGTCGTTATCCAAGTAAAAATCAATTAGTGCCCAAGCAAATCATAGAGCAA  417
Rma     361 TTGATTGCCAGTCGTTATCCAAGTAAGAATCAATTAGTGCCCAAGCAAATCATAGCGCAA  420
Ifos_S  361 TTGATTACCAGTCGTTATCCAAGTAAGAATCAACTAATACCCAAGCAAATCATAGCTCAA  420
Apha_S  361 TTGATTATCAGTCGTTATCCAAGTAAGAATCAACTAGTACCCAAGCAAATCATAGCGCAA  420
Bsep_S  361 CTGATTACTAATCGTTATCCTTTGCCAGGTCAGTTGGTTCCGAGACAAATTATGGCGCAG  420
Akaw_S  121   F  I  T  S  R  Y  P  I  K  D  Q  L  V  P  K  K  V  I  S  Q  140
Clau_S  121   L  I  T  S  R  Y  P  S  K  N  K  L  V  P  K  K  V  I  S  Q  140
Pkil_S  121   L  I  T  S  R  Y  P  S  K  N  Q  L  V  P  K  K  V  I  S  Q  140
Psoy_S  121   L  I  T  S  R  Y  P  S  K  N  Q  L  V  P  K  K  V  I  S  Q  140
Vok     121   L  I  T  S  H  Y  P  S  K  N  Q  L  V  P  K  K  V  I  S  Q  140
Cpac_S  120   L  I  T  S  R  Y  P  S  K  N  Q  L  V  P  K  Q  V  I  A  Q  139
Cfau_S  120   L  I  T  S  R  Y  P  S  K  N  Q  L  V  P  K  Q  I  I  A  Q  139
Cnau_S  120   L  I  T  S  R  Y  P  S  K  N  Q  L  V  P  K  Q  I  I  A  Q  139
Pste_S  120   L  I  T  S  R  Y  P  S  K  N  Q  L  V  P  K  Q  I  I  E  Q  139
Rma     121   L  I  A  S  R  Y  P  S  K  N  Q  L  V  P  K  Q  I  I  A  Q  140
Ifos_S  121   L  I  T  S  R  Y  P  S  K  N  Q  L  I  P  K  Q  I  I  A  Q  140
Apha_S  121   L  I  I  S  R  Y  P  S  K  N  Q  L  V  P  K  Q  I  I  A  Q  140
Bsep_S  121   L  I  T  N  R  Y  P  L  P  G  Q  L  V  P  R  Q  I  M  A  Q  140
```

```
Akaw_S  421 AATTGGGAGCGATATTGTATGTATATTCAGGCCGCTAAGCAAACTTTATTAGCAATTGAT  480
Clau_S  421 AATTGGGCACGATATTGTATGTATTTTCAGGCTGCTAAGAAAACTTTATTAGCGGTTGAT  480
Pkil_S  421 AATTGGGCACGATATTGTATGTATTTTCAGGCCGCTAAGCAAACTTTATTAGCGATTGAT  480
Psoy_S  421 AATTGGGCACGATATTGTATGTATTTTCAGGCCGCTAAGCAAACTTTATTAGCGATTGAT  480
Vok     421 AATTGGGCACGATATTCTATGTATTCTCAGGCCGCTAAGCAAACTTTACTAGCGATTAAT  480
Cpac_S  418 AATTGGGCAAGAAATGATATTTATCTTCAAGCTGCCAAGCAGACATTATTGGCAATTGAT  477
Cfau_S  418 AACTGGGCAAGAAATGATATTTATCTTCAAGCTGCCAAGCAGACGTTATTGGCAATTGAT  477
Cnau_S  418 AACTGGGCAAGAAATGATATTTATCTTCAAGCTGCCAAGCAGACATTATTGGCAATTGAT  477
Pste_S  418 AACTGGGCAAGAAATGATATTTATCTTCAAGCTGCCAAGCAGACATTATTGGCAATTGAT  477
Rma     421 AATTGTGTAAGAAATGATATTTATCTTAAAGCCGCTAAGCAAACCTTATTAGCAATTGAT  480
Ifos_S  421 AATTGGGCAAGAAATGATATTTATCTTCAAGCTGCCAAGCAAACATTATTGGCAATTGAT  480
Apha_S  421 AACTGGGCAAGAAATGATGTTTATCTTCAAGCTGCGAAGCAAACATTATTGGCAATTGAT  480
Bsep_S  421 CAATGGGAGCGAAATAAAGCCTATCAGATAGCAGCACAGCAGACATTAATGTTGATTGAT  480
Akaw_S  141   N  W  E  R  Y  C  M  Y  I  Q  A  A  K  Q  T  L  L  A  I  D  160
Clau_S  141   N  W  A  R  Y  C  M  Y  F  Q  A  A  K  K  T  L  L  A  V  D  160
Pkil_S  141   N  W  A  R  Y  C  M  Y  F  Q  A  A  K  Q  T  L  L  A  I  D  160
Psoy_S  141   N  W  A  R  Y  C  M  Y  F  Q  A  A  K  Q  T  L  L  A  I  D  160
Vok     141   N  W  A  R  Y  S  M  Y  S  Q  A  A  K  Q  T  L  L  A  I  N  160
Cpac_S  140   N  W  A  R  N  D  I  Y  L  Q  A  A  K  Q  T  L  L  A  I  D  159
Cfau_S  140   N  W  A  R  N  D  I  Y  L  Q  A  A  K  Q  T  L  L  A  I  D  159
Cnau_S  140   N  W  A  R  N  D  I  Y  L  Q  A  A  K  Q  T  L  L  A  I  D  159
Pste_S  140   N  W  A  R  N  D  I  Y  L  Q  A  A  K  Q  T  L  L  A  I  D  159
Rma     141   N  C  V  R  N  D  I  Y  L  K  A  A  K  Q  T  L  L  A  I  D  160
Ifos_S  141   N  W  A  R  N  D  I  Y  L  Q  A  A  K  Q  T  L  L  A  I  D  160
Apha_S  141   N  W  A  R  N  D  V  Y  L  Q  A  A  K  Q  T  L  L  A  I  D  160
Bsep_S  141   Q  W  E  R  N  K  A  Y  Q  I  A  A  Q  Q  T  L  M  L  I  D  160
```

```
Akaw_S  481 GAACTTGAATATCAAGATAGTGTTGAGTCGATTCTTTTGTATTTGGATAATAATGTTGAT  540
Clau_S  481 GAACTTGAATATCAAGATAGTATTGAGTCGATTCTTTTATATTTGGATAATAATGTTGAT  540
Pkil_S  481 AAGCTTGAATATCAAGATAGTGTTGAGTCGATCCTTTTATATTTAGATAATAATGTTGAT  540
Psoy_S  481 AAGCTTGAATATCAAGATAGTGTTGAGTCGATCCTTTTATATTTAGATAATAATGTTGAT  540
Vok     481 GAACTTGAATATCAAGATAGTGTTGAGTCGATTCTTTTATATTTGGATAATAATATTGAT  540
Cpac_S  478 GAGTCTGAATATCAAAATAGTATTGAGTCGGTGCTTCTATATTTGGATAATAATGTTAAT  537
Cfau_S  478 GAGTCTGAATATCAAAATAGTATTGAGTCGGTGCTTCTATATTTGGATAATAATGTTAAT  537
Cnau_S  478 GAGCCCGAATATCAAAATAGTATTGAGTCGGTGCTTCTATATTTGGATAATAATGTTAAT  537
Pste_S  478 GAGCCTGAATATCAAAATAGTATTGAGTCGGTGCTTCTATATTTGGATAATAATGTTAAT  537
Rma     481 GAGTCTGAATATCAAAATAGTATTGAGTCGGTACTCCTATATTTGGATAATAATGTTAAT  540
Ifos_S  481 GAGTCTGAATATCAAAATAGCATTGAGTCAGTGCTGCTATATTTGGATAATAATGTTAAT  540
Apha_S  481 GAGCCTGAATATCAAAATAGCATTGAGTCGGTGCTGTTATATTTGGATAATAATGTTAAT  540
Bsep_S  481 GATGAGGAGTATGGCAAGGATATTCAAAATTTGTTGTTACACCTGGATAATAATGTCAGC  540
Akaw_S  161   E  L  E  Y  Q  D  S  V  E  S  I  L  L  Y  L  D  N  N  V  D  180
Clau_S  161   E  L  E  Y  Q  D  S  I  E  S  I  L  L  Y  L  D  N  N  V  D  180
Pkil_S  161   K  L  E  Y  Q  D  S  V  E  S  I  L  L  Y  L  D  N  N  V  D  180
Psoy_S  161   K  L  E  Y  Q  D  S  V  E  S  I  L  L  Y  L  D  N  N  V  D  180
Vok     161   E  L  E  Y  Q  D  S  V  E  S  I  L  L  Y  L  D  N  N  I  D  180
Cpac_S  160   E  S  E  Y  Q  N  S  I  E  S  V  L  L  Y  L  D  N  N  V  N  179
Cfau_S  160   E  S  E  Y  Q  N  S  I  E  S  V  L  L  Y  L  D  N  N  V  N  179
Cnau_S  160   E  P  E  Y  Q  N  S  I  E  S  V  L  L  Y  L  D  N  N  V  N  179
Pste_S  160   E  P  E  Y  Q  N  S  I  E  S  V  L  L  Y  L  D  N  N  V  N  179
Rma     161   E  S  E  Y  Q  N  S  I  E  S  V  L  L  Y  L  D  N  N  V  N  180
Ifos_S  161   E  S  E  Y  Q  N  S  I  E  S  V  L  L  Y  L  D  N  N  V  N  180
Apha_S  161   E  P  E  Y  Q  N  S  I  E  S  V  L  L  Y  L  D  N  N  V  N  180
Bsep_S  161   D  E  E  Y  G  K  D  I  Q  N  L  L  L  H  L  D  N  N  V  S  180
```

```
Akaw_S  541 AGATTTTATCAGTTAATTACGGATATGCTGGCTAAAAGAGATCAGTGGATTCTGAAATTG  600
Clau_S  541 AGATTTTATCAGTTGGTTACGGATATGCTGGCTAAAAGAGATCAGTGGATCTTGAAATTG  600
Pkil_S  541 AGATTTTATCAGTTGATTACGGATATGTTGGCTAAAAGAGATCAGTGGATTTTGAAATTG  600
Psoy_S  541 AGATTTTATCAGTTGATTACGGATATGTTGGCTAAAAGAGATCAGTGGATTTTGAAATTG  600
Vok     541 AGATTTTATCAGTTGATTACGGACATGTTGGCTAAAAGAGATCAGTGGATTTTGAAATTG  600
Cpac_S  538 AAGTTTTATCGGTTGATTACAGATATGCTGGCTAAGCGTGATCAGTGGCTTTTGAAATTG  597
Cfau_S  538 AAGTTTTATCGGTTGATTACAGATATGCTGGCTAAGCGTGATCAGTGGCTTTTGAAATTG  597
Cnau_S  538 AAGTTTTATCGGTTGATTACAGATATGCTGGCTAAGCGTGATCAGTGGCTTTTGAAGTTG  597
Pste_S  538 AAGTTTTATCGGTTGATTACAGATATGCTGGCTAAGCGTGATCAGTGGCTTTTGAAGTTG  597
Rma     541 AAATTTTATCGTTTGATTATAGATATGCTGGCTAAACGTGACCAGTGGCTTTTGAAGCTG  600
Ifos_S  541 AAGTTTTATCGTTTGATTACGGACATGTTGGCTAAGCGTGATCAGTGGCTTTTGAAGTTG  600
Apha_S  541 AAGTTTTATCGTTTGATTACGGATATGCTGGCTAAGCGTGATCAGTGGCTTTTGAAGTTG  600
Bsep_S  541 AAGTTTGAGAGCTTGGTGGTGCAGATGCTGTCGAAGCGGGATCAATGGTTGGGGCGCTTG  600
Akaw_S  181   R  F  Y  Q  L  I  T  D  M  L  A  K  R  D  Q  W  I  L  K  L  200
Clau_S  181   R  F  Y  Q  L  V  T  D  M  L  A  K  R  D  Q  W  I  L  K  L  200
Pkil_S  181   R  F  Y  Q  L  I  T  D  M  L  A  K  R  D  Q  W  I  L  K  L  200
Psoy_S  181   R  F  Y  Q  L  I  T  D  M  L  A  K  R  D  Q  W  I  L  K  L  200
Vok     181   R  F  Y  Q  L  I  T  D  M  L  A  K  R  D  Q  W  I  L  K  L  200
Cpac_S  180   K  F  Y  R  L  I  T  D  M  L  A  K  R  D  Q  W  L  L  K  L  199
Cfau_S  180   K  F  Y  R  L  I  T  D  M  L  A  K  R  D  Q  W  L  L  K  L  199
Cnau_S  180   K  F  Y  R  L  I  T  D  M  L  A  K  R  D  Q  W  L  L  K  L  199
Pste_S  180   K  F  Y  R  L  I  T  D  M  L  A  K  R  D  Q  W  L  L  K  L  199
Rma     181   K  F  Y  R  L  I  I  D  M  L  A  K  R  D  Q  W  L  L  K  L  200
Ifos_S  181   K  F  Y  R  L  I  T  D  M  L  A  K  R  D  Q  W  L  L  K  L  200
Apha_S  181   K  F  Y  R  L  I  T  D  M  L  A  K  R  D  Q  W  L  L  K  L  200
Bsep_S  181   K  F  E  S  L  V  V  Q  M  L  S  K  R  D  Q  W  L  G  R  L  200
```

```
Akaw_S  601 TATCAACACGATACGCTTAATGTTGAAACCTTACGGATAAGTTCTGAAAAGGTTATTATT  660
Clau_S  601 TATCAACATGGTACGCTTAATATTGAAACTTTGCGGCTAAGTTCTGAAAAGGTTATCATT  660
Pkil_S  601 TATCAACATGGTACGCTTAATATTGAAACCTTGCGGCTAAGTTCTGAAAAGATTATTATT  660
Psoy_S  601 TATCAACATGGTACGCTTAATATTGAAACCTTGCGGCTAAGTTCTGAAAAGATTATTATT  660
Vok     601 TATCAACATGGTACGCTTAATATTGAAACCTTGCGGCTAAGTTCTGAAAAGGTTATTATT  660
Cpac_S  598 TATCAACATGGTGTACTTAATATTGAAACCTTGCAGTTGAGTTCTGAAAGGGTTATCATT  657
Cfau_S  598 TACCAACATGGTGTACTTAATATTGAAACCTTGCAGTTGAGTTCTGAAAGGGTTATCACT  657
Cnau_S  598 TATCAACATGGTGTACTTAATATTGAAACCTTGCAGTTGAGTTCTGAGAGGGTTATCATC  657
Pste_S  598 TATCAACATGGTGTACTTAATATTGAAACCCTGCAGTTGAGTTCTGAAAGGGTTATCACC  657
Rma     601 TATCAGCATGGAGTGCTTAATATTGAAACTTTGAGGTTAAGCTCTAAAAAGATTATTATC  660
Ifos_S  601 TACCAGCATGGTGTGCTTAATATTAAAACCTTACGGTTGAGTTCTGAAAGGGTGATTACG  660
Apha_S  601 TACCAGCATGGCGCGCTTAATATTAAAACCTTGCGGTTGAGTTCTGAAAGGGTGATTACA  660
Bsep_S  601 TATCGGGATAATGTGCTAGATTTACAAATATTGCAAGACAGTGCTAGAACGATTGTGAAA  660
Akaw_S  201   Y  Q  H  D  T  L  N  V  E  T  L  R  I  S  S  E  K  V  I  I  220
Clau_S  201   Y  Q  H  G  T  L  N  I  E  T  L  R  L  S  S  E  K  V  I  I  220
Pkil_S  201   Y  Q  H  G  T  L  N  I  E  T  L  R  L  S  S  E  K  I  I  I  220
Psoy_S  201   Y  Q  H  G  T  L  N  I  E  T  L  R  L  S  S  E  K  I  I  I  220
Vok     201   Y  Q  H  G  T  L  N  I  E  T  L  R  L  S  S  E  K  V  I  I  220
Cpac_S  200   Y  Q  H  G  V  L  N  I  E  T  L  Q  L  S  S  E  R  V  I  I  219
Cfau_S  200   Y  Q  H  G  V  L  N  I  E  T  L  Q  L  S  S  E  R  V  I  T  219
Cnau_S  200   Y  Q  H  G  V  L  N  I  E  T  L  Q  L  S  S  E  R  V  I  I  219
Pste_S  200   Y  Q  H  G  V  L  N  I  E  T  L  Q  L  S  S  E  R  V  I  T  219
Rma     201   Y  Q  H  G  V  L  N  I  E  T  L  R  L  S  S  K  K  I  I  I  220
Ifos_S  201   Y  Q  H  G  V  L  N  I  K  T  L  R  L  S  S  E  R  V  I  T  220
Apha_S  201   Y  Q  H  G  A  L  N  I  K  T  L  R  L  S  S  E  R  V  I  T  220
Bsep_S  201   Y  R  D  N  V  L  D  L  Q  I  L  Q  D  S  A  R  T  I  V  K  220
```

```
Akaw_S  661 CAACATTTATTTCTGTTAAAAAATGAAGTAGAATATTATTTTGATCCAACTTTTTTTAAA  720
Clau_S  661 CAACATTTATTTCTGTTAAAAAATGAAGTAGAATATTATTTTGATCCAACTTTTTTTAAA  720
Pkil_S  661 CAACATTTATTTCTGTTAAAAAATGAAGTAGAATATTATTTTGATCCAACTTTTTTTAAA  720
Psoy_S  661 CAACATTTATTTCTGTTAAAAAATGAAGTAGAATATTATTTTGATCCAACTTTTTTTAAA  720
Vok     661 CAACATTTATCTCTGTTAAAAAATGAAGTAGAATATTATTTTGATCCAATTTTTTTTAAA  720
Cpac_S  658 CAACACTTATTGTTGTTAAAAAATGAGGCAGACTGTTATTTTGATGCTACTTTTTTTGAA  717
Cfau_S  658 CAACATTTATTGTTGTTAAAAAATGAGGCAGACTGTTATTTTGATGCTACTTTTTTTGAA  717
Cnau_S  658 CAACACTTATTGTTGTTAAAAAATGAGGCAGACTGTTATTTTGATGCTACTTTTTTTAAA  717
Pste_S  658 CAACACTTATTGTTGTTAAAAAATGAGGCGGACTGTTATTTTGATGCTACTTTTTTTGAA  717
Rma     661 CAACATTTGTTGCTGTTAAAAAATGAGGTTGAGCGCTATTTTAATGTTACTTTTTTTAAA  720
Ifos_S  661 CAGCACTTACAATTGTTAAAAAATGAGGCTAAGCACCACCTTGATGGTGCTTTTTTTGAC  720
Apha_S  661 CAGCACCTACAGTTGTTAAAAAATGAGGCTAAGCACCACCTTGATGGTGCTTTTTTTGAC  720
Bsep_S  661 CAATATTTTGAGTATTTACAACCATTGGCTAAAGTGTATTTAGATGATGAATTTTTTGGA  720
Akaw_S  221   Q  H  L  F  L  L  K  N  E  V  E  Y  Y  F  D  P  T  F  F  K  240
Clau_S  221   Q  H  L  F  L  L  K  N  E  V  E  Y  Y  F  D  P  T  F  F  K  240
Pkil_S  221   Q  H  L  F  L  L  K  N  E  V  E  Y  Y  F  D  P  T  F  F  K  240
Psoy_S  221   Q  H  L  F  L  L  K  N  E  V  E  Y  Y  F  D  P  T  F  F  K  240
Vok     221   Q  H  L  S  L  L  K  N  E  V  E  Y  Y  F  D  P  I  F  F  K  240
Cpac_S  220   Q  H  L  L  L  L  K  N  E  A  D  C  Y  F  D  A  T  F  F  E  239
Cfau_S  220   Q  H  L  L  L  L  K  N  E  A  D  C  Y  F  D  A  T  F  F  E  239
Cnau_S  220   Q  H  L  L  L  L  K  N  E  A  D  C  Y  F  D  A  T  F  F  K  239
Pste_S  220   Q  H  L  L  L  L  K  N  E  A  D  C  Y  F  D  A  T  F  F  E  239
Rma     221   Q  H  L  L  L  L  K  N  E  V  E  R  Y  F  N  V  T  F  F  K  240
Ifos_S  221   Q  H  L  Q  L  L  K  N  E  A  K  H  H  L  D  G  A  F  F  D  240
Apha_S  221   Q  H  L  Q  L  L  K  N  E  A  K  H  H  L  D  G  A  F  F  D  240
Bsep_S  221   Q  Y  F  E  Y  L  Q  P  L  A  K  V  Y  L  D  D  E  F  F  G  240
```

```
Akaw_S  721 TTGTTAAAATATAATACTAAGCCAGAATTTGCTCATATTCAAGGCGTGCCAGATGCCAAT  780
Clau_S  721 TTGTTAAAATATAATTCTAAGCTAGAATTTGCTCAGATTCAATGCGTGCCAGATGCCACT  780
Pkil_S  721 TTGTTAAAATATAACTCTAAGCTAGAATTTGCTCAGATTCAAGACGTGCCAGATGCCACT  780
Psoy_S  721 TTGTTAAAATATAACTCTAAGCTAGAATTTGCTCAGATTCAAGACGTGCCAGATGCCACT  780
Vok     721 TTGTTAAAATATAATTCTAAGCTAGAATTTGCTCAGATTAAAGACGTGCCAGATGCCACT  780
Cpac_S  718 CTGTTAAAAGATAATACTAAGCCAGAGCTTATGAAAATTCAAGGTGTGCCAGATGCCACT  777
Cfau_S  718 CTGTTAAAAGATAATACTAAGCCAGAGCTTATGAAAATTCAAAGTGTGCCAGATGCCACT  777
Cnau_S  718 CTGTTAAAAGATAATACTAAGCCAGAGCTTATGCAAATTCAAGGTGTGCCAGATGCCACT  777
Pste_S  718 CTGTTAAAAGATAATACTAAGCCAGAGCTTATGAAAATTCAAGGTGTGCCAGATGCCACT  777
Rma     721 CTGTTAAAAGATAACACTAAACCGGAATTTGCACAAATTCAAGGTGTACCAGATGCCACT  780
Ifos_S  721 TTATTAAAATATAATACTCAATCTAAATTTTCGCAAATACAGGCGCTGCCAAATACAAGT  780
Apha_S  721 TTATTAAAATATAATATTCAATCTAAATTTTCGCAAATACAGGCACTGCCAAATACAAGT  780
Bsep_S  721 TTGTTGTCTGCTCGTGAAGGT------------AAGTTATACACCCTGCCAACGACTAAT  768
Akaw_S  241   L  L  K  Y  N  T  K  P  E  F  A  H  I  Q  G  V  P  D  A  N  260
Clau_S  241   L  L  K  Y  N  S  K  L  E  F  A  Q  I  Q  C  V  P  D  A  T  260
Pkil_S  241   L  L  K  Y  N  S  K  L  E  F  A  Q  I  Q  D  V  P  D  A  T  260
Psoy_S  241   L  L  K  Y  N  S  K  L  E  F  A  Q  I  Q  D  V  P  D  A  T  260
Vok     241   L  L  K  Y  N  S  K  L  E  F  A  Q  I  K  D  V  P  D  A  T  260
Cpac_S  240   L  L  K  D  N  T  K  P  E  L  M  K  I  Q  G  V  P  D  A  T  259
Cfau_S  240   L  L  K  D  N  T  K  P  E  L  M  K  I  Q  S  V  P  D  A  T  259
Cnau_S  240   L  L  K  D  N  T  K  P  E  L  M  Q  I  Q  G  V  P  D  A  T  259
Pste_S  240   L  L  K  D  N  T  K  P  E  L  M  K  I  Q  G  V  P  D  A  T  259
Rma     241   L  L  K  D  N  T  K  P  E  F  A  Q  I  Q  G  V  P  D  A  T  260
Ifos_S  241   L  L  K  Y  N  T  Q  S  K  F  S  Q  I  Q  A  L  P  N  T  S  260
Apha_S  241   L  L  K  Y  N  I  Q  S  K  F  S  Q  I  Q  A  L  P  N  T  S  260
Bsep_S  241   L  L  S  A  R  E  G  #  #  #  #  K  L  Y  T  L  P  T  T  N  256
```

```
Akaw_S  781 ATTAAATCGCTAGAAATTTGGAAAAATTTAAGTAGGTTATGTTTGACAGCACAAGGC---  837
Clau_S  781 ATTAAATCGCTAGAAGTTTGGAAAAATTTGAGCAGGTTATGTTTGACGGTACAAGGC---  837
Pkil_S  781 ATTAAATCGCTAGAAGTTTGGAAAAATTTGAGTCGGTTATGTTTGACGGTACAGGGT---  837
Psoy_S  781 ATTAAATCGCTAGAAGTTTGGAAAAATTTGAGTCGGTTATGTTTGACGGTACAGGGT---  837
Vok     781 ATTAAATCGCTAGAAGTTTGGAAAAATTTGAGTCGGTTATGTTTGACGGTACAAGGT---  837
Cpac_S  778 ATTGAATCGCTAGAGGCTTGGAAAAGCTTGTGTCAGCTGTGTTTAACCACTCAGGGC---  834
Cfau_S  778 ATTGAATCGCTAGAGACTTGGAAAGACTTGTGTCAGCTGTGTTTAACCACTCAGGGT---  834
Cnau_S  778 ATTGAATCGCTAGAGGCTTGGAAAAACTTGTGTCAGCTGTGTTTAACTACTCAGGGC---  834
Pste_S  778 ATTGAATCGCTAGAGGCCTGGAAAAACTTGTGTCAGCTGTGTTTAACCACTCAGGGC---  834
Rma     781 GTTGAATCGTTAGAGGTCTGGAAAAACTTATGTCAGCTATGTTTGACTACGCAAGGT---  837
Ifos_S  781 ATTGAGTGTTTAGATGTATGGGTAAATATAGCAGACCTATTGCTTGATGGTAAAAGCAAA  840
Apha_S  781 ATTGGGTGCTTAGATGTATGGGCAAATATAGCAGACCTATTGCTTGATGGTAAAAGCAAA  840
Bsep_S  769 TTTTCGGATTTGGAGGCGTGGCAGACCATTGCAGATTTATGTTTGACCAAAAAAGGC---  825
Akaw_S  261   I  K  S  L  E  I  W  K  N  L  S  R  L  C  L  T  A  Q  G  #  279
Clau_S  261   I  K  S  L  E  V  W  K  N  L  S  R  L  C  L  T  V  Q  G  #  279
Pkil_S  261   I  K  S  L  E  V  W  K  N  L  S  R  L  C  L  T  V  Q  G  #  279
Psoy_S  261   I  K  S  L  E  V  W  K  N  L  S  R  L  C  L  T  V  Q  G  #  279
Vok     261   I  K  S  L  E  V  W  K  N  L  S  R  L  C  L  T  V  Q  G  #  279
Cpac_S  260   I  E  S  L  E  A  W  K  S  L  C  Q  L  C  L  T  T  Q  G  #  278
Cfau_S  260   I  E  S  L  E  T  W  K  D  L  C  Q  L  C  L  T  T  Q  G  #  278
Cnau_S  260   I  E  S  L  E  A  W  K  N  L  C  Q  L  C  L  T  T  Q  G  #  278
Pste_S  260   I  E  S  L  E  A  W  K  N  L  C  Q  L  C  L  T  T  Q  G  #  278
Rma     261   V  E  S  L  E  V  W  K  N  L  C  Q  L  C  L  T  T  Q  G  #  279
Ifos_S  261   I  E  C  L  D  V  W  V  N  I  A  D  L  L  L  D  G  K  S  K  280
Apha_S  261   I  G  C  L  D  V  W  A  N  I  A  D  L  L  L  D  G  K  S  K  280
Bsep_S  257   F  S  D  L  E  A  W  Q  T  I  A  D  L  C  L  T  K  K  G  #  275
```

```
Akaw_S  838 ---------AAGTGGCGTTCATCATTAAATAAAAATAATGGTTTTCCTGCAGAGTTAAAA  888
Clau_S  838 ---------AGGTGGCGTTCATCATTGAATAAAAATAATGGTTTTCCTGTAGAGTTAAAA  888
Pkil_S  838 ---------AAGTGGCGTTCATCATTGAATAAAAATAATGGTTTTCCTGCAGAATTAAAA  888
Psoy_S  838 ---------AAGTGGCGTTCATCATTGAATAAAAATAATGGTTTTCCTGCAGAATTAAAA  888
Vok     838 ---------AAGTGGCGTTCATCATTGAATAAAAATAATGGTTTTCCTGCAGAGTTAAAA  888
Cpac_S  835 ---------AAGTGGCGTACATCGTTGAATAAAAATAACGGCTTTCCCACAGAGTTAAAA  885
Cfau_S  835 ---------AAGTGGCGTACATCATTGAATAAAAATAACGGTTTTCCCACAGAGTTAAAA  885
Cnau_S  835 ---------AAATGGCGTACATCATTGAATAAAAATAACGGCTTTCCCCCAGAGTTAAAA  885
Pste_S  835 ---------AAGTGGCGTACATCATTGAATAAAAATAACGGCTTTCCCACAGAGTTAAAA  885
Rma     838 ---------AAGTGGCGTACATCATTGAATAAAAATAACGGTTTTCCCGTAGAATTAAAA  888
Ifos_S  841 AAAAACAATAAGTGGCGCAAAAGTGTTAATACAAACAATGGATTTCCAGCTGAAGTAAAG  900
Apha_S  841 AAAAACAATAAGTGGCGTAAAAGTGTTAATACGAACAATGGATTTCCAGCTGAAGTGAAA  900
Bsep_S  826 ---------ACATGGCGTACAGCAGTTAATAAA---------------------CTCAAG  855
Akaw_S  280   #  #  #  K  W  R  S  S  L  N  K  N  N  G  F  P  A  E  L  K  296
Clau_S  280   #  #  #  R  W  R  S  S  L  N  K  N  N  G  F  P  V  E  L  K  296
Pkil_S  280   #  #  #  K  W  R  S  S  L  N  K  N  N  G  F  P  A  E  L  K  296
Psoy_S  280   #  #  #  K  W  R  S  S  L  N  K  N  N  G  F  P  A  E  L  K  296
Vok     280   #  #  #  K  W  R  S  S  L  N  K  N  N  G  F  P  A  E  L  K  296
Cpac_S  279   #  #  #  K  W  R  T  S  L  N  K  N  N  G  F  P  T  E  L  K  295
Cfau_S  279   #  #  #  K  W  R  T  S  L  N  K  N  N  G  F  P  T  E  L  K  295
Cnau_S  279   #  #  #  K  W  R  T  S  L  N  K  N  N  G  F  P  P  E  L  K  295
Pste_S  279   #  #  #  K  W  R  T  S  L  N  K  N  N  G  F  P  T  E  L  K  295
Rma     280   #  #  #  K  W  R  T  S  L  N  K  N  N  G  F  P  V  E  L  K  296
Ifos_S  281   K  N  N  K  W  R  K  S  V  N  T  N  N  G  F  P  A  E  V  K  300
Apha_S  281   K  N  N  K  W  R  K  S  V  N  T  N  N  G  F  P  A  E  V  K  300
Bsep_S  276   #  #  #  T  W  R  T  A  V  N  K  #  #  #  #  #  #  #  L  K  285
```

```
Akaw_S  889 ATGCAAAAACGATCTATTATCAAAATATTTCAATCTTTATCTAGTCATCAACAATTAAGA  948
Clau_S  889 ATACAGAAACGAGCTATTATCAAAATATTTCAATCTTTATCTAGTCATGAACAATTAAGA  948
Pkil_S  889 ATGCAAAAACTAGCTATTATCAAAATATTTCAATCTTTATCTAGTCATCAACAATTAAGA  948
Psoy_S  889 ATGCAAAAACTAGCTATTATCAAAATATTTCAATCTTTATCTAGTCATCAACAATTAAGA  948
Vok     889 ATGCAAAAACTAGCTATTATCAAAATATTTCAATCTTTATCTAGTCATCAACAATTAAGA  948
Cpac_S  886 ACGCAAAAACAATCCATTATTAAAATTTTTCAAGATTTGTCTAGCCAACAGCAATTAAAG  945
Cfau_S  886 ACGCAAAAACAATCCATTATTAAAATTTTTCAAGATTTGTCTAGCCAACAGCAATTAAAG  945
Cnau_S  886 ACGCAAAAACAATCCATTATTAAAATTTTTCAAGACTTGTCTAGCCAACGGCAATTAAAG  945
Pste_S  886 ACGCAAAAACAATCCATTATTAAAATTTTTCAAGACTTGTCTAGCCAACAGCAATTAAAG  945
Rma     889 ACACAAAAACAAGCTGTTATTAAAATTTTTCAAGACTTATCTAGCCATCAGCAATTAAGG  948
Ifos_S  901 GCACAAAAAGACGAGTTTATTAAATTTTTAAACCATCTTCAAGGTAAAGATAATTTCAAA  960
Apha_S  901 GCACAAAAAGACGAGTTTATTAAATTTTTAAACCATTTTCAAGGTAAAGATAATTTTAAA  960
Bsep_S  856 ------------------------------GAAAACTTGTCCGAACAAAAAGCACTTGGC  885
Akaw_S  297   M  Q  K  R  S  I  I  K  I  F  Q  S  L  S  S  H  Q  Q  L  R  316
Clau_S  297   I  Q  K  R  A  I  I  K  I  F  Q  S  L  S  S  H  E  Q  L  R  316
Pkil_S  297   M  Q  K  L  A  I  I  K  I  F  Q  S  L  S  S  H  Q  Q  L  R  316
Psoy_S  297   M  Q  K  L  A  I  I  K  I  F  Q  S  L  S  S  H  Q  Q  L  R  316
Vok     297   M  Q  K  L  A  I  I  K  I  F  Q  S  L  S  S  H  Q  Q  L  R  316
Cpac_S  296   T  Q  K  Q  S  I  I  K  I  F  Q  D  L  S  S  Q  Q  Q  L  K  315
Cfau_S  296   T  Q  K  Q  S  I  I  K  I  F  Q  D  L  S  S  Q  Q  Q  L  K  315
Cnau_S  296   T  Q  K  Q  S  I  I  K  I  F  Q  D  L  S  S  Q  R  Q  L  K  315
Pste_S  296   T  Q  K  Q  S  I  I  K  I  F  Q  D  L  S  S  Q  Q  Q  L  K  315
Rma     297   T  Q  K  Q  A  V  I  K  I  F  Q  D  L  S  S  H  Q  Q  L  R  316
Ifos_S  301   A  Q  K  D  E  F  I  K  F  L  N  H  L  Q  G  K  D  N  F  K  320
Apha_S  301   A  Q  K  D  E  F  I  K  F  L  N  H  F  Q  G  K  D  N  F  K  320
Bsep_S  286   #  #  #  #  #  #  #  #  #  #  E  N  L  S  E  Q  K  A  L  G  295
```

```
Akaw_S  949 GAGTTATTAGCAGGAGTTGAACACTTGCCTGATATTAATTTTTCAACTGATCAAATTAAT 1008
Clau_S  949 GAGTTATTATCAGGGGTTGAACATTTGCCTGATATTAATTTTTCAACTGATCAAATTAAT 1008
Pkil_S  949 GAGTTATTAGCGGGGGTTGAACATTTGCCTGATATTAATTTTTCAACTGATCAAATTAAT 1008
Psoy_S  949 GAGTTATTAGCGGGGGTTGAACATTTGCCTGATATTAATTTTTCAACTGATCAAATTAAT 1008
Vok     949 GAGTTATTAGCAGGGGTTGAATATTTGCCTGATATTAATTTTTCAACTGATCAAATTAAT 1008
Cpac_S  946 GAATTATTAGCAGGGGTTGAACAATTGCCTGATGTTGATTTTTCAACTAATCAAATTAAT 1005
Cfau_S  946 GAATTATTAGCAGGGGTTAAACAATTGCCTGATGTTGATTTTTCAACCAATCAAATTAAT 1005
Cnau_S  946 GAATTATTAGCAGGGGTTGAACAATTGCCTGATATTGATTTTTCAACCAATCAAATTAAT 1005
Pste_S  946 GAATTATTAGCAGGGGTTGAACAATTGCCTGATGTTGATTTTTCAACCAATCAAATTAAT 1005
Rma     949 AAATTATTAGCAGGAGTTGAGCAATTACCTGATGTGAACTTTTCAACCAATCAAATTAAT 1008
Ifos_S  961 GATTTGTTATTTGAGACACTTAATTTACCTGATGTTGATTTTTCAATCAATCAAATTAAC 1020
Apha_S  961 GATTTGTTATTTGAGACATTTAACTTGCCCGATGTTGATTTTTCAACCAATCAAATTAAT 1020
Bsep_S  886 GAAGCATTGCAAGGCTTAAGGAGTTTGCCAAATATTGATTTTTCGCAAGCACAGGCGGAT  945
Akaw_S  317   E  L  L  A  G  V  E  H  L  P  D  I  N  F  S  T  D  Q  I  N  336
Clau_S  317   E  L  L  S  G  V  E  H  L  P  D  I  N  F  S  T  D  Q  I  N  336
Pkil_S  317   E  L  L  A  G  V  E  H  L  P  D  I  N  F  S  T  D  Q  I  N  336
Psoy_S  317   E  L  L  A  G  V  E  H  L  P  D  I  N  F  S  T  D  Q  I  N  336
Vok     317   E  L  L  A  G  V  E  Y  L  P  D  I  N  F  S  T  D  Q  I  N  336
Cpac_S  316   E  L  L  A  G  V  E  Q  L  P  D  V  D  F  S  T  N  Q  I  N  335
Cfau_S  316   E  L  L  A  G  V  K  Q  L  P  D  V  D  F  S  T  N  Q  I  N  335
Cnau_S  316   E  L  L  A  G  V  E  Q  L  P  D  I  D  F  S  T  N  Q  I  N  335
Pste_S  316   E  L  L  A  G  V  E  Q  L  P  D  V  D  F  S  T  N  Q  I  N  335
Rma     317   K  L  L  A  G  V  E  Q  L  P  D  V  N  F  S  T  N  Q  I  N  336
Ifos_S  321   D  L  L  F  E  T  L  N  L  P  D  V  D  F  S  I  N  Q  I  N  340
Apha_S  321   D  L  L  F  E  T  F  N  L  P  D  V  D  F  S  T  N  Q  I  N  340
Bsep_S  296   E  A  L  Q  G  L  R  S  L  P  N  I  D  F  S  Q  A  Q  A  D  315
```

```
Akaw_S 1009 GCTCTGCAAGATATTGCACAAGTATTAAAGTTGGCTGTTTCTCAATTGAAAATTTTATTT 1068
Clau_S 1009 GCTCTGCAAGATATTGCACAAGTATTAAAGTTAGCTGTTTCTCAATTGAAAATTTTATTT 1068
Pkil_S 1009 GCTCTGCAAGATATTGCACAAGTATTAAAGTTGGCTGTTTCTCAATTGAAAATTTTATTT 1068
Psoy_S 1009 GCTCTGCAAGATATTGCACAAGTATTAAAGTTGGCTGTTTCTCAATTGAAAATTTTATTT 1068
Vok    1009 GTTCTGCAAGATATTGCACAAGTATTAAAGTTGGCTGTTTCTCAATTGAAAATTTTATTT 1068
Cpac_S 1006 GTCCTTCAAGACATTGCACAAGTATTAAAGTTAGCTGTTGCTCAATTAAATATTTTGTTT 1065
Cfau_S 1006 GCCCTTCAAGACATTGCACAAGTATTAAAGTTAGCTGTTACTCAATTAAATATTTTGTTT 1065
Cnau_S 1006 GCTCTTCAAGACATTGCACAAGTATTAAAGTTAGCTGTTGTTCAATTAAATATTTTGTTT 1065
Pste_S 1006 GCCCTTCAAGACATTGCACAAGTATTAAAGCTAGCTGTTGCTCAATTAAATATTTTGTTT 1065
Rma    1009 ATCCTTCAAGATATTGCACAAGTATTAAAGTTGGCTGTTATTCAATTAAATATTTTGTTT 1068
Ifos_S 1021 GCCCTTCAAGACATTGCACAAGTATTAAAGTTGGCCGTTGCTCAATTAAATGTTTTGTTT 1080
Apha_S 1021 GCCCTCCAAGACATTGCACAAGTATTAAAGTTGGCTGTTGCTCAATTAAATGTTTTGTTT 1080
Bsep_S  946 ATTTTGCAGACCATTGCACAGGTATTAAAATTATCTGTGGCACAACTCAATATCTATTTT 1005
Akaw_S  337   A  L  Q  D  I  A  Q  V  L  K  L  A  V  S  Q  L  K  I  L  F  356
Clau_S  337   A  L  Q  D  I  A  Q  V  L  K  L  A  V  S  Q  L  K  I  L  F  356
Pkil_S  337   A  L  Q  D  I  A  Q  V  L  K  L  A  V  S  Q  L  K  I  L  F  356
Psoy_S  337   A  L  Q  D  I  A  Q  V  L  K  L  A  V  S  Q  L  K  I  L  F  356
Vok     337   V  L  Q  D  I  A  Q  V  L  K  L  A  V  S  Q  L  K  I  L  F  356
Cpac_S  336   V  L  Q  D  I  A  Q  V  L  K  L  A  V  A  Q  L  N  I  L  F  355
Cfau_S  336   A  L  Q  D  I  A  Q  V  L  K  L  A  V  T  Q  L  N  I  L  F  355
Cnau_S  336   A  L  Q  D  I  A  Q  V  L  K  L  A  V  V  Q  L  N  I  L  F  355
Pste_S  336   A  L  Q  D  I  A  Q  V  L  K  L  A  V  A  Q  L  N  I  L  F  355
Rma     337   I  L  Q  D  I  A  Q  V  L  K  L  A  V  I  Q  L  N  I  L  F  356
Ifos_S  341   A  L  Q  D  I  A  Q  V  L  K  L  A  V  A  Q  L  N  V  L  F  360
Apha_S  341   A  L  Q  D  I  A  Q  V  L  K  L  A  V  A  Q  L  N  V  L  F  360
Bsep_S  316   I  L  Q  T  I  A  Q  V  L  K  L  S  V  A  Q  L  N  I  Y  F  335
```

```
Akaw_S 1069 AATGTTAATCAAACGCATGATTTTATTCAAGTTTCTTTGGATGCAGACCAGGCTTTAGAT 1128
Clau_S 1069 GATGTTAATCAAACGCATGATTTTATTCAAGTTTCTTTAGATGCTGATCAGGCTTTAGAT 1128
Pkil_S 1069 GATGTTAATCAAACGCATGATTTTATTCAAGTTTCCTTGGATGCAGACCAGGCTTTAGAT 1128
Psoy_S 1069 GATGTTAATCAAACGCATGATTTTATTCAAGTTTCCTTGGATGCAGACCAGGCTTTAGAT 1128
Vok    1069 GATGTTAATCAAACGCATGATTTTATTCAAGTTTCCTTGGATGCAGACCAGGCTTTAGAT 1128
Cpac_S 1066 GATGTTAATCAAACGCATGATTTTATCCAAGTTGCTTTGGATGCAGACCAAGCGTTAGAT 1125
Cfau_S 1066 GATGTTCATCAAACGCATGATTTTATCCAAGTTGCCTTGGATGCAGAGCAAGCGTTTGAT 1125
Cnau_S 1066 GATGTTAATCAAATGCATGATTTTATCCAAGTTGCCTTGGATGCAGACCAAGCGCTAGAT 1125
Pste_S 1066 GGTGTTAATCAAACGCATGATTTTATCCAAGTTGCCTTGGATGCAGACCAAGCGCTAGAT 1125
Rma    1069 GATGTTAATCAAACGTATGATTTCATCCAGGTTGCTTTAGATGCAAATCAGGCACTAGAT 1128
Ifos_S 1081 GATGTTAATCAAACGCATGATTTTATCCAAGTTGCCCTAGATGCAGACCATGCGTTAGAT 1140
Apha_S 1081 GATGTTAATCAAACGCATGATTTTATTCAGGTTGCCTTGGATGCAGATCAGGCTTTAGAT 1140
Bsep_S 1006 GAAGCGCAGCAAGCACACGATTTTATTGAGGTGGCGTTGAATGCTAATCAGGCATTGGAC 1065
Akaw_S  357   N  V  N  Q  T  H  D  F  I  Q  V  S  L  D  A  D  Q  A  L  D  376
Clau_S  357   D  V  N  Q  T  H  D  F  I  Q  V  S  L  D  A  D  Q  A  L  D  376
Pkil_S  357   D  V  N  Q  T  H  D  F  I  Q  V  S  L  D  A  D  Q  A  L  D  376
Psoy_S  357   D  V  N  Q  T  H  D  F  I  Q  V  S  L  D  A  D  Q  A  L  D  376
Vok     357   D  V  N  Q  T  H  D  F  I  Q  V  S  L  D  A  D  Q  A  L  D  376
Cpac_S  356   D  V  N  Q  T  H  D  F  I  Q  V  A  L  D  A  D  Q  A  L  D  375
Cfau_S  356   D  V  H  Q  T  H  D  F  I  Q  V  A  L  D  A  E  Q  A  F  D  375
Cnau_S  356   D  V  N  Q  M  H  D  F  I  Q  V  A  L  D  A  D  Q  A  L  D  375
Pste_S  356   G  V  N  Q  T  H  D  F  I  Q  V  A  L  D  A  D  Q  A  L  D  375
Rma     357   D  V  N  Q  T  Y  D  F  I  Q  V  A  L  D  A  N  Q  A  L  D  376
Ifos_S  361   D  V  N  Q  T  H  D  F  I  Q  V  A  L  D  A  D  H  A  L  D  380
Apha_S  361   D  V  N  Q  T  H  D  F  I  Q  V  A  L  D  A  D  Q  A  L  D  380
Bsep_S  336   E  A  Q  Q  A  H  D  F  I  E  V  A  L  N  A  N  Q  A  L  D  355
```

```
Akaw_S 1129 GAATAT---CATGTCAGTGATATAGCGTTATTCTTGGATAGTAAAATCCAGCATATTTTA 1185
Clau_S 1129 GAATAT---CATGTCGGTGACATAGCGTTATTCTTGGATAATAAAATCCAGCATATTTTG 1185
Pkil_S 1129 GAACAT---TATGTCAGTGATATAGCGTTATTCTTGGATAATAAAATCCAGCATATTTTG 1185
Psoy_S 1129 GAACAT---TATGTCAGTGATATAGCGTTATTCTTGGATAATAAAATCCAGCATATTTTG 1185
Vok    1129 GAATAT---CATGTCAGTGATATAGCGTTATTCTTGGATAATAAAATCCAGCATATTTTG 1185
Cpac_S 1126 GAACAT---CAAGTCAGTGATATTGCACTATTTTTAGATAATAAAGTTCAGCATATTTTA 1182
Cfau_S 1126 GAACAT---CAAGTCAGTAATATTGCACTATTTTTAGATAATAAAGTCCAGCATATTTTA 1182
Cnau_S 1126 GAACAT---CAAGTCAGTGATATTGCACTATTTTTAGATACTAAAGTTCAGCATATTTTA 1182
Pste_S 1126 GAACAT---CAAGTCAGTGATATTGCACTATTTTTAGATAATAAAGTCCAGCATATTTTA 1182
Rma    1129 GAGCAT---CAAGTGGGTGATGTTGCACTGTTCTTGGATAATAAAGTTCAGCATATTTTG 1185
Ifos_S 1141 GAACAT---CAAGTGAGTGATGTTGCACTATTCTTGGACAACAAGGTTCAGCATATTTTG 1197
Apha_S 1141 GAGCAT---CAAGTCAGTGATATTGCACTATTCTTGGACAACAAAGTCCAGCATATTTTG 1197
Bsep_S 1066 AGTCAAATTGGCGTGAGCGATATTGCCCTATTTATGGATTATAAAGTGCAACATTTGCTG 1125
Akaw_S  377   E  Y  #  H  V  S  D  I  A  L  F  L  D  S  K  I  Q  H  I  L  395
Clau_S  377   E  Y  #  H  V  G  D  I  A  L  F  L  D  N  K  I  Q  H  I  L  395
Pkil_S  377   E  H  #  Y  V  S  D  I  A  L  F  L  D  N  K  I  Q  H  I  L  395
Psoy_S  377   E  H  #  Y  V  S  D  I  A  L  F  L  D  N  K  I  Q  H  I  L  395
Vok     377   E  Y  #  H  V  S  D  I  A  L  F  L  D  N  K  I  Q  H  I  L  395
Cpac_S  376   E  H  #  Q  V  S  D  I  A  L  F  L  D  N  K  V  Q  H  I  L  394
Cfau_S  376   E  H  #  Q  V  S  N  I  A  L  F  L  D  N  K  V  Q  H  I  L  394
Cnau_S  376   E  H  #  Q  V  S  D  I  A  L  F  L  D  T  K  V  Q  H  I  L  394
Pste_S  376   E  H  #  Q  V  S  D  I  A  L  F  L  D  N  K  V  Q  H  I  L  394
Rma     377   E  H  #  Q  V  G  D  V  A  L  F  L  D  N  K  V  Q  H  I  L  395
Ifos_S  381   E  H  #  Q  V  S  D  V  A  L  F  L  D  N  K  V  Q  H  I  L  399
Apha_S  381   E  H  #  Q  V  S  D  I  A  L  F  L  D  N  K  V  Q  H  I  L  399
Bsep_S  356   S  Q  I  G  V  S  D  I  A  L  F  M  D  Y  K  V  Q  H  L  L  375
```

```
Akaw_S 1186 ATTGATGAGTTTCAAGATACCTCAGTTATACAGTTTTCTTTATTAGAAAAGTTAATTGCC 1245
Clau_S 1186 ATTGATGAGTTTCAAGATATCTCAGTTATACAGTTTTCTTTATTAGAAAAATTAATTTCC 1245
Pkil_S 1186 ATTGATGAGTTTCAAGATACCTCAGTTATACAGTTTTCTTTATTAGAAAAATTAATTGCT 1245
Psoy_S 1186 ATTGATGAGTTTCAAGATACCTCAGTTATACAGTTTTCTTTATTAGAAAAATTAATTGCT 1245
Vok    1186 ATTGATGAGTTTCAAGATACCTCAGTTATACAGTTTTCTTTATTAGAAAAATTAATTGCT 1245
Cpac_S 1183 ATTGATGAGTTTCAAGATACGTCAACCACTCAGTTTGCTTTATTGGAAAAATTGCTTACT 1242
Cfau_S 1183 ATTGATGAGTTTCAAGATACGTCAGCCATCCAGTTTGCTTTATTGGAAAAATTGCTTGCT 1242
Cnau_S 1183 ATTGATGAGTTTCAAGATACGTCTGCCACGCAGTTTGCTTTATTGGAAAAATTGCTCGCT 1242
Pste_S 1183 ATTGATGAGTTTCAAGATACGTCTGCCACGCAGTTTGCCTTATTGGAAAAATTGCTTGCT 1242
Rma    1186 ATTGATGAGTTTCAAGACACCTCAGCTACTCAATTTGTTTTATTAGAAAAGTTGATTATT 1245
Ifos_S 1198 ATTGATGAGTTTCAAGACACCTCAGCCACTCAGTTTGTTTTATTAGAAAAGTTGATTGTT 1257
Apha_S 1198 ATTGATGAGTTTCAAGACACCTCAGCTACGCAGTTTTCTTTATTAGAAAAGTTGATTGTT 1257
Bsep_S 1126 ATTGACGAGTTTCAAGACACTTCGGCATCACAGTTTAATACGGTTGAAAAATTGATAGAA 1185
Akaw_S  396   I  D  E  F  Q  D  T  S  V  I  Q  F  S  L  L  E  K  L  I  A  415
Clau_S  396   I  D  E  F  Q  D  I  S  V  I  Q  F  S  L  L  E  K  L  I  S  415
Pkil_S  396   I  D  E  F  Q  D  T  S  V  I  Q  F  S  L  L  E  K  L  I  A  415
Psoy_S  396   I  D  E  F  Q  D  T  S  V  I  Q  F  S  L  L  E  K  L  I  A  415
Vok     396   I  D  E  F  Q  D  T  S  V  I  Q  F  S  L  L  E  K  L  I  A  415
Cpac_S  395   I  D  E  F  Q  D  T  S  T  T  Q  F  A  L  L  E  K  L  L  T  414
Cfau_S  395   I  D  E  F  Q  D  T  S  A  I  Q  F  A  L  L  E  K  L  L  A  414
Cnau_S  395   I  D  E  F  Q  D  T  S  A  T  Q  F  A  L  L  E  K  L  L  A  414
Pste_S  395   I  D  E  F  Q  D  T  S  A  T  Q  F  A  L  L  E  K  L  L  A  414
Rma     396   I  D  E  F  Q  D  T  S  A  T  Q  F  V  L  L  E  K  L  I  I  415
Ifos_S  400   I  D  E  F  Q  D  T  S  A  T  Q  F  V  L  L  E  K  L  I  V  419
Apha_S  400   I  D  E  F  Q  D  T  S  A  T  Q  F  S  L  L  E  K  L  I  V  419
Bsep_S  376   I  D  E  F  Q  D  T  S  A  S  Q  F  N  T  V  E  K  L  I  E  395
```

```
Akaw_S 1246 AACTGGCATTCAGGTGATGGAACAACACTATTCTTAGTAGGTGATCCTATGCAATCTATT 1305
Clau_S 1246 AACTGGTACTCAGGCGATGGAAAAACGCTATTTTTAGTAGGTGATCCTATGCAGTCTATT 1305
Pkil_S 1246 AATTGGCACTCAGGTGATGGGAAAACACTATTTTTAGTAGGTGATCCTATGCAGTCTATT 1305
Psoy_S 1246 AATTGGCACTCAGGTGATGGGAAAACACTATTTTTAGTAGGTGATCCTATGCAGTCTATT 1305
Vok    1246 AATTGGTACTCAGGTGATGGGAAAACACTATTTTTAGTAGGTGATCCTATGCAGTCTATT 1305
Cpac_S 1243 AGTTGGCAAATAGGTGACGGTAAAACACTGTTTTTAGTGGGTGATCCCATGCAATCTATT 1302
Cfau_S 1243 AGTTGGCAAATAGGTGATGGTAAAACACTGTTTTTAGTGGGTGATCCCATGCAATCTATT 1302
Cnau_S 1243 AGTTGGCAAATAGGTGATGGTAAAACACTATTTTTAGTGGGTGATCCTATGCAATCTATT 1302
Pste_S 1243 AGTTGGCAAATAGGTGATGGTAAAACACTGTTTTTAGTGGGTGATCCCATGCAATCTATT 1302
Rma    1246 AATTGGCAAGTAGGTGATGGTAAGACATTGTTTTTAGTGGGTGACCCCATGCAGTCTATT 1305
Ifos_S 1258 AATTGGCAAGTAGGTGATGGTAAAACGCTGTTTTTAGTGGGCGACCCCATGCAGTCTATT 1317
Apha_S 1258 AATTGGCAAGTAGGCGATGGTAAAACACTGTTTTTAGTGGGCGACCCCATGCAGTCTATT 1317
Bsep_S 1186 CAATGGCAAACAGATGATGAAAAAACGCTGTTCTTGGTGGGCGATCCAATGCAATCAATT 1245
Akaw_S  416   N  W  H  S  G  D  G  T  T  L  F  L  V  G  D  P  M  Q  S  I  435
Clau_S  416   N  W  Y  S  G  D  G  K  T  L  F  L  V  G  D  P  M  Q  S  I  435
Pkil_S  416   N  W  H  S  G  D  G  K  T  L  F  L  V  G  D  P  M  Q  S  I  435
Psoy_S  416   N  W  H  S  G  D  G  K  T  L  F  L  V  G  D  P  M  Q  S  I  435
Vok     416   N  W  Y  S  G  D  G  K  T  L  F  L  V  G  D  P  M  Q  S  I  435
Cpac_S  415   S  W  Q  I  G  D  G  K  T  L  F  L  V  G  D  P  M  Q  S  I  434
Cfau_S  415   S  W  Q  I  G  D  G  K  T  L  F  L  V  G  D  P  M  Q  S  I  434
Cnau_S  415   S  W  Q  I  G  D  G  K  T  L  F  L  V  G  D  P  M  Q  S  I  434
Pste_S  415   S  W  Q  I  G  D  G  K  T  L  F  L  V  G  D  P  M  Q  S  I  434
Rma     416   N  W  Q  V  G  D  G  K  T  L  F  L  V  G  D  P  M  Q  S  I  435
Ifos_S  420   N  W  Q  V  G  D  G  K  T  L  F  L  V  G  D  P  M  Q  S  I  439
Apha_S  420   N  W  Q  V  G  D  G  K  T  L  F  L  V  G  D  P  M  Q  S  I  439
Bsep_S  396   Q  W  Q  T  D  D  E  K  T  L  F  L  V  G  D  P  M  Q  S  I  415
```

```
Akaw_S 1306 TATTTATTTAGGCAATCTCAGGTAGGTTTATTTTTGCAGGTTAGAGCGCAAGGAATTGCT 1365
Clau_S 1306 TACTTATTTAGGCAATCTCAGGTAGGTTTATTTTTGCAGGTTAGAGCACGAGGAATTGCT 1365
Pkil_S 1306 TACTTATTTAGGCAATCTCAGGTAGGTTTATTTTTACAAGTTAGAACGCAAGGAATTGCT 1365
Psoy_S 1306 TACTTATTTAGGCAATCTCAGGTAGGTTTATTTTTACAAGTTAGAACGCAAGGAATTGCT 1365
Vok    1306 TACTTATTTAGGCAATCTCAGGTAGGTTTGTTTTTACAAGTTAGAACGCGAGGAATTGCT 1365
Cpac_S 1303 TACTTGTTTAGGCAATCTCAGGTGGGTTTGTTTTTGCAGGTTCGGGCACGAGGTATTGCT 1362
Cfau_S 1303 TACTTGTTTAGGCAATCTCAGGTGGGTTTGTTTTTGCAGGTACGGGCACGAGGCATTGCT 1362
Cnau_S 1303 TACTTGTTTAGGCAATCTCAGGTGGGTTTGTTTTTGCAAGTTCGGGCACGAGGCATTGCT 1362
Pste_S 1303 TACTTGTTTAGACAATCTCAGGTGGGTTTGTTTTTGCAGGTTCGGGCACGAGGCATTGCT 1362
Rma    1306 TATTTATTCAGGCAATCTCAAGTGGGCTTATTTTTGCAAGTTCGAGTACAAGGCATTGCT 1365
Ifos_S 1318 TACTTATTTAGGCAATCTCAGGTGGGCTTATTTTTACAGGTTCGGACGCAAGGCATTGCC 1377
Apha_S 1318 TACTTATTTCGGCAATCTCAGGTGGGCTTGTTTTTACAGGTTCGGACGCAAGGCATTGCC 1377
Bsep_S 1246 TACCGATTTAGAGAATCTCAGGTGGGGTTATTTTTGCAAGTGCGAGATAAGGGAATTGCG 1305
Akaw_S  436   Y  L  F  R  Q  S  Q  V  G  L  F  L  Q  V  R  A  Q  G  I  A  455
Clau_S  436   Y  L  F  R  Q  S  Q  V  G  L  F  L  Q  V  R  A  R  G  I  A  455
Pkil_S  436   Y  L  F  R  Q  S  Q  V  G  L  F  L  Q  V  R  T  Q  G  I  A  455
Psoy_S  436   Y  L  F  R  Q  S  Q  V  G  L  F  L  Q  V  R  T  Q  G  I  A  455
Vok     436   Y  L  F  R  Q  S  Q  V  G  L  F  L  Q  V  R  T  R  G  I  A  455
Cpac_S  435   Y  L  F  R  Q  S  Q  V  G  L  F  L  Q  V  R  A  R  G  I  A  454
Cfau_S  435   Y  L  F  R  Q  S  Q  V  G  L  F  L  Q  V  R  A  R  G  I  A  454
Cnau_S  435   Y  L  F  R  Q  S  Q  V  G  L  F  L  Q  V  R  A  R  G  I  A  454
Pste_S  435   Y  L  F  R  Q  S  Q  V  G  L  F  L  Q  V  R  A  R  G  I  A  454
Rma     436   Y  L  F  R  Q  S  Q  V  G  L  F  L  Q  V  R  V  Q  G  I  A  455
Ifos_S  440   Y  L  F  R  Q  S  Q  V  G  L  F  L  Q  V  R  T  Q  G  I  A  459
Apha_S  440   Y  L  F  R  Q  S  Q  V  G  L  F  L  Q  V  R  T  Q  G  I  A  459
Bsep_S  416   Y  R  F  R  E  S  Q  V  G  L  F  L  Q  V  R  D  K  G  I  A  435
```

```
Akaw_S 1366 AATATTAAGCCTAAATTCTTACAGCTTTGTACTAATTTTCGTTCCTCTCAATCTGTGGTT 1425
Clau_S 1366 AATATTAATCCTAAATTCTTACAACTTTATACTAATTTTCGTTCCTCTCAATCTGTTGTT 1425
Pkil_S 1366 AATATTAAGCCTGAATTCTTACAACTTCATACTAATTTTCGTTCTTCTCAATCTGTGGTT 1425
Psoy_S 1366 AATATTAAGCCTGAATTCTTACAACTTCATACTAATTTTCGTTCTTCTCAATCTGTGGTT 1425
Vok    1366 AATATTAAACCTGAATTCTTACAACTTCATACTAATTTTCGTTCCTCTCAATCTGTGGTT 1425
Cpac_S 1363 AATATTAAACCTGAATTTTTACAACTGAGTACCAATTTTCGCTCCTCTAAATCTGTGGTC 1422
Cfau_S 1363 AATATTAAACCTGAATTTTTACAACTGAGTACTAATTTTCGTTCCTCTAAATCTGTGGTT 1422
Cnau_S 1363 AATATTAAACCTGAATTTTTACAACTGAGTACTAATTTTCGCTCCTCTAAATCTGTGGTT 1422
Pste_S 1363 AATATTAAACCTGAATTTTTACAACTAAGTACCAATTTTCGCTCCTCTAAATCTGTGGTT 1422
Rma    1366 AATATTAAGCCTAAATTTTTACAACTAAGTACTAATTTTCGTTCCTCTGAATCTGTTGTT 1425
Ifos_S 1378 AGTATTAAGCCTGAATTTTTACAACTGAGTACTAATTTTCGCTCCTCTAGATCTGTGGTT 1437
Apha_S 1378 AATATTAAGCCTGAATTTTTACAACTGAGTACTAATTTTCGCTCCTCTAAATCTGTGGTT 1437
Bsep_S 1306 AACATTCGTCCAACTTCATTGGTATTGAGTACCAATTTTCGCTCTTCAAAAAGTATTGTT 1365
Akaw_S  456   N  I  K  P  K  F  L  Q  L  C  T  N  F  R  S  S  Q  S  V  V  475
Clau_S  456   N  I  N  P  K  F  L  Q  L  Y  T  N  F  R  S  S  Q  S  V  V  475
Pkil_S  456   N  I  K  P  E  F  L  Q  L  H  T  N  F  R  S  S  Q  S  V  V  475
Psoy_S  456   N  I  K  P  E  F  L  Q  L  H  T  N  F  R  S  S  Q  S  V  V  475
Vok     456   N  I  K  P  E  F  L  Q  L  H  T  N  F  R  S  S  Q  S  V  V  475
Cpac_S  455   N  I  K  P  E  F  L  Q  L  S  T  N  F  R  S  S  K  S  V  V  474
Cfau_S  455   N  I  K  P  E  F  L  Q  L  S  T  N  F  R  S  S  K  S  V  V  474
Cnau_S  455   N  I  K  P  E  F  L  Q  L  S  T  N  F  R  S  S  K  S  V  V  474
Pste_S  455   N  I  K  P  E  F  L  Q  L  S  T  N  F  R  S  S  K  S  V  V  474
Rma     456   N  I  K  P  K  F  L  Q  L  S  T  N  F  R  S  S  E  S  V  V  475
Ifos_S  460   S  I  K  P  E  F  L  Q  L  S  T  N  F  R  S  S  R  S  V  V  479
Apha_S  460   N  I  K  P  E  F  L  Q  L  S  T  N  F  R  S  S  K  S  V  V  479
Bsep_S  436   N  I  R  P  T  S  L  V  L  S  T  N  F  R  S  S  K  S  I  V  455
```

UvrD_C domain like region (464-826)

```
Akaw_S 1426 GAGGAAAATAATGAAATATTCTCAAAGATATTTCCTAATCAAGAAGAGGCTTATAAGGGC 1485
Clau_S 1426 GATGGAAATAATGAAATATTCTCAAAGATATTTCCTAATAAGGAAGAGGCTTATAAGGGT 1485
Pkil_S 1426 GAGGGAAATAATGAAATATTCTCAAAGATATTTCCTAATCAGGAAGAAGCTTATAAGGGC 1485
Psoy_S 1426 GAGGGAAATAATGAAATATTCTCAAAGATATTTCCTAATCAGGAAGAAGCTTATAAGGGC 1485
Vok    1426 GAGGGAAATAATGAAATATTCTCAAAGATATTTCCTAATCAGGAAGAAGCTTATAAGGGC 1485
Cpac_S 1423 GAGGGTAATAATAAAATATTTTCAAAAATATTTCCTCAACATGAAGATGCTTATAAAGGC 1482
Cfau_S 1423 GAGGGTAATAATAAAATATTTTCAAAGATATTTCCTCAACATGAAGATGCTTATAAAGGC 1482
Cnau_S 1423 GAGGGTAATAATAAAATATTTTCAAAGATATTTCCTCAACATGAAGATGCTTATAAAGGC 1482
Pste_S 1423 GAGGGTAATAATAAAATATTTTCAAAGATATTTCCTCAACATGAAGATGCTTATAAAGGC 1482
Rma    1426 GAAGGTAATAATAAAATATTTTCAAAGATATTTCCTCAGCAGGAAGATGCTTTTAAGGGC 1485
Ifos_S 1438 GAAGGTAATAATAAAATATTTTCAAAGGTATTTCCTCAGCAGGAAGATGCCTCTAAGGGT 1497
Apha_S 1438 GAAGGTAATAATAAAATATTTTCAAAGATATTTCCTCAGCAGGAAGATGCCTTTAAGGGT 1497
Bsep_S 1366 GAGGGCAATAATGGATTTTTCAGTAAGATTTTCCCTGAGTGTGACGATGTACATAAAGGT 1425
Akaw_S  476   E  E  N  N  E  I  F  S  K  I  F  P  N  Q  E  E  A  Y  K  G   495
Clau_S  476   D  G  N  N  E  I  F  S  K  I  F  P  N  K  E  E  A  Y  K  G   495
Pkil_S  476   E  G  N  N  E  I  F  S  K  I  F  P  N  Q  E  E  A  Y  K  G   495
Psoy_S  476   E  G  N  N  E  I  F  S  K  I  F  P  N  Q  E  E  A  Y  K  G   495
Vok     476   E  G  N  N  E  I  F  S  K  I  F  P  N  Q  E  E  A  Y  K  G   495
Cpac_S  475   E  G  N  N  K  I  F  S  K  I  F  P  Q  H  E  D  A  Y  K  G   494
Cfau_S  475   E  G  N  N  K  I  F  S  K  I  F  P  Q  H  E  D  A  Y  K  G   494
Cnau_S  475   E  G  N  N  K  I  F  S  K  I  F  P  Q  H  E  D  A  Y  K  G   494
Pste_S  475   E  G  N  N  K  I  F  S  K  I  F  P  Q  H  E  D  A  Y  K  G   494
Rma     476   E  G  N  N  K  I  F  S  K  I  F  P  Q  Q  E  D  A  F  K  G   495
Ifos_S  480   E  G  N  N  K  I  F  S  K  V  F  P  Q  Q  E  D  A  S  K  G   499
Apha_S  480   E  G  N  N  K  I  F  S  K  I  F  P  Q  Q  E  D  A  F  K  G   499
Bsep_S  456   E  G  N  N  G  F  F  S  K  I  F  P  E  C  D  D  V  H  K  G   475
```

UvrD_C domain like region (464-826)

```
Akaw_S 1486 GCAATTAAATATGAATATTCAAAGGCCCATTTTACTGCTGAAAGTAAAAGAGCTATTAAT 1545
Clau_S 1486 GCAATTAAGTATGAGTATTCAAAGGCCCATTTTACTACTGAAAGTAAAAAAGCTATTAAT 1545
Pkil_S 1486 GCAATTAAGTATGAGTATTCAAAGGCCTATTTTACTACTGAAAGCAAAAGAGCTATTAAT 1545
Psoy_S 1486 GCAATTAAGTATGAGTATTCAAAGGCCTATTTTACTACTGAAAGCAAAAGAGCTATTAAT 1545
Vok    1486 GCAATTAAGTATGAGTATTCAAAGGCCTATTTTACTGCTGAAAGCAAAAGAGCTATTAAT 1545
Cpac_S 1483 GCGATTAAGTATGAATATTCACAGGCTAATTCCGTTGATGAAGATGAAAACGCCATTAAC 1542
Cfau_S 1483 GCGATTAAGTATGAATATTCACAGGCTAATTCCGTTGATGAAGATGAAAATGCCATTAAC 1542
Cnau_S 1483 GCGATTAAGTATGAATATTCACAGGCTAATTCTGTTGATGAAGATGAAAATGCCATTAAC 1542
Pste_S 1483 GCGATTAAGTATGAATATTCACAGGCTAATTCCGTTGATGAAGATGAAAATGCCATTAAC 1542
Rma    1486 GCGATTAAGTATGAACATTCACAGGCTAATTTTGTTGATGAAAATGAAAACGCCATTAGA 1545
Ifos_S 1498 GCAATTAAGTATGAACATTCACAGACCAATTCTATTGACAAAGATGGCAATGCCATTAAC 1557
Apha_S 1498 GCAATTAAGTATGAACATTCACAGGCCAATTCTATTGATAAAGGTGAAAATGCCATTAAC 1557
Bsep_S 1426 GCAATTTCTTACTCACCATCGCTTGCAAATTCGGATACAAAGGATGACAATGCCATTGCA 1485
Akaw_S  496   A  I  K  Y  E  Y  S  K  A  H  F  T  A  E  S  K  R  A  I  N   515
Clau_S  496   A  I  K  Y  E  Y  S  K  A  H  F  T  T  E  S  K  K  A  I  N   515
Pkil_S  496   A  I  K  Y  E  Y  S  K  A  Y  F  T  T  E  S  K  R  A  I  N   515
Psoy_S  496   A  I  K  Y  E  Y  S  K  A  Y  F  T  T  E  S  K  R  A  I  N   515
Vok     496   A  I  K  Y  E  Y  S  K  A  Y  F  T  A  E  S  K  R  A  I  N   515
Cpac_S  495   A  I  K  Y  E  Y  S  Q  A  N  S  V  D  E  D  E  N  A  I  N   514
Cfau_S  495   A  I  K  Y  E  Y  S  Q  A  N  S  V  D  E  D  E  N  A  I  N   514
Cnau_S  495   A  I  K  Y  E  Y  S  Q  A  N  S  V  D  E  D  E  N  A  I  N   514
Pste_S  495   A  I  K  Y  E  Y  S  Q  A  N  S  V  D  E  D  E  N  A  I  N   514
Rma     496   A  I  K  Y  E  H  S  Q  A  N  F  V  D  E  N  E  N  A  I  R   515
Ifos_S  500   A  I  K  Y  E  H  S  Q  T  N  S  I  D  K  D  G  N  A  I  N   519
Apha_S  500   A  I  K  Y  E  H  S  Q  A  N  S  I  D  K  G  E  N  A  I  N   519
Bsep_S  476   A  I  S  Y  S  P  S  L  A  N  S  D  T  K  D  D  N  A  I  A   495
```

UvrD_C domain like region (464-826)

```
Akaw_S 1546 TTTTATCCATTTGCATATAAACGTTATGATTTTGAGGCACAACAAGTATTAAAAATAATT 1605
Clau_S 1546 TTTTATCCATTTGCATATAAACGTTATGATTTTGAAGCACGACAAGTATTAAAAATAATT 1605
Pkil_S 1546 TTTTATCCATTTGCATATAAACGTTATGATTGTGAAGCACAACAAGTATTAAAAATTATT 1605
Psoy_S 1546 TTTTATCCATTTGCATATAAACGTTATGATTGTGAAGCACAACAAGTATTAAAAATTATT 1605
Vok    1546 TTTTACCCATTTGCATATAAACGTTATGATTTTGAGGCACAACAAGTATTAAAAATTATT 1605
Cpac_S 1543 TTTTATCCATTTGCGCATAAACGTTATGATTTTGAGGCACAAAAAGTGCTTGAAATTATT 1602
Cfau_S 1543 TTTTATCCATTTGCGCATAAACGTTATGATTTTGAGGCACAAAAAGTGCTTGAAATTATT 1602
Cnau_S 1543 TTTTATTCATTTGCGTATAAACGTTATGATTTTGAGGCACAAAAAGTGCTTGAAATTATT 1602
Pste_S 1543 TTTTATCCATTTGCGCATAAACGTTATGATTTTGAGGCACAAAAAGTGCTTGAAATTATT 1602
Rma    1546 TTTTATCCATTTGCGTATAAACGCTATGATTTTGAGGCACAGAAAGTGCTTGAAATTATT 1605
Ifos_S 1558 TTTTATCCATTTGCGCACAAGCGTTATGATTTTGAGGCACAAAAAGTGCTTGAAATTATC 1617
Apha_S 1558 TTTTATCCATTTGCACACAAGTGTTATGATTTTGAGGCACAAAAAGTGCTTGAAATTATC 1617
Bsep_S 1486 TTCTATCCCTTTGCCCACGACCAATATCTATGTGAAGCAAAAACGATTGGCACGATTGTC 1545
Akaw_S  516   F  Y  P  F  A  Y  K  R  Y  D  F  E  A  Q  Q  V  L  K  I  I   535
Clau_S  516   F  Y  P  F  A  Y  K  R  Y  D  F  E  A  R  Q  V  L  K  I  I   535
Pkil_S  516   F  Y  P  F  A  Y  K  R  Y  D  C  E  A  Q  Q  V  L  K  I  I   535
Psoy_S  516   F  Y  P  F  A  Y  K  R  Y  D  C  E  A  Q  Q  V  L  K  I  I   535
Vok     516   F  Y  P  F  A  Y  K  R  Y  D  F  E  A  Q  Q  V  L  K  I  I   535
Cpac_S  515   F  Y  P  F  A  H  K  R  Y  D  F  E  A  Q  K  V  L  E  I  I   534
Cfau_S  515   F  Y  P  F  A  H  K  R  Y  D  F  E  A  Q  K  V  L  E  I  I   534
Cnau_S  515   F  Y  S  F  A  Y  K  R  Y  D  F  E  A  Q  K  V  L  E  I  I   534
Pste_S  515   F  Y  P  F  A  H  K  R  Y  D  F  E  A  Q  K  V  L  E  I  I   534
Rma     516   F  Y  P  F  A  Y  K  R  Y  D  F  E  A  Q  K  V  L  E  I  I   535
Ifos_S  520   F  Y  P  F  A  H  K  R  Y  D  F  E  A  Q  K  V  L  E  I  I   539
Apha_S  520   F  Y  P  F  A  H  K  C  Y  D  F  E  A  Q  K  V  L  E  I  I   539
Bsep_S  496   F  Y  P  F  A  H  D  Q  Y  L  C  E  A  K  T  I  G  T  I  V   515
```

UvrD_C domain like region (464-826)

```
Akaw_S 1606 ------------CAGAATAACCCAACAAAAGAGATTGCGATTTTAGTTAGAAATCGTTCA 1653
Clau_S 1606 ------------CAGAATAATCCAAGAAAAGAGATTGCTATTTTAGTTAGAAATCGTTCA 1653
Pkil_S 1606 ------------CAGAATAATCCAACAAAAGAGATTGCTATTTTAGTTAGAAATCGTTCA 1653
Psoy_S 1606 ------------CAGAATAATCCAACAAAAGAGATTGCTATTTTAGTTAGAAATCGTTCA 1653
Vok    1606 ------------CAGAACAATCCAACAAAAGAGATTGCCATTTTAGTTAGAAATCGTTCA 1653
Cpac_S 1603 ------------CAAAATAATCCAACAAGAGAGATTGTTATTTTGGTGAGAAACCGTTCG 1650
Cfau_S 1603 ------------CAAAATAATCCAACAAAAGAGATTGTTATTTTGGTGAGAAACCGTGCG 1650
Cnau_S 1603 ------------CAAAATAATCCAACAAAAGAGATTGTTATTTTGGTGAGAAACCGTTCG 1650
Pste_S 1603 ------------CAAAATAATCCAACAAAAGAGATTGTTATTTTGGTGAGAAACCGTTCG 1650
Rma    1606 ------------CAAAATAATCCAACAAAAGAGATCGTTATTTTGGTGAGAAGTCGTTCA 1653
Ifos_S 1618 ------------CAAAGCAACCCGGCAAAAGAGATTGCTATTTTGGTTAGAAACCGTTCA 1665
Apha_S 1618 ------------CAAAGCAACCCAACAAAAGAGATTGCTATTTTAGTTAGAAACCGTTCG 1665
Bsep_S 1546 GCCGCTAGTTTGGCGAAAAATACACAAAATACAATTGCGATTTTGGTGCGTGGGCGGGCA 1605
Akaw_S  536   #  #  #  #  Q  N  N  P  T  K  E  I  A  I  L  V  R  N  R  S   551
Clau_S  536   #  #  #  #  Q  N  N  P  R  K  E  I  A  I  L  V  R  N  R  S   551
Pkil_S  536   #  #  #  #  Q  N  N  P  T  K  E  I  A  I  L  V  R  N  R  S   551
Psoy_S  536   #  #  #  #  Q  N  N  P  T  K  E  I  A  I  L  V  R  N  R  S   551
Vok     536   #  #  #  #  Q  N  N  P  T  K  E  I  A  I  L  V  R  N  R  S   551
Cpac_S  535   #  #  #  #  Q  N  N  P  T  R  E  I  V  I  L  V  R  N  R  S   550
Cfau_S  535   #  #  #  #  Q  N  N  P  T  K  E  I  V  I  L  V  R  N  R  A   550
Cnau_S  535   #  #  #  #  Q  N  N  P  T  K  E  I  V  I  L  V  R  N  R  S   550
Pste_S  535   #  #  #  #  Q  N  N  P  T  K  E  I  V  I  L  V  R  N  R  S   550
Rma     536   #  #  #  #  Q  N  N  P  T  K  E  I  V  I  L  V  R  S  R  S   551
Ifos_S  540   #  #  #  #  Q  S  N  P  A  K  E  I  A  I  L  V  R  N  R  S   555
Apha_S  540   #  #  #  #  Q  S  N  P  T  K  E  I  A  I  L  V  R  N  R  S   555
Bsep_S  516   A  A  S  L  A  K  N  T  Q  N  T  I  A  I  L  V  R  G  R  A   535
```

UvrD_C domain like region (464-826)

```
Akaw_S 1654 CATTTAAATAATATTGTACCTGTTTTAAAGCAGGCTAATATTAAATTTGAAGCAGTGAAA 1713
Clau_S 1654 CATTTAAATAATATTGTACCTGTTTTAAATCAGGCTAATATTAAATTTGAGGCAGTAAAA 1713
Pkil_S 1654 CATTTAAATAATATTGTACCTGTTTTGAAGCAGGCTAATATTAAATTTGAGGCAGTGAAA 1713
Psoy_S 1654 CATTTAAATAATATTGTACCTGTTTTGAAGCAGGCTAATATTAAATTTGAGGCAGTGAAA 1713
Vok    1654 CATTTAAATAACATTGTACCTGTTTTAAAGCAGGCTAATATTAATTTGAGGCAGTGAAA 1713
Cpac_S 1651 CATTTAGGTGATATTGTGCCCGTTTTGAAGTGTGCTAATATTGAGTTTGAAGCATTAAAA 1710
Cfau_S 1651 CATTTAGGTGATATTGTGCCCGTTTTGAAGTGTGCTAATATTGAATTTGAAGCATTAAAA 1710
Cnau_S 1651 CATTTAGGTGATATTGTGCCCGTTCTGAAGTGTGCTAATATTGAATTTGAAGCATTAAAA 1710
Pste_S 1651 CATTTAGGTGATATTGTGTCCGTTTTGAAGTGTGCTAATATTGAATTTGAAGCATTAAAA 1710
Rma    1654 CATCTAGATGATATTGTATCCATTCTGAAGCGTGCTAATATTGAATTTGAGGCATTAAAA 1713
Ifos_S 1666 CACCTAGGTAATATTGTGCCCGTTTTGAAGTGTGCTAATATTCAATTTGAGGCATTAAAA 1725
Apha_S 1666 CACCTAGGTAATATTGTGCCCGTTTTGAAGCGTGCCAATATTCAATTTGAGGCATTAAGA 1725
Bsep_S 1606 CATTTGTTGCACATCGCACAACAGTTAATGGATGAGAAAATTGCATTTGAATCGGTAGAT 1665
Akaw_S  552   H  L  N  N  I  V  P  V  L  K  Q  A  N  I  K  F  E  A  V  K  571
Clau_S  552   H  L  N  N  I  V  P  V  L  N  Q  A  N  I  K  F  E  A  V  K  571
Pkil_S  552   H  L  N  N  I  V  P  V  L  K  Q  A  N  I  K  F  E  A  V  K  571
Psoy_S  552   H  L  N  N  I  V  P  V  L  K  Q  A  N  I  K  F  E  A  V  K  571
Vok     552   H  L  N  N  I  V  P  V  L  K  Q  A  N  I  N  F  E  A  V  K  571
Cpac_S  551   H  L  G  D  I  V  P  V  L  K  C  A  N  I  E  F  E  A  L  K  570
Cfau_S  551   H  L  G  D  I  V  P  V  L  K  C  A  N  I  E  F  E  A  L  K  570
Cnau_S  551   H  L  G  D  I  V  P  V  L  K  C  A  N  I  E  F  E  A  L  K  570
Pste_S  551   H  L  G  D  I  V  S  V  L  K  C  A  N  I  E  F  E  A  L  K  570
Rma     552   H  L  D  D  I  V  S  I  L  K  R  A  N  I  E  F  E  A  L  K  571
Ifos_S  556   H  L  G  N  I  V  P  V  L  K  C  A  N  I  Q  F  E  A  L  K  575
Apha_S  556   H  L  G  N  I  V  P  V  L  K  R  A  N  I  Q  F  E  A  L  R  575
Bsep_S  536   H  L  L  H  I  A  Q  Q  L  M  D  E  K  I  A  F  E  S  V  D  555
```

UvrD_C domain like region (464-826)

```
Akaw_S 1714 ATATTTCCATTAAAAGATGATTTATTTACCCGTGATTTGCTTAGCTTAACTCTGGCATTA 1773
Clau_S 1714 ATACTTCCATTAAAAGATGATTTATTTACCCGTGATTTGCTTAGCTTAACTCGGGCATTA 1773
Pkil_S 1714 ATACTTCCATTAAAAGATGATTTATTTACCCGTGATTTGCTTAGCTTAACTCGGGCATTA 1773
Psoy_S 1714 ATACTTCCATTAAAAGATGATTTATTTACCCGTGATTTGCTTAGCTTAACTCGGGCATTA 1773
Vok    1714 ATACTTCCATTAAAAGATGATTTATTTACCCGTGATTTGCTTAGCTTAACTCGGGCATTA 1773
Cpac_S 1711 ACGACCCCATTGAGGGAAGATTTATTTACTCGTGATTTGCTTAGTTTGACTCGAGCACTT 1770
Cfau_S 1711 ACAGCCTCATTGAGGGAAGATTTATTTACTCGTGATTTACTTAGTTTGACTCGAGCACTT 1770
Cnau_S 1711 ACAGCCCCATTGAGGGAAGATTTATTTACTCGTGATTTGCTTAGTTTGACTCGAGCACTT 1770
Pste_S 1711 ACAGCCCCATTGAGGGAAGATTTATTTACTCGTGATTTGCTTAGTTTGACTCGAGCACTT 1770
Rma    1714 ATACTTCCATTAAAGGAAGATTTATTTACTCGTGACTTGCTGAGTTTGACTCGAGCGCTC 1773
Ifos_S 1726 ACATCCCCATTAAAGCAAGATTTATTTACTCGTGATTTGCTTAGTCTGACTCGAGCACTT 1785
Apha_S 1726 ACATCCCCATTAAAGGAGGATTTATTTACCCGTGATTTGCTTAGTCTGACTCGAGCGCTT 1785
Bsep_S 1666 ATTACCGAATTAAAAGACCATTTATTGACGCGGACCTGCTCTCGCTAAGTAAGGCTTTA 1725
Akaw_S  572   I  F  P  L  K  D  D  L  F  T  R  D  L  L  S  L  T  L  A  L  591
Clau_S  572   I  L  P  L  K  D  D  L  F  T  R  D  L  L  S  L  T  R  A  L  591
Pkil_S  572   I  L  P  L  K  D  D  L  F  T  R  D  L  L  S  L  T  R  A  L  591
Psoy_S  572   I  L  P  L  K  D  D  L  F  T  R  D  L  L  S  L  T  R  A  L  591
Vok     572   I  L  P  L  K  D  D  L  F  T  R  D  L  L  S  L  T  R  A  L  591
Cpac_S  571   T  T  P  L  R  E  D  L  F  T  R  D  L  L  S  L  T  R  A  L  590
Cfau_S  571   T  A  S  L  R  E  D  L  F  T  R  D  L  L  S  L  T  R  A  L  590
Cnau_S  571   T  A  P  L  R  E  D  L  F  T  R  D  L  L  S  L  T  R  A  L  590
Pste_S  571   T  A  P  L  R  E  D  L  F  T  R  D  L  L  S  L  T  R  A  L  590
Rma     572   I  L  P  L  K  E  D  L  F  T  R  D  L  L  S  L  T  R  A  L  591
Ifos_S  576   T  S  P  L  K  Q  D  L  F  T  R  D  L  L  S  L  T  R  A  L  595
Apha_S  576   T  S  P  L  K  E  D  L  F  T  R  D  L  L  S  L  T  R  A  L  595
Bsep_S  556   I  T  E  L  K  D  H  L  L  T  R  D  L  L  S  L  S  K  A  L  575
```

UvrD_C domain like region (464-826)

```
Akaw_S 1774 AGATATTTAGGTGATAAACTTGCTTGGTTAGCTATTTTAAGAGCTCCTTGGTGTGGATTG 1833
Clau_S 1774 AGATATTTAGGTGATAAACTTGCTTGGTTATCTATTTTAAGAGCTCCTTGGTGCGGATTG 1833
Pkil_S 1774 ATATGTTTAGGTGATAAACTTGCTTGGTTAGCTATTTTAAGAGCTCCTTGGTGTGGATTA 1833
Psoy_S 1774 ATATGTTTAGGTGATAAACTTGCTTGGTTAGCTATTTTAAGAGCTCCTTGGTGTGGATTA 1833
Vok    1774 ATATATTTAGGTGATAAACTTGCTTGGTTAGCTATTTTAAGAGCTCCTTGGTGCGGGTTA 1833
Cpac_S 1771 AAACATTTAGGCGATAAACTTGCTTGGTTGGCCATCTTGAGAGCGCCTTGGTGTGGGTTA 1830
Cfau_S 1771 AAACATTTAGGTGATAAACTTGCTTGGTTGGCCATTTTAAGAGCGCCTTGGTGTGGCTTG 1830
Cnau_S 1771 AAACATTTAGGTGATAAACTTGCTTGGTTGGCCATTTTAAGAGCGCCTTGGTGTGGGTTA 1830
Pste_S 1771 AAACATTTAGGTGACAAACTTGCTTGGTTGGCCATTTTAAGAGCGCCTTGGTGTGGGTTA 1830
Rma    1774 AAACATTTGGGTGATAAACTTGCTTGGCTAGCTATCTTAAGAGCGCCATGGTGCGGGCTA 1833
Ifos_S 1786 AAACATCTGGGCGATAAACTTGCCTGGTTAGCTATTTTAAGAGCGCCTTGGTGTGGATTG 1845
Apha_S 1786 AAACATCTGGCTGATAAACTTGCCTGGTTAGCTATTTTAAGAGCGCCTTGGTGTGGATTG 1845
Bsep_S 1726 TTGCATTTGGGCGATAAACTGGCGTGGTTGAGTGTGTTGCGCGCCCCTTGGTGCGGCTTG 1785
Akaw_S  592   R  Y  L  G  D  K  L  A  W  L  A  I  L  R  A  P  W  C  G  L   611
Clau_S  592   R  Y  L  G  D  K  L  A  W  L  S  I  L  R  A  P  W  C  G  L   611
Pkil_S  592   I  C  L  G  D  K  L  A  W  L  A  I  L  R  A  P  W  C  G  L   611
Psoy_S  592   I  C  L  G  D  K  L  A  W  L  A  I  L  R  A  P  W  C  G  L   611
Vok     592   I  Y  L  G  D  K  L  A  W  L  A  I  L  R  A  P  W  C  G  L   611
Cpac_S  591   K  H  L  G  D  K  L  A  W  L  A  I  L  R  A  P  W  C  G  L   610
Cfau_S  591   K  H  L  G  D  K  L  A  W  L  A  I  L  R  A  P  W  C  G  L   610
Cnau_S  591   K  H  L  G  D  K  L  A  W  L  A  I  L  R  A  P  W  C  G  L   610
Pste_S  591   K  H  L  G  D  K  L  A  W  L  A  I  L  R  A  P  W  C  G  L   610
Rma     592   K  H  L  G  D  K  L  A  W  L  A  I  L  R  A  P  W  C  G  L   611
Ifos_S  596   K  H  L  G  D  K  L  A  W  L  A  I  L  R  A  P  W  C  G  L   615
Apha_S  596   K  H  L  A  D  K  L  A  W  L  A  I  L  R  A  P  W  C  G  L   615
Bsep_S  576   L  H  L  G  D  K  L  A  W  L  S  V  L  R  A  P  W  C  G  L   595
```

UvrD_C domain like region (464-826)

```
Akaw_S 1834 TTGTTAGAAGATTTACTTGTATTATCACAAAATAATGAACGTGTTATCTTTGATCTTATT 1893
Clau_S 1834 TTGTTAGAAGATTTACTTGTATTATCACAAAATAATAAACGTGTTATCTTTGATCTTATT 1893
Pkil_S 1834 TTGTTAGAAGATTTACTTGTATTATCACAAAATAATGAACGTGTTATCTTTGATCTTATT 1893
Psoy_S 1834 TTGTTAGAAGATTTACTTGTATTATCACAAAATAATGAACGTGTTATCTTTGATCTTATT 1893
Vok    1834 TTGTTAGAAGATTTACTTGTATTATCACAAAATAATGAACGTGTTATCTTTGATCTTATT 1893
Cpac_S 1831 TTATTAGAAGATTTACTTGTATTGTCACAACAAGACGGGCGTGTTATTTTTGATTTAATT 1890
Cfau_S 1831 TTGTTAGAAGATTTACTTGTATTGTCACAACAAGACGGGCGTGTTATTTTTGATTTAATT 1890
Cnau_S 1831 TTATTAGAAGATTTACTTGTATTGTCACAACAAGACGGGCGTGTTATTTTTGATTTAATT 1890
Pste_S 1831 TTATTAGAAGATTTACTTGTATTATCACAACAAGACGGGCGTGTTATTTTTGATTTAATT 1890
Rma    1834 TTATTAGAAGATTTACTTGTATTGTCACAACAGGATGAGCGTGTTATTTTTGATTTAATT 1893
Ifos_S 1846 TTGTTAGAAGACTTGCTTGTATTGTCACAACAGACTGAGTGTGTTATTTTTGATTTAATT 1905
Apha_S 1846 TTGTTAGAAGATCTGCTTGTATTGTCACAACAGGATGGGTGTGTTATTTTTGATTTAATT 1905
Bsep_S 1786 ATTTTGGATGATTTGTTGGTGCTATCTGAGGATGATAATCAGATAATTTATAGGCAACTA 1845
Akaw_S  612   L  L  E  D  L  L  V  L  S  Q  N  N  E  R  V  I  F  D  L  I   631
Clau_S  612   L  L  E  D  L  L  V  L  S  Q  N  N  K  R  V  I  F  D  L  I   631
Pkil_S  612   L  L  E  D  L  L  V  L  S  Q  N  N  E  R  V  I  F  D  L  I   631
Psoy_S  612   L  L  E  D  L  L  V  L  S  Q  N  N  E  R  V  I  F  D  L  I   631
Vok     612   L  L  E  D  L  L  V  L  S  Q  N  N  E  R  V  I  F  D  L  I   631
Cpac_S  611   L  L  E  D  L  L  V  L  S  Q  Q  D  G  R  V  I  F  D  L  I   630
Cfau_S  611   L  L  E  D  L  L  V  L  S  Q  Q  D  G  R  V  I  F  D  L  I   630
Cnau_S  611   L  L  E  D  L  L  V  L  S  Q  Q  D  G  R  V  I  F  D  L  I   630
Pste_S  611   L  L  E  D  L  L  V  L  S  Q  Q  D  G  R  V  I  F  D  L  I   630
Rma     612   L  L  E  D  L  L  V  L  S  Q  Q  D  E  R  V  I  F  D  L  I   631
Ifos_S  616   L  L  E  D  L  L  V  L  S  Q  Q  T  E  C  V  I  F  D  L  I   635
Apha_S  616   L  L  E  D  L  L  V  L  S  Q  Q  D  G  C  V  I  F  D  L  I   635
Bsep_S  596   I  L  D  D  L  L  V  L  S  E  D  D  N  Q  I  I  Y  R  Q  L   615
```

UvrD_C domain like region (464-826)

```
Akaw_S 1894 CGGGATGATCAGACATTAAAAGATCTGAGTTTAGATGGACGTATTCGGGTGGATAATTTT 1953
Clau_S 1894 CAGGATGATAAGATATTACAAGATCTAAGTTCAGATGGACGTATTCGTGTGGATAATTTT 1953
Pkil_S 1894 CGGGATGATCAGACATTACAAGGTCTGAGTTTAGATGGACGTATTCGTGTGGATAATTTT 1953
Psoy_S 1894 CGGGATGATCAGACATTACAAGGTCTGAGTTTAGATGGACGTATTCGTGTGGATAATTTT 1953
Vok    1894 CGAGATGATCAGACATTACAAGGTTTGAGTTCAGATGGACGTATTCGTGTGGATAATTTT 1953
Cpac_S 1891 CAAGATAAGCAAACTTTACAAAATTTAAGCAAAGATGGACAATCGCGCATGCGAAATTTT 1950
Cfau_S 1891 CAGGATAAGCAAACTTTACAAAATTTAAGCAAAGATGGACAATCACGCATGCGAAATTTT 1950
Cnau_S 1891 CAGGATAAGAAAATTTTACAAAATTTAAGCAAAGATGGACAATCACGTATGCGAAATTTT 1950
Pste_S 1891 CAGGATAAAAAAACTTTACAAAATTTAAGTAAAGATGGACAATCGCGCATGCGAAATTTT 1950
Rma    1894 CAAGATGAGCAAGTTTTACAAGGTTTGAGCGAAAATGGACAATTACGTATACGAAATTTT 1953
Ifos_S 1906 CAAGATGAACAAGTTTTACAAGGTTTGAGCGAGAACGGACAATTGTGCGTGCGAAATTTT 1965
Apha_S 1906 CAAGATGAGCAAGTTTTACAAGATTTGAGCGAGAACGGACAATTGCGTGTGCGAAATTTT 1965
Bsep_S 1846 AATGATGAGGCGATATTGGTAAAAATGAGTGCAGATGGGCGTGAGCGTGCACAGCATCTA 1905
Akaw_S  632   R  D  D  Q  T  L  K  D  L  S  L  D  G  R  I  R  V  D  N  F  651
Clau_S  632   Q  D  D  K  I  L  Q  D  L  S  S  D  G  R  I  R  V  D  N  F  651
Pkil_S  632   R  D  D  Q  T  L  Q  G  L  S  L  D  G  R  I  R  V  D  N  F  651
Psoy_S  632   R  D  D  Q  T  L  Q  G  L  S  L  D  G  R  I  R  V  D  N  F  651
Vok     632   R  D  D  Q  T  L  Q  G  L  S  S  D  G  R  I  R  V  D  N  F  651
Cpac_S  631   Q  D  K  Q  T  L  Q  N  L  S  K  D  G  Q  S  R  M  R  N  F  650
Cfau_S  631   Q  D  K  Q  T  L  Q  N  L  S  K  D  G  Q  S  R  M  R  N  F  650
Cnau_S  631   Q  D  K  K  I  L  Q  N  L  S  K  D  G  Q  S  R  M  R  N  F  650
Pste_S  631   Q  D  K  K  T  L  Q  N  L  S  K  D  G  Q  S  R  M  R  N  F  650
Rma     632   Q  D  E  Q  V  L  Q  G  L  S  E  N  G  Q  L  R  I  R  N  F  651
Ifos_S  636   Q  D  E  Q  V  L  Q  G  L  S  E  N  G  Q  L  C  V  R  N  F  655
Apha_S  636   Q  D  E  Q  V  L  Q  D  L  S  E  N  G  Q  L  R  V  R  N  F  655
Bsep_S  616   N  D  E  A  I  L  V  K  M  S  A  D  G  R  E  R  A  Q  H  L  635
```

UvrD_C domain like region (464-826)

```
Akaw_S 1954 GCCCATATTTTAGGTGATGTTGTTAATCAGCAATCTAGATTTAGTTTTACTCAGGTACTT 2013
Clau_S 1954 GCTTATGTTTTAAGTGATATTGTTAATCAACAATCTAGATTTAGTTTTACTCAGCTACTT 2013
Pkil_S 1954 GCCTATGTTTTAGGTAATATTGTTAATCAACAATCTAGATTTAGTTTTACTCAAGTACTT 2013
Psoy_S 1954 GCCTATGTTTTAGGTAATATTGTTAATCAACAATCTAGATTTAGTTTTACTCAAGTACTT 2013
Vok    1954 GCCTATGTTTTAGGTAATATTGTTAATCAACAATCTAGATTTAGTTTTACTCAAGTACTT 2013
Cpac_S 1951 GTCCATGCTGTACGTGATATTATCAATCAGAAATCTAGGTTTAGCTTCATGAGGGTGCTT 2010
Cfau_S 1951 GTCCATACTGTACGTGATATTATCAATCAGAAATCTAGGTTTAGTTTCACGAGGGTGCTT 2010
Cnau_S 1951 GTCCATGCTGTACGTGATATTATTAATCAGAAATCTAGGTTTAGTTTCACGAAGGCGCTT 2010
Pste_S 1951 GTCCGTGCTGTACGTGATATTATCAGTCAAAAATCTAGATTTAGTTTCACGAGGGTGCTT 2010
Rma    1954 GTCCATGTTTTTTGTGATATTGTTAATCAACAATCTAGATTTAGTTTTACGAAGGTGCTT 2013
Ifos_S 1966 GCCCATGTTTTACGTGATATTGTTAATCAACAATCTAGATTTAGTTTCACAAAGGTGCTT 2025
Apha_S 1966 GCTCATGTTTTACGTGATATTGTTAATCAACAATCTAGATTTAGTTTCACAAAGGTGCTT 2025
Bsep_S 1906 TATCAGTGTTTGCAAGAGGTTATTAGCAATCAAGGGCGATTTAATTTTGTGGAATTATTG 1965
Akaw_S  652   A  H  I  L  G  D  V  V  N  Q  Q  S  R  F  S  F  T  Q  V  L  671
Clau_S  652   A  Y  V  L  S  D  I  V  N  Q  Q  S  R  F  S  F  T  Q  L  L  671
Pkil_S  652   A  Y  V  L  G  N  I  V  N  Q  Q  S  R  F  S  F  T  Q  V  L  671
Psoy_S  652   A  Y  V  L  G  N  I  V  N  Q  Q  S  R  F  S  F  T  Q  V  L  671
Vok     652   A  Y  V  L  G  N  I  V  N  Q  Q  S  R  F  S  F  T  Q  V  L  671
Cpac_S  651   V  H  A  V  R  D  I  I  N  Q  K  S  R  F  S  F  M  R  V  L  670
Cfau_S  651   V  H  T  V  R  D  I  I  N  Q  K  S  R  F  S  F  T  R  V  L  670
Cnau_S  651   V  H  A  V  R  D  I  I  N  Q  K  S  R  F  S  F  T  K  A  L  670
Pste_S  651   V  R  A  V  R  D  I  I  S  Q  K  S  R  F  S  F  T  R  V  L  670
Rma     652   V  H  V  F  C  D  I  V  N  Q  Q  S  R  F  S  F  T  K  V  L  671
Ifos_S  656   A  H  V  L  R  D  I  V  N  Q  Q  S  R  F  S  F  T  K  V  L  675
Apha_S  656   A  H  V  L  R  D  I  V  N  Q  Q  S  R  F  S  F  T  K  V  L  675
Bsep_S  636   Y  Q  C  L  Q  E  V  I  S  N  Q  G  R  F  N  F  V  E  L  L  655
```

UvrD_C domain like region (464-826)

```
Akaw_S 2014 GAATTTGCGATTAATCAATTAGTACCTCAAAAT---TCCTTATCTGTTAAACAGTCTATG 2070
Clau_S 2014 GAATTTGCGATTAATCAATTAGTACCTCCAAAT---TCTTTATCTGTTAAACAGTCTATG 2070
Pkil_S 2014 GAATTTGCGATTAATCAATTAGTACCTCTAAAT---TCTTTATCTGTTAAACAGTCTATG 2070
Psoy_S 2014 GAATTTGCGATTAATCAATTAGTACCTCTAAAT---TCTTTATCTGTTAAACAGTCTATG 2070
Vok    2014 GAATTTGCGATTAATCAATTAGTACCTCCAAAT---TCTTTATCTGTTAAACAGTCTATG 2070
Cpac_S 2011 GAATTTGCCATTAATCAGTTAGCACCTCAGAAT---TCCTTATCCGTTAAGCAGTCTATG 2067
Cfau_S 2011 GAATTTGCCATTAATCAGTTAGCACCTCAGAGT---TCCTTATCCGTTAAGCAGTCTATG 2067
Cnau_S 2011 GAATTTTCCATTAATCAGTTAGCACCTCAGAGT---TCCTTATCCGTTAAGCAGTTTATG 2067
Pste_S 2011 GAATTTGCCATTAATCAGTTAGCACCTCAGAGT---TCCTTATCCGTTAAGCAATCTATG 2067
Rma    2014 GCATTTGCGATTAATCAGTTAGCACCTCAGAGT---TCTTTATCCGTTAAGCGATCTATG 2070
Ifos_S 2026 GAATTTGCGATTAACCAGCTAGCACCGCAGAAT---TCCTTATCTGTTAAGCAGTCTATG 2082
Apha_S 2026 GAATTTGCGATTAACCAGTTAGCACCGCAGGAT---TCCTTATCCGTTAAACAATCTATG 2082
Bsep_S 1966 ACCCATACAATGAATCAATTGGGGTTAAAAAATGAGACTTTAGCAAAAACGGAATTGGCA 2025
Akaw_S  672   E  F  A  I  N  Q  L  V  P  Q  N  #  S  L  S  V  K  Q  S  M  690
Clau_S  672   E  F  A  I  N  Q  L  V  P  P  N  #  S  L  S  V  K  Q  S  M  690
Pkil_S  672   E  F  A  I  N  Q  L  V  P  L  N  #  S  L  S  V  K  Q  S  M  690
Psoy_S  672   E  F  A  I  N  Q  L  V  P  L  N  #  S  L  S  V  K  Q  S  M  690
Vok     672   E  F  A  I  N  Q  L  V  P  P  N  #  S  L  S  V  K  Q  S  M  690
Cpac_S  671   E  F  A  I  N  Q  L  A  P  Q  N  #  S  L  S  V  K  Q  S  M  689
Cfau_S  671   E  F  A  I  N  Q  L  A  P  Q  S  #  S  L  S  V  K  Q  S  M  689
Cnau_S  671   E  F  S  I  N  Q  L  A  P  Q  S  #  S  L  S  V  K  Q  F  M  689
Pste_S  671   E  F  A  I  N  Q  L  A  P  Q  S  #  S  L  S  V  K  Q  S  M  689
Rma     672   A  F  A  I  N  Q  L  A  P  Q  S  #  S  L  S  V  K  R  S  M  690
Ifos_S  676   E  F  A  I  N  Q  L  A  P  Q  N  #  S  L  S  V  K  Q  S  M  694
Apha_S  676   E  F  A  I  N  Q  L  A  P  Q  D  #  S  L  S  V  K  Q  S  M  694
Bsep_S  656   T  H  T  M  N  Q  L  G  L  K  N  E  T  L  A  K  T  E  L  A  675
```

UvrD_C domain like region (464-826)

```
Akaw_S 2071 ATTAAAACTCAGTTCTTGCAAATTATTTATAATTGTGAGTTTGAGCAACAGTTGGATATT 2130
Clau_S 2071 ATTAAAACTCAGTTCTTGCAAATTATTTATGACTGTGAGTTTGAGCAACAGTTGGATGTT 2130
Pkil_S 2071 ATTAAAACTCAGTTTTTGCAAATTATTTATGACTGTGAGTTTGAGCAACAGTTGGATATT 2130
Psoy_S 2071 ATTAAAACTCAGTTTTTGCAAATTATTTATGACTGTGAGTTTGAGCAACAGTTGGATATT 2130
Vok    2071 ATTAAAACTCAGTTCTTGCAAATTATTTATGACTGTGAGTTTGAGCAACAGTTGGATATT 2130
Cpac_S 2068 ATTAAAACCCAATTCTTGCAAATTATTCATGATTGTGAGTCTGCGCAACAGCTTGACATT 2127
Cfau_S 2068 ATTAAAACCCAATTCTTGCAAATTATTTATGATTGTGAGTCTGCACAGCAGCTTGATATT 2127
Cnau_S 2068 ATTAAAACCCAATTCTTGAAAATTATTTATGATTGTGAGTCTGCGCAACAGCTTGATATT 2127
Pste_S 2068 ATTAAAACCCAATTCTTGCAAATTATTCATGATTGTGAGTTTGCGCAACAGCTTGATATT 2127
Rma    2071 ATTAAAATCCAATTCTTGCAAATTATTCATGATTGTGAGTCTGTGCAACAACTTGATATT 2130
Ifos_S 2083 ATTAAAGCTCAGTTTTTGCAAATTATTCATGATTGTGAGTCTGCGCAACAACTTGATATT 2142
Apha_S 2083 ATTAAAGCTCAGTTTTTGCAAATTATTCATGATTGTGAGTCTGCGCAACAACTTGATATT 2142
Bsep_S 2026 ATTAAAGACAAATTTTTGCAGATTATTTATGAATGCGAACAGCAACAATTACTGAGCGCG 2085
Akaw_S  691   I  K  T  Q  F  L  Q  I  I  Y  N  C  E  F  E  Q  Q  L  D  I  710
Clau_S  691   I  K  T  Q  F  L  Q  I  I  Y  D  C  E  F  E  Q  Q  L  D  V  710
Pkil_S  691   I  K  T  Q  F  L  Q  I  I  Y  D  C  E  F  E  Q  Q  L  D  I  710
Psoy_S  691   I  K  T  Q  F  L  Q  I  I  Y  D  C  E  F  E  Q  Q  L  D  I  710
Vok     691   I  K  T  Q  F  L  Q  I  I  Y  D  C  E  F  E  Q  Q  L  D  I  710
Cpac_S  690   I  K  T  Q  F  L  Q  I  I  H  D  C  E  S  A  Q  Q  L  D  I  709
Cfau_S  690   I  K  T  Q  F  L  Q  I  I  Y  D  C  E  S  A  Q  Q  L  D  I  709
Cnau_S  690   I  K  T  Q  F  L  K  I  I  Y  D  C  E  S  A  Q  Q  L  D  I  709
Pste_S  690   I  K  T  Q  F  L  Q  I  I  H  D  C  E  F  A  Q  Q  L  D  I  709
Rma     691   I  K  I  Q  F  L  Q  I  I  H  D  C  E  S  V  Q  Q  L  D  I  710
Ifos_S  695   I  K  A  Q  F  L  Q  I  I  H  D  C  E  S  A  Q  Q  L  D  I  714
Apha_S  695   I  K  A  Q  F  L  Q  I  I  H  D  C  E  S  A  Q  Q  L  D  I  714
Bsep_S  676   I  K  D  K  F  L  Q  I  I  Y  E  C  E  Q  Q  Q  L  L  S  A  695
```

UvrD_C domain like region (464-826)

```
Akaw_S 2131 GAAACTATTAATCAAATGTTAGATGAATTATATACACCTAGTGTGGATGTATTGAATGCA 2190
Clau_S 2131 GAAACTATTAATCAAATGTTAGATGAATTGTATACACCTAGTGTGAATGTATTGAATGCA 2190
Pkil_S 2131 GAAACTATTAATAAAATGTTAGATGAATTGTATACACCTAGTGTGAATGTATTGAATGCA 2190
Psoy_S 2131 GAAACTATTAATAAAATGTTAGATGAATTGTATACACCTAGTGTGAATGTATTGAATGCA 2190
Vok    2131 GAAACTATTAATAAAATGTTAGATGAATTGTATACACCTAGTGTGAATGTATTGAATGCA 2190
Cpac_S 2128 GGAACCATTAATCAAATGTTAGATGAGTTGTATGCACCCAGTGTAGAC---------GCA 2178
Cfau_S 2128 GGAATCATTAATCAAATGTTAGATGAGTTGTATACACCCAGTGTAAAT---------TCA 2178
Cnau_S 2128 GAAACCATTAATCAAATGTTAGATGAGTTGTATGCACCCAGTGTAGAT---------GCA 2178
Pste_S 2128 GAAACCATTAATCAAATGTTAGATGAGTTGTATGCACCCAGTGTAAAT---------GCA 2178
Rma    2131 GAAACCATTAATCAAATGTTAGATGGGTTGTATGCACCTAGTGTAAAT---------GCA 2181
Ifos_S 2143 GAGACCATTAATCAAATGTTAGATGAGTTGTATGCACCTAGTGTAAAT---------GCA 2193
Apha_S 2143 GAAACCATTAATCAAATGTTAGGTGAGTTGTATGCGCCCAGTGTAAAT---------GCA 2193
Bsep_S 2086 GATACCATTGAACAGATGATAGAAAAGTTATATGCACCGAGTGAAAAG---------GCG 2136
Akaw_S  711  E  T  I  N  Q  M  L  D  E  L  Y  T  P  S  V  D  V  L  N  A  730
Clau_S  711  E  T  I  N  Q  M  L  D  E  L  Y  T  P  S  V  N  V  L  N  A  730
Pkil_S  711  E  T  I  N  K  M  L  D  E  L  Y  T  P  S  V  N  V  L  N  A  730
Psoy_S  711  E  T  I  N  K  M  L  D  E  L  Y  T  P  S  V  N  V  L  N  A  730
Vok     711  E  T  I  N  K  M  L  D  E  L  Y  T  P  S  V  N  V  L  N  A  730
Cpac_S  710  G  T  I  N  Q  M  L  D  E  L  Y  A  P  S  V  D  #  #  #  A  726
Cfau_S  710  G  I  I  N  Q  M  L  D  E  L  Y  T  P  S  V  N  #  #  #  S  726
Cnau_S  710  E  T  I  N  Q  M  L  D  E  L  Y  A  P  S  V  D  #  #  #  A  726
Pste_S  710  E  T  I  N  Q  M  L  D  E  L  Y  A  P  S  V  N  #  #  #  A  726
Rma     711  E  T  I  N  Q  M  L  D  G  L  Y  A  P  S  V  N  #  #  #  A  727
Ifos_S  715  E  T  I  N  Q  M  L  D  E  L  Y  A  P  S  V  N  #  #  #  A  731
Apha_S  715  E  T  I  N  Q  M  L  G  E  L  Y  A  P  S  V  N  #  #  #  A  731
Bsep_S  696  D  T  I  E  Q  M  I  E  K  L  Y  A  P  S  E  K  #  #  #  A  712
```

UvrD_C domain like region (464-826)

```
Akaw_S 2191 CGAATTAAGTTAATGACTATTCATGAAGCGAAAGGTTTGGAATTTGAGTTGGTTATTATT 2250
Clau_S 2191 CGAATTAAGTTAATGACTATTCATGAAGCGAAAGGCTTGGAGTTTGAGTTGGTTATTATT 2250
Pkil_S 2191 CGAATTAAGTTAATGACTATTCATGAAGCGAAAGGTTTGGAATTTGAGTTGGTTATTATT 2250
Psoy_S 2191 CGAATTAAGTTAATGACTATTCATGAAGCGAAAGGTTTGGAATTTGAGTTGGTTATTATT 2250
Vok    2191 CGAATTAAGTTAATGACTATTCATGAAGCAAAAGGTTTGGAATTTGAGTTGGTTATTATT 2250
Cpac_S 2179 CGAATTAAGCTAATGACCATCCATGAAGCTAAAGGTTTGGAGTTTGAGTTGGTTGTTATT 2238
Cfau_S 2179 CGAATTAAGCTAATGACCATCCATGAAGCTAAAGGTTTGGAGTTTGAGTTGGTTGTTATT 2238
Cnau_S 2179 CGAATTAAGTTAATGACTATCCATGAAGCTAAAGGTTTGGAGTTTGAGTTGGTTGTTATT 2238
Pste_S 2179 CGAATTAAGCTAATGACCATCCATGAAGCTAAAGGCTTGGAGTTTGAGTTGGTTGTTATT 2238
Rma    2182 CGAATTAAGCTAATGACCATTCATGAGGCTAAAGGCTTGGAATTTGAGTTGGTTATTATT 2241
Ifos_S 2194 CGAATTAAGCTAATGACCATCCATGAAGCTAAAGGTTTGGAGTTTGAATTGGTTATTATT 2253
Apha_S 2194 CGAATTAAGCTAATGACCATCCATGAAGCTAAAGGTTTGGAGTTTGAATTGGTTATTATT 2253
Bsep_S 2137 CAGGTGAAGTTAATGACAGTGCATGCATCAAAGGGCCTGGAGTTTGACACGGTGATTATT 2196
Akaw_S  731  R  I  K  L  M  T  I  H  E  A  K  G  L  E  F  E  L  V  I  I  750
Clau_S  731  R  I  K  L  M  T  I  H  E  A  K  G  L  E  F  E  L  V  I  I  750
Pkil_S  731  R  I  K  L  M  T  I  H  E  A  K  G  L  E  F  E  L  V  I  I  750
Psoy_S  731  R  I  K  L  M  T  I  H  E  A  K  G  L  E  F  E  L  V  I  I  750
Vok     731  R  I  K  L  M  T  I  H  E  A  K  G  L  E  F  E  L  V  I  I  750
Cpac_S  727  R  I  K  L  M  T  I  H  E  A  K  G  L  E  F  E  L  V  V  I  746
Cfau_S  727  R  I  K  L  M  T  I  H  E  A  K  G  L  E  F  E  L  V  V  I  746
Cnau_S  727  R  I  K  L  M  T  I  H  E  A  K  G  L  E  F  E  L  V  V  I  746
Pste_S  727  R  I  K  L  M  T  I  H  E  A  K  G  L  E  F  E  L  V  V  I  746
Rma     728  R  I  K  L  M  T  I  H  E  A  K  G  L  E  F  E  L  V  I  I  747
Ifos_S  732  R  I  K  L  M  T  I  H  E  A  K  G  L  E  F  E  L  V  I  I  751
Apha_S  732  R  I  K  L  M  T  I  H  E  A  K  G  L  E  F  E  L  V  I  I  751
Bsep_S  713  Q  V  K  L  M  T  V  H  A  S  K  G  L  E  F  D  T  V  I  I  732
```

UvrD_C domain like region (464-826)

```
Akaw_S 2251 CCTGGATTGGGAAGAGCGTCACAAAATAATAAACCACCTATCATTCACTTACAAGAATTT 2310
Clau_S 2251 CCTGGGTTGGGAAGAGCGTCACAAAATAATAAATCACCTATCATTCACTTACAAGAGTTT 2310
Pkil_S 2251 CCTGGGTTGGGAAGAGCATCACAAAATAATAAATCACCTATCATTCATTTACAAGAATTT 2310
Psoy_S 2251 CCTGGGTTGGGAAGAGCATCACAAAATAATAAATCACCTATCATTCATTTACAAGAATTT 2310
Vok    2251 CCTGGGTTGGGAAGAGTGTCACAAAATAATAAATCACCTATCATTCATTTACAAGAATTT 2310
Cpac_S 2239 CTTGGGTTGGGCAGAGTGCCACAAAATAATAAACTGCCCATCATTCACTTGCAAGAGTTT 2298
Cfau_S 2239 CTTGGGTTGGGTAGAGCGCCACAAAATAATAAACCGTCCATCATTCACTTGCAAGAGTTT 2298
Cnau_S 2239 CTTGGGTTGGGTAGGACGCCACAAAATAATAAACCGTCCATCATTCATTTGCAAGAGTTT 2298
Pste_S 2239 CTTGGGTTGGGTAGAGCGCCACAAAATAATAAACCGTCCATAATTCACTTGCAAGAGTTT 2298
Rma    2242 CCTGGGTTGGGTAGAATGCCACAAAATAACATACCATCCATCATTCACTTGCAAGAATTT 2301
Ifos_S 2254 CCTGGGCTGGGTAGAGCGCCACAAAATAACAAACCACCCATCATTCACCTGCAAGAATTT 2313
Apha_S 2254 CCTGGGCTGGGTAAAGCGCCACAAAATAACAAACCACCCATCATTCACTTGCAAGAATTT 2313
Bsep_S 2197 CCGGGCTTGGGTCGGAGTTCTGGGAGGGATGATTCGCTGATTATTCGATTGCGTGAATTT 2256
Akaw_S  751   P  G  L  G  R  A  S  Q  N  N  K  P  P  I  I  H  L  Q  E  F  770
Clau_S  751   P  G  L  G  R  A  S  Q  N  N  K  S  P  I  I  H  L  Q  E  F  770
Pkil_S  751   P  G  L  G  R  A  S  Q  N  N  K  S  P  I  I  H  L  Q  E  F  770
Psoy_S  751   P  G  L  G  R  A  S  Q  N  N  K  S  P  I  I  H  L  Q  E  F  770
Vok     751   P  G  L  G  R  V  S  Q  N  N  K  S  P  I  I  H  L  Q  E  F  770
Cpac_S  747   L  G  L  G  R  V  P  Q  N  N  K  L  P  I  I  H  L  Q  E  F  766
Cfau_S  747   L  G  L  G  R  A  P  Q  N  N  K  P  S  I  I  H  L  Q  E  F  766
Cnau_S  747   L  G  L  G  R  T  P  Q  N  N  K  P  S  I  I  H  L  Q  E  F  766
Pste_S  747   L  G  L  G  R  A  P  Q  N  N  K  P  S  I  I  H  L  Q  E  F  766
Rma     748   P  G  L  G  R  M  P  Q  N  N  I  P  S  I  I  H  L  Q  E  F  767
Ifos_S  752   P  G  L  G  R  A  P  Q  N  N  K  P  P  I  I  H  L  Q  E  F  771
Apha_S  752   P  G  L  G  K  A  P  Q  N  N  K  P  P  I  I  H  L  Q  E  F  771
Bsep_S  733   P  G  L  G  R  S  S  G  R  D  D  S  L  I  I  R  L  R  E  F  752
```

UvrD_C domain like region (464-826)

```
Akaw_S 2311 AGTAATCAGTCTTTATTATTAGCACCGATAAGGTCTTATACACAATTACATGATAGTTGT 2370
Clau_S 2311 AGTAATCAGTCTTTATTATTAGCGCCGATAAGGTCTTATACACAATTAAATGATAGTTGT 2370
Pkil_S 2311 AGTAATCAGTCTTTATTATTAGCACCGATAAGGTCTTATATACAATTAAATGATAGTCGT 2370
Psoy_S 2311 AGTAATCAGTCTTTATTATTAGCACCGATAAGGTCTTATATACAATTAAATGATAGTCGT 2370
Vok    2311 AGTAATCAGTCTTTATTATTAGCGCCGATAAGGTCTTATACACAATTAAATGATAGTTGT 2370
Cpac_S 2299 AGCAATCGGTCGTTATTATTAGCCCCGATAAGACTTTATACGCAACTAGATGACAGCCGT 2358
Cfau_S 2299 AGCAATCGGTCGTTATTATTAGCCCCGATAAGGCCTTATACGCAACTAGATGACAGCCGT 2358
Cnau_S 2299 AGCAATCGGTCGTTATTATTAGCCCCGATAAGGGCTTATACGCAACTAGATGACAGCCGT 2358
Pste_S 2299 AGCAATCGGTCGTTATTATTAGCCCCGATAAGGGCTTATACGAAACTAGATGACAGCCGT 2358
Rma    2302 AGCAATCAATCATTATTATTAGCGCCGATAAGATCTTATACGCAATTAGATGACAGTCAT 2361
Ifos_S 2314 AGCAATCAGTCGTTATTATTAGCACCAATAAGGGCTTATACGCAATTAGATGACAGCCGT 2373
Apha_S 2314 AGCAATCAGTCGTTATTATTGGCACCGATAAGGTCTTATACACAACTAGATGACAGCCAT 2373
Bsep_S 2257 TCGAATAAGGATTTACTATTAGCACCAATGAAGTCGGCATCTGCCACACAAGAAAGTGGG 2316
Akaw_S  771   S  N  Q  S  L  L  L  A  P  I  R  S  Y  T  Q  L  H  D  S  C  790
Clau_S  771   S  N  Q  S  L  L  L  A  P  I  R  S  Y  T  Q  L  N  D  S  C  790
Pkil_S  771   S  N  Q  S  L  L  L  A  P  I  R  S  Y  I  Q  L  N  D  S  R  790
Psoy_S  771   S  N  Q  S  L  L  L  A  P  I  R  S  Y  I  Q  L  N  D  S  R  790
Vok     771   S  N  Q  S  L  L  L  A  P  I  R  S  Y  T  Q  L  N  D  S  C  790
Cpac_S  767   S  N  R  S  L  L  L  A  P  I  R  L  Y  T  Q  L  D  D  S  R  786
Cfau_S  767   S  N  R  S  L  L  L  A  P  I  R  P  Y  T  Q  L  D  D  S  R  786
Cnau_S  767   S  N  R  S  L  L  L  A  P  I  R  A  Y  T  Q  L  D  D  S  R  786
Pste_S  767   S  N  R  S  L  L  L  A  P  I  R  A  Y  T  K  L  D  D  S  R  786
Rma     768   S  N  Q  S  L  L  L  A  P  I  R  S  Y  T  Q  L  D  D  S  H  787
Ifos_S  772   S  N  Q  S  L  L  L  A  P  I  R  A  Y  T  Q  L  D  D  S  R  791
Apha_S  772   S  N  Q  S  L  L  L  A  P  I  R  S  Y  T  Q  L  D  D  S  H  791
Bsep_S  753   S  N  K  D  L  L  L  A  P  M  K  S  A  S  A  T  Q  E  S  G  772
```

UvrD_C domain like region (464-826)

```
Akaw_S 2371 ACTTATGTTTATTTAAAACATATTAAATCCCAACAAGATAGGTTTGAAGCTATGCGTTTA 2430
Clau_S 2371 ACTTATGTTTATTTAAAGCATATTAAATCACAACAAGACAGGTTTGAAACCATTCGTTTA 2430
Pkil_S 2371 ACTTATGTTTATTTGAAGCATATTAAATCACAACAAGATAGGTTTGAAACCATGCGTTTA 2430
Psoy_S 2371 ACTTATGTTTATTTGAAGCATATTAAATCACAACAAGATAGGTTTGAAACCATGCGTTTA 2430
Vok    2371 ACTTATGTTTATTTAAAGCATATTAAATCACAACAAGATAGGTTTGAAACCATGCGTTTA 2430
Cpac_S 2359 ACTTATACCTATTTAAAGCATGTTGAATCACAGCAAAATAAGTTTGAAACGATGCGTTTA 2418
Cfau_S 2359 ACTTATACCTATTTAAAGCATATTGAATCACAGCAAAATAAGTTTGAAACGATGCGTTTA 2418
Cnau_S 2359 ACTTATACCTATTTAAAGCATGTTGAATCACAGAAAAATAAGTTTGAAACGATGCGTTTA 2418
Pste_S 2359 ACTTATACCTATTTAAAGCATGTTGAATCACAGCAAAATAAGTTTGAAACAATGCGTTTA 2418
Rma    2362 ACTTATACCTACTTGAAGCATATTAAATCACAACAAAATAAGTTTGAAACGATGCGTTTA 2421
Ifos_S 2374 ACTTATACCTACTTAAGGCATATTGAATCACAACAAAATAAGTTTGAAACCATGCGTTTA 2433
Apha_S 2374 ACTTACACCTATTTAAAGCATATTGCATTACAACAAAATAAGTTTGAAACGATGCGTTTA 2433
Bsep_S 2317 GTATATCGGTATTTGAAATCTATTGAAACAGAGCAAAACTATTATGAAAGCATGCGCTTA 2376
Akaw_S  791   T  Y  V  Y  L  K  H  I  K  S  Q  Q  D  R  F  E  A  M  R  L  810
Clau_S  791   T  Y  V  Y  L  K  H  I  K  S  Q  Q  D  R  F  E  T  I  R  L  810
Pkil_S  791   T  Y  V  Y  L  K  H  I  K  S  Q  Q  D  R  F  E  T  M  R  L  810
Psoy_S  791   T  Y  V  Y  L  K  H  I  K  S  Q  Q  D  R  F  E  T  M  R  L  810
Vok     791   T  Y  V  Y  L  K  H  I  K  S  Q  Q  D  R  F  E  T  M  R  L  810
Cpac_S  787   T  Y  T  Y  L  K  H  V  E  S  Q  Q  N  K  F  E  T  M  R  L  806
Cfau_S  787   T  Y  T  Y  L  K  H  I  E  S  Q  Q  N  K  F  E  T  M  R  L  806
Cnau_S  787   T  Y  T  Y  L  K  H  V  E  S  Q  K  N  K  F  E  T  M  R  L  806
Pste_S  787   T  Y  T  Y  L  K  H  V  E  S  Q  Q  N  K  F  E  T  M  R  L  806
Rma     788   T  Y  T  Y  L  K  H  I  K  S  Q  Q  N  K  F  E  T  M  R  L  807
Ifos_S  792   T  Y  T  Y  L  R  H  I  E  S  Q  Q  N  K  F  E  T  M  R  L  811
Apha_S  792   T  Y  T  Y  L  K  H  I  A  L  Q  Q  N  K  F  E  T  M  R  L  811
Bsep_S  773   V  Y  R  Y  L  K  S  I  E  T  E  Q  N  Y  Y  E  S  M  R  L  792
```

UvrD_C domain like region (464-826)

```
Akaw_S 2431 TTATATGTAGCTATGACGCGTGCAAAATATAAAATTCATTTATTGGCAACATTAAGTCAA 2490
Clau_S 2431 TTATATGTAGCTATGACACGTGCAAAATATAAAATTCATTTATTAGCAACATTAAGTCAA 2490
Pkil_S 2431 TTATATGTAGCTATGACACGTGCAAAATATAAAATTCATTTATTAGCAACATTAAGTCAA 2490
Psoy_S 2431 TTATATGTAGCTATGACACGTGCAAAATATAAAATTCATTTATTAGCAACATTAAGTCAA 2490
Vok    2431 TTATATGTAGCTATGACACGTGCAAAATATAAAATTTATTTATTAGCAACATTAAGTCAA 2490
Cpac_S 2419 TTGTACGTGGCAATGACACGTGCTAAGTATGAAATCCATTTATTAGGAACGCTAAACCAA 2478
Cfau_S 2419 TTGTACGTGGCAATGACACGTGCTAAGCATGAAATCCATCTATTAGGAACGTTAAACCAG 2478
Cnau_S 2419 TTGTACGTGGCAATGACACGTGCTAAGCATGAAATCCATTTATTAGGAACGCTAAATCAA 2478
Pste_S 2419 TTGTACGTGGCAATGACACGTGCTAAGCATGAAATCCATTTATTAGGAACGTTAAACCAA 2478
Rma    2422 TTGTACGTAGCAATGACACGTGCTAAGCTTGAAATTCATTTATTAGGAACGCTAAATCAA 2481
Ifos_S 2434 TTGTACGTGGCAATGACACGTGCTAAGTTTGAAATTCATTTATTAGGAACGCTAAATCAA 2493
Apha_S 2434 TTGTACGTGGCAATGACACGTGCTAAGTTTGAAATTCATTTATTAGGAACGCTAAATAAA 2493
Bsep_S 2377 TTGTATGTGGCAATGACGCGAGCAAAAAGCCATTTACACCTATTGGGGGCAGTAAATAAG 2436
Akaw_S  811   L  Y  V  A  M  T  R  A  K  Y  K  I  H  L  L  A  T  L  S  Q  830
Clau_S  811   L  Y  V  A  M  T  R  A  K  Y  K  I  H  L  L  A  T  L  S  Q  830
Pkil_S  811   L  Y  V  A  M  T  R  A  K  Y  K  I  H  L  L  A  T  L  S  Q  830
Psoy_S  811   L  Y  V  A  M  T  R  A  K  Y  K  I  H  L  L  A  T  L  S  Q  830
Vok     811   L  Y  V  A  M  T  R  A  K  Y  K  I  Y  L  L  A  T  L  S  Q  830
Cpac_S  807   L  Y  V  A  M  T  R  A  K  Y  E  I  H  L  L  G  T  L  N  Q  826
Cfau_S  807   L  Y  V  A  M  T  R  A  K  H  E  I  H  L  L  G  T  L  N  Q  826
Cnau_S  807   L  Y  V  A  M  T  R  A  K  H  E  I  H  L  L  G  T  L  N  Q  826
Pste_S  807   L  Y  V  A  M  T  R  A  K  H  E  I  H  L  L  G  T  L  N  Q  826
Rma     808   L  Y  V  A  M  T  R  A  K  L  E  I  H  L  L  G  T  L  N  Q  827
Ifos_S  812   L  Y  V  A  M  T  R  A  K  F  E  I  H  L  L  G  T  L  N  Q  831
Apha_S  812   L  Y  V  A  M  T  R  A  K  F  E  I  H  L  L  G  T  L  N  K  831
Bsep_S  793   L  Y  V  A  M  T  R  A  K  S  H  L  H  L  L  G  A  V  N  K  812
```

UvrD_C domain like region (464-826)

```
Akaw_S 2491 AATAATCAAGCCATTAGAAATACTTTTTTAAAATTATTAGAACCTATATTTCAGAACCAG 2550
Clau_S 2491 AGTAATCAAGCTATTAGAAATACTTTTTTAAAATTATTAGAACCTATATTTCAAAACCAG 2550
Pkil_S 2491 AGCAATCAAGCCATTAGAAATACTTTTTTAAAATTATTAGAACCTATATTTCAGAACCAG 2550
Psoy_S 2491 AGCAATCAAGCCATTAGAAATACTTTTTTAAAATTATTAGAACCTATATTTCAGAACCAG 2550
Vok    2491 AGTAATCAAGCCATTAGAAACACTTTTTTAAAATTATTAGAACCTATATTTCAGAACCGG 2550
Cpac_S 2479 AGCAATCAAGCTAGTAGCAATACTTTTTTAAAACTATTAGCACCTATATTTCAGCATCAA 2538
Cfau_S 2479 AGCAATCAAGCTAGTAGCAATACTTTTTTAAAACTATTAGCACCTATATTTCAGCATCAA 2538
Cnau_S 2479 AGCAATCAGGCCAGTAGCAATACTTTTTTAAAACTATTAGCACCTATATTTCAGCATCAA 2538
Pste_S 2479 AGCAATCAAGCCAGTAGCAATACTTTTTTAAAACTATTAGCACCTATATTTCAGCATCAA 2538
Rma    2482 AGCAATCAAGCTAGTAGTAATACTTTTTTAAAACTATTGGCGCCCATATTTCAGCATCAA 2541
Ifos_S 2494 AGCAATCAAGCCAGTAGCAATACTTTTTTAAAACTATTGGCACCTATATTTCAGCATCAA 2553
Apha_S 2494 AGCAATCAAGCCAGTAGCAATACTTTTTTAAAACTATTGGCACCTATATTTCAGCATCAA 2553
Bsep_S 2437 TCAGGGAATATCGGTAAAAATACGCTGTTGGAATTATTGGGGCAGTTTTTACACATCGG 2496
Akaw_S  831   N  N  Q  A  I  R  N  T  F  L  K  L  L  E  P  I  F  Q  N  Q  850
Clau_S  831   S  N  Q  A  I  R  N  T  F  L  K  L  L  E  P  I  F  Q  N  Q  850
Pkil_S  831   S  N  Q  A  I  R  N  T  F  L  K  L  L  E  P  I  F  Q  N  Q  850
Psoy_S  831   S  N  Q  A  I  R  N  T  F  L  K  L  L  E  P  I  F  Q  N  Q  850
Vok     831   S  N  Q  A  I  R  N  T  F  L  K  L  L  E  P  I  F  Q  N  R  850
Cpac_S  827   S  N  Q  A  S  S  N  T  F  L  K  L  L  A  P  I  F  Q  H  Q  846
Cfau_S  827   S  N  Q  A  S  S  N  T  F  L  K  L  L  A  P  I  F  Q  H  Q  846
Cnau_S  827   S  N  Q  A  S  S  N  T  F  L  K  L  L  A  P  I  F  Q  H  Q  846
Pste_S  827   S  N  Q  A  S  S  N  T  F  L  K  L  L  A  P  I  F  Q  H  Q  846
Rma     828   S  N  Q  A  S  S  N  T  F  L  K  L  L  A  P  I  F  Q  H  Q  847
Ifos_S  832   S  N  Q  A  S  S  N  T  F  L  K  L  L  A  P  I  F  Q  H  Q  851
Apha_S  832   S  N  Q  A  S  S  N  T  F  L  K  L  L  A  P  I  F  Q  H  Q  851
Bsep_S  813   S  G  N  I  G  K  N  T  L  L  E  L  L  G  Q  F  F  T  H  R  832
```

```
Akaw_S 2551 TTTGACCAACTT------AAACCATTAGTTATTGAGGATAATAAAGAACCCATCCTAGCG 2604
Clau_S 2551 TTTGACCAGCTC------AAGTCTTCAGTTATTGAGGATAATAAACAACCCATTCGAGCG 2604
Pkil_S 2551 TTTGACCAGCTT------AAGTCGTCAGTTATTGAGGATAATAAACAACCCATACAAGCG 2604
Psoy_S 2551 TTTGACCAGCTT------AAGTCGTCAGTTATTGAGGATAATAAACAACCCATACAAGCG 2604
Vok    2551 TTTGACCAGCTT------AAGTCGTCAGTCATTGAGGATAATAAACAACCCATACGAGCG 2604
Cpac_S 2539 TTTGACAAACTT------ACACTATCAACCATTGAGGATAATCAGCTACCCATTCGAGCA 2592
Cfau_S 2539 TTTGACAAGCTT------ACACTATCAACTATTGAGGATAATCAGCCACCCATTCGAGCA 2592
Cnau_S 2539 TTTGACAAGTTT------ACACTATCAACCATTGAGAATAATCAGCCACCCATTCGAGCA 2592
Pste_S 2539 TTTGACAAGCTT------ACACTATCAACCATTGAGAATAATCAGCCACCCATTCGAGCA 2592
Rma    2542 TTTGACAAGCTT------AAACTATCAACCACTGAGGACAATCAACCACTCGTTCAAGCG 2595
Ifos_S 2554 TTTGACAAGCTC------AAACTATCAACCACTGAGGATAATCAA---TCCGTTCAAGCA 2604
Apha_S 2554 TTTGACAAGCTC------AAACTATCAACCACTGAGGATAATCAACAACCCGTTCAAGCG 2607
Bsep_S 2497 TTTGATGATATTGACAAGACGCCTGATACAGTGGAATCAGCG------------GAAATT 2544
Akaw_S  851   F  D  Q  L  #  #  K  P  L  V  I  E  D  N  K  E  P  I  L  A  868
Clau_S  851   F  D  Q  L  #  #  K  S  S  V  I  E  D  N  K  Q  P  I  R  A  868
Pkil_S  851   F  D  Q  L  #  #  K  S  S  V  I  E  D  N  K  Q  P  I  Q  A  868
Psoy_S  851   F  D  Q  L  #  #  K  S  S  V  I  E  D  N  K  Q  P  I  Q  A  868
Vok     851   F  D  Q  L  #  #  K  S  S  V  I  E  D  N  K  Q  P  I  R  A  868
Cpac_S  847   F  D  K  L  #  #  T  L  S  T  I  E  D  N  Q  L  P  I  R  A  864
Cfau_S  847   F  D  K  L  #  #  T  L  S  T  I  E  D  N  Q  P  P  I  R  A  864
Cnau_S  847   F  D  K  F  #  #  T  L  S  T  I  E  N  N  Q  P  P  I  R  A  864
Pste_S  847   F  D  K  L  #  #  T  L  S  T  I  E  N  N  Q  P  P  I  R  A  864
Rma     848   F  D  K  L  #  #  K  L  S  T  T  E  D  N  Q  P  L  V  Q  A  865
Ifos_S  852   F  D  K  L  #  #  K  L  S  T  T  E  D  N  Q  #  S  V  Q  A  868
Apha_S  852   F  D  K  L  #  #  K  L  S  T  T  E  D  N  Q  Q  P  V  Q  A  869
Bsep_S  833   F  D  D  I  D  K  T  P  D  T  V  E  S  A  #  #  #  #  E  I  848
```

```
Akaw_S 2605 CCAGAGTTAGTACGTTATATTAAGCCTAGAGAATATAATAATTTGCTTGATAGAAATAAG 2664
Clau_S 2605 CCAGAGTTAGTACGTTATATTAAGCCTATAGAATATAATAATTTGCCTGATAGAAGTAAG 2664
Pkil_S 2605 CCAGAGTTAGTACGTTATATTAAGCCTATAGAATATAATAATTTACCCGATAGAAGTAAG 2664
Psoy_S 2605 CCAGAGTTAGTACGTTATATTAAGCCTATAGAATATAATAATTTACCCGATAGAAGTAAG 2664
Vok    2605 CCAGAGTTAGTACGTTATATTAAGCCTATAGAATATAATAATTTGCTCGATAGAAGTAAG 2664
Cpac_S 2593 CCAGAGTTGGTGCGTTATGTTAAGCCTTTAGAATATGGTAATTTGCTTGATGAAAGTATG 2652
Cfau_S 2593 CCAGAGTTGGTTCGTTATGTTAAGCCTTTAGAATATGATAATTTTCTTGATGAGAGTATG 2652
Cnau_S 2593 CCAGAGTTGGTGCGTTATGTTAAGCCTTTAGAGTATGATAATTTGCTTGATGAAAGTATG 2652
Pste_S 2593 CCAGAGTTGGTGCGTTATGTTAAGCCTTTAGAATATGATAATTTGCTTGATGAAAGTATG 2652
Rma    2596 CCAGAGTTTGTGCGTTATATTAAGCCCTTAGAATATGATAATTTGCCTGATGAAAGTAAG 2655
Ifos_S 2605 CCAGAGTTGGTGCGTTATATTGAGCCTTTAGAATATGATAATTTGCCTGATGAAAGTAAG 2664
Apha_S 2608 CCAGAGTTGGTACGTTATATTAAGCCCTTAGAATATGATAATTTGCCTGATGAAAGTAAG 2667
Bsep_S 2545 TTGCAGTTAGCGCGTGTTTCCGAGTTAAAAACGCCAGTTAATCGAATGCAAGAAAAAGGC 2604
Akaw_S  869  P  E  L  V  R  Y  I  K  P  R  E  Y  N  N  L  L  D  R  N  K   888
Clau_S  869  P  E  L  V  R  Y  I  K  P  I  E  Y  N  N  L  P  D  R  S  K   888
Pkil_S  869  P  E  L  V  R  Y  I  K  P  I  E  Y  N  N  L  P  D  R  S  K   888
Psoy_S  869  P  E  L  V  R  Y  I  K  P  I  E  Y  N  N  L  P  D  R  S  K   888
Vok     869  P  E  L  V  R  Y  I  K  P  I  E  Y  N  N  L  L  D  R  S  K   888
Cpac_S  865  P  E  L  V  R  Y  V  K  P  L  E  Y  G  N  L  L  D  E  S  M   884
Cfau_S  865  P  E  L  V  R  Y  V  K  P  L  E  Y  D  N  F  L  D  E  S  M   884
Cnau_S  865  P  E  L  V  R  Y  V  K  P  L  E  Y  D  N  L  L  D  E  S  M   884
Pste_S  865  P  E  L  V  R  Y  V  K  P  L  E  Y  D  N  L  L  D  E  S  M   884
Rma     866  P  E  F  V  R  Y  I  K  P  L  E  Y  D  N  L  P  D  E  S  K   885
Ifos_S  869  P  E  L  V  R  Y  I  E  P  L  E  Y  D  N  L  P  D  E  S  K   888
Apha_S  870  P  E  L  V  R  Y  I  K  P  L  E  Y  D  N  L  P  D  E  S  K   889
Bsep_S  849  L  Q  L  A  R  V  S  E  L  K  T  P  V  N  R  M  Q  E  K  G   868
```

```
Akaw_S 2665 AAAAAAATGAACTTTCAGTTTAATGTTGATTTGCAATATAAAAGCTTACTTGGTACTTTA 2724
Clau_S 2665 AAAAAAATGAACTTTCAGCTTAATGTTGATTTGCAATATAAAAGCTTACTTGGTACTTTA 2724
Pkil_S 2665 AAAAAAATGAACTTTCAGCTTAATGTTGATTTGCAATATAAAAGTTTACTTGGTATTTTA 2724
Psoy_S 2665 AAAAAAATGAATTTTCAGCTTAATGTTGATTTGCAATATAAAAGTTTACTTGGTATTTTA 2724
Vok    2665 AAAAAAATGAACTTTCAGCTTAATGTTGATTTGCAATATAAAAGCTTACTTGGTATTTTA 2724
Cpac_S 2653 GAGGAAATGGATTTTCAGTTGGGTATTGATTTACAATACAAAAGCCTGCTTGACACTTTG 2712
Cfau_S 2653 GAGGAAATGGATTTTCAGTTGAGTGTTGATTTACAATATAAAAGCCTGCTTGGCACTCTG 2712
Cnau_S 2653 GAGGAAATGGATTTTCAGTTGAGTGTTGATTCACAATACAAAAGCCTACTTGGCACTTTG 2712
Pste_S 2653 GAGGAAATGGATTTTCAGTTGAGTGTTGATTTACAATACAAAAGCCTGCTTGGCACTTTG 2712
Rma    2656 AAAAAAATGGATTTTCAACTTAGTGTTGATTTGCAATACAAAAGCTTACTTGGCACTTTA 2715
Ifos_S 2665 GAAAAAATGGACTTTCAGCTTAGTGTTGATTTGCAATACAAAAGCTTGCTTGGTACTTTG 2724
Apha_S 2668 GAAAAAATGGACTTTCAGCTTAGTGTTGATTTGCAATACAAAAGCTTGCTTGGCACTTTG 2727
Bsep_S 2605 GAAATGGTAGAATATCAGCAGAATTTTGAACGCTTATTTAAGCGTGCATTAGGCACTTTG 2664
Akaw_S  889  K  K  M  N  F  Q  F  N  V  D  L  Q  Y  K  S  L  L  G  T  L   908
Clau_S  889  K  K  M  N  F  Q  L  N  V  D  L  Q  Y  K  S  L  L  G  T  L   908
Pkil_S  889  K  K  M  N  F  Q  L  N  V  D  L  Q  Y  K  S  L  L  G  I  L   908
Psoy_S  889  K  K  M  N  F  Q  L  N  V  D  L  Q  Y  K  S  L  L  G  I  L   908
Vok     889  K  K  M  N  F  Q  L  N  V  D  L  Q  Y  K  S  L  L  G  I  L   908
Cpac_S  885  E  E  M  D  F  Q  L  G  I  D  L  Q  Y  K  S  L  L  D  T  L   904
Cfau_S  885  E  E  M  D  F  Q  L  S  V  D  L  Q  Y  K  S  L  L  G  T  L   904
Cnau_S  885  E  E  M  D  F  Q  L  S  V  D  S  Q  Y  K  S  L  L  G  T  L   904
Pste_S  885  E  E  M  D  F  Q  L  S  V  D  L  Q  Y  K  S  L  L  G  T  L   904
Rma     886  K  K  M  D  F  Q  L  S  V  D  L  Q  Y  K  S  L  L  G  T  L   905
Ifos_S  889  E  K  M  D  F  Q  L  S  V  D  L  Q  Y  K  S  L  L  G  T  L   908
Apha_S  890  E  K  M  D  F  Q  L  S  V  D  L  Q  Y  K  S  L  L  G  T  L   909
Bsep_S  869  E  M  V  E  Y  Q  Q  N  F  E  R  L  F  K  R  A  L  G  T  L   888
```

```
Akaw_S 2725 TTACATCAATATTATGCAGATGAATTGTTCGCTCCAGATAGGCAAAATATAAGAGTAAGA 2784
Clau_S 2725 TTACATCAATATTATGCAGATGAATTGTTCGCGCCAGATAGGCAAAATATAAGAGTAAGA 2784
Pkil_S 2725 TTACATCAATATTATGCAGATGAATTGTTCTCGCCAGATAGGCAAAATATAAGAGTAAGA 2784
Psoy_S 2725 TTACATCAATATTATGCAGATGAATTGTTCTCGCCAGATAGGCAAAATATAAGAGTAAGA 2784
Vok    2725 TTACATCAATATTATGCAAATGAATTGTTCTCGCCAGATAGGCAAAATATAAGAGTAAGA 2784
Cpac_S 2713 TTGTATCAATATTATGAAAACGGATTGTTCTCGCCAGACAAACAAAGTATAAGAGTAAGA 2772
Cfau_S 2713 TTGCATCAATATTATGAAGACGGATTGTTCTCGCCAGACAGACAAAGTATAAGTGTAAGA 2772
Cnau_S 2713 TTGCATCAATATTATGAAGACGGATTGTTCTCGCCAGACAGACAAAGTATAAGCGTAAGA 2772
Pste_S 2713 TTGCATCAATATTATGAAGACGGATTATTCTTGCCAGACAGACAAAGTATAAGCGTAAGA 2772
Rma    2716 TTGCATCAATATTATGAAGACAGTTTGTTCTCACCAGATAAACAAAGTATAAGAGCAAGA 2775
Ifos_S 2725 TTGCATCAATATTATGAAGACGGGTTGTTTTCACCAGATAAACAAAGTATAAGAGTAAGG 2784
Apha_S 2728 TTGCATCAATATTATGAAGACGGGTTGTTTTCACCAGAAAAACAAAGTATAAGAGCAAGA 2787
Bsep_S 2665 GTGCATCAATATTATGAGCATCAATTGTTTGAGCCGAGCGCAGAGAATATTCGTAATCAA 2724
Akaw_S  909   L  H  Q  Y  Y  A  D  E  L  F  A  P  D  R  Q  N  I  R  V  R   928
Clau_S  909   L  H  Q  Y  Y  A  D  E  L  F  A  P  D  R  Q  N  I  R  V  R   928
Pkil_S  909   L  H  Q  Y  Y  A  D  E  L  F  S  P  D  R  Q  N  I  R  V  R   928
Psoy_S  909   L  H  Q  Y  Y  A  D  E  L  F  S  P  D  R  Q  N  I  R  V  R   928
Vok     909   L  H  Q  Y  Y  A  N  E  L  F  S  P  D  R  Q  N  I  R  V  R   928
Cpac_S  905   L  Y  Q  Y  Y  E  N  G  L  F  S  P  D  K  Q  S  I  R  V  R   924
Cfau_S  905   L  H  Q  Y  Y  E  D  G  L  F  S  P  D  R  Q  S  I  S  V  R   924
Cnau_S  905   L  H  Q  Y  Y  E  D  G  L  F  S  P  D  R  Q  S  I  S  V  R   924
Pste_S  905   L  H  Q  Y  Y  E  D  G  L  F  L  P  D  R  Q  S  I  S  V  R   924
Rma     906   L  H  Q  Y  Y  E  D  S  L  F  S  P  D  K  Q  S  I  R  A  R   925
Ifos_S  909   L  H  Q  Y  Y  E  D  G  L  F  S  P  D  K  Q  S  I  R  V  R   928
Apha_S  910   L  H  Q  Y  Y  E  D  G  L  F  S  P  E  K  Q  S  I  R  A  R   929
Bsep_S  889   V  H  Q  Y  Y  E  H  Q  L  F  E  P  S  A  E  N  I  R  N  Q   908
```

```
Akaw_S 2785 TTGGTTGAATACGGTATTGGTAATATGGATATAGATGACTCTATAGATTTTATTATTAAT 2844
Clau_S 2785 TTGGTTGAATGTGGTATTGGTAGTATGGATATAGATGATTCTATAGACTTTATTATTAAT 2844
Pkil_S 2785 TTGGTTGAATTCGGTATTAGTAATATGGATATAGATGATTCTATAGACTTTATTATTGAT 2844
Psoy_S 2785 TTGGTTGAATTCGGTATTAGTAATATGGATATAGATGATTCTATAGACTTTATTATTGAT 2844
Vok    2785 TTGGTTGAATTCGGTATTAGTAATATGGATATAGATGATTCTATAGACTTTATTATTGAT 2844
Cpac_S 2773 TTGGTGGAATACGGTGTTGGCAATATGGATATTGACACTTATATCGATTTTATTGTCAAC 2832
Cfau_S 2773 TTGGTGGAATGCGGTGTTGGCAATACGGATATTGACAGTTATATCGATTTTATTGTCAAC 2832
Cnau_S 2773 TTGGTGGAATTCGGTGTTGGCAATATGGATATTGACAGTTATATCGATTTTATTGTCAAC 2832
Pste_S 2773 TTGGTGGAATGTGGTGTTGGCAATATGGATATTGATAGTTATATCGATTTTATTGTCAAC 2832
Rma    2776 CTAATGGAGTGTGGTGTTGGTAATATGGATATTGACAGTCATATCAATTTTATTGTCAAT 2835
Ifos_S 2785 CTAATGGAGTGTGGTGTTGGTAACACGGACATTGACAGTCATATCGATTTTATTGTCAAC 2844
Apha_S 2788 CTAATGGAGTGTGGTGTTGGTAATATGGACATTGACAGTCATATCGATTTTATTGTCAAC 2847
Bsep_S 2725 TTGATTAGTATAGGCACGCCGCCGAGTGAGATTGAAAAATGGCAGGCGGTTATCACTAAA 2784
Akaw_S  929   L  V  E  Y  G  I  G  N  M  D  I  D  D  S  I  D  F  I  I  N   948
Clau_S  929   L  V  E  C  G  I  G  S  M  D  I  D  D  S  I  D  F  I  I  N   948
Pkil_S  929   L  V  E  F  G  I  S  N  M  D  I  D  D  S  I  D  F  I  I  D   948
Psoy_S  929   L  V  E  F  G  I  S  N  M  D  I  D  D  S  I  D  F  I  I  D   948
Vok     929   L  V  E  F  G  I  S  N  M  D  I  D  D  S  I  D  F  I  I  D   948
Cpac_S  925   L  V  E  Y  G  V  G  N  M  D  I  D  T  Y  I  D  F  I  V  N   944
Cfau_S  925   L  V  E  C  G  V  G  N  T  D  I  D  S  Y  I  D  F  I  V  N   944
Cnau_S  925   L  V  E  F  G  V  G  N  M  D  I  D  S  Y  I  D  F  I  V  N   944
Pste_S  925   L  V  E  C  G  V  G  N  M  D  I  D  S  Y  I  D  F  I  V  N   944
Rma     926   L  M  E  C  G  V  G  N  M  D  I  D  S  H  I  N  F  I  V  N   945
Ifos_S  929   L  M  E  C  G  V  G  N  T  D  I  D  S  H  I  D  F  I  V  N   948
Apha_S  930   L  M  E  C  G  V  G  N  M  D  I  D  S  H  I  D  F  I  V  N   949
Bsep_S  909   L  I  S  I  G  T  P  P  S  E  I  E  K  W  Q  A  V  I  T  K   928
```

```
Akaw_S 2845 ATGCTTAATTTAACGAAACAAGATAAATACTTTTCGTGGTTGTTTAAACAAAGAGTATCA 2904
Clau_S 2845 ATGCTTAATTTAACGAAACAAGATAAATACTTTTCGTGGTTGTTTAAACAAAGAGTATCA 2904
Pkil_S 2845 ATGCTTAATTTAACGAAAGAAGATAAATACTTTTCGTGGTTGTTTAAACAAAGAGTATCA 2904
Psoy_S 2845 ATGCTTAATTTAACGAAAGAAGATAAATACTTTTCGTGGTTGTTTAAACAAAGAGTATCA 2904
Vok    2845 ATGCTTAATTTAACGAAACAAGATAAATACTTTTCGTGGTTGTTTAAACAAAGAGTATCA 2904
Cpac_S 2833 ATGCTTAATTTAACGAAACAAGACAAACATTTTTCTTGGCTGTTTAAACAAAGAACATCA 2892
Cfau_S 2833 ATGCTTAATTTAACGAAACAAGACAAACACTTTTCTTGGCTATTCAAACAAAGAACATCA 2892
Cnau_S 2833 ATGCTCAATTTAACGAAACAAGACAAACACTTTTCTTGGCTGTTCAAACAAAGAACATCA 2892
Pste_S 2833 ATGCTTAATTTAACGAAACAAGACAAACACTTTTCTTGGCTGTTCAAACAAAGAATATCA 2892
Rma    2836 ATGCTTAATTTAACGAAACAAGACAAACACTTTTCTTGGTTGTTTAAGCAAAGAACATCA 2895
Ifos_S 2845 ATGCTTAATTTAACGAAACAAGATAAACACTTTTCTTGGTTGTTTAAGCAAAGAATATCA 2904
Apha_S 2848 ATGCTTAATTTAACGAAACAAGACAAACACTTTTCTTGGTTGTTCAAACAAAGAACATCA 2907
Bsep_S 2785 TTATTGAATAACACCAAAAATGATGCTCAATTCGAGTGGCTGTTTAAAGACCGAGCATCC 2844
Akaw_S  949   M  L  N  L  T  K  Q  D  K  Y  F  S  W  L  F  K  Q  R  V  S  968
Clau_S  949   M  L  N  L  T  K  Q  D  K  Y  F  S  W  L  F  K  Q  R  V  S  968
Pkil_S  949   M  L  N  L  T  K  E  D  K  Y  F  S  W  L  F  K  Q  R  V  S  968
Psoy_S  949   M  L  N  L  T  K  E  D  K  Y  F  S  W  L  F  K  Q  R  V  S  968
Vok     949   M  L  N  L  T  K  Q  D  K  Y  F  S  W  L  F  K  Q  R  V  S  968
Cpac_S  945   M  L  N  L  T  K  Q  D  K  H  F  S  W  L  F  K  Q  R  T  S  964
Cfau_S  945   M  L  N  L  T  K  Q  D  K  H  F  S  W  L  F  K  Q  R  T  S  964
Cnau_S  945   M  L  N  L  T  K  Q  D  K  H  F  S  W  L  F  K  Q  R  T  S  964
Pste_S  945   M  L  N  L  T  K  Q  D  K  H  F  S  W  L  F  K  Q  R  I  S  964
Rma     946   M  L  N  L  T  K  Q  D  K  H  F  S  W  L  F  K  Q  R  T  S  965
Ifos_S  949   M  L  N  L  T  K  Q  D  K  H  F  S  W  L  F  K  Q  R  I  S  968
Apha_S  950   M  L  N  L  T  K  Q  D  K  H  F  S  W  L  F  K  Q  R  T  S  969
Bsep_S  929   L  L  N  N  T  K  N  D  A  Q  F  E  W  L  F  K  D  R  A  S  948
```

```
Akaw_S 2905 ACCCAGGTTGAAGTAGAATTTATTAATGACAAACGTAATATTATTATTGATCGATTATTT 2964
Clau_S 2905 ACCCAGGTTGAAGTAGAATTTATTAATGACAAACGTAACATTATTATTGATCGATTATTT 2964
Pkil_S 2905 ACCCAGGTTGAAGTAGAATTTGTTAATGACAAACGTAACATTATTATTGATCGATTATTT 2964
Psoy_S 2905 ACCCAGGTTGAAGTAGAATTTGTTAATGACAAACGTAACATTATTATTGATCGATTATTT 2964
Vok    2905 ACCCAGGTTGAAGTAGAATTTATTAATGACAAACGTAACATTATTATTGATCGATTATTT 2964
Cpac_S 2893 ACCCAAGTTGAGGCAGAATTTATAAGTGACAAGCGTAGCATTATTATTGATCGATTATTT 2952
Cfau_S 2893 ACCCAAGTTGAGGCAGAGTTTATAAGTGACAAGCGTAGCGTTATTATTGATCGATTATTT 2952
Cnau_S 2893 ACTAAAGTTGAGGCAGAGTTTATAAGTGACAAGCGTAGCGTTATTATTGATCGATTATTT 2952
Pste_S 2893 ACTCAAGTTGAGGCAGAGTTTATAAGTGACAAGCGTAGCGTTATTATTGATCGATTATTT 2952
Rma    2896 ACTCAAGTTGAGGTAGAATTTATAAATGACAAGCATAGTGTTATTATTGATCGATTATTT 2955
Ifos_S 2905 ACCCAAGTTGAGGCAGAATTTATAAGTCACAAGCACAGTGTTATTATTGATCGATTATTT 2964
Apha_S 2908 ACCCAAGTTGAGGCAGAGTTTATAAGTGACAAGTGTAGCGTTATTATTGATCGATTATTT 2967
Bsep_S 2845 GCACGAAATGAAGCGGAATTTAGCATTAATGGTAGCACCATCGCTATTGACAGACTATTT 2904
Akaw_S  969   T  Q  V  E  V  E  F  I  N  D  K  R  N  I  I  I  D  R  L  F  988
Clau_S  969   T  Q  V  E  V  E  F  I  N  D  K  R  N  I  I  I  D  R  L  F  988
Pkil_S  969   T  Q  V  E  V  E  F  V  N  D  K  R  N  I  I  I  D  R  L  F  988
Psoy_S  969   T  Q  V  E  V  E  F  V  N  D  K  R  N  I  I  I  D  R  L  F  988
Vok     969   T  Q  V  E  V  E  F  I  N  D  K  R  N  I  I  I  D  R  L  F  988
Cpac_S  965   T  Q  V  E  A  E  F  I  S  D  K  R  S  I  I  I  D  R  L  F  984
Cfau_S  965   T  Q  V  E  A  E  F  I  S  D  K  R  S  V  I  I  D  R  L  F  984
Cnau_S  965   T  K  V  E  A  E  F  I  S  D  K  R  S  V  I  I  D  R  L  F  984
Pste_S  965   T  Q  V  E  A  E  F  I  S  D  K  R  S  V  I  I  D  R  L  F  984
Rma     966   T  Q  V  E  V  E  F  I  N  D  K  H  S  V  I  I  D  R  L  F  985
Ifos_S  969   T  Q  V  E  A  E  F  I  S  H  K  H  S  V  I  I  D  R  L  F  988
Apha_S  970   T  Q  V  E  A  E  F  I  S  D  K  C  S  V  I  I  D  R  L  F  989
Bsep_S  949   A  R  N  E  A  E  F  S  I  N  G  S  T  I  A  I  D  R  L  F  968
```

PDDEXK_1 domain (980-1031)

```
Akaw_S 2965 ATTGATAAAGGCACTTTATGGATTATTGATTTTAAAACTGAAAGACAAGTAAATAATGAG 3024
Clau_S 2965 ATTGATGAAGGCGCTTTGTGGGTTATTGATTTTAAAACTGAAAGACAAGTAAATAATGAG 3024
Pkil_S 2965 ATTGATGAAGGCACTTTGTGGGTTATTGATTTTAAAACTGAAAGACAAGTAAATAATGAG 3024
Psoy_S 2965 ATTGATGAAGGCACTTTGTGGGTTATTGATTTTAAAACTGAAAGACAAGTAAATAATGAG 3024
Vok    2965 ATTGATGAAGGCATTTTGTGGGTTATTGATTTTAAAACTGAAAGACAAGTAAATAATGAG 3024
Cpac_S 2953 ATTGATGAAGATATTTTGTGGATTATTGATTTTAAAACCACAAGACAAGCGAATGATGAA 3012
Cfau_S 2953 ATTGATGAAGGTATTTTGTGGATTATTGATTTTAAAACCACAAGACAAGCGAATGATGAA 3012
Cnau_S 2953 ATTGATGAAGATATTTTGTGGATTATTGATTTTAAAACCACAAGACAAGCGAATAATGAA 3012
Pste_S 2953 ATTGATGAAGATATTTTGTGGATTATTGATTTTAAAACCACAAGACAAGCGAATGATGAG 3012
Rma    2956 ATTGATGAAGACACTTTGTGGATTATTGATTTTAAAACTGCAACGAAATCGAATAATGAA 3015
Ifos_S 2965 ATTGATGAAGATACTTTGTGGATTATTGATTTTAAAACTGCAACGAAATCGAATGATGAA 3024
Apha_S 2968 ATTGATGAAGATATTTTGTGGATTATTGATTTTAAAACCACAAGACAAACGAATGATGAA 3027
Bsep_S 2905 ATTGATGAGGGGACTTTATGGGTGATTGATTTTAAAACAGCCAAACCTGCTGAGGATGAG 2964
Akaw_S  989   I  D  K  G  T  L  W  I  I  D  F  K  T  E  R  Q  V  N  N  E 1008
Clau_S  989   I  D  E  G  A  L  W  V  I  D  F  K  T  E  R  Q  V  N  N  E 1008
Pkil_S  989   I  D  E  G  T  L  W  V  I  D  F  K  T  E  R  Q  V  N  N  E 1008
Psoy_S  989   I  D  E  G  T  L  W  V  I  D  F  K  T  E  R  Q  V  N  N  E 1008
Vok     989   I  D  E  G  I  L  W  V  I  D  F  K  T  E  R  Q  V  N  N  E 1008
Cpac_S  985   I  D  E  D  I  L  W  I  I  D  F  K  T  T  R  Q  A  N  D  E 1004
Cfau_S  985   I  D  E  G  I  L  W  I  I  D  F  K  T  T  R  Q  A  N  D  E 1004
Cnau_S  985   I  D  E  D  I  L  W  I  I  D  F  K  T  T  R  Q  A  N  N  E 1004
Pste_S  985   I  D  E  D  I  L  W  I  I  D  F  K  T  T  R  Q  A  N  D  E 1004
Rma     986   I  D  E  D  T  L  W  I  I  D  F  K  T  A  T  K  S  N  N  E 1005
Ifos_S  989   I  D  E  D  T  L  W  I  I  D  F  K  T  A  T  K  S  N  D  E 1008
Apha_S  990   I  D  E  D  I  L  W  I  I  D  F  K  T  T  R  Q  T  N  D  E 1009
Bsep_S  969   I  D  E  G  T  L  W  V  I  D  F  K  T  A  K  P  A  E  D  E  988
```

PDDEXK_1 domain (980-1031)

```
Akaw_S 3025 TCAATAGTACAATTTATACAAAGGCAAAAAATTAAACATAGCCATCAATTATTATTTTAT 3084
Clau_S 3025 TCAATAGTACAATTTATACAAAGGCAAAAAATTAAACACAGCCATCAATTGTTATTTTAT 3084
Pkil_S 3025 TCAATAGTACAATTTATACAAAGGCAAAAAATTAAACATAGCCATCAATTATTATTTTAT 3084
Psoy_S 3025 TCAATAGTACAATTTATACAAAGGCAAAAAATTAAACATAGCCATCAATTATTATTTTAT 3084
Vok    3025 TCAATAGTACAATTTATACAAAGGCAAAAAATTAAACATAGCCATCAATTATTATTTTAT 3084
Cpac_S 3013 TCAATAGCACAATTTATACAAAGACAAAAAGCCAAATACACTCAACAATTATTATCCTAT 3072
Cfau_S 3013 TCAATAGCACAATTTATACAAAGGCAAAAAGCCAAACACACTCAACAATTATTATCCTAT 3072
Cnau_S 3013 TCAATAGCACAATTTATACAAAGGCAAAAAATCAAACATACTCAGCAATTACTATCCTAT 3072
Pste_S 3013 TCAATAGCACAATTTATACAAAGGCAAAAAACCAAACATACTCAACAATTATTATTCTAT 3072
Rma    3016 TCAATAGCGCAATTTATACAAAGACAAAAAATCAAATATACTCAACAATTGTTGTCTTAT 3075
Ifos_S 3025 TCAATCGCACAATTTATACAAAGGCAAAAAACCAAATATACGCAACAATTATTGTCCTAT 3084
Apha_S 3028 TCAATAGCACAATTTATACAAAGTCAAAAAACCAAACATACGAAACAATTATTGTCCTAT 3087
Bsep_S 2965 CCATTAGAAGCCTTTATTAAACGACAACAGCAAGAACACACAAAACAATTGCAGTTCTAT 3024
Akaw_S 1009   S  I  V  Q  F  I  Q  R  Q  K  I  K  H  S  H  Q  L  L  F  Y 1028
Clau_S 1009   S  I  V  Q  F  I  Q  R  Q  K  I  K  H  S  H  Q  L  L  F  Y 1028
Pkil_S 1009   S  I  V  Q  F  I  Q  R  Q  K  I  K  H  S  H  Q  L  L  F  Y 1028
Psoy_S 1009   S  I  V  Q  F  I  Q  R  Q  K  I  K  H  S  H  Q  L  L  F  Y 1028
Vok    1009   S  I  V  Q  F  I  Q  R  Q  K  I  K  H  S  H  Q  L  L  F  Y 1028
Cpac_S 1005   S  I  A  Q  F  I  Q  R  Q  K  A  K  Y  T  Q  Q  L  L  S  Y 1024
Cfau_S 1005   S  I  A  Q  F  I  Q  R  Q  K  A  K  H  T  Q  Q  L  L  S  Y 1024
Cnau_S 1005   S  I  A  Q  F  I  Q  R  Q  K  I  K  H  T  Q  Q  L  L  S  Y 1024
Pste_S 1005   S  I  A  Q  F  I  Q  R  Q  K  T  K  H  T  Q  Q  L  L  F  Y 1024
Rma    1006   S  I  A  Q  F  I  Q  R  Q  K  I  K  Y  T  Q  Q  L  L  S  Y 1025
Ifos_S 1009   S  I  A  Q  F  I  Q  R  Q  K  T  K  Y  T  Q  Q  L  L  S  Y 1028
Apha_S 1010   S  I  A  Q  F  I  Q  S  Q  K  T  K  H  T  K  Q  L  L  S  Y 1029
Bsep_S  989   P  L  E  A  F  I  K  R  Q  Q  Q  E  H  T  K  Q  L  Q  F  Y 1008
```

PDDEXK_1 domain (980-1031)

```
Akaw_S 3085 AAAGTGACTTTATCAAAATGTTATTCAATGAAGGTAAAGTGCGCTTTATATTGCCCGGCA 3144
Clau_S 3085 AAAGTGATTTTAACAGAATGTTATTCAATGGAGGTAAAGTGCGCTTTATATTGTCCAGCA 3144
Pkil_S 3085 AAAGTGACTTTATCAGAATGTTATTCAATGGAGGTAAAGTGCGCTTTATATTGTCCAGCA 3144
Psoy_S 3085 AAAGTGACTTTATCAGAATGTTATTCAATGGAGGTAAAGTGCGCTTTATATTGTCCAGCA 3144
Vok    3085 AAAGTGACTTTATCAGAATGTTATTCAATGGAGGTAAAGTGCGCTTTATATTGTCCATCA 3144
Cpac_S 3073 AAAGTGGTTTTATCAGCGTGTTATTCAATGGAAATAAAGTGTGTTCTATATTGTCCAGCA 3132
Cfau_S 3073 AAAGTGGCTTTATCAGCGTGTTATTCAATGGAAATAAAGTGTGTTCTATATTATCCAGCA 3132
Cnau_S 3073 AAAGTGGTTTTATCAGCGTGTTATTCAATGGAAATAAAGTGTGCTTTATATTGTCCAGCA 3132
Pste_S 3073 AAAGTGGTTTTATCAGCGTGTTATTCAATGGAAATAAAGTGTGCTTTATATTGTCCAGCG 3132
Rma    3076 AAGGTAGCTTTATCAGAGTATTATTCAATGGAAATAAAATGTGCTTTATATTGTTCATCA 3135
Ifos_S 3085 AAAGTGGCTTTATCAGCGTGTTATTCAATGGAAATCAAGTGCGCTTTGTATTGCCCAGCA 3144
Apha_S 3088 AAAGTGGTTTTATCAGCGTGTTATTCAATGGAAATAAAGTGCGCTTTGTATTGTCCAGCA 3147
Bsep_S 3025 AAAACCGCAATGTCTGAGATTTACGACTATCCTGTGCGTTGCGCACTATATTGCCCTAGC 3084
Akaw_S 1029   K  V  T  L  S  K  C  Y  S  M  K  V  K  C  A  L  Y  C  P  A 1048
Clau_S 1029   K  V  I  L  T  E  C  Y  S  M  E  V  K  C  A  L  Y  C  P  A 1048
Pkil_S 1029   K  V  T  L  S  E  C  Y  S  M  E  V  K  C  A  L  Y  C  P  A 1048
Psoy_S 1029   K  V  T  L  S  E  C  Y  S  M  E  V  K  C  A  L  Y  C  P  A 1048
Vok    1029   K  V  T  L  S  E  C  Y  S  M  E  V  K  C  A  L  Y  C  P  S 1048
Cpac_S 1025   K  V  V  L  S  A  C  Y  S  M  E  I  K  C  V  L  Y  C  P  A 1044
Cfau_S 1025   K  V  A  L  S  A  C  Y  S  M  E  I  K  C  V  L  Y  Y  P  A 1044
Cnau_S 1025   K  V  V  L  S  A  C  Y  S  M  E  I  K  C  A  L  Y  C  P  A 1044
Pste_S 1025   K  V  V  L  S  A  C  Y  S  M  E  I  K  C  A  L  Y  C  P  A 1044
Rma    1026   K  V  A  L  S  E  Y  Y  S  M  E  I  K  C  A  L  Y  C  S  S 1045
Ifos_S 1029   K  V  A  L  S  A  C  Y  S  M  E  I  K  C  A  L  Y  C  P  A 1048
Apha_S 1030   K  V  V  L  S  A  C  Y  S  M  E  I  K  C  A  L  Y  C  P  A 1049
Bsep_S 1009   K  T  A  M  S  E  I  Y  D  Y  P  V  R  C  A  L  Y  C  P  S 1028
```

PDDEXK_1 domain (980-1031)

```
Akaw_S 3145 GTACAAGAATTGATTAGAATTGGC---------------------TAA 3171
Clau_S 3145 GTACAAGAATTGATTAGAATTAGC---------------------TAA 3171
Pkil_S 3145 GTACAAAAATTGATTAGAATTGAC---------------------TAA 3171
Psoy_S 3145 GTACAAAAATTGATTAGAATTGAC---------------------TAA 3171
Vok    3145 GTACAAAAATTGATTAGAATTGAC---------------------TAA 3171
Cpac_S 3133 GTACAAGAATTGATTGAAATTGCC---------------------TAA 3159
Cfau_S 3133 GTACAAGAATTGATTGAAATTGGC---------------------TAA 3159
Cnau_S 3133 GTACAAGAATTGATTGAAATTGGC---------------------TAA 3159
Pste_S 3133 ATACAAGAATTGATTGAAATTGAC---------------------TAA 3159
Rma    3136 GTACAAGAGTTAATTGAAATTTAC---------------------TAA 3162
Ifos_S 3145 GTGCAAGAATTGATTGAAATTGACTCAATGAAATCTTTGCATAAATAA 3192
Apha_S 3148 GTGCAAGAATTGATTGAAATTGAT---------------------TAG 3174
Bsep_S 3085 GTCAAACAATTAATCGACATTTCC---------------------TAA 3111
Akaw_S 1049   V  Q  E  L  I  R  I  G  #  #  #  #  #  #  #  * 1057
Clau_S 1049   V  Q  E  L  I  R  I  S  #  #  #  #  #  #  #  * 1057
Pkil_S 1049   V  Q  K  L  I  R  I  D  #  #  #  #  #  #  #  * 1057
Psoy_S 1049   V  Q  K  L  I  R  I  D  #  #  #  #  #  #  #  * 1057
Vok    1049   V  Q  K  L  I  R  I  D  #  #  #  #  #  #  #  * 1057
Cpac_S 1045   V  Q  E  L  I  E  I  A  #  #  #  #  #  #  #  * 1053
Cfau_S 1045   V  Q  E  L  I  E  I  G  #  #  #  #  #  #  #  * 1053
Cnau_S 1045   V  Q  E  L  I  E  I  G  #  #  #  #  #  #  #  * 1053
Pste_S 1045   I  Q  E  L  I  E  I  D  #  #  #  #  #  #  #  * 1053
Rma    1046   V  Q  E  L  I  E  I  Y  #  #  #  #  #  #  #  * 1054
Ifos_S 1049   V  Q  E  L  I  E  I  D  S  M  K  S  L  H  K  * 1064
Apha_S 1050   V  Q  E  L  I  E  I  D  #  #  #  #  #  #  #  * 1058
Bsep_S 1029   V  K  Q  L  I  D  I  S  #  #  #  #  #  #  #  * 1037
```
